# Scalable Access to *N*‑Acylindole Linkages: Enabling the Synthesis of Antitrypanosomal Noncanonical Cyclic Peptides for Chagas Disease

**DOI:** 10.1002/anie.202526149

**Published:** 2026-02-27

**Authors:** Jie Zhang, Hugh Nakamura

**Affiliations:** ^1^ Department of Chemistry The Hong Kong University of Science and Technology (HKUST) Hong Kong SAR China

**Keywords:** Chagas disease, infectious disease, neglected tropical diseases (NTDs), noncanonical cyclic peptide, parasites

## Abstract

Chagas disease is classified as a neglected tropical disease (NTD) and is predominantly endemic in South America. Despite advances in modern medicine, more than 10 million people worldwide are currently infected, and approximately 12 000 deaths are reported annually. The World Health Organization (WHO) aims to eliminate Chagas disease by 2030. Although more than 100 years have passed since the discovery of Chagas disease, no effective vaccine has yet been developed. The only available chemotherapeutic agents are nifurtimox and benznidazole, both of which were introduced more than half a century ago and are included in the WHO's list of essential medicines. However, because these drugs are associated with severe adverse effects and are effective only during the early phase of infection, they have not achieved eradication of Chagas disease. In this study, a scalable synthetic method was developed for noncanonical cyclic peptides bearing a rare *N*‐acylindole linkage, a structural motif not typically observed in conventional cyclic peptides. This strategy enabled the synthesis of bulbiferamide A, a potent antitrypanosomal agent reported to be effective against Chagas disease, as well as a variety of related analogues that had previously been difficult to access synthetically.

## Graphical abstract

A gram‐scale supply of bulbiferamide A, which features a rare *N*‐acylindole linkage and possesses potent inhibitory activity against *Trypanosoma cruzi* epimastigotes (IC_50_ = 4.1 µM)—the causative parasite of Chagas disease—was successfully achieved. Moreover, the cyclization strategy developed in this study facilitated the synthesis of noncanonical cyclic peptides bearing *N*‐acylindole linkages across a range of ring sizes.

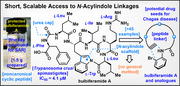

## 1 Introduction

**FIGURE 1:**
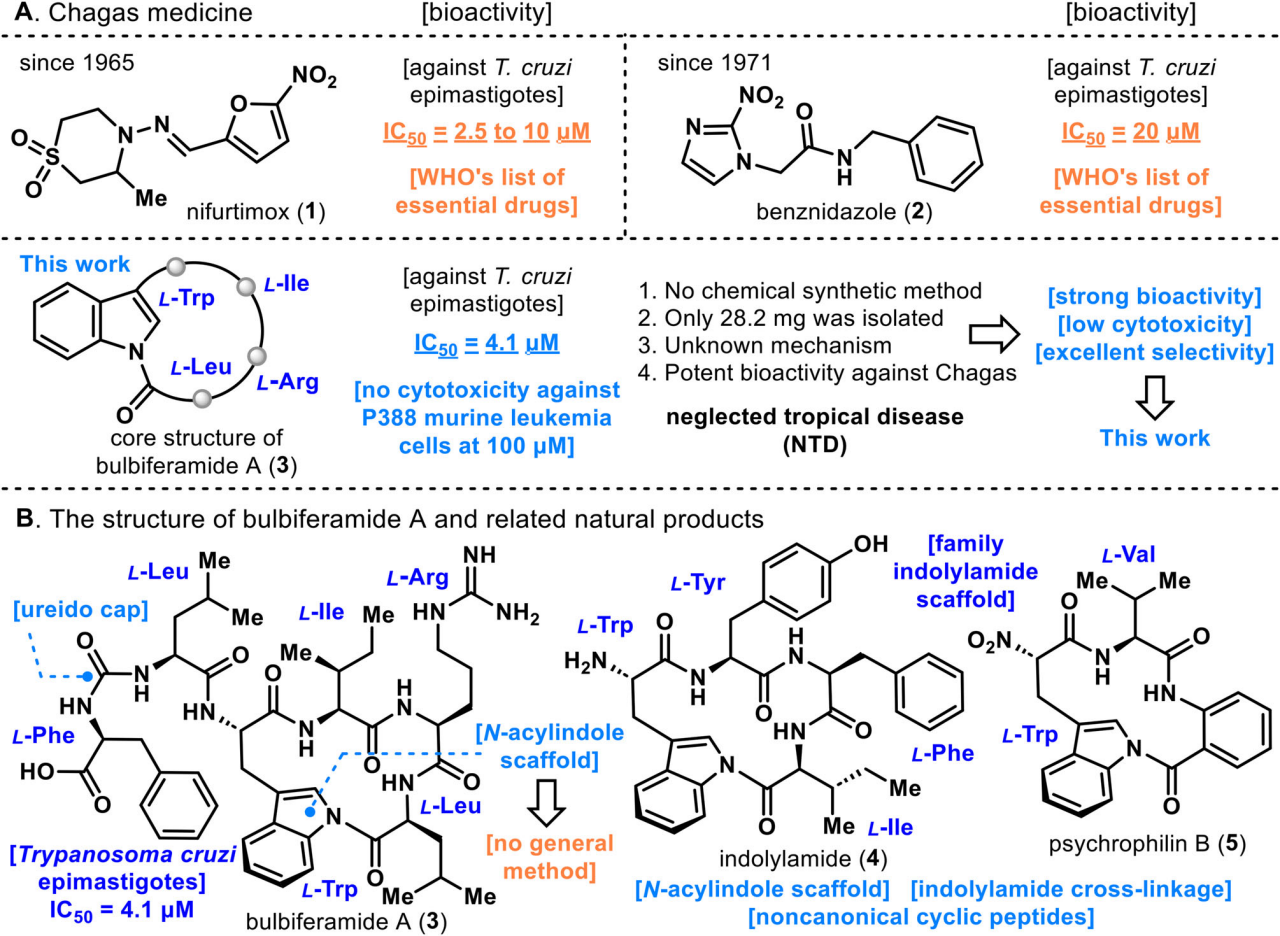
(A) Chagas medicine. (B) The structure of bulbiferamide A (**3**) and related natural products.

Chagas disease, designated by the WHO as one of the neglected tropical diseases (NTDs) targeted for eradication, was first identified in South America in 1909 [1]. The disease is transmitted by triatomine insects (commonly referred to as “kissing bugs”), which serve as vectors for the protozoan parasite *Trypanosoma cruzi* that invades the human host [2, 3, 4]. It is estimated that more than 10 million individuals, predominantly in Latin America, are currently infected with Chagas disease, while nearly 80 million are at risk of infection [5, 6, 7, 8]. Each year, approximately 12,000 deaths and 40,000 new infections are reported [9, 10]. As therapeutic agents for Chagas disease, nifurtimox (**1**) and benznidazole (**2**), both commercialized in the 1960s and 1970s and listed among the WHO's essential medicines, are recognized as the established treatments (Figure 1A) [11]. However, these drugs were developed more than half a century ago and present several limitations [12, 13, 14, 15, 16, 17, 18, 19, 20, 21, 22, 23, 24, 25, 26, 27].

Bulbiferamide A (**3**) is a novel cyclic peptide that was independently isolated, structurally elucidated, and reported in 2023 by the Igarashi and Agarwal groups from marine obligate bacteria (Figure 1B) [28, 29]. A distinctive structural feature of bulbiferamide A (**3**) is the presence of an exceptionally rare *N*‐acylindole linkage within the oligomeric peptide ring, a motif not typically observed in cyclic peptides. The *N*‐acylindole framework itself is found in more than one hundred indole alkaloids [30]. However, prior to the discovery of the psychrophilins [31, 32, 33], no natural products had been reported that possess an *N*‐acylindole linkage embedded within an oligomeric peptide ring. Thus, although the *N*‐acylindole linkage in bulbiferamide A (**3**) appears structurally simple, its occurrence is exceedingly uncommon. Moreover, the inherently low nucleophilicity of the indole NH group, combined with the intrinsic instability of *N*‑acylindoles to nucleophilic attack, restricts the development of general synthetic methodologies for constructing this linkage [34, 35, 36, 37, 38, 39, 40, 41, 42, 43, 44, 45, 46].

To date, the sole means of accessing *N*‐acylindole linkages has relied on enzymatic processes [47, 48, 49]. Other compounds containing this motif include indolylamide (**4**), which was artificially generated using enzymatic methods [50]. Prior to the discovery of bulbiferamide A (**3**), psychrophilin B (**5**) represented the only known oligomeric cyclic peptide possessing an intramolecular *N*‐acylindole linkage [32]. It is also noteworthy that bulbiferamide A (**3**) bears a ureido functionality at its *N*‐terminus. Given the pronounced electrophilicity of the *N*‐acylindole moiety, a free terminal amino group could undergo *N,N*‐acyl transfer, potentially resulting in cleavage of the *N*‐acylindole linkage. It is therefore hypothesized that the *N*‐terminus of the amino acid is “ureido‐capped” during biosynthesis to prevent such degradation, thereby enhancing the stability of the otherwise labile *N*‐acylindole linkage.

With respect to biological activity, bulbiferamide A (**3**) exhibits potent trypanocidal effects against *T. cruzi* epimastigotes, the causative parasite of Chagas disease, with an IC_50_ value of 4.1 µM [28]. This activity is markedly stronger than that of the current standard drug benznidazole (**2**), which displays an IC_50_ of 20 µM [28, 51, 52]. Importantly, bulbiferamide A (**3**) demonstrates negligible cytotoxicity toward mammalian cells, showing no activity against P388 murine leukemia cells even at 100 µM [28]. Consequently, bulbiferamide A (**3**) exhibits substantially superior selectivity compared to the established drugs nifurtimox (**1**) and benznidazole (**2**). Taken together, bulbiferamide A (**3**) represents an ideal potential lead compound for Chagas disease therapy, combining potent antiparasitic activity with minimal mammalian cytotoxicity. However, due to the difficulty associated with constructing an *N*‐acylindole linkage within an oligopeptide ring, no total synthesis of bulbiferamide A (**3**) has been reported to date.

**FIGURE 2:**
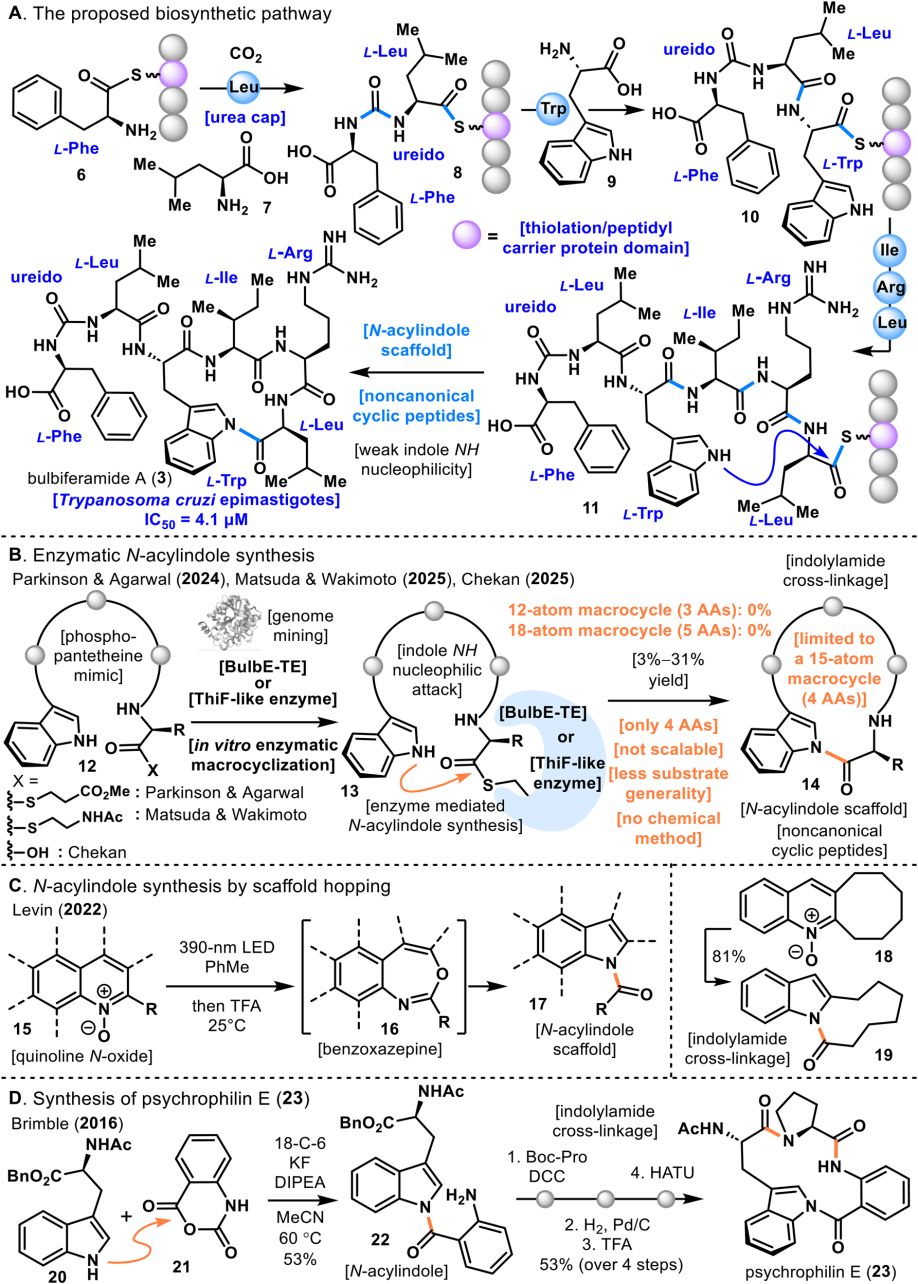
(A) The proposed biosynthetic pathway. (B) Enzymatic *N‐*acylindole synthesis. (C) *N*‐acylindole synthesis by scaffold hopping. (D) Synthesis of psychrophilin E (**23**).

The proposed biosynthetic pathway of bulbiferamide A (**3**) is illustrated in Figure 2A [47]. Bulbiferamide A (**3**) is believed to be assembled by a biosynthetic gene cluster present in marine bacteria of the genus *Microbulbifer*, which constructs six peptide units. The elongation of the peptide chain is thought to initiate with Phe (phenylalanine), followed by the formation of a ureido structure with Leu**7** (leucine). This ureido unit, composed of two amino acids, is subsequently converted into thioester **8** by the thiolation domain. Trp**9** (tryptophan) is then incorporated into intermediate **8**, which after thioesterification is transformed into **10**. Through analogous elongation steps, Ile (isoleucine), Arg (arginine), and Leu are sequentially introduced, yielding intermediate **11** with a linear hexapeptide structure. Finally, nucleophilic attack by the indole NH group constructs the *N*‐acylindole linkage, resulting in the biosynthesis of the cyclic peptide bulbiferamide A (**3**). In general, *N*‐acylindole linkages are unstable toward nucleophilic functionalities such as amines due to their pronounced electrophilicity; however, as noted above, the ureido capping of the terminal amino group is hypothesized to contribute to the stabilization of the *N*‐acylindole linkage in bulbiferamide A (**3**).

Even with the advances of modern organic synthesis, methods for supplying compounds bearing intramolecular *N*‐acylindole linkages within oligomeric cyclic peptides remain extremely limited and largely dependent on enzymatic approaches [47, 48, 49, 50]. In 2024, Parkinson and Agarwal reported the enzymatic generation of cyclic peptide **14** containing an *N*‐acylindole linkage by mimicking biosynthesis (Figure 2B) [47]. They expressed the BulbE‐terminal thioesterase (TE) domain, identified in the genome of *Microbulbifer* sp., a marine obligate bacterium producing bulbiferamide A (**3**), in *E. coli*. Using this expressed BulbE‐TE, they attempted cyclization of linear peptide **12**, which had been chemically synthesized to bear thioester and indole termini. As a result, cyclic peptide 14 containing an intramolecular *N*‐acylindole linkage was obtained in yields ranging from 3% to 31%. During this process, intermediate **13**, in which BulbE‐TE and linear peptide **12** were connected via a thioester bond, was shown to be indispensable for *N*‐acylindole linkage formation. These findings demonstrated that BulbE‐TE is essential for the cyclization step in the biosynthesis of bulbiferamide A (3).

However, this enzymatic method was limited to constructing a 15‐atom macrocycle composed of four amino acids, presumably due to the restricted flexibility of the enzyme pocket accommodating the substrate. Indeed, attempts by Parkinson and Agarwal to generate 12‐atom macrocycles (three amino acids) or 18‐atom macrocycles (five amino acids) using BulbE‐TE were unsuccessful. In 2025, Matsuda and Wakimoto similarly employed BulbE‐TE as a cyclization enzyme to construct *N*‐acylindole linkage **14**, and revealed that the use of SNAC (*N*‐acetyl cysteamine) at the thioester site of precursor **12** was highly effective in promoting cyclization [48]. In parallel, Chekan and co‐workers in 2025 demonstrated for the first time that ThiF‐like enzymes catalyze macrocyclization via *N*‐acylindole linkage formation in ribosomally synthesized and post‐translationally modified peptides (RiPPs) [50]. Prior to this, macrocyclization through *N*‐acylindole linkage formation had only been reported in nonribosomal peptide synthetase (NRPS)‐derived natural products (bulbiferamides, psychrophilins), with no examples in RiPPs. Taken together, the enzymatic macrocyclizations reported by Parkinson and Agarwal, Matsuda and Wakimoto, and Chekan et al. successfully afforded target compounds, but were restricted to 15‐atom macrocycles composed of four amino acids, and moreover were not scalable [47, 48, 50].

Although limited, several chemical synthetic approaches to macrocycles containing *N*‐acylindole linkages have been reported (Figure 2C, D). In 2022, Levin and coworkers described a photochemical scaffold hopping strategy using quinoline *N*‐oxide (**15**) [53], in which a seven‐membered ring motif **16** was generated in situ and subsequently processed to yield *N*‐acylindole (**17**). This method was shown to be applicable to a variety of substrates, and the scaffold hopping approach was further extended to macrocycle synthesis. Application of the reaction conditions to quinoline *N*‐oxide (**18**) containing an eight‐membered ring afforded macrocycle **19** bearing an *N*‐acylindole linkage in 81% yield. In 2016, Brimble and coworkers reported the total synthesis of psychrophilin E (**23**) [54]. Heating tryptophan derivative **20** with an anhydride derivative **21** under basic conditions at 60 °C afforded the *N*‐acylindole (**22**). Subsequent functional group transformations, followed by amide‐coupling‐mediated macrocyclization, ultimately enabled the total synthesis of psychrophilin E (**23**).

## 2 Results and Discussion

**FIGURE 3:**
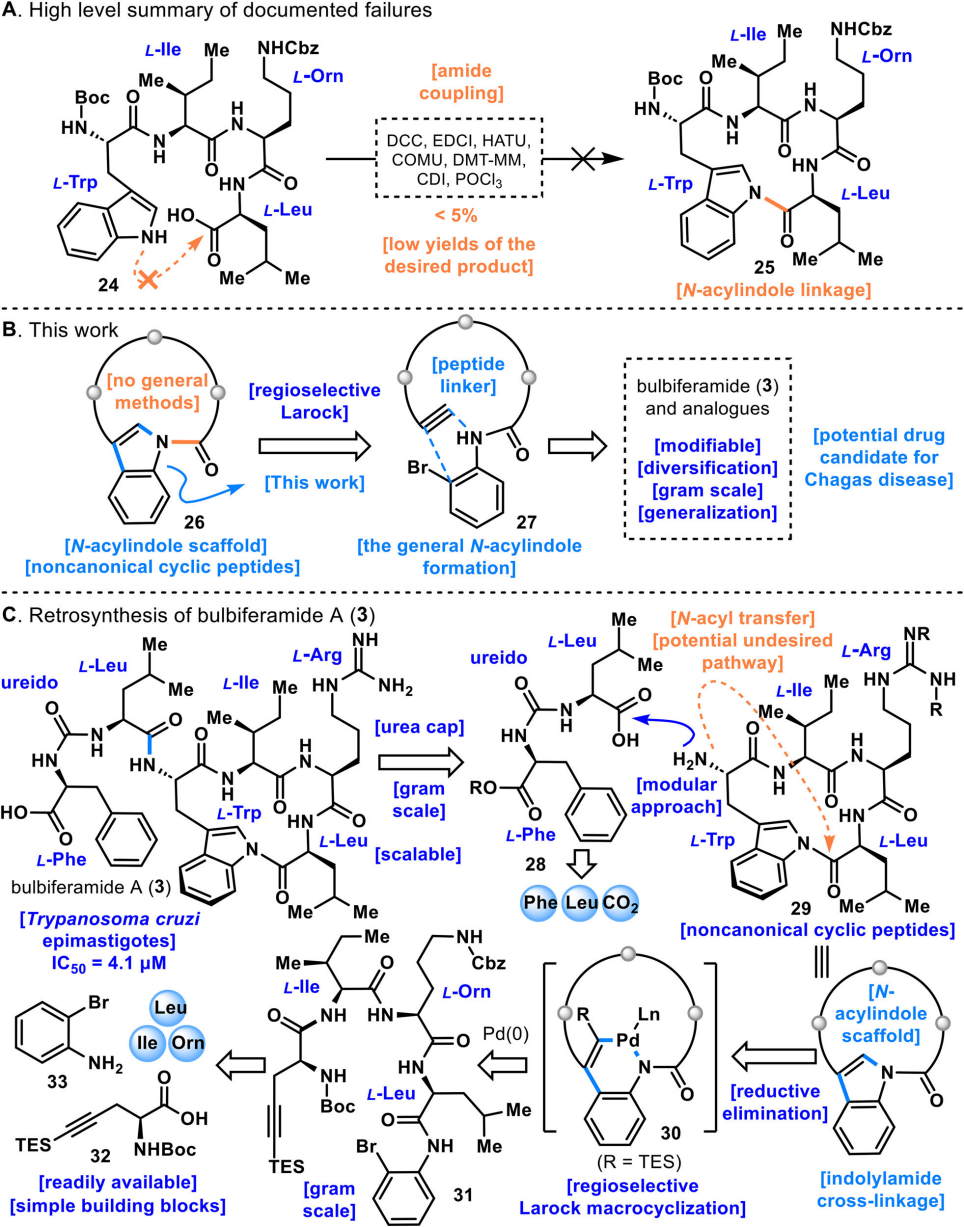
(A) High level summary of documented failures. (B) This work. (C) Retrosynthesis of bulbiferamide A (**3**).

We aimed to develop a general and scalable synthetic method for constructing the *N*‑acylindole linkage and to establish a supply route for bulbiferamide A (**3**), which exhibits potent inhibitory activity against *T. cruzi* epimastigotes, the causative parasite of Chagas disease (IC_50_ = 4.1 µM) (Figure 3) [28]. Initially, the construction of *N*‑acylindole linkage **25** from the linear peptide **24** was attempted under conventional amide coupling conditions (Figure 3A). Various standard coupling reagents, including *N,N*′‑diisopropylcarbodiimide (DIC), *N,N*′‐dicyclohexylcarbodiimide (DCC), 1‐(3‐dimethylaminopropyl)‐3‐ethylcarbodiimide hydrochloride (EDCI), hexafluorophosphate azabenzotriazole tetramethyl uronium (HATU), 1‐[(1‐(cyano‐2‐ethoxy‐2‐oxoethylideneaminooxy)‐dimethylamino‐morpholino)] uronium hexafluorophosphate (COMU), 4‐(4,6‐dimethoxy‐1,3,5‐triazin‐2‐yl)‐4‐methyl morpholinium chloride (DMT‑MM), and carbonyldiimidazole (CDI), were examined for the synthesis of *N*‑acylindole linkage **25**. However, none of these conditions afforded the desired product, and only trace amounts (<5%) of **25** were observed when HATU was employed. In addition, cyclization was attempted by generating the acid chloride of precursor **24** using POCl_3_, but this condition also failed to yield the desired cyclized product **25** (for further details, see Supporting Information). The lack of progress in the cyclization reaction is presumed to arise from the markedly weak nucleophilicity of the indole nitrogen, whose lone pair contributes to aromaticity and is delocalized within the π‑conjugated system. Indeed, in most of the conditions tested, the reaction was found to stall at the activated ester stage. These observations clearly indicate that although the carboxylic acid of linear peptide **24** can be activated by amide coupling reagents, subsequent nucleophilic attack by the indole nitrogen does not proceed.

Based on these preliminary investigations, we sought to establish a general synthetic route to *N*‑acylindole linkage **26** (Figure 3B). Since the intrinsic electronic properties of the indole nitrogen cannot be altered, we reasoned that instead of constructing the N─C bond between the indole and the carbonyl group at a late stage, it would be more effective to first prepare an *N*‑acylated aniline derivative **27** and subsequently employ a Pd(0)‑catalyzed regioselective Larock macrocyclization to assemble the *N*‑acylindole linkage **26** [55, 56, 57, 58, 59, 60, 61]. A key feature of this strategy is the use of an aniline derivative, which is more nucleophilic than the indole nitrogen, to readily construct the *N*‑acyl framework at an early stage of the synthesis. Furthermore, by applying this approach to diverse substrates, it was anticipated that a general synthetic method for cyclic peptides bearing the *N*‑acylindole linkage could be established.

The retrosynthetic analysis of bulbiferamide A (**3**) is shown in Figure 3C. A critical aspect in the synthesis of bulbiferamide A (**3**) is the construction of the labile *N*‑acylindole linkage under mild conditions. Following its formation, it is essential to mask the amine functionality at an early stage with a ureido cap to mitigate instability. Indeed, Parkinson and Agarwal have suggested that when a free amine is present in the substrate, *N*,*N*‑acyl transfer may occur, leading to cleavage of the *N*‑acylindole linkage [47]. Accordingly, after the construction of the unstable *N*‑acylindole linkage **29**, stabilization was planned by early conjugation with compound **28** containing a ureido moiety, thereby enabling the total synthesis of bulbiferamide A (**3**).

Our group has recently reported several syntheses of RiPPs employing Larock macrocyclization (cihunamide B [62], lapparbin [63], micitide 982 [64], neopetromin [65], and strecintide 839 [66]. Among these noncanonical cyclic peptides containing indole frameworks, many exhibit potent biological activities but can only be isolated in milligram quantities from natural sources.

Moreover, due to their highly strained cyclic structures, conventional cyclization methods such as amide coupling or Ullmann coupling fail to proceed. Thus, a general and versatile synthetic methodology was required. Extensive screening of cyclization conditions revealed that construction of biaryl motifs via Larock macrocyclization was highly effective for the supply of these RiPPs. Based on this background, it was anticipated that *N*‑acylindole linkage **29** could be constructed from cyclization precursor **31** via intermediate **30** using Pd(0)‑catalyzed Larock macrocyclization. Notably, this cyclization proceeds under relatively mild conditions employing weak bases, without the need for strongly acidic or basic environments. Cyclization precursor **31** was planned to be synthesized from readily available and inexpensive amino acid fragments, alkyne fragment (**32**), and 2‑bromoaniline (**33**).

**SCHEME 1:**
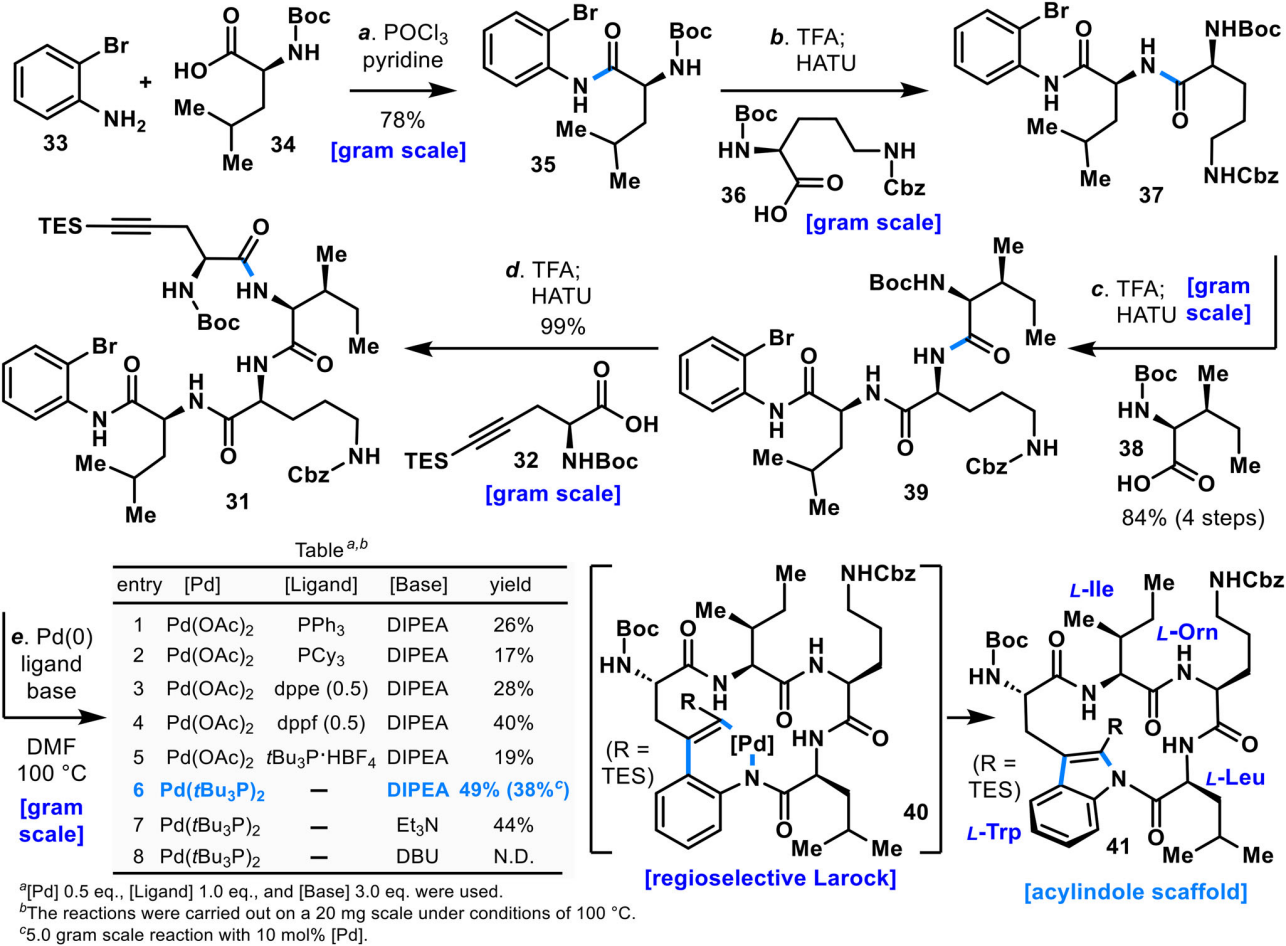
Construction of the *N*‑acylindole linkage (**41**)^*a*^.*^ a^* For detailed reagents and conditions, see the Supporting Information.

The total synthesis of bulbiferamide A (**3**) commenced with the amide coupling of 2‑bromoaniline (**33**) and Boc (*tert*‐butoxycarbonyl)‑leucine (**34**) (Scheme 1). Due to the low nucleophilicity of aniline derivative **33**, no coupling occurred under HATU conditions. Although EDCI/DMAP (4‐dimethylaminopyridine) promoted the reaction, the desired product **35** was obtained in less than 20% yield. After extensive investigation, the optimal method was identified as activation of Boc‑leucine (**34**) as its acid chloride using POCl_3_, followed by introduction of 2‑bromoaniline (**33**). The resulting coupling product **35** was subsequently converted to cyclization precursor **31** through sequential Boc deprotection with TFA (trifluoroacetic acid) and amide couplings mediated by HATU, introducing Boc‑ornithine (**36**), Boc‑isoleucine (**38**), and alkyne fragment (**32**).

Construction of the *N*‑acylindole linkage **41** was then examined (table in Scheme 1). In general, Larock macrocyclization benefits from bulky, electron‑rich ligands. Accordingly, Pd(OAc)_2_ (0.5 equiv) was employed with organic bases, and various ligands were screened. Initial trials with PPh_3_ and PCy_3_ (1 equiv each) afforded the desired *N*‑acylindole linkage **41**, but in low yields of 26% and 17%, respectively, with substantial amounts of unreacted precursor **31** remaining (entries 1–2). Use of dppe [1,2‐bis(diphenylphosphino)ethane] (0.5 equiv) gave 28% yield, accompanied by dehalogenated byproducts (entry 3). In contrast, dppf [1,1′‐bis(diphenylphosphino)ferrocene] (0.5 equiv) improved the yield to 40% (entry 4).

Further ligand screening revealed that the combination of *t*Bu_3_P·HBF_4_ with Pd(OAc)_2_ afforded only 19% yield (entry 5), whereas use of the Pd(*t*Bu_3_P)_2_ complex increased the yield to 49% (entry 6). The lower yield with *t*Bu_3_P·HBF_4_ is presumed to result from inhibition by proton sources present in the reaction medium. Remarkably, Larock macrocyclization proceeded on a 5 g scale with Pd(*t*Bu_3_P)_2_ reduced to 10 mol%, still affording **41** in 38% yield (entry 6). Whereas previously reported enzymatic methods employing *E. coli* cultures suffered from scalability issues, this approach enabled gram‑scale supply of the *N*‑acylindole linkage [47, 48, 50].

**SCHEME 2:**
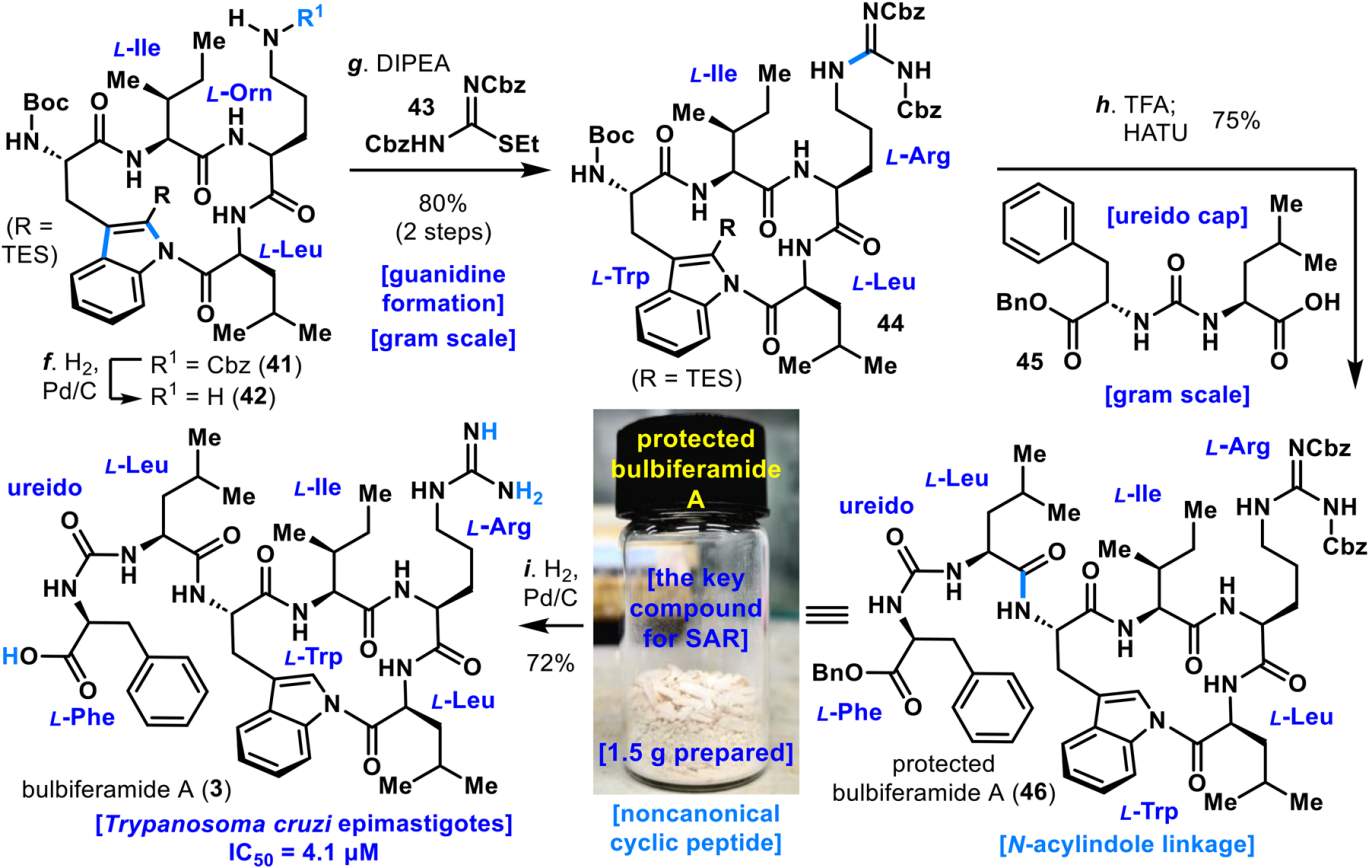
Total synthesis of bulbiferamide A (**3**)*^a^*. *^a^* For detailed reagents and conditions, see the Supporting Information.

Screening of bases revealed that Et_3_N afforded 44% yield, which was inferior to *N*,*N*‑diisopropylethylamine (DIPEA) (entry 7). Use of 1,8‐diazabicyclo[5.4.0]undecane‐7‐ene (DBU) resulted in substrate decomposition and no product formation (entry 8). Additional inorganic bases and solvents were also examined, but entry 6 provided the most favorable outcome (for further details, see Supporting Information). Importantly, the cyclization did not require dilute conditions, and no oligomerization or other side reactions were observed. The obtained cyclized product **41** was handled with care due to concerns about potential instability arising from N─C bond cleavage of the *N*‑acylindole linkage. Compound **41** was subjected to Cbz (benzyloxycarbonyl) deprotection with Pd/C and H_2_ to afford amine **42**, which was subsequently converted to compound **44** by introduction of a guanidine moiety (Scheme 2). Removal of the Boc group from **44** under acidic conditions was then investigated. Treatment with HCl in DCM (dichloromethane) (2.0 M) resulted only in substrate decomposition, likely due to cleavage of the *N*‑acylindole linkage. Screening of alternative conditions revealed that Boc removal proceeded smoothly without decomposition when TFA/DCM (1:9) was employed. Following TES (triethylsilyl) and Boc deprotection with TFA, compound **44** was subsequently coupled to the ureido‑containing fragment **45** using HATU, thereby furnishing the protected bulbiferamide A (**46**) in a quantity of 1.5 g. The ureido‑capped protected bulbiferamide A (**46**) was found to be a stable solid, storable at ambient temperature. Finally, removal of the benzyl ester and two Cbz groups from **46** under Pd/C, H_2_ in the presence of acetic acid furnished bulbiferamide A (**3**), thereby completing its total synthesis with the *N*‑acylindole linkage intact.

As described above, the synthesized bulbiferamide A (**3**) not only exhibits potent inhibitory activity against *T. cruzi* epimastigotes, the causative parasite of Chagas disease (IC_50_ = 4.1 µM), but also shows extremely low toxicity toward mammalian cells even at high concentrations (no cytotoxicity against P388 murine leukemia cells at 100 µM), as confirmed by Igarashi and coworkers in 2023 [28]. Thus, bulbiferamide A (**3**) possesses advantages not found in the existing Chagas disease drugs nifurtimox (**1**) and benznidazole (**2**), which were developed half a century ago and are associated with severe side effects [20, 21, 22, 23].

**FIGURE 4:**
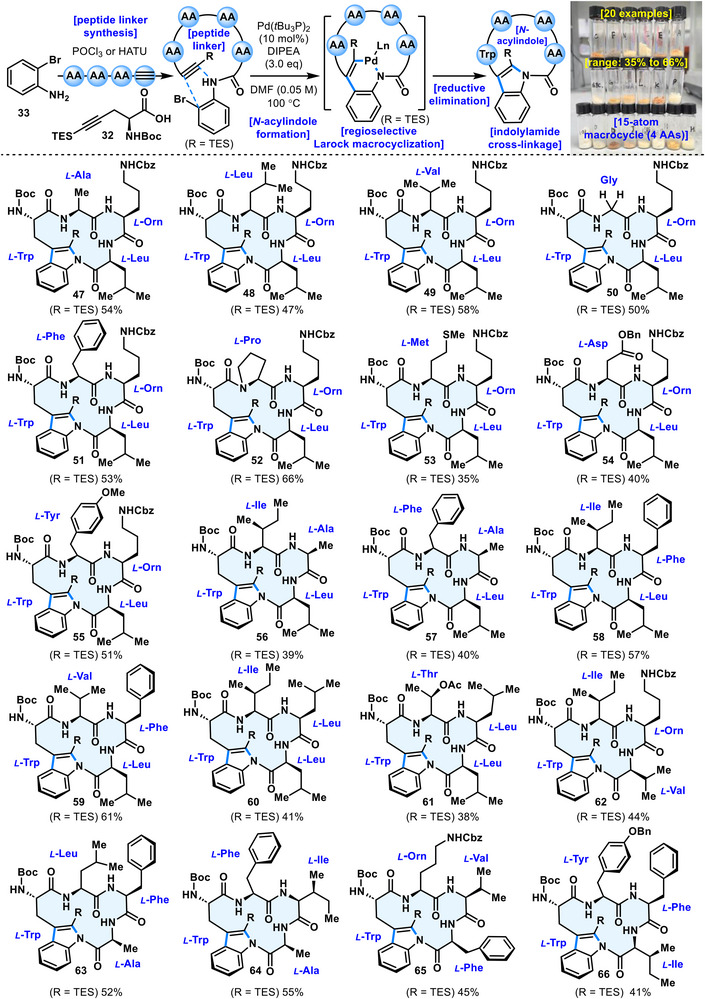
Substrate scope*^a^*. *^a^* For detailed reagents and conditions, see the Supporting Information.

Against this background, the generality of *N*‑acylindole linkage formation via the present cyclization reaction was investigated with a view toward the synthesis of bulbiferamide A (**3**) analogues (Figure 4). Application to various oligopeptide linkers was examined. First, we attempted to modify the third amino acid residue on the natural cyclic scaffold by replacing the natural *L*‐isoleucine with various other amino acid residues. Substrates ranging from the simplest glycine to *L‐*aspartic acid, *L‐*tyrosine, and even sulfur‐containing *L‐*methionine etc. (**47–55**) all afforded the corresponding cyclized products in comparable yields. Subsequently, further alteration of the second amino acid residue on the ring skeleton (**56–61**), or simultaneous introduction arbitrary amino acid residues on the cyclic framework (**62–66**), did not significantly affect the cyclization process. This demonstrated that Larock macrocyclization successfully afforded *N*‑acylindole linkages across diverse amino acid‑containing linkers, providing cyclized products on the several‑hundred‑milligram scale in yields ranging from 35% to 66%. Notably, substrates containing proline gave particularly favorable results, with cyclized product **52** obtained in 66% yield. This outcome is presumed to arise from conformational rigidity imparted by proline, which facilitates the cyclization process.

**FIGURE 5:**
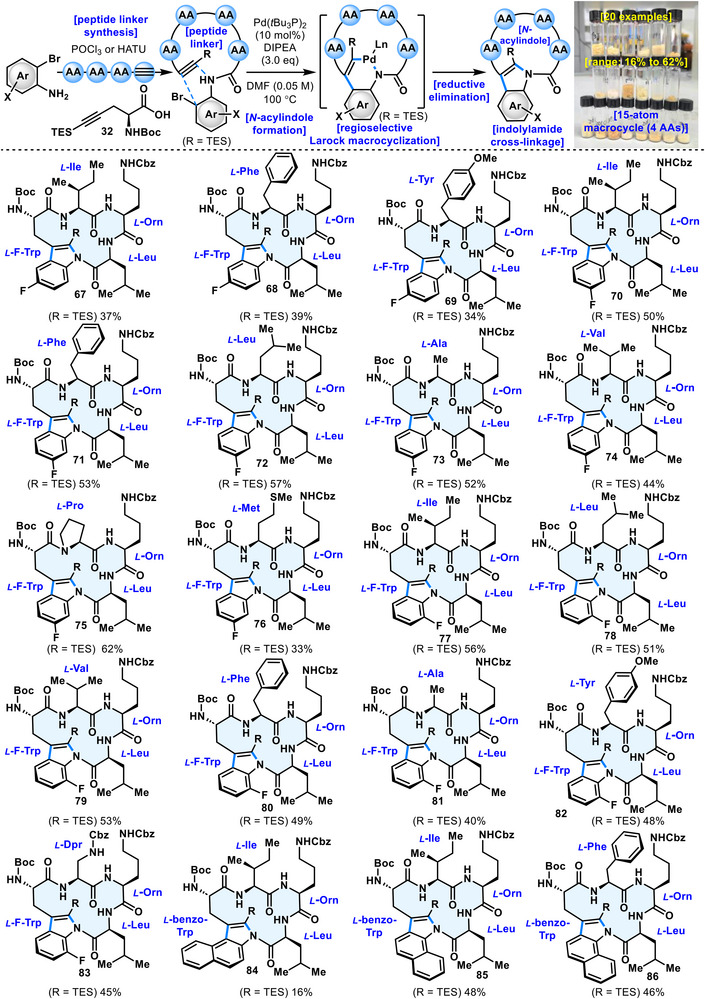
Substrate scope*^a^*. *^a^* For detailed reagents and conditions, see the Supporting Information.

Next, the generality of the aromatic moiety was assessed by constructing *N*‑acylindole linkages from various indole derivatives (Figure 5). The cyclization reaction proceeded smoothly for compounds substituted with fluorine at the C5 position of the indole (**67–69**). Similarly, substrates bearing fluorine substituents at the C6 and C7 positions (**70–83**) underwent successful *N*‑acylindole linkage formation. It is also noteworthy that cyclized products containing three consecutive aromatic rings (**84–86**) were accessible via this reaction. For cyclized product **84**, debromination was observed during the reaction, resulting in a yield of 16%.

**FIGURE 6:**
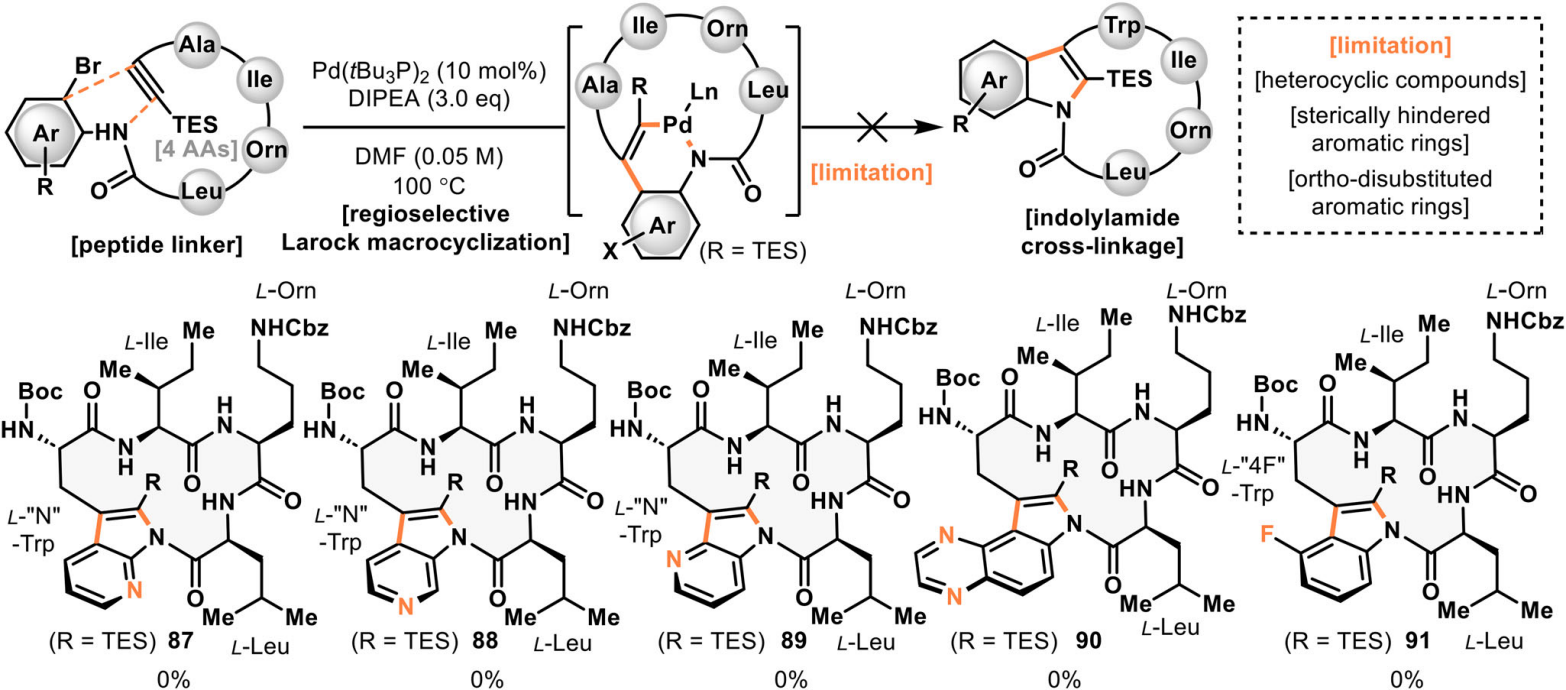
Substrate scope (failed attempts).

The limitations of this cyclization are summarized in Figure 6. To probe the scope, substrates incorporating various heterocyclic compounds were tested. In the case of aza‑indole derivatives (**87–89**), the cyclization did not proceed at all, and *N*‑acylindole linkages could not be constructed. In many of these substrates, debromination was observed, and no desired cyclized products were detected. Similarly, substrates containing pyrazine frameworks failed to yield the desired cyclized product **90**, with only debrominated byproducts obtained. Furthermore, the reaction was not applicable to indole derivatives substituted with fluorine at the C4 position (**91**), likely due to increased steric hindrance around the aromatic ring.

Taken together, these results demonstrate that favorable outcomes were achieved in cyclizations employing different amino acids and various non‑natural ortho‑bromoaniline linkers, highlighting the broad applicability of this Larock macrocyclization strategy for the synthesis of indole *N*‑acyl cyclic peptides (Figures 4, 5). However, the cyclized products obtained thus far were limited to 15‑membered rings (4 amino acids).

**FIGURE 7:**
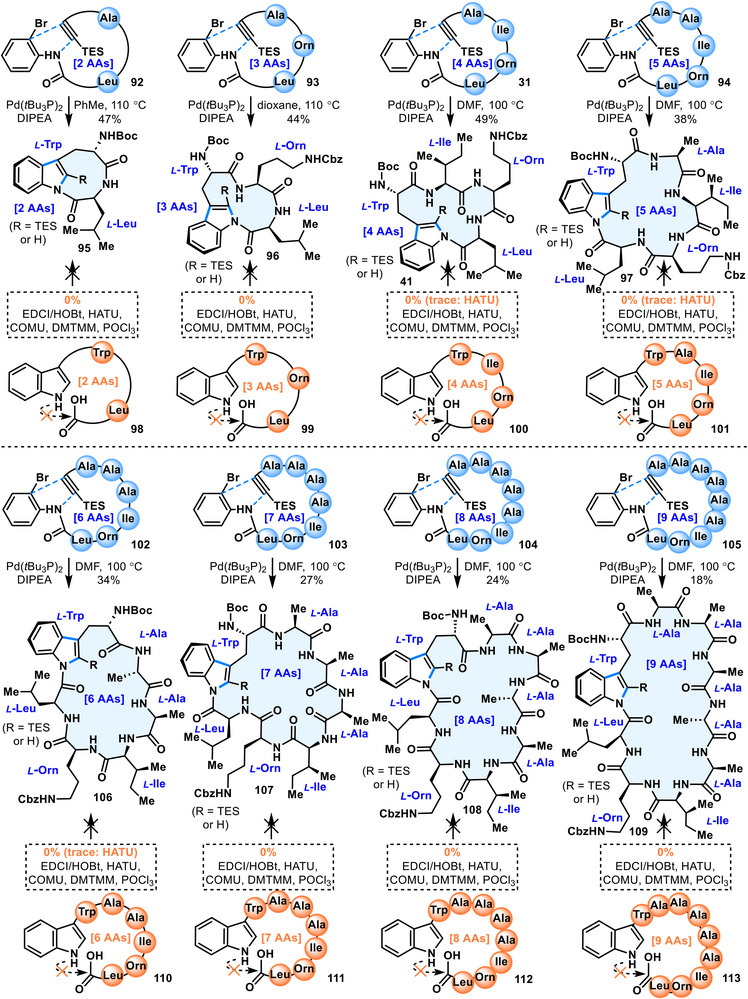
Substrate scope*^a^*. *^a^* For detailed reagents and conditions, see the Supporting Information.

Previous studies by Parkinson and Agarwal, Matsuda and Wakimoto, and Chekan and coworkers similarly reported only 15‑membered rings, with enzymatic cyclization failing for smaller 12‑membered rings (3 amino acids) and larger 18‑membered rings (5 amino acids) [47, 48, 50]. Even smaller 9‑membered rings (2 amino acids) and larger macrocycles such as 21‑membered (6 amino acids), 24‑membered (7 amino acids), and 27‑membered (8 amino acids) rings have not been attempted. To overcome this limitation, the present cyclization reaction was applied to the synthesis of *N*‑acylindole linkages with various ring sizes (Figure 7). First, substrate **37** was coupled with alkyne fragment **32** via simple amide condensation to afford ortho‑bromoaniline Larock precursor **93** containing three amino acid residues. Owing to its low residue count, precursor **93** was soluble in 1,4‐dioxane, and cyclization was performed in 1,4‐dioxane with 30 mol% Pd catalyst and 3 equiv DIPEA at 110 °C for 6 h.

Despite the significant ring strain, the desired 12‑membered *N*‑acylindole linkage compound **96** was obtained in 44% isolated yield. Reducing the amino acid residue count to two, precursor **92** was subjected to the same conditions using toluene as solvent, and unexpectedly, cyclization proceeded smoothly to afford the highly strained 9‑membered indole *N*‑acyl cyclic peptide **95** in 47% isolated yield. These results demonstrate the unique advantage of Larock macrocyclization in enabling the synthesis of highly strained small‑ring *N*‑acylindole linkages, which cannot be achieved by enzymatic catalysis or biosynthesis. For comparison, linear peptides **98–100** were synthesized and subjected to macrocyclization via amide coupling. Conventional amide coupling reagents [EDCI/HOBt (1‐hydroxybenzotriazole), HATU, COMU, DMT‑MM] as well as stronger acylating agents (POCl_3_) were tested, but none afforded the desired cyclized products. Only trace amounts (<5%) of cyclized products were observed with HATU in some cases, insufficient for isolation. This outcome is attributed to the extremely weak nucleophilicity of the indole nitrogen, which stalls the reaction at the activated ester stage, compounded by the high ring strain.

Inspired by the work of Agarwal and coworkers, larger ring systems (≥18‑membered, 5 amino acids) were also investigated [47]. Substrate **39** was subjected to sequential Boc deprotection, Boc‑*L*‑alanine coupling, further Boc deprotection, and introduction of alkyne acid **32** to yield the 5‑residue Larock precursor **94**. Due to solubility issues, DMF was employed as solvent, and under standard cyclization conditions, Pd‑catalyzed indole *N*‑acyl macrocyclization proceeded smoothly to afford cyclized product **97**. Although precursor **94** exhibited poor solubility, the cyclized product showed markedly improved solubility, and the desired 18‑membered compound **97** was obtained in 38% isolated yield. In contrast, Agarwal's enzymatic method failed to detect any product [47]. The successful synthesis of the 18‑membered ring suggested the feasibility of expanding to larger amino acid counts. Accordingly, precursors **102** (6 residues), **103** (7 residues), **104** (8 residues), and **105** (9 residues) were synthesized. Under standard conditions, these precursors afforded the corresponding macrocyclic products **106–109** whereas conventional amide coupling reagents and POCl_3_ also proved ineffective. These findings underscore the unique advantage of this strategy over enzymatic methods in enabling the synthesis of larger, non‑natural indole *N*‑acyl macrocyclic peptides.

## 3 Conclusion

Chagas disease, despite being discovered more than a century ago, remains a tropical illness for which no effective vaccine is available. Moreover, although Chagas disease therapeutics are designated on the WHO's list of essential drugs, the agents currently in use were commercialized over 50 years ago and continue to be employed today, raising concerns regarding the emergence of resistant strains and severe side effects. In this study, we developed a new, scalable, and general synthetic method for constructing the *N*‑acylindole linkage, which had previously lacked a chemical synthesis route. This approach enabled gram‑scale supply of bulbiferamide A (**3**), a compound exhibiting potent inhibitory activity against *T. cruzi* epimastigotes (IC_50_ = 4.1 µM) with favorable selectivity toward mammalian cells. Furthermore, cyclizations involving diverse ring sizes, different linkers, and various indole derivatives—previously inaccessible by enzymatic methods—were successfully achieved using this strategy, thereby demonstrating the ease of analogue synthesis. These findings establish a foundation for the future synthesis of analogues of noncanonical cyclic peptides and for the broader supply of *N*‑acylindole linkages.

## Conflicts of Interest

The authors declare no conflicts of interest.

## Supporting information




**Supporting File 1**: anie71641‐sup‐0001‐SuppMat.pdf.

## Data Availability

The data that support the findings of this study are available in the supplementary material of this article.
