# Supplementary material for: Scalable Access to N‑Acylindole Linkages: Enabling the Synthesis of Antitrypanosomal Noncanonical Cyclic Peptides for Chagas Disease
Source: Angew Chem Int Ed Engl. 2026 Feb 27;65(15):e26149. doi: 10.1002/anie.202526149 (PMC13053918; doi:10.1002/anie.202526149)
Supplement: Supplementary file 1 — Supporting File 1: anie71641‐sup‐0001‐SuppMat.pdf. [file ANIE-65-e26149-s001.pdf]

SUPPORTING INFORMATION

Scalable Access to *N*-Acylindole Linkages: Enabling the Synthesis of Antitrypanosomal Noncanonical Cyclic Peptides for Chagas Disease

Jie Zhang, Hugh Nakamura *

The Hong Kong University of Science and Technology (HKUST)
Clear Water Bay, 999077, Hong Kong SAR, China

Content

General Experimental 3

Abbreviations 3

Synthetic route of Bulbiferamide A (3) 5

Details of the condition screening of Larock macrocyclization 6

Initial attempt of amide coupling cyclization 7

Total synthesis of Bulbiferamide A (3) 8

^{1}H NMR Comparison of Bulbiferamide A (3) 15

^{13}C NMR Comparison of Bulbiferamide A (3) 17

LCMS of Bulbiferamide A (3) after HPLC purification (negative scan) 19

HRMS of Bulbiferamide A (3) 20

Synthesis of compounds 43 and 45 21

Synthesis of cyclization compounds 47–86; 95–97 and 106–109 23

Synthesis of cyclization precursors 61

- **Synthesis of precursors 92–94 and 102–105** 61
- **General synthetic procedure for Larock precursors S-4–43** 69

Synthesis of acylated aromatic amine derivatives 97

Copies of NMR 103

General Experimental

Reagents were purchased from commercial sources (Bide, Energy, TCI, and Sigma-Aldrich) and used without further purification unless otherwise stated. Yields refer to chromatographically unless otherwise stated. Reactions were monitored by LC/MS and thin layer chromatography (TLC). TLC was performed using 0.2-0.25 mm silica plates, and using short-wave UV light as the visualizing agent, phosphomolybdic acid with $Ce(SO_4)_2$, or $KMnO_4$ via heat as developing agents. NMR spectra were recorded on Bruker AVII 400 and JEOL 600 instruments and are calibrated using residual undeuterated solvent ($CHCl_3$ at 7.26 ppm ^{1}H NMR, 77.16 ppm ^{13}C NMR; DMSO-d_6 at 2.50 ppm ^{1}H NMR, 39.52 ppm ^{13}C NMR; CD_3OD at 3.31 ppm ^{1}H NMR, 49.00 ppm ^{13}C NMR). The following abbreviations were used to explain multiplicities: s = singlet, d = doublet, t = triplet, q = quartet, m = multiplet, br = broad. Column chromatography was performed using 230-400 mesh silica gel, and PTLC was performed using 0.2-0.25 mm silica plates. High-resolution mass spectra (HRMS) were recorded on an Agilent LC/MSD TOF mass spectrometer by electrospray ionization time of flight reflectron experiments.

Abbreviations

Ac	Acetyl
aq.	Aqueous
Boc	*tert*-Butoxycarbonyl
Bu	Butyl
Cbz	Benzyloxycarbonyl
CDI	Carbonyldiimidazole
COMU	1-[(1-(Cyano-2-ethoxy-2-oxoethylideneaminooxy)-dimethylamino-morpholino)] uronium hexafluorophosphate
DBU	1,8-Diazabicyclo[5.4.0]undecane-7-ene
DCC	*N,N'*-Dicyclohexylcarbodiimide
DCM	Dichloromethane
DIC	*N,N'*-Diisopropylcarbodiimide
DIPEA	*N*-Diisopropylethylamine
DMAc	*N,N*-Dimethylacetamide
DMAP	4-Dimethylaminopyridine
DMF	*N,N*-Dimethylformamide

DMSO	Dimethyl sulfoxide
DMT-MM	4-(4,6-Dimethoxy-1,3,5-triazin-2-yl)-4-methyl morpholinium chloride
dppe	1,2-Bis(diphenylphosphino)ethane
dppf	1,1'-Bis(diphenylphosphino)ferrocene
EDCI	1-(3-Dimethylaminopropyl)-3-ethylcarbodiimide hydrochloride
Et	Ethyl
HATU	Hexafluorophosphate Azabenzotriazole Tetramethyl Uronium
HOAt	1-Hydroxy-7-azabenzotriazole
HOBt	1-Hydroxybenzotriazole
HPLC	High Performance Liquid Chromatography
HRMS	High Resolution Mass Spectrometry
LCMS	Liquid Chromatograph Mass Spectrometer
Me	Methyl
NMP	*N*-Methyl-2-pyrrolidone
NMR	Nuclear Magnetic Resonance Spectroscopy
PCy_3	Tricyclohexyl phosphine
Ph	Phenyl
PPh_3	Triphenylphosphine
TES	Triethylsilyl
TFA	Trifluoroacetic acid
THF	Tetrahydrofuran
TLC	Thin Layer Chromatography

Synthetic route of Bulbiferamide A (3)

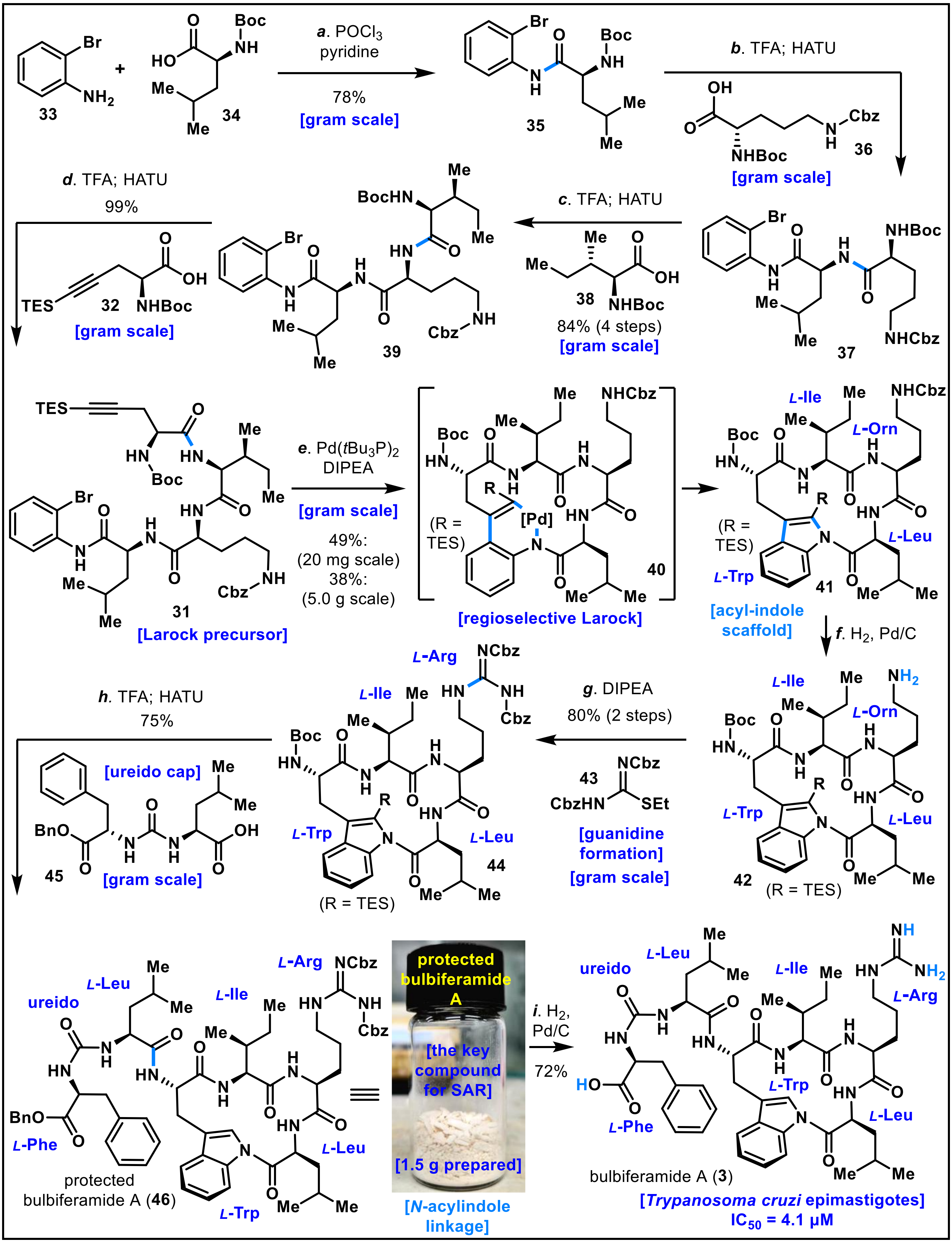

Details of the condition screening of Larock macrocyclization

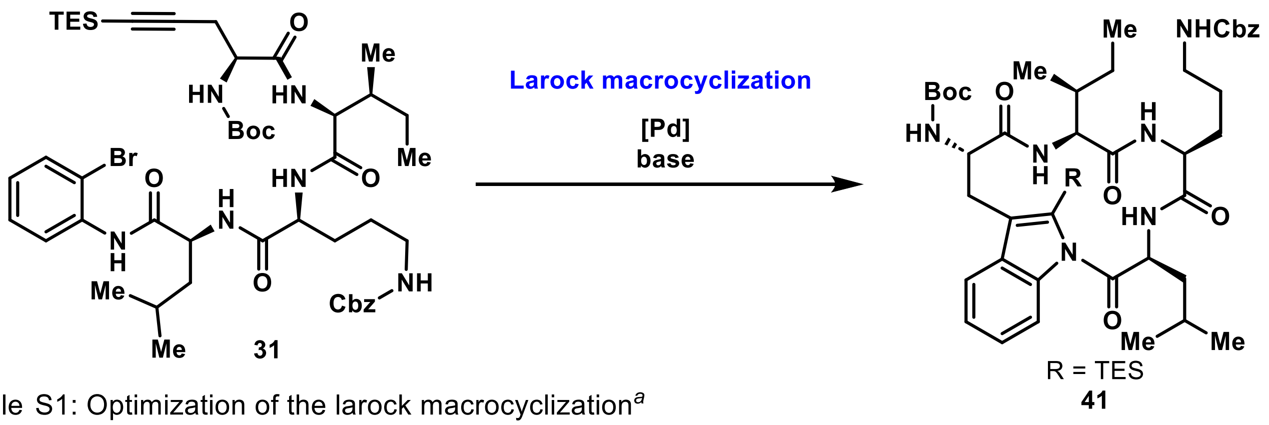

Table S1: Optimization of the larock macrocyclization[a]

entry	[Pd] 0.5 eq.	[Ligand] 1.0 eq.	[Base] 3.0 eq.	[Solvents] 0.05M	yield[b]
1	$Pd(OAc)_2$	PPh_3	DIPEA	DMF	26%
2	$Pd(OAc)_2$	PCy_3	DIPEA	DMF	17%
3	$Pd(OAc)_2$	dppe (0.5)	DIPEA	DMF	28%
4	$Pd(OAc)_2$	dppf (0.5)	DIPEA	DMF	40%
5	$Pd(OAc)_2$	$t\text{Bu}_3\text{P}\bullet\text{HBF}_4$	DIPEA	DMF	19%
6	$Pd(t\text{Bu}_3\text{P})_2$	—	DIPEA	DMF	49% (38%[c])
7	$Pd(t\text{Bu}_3\text{P})_2$	—	Et_3N	DMF	44%
8	$Pd(t\text{Bu}_3\text{P})_2$	—	DBU	DMF	N.D.
9	$Pd(t\text{Bu}_3\text{P})_2$	—	K_2CO_3	DMF	N.D.
10	$Pd(t\text{Bu}_3\text{P})_2$	—	Cs_2CO_3	DMF	N.D.
11	$Pd(t\text{Bu}_3\text{P})_2$	—	NaOAc	DMF	<10%
12	$Pd(t\text{Bu}_3\text{P})_2$	—	DIPEA	DMAc	45%
13	$Pd(t\text{Bu}_3\text{P})_2$	—	DIPEA	NMP	42%
14	$Pd(t\text{Bu}_3\text{P})_2$	—	DIPEA	Dioxane	37%
15	$Pd(t\text{Bu}_3\text{P})_2$	—	DIPEA	Toluene	35%

[a]The reactions were carried out on a 20 mg scale under conditions of 100 °C for 6 h. [b] Isolated yields.
[c]5.0 gram scale reaction with 10 mol% [Pd].

Initial attempt of amide coupling cyclization

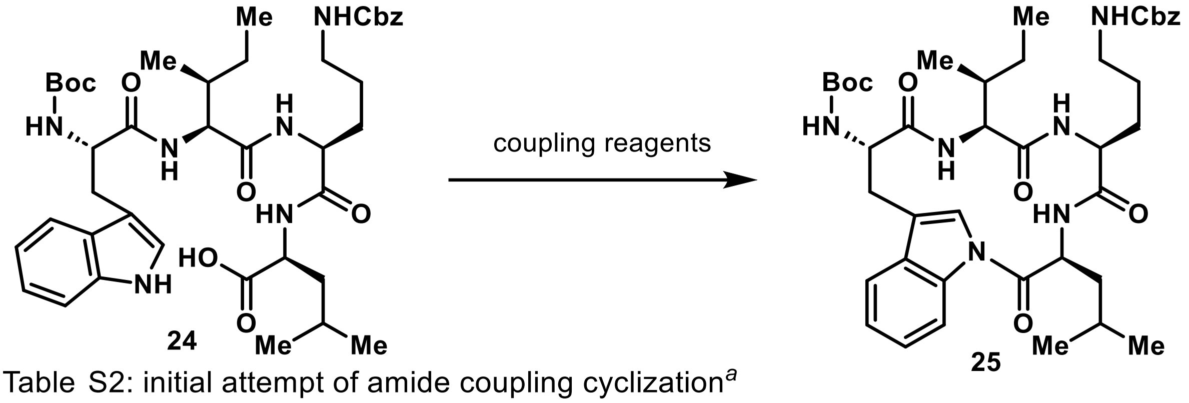

Table S2: initial attempt of amide coupling cyclization[a]

entry	amide coupling conditions[b]	yield[c]
1	DIC/HOBT	N.D.[e]
2	DCC/HOBT	N.D.
3	EDCI/HOBT	N.D.
4	EDCI/HOAT	trace
5	EDCI/DMAP	N.D.
6	HATU	< 5%
7	COMU	N.D.
8	DMT-MM	N.D.
9	CDI	trace
10	$POCl_3$[d]	trace

[a]The reactions were carried out on a 30 mg scale in DMF (0.01 M) at rt for 6 h. [b]2.0 eq. of coupling reagents and additives were used. [c]Isolated yields. [d]Pyridine was used as solvent. [e]N. D. = not detected.

Total synthesis of Bulbiferamide A (3)

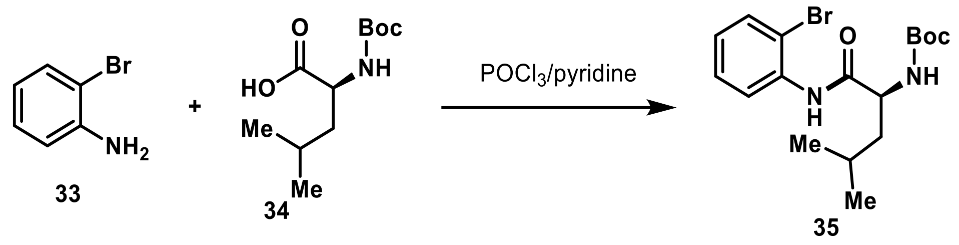

To a solution of 2-bromoaniline **33** (1.72 g, 10.0 mmol, 1.0 equiv.) and Boc-*L*-leucine **34** (2.31 g, 10.0 mmol, 1.0 equiv.) in 30 mL pyridine was added $POCl_3$ (1.0 mL, 11.0 mmol, 1.1 equiv.) dropwise over 30 min at −10 °C. Then the reaction mixture was allowed to stir at −10 °C for another 30 min. Once the starting material was consumed completely, the mixture was quenched by saturated *aq.* $NaHCO_3$ (50 mL) and extracted with DCM (3 X 50 mL). The organic layers were combined, washed by saturated *aq.* NaCl, dried over Na_2SO_4 and removed under reduced pressure to give the residue; the residue was purified by silica gel chromatography (5% EtOAc/hexane) to give the compound **35** (3.01 g, 78% yield).

Compound 35

Physical State: colorless oil

^{1}H NMR (600 MHz, $CDCl_3$): δ 8.51 (s, 1H), 8.33 (d, J = 8.6 Hz, 1H), 7.50 (dd, J = 8.0, 1.5 Hz, 1H), 7.28 (s, 1H), 7.01 – 6.87 (m, 1H), 5.14 – 4.86 (m, 1H), 4.29 (s, 1H), 1.81 (dt, J = 14.0, 6.5 Hz, 1H), 1.78 – 1.70 (m, 1H), 1.55 (ddd, J = 13.7, 9.7, 5.5 Hz, 1H), 1.44 (s, 9H), 0.96 (dd, J = 9.3, 6.5 Hz, 6H).

^{13}C NMR (151 MHz, $CDCl_3$): δ 171.05, 155.78, 135.62, 132.37, 128.42, 125.38, 121.92, 113.69, 80.65, 54.25, 40.96, 28.42, 24.98, 23.07, 21.95.

HRMS (ESI-TOF): calculated for $C_{17}H_{25}BrN_2NaO_3^+$ $[M+Na]^+$: 407.0941, found: 407.0945.

TLC: R_f = 0.6 (15:1 Hexane:EtOAc, $Ce_2(SO_4)_3$ in phosphomolybdic acid).

$[\alpha]^{25}_D$: −11.6 (c = 1.0, $CHCl_3$)

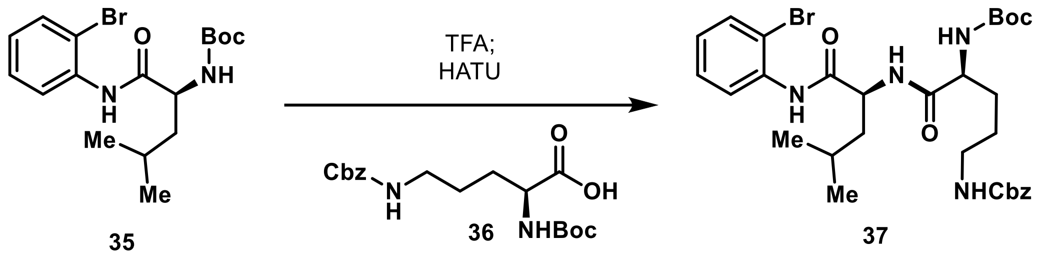

The compound **35** (3.0 g, 7.8 mmol, 1.0 equiv.) was dissolved in 30 mL DCM and TFA (10 mL) was added dropwise. The reaction mixture was stirred at rt for 1 h. Then 40 mL toluene was added, and the solution was concentrated *in vacuo* (three times with toluene) to give the residue. The residue was used in the next step directly without further purification.

To a solution of the crude, compound **36** (3.42 g, 9.36 mmol, 1.2 equiv.) and DIPEA (5.4 mL, 31.2 mmol, 4.0 equiv.) in 40 mL anhydrous DMF was added HATU (3.56 g, 9.36 mmol, 1.2 equiv.) in portion wise. The reaction mixture was stirred at rt for 1 h, then quenched by 0.5 M HCl (100 mL) and extracted with EtOAc (50 mL) three times. The organic layers were combined and washed with saturated *aq.* NaCl, dried over Na_2SO_4 and removed under reduced pressure to give the residue **37**; the residue was used in the next step directly without further purification.

Compound 37

Physical State: white solid

^{1}H NMR (600 MHz, CDCl$_3$): δ 8.35 – 8.21 (m, 2H), 7.50 (d, J = 8.1 Hz, 1H), 7.32 (d, J = 3.9 Hz, 4H), 7.28 (dd, J = 11.6, 6.4 Hz, 2H), 7.16 (d, J = 7.5 Hz, 1H), 6.96 (t, J = 7.7 Hz, 1H), 5.19 (d, J = 8.3 Hz, 1H), 5.10 (d, J = 11.9 Hz, 1H), 5.03 (d, J = 12.3 Hz, 1H), 4.95 (d, J = 6.5 Hz, 1H), 4.56 (q, J = 7.8, 7.2 Hz, 1H), 4.36 (s, 1H), 3.44 (s, 1H), 3.12 (dd, J = 14.3, 5.8 Hz, 1H), 1.93 – 1.72 (m, 3H), 1.66 (s, 1H), 1.63 – 1.49 (m, 3H), 1.42 (s, 9H), 0.95 (dd, J = 21.6, 6.3 Hz, 6H).

^{13}C NMR (151 MHz, CDCl$_3$): δ 172.97, 170.27, 157.28, 156.06, 136.43, 135.62, 132.44, 128.62, 128.22, 125.53, 122.29, 113.85, 110.63, 80.25, 66.99, 52.89, 40.46, 39.60, 30.06, 28.38, 26.52, 24.80, 23.11, 21.92.

HRMS (ESI-TOF): calculated for $C_{30}H_{41}BrN_4NaO_6^+$ [M+Na]$^+$: 655.2102, found: 655.2109.

TLC: R_f = 0.5 (5:1 Hexane:EtOAc, $Ce_2(SO_4)_3$ in phosphomolybdic acid).

$[\alpha]^{25}_D$: –12.2 (c = 1.0, $CHCl_3$)

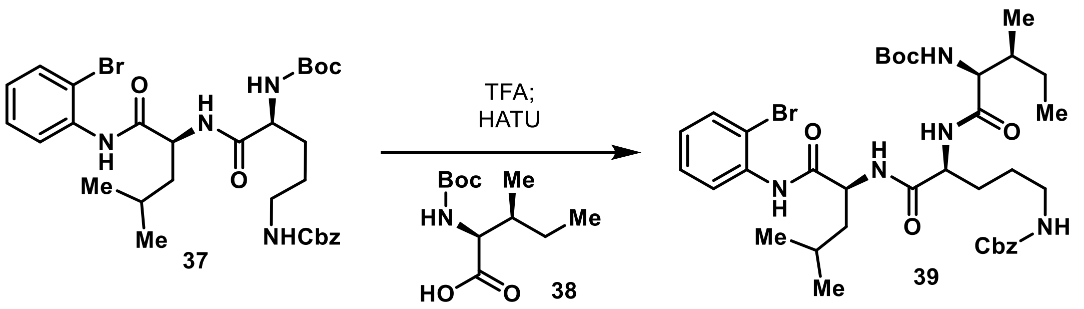

The crude **37** was dissolved in DCM (30 mL), TFA (10 mL) was added dropwise, the reaction was stirred at rt for 1 h. Then 40 mL toluene was added, and the solution was concentrated *in vacuo* (three times with toluene) to give the residue. The residue was used in the next step directly without further purification.

To a solution of the crude, compound **38** (2.16 g, 9.36 mmol, 1.2 equiv.) and DIPEA (5.4 mL, 31.2 mmol, 4.0 equiv.) in 40 mL anhydrous DMF was added HATU (3.56 g, 9.36 mmol, 1.2 equiv.) in portion wise. The reaction mixture was stirred at rt for 1 h then quenched by 0.5 M HCl (100 mL) and extracted with EtOAc (50 mL) three times. The organic layers were combined and washed with saturated *aq.* NaCl, dried over Na_2SO_4 and removed under reduced pressure to give the residue; the residue was purified by silica gel chromatography (5% Acetone/DCM) to give the compound **39** (5.02 g, 84% yield over 4 steps).

Compound 39

Physical State: white solid

^{1}H NMR (600 MHz, CDCl$_3$): δ 8.57 (s, 1H), 7.94 (d, J = 8.1 Hz, 1H), 7.51 (d, J = 8.3 Hz, 1H), 7.44 (d, J = 8.1 Hz, 1H), 7.29 (d, J = 4.3 Hz, 5H), 7.17 (t, J = 7.9 Hz, 1H), 6.91 (t, J = 7.7 Hz, 1H), 5.34 (d, J = 8.8 Hz, 1H), 5.19 (d, J = 6.4 Hz, 1H), 5.04 (t, J = 9.4 Hz, 2H), 4.87 – 4.75 (m, 1H), 4.73 – 4.60 (m, 1H), 4.22 (t, J = 8.0 Hz, 1H), 3.46 – 3.26 (m, 1H), 3.08 – 2.94 (m, 1H), 1.95 (s, 1H), 1.86 (q, J = 6.0 Hz, 1H), 1.77 (ddq, J = 20.0, 13.4, 6.8 Hz, 3H), 1.64 (ddd, J = 28.0, 13.7, 6.0 Hz, 2H), 1.48 (dq, J = 21.1, 8.1, 7.4 Hz, 3H), 1.36 (s, 9H), 1.07 (dt, J = 15.0, 7.8 Hz, 1H), 0.95 (d, J = 6.3 Hz, 3H), 0.93 – 0.83 (m, 9H).

^{13}C NMR (151 MHz, CDCl$_3$): δ 172.34, 172.30, 170.80, 157.35, 156.15, 136.61, 135.61, 132.53, 128.55, 128.22, 128.13, 126.05, 124.03, 79.91, 66.86, 59.19, 52.74, 51.62, 40.72, 39.63, 37.81, 30.19, 28.39, 26.40, 24.92, 24.77, 23.05, 22.08, 15.69, 11.50.

HRMS (ESI-TOF): calculated for $C_{36}H_{52}BrN_5NaO_7^+$ [M+Na]$^+$: 768.2942, found: 768.2944.

TLC: R_f = 0.4 (20:1 DCM:Acetone, $Ce_2(SO_4)_3$ in phosphomolybdic acid).

$[\alpha]^{25}_D$: –24.4 (c = 1.0, $CHCl_3$)

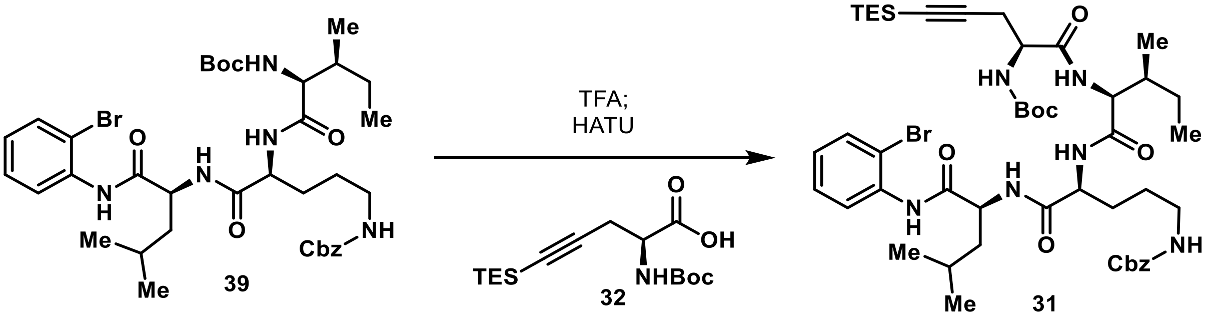

Compound **39** (5.0 g, 6.71 mmol, 1.0 equiv.) was dissolved in 25 mL DCM and TFA (8 mL) was added dropwise. The reaction mixture was stirred at rt for 1 h. Then 30 mL toluene was added and evaporated *in vacuo* (three times with toluene) to give the residue. The residue was used in the next step directly without further purification.

The crude and compound **32** (2.63 g, 8.05 mmol, 1.2 equiv.) were dissolved in DMF (30 mL). DIPEA (4.7 mL, 26.8 mmol, 4.0 equiv.) and HATU (3.06 g, 8.05 mmol, 1.2 equiv.) were added sequentially. The mixture was stirred at rt for 1 h then quenched by 0.5 M HCl (80 mL) and extracted with EtOAc (40 mL) three times. The combined organic layers were washed with saturated aqueous NaCl, dried over Na_2SO_4, and concentrated *in vacuo*. The resulting residue was purified by recrystallization (Hexane/DCM) to give the compound **31** as a white solid (6.3 g, 99% yield).

Compound 31

Physical State: white solid

^{1}H NMR (600 MHz, DMSO-*d*$_6$): δ 9.41 (s, 1H), 8.11 (d, *J* = 8.0 Hz, 1H), 8.03 (d, *J* = 8.0 Hz, 1H), 7.64 – 7.54 (m, 3H), 7.36 – 7.23 (m, 6H), 7.20 (t, *J* = 5.8 Hz, 1H), 7.14 – 7.05 (m, 2H), 4.95 (s, 2H), 4.48 (q, *J* = 7.7 Hz, 1H), 4.27 (td, *J* = 8.3, 5.4 Hz, 1H), 4.17 (dd, *J* = 8.8, 6.5 Hz, 1H), 4.12 (td, *J* = 9.5, 4.2 Hz, 1H), 2.93 (q, *J* = 6.5 Hz, 2H), 2.56 (dd, *J* = 17.0, 4.1 Hz, 1H), 2.43 (dd, *J* = 17.1, 10.2 Hz, 1H), 1.65 (ddt, *J* = 16.0, 12.8, 6.4 Hz, 3H), 1.55 (t, *J* = 7.3 Hz, 2H), 1.52 – 1.36 (m, 4H), 1.35 (s, 9H), 1.30 – 1.18 (m, 1H), 1.04 – 0.97 (m, 1H), 0.89 (t, *J* = 7.9 Hz, 12H), 0.83 (d, *J* = 6.5 Hz, 3H), 0.75 (q, *J* = 7.0 Hz, 6H), 0.47 (q, *J* = 7.9 Hz, 6H).

^{13}C NMR (151 MHz, DMSO-*d*$_6$): δ 172.06, 171.52, 171.04, 170.63, 156.65, 155.74, 137.74, 136.43, 133.21, 128.88, 128.59, 128.29, 127.07, 118.10, 106.10, 83.02, 78.77, 65.69, 57.14, 54.00, 52.59, 52.04, 41.17, 37.74, 29.80, 28.66, 28.59, 26.55, 24.61, 23.65, 22.03, 15.69, 11.71, 7.88, 4.48.

HRMS (ESI-TOF): calculated for $C_{47}H_{71}BrN_6NaO_8Si^+$ $[M+Na]^+$: 977.4178, found: 977.4177.

TLC: R$_f$ = 0.4 (15:1 DCM:Acetone, $Ce_2(SO_4)_3$ in phosphomolybdic acid).

$[\alpha]^{25}_{D}$: –31.7 (*c* = 1.0, $CHCl_3$)

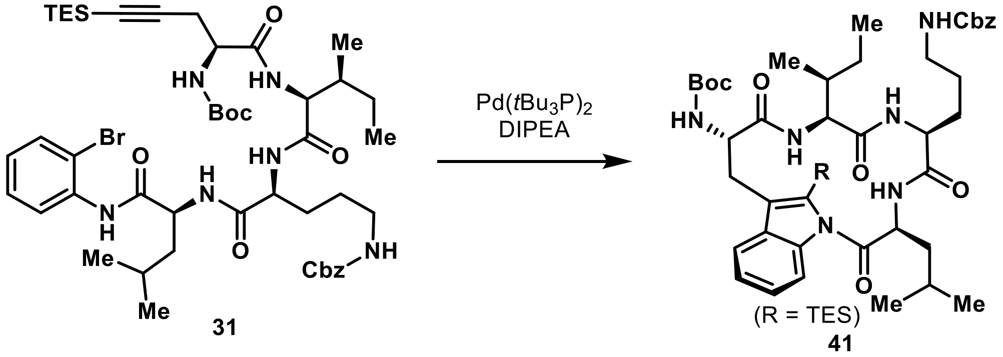

To a solution of compound **31** (5.0 g, 5.24 mmol, 1.0 equiv.) in 100 mL anhydrous DMF was added DIPEA (2.7 mL, 15.7 mmol, 3.0 equiv.) and Pd(*t*Bu$_3$P)$_2$ (271 mg, 0.53 mmol, 0.1 equiv.). The reaction mixture was

stirred at 100 °C under nitrogen atmosphere for 6 h. After the consumption of the starting material, the mixture was diluted with 200 mL EtOAc, washed with 0.5 M HCl and saturated *aq.* NaCl. The organic layer was dried over Na_2SO_4 and removed under reduced pressure to give the residue; the residue was purified by silica gel chromatography (35% EtOAc/hexane) to give the compound **41** (1.8 g, 38% yield).

Compound 41
Physical State: amorphous solid
^{1}H NMR (600 MHz, $CDCl_3$): δ 7.72 (d, *J* = 8.4 Hz, 1H), 7.66 (d, *J* = 7.8 Hz, 1H), 7.41 (d, *J* = 7.6 Hz, 2H), 7.38 – 7.34 (m, 3H), 7.33 – 7.29 (m, 1H), 7.17 (ddd, *J* = 8.2, 7.0, 1.2 Hz, 1H), 7.10 (t, *J* = 7.4 Hz, 1H), 6.28 (d, *J* = 7.4 Hz, 1H), 5.97 (d, *J* = 8.9 Hz, 1H), 5.31 – 5.14 (m, 3H), 4.90 (ddd, *J* = 12.4, 8.9, 3.6 Hz, 1H), 4.78 (s, 1H), 4.38 (dt, *J* = 24.1, 6.6 Hz, 2H), 4.19 (ddd, *J* = 11.3, 7.4, 4.2 Hz, 1H), 3.39 (t, *J* = 12.6 Hz, 1H), 3.13 (dd, *J* = 13.0, 3.6 Hz, 1H), 2.67 – 2.57 (m, 1H), 2.53 (ddd, *J* = 14.7, 10.7, 4.4 Hz, 1H), 2.29 – 2.18 (m, 1H), 2.01 (d, *J* = 14.7 Hz, 2H), 1.88 (ddt, *J* = 13.2, 10.0, 4.8 Hz, 1H), 1.76 (ddd, *J* = 14.2, 9.8, 4.2 Hz, 1H), 1.60 (ddt, *J* = 10.5, 6.7, 4.2 Hz, 2H), 1.45 (s, 9H), 1.38 – 1.31 (m, 1H), 1.30 – 1.18 (m, 2H), 1.05 – 1.00 (m, 3H), 0.97 (d, *J* = 6.7 Hz, 3H), 0.95 – 0.92 (m, 12H), 0.91 – 0.86 (m, 3H), 0.84 (d, *J* = 6.9 Hz, 3H), 0.77 (t, *J* = 7.4 Hz, 3H).
^{13}C NMR (151 MHz, $CDCl_3$): δ 174.54, 171.84, 171.46, 171.06, 156.79, 155.61, 140.04, 136.67, 129.36, 128.59, 128.19, 128.09, 124.88, 122.03, 118.68, 114.07, 80.57, 67.10, 58.61, 58.00, 53.99, 50.48, 40.32, 36.37, 36.29, 29.98, 28.39, 24.56, 24.48, 24.26, 23.59, 21.97, 15.57, 11.52, 7.82, 5.46.
HRMS (ESI-TOF): calculated for $C_{47}H_{70}N_6NaO_8Si^+$ $[M+Na]^+$: 897.4919, found: 897.4938.
TLC: R_f = 0.5 (2:1 Hexane:EtOAc, $Ce_2(SO_4)_3$ in phosphomolybdic acid).
$[\alpha]^{25}_D$: –57.4 (*c* = 1.0, $CHCl_3$)

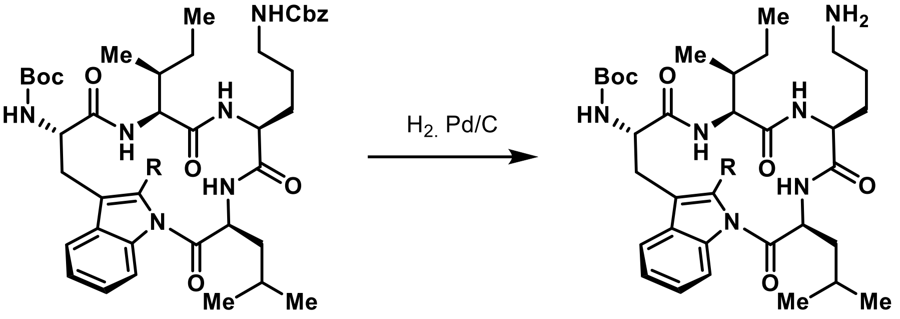

R = TES
41
R = TES
42

Compound **41** (1.8 g, 2.0 mmol) was dissolved in 20 mL MeOH and Pd/C (200 mg) was added. The reaction mixture was allowed to stir at rt under hydrogen atmosphere for 12 h. When complete, the solid was filtered and the solvent was removed under reduced pressure to give the residue **42**; the residue was used in the next step directly without further purification.

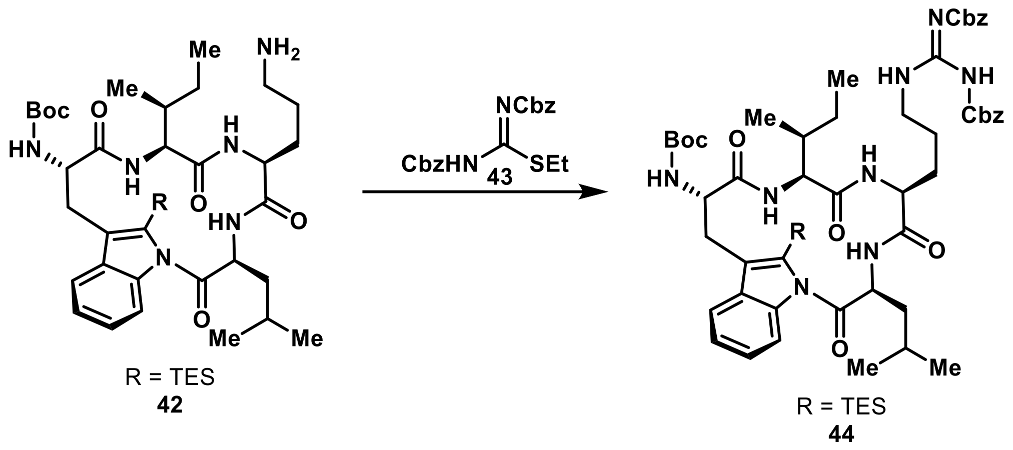

Crude product **42** and compound **43** (2.2 g, 6 mmol, 3.0 equiv.) were dissolved in 10 mL anhydrous DMF, and DIPEA (1.04 mL, 6 mmol, 3.0 equiv.) were added. Then the reaction was stirred at rt for 24 h. When complete, the reaction mixture was diluted with 50 mL EtOAc, washed with 0.5 M HCl and saturated *aq.* NaCl. The organic layer was dried over Na_2SO_4 and removed under reduced pressure to give the residue; the residue was purified by silica gel chromatography (40% EtOAc/hexane) to give the compound **44** (1.68 g, 80% yield).

Compound 44

Physical State: colorless oil

^{1}H NMR (600 MHz, $CDCl_3$): δ 11.70 (s, 1H), 8.19 (t, *J* = 5.7 Hz, 1H), 7.68 (d, *J* = 8.3 Hz, 1H), 7.60 (d, *J* = 7.8 Hz, 1H), 7.43 – 7.39 (m, 2H), 7.39 – 7.32 (m, 7H), 7.32 – 7.27 (m, 1H), 7.21 (ddd, *J* = 8.4, 7.1, 1.2 Hz, 1H), 7.08 (t, *J* = 7.4 Hz, 1H), 6.93 (d, *J* = 7.8 Hz, 1H), 6.14 (d, *J* = 7.4 Hz, 1H), 5.46 (d, *J* = 9.5 Hz, 1H), 5.25 – 5.17 (m, 3H), 5.15 – 5.09 (m, 2H), 4.87 (ddd, *J* = 12.5, 8.8, 4.0 Hz, 1H), 4.29 (dd, *J* = 7.7, 5.0 Hz, 1H), 4.21 (td, *J* = 8.9, 4.1 Hz, 1H), 4.14 (ddd, *J* = 11.4, 7.4, 4.4 Hz, 1H), 3.36 (t, *J* = 12.7 Hz, 1H), 3.16 (dd, *J* = 13.0, 4.0 Hz, 1H), 3.09 (ddt, *J* = 26.4, 12.9, 6.2 Hz, 2H), 2.57 (ddd, *J* = 14.7, 10.6, 4.4 Hz, 1H), 2.17 (s, 2H), 1.88 (td, *J* = 6.3, 2.9 Hz, 1H), 1.74 (ddd, *J* = 14.2, 9.8, 4.3 Hz, 1H), 1.65 – 1.58 (m, 1H), 1.46 (s, 9H), 1.15 (ddt, *J* = 14.9, 7.4, 3.8 Hz, 1H), 1.06 – 0.86 (m, 24H), 0.81 (d, *J* = 6.9 Hz, 3H), 0.74 (t, *J* = 7.3 Hz, 3H).

^{13}C NMR (151 MHz, $CDCl_3$): δ 174.63, 171.53, 171.15, 170.64, 163.56, 156.07, 155.89, 153.89, 140.23, 136.72, 134.72, 134.69, 128.93, 128.84, 128.81, 128.63, 128.57, 128.47, 128.17, 127.64, 125.09, 122.06, 118.57, 114.12, 80.97, 68.41, 67.28, 58.76, 58.56, 53.46, 51.25, 40.80, 36.30, 36.01, 29.69, 28.41, 27.05, 24.51, 24.37, 24.34, 23.54, 22.02, 15.68, 11.58, 7.80, 5.30.

HRMS (ESI-TOF): calculated for $C_{56}H_{78}N_8NaO_{10}Si^+$ $[M+Na]^+$: 1073.5502, found: 1073.5507.

TLC: R_f = 0.6 (2:1 Hexane:EtOAc, $Ce_2(SO_4)_3$ in phosphomolybdic acid).

$[\alpha]^{25}_D$: –59.9 (*c* = 1.0, $CHCl_3$)

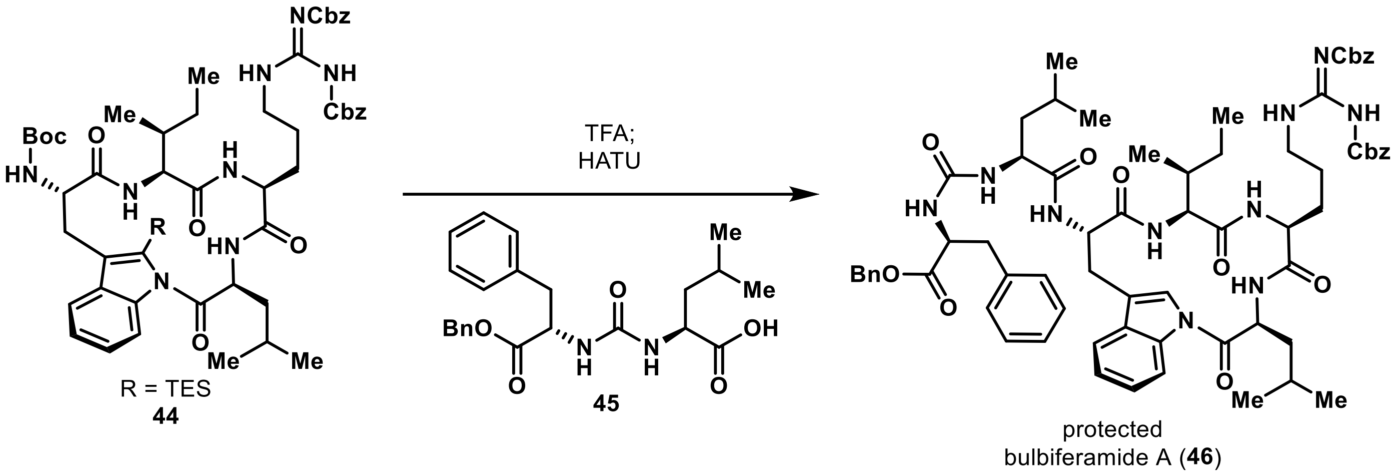

Compound **44** (1.68 g, 1.6 mmol) was dissolved in 27 mL DCM and TFA (3 mL) was added dropwise. The reaction mixture was stirred at rt for 1 h. Then 100 mL of toluene was added, and the solution was concentrated *in vacuo* (three times with toluene) to give the residue. The residue was used in the next step directly without further purification.

The crude and compound **45** (793 mg, 1.92 mmol, 1.2 equiv.) were dissolved in anhydrous DMF (20 mL). DIPEA (1.12 mL, 6.4 mmol, 4.0 equiv.) and HATU (730 mg, 1.92 mmol, 1.2 equiv.) were added sequentially. The mixture was allowed to stir at rt for 1 h then quenched by 0.5 M HCl (60 mL) and extracted with DCM (30 mL) three times. The organic layers were combined and washed with saturated *aq.* NaCl, dried over Na_2SO_4 and removed under reduced pressure to give the residue; the residue was purified by silica gel chromatography (5% MeOH/DCM) to give the compound **46** (1.5 g, 75% yield).

Compound 46

Physical State: white solid

^{1}H NMR (600 MHz, $CDCl_3$): δ 11.67 (s, 1H), 8.40 (d, J = 8.3 Hz, 2H), 8.30 (t, J = 5.6 Hz, 1H), 7.75 – 7.67 (m, 2H), 7.66 – 7.60 (m, 1H), 7.54 – 7.40 (m, 4H), 7.39 – 7.27 (m, 18H), 7.23 – 7.15 (m, 2H), 5.43 (s, 1H), 5.11 (d, J = 18.7 Hz, 6H), 4.75 (s, 1H), 4.44 (q, J = 8.0 Hz, 1H), 4.19 (s, 1H), 3.94 (t, J = 8.2 Hz, 1H), 3.38 (dq, J = 13.7, 6.7 Hz, 2H), 3.05 (d, J = 6.7 Hz, 3H), 2.03 (dt, J = 13.7, 7.0 Hz, 1H), 1.75 (td, J = 13.2, 12.2, 5.4 Hz, 1H), 1.57 (dt, J = 15.1, 7.5 Hz, 5H), 1.45 (s, 3H), 1.30 (m, 4H), 1.04 – 0.89 (m, 12H), 0.89 – 0.64 (m, 9H).

^{13}C NMR (151 MHz, $CDCl_3$): δ 172.33, 171.91, 170.50, 168.85, 163.68, 156.10, 155.28, 153.87, 140.56, 139.97, 139.71, 136.82, 136.45, 135.08, 134.68, 132.18, 129.60, 128.87, 128.77, 128.68, 128.58, 128.25, 125.74, 124.03, 123.42, 118.94, 116.92, 80.32, 77.53, 77.45, 74.99, 68.27, 67.23, 53.95, 51.90, 40.44, 39.65, 28.51, 28.42, 25.60, 24.86, 23.08, 22.51, 15.50, 10.73.

HRMS (ESI-TOF): calculated for $C_{68}H_{82}N_{10}NaO_{12}^+$ $[M+Na]^+$: 1253.6006, found: 1253.6027.

TLC: R_f = 0.5 (10:1 DCM:MeOH, $Ce_2(SO_4)_3$ in phosphomolybdic acid).

$[\alpha]^{25}_D$: –15.2 (c = 1.0, $CHCl_3$)

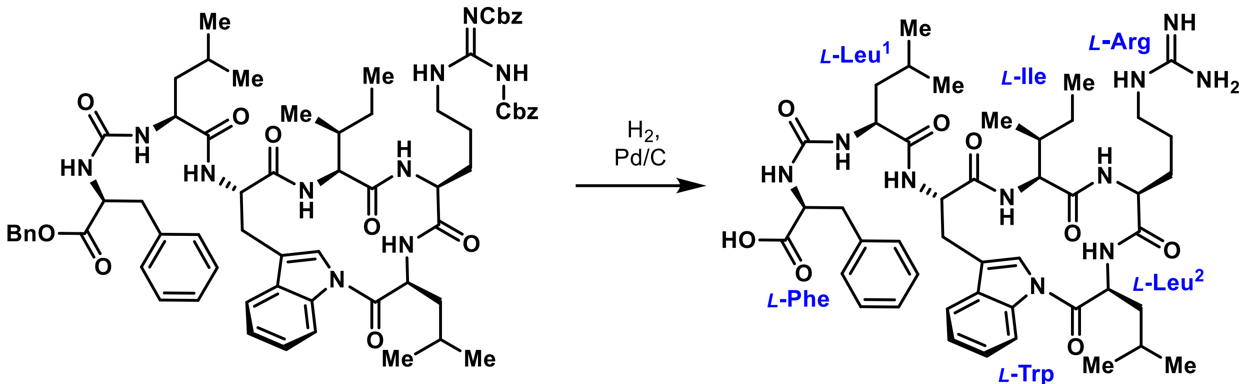

A solution of compound **46** (400 mg, 0.325 mmol, 1.0 equiv.) in MeOH (20 mL) was treated with AcOH (20 μL, 0.325 mmol, 1.0 equiv.) and Pd/C (200 mg). The reaction mixture was stirred at room temperature under hydrogen atmosphere for 12 h. Upon completion, the solid was removed by filtration, and the filtrate was concentrated *in vacuo*. The resulting residue was purified by preparative HPLC to afford the natural product **Bulbiferamide A** (**3**) (210 mg, 72% yield).

Compound 3

Physical State: white solid

^{1}H NMR (600 MHz, DMSO-*d*$_6$): δ 10.78 (s, 1H), 9.60 (d, *J* = 9.5 Hz, 1H), 9.10 (d, *J* = 6.9 Hz, 1H), 8.53 (d, *J* = 10.6 Hz, 1H), 8.43 – 8.30 (m, 1H), 7.58 (s, 1H), 7.53 (s, 1H), 7.50 – 7.46 (m, 1H), 7.40 – 6.70 (3H), 7.36 – 7.33 (m, 1H), 7.33 – 7.30 (m, 1H), 7.25 – 7.21 (m, 2H), 7.21 – 7.19 (m, 1H), 7.17 (d, *J* = 9.5 Hz, 1H), 7.03 (t, *J* = 7.4 Hz, 2H), 6.93 (t, *J* = 7.4 Hz, 1H), 4.64 – 4.57 (m, 1H), 4.54 (ddd, *J* = 12.0, 7.2, 3.7 Hz, 1H), 4.47 – 4.43 (m, 1H), 4.43 – 4.38 (m, 1H), 4.20 (q, *J* = 9.1, 8.4 Hz, 1H), 4.03 (dd, *J* = 10.5, 4.8 Hz, 1H), 3.33 (s, 1H), 3.09 (q, *J* = 7.3 Hz, 2H), 2.92 (s, 2H), 2.40 (d, *J* = 14.5 Hz, 1H), 2.09 (dt, *J* = 14.5, 7.3 Hz, 1H), 1.93 – 1.88 (m, 1H), 1.87 – 1.82 (m, 1H), 1.79 – 1.74 (m, 1H), 1.75 – 1.72 (m, 1H), 1.72 – 1.66 (m, 1H), 1.66 – 1.58 (m, 1H), 1.49 – 1.43 (m, 1H), 1.42 – 1.36 (m, 2H), 1.36 – 1.32 (m, 1H), 1.30 – 1.25 (m, 1H), 1.19 – 1.09 (m, 1H), 1.02 (d, *J* = 6.5 Hz, 3H), 0.90 (d, *J* = 6.5 Hz, 3H), 0.88 (d, *J* = 6.5 Hz, 3H), 0.86 (d, *J* = 6.5 Hz, 3H), 0.77 (t, *J* = 7.6 Hz, 4H), 0.76 (d, *J* = 7.1 Hz, 3H)

^{13}C NMR (151 MHz, DMSO-*d*$_6$): δ 176.10, 174.25, 171.75, 170.94, 170.70, 168.98, 156.63, 156.45, 138.28, 135.47, 130.18, 129.82, 127.15, 125.33, 124.99, 123.49, 120.94, 118.14, 117.48, 116.75, 57.76, 57.51, 55.22, 54.36, 50.21, 49.94, 43.51, 40.28, 40.03, 38.78, 36.52, 29.27, 25.20, 24.90, 24.84, 23.79, 23.58, 22.83, 22.18, 21.95, 21.88, 15.73, 11.52.

HRMS (ESI-TOF): calculated for $C_{45}H_{65}N_{10}O_8^+$ $[M+H]^+$: 873.4981, found: 873.4963; calculated for $C_{45}H_{64}N_{10}NaO_8^+$ $[M+Na]^+$: 895.4801, found: 895.4785.

$[\alpha]^{25}_D$: –11.9 (*c* = 0.1, MeOH)

^{1}H NMR Comparison of Bulbiferamide A (3)

	Reported [1] ^{1}H NMR (500 MHz, DMSO-d_6)	Synthetic (This work) ^{1}H NMR (600 MHz, DMSO-d_6)
L-Phe	4.44 (m, 1H)	4.45 (m, 1H)
	2.91 (s, 2H)	2.92 (s, 2H)
	7.21 (d, *J* = 7.4 Hz, 2H)	7.22 (d, *J* = 7.6 Hz, 2H)
	7.02 (dd, *J* = 7.4, 7.4 Hz, 2H)	7.03 (t, *J* = 7.4 Hz, 2H)
	6.93 (t, *J* = 7.4 Hz, 1H)	6.93 (t, *J* = 7.4 Hz, 1H)
	7.18 (m, 1H)	7.19 (m, 1H)
L-Leu[1]	4.60 (ddd, *J* = 8.2, 8.2, 8.2 Hz, 1H)	4.60 (m, 1H)
	1.38 (m, 2H)	1.39 (m, 2H)
	1.62 (m, 1H)	1.63 (m, 1H)
	0.90 (d, *J* = 6.7 Hz, 3H)	0.90 (d, *J* = 6.5 Hz, 3H)
	0.86 (d, *J* = 6.5 Hz, 3H)	0.86 (d, *J* = 6.5 Hz, 3H)
	7.15 (m, 1H)	7.16 (m, 1H)
L-Trp	4.53 (m, 1H)	4.54 (m, 1H)
	3.35 (1H)[a]	3.33 (1H)[a]
	2.39 (d, *J* = 13.7 Hz, 1H)	2.40 (d, *J* = 14.5 Hz, 1H)
	7.47 (1H)[b]	7.47 (1H)[b]
	7.31 (1H)[b]	7.32 (1H)[b]
	7.33 (1H)[b]	7.34 (1H)[b]
	8.34 (1H)[b]	8.35 (1H)[b]
	7.56 (s, 1H)	7.57 (s, 1H)
	9.09 (d, *J* = 6.3 Hz, 1H)	9.10 (d, *J* = 6.9 Hz, 1H)
L-Ile	4.03 (dd, *J* = 4.7, 10.4 Hz, 1H)	4.03 (dd, *J* = 4.8, 10.5 Hz, 1H)
	1.72 (m, 1H)	1.72 (m, 1H)
	1.26 (m, 1H)	1.27 (m, 1H)
	1.13 (m, 1H)	1.14 (m, 1H)
	0.76 (t, *J* = 7.2 Hz, 3H)	0.77 (t, *J* = 7.6 Hz, 3H)
	0.75 (d, *J* = 6.9 Hz, 3H)	0.76 (d, *J* = 7.1 Hz, 3H)
	8.52 (d, *J* = 10.7 Hz, 1H)	8.53 (d, *J* = 10.6 Hz, 1H)
L-Arg	4.20 (ddd, *J* = 8.2, 8.7, 9.1 Hz, 1H)	4.20 (q, *J* = 8.4, 9.1 Hz, 1H)
	1.90 (m, 1H)	1.91 (m, 1H)
	1.68 (m, 1H)	1.69 (m, 1H)
	1.44 (m, 1H)	1.45 (m, 1H)
	1.34 (m, 1H)	1.34 (m, 1H)
	3.09 (s, 2H)	3.09 (s, 2H)
	9.58 (d, *J* = 9.2 Hz, 1H)	9.60 (d, *J* = 9.5 Hz, 1H)
	7.52 (s, 1H)	7.53 (s, 1H)
	6.70 – 7.40 (3H)	6.70 – 7.40 (3H)
L-Leu[2]	4.42 (m, 1H)	4.43 (m, 1H)
	2.08 (ddd, *J* = 7.0, 7.0, 14.2 Hz, 1H)	2.09 (dt, *J* = 7.3, 14.5 Hz, 1H)
	1.85 (m, 1H)	1.85 (m, 1H)
	1.73 (m, 1H)	1.73 (m, 1H)
	0.88 (d, *J* = 6.6 Hz, 3H)	0.88 (d, *J* = 6.5 Hz, 3H)

	1.01 (d, J = 6.5 Hz, 3H)	1.02 (d, J = 6.5 Hz, 3H)
	10.75 (s 1H)	10.78 (s 1H)

[a]Overlapped with the H_2O signal. [b]Coupling constants could not be determined due to second-order effect.

[1] Lu, S.-Y.; Zhang, Z.-W.; Sharma, A. R.; Nakajima-Shimada, J.; Harunari, E.; Oku, N.; Trianto, A.; Igarashi, Y. *J. Nat. Prod.* **2023**, *86,* 1081−1086.

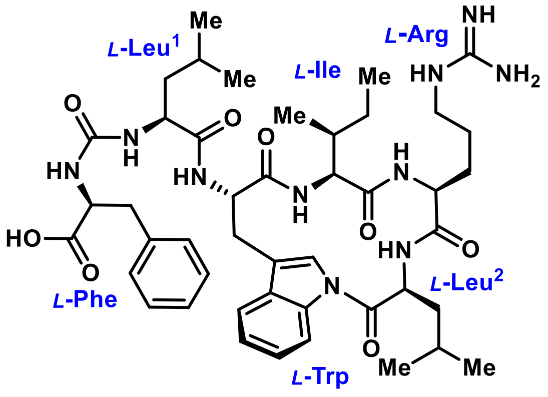

bulbiferamide A (**3**)

^{13}C NMR Comparison of Bulbiferamide A (3)

	Reported [1] ^{1}H NMR (125 MHz, DMSO-d_6)	Synthetic (This work) ^{1}H NMR (151 MHz, DMSO-d_6)
L-Phe	176.1	176.1
	55.2	55.2
	39.7	40.0
	138.3	138.3
	129.9	129.8
	127.2	127.2
	125.4	125.3
ureido	156.5	156.5
L-Leu^1	174.3	174.3
	50.2	50.2
	43.6	43.5
	23.8	23.8
	22.9	22.8
	21.9	21.9
L-Trp	169.0	169.0
	50.0	49.9
	24.8	24.8
	117.5	117.5
	130.2	130.2
	118.2	118.1
	123.5	123.5
	125.0	125.0
	116.8	116.8
	135.5	135.5
	121.0	121.0
L-Ile	170.7	170.7
	57.5	57.5
	36.6	36.5
	23.6	23.6
	11.6	11.5
	15.8	15.7
L-Arg	171.8	171.7
	54.4	54.4
	29.3	29.3
	25.2	25.2
	40.3	40.3
	156.7	156.7
L-Leu^2	171.0	171.0
	57.8	57.8
	38.8	38.8
	25.0	24.9
	22.2	22.2

	22.0	21.9

[1] Lu, S.-Y.; Zhang, Z.-W.; Sharma, A. R.; Nakajima-Shimada, J.; Harunari, E.; Oku, N.; Trianto, A.; Igarashi, Y. *J. Nat. Prod.* **2023**, *86,* 1081−1086.

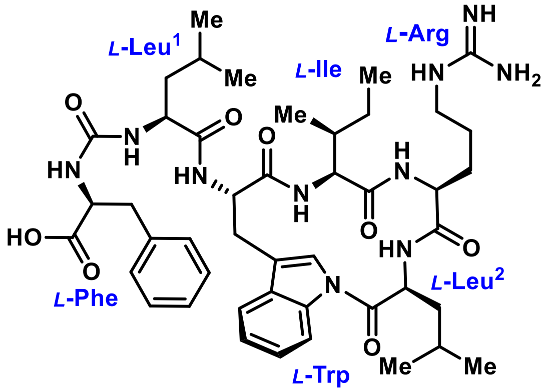

bulbiferamide A (**3**)

LCMS of Bulbiferamide A (3) after HPLC purification (negative scan)

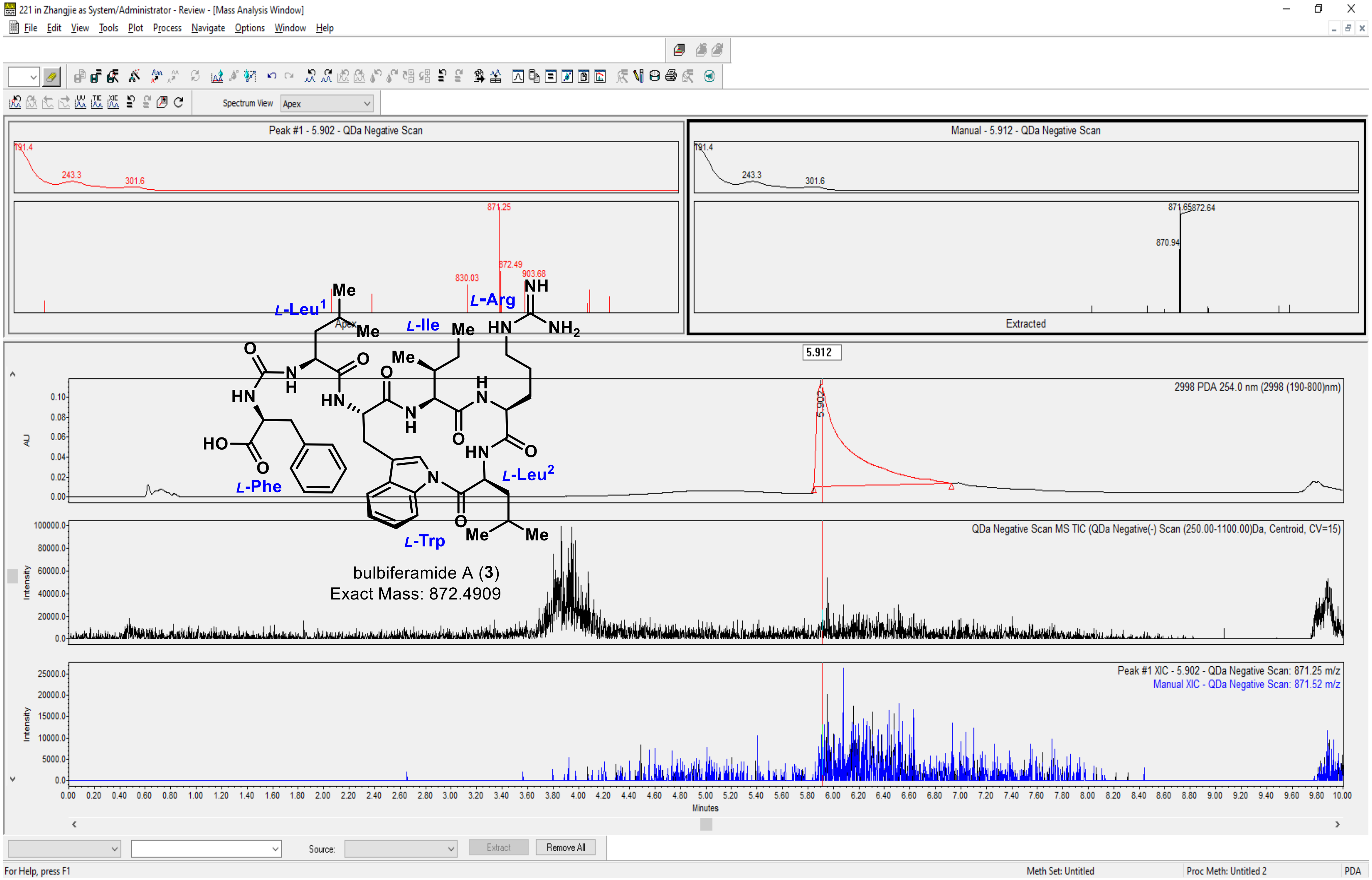

HRMS of Bulbiferamide A (3)

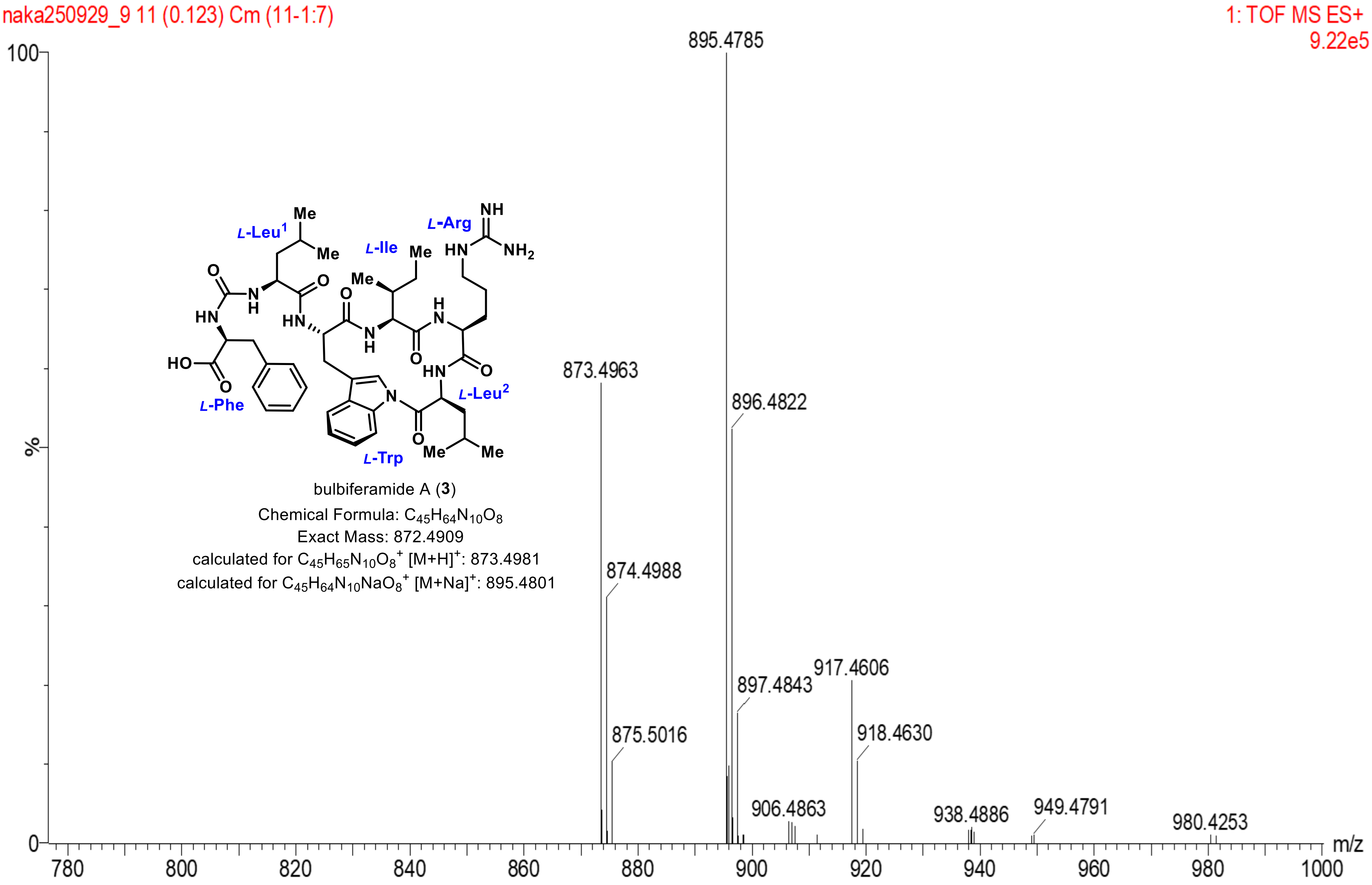

Synthesis of compounds 43 and 45

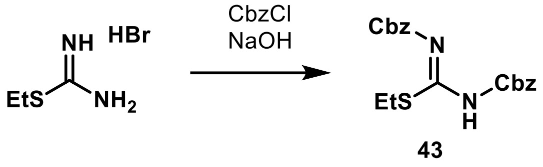

To a solution of *S*-Ethyl isothiouronium bromide (3.70 g, 20 mmol, 1.0 equiv.) in DCM (64 mL) and 5M NaOH aqueous solution (16 mL) was added CbzCl (8.4 mL, 60.0 mmol, 3.0 equiv.) dropwise at 0 °C while stirring vigorously. Then the reaction mixture was allowed to warm to rt and stir for 40 h. The mixture was added 100 mL water and extracted with DCM (50 mL) three times. Organic layers were combined, washed by saturated *aq.* NaCl, dried over Na_2SO_4 and removed under reduced pressure. The resulting residue was purified by silica gel chromatography (5% EtOAc/hexane) to give the compound **43** (2.4 g, 32% yield).

Compound 43
Physical State: colorless oil
^{1}H NMR (600 MHz, CDCl$_3$): δ 11.83 (s, 1H), 7.36 (q, *J* = 7.7 Hz, 10H), 5.18 (s, 4H), 3.05 (q, *J* = 7.4 Hz, 2H), 1.30 (t, *J* = 7.4 Hz, 3H).
^{13}C NMR (151 MHz, CDCl$_3$): δ 172.47, 161.27, 151.59, 135.97, 134.75, 128.88, 128.78, 128.66, 128.38, 68.48, 68.07, 25.71, 13.17.
HRMS (ESI-TOF): calculated for $C_{19}H_{20}N_2NaO_4S^+$ [M+Na]$^+$: 395.1036, found: 395.1051.
TLC: R$_f$ = 0.5 (10:1 Hexane:EtOAc, $Ce_2(SO_4)_3$ in phosphomolybdic acid).

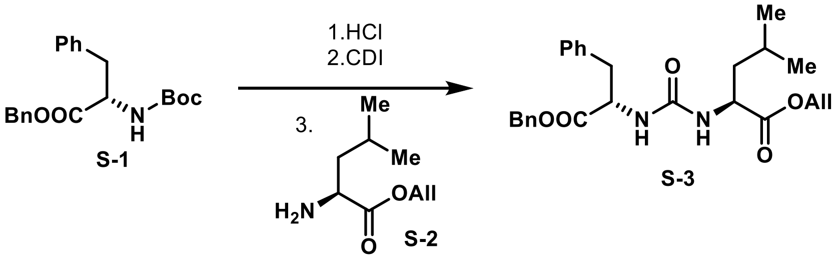

Benzyl (*tert*-butoxycarbonyl)-*L*-phenylalaninate **S-1** (7.7 g, 20.0 mmol, 1.0 equiv.) was dissolved in 50 mL DCM and 4M HCL dioxane (25 mL) was added dropwise. The reaction mixture was stirred at rt for 1 h. Then 100 mL of toluene was added and concentrated *in vacuo* to give the residue. The residue was used in the next step directly without further purification.

To a solution of the crude and CDI (3.26 g, 20.0 mmol, 1.0 equiv.) in DCM (50 mL) was added DIPEA (7.0 mL, 40.0 mmol, 2.0 equiv.) dropwise. The reaction mixture was stirred at rt for 10 h and quenched by 0.5 M HCl (100 mL), extracted with DCM (50 mL) three times. The organic layers were combined and washed with saturated *aq.* NaCl, dried over Na_2SO_4 and removed under reduced pressure to give the residue; the residue was used in the next step directly without further purification.

The crude and compound **S-2** (3.44 g, 20.0 mmol, 1.0 equiv.) were dissolved in DCM (100 mL) then DIPEA (7.0 mL, 40.0 mmol, 2.0 equiv.) was added dropwise. The reaction mixture was allowed to stir at rt for 12 h then quenched by 0.5 M HCl (100 mL) and extracted with DCM (60 mL) three times. The organic layers were combined and washed with saturated *aq.* NaCl, dried over Na_2SO_4 and removed under reduced pressure. The resulting residue was purified by silica gel chromatography (20% EtOAc/hexane) to give the compound **S-3** (7.99 g, 88% yield).

Compound S-3
Physical State: white solid
^{1}H NMR (600 MHz, $CDCl_3$): δ 7.33 (dd, J = 5.0, 1.9 Hz, 3H), 7.29 – 7.23 (m, 2H), 7.18 – 7.11 (m, 3H), 7.00 – 6.93 (m, 2H), 5.88 (ddt, J = 17.2, 10.4, 5.7 Hz, 1H), 5.40 (ddd, J = 13.4, 8.5, 2.2 Hz, 2H), 5.32 (dq, J = 17.2, 1.5 Hz, 1H), 5.23 (dq, J = 10.5, 1.3 Hz, 1H), 5.11 (d, J = 12.2 Hz, 1H), 4.98 (d, J = 12.2 Hz, 1H), 4.90 – 4.81 (m, 1H), 4.60 (ddt, J = 13.2, 5.8, 1.4 Hz, 1H), 4.56 – 4.49 (m, 2H), 3.13 – 2.96 (m, 2H), 1.66 (dh, J = 8.5, 6.5 Hz, 1H), 1.54 (ddd, J = 13.7, 8.4, 5.2 Hz, 1H), 1.45 – 1.35 (m, 1H), 0.90 (dd, J = 9.3, 6.6 Hz, 6H).
^{13}C NMR (151 MHz, $CDCl_3$): δ 174.53, 172.62, 156.70, 135.99, 135.24, 131.79, 129.64, 129.58, 128.67, 128.55, 128.49, 127.00, 118.70, 67.23, 65.96, 54.00, 51.61, 42.12, 38.69, 29.79, 24.84, 22.98, 21.96.
HRMS (ESI-TOF): calculated for $C_{26}H_{32}N_2NaO_5^+$ $[M+Na]^+$: 475.2202, found: 475.2211.
TLC: R_f = 0.5 (4:1 Hexane:EtOAc, $Ce_2(SO_4)_3$ in phosphomolybdic acid).
$[\alpha]^{25}_D$: +15.2 (c = 1.0, $CHCl_3$)

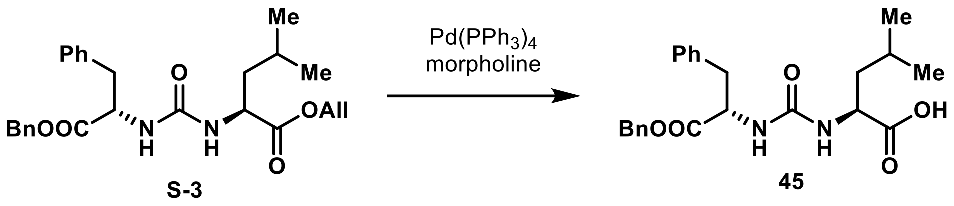

To a solution of compound **S-3** (1.8 g, 4.0 mmol, 1.0 equiv.) in THF (20 mL) was added $Pd(PPh_3)_4$ (230 mg, 0.2 mmol, 5 mol%) and morpholine (2.0 mL, 24.0 mmol, 6.0 equiv.). The reaction mixture was allowed to stir at rt under nitrogen atmosphere for 1 h. When complete, the mixture was added 0.5 M HCl (80 mL), extracted with DCM (30 mL) three times. The organic layers were combined and washed with saturated *aq.* NaCl, dried over Na_2SO_4 and removed under reduced pressure. The resulting residue was used in the next step directly without further purification.

Synthesis of cyclization compounds 47–86; 95–97 and 106–109

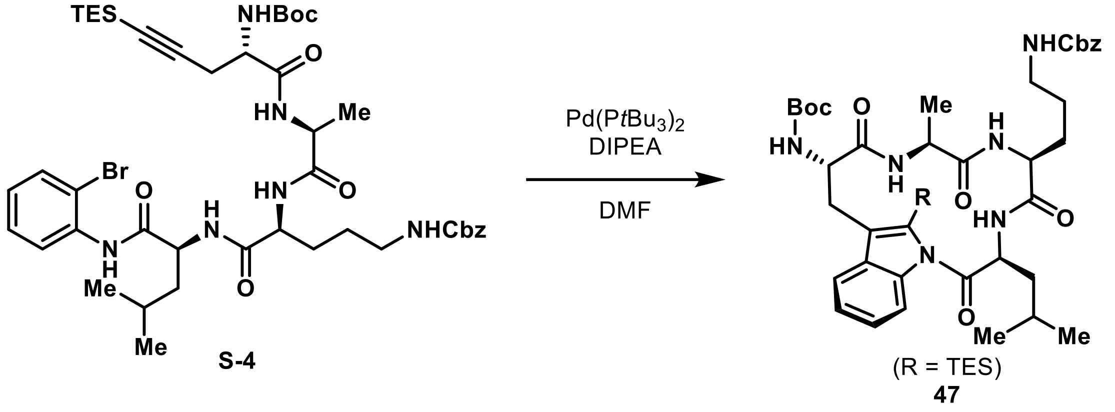

To a solution of compound **S-4** (729 mg, 0.798 mmol, 1.0 equiv.) in 16 mL anhydrous DMF was added DIPEA (0.42 mL, 2.4 mmol, 3.0 equiv.) and Pd(tBu$_3$P)$_2$ (40 mg, 0.08 mmol, 0.1 equiv.). The reaction mixture was stirred at 100 °C under nitrogen atmosphere for 12 h. After the consumption of the starting material, the mixture was diluted with 50 mL EtOAc, washed with 0.5 M HCl and saturated *aq*. NaCl. The organic layer was dried over Na_2SO_4 and removed under reduced pressure to give the residue; the residue was purified by silica gel chromatography (30% EtOAc/hexane) to give the compound **47** (359 mg, 54% yield).

Compound 47
Physical State: amorphous solid
^{1}H NMR (400 MHz, CDCl$_3$): δ 7.72 (dd, J = 12.5, 8.1 Hz, 2H), 7.51 (d, J = 7.8 Hz, 1H), 7.46 – 7.31 (m, 5H), 7.20 (t, J = 7.7 Hz, 1H), 7.10 (t, J = 7.5 Hz, 1H), 6.20 (d, J = 7.4 Hz, 1H), 5.70 (d, J = 9.7 Hz, 1H), 5.31 – 5.19 (m, 3H), 5.00 (ddd, J = 12.5, 9.1, 3.7 Hz, 1H), 4.89 (t, J = 6.1 Hz, 1H), 4.56 – 4.41 (m, 1H), 4.35 – 4.13 (m, 2H), 3.38 (t, J = 12.6 Hz, 1H), 3.17 (dd, J = 13.1, 3.8 Hz, 1H), 2.72 (d, J = 14.0 Hz, 1H), 2.55 (td, J = 10.5, 5.3 Hz, 1H), 2.44 (d, J = 26.4 Hz, 2H), 1.77 (ddd, J = 14.2, 9.8, 4.3 Hz, 1H), 1.62 (ddd, J = 10.0, 6.7, 4.0 Hz, 1H), 1.49 (s, 9H), 1.46 – 1.39 (m, 2H), 1.25 (d, J = 7.3 Hz, 3H), 0.96 (dq, J = 11.1, 5.5, 4.6 Hz, 21H), 0.83 – 0.75 (m, 1H).
^{13}C NMR (101 MHz, CDCl$_3$): δ 173.84, 171.82, 170.09, 170.00, 156.08, 154.88, 139.40, 135.90, 133.43, 128.77, 127.90, 127.51, 127.42, 127.20, 124.28, 121.35, 118.20, 113.18, 79.98, 66.41, 57.56, 52.99, 50.47, 48.63, 39.85, 35.62, 29.71, 27.69, 24.20, 23.95, 23.78, 22.87, 21.27, 16.97, 7.10, 4.61.
HRMS (ESI-TOF): calculated for $C_{44}H_{64}N_6NaO_8Si^+$ $[M+Na]^+$: 855.4447, found: 855.4452.
TLC: R$_f$ = 0.5 (1:1 Hexane:EtOAc, $Ce_2(SO_4)_3$ in phosphomolybdic acid).
[α]$^{25}_D$: –112.5 (c = 1.0, CHCl$_3$)

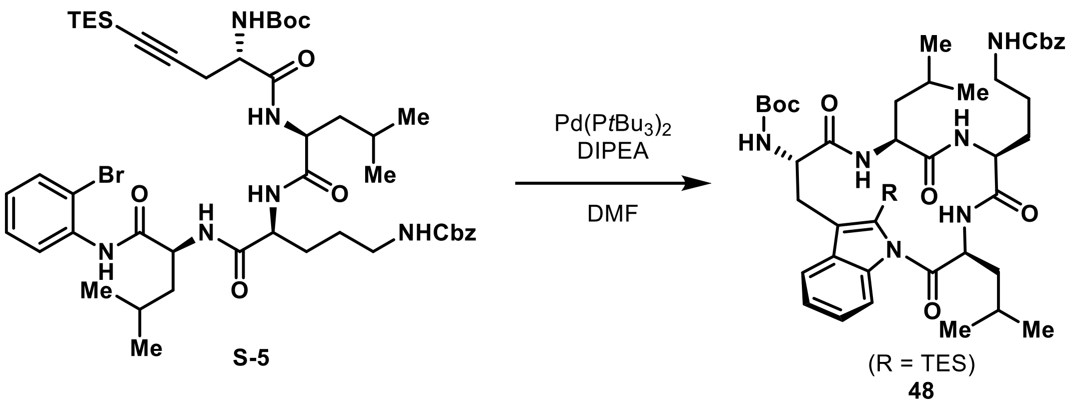

To a solution of compound **S-5** (697 mg, 0.730 mmol, 1.0 equiv.) in 15 mL anhydrous DMF was added DIPEA (0.38 mL, 2.2 mmol, 3.0 equiv.) and Pd(*t*$Bu_3P)_2$ (35 mg, 0.07 mmol, 0.1 equiv.). The reaction mixture was stirred at 100 °C under nitrogen atmosphere for 12 h. After the consumption of the starting material, the mixture was diluted with 50 mL EtOAc, washed with 0.5 M HCl and saturated *aq.* NaCl. The organic layer was dried over Na_2SO_4 and removed under reduced pressure to give the residue; the residue was purified by silica gel chromatography (20% EtOAc/hexane) to give the compound **48** (300 mg, 47% yield).

Compound 48
Physical State: amorphous solid
^{1}H NMR (400 MHz, $CDCl_3$): δ 7.69 (dd, *J* = 15.3, 8.1 Hz, 2H), 7.52 – 7.29 (m, 6H), 7.20 (t, *J* = 7.7 Hz, 1H), 7.11 (t, *J* = 7.4 Hz, 1H), 6.17 (d, *J* = 7.3 Hz, 1H), 5.69 (d, *J* = 9.7 Hz, 1H), 5.30 (d, *J* = 12.4 Hz, 1H), 5.20 (dd, *J* = 10.5, 3.7 Hz, 2H), 4.97 (ddd, *J* = 12.6, 9.0, 3.8 Hz, 1H), 4.82 (t, *J* = 5.9 Hz, 1H), 4.44 (d, *J* = 10.3 Hz, 1H), 4.30 (dt, *J* = 10.3, 5.4 Hz, 1H), 4.17 (ddd, *J* = 11.3, 7.3, 4.2 Hz, 1H), 3.41 (t, *J* = 12.7 Hz, 1H), 3.15 (dd, *J* = 13.0, 3.8 Hz, 1H), 2.72 (d, *J* = 14.8 Hz, 1H), 2.58 (ddd, *J* = 14.6, 10.7, 4.2 Hz, 1H), 2.47 (s, 1H), 2.33 (s, 1H), 1.76 (ddd, *J* = 14.2, 9.8, 4.2 Hz, 1H), 1.69 – 1.56 (m, 3H), 1.47 (s, 9H), 1.43 – 1.28 (m, 2H), 1.07 – 1.02 (m, 2H), 1.00 – 0.78 (m, 27H).
^{13}C NMR (101 MHz, $CDCl_3$): δ 173.85, 171.84, 170.53, 170.17, 156.04, 154.84, 139.43, 135.90, 133.60, 128.51, 127.89, 127.52, 127.14, 124.24, 121.32, 118.03, 113.23, 79.93, 66.36, 57.66, 52.90, 51.71, 50.19, 39.85, 39.62, 35.45, 29.21, 27.62, 24.11, 23.78, 22.87, 22.52, 21.25, 20.38, 7.12, 4.65.
HRMS (ESI-TOF): calculated for $C_{47}H_{70}N_6NaO_8Si^+$ $[M+Na]^+$: 897.4919, found: 897.4924.
TLC: R_f = 0.5 (2:1 Hexane:EtOAc, $Ce_2(SO_4)_3$ in phosphomolybdic acid).
$[\alpha]^{25}_D$: –88.8 (*c* = 1.0, $CHCl_3$)

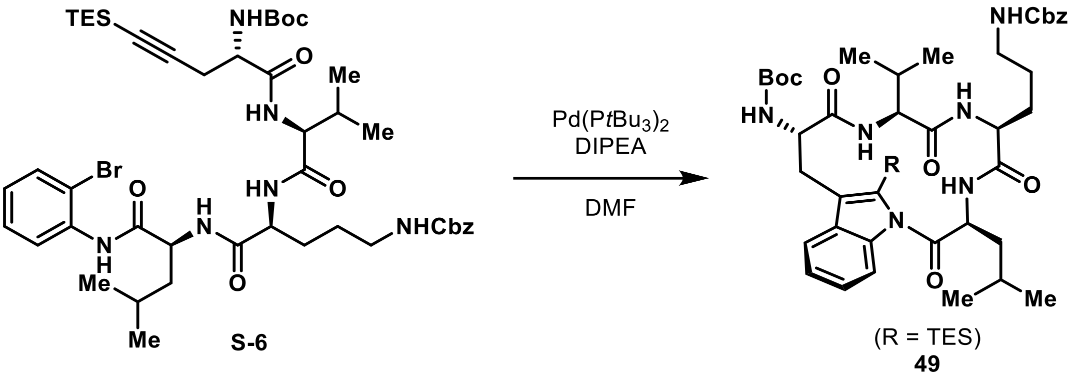

To a solution of compound **S-6** (603 mg, 0.641 mmol, 1.0 equiv.) in 13 mL anhydrous DMF was added DIPEA (0.33 mL, 1.9 mmol, 3.0 equiv.) and Pd(*t*$Bu_3P)_2$ (30 mg, 0.06 mmol, 0.1 equiv.). The reaction mixture was stirred at 100 °C under nitrogen atmosphere for 12 h. After the consumption of the starting material, the mixture was diluted with 39 mL EtOAc, washed with 0.5 M HCl and saturated *aq.* NaCl. The organic layer was dried over Na_2SO_4 and removed under reduced pressure to give the residue; the residue was purified by silica gel chromatography (20% EtOAc/hexane) to give the compound **49** (320 mg, 58% yield).

Compound 49
Physical State: amorphous solid
^{1}H NMR (400 MHz, $CDCl_3$): δ 7.74 (d, *J* = 8.3 Hz, 1H), 7.68 (d, *J* = 7.8 Hz, 1H), 7.49 – 7.29 (m, 6H), 7.19 (ddd, *J* = 8.4, 7.1, 1.2 Hz, 1H), 7.11 (t, *J* = 7.4 Hz, 1H), 6.25 (d, *J* = 7.4 Hz, 1H), 5.96 (d, *J* = 9.5 Hz, 1H), 5.23 (q, *J* = 13.3, 11.4 Hz, 3H), 4.95 (ddd, *J* = 12.3, 8.9, 3.5 Hz, 1H), 4.84 (t, *J* = 5.9 Hz, 1H), 4.39 (q, *J* = 8.3, 7.7 Hz, 2H), 4.21 (ddd, *J* = 11.3, 7.5, 4.3 Hz, 1H), 3.41 (t, *J* = 12.5 Hz, 1H), 3.15 (dd, *J* = 13.0, 3.6 Hz, 1H), 2.67 – 2.45 (m, 2H), 2.32 (s, 1H), 2.19 (dq, *J* = 13.3, 6.6 Hz, 2H), 1.78 (ddd, *J* = 14.2, 9.8, 4.2 Hz, 1H), 1.65 – 1.59

(m, 1H), 1.47 (s, 9H), 1.35 (t, J = 6.5 Hz, 1H), 1.32 – 1.22 (m, 1H), 1.07 – 1.03 (m, 1H), 1.02 – 0.86 (m, 24H), 0.73 (d, J = 6.8 Hz, 3H).

^{13}C NMR (101 MHz, CDCl$_3$): δ 173.80, 171.02, 170.88, 170.28, 156.04, 154.85, 139.29, 135.97, 128.73, 127.88, 127.46, 127.36, 124.20, 121.33, 118.03, 113.29, 79.82, 66.39, 58.25, 57.23, 53.28, 49.76, 39.58, 35.57, 29.30, 29.05, 27.69, 23.87, 23.82, 23.38, 22.89, 21.25, 18.51, 16.60, 7.13, 4.75.

HRMS (ESI-TOF): calculated for $C_{46}H_{68}N_6NaO_8Si^+$ [M+Na]$^+$: 883.4760, found: 883.4752.

TLC: R_f = 0.4 (2:1 Hexane:EtOAc, $Ce_2(SO_4)_3$ in phosphomolybdic acid).

[α]$^{25}_D$: –90.5 (c = 1.0, CHCl$_3$)

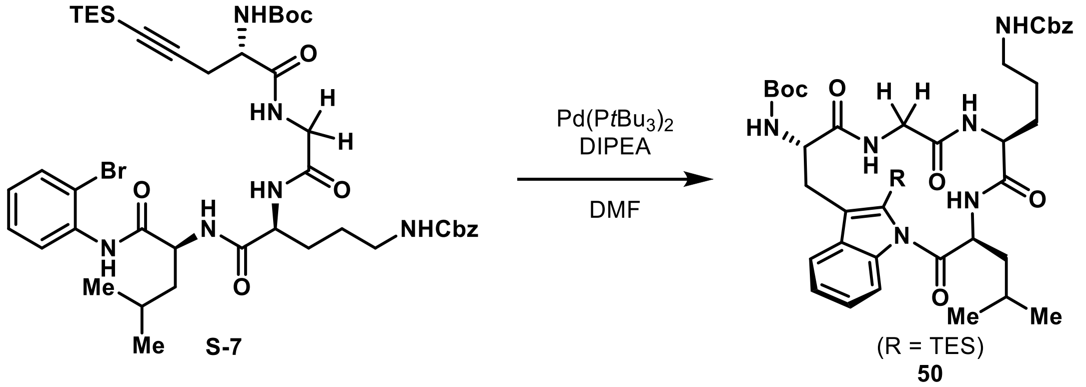

To a solution of compound **S-7** (680 mg, 0.755 mmol, 1.0 equiv.) in 15 mL anhydrous DMF was added DIPEA (0.38 mL, 2.2 mmol, 3.0 equiv.) and Pd(tBu$_3$P)$_2$ (40 mg, 0.08 mmol, 0.1 equiv.). The reaction mixture was stirred at 100 °C under nitrogen atmosphere for 12 h. After the consumption of the starting material, the mixture was diluted with 45 mL EtOAc, washed with 0.5 M HCl and saturated *aq.* NaCl. The organic layer was dried over Na_2SO_4 and removed under reduced pressure to give the residue; the residue was purified by silica gel chromatography (40% EtOAc/hexane) to give the compound **50** (309 mg, 50% yield).

Compound 50

Physical State: amorphous solid

^{1}H NMR (400 MHz, CDCl$_3$): δ 7.78 – 7.68 (m, 2H), 7.44 (d, J = 7.4 Hz, 1H), 7.38 (t, J = 7.3 Hz, 2H), 7.36 – 7.30 (m, 2H), 7.21 (t, J = 7.7 Hz, 1H), 7.10 (t, J = 7.5 Hz, 1H), 6.25 (d, J = 7.3 Hz, 1H), 5.46 (d, J = 9.9 Hz, 1H), 5.29 – 5.21 (m, 2H), 5.07 (d, J = 12.4 Hz, 1H), 4.86 (s, 1H), 4.36 (ddd, J = 26.6, 12.6, 6.2 Hz, 2H), 4.19 (ddd, J = 11.3, 7.4, 4.2 Hz, 1H), 3.41 (t, J = 12.6 Hz, 1H), 3.29 (d, J = 17.2 Hz, 1H), 3.17 (dd, J = 13.1, 3.8 Hz, 1H), 2.78 – 2.67 (m, 1H), 2.63 – 2.45 (m, 2H), 2.32 (s, 1H), 1.76 (ddd, J = 14.2, 9.9, 4.1 Hz, 1H), 1.59 (ddd, J = 9.8, 6.5, 3.6 Hz, 2H), 1.49 (s, 9H), 1.45 – 1.37 (m, 2H), 1.08 – 0.84 (m, 21H), 0.72 (s, 1H), 0.58 (dt, J = 18.9, 8.1 Hz, 1H).

^{13}C NMR (101 MHz, CDCl$_3$): δ 173.83, 170.62, 169.86, 168.44, 156.12, 154.71, 139.38, 135.85, 127.94, 127.56, 127.44, 127.14, 124.33, 121.41, 118.22, 113.27, 80.00, 66.39, 57.59, 52.90, 50.43, 41.99, 39.95, 35.56, 30.01, 27.75, 24.02, 23.74, 22.90, 21.19, 7.10, 7.05, 4.62.

HRMS (ESI-TOF): calculated for $C_{43}H_{62}N_6NaO_8Si^+$ [M+Na]$^+$: 841.4291, found: 841.4291.

TLC: R_f = 0.4 (1:1 Hexane:EtOAc, $Ce_2(SO_4)_3$ in phosphomolybdic acid).

[α]$^{25}_D$: –33.0 (c = 1.0, CHCl$_3$)

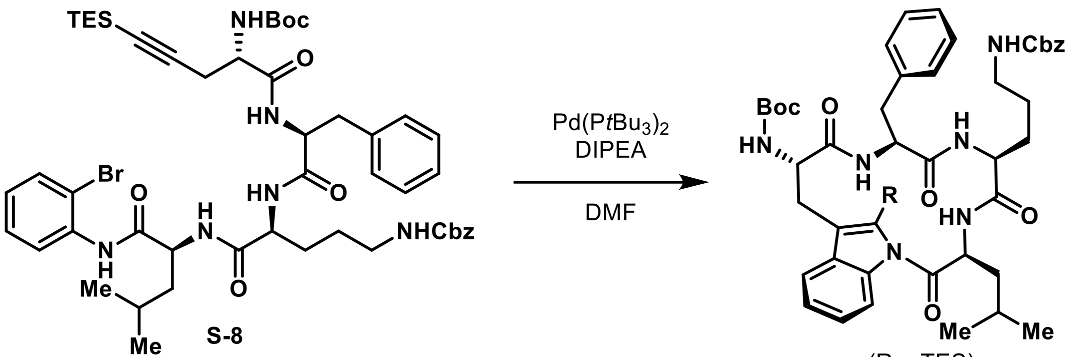

To a solution of compound **S-8** (692 mg, 0.700 mmol, 1.0 equiv.) in 14 mL anhydrous DMF was added DIPEA (0.37 mL, 2.1 mmol, 3.0 equiv.) and Pd(tBu$_3$P)$_2$ (35 mg, 0.07 mmol, 0.1 equiv.). The reaction mixture was stirred at 100 °C under nitrogen atmosphere for 12 h. After the consumption of the starting material, the mixture was diluted with 42 mL EtOAc, washed with 0.5 M HCl and saturated *aq.* NaCl. The organic layer was dried over Na_2SO_4 and removed under reduced pressure to give the residue; the residue was purified by silica gel chromatography (20% EtOAc/hexane) to give the compound **51** (337 mg, 53% yield).

Compound 51
Physical State: amorphous solid
^{1}H NMR (400 MHz, CDCl$_3$): δ 7.70 (dd, J = 8.3, 4.0 Hz, 2H), 7.62 (d, J = 8.7 Hz, 1H), 7.50 (d, J = 7.5 Hz, 2H), 7.41 (t, J = 7.2 Hz, 2H), 7.37 – 7.32 (m, 1H), 7.22 – 7.12 (m, 6H), 7.07 (t, J = 7.5 Hz, 1H), 6.14 (d, J = 7.4 Hz, 1H), 5.69 (d, J = 9.7 Hz, 1H), 5.41 – 5.22 (m, 2H), 5.06 (d, J = 8.9 Hz, 1H), 5.00 – 4.90 (m, 1H), 4.86 – 4.65 (m, 2H), 4.35 (dt, J = 10.1, 5.0 Hz, 1H), 4.20 (ddd, J = 11.3, 7.5, 4.3 Hz, 1H), 3.35 (t, J = 12.5 Hz, 1H), 3.12 (dd, J = 13.1, 3.5 Hz, 1H), 3.04 (dd, J = 14.0, 4.5 Hz, 1H), 2.95 (dd, J = 13.8, 8.1 Hz, 1H), 2.62 – 2.41 (m, 2H), 2.20 (s, 1H), 1.78 (ddd, J = 14.2, 9.7, 4.2 Hz, 1H), 1.61 (ddd, J = 12.6, 6.8, 3.7 Hz, 1H), 1.49 (s, 9H), 1.31 – 1.13 (m, 2H), 1.05 – 0.84 (m, 21H), 0.65 (s, 1H), 0.47 (h, J = 6.6 Hz, 1H).
^{13}C NMR (101 MHz, CDCl$_3$): δ 173.70, 170.31, 170.26, 169.94, 155.88, 154.61, 139.29, 136.00, 135.35, 128.92, 128.74, 127.91, 127.89, 127.52, 127.39, 127.31, 126.36, 124.31, 121.32, 118.21, 113.17, 79.69, 66.38, 57.37, 53.61, 53.09, 50.22, 39.74, 36.54, 35.52, 29.83, 27.78, 23.79, 23.57, 23.21, 22.88, 21.27, 7.11, 4.71.
HRMS (ESI-TOF): calculated for $C_{50}H_{68}N_6NaO_8Si^+$ [M+Na]$^+$: 931.4760, found: 931.4756.
TLC: R_f = 0.5 (3:1 Hexane:EtOAc, $Ce_2(SO_4)_3$ in phosphomolybdic acid).
[α]$^{25}_{D}$: –89.5 (c = 1.0, $CHCl_3$)

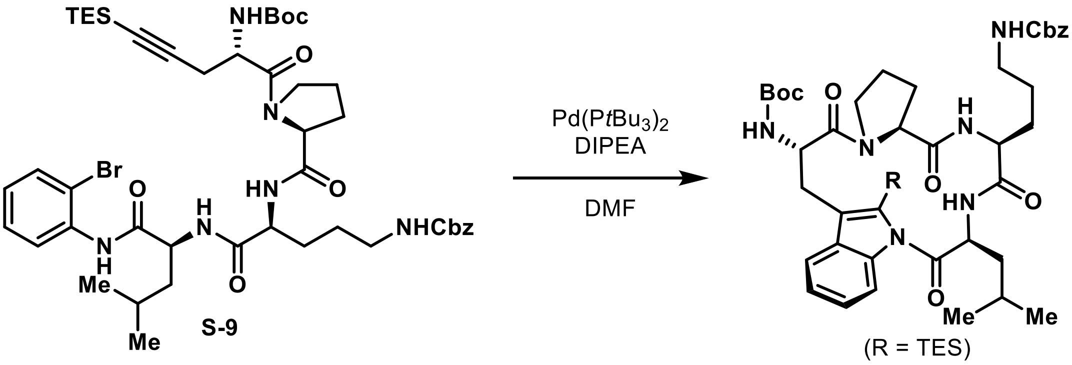

To a solution of compound **S-9** (495 mg, 0.527 mmol, 1.0 equiv.) in 11 mL anhydrous DMF was added DIPEA (0.28 mL, 1.6 mmol, 3.0 equiv.) and Pd(tBu$_3$P)$_2$ (25 mg, 0.05 mmol, 0.1 equiv.). The reaction mixture was

stirred at 100 °C under nitrogen atmosphere for 12 h. After the consumption of the starting material, the mixture was diluted with 33 mL EtOAc, washed with 0.5 M HCl and saturated *aq.* NaCl. The organic layer was dried over Na_2SO_4 and removed under reduced pressure to give the residue; the residue was purified by silica gel chromatography (30% EtOAc/hexane) to give the compound **52** (299 mg, 66% yield).

Compound 52

Physical State: amorphous solid

^{1}H NMR (400 MHz, $CDCl_3$): δ 7.78 (d, *J* = 7.7 Hz, 1H), 7.71 (d, *J* = 8.2 Hz, 1H), 7.42 – 7.29 (m, 6H), 7.25 – 7.14 (m, 2H), 6.19 (d, *J* = 7.3 Hz, 1H), 5.50 (td, *J* = 10.9, 4.4 Hz, 1H), 5.13 – 5.04 (m, 3H), 4.85 – 4.71 (m, 2H), 4.50 (dd, *J* = 8.5, 3.3 Hz, 1H), 4.28 (dt, *J* = 10.6, 5.4 Hz, 1H), 4.16 (ddd, *J* = 11.3, 7.3, 4.4 Hz, 1H), 3.78 (q, *J* = 9.1 Hz, 1H), 3.51 (dt, *J* = 9.8, 5.9 Hz, 1H), 3.35 (t, *J* = 12.4 Hz, 1H), 3.20 (dd, *J* = 13.1, 4.5 Hz, 1H), 2.91 (p, *J* = 6.8 Hz, 2H), 2.58 (ddd, *J* = 13.5, 10.1, 3.8 Hz, 1H), 2.02 (dp, *J* = 10.2, 3.4 Hz, 2H), 1.94 (s, 2H), 1.73 (dq, *J* = 9.8, 4.8, 4.2 Hz, 1H), 1.61 (dt, *J* = 7.0, 3.4 Hz, 1H), 1.53 (s, 9H), 1.44 (t, *J* = 5.0 Hz, 1H), 1.06 – 1.02 (m, 1H), 0.99 – 0.74 (m, 21H), 0.54 (q, *J* = 8.1 Hz, 1H).

^{13}C NMR (101 MHz, $CDCl_3$): δ 173.56, 170.25, 169.65, 169.48, 155.60, 154.40, 139.54, 134.44, 128.91, 127.94, 127.52, 126.77, 124.33, 121.60, 117.93, 113.29, 80.28, 65.95, 59.99, 58.09, 50.34, 49.98, 46.95, 39.81, 35.52, 30.86, 28.99, 27.73, 26.85, 23.91, 23.73, 23.69, 22.87, 21.24, 7.08, 4.40.

HRMS (ESI-TOF): calculated for $C_{46}H_{66}N_6NaO_8Si^+$ $[M+Na]^+$: 881.4604, found: 881.4606.

TLC: R_f = 0.4 (2:1 Hexane:EtOAc, $Ce_2(SO_4)_3$ in phosphomolybdic acid).

$[\alpha]^{25}_D$: –83.1 (*c* = 1.0, $CHCl_3$)

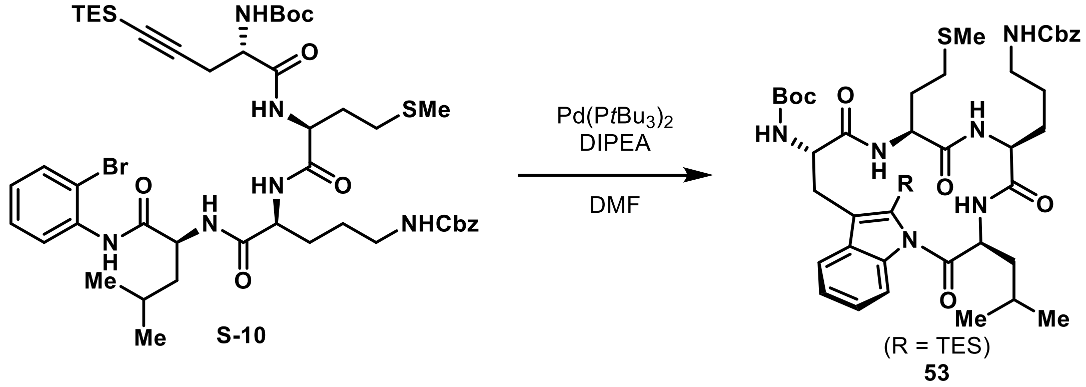

To a solution of compound **S-10** (775 mg, 0.797 mmol, 1.0 equiv.) in 16 mL anhydrous DMF was added DIPEA (0.42 mL, 2.4 mmol, 3.0 equiv.) and Pd(tBu$_3$P)$_2$ (40 mg, 0.08 mmol, 0.1 equiv.). The reaction mixture was stirred at 100 °C under nitrogen atmosphere for 12 h. After the consumption of the starting material, the mixture was diluted with 48 mL EtOAc, washed with 0.5 M HCl and saturated *aq.* NaCl. The organic layer was dried over Na_2SO_4 and removed under reduced pressure to give the residue; the residue was purified by silica gel chromatography (25% EtOAc/hexane) to give the compound **53** (249 mg, 35% yield).

Compound 53

Physical State: amorphous solid

^{1}H NMR (600 MHz, $CDCl_3$): δ 7.68 (d, *J* = 8.4 Hz, 1H), 7.64 (d, *J* = 8.0 Hz, 1H), 7.43 (d, *J* = 7.6 Hz, 2H), 7.37 (dd, *J* = 8.4, 6.7 Hz, 2H), 7.34 – 7.29 (m, 1H), 7.19 (t, *J* = 7.5 Hz, 1H), 7.08 (t, *J* = 7.6 Hz, 1H), 6.15 (d, *J* = 7.5 Hz, 1H), 5.69 (d, *J* = 9.7 Hz, 1H), 5.26 (d, *J* = 12.4 Hz, 1H), 5.23 – 5.16 (m, 2H), 4.95 (ddd, *J* = 12.6, 8.9, 3.8 Hz, 1H), 4.79 (s, 1H), 4.58 (d, *J* = 10.8 Hz, 1H), 4.30 (dt, *J* = 10.7, 5.4 Hz, 1H), 4.16 (ddd, *J* = 11.4, 7.5, 4.3 Hz, 1H), 3.38 (t, *J* = 12.7 Hz, 1H), 3.13 (dd, *J* = 13.1, 3.8 Hz, 1H), 2.69 (s, 1H), 2.54 (tdd, *J* = 13.7, 9.7, 4.4 Hz, 2H), 2.41 (dt, *J* = 13.4, 8.0 Hz, 2H), 2.14 (dtt, *J* = 12.2, 8.0, 3.8 Hz, 1H), 2.09 – 2.04 (m, 2H),

2.02 (s, 3H), 1.74 (dddd, J = 16.8, 13.3, 9.6, 4.6 Hz, 2H), 1.59 (ddq, J = 13.0, 6.5, 3.6, 3.0 Hz, 1H), 1.46 (s, 9H), 1.42 – 1.37 (m, 1H), 1.02 (d, J = 7.2 Hz, 2H), 1.00 – 0.84 (m, 21H).
^{13}C NMR (151 MHz, CDCl$_3$): δ 174.58, 171.62, 171.36, 170.73, 156.83, 155.56, 140.11, 136.58, 128.64, 128.28, 128.19, 125.00, 122.07, 118.78, 113.99, 80.70, 67.16, 58.33, 53.84, 52.75, 51.10, 40.64, 36.25, 30.88, 30.19, 30.13, 28.42, 24.78, 24.50, 23.57, 21.95, 15.14, 7.81, 5.37.
HRMS (ESI-TOF): calculated for $C_{46}H_{68}N_6NaO_8SSi^+$ [M+Na]$^+$: 915.4481, found: 915.4484.
TLC: R_f = 0.6 (2:1 Hexane:EtOAc, $Ce_2(SO_4)_3$ in phosphomolybdic acid).
[α]$^{25}_D$: –42.9 (c = 1.0, CHCl$_3$)

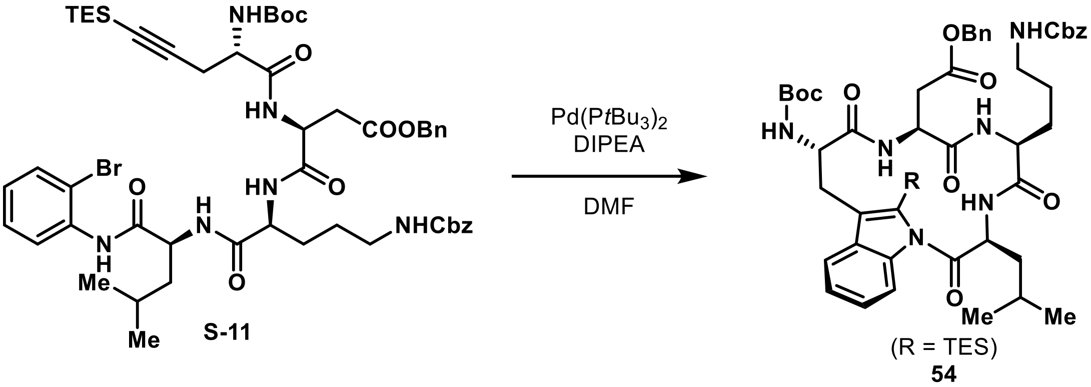

To a solution of compound **S-11** (558 mg, 0.533 mmol, 1.0 equiv.) in 12 mL anhydrous DMF was added DIPEA (0.28 mL, 1.6 mmol, 3.0 equiv.) and Pd(tBu$_3$P)$_2$ (25 mg, 0.05 mmol, 0.1 equiv.). The reaction mixture was stirred at 100 °C under nitrogen atmosphere for 12 h. After the consumption of the starting material, the mixture was diluted with 33 mL EtOAc, washed with 0.5 M HCl and saturated *aq.* NaCl. The organic layer was dried over Na_2SO_4 and removed under reduced pressure to give the residue; the residue was purified by silica gel chromatography (20% EtOAc/hexane) to give the compound **54** (206 mg, 40% yield).

Compound 54
Physical State: amorphous solid
^{1}H NMR (600 MHz, CDCl$_3$): δ 7.74 (d, J = 7.9 Hz, 1H), 7.70 (d, J = 8.5 Hz, 1H), 7.38 (d, J = 9.0 Hz, 1H), 7.34 (d, J = 7.2 Hz, 2H), 7.29 (d, J = 6.5 Hz, 6H), 7.21 (d, J = 6.1 Hz, 2H), 7.15 (t, J = 7.4 Hz, 1H), 6.08 (d, J = 7.5 Hz, 1H), 5.40 (d, J = 10.1 Hz, 1H), 5.17 (d, J = 8.8 Hz, 1H), 5.11 (s, 2H), 5.05 (d, J = 12.2 Hz, 1H), 4.95 (dq, J = 12.3, 3.7 Hz, 2H), 4.82 (dt, J = 11.6, 5.1 Hz, 2H), 4.28 (dd, J = 10.7, 5.4 Hz, 1H), 4.23 – 4.13 (m, 1H), 3.39 (t, J = 12.6 Hz, 1H), 3.17 (dd, J = 13.1, 3.7 Hz, 1H), 3.11 (dd, J = 17.8, 5.1 Hz, 1H), 2.81 (dt, J = 13.7, 7.0 Hz, 1H), 2.71 – 2.55 (m, 3H), 1.77 (ddd, J = 14.3, 9.9, 4.2 Hz, 1H), 1.63 – 1.55 (m, 1H), 1.46 (s, 9H), 1.36 (d, J = 10.6 Hz, 1H), 1.01 – 0.88 (m, 23H), 0.66 (q, J = 7.5, 6.7 Hz, 1H), 0.61 – 0.53 (m, 1H).
^{13}C NMR (151 MHz, CDCl$_3$): δ 174.52, 171.18, 171.04, 170.42, 170.37, 156.58, 155.54, 140.06, 136.73, 135.16, 133.99, 128.95, 128.68, 128.58, 128.52, 128.27, 128.23, 128.14, 127.83, 125.39, 122.50, 118.98, 114.09, 80.82, 67.19, 66.79, 58.25, 54.04, 51.54, 49.07, 40.59, 36.37, 35.53, 30.64, 28.41, 25.78, 24.45, 23.57, 23.14, 21.91, 7.79, 5.38.
HRMS (ESI-TOF): calculated for $C_{52}H_{70}N_6NaO_{10}Si^+$ [M+Na]$^+$: 989.4815, found: 989.4814.
TLC: R_f = 0.5 (3:1 Hexane:EtOAc, $Ce_2(SO_4)_3$ in phosphomolybdic acid).
[α]$^{25}_D$: –126.7 (c = 1.0, CHCl$_3$)

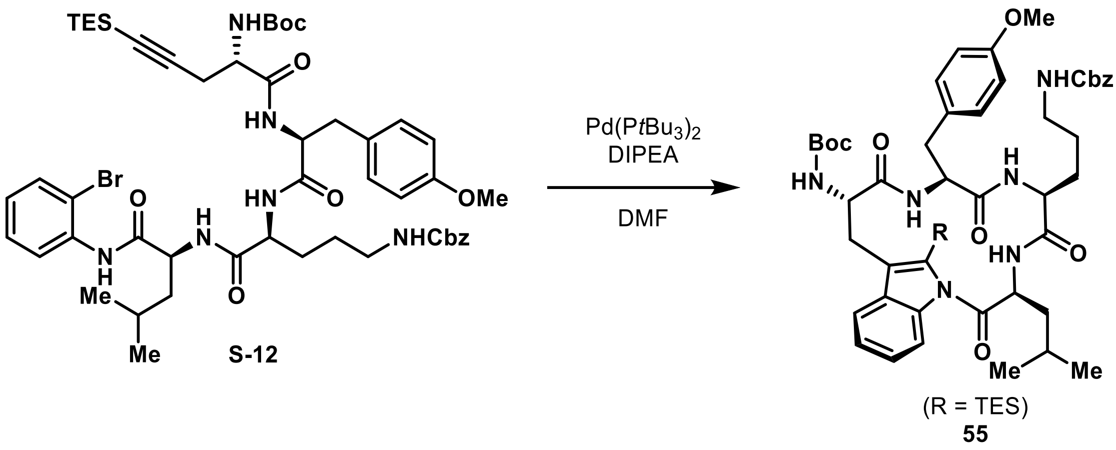

To a solution of compound **S-12** (596 mg, 0.585 mmol, 1.0 equiv.) in 12 mL anhydrous DMF was added DIPEA (0.3 mL, 1.76 mmol, 3.0 equiv.) and Pd(*t*$Bu_3P)_2$ (30 mg, 0.06 mmol, 0.1 equiv.). The reaction mixture was stirred at 100 °C under nitrogen atmosphere for 12 h. After the consumption of the starting material, the mixture was diluted with 36 mL EtOAc, washed with 0.5 M HCl and saturated *aq.* NaCl. The organic layer was dried over Na_2SO_4 and removed under reduced pressure to give the residue; the residue was purified by silica gel chromatography (20% EtOAc/hexane) to give the compound **55** (280 mg, 51% yield).

Compound 55

Physical State: amorphous solid

^{1}H NMR (600 MHz, $CDCl_3$): δ 7.68 (t, *J* = 6.9 Hz, 2H), 7.56 (dd, *J* = 8.8, 4.2 Hz, 1H), 7.48 (d, *J* = 7.6 Hz, 2H), 7.38 (t, *J* = 7.6 Hz, 2H), 7.35 – 7.29 (m, 1H), 7.18 – 7.09 (m, 1H), 7.09 – 6.99 (m, 3H), 6.67 (d, *J* = 8.0 Hz, 2H), 6.09 (d, *J* = 7.5 Hz, 1H), 5.65 (d, *J* = 9.8 Hz, 1H), 5.35 (d, *J* = 12.5 Hz, 1H), 5.24 (d, *J* = 12.5 Hz, 1H), 5.06 (d, *J* = 8.8 Hz, 1H), 4.99 – 4.83 (m, 1H), 4.75 (s, 1H), 4.68 (s, 1H), 4.32 (dd, *J* = 9.9, 5.4 Hz, 1H), 4.18 (ddd, *J* = 11.3, 7.4, 4.3 Hz, 1H), 3.67 (s, 3H), 3.34 (t, *J* = 12.5 Hz, 1H), 3.11 (dd, *J* = 13.1, 3.6 Hz, 1H), 2.99 – 2.84 (m, 2H), 2.54 (ddd, *J* = 14.6, 10.6, 4.4 Hz, 1H), 2.42 (s, 1H), 2.15 (s, 1H), 2.02 (d, *J* = 7.4 Hz, 1H), 1.76 (ddd, *J* = 14.2, 9.8, 4.3 Hz, 1H), 1.64 – 1.53 (m, 1H), 1.47 (s, 9H), 1.26 – 1.16 (m, 2H), 1.04 – 0.96 (m, 7H), 0.96 – 0.82 (m, 14H).

^{13}C NMR (151 MHz, $CDCl_3$): δ 174.42, 171.11, 170.99, 170.68, 158.71, 156.56, 155.38, 140.02, 136.78, 133.74, 130.50, 129.65, 128.62, 128.23, 128.14, 125.03, 122.02, 118.93, 114.00, 113.89, 80.42, 67.07, 58.08, 55.22, 54.51, 53.84, 50.91, 40.42, 36.42, 36.24, 30.52, 28.45, 24.51, 24.31, 23.84, 23.57, 21.99, 7.81, 5.43.

HRMS (ESI-TOF): calculated for $C_{51}H_{70}N_6NaO_9Si^+$ $[M+Na]^+$: 961.4866, found: 961.4918.

TLC: R_f = 0.6 (3:1 Hexane:EtOAc, $Ce_2(SO_4)_3$ in phosphomolybdic acid).

$[\alpha]^{25}_D$: –105.6 (*c* = 1.0, $CHCl_3$)

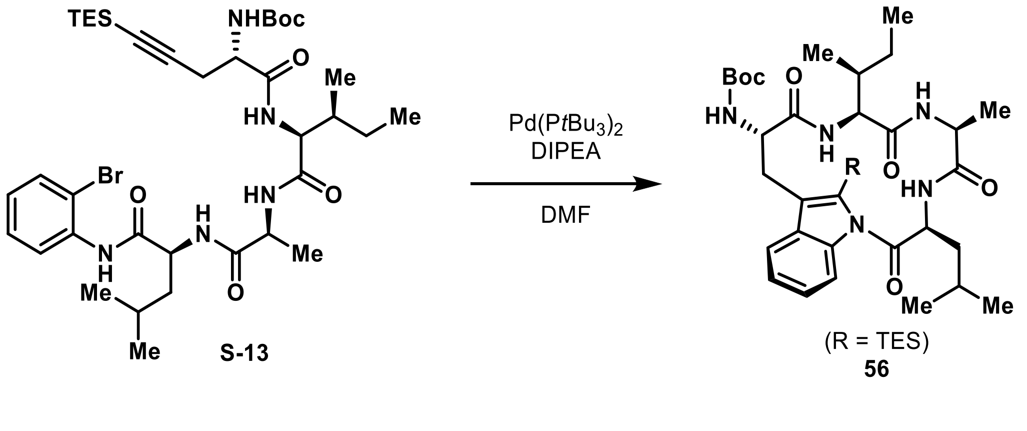

To a solution of compound **S-13** (571 mg, 0.734 mmol, 1.0 equiv.) in 15 mL anhydrous DMF was added DIPEA (0.38 mL, 2.2 mmol, 3.0 equiv.) and Pd(tBu$_3$P)$_2$ (35 mg, 0.07 mmol, 0.1 equiv.). The reaction mixture was stirred at 100 °C under nitrogen atmosphere for 12 h. After the consumption of the starting material, the mixture was diluted with 45 mL EtOAc, washed with 0.5 M HCl and saturated *aq.* NaCl. The organic layer was dried over Na_2SO_4 and removed under reduced pressure to give the residue; the residue was purified by silica gel chromatography (15% EtOAc/hexane) to give the compound **56** (200 mg, 39% yield).

Compound 56
Physical State: amorphous solid
^{1}H NMR (600 MHz, CDCl$_3$): δ 7.67 (d, J = 8.1 Hz, 2H), 7.26 (s, 1H), 7.19 (t, J = 7.5 Hz, 1H), 6.59 (d, J = 6.6 Hz, 1H), 6.11 (d, J = 7.3 Hz, 1H), 5.16 (d, J = 8.6 Hz, 1H), 5.04 – 4.88 (m, 2H), 4.35 – 4.23 (m, 1H), 4.19 – 4.03 (m, 2H), 3.36 (t, J = 12.7 Hz, 1H), 3.18 (dd, J = 13.2, 4.2 Hz, 1H), 2.59 (ddd, J = 14.9, 10.7, 4.5 Hz, 1H), 2.02 – 1.92 (m, 1H), 1.71 (d, J = 4.0 Hz, 1H), 1.64 – 1.57 (m, 1H), 1.48 (s, 9H), 1.15 (td, J = 8.1, 3.8 Hz, 1H), 0.96 (ddt, J = 21.4, 15.3, 6.9 Hz, 22H), 0.86 (d, J = 6.9 Hz, 3H), 0.81 (t, J = 7.3 Hz, 3H), 0.57 (d, J = 7.0 Hz, 3H).
^{13}C NMR (151 MHz, CDCl$_3$): δ 174.78, 171.75, 171.48, 170.36, 156.14, 140.28, 135.27, 128.57, 127.30, 124.97, 122.19, 118.38, 114.08, 81.26, 58.97, 53.04, 47.28, 36.24, 35.77, 29.37, 28.41, 24.46, 24.15, 23.52, 22.01, 16.71, 15.93, 11.70, 7.77, 5.16.
HRMS (ESI-TOF): calculated for $C_{37}H_{59}N_5NaO_6Si^+$ [M+Na]$^+$: 720.4127, found: 720.4133.
TLC: R$_f$ = 0.5 (3:1 Hexane:EtOAc, $Ce_2(SO_4)_3$ in phosphomolybdic acid).
[α]$^{25}_D$: –83.3 (c = 1.0, CHCl$_3$)

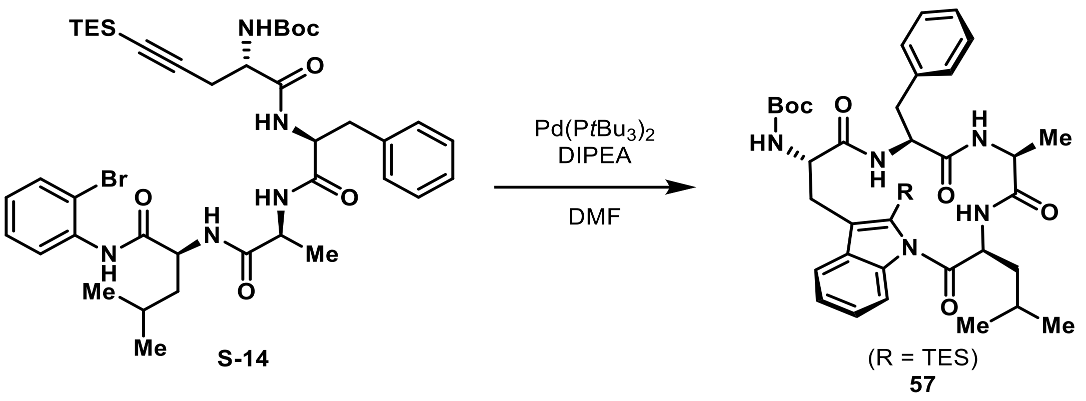

To a solution of compound **S-14** (665 mg, 0.820 mmol, 1.0 equiv.) in 17 mL anhydrous DMF was added DIPEA (0.43 mL, 2.46 mmol, 3.0 equiv.) and Pd(tBu$_3$P)$_2$ (40 mg, 0.08 mmol, 0.1 equiv.). The reaction mixture was stirred at 100 °C under nitrogen atmosphere for 12 h. After the consumption of the starting material, the mixture was diluted with 51 mL EtOAc, washed with 0.5 M HCl and saturated *aq.* NaCl. The organic layer was dried over Na_2SO_4 and removed under reduced pressure to give the residue; the residue was purified by silica gel chromatography (20% EtOAc/hexane) to give the compound **57** (240 mg, 40% yield).

Compound 57
Physical State: amorphous solid
^{1}H NMR (600 MHz, CDCl$_3$): δ 7.61 (d, J = 8.4 Hz, 1H), 7.57 (d, J = 7.9 Hz, 1H), 7.27 (s, 1H), 7.25 – 7.23 (m, 1H), 7.23 – 7.17 (m, 2H), 7.09 (dd, J = 14.3, 7.2 Hz, 3H), 6.60 (d, J = 6.8 Hz, 1H), 6.09 (d, J = 7.3 Hz, 1H), 5.11 (d, J = 8.5 Hz, 1H), 4.85 (ddd, J = 12.7, 8.5, 4.2 Hz, 1H), 4.80 (d, J = 9.5 Hz, 1H), 4.56 (q, J = 6.1 Hz, 1H), 4.27 – 4.17 (m, 1H), 4.10 (ddd, J = 11.4, 7.3, 4.3 Hz, 1H), 3.30 (t, J = 12.8 Hz, 1H), 3.13 (dd, J = 13.2, 4.3 Hz, 1H), 3.05 (dd, J = 14.2, 6.6 Hz, 1H), 2.88 (dd, J = 14.2, 4.9 Hz, 1H), 2.57 (ddd, J = 14.7, 10.6,

4.4 Hz, 1H), 1.72 (ddd, J = 14.2, 9.7, 4.3 Hz, 1H), 1.63 – 1.57 (m, 1H), 1.47 (s, 9H), 0.99 – 0.87 (m, 21H), 0.44 (d, J = 7.0 Hz, 3H).

^{13}C NMR (151 MHz, CDCl3): δ 174.74, 171.34, 171.11, 170.19, 155.84, 140.18, 135.31, 135.08, 129.25, 128.91, 128.59, 127.49, 127.29, 124.92, 122.12, 118.39, 113.96, 81.09, 58.88, 54.23, 53.04, 47.53, 36.97, 36.33, 29.87, 28.47, 24.48, 23.51, 22.01, 16.28, 7.75, 5.12.

HRMS (ESI-TOF): calculated for $C_{40}H_{57}N_5NaO_6Si^+$ [M+Na]$^+$: 754.3970, found: 754.3972.

TLC: R_f = 0.6 (3:1 Hexane:EtOAc, $Ce_2(SO_4)_3$ in phosphomolybdic acid).

[α]$^{25}_{D}$: –135.5 (c = 1.0, $CHCl_3$)

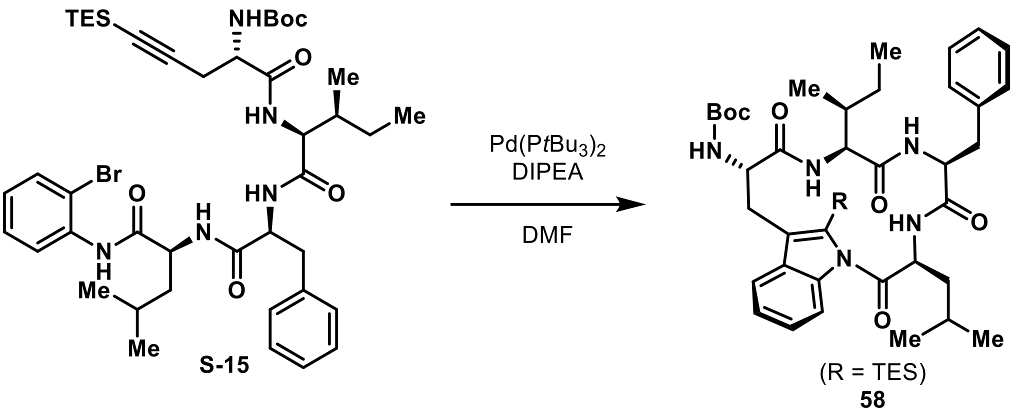

To a solution of compound **S-15** (418 mg, 0.49 mmol, 1.0 equiv.) in 10 mL anhydrous DMF was added DIPEA (0.26 mL, 1.5 mmol, 3.0 equiv.) and Pd(tBu3P)2 (25 mg, 0.05 mmol, 0.1 equiv.). The reaction mixture was stirred at 100 °C under nitrogen atmosphere for 12 h. After the consumption of the starting material, the mixture was diluted with 30 mL EtOAc, washed with 0.5 M HCl and saturated *aq.* NaCl. The organic layer was dried over Na_2SO_4 and removed under reduced pressure to give the residue; the residue was purified by silica gel chromatography (20% EtOAc/hexane) to give the compound **58** (216 mg, 57% yield).

Compound 58

Physical State: amorphous solid

^{1}H NMR (600 MHz, CDCl3): δ 7.78 – 7.71 (m, 2H), 7.34 (tt, J = 7.4, 5.7 Hz, 2H), 7.14 (td, J = 7.3, 6.3, 1.4 Hz, 2H), 7.11 – 7.06 (m, 1H), 6.77 – 6.71 (m, 2H), 6.51 (d, J = 6.3 Hz, 1H), 6.00 (d, J = 7.2 Hz, 1H), 5.11 (d, J = 8.6 Hz, 1H), 4.95 (ddd, J = 12.7, 8.5, 4.4 Hz, 1H), 4.78 (d, J = 9.5 Hz, 1H), 4.50 (ddd, J = 12.1, 9.5, 4.3 Hz, 1H), 4.11 (ddd, J = 11.4, 7.2, 4.5 Hz, 1H), 3.99 (dd, J = 6.2, 4.7 Hz, 1H), 3.34 (t, J = 12.7 Hz, 1H), 3.19 (ddd, J = 15.0, 12.4, 4.4 Hz, 2H), 2.71 (ddd, J = 14.8, 10.8, 4.4 Hz, 1H), 1.73 (dd, J = 14.2, 4.5 Hz, 1H), 1.64 (dddd, J = 9.6, 6.7, 4.8, 2.6 Hz, 2H), 1.50 (dt, J = 7.0, 3.9 Hz, 1H), 1.46 (s, 9H), 1.03 – 0.90 (m, 21H), 0.56 (d, J = 6.9 Hz, 3H), 0.49 (t, J = 7.0 Hz, 3H), 0.44 (dtd, J = 13.4, 7.3, 6.5, 3.6 Hz, 1H), 0.06 (ddd, J = 12.9, 7.3, 3.1 Hz, 1H).

^{13}C NMR (151 MHz, CDCl3): δ 174.35, 171.55, 171.01, 170.37, 156.23, 140.78, 137.07, 136.11, 129.09, 128.75, 128.60, 127.50, 126.70, 125.27, 122.32, 118.52, 114.03, 81.34, 59.51, 59.35, 52.64, 52.54, 36.64, 36.11, 34.98, 29.18, 28.41, 24.45, 23.52, 23.17, 22.13, 15.34, 11.66, 7.78, 5.13.

HRMS (ESI-TOF): calculated for $C_{43}H_{63}N_5NaO_6Si^+$ [M+Na]$^+$: 796.4440, found: 796.4441.

TLC: R_f = 0.6 (3:1 Hexane:EtOAc, $Ce_2(SO_4)_3$ in phosphomolybdic acid).

[α]$^{25}_{D}$: –46.0 (c = 1.0, $CHCl_3$)

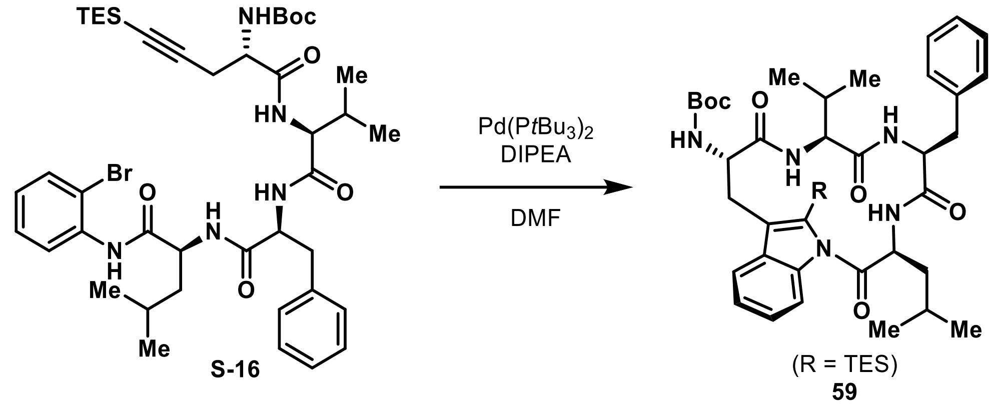

To a solution of compound **S-16** (597 mg, 0.711 mmol, 1.0 equiv.) in 14 mL anhydrous DMF was added DIPEA (0.37 mL, 2.1 mmol, 3.0 equiv.) and Pd(tBu$_3$P)$_2$ (35 mg, 0.07 mmol, 0.1 equiv.). The reaction mixture was stirred at 100 °C under nitrogen atmosphere for 12 h. After the consumption of the starting material, the mixture was diluted with 42 mL EtOAc, washed with 0.5 M HCl and saturated *aq.* NaCl. The organic layer was dried over Na_2SO_4 and removed under reduced pressure to give the residue; the residue was purified by silica gel chromatography (20% EtOAc/hexane) to give the compound **59** (330 mg, 61% yield).

Compound 59
Physical State: amorphous solid
^{1}H NMR (600 MHz, CDCl$_3$): δ 7.77 – 7.70 (m, 2H), 7.37 – 7.31 (m, 2H), 7.12 (dd, *J* = 8.2, 6.9 Hz, 2H), 7.08 – 7.03 (m, 1H), 6.78 – 6.70 (m, 2H), 6.57 (d, *J* = 6.2 Hz, 1H), 6.03 (d, *J* = 7.2 Hz, 1H), 5.13 (d, *J* = 8.5 Hz, 1H), 4.95 (ddd, *J* = 12.6, 8.4, 4.3 Hz, 1H), 4.81 (d, *J* = 9.4 Hz, 1H), 4.48 (ddd, *J* = 12.0, 9.4, 4.4 Hz, 1H), 4.12 (ddd, *J* = 11.4, 7.2, 4.4 Hz, 1H), 3.93 (dd, *J* = 6.2, 4.9 Hz, 1H), 3.35 (t, *J* = 12.8 Hz, 1H), 3.18 (ddd, *J* = 21.3, 14.0, 4.4 Hz, 2H), 2.70 (ddd, *J* = 14.7, 10.8, 4.4 Hz, 1H), 1.78 – 1.70 (m, 2H), 1.63 (dtd, *J* = 9.6, 6.6, 4.3 Hz, 1H), 1.46 (s, 9H), 1.04 – 0.91 (m, 21H), 0.60 (d, *J* = 7.0 Hz, 3H), 0.08 (d, *J* = 6.9 Hz, 3H).
^{13}C NMR (151 MHz, CDCl$_3$): δ 174.38, 171.55, 170.95, 170.38, 156.25, 140.76, 137.14, 136.06, 129.10, 128.71, 128.69, 127.54, 126.71, 125.25, 122.32, 118.52, 114.05, 81.34, 59.57, 59.43, 52.67, 52.59, 36.57, 36.14, 29.19, 28.63, 28.42, 24.48, 23.52, 22.10, 19.14, 16.23, 7.78, 5.13.
HRMS (ESI-TOF): calculated for $C_{42}H_{61}N_5NaO_6Si^+$ [M+Na]$^+$: 782.4283, found: 782.4280.
TLC: R$_f$ = 0.4 (4:1 Hexane:EtOAc, $Ce_2(SO_4)_3$ in phosphomolybdic acid).
[α]$^{25}_{D}$: –83.7 (*c* = 1.0, $CHCl_3$)

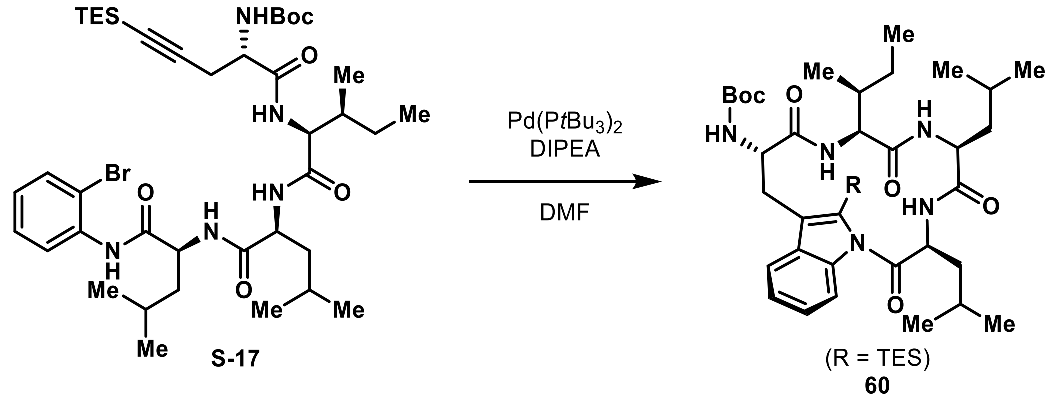

To a solution of compound **S-17** (730 mg, 0.890 mmol, 1.0 equiv.) in 18 mL anhydrous DMF was added DIPEA (0.46 mL, 2.67 mmol, 3.0 equiv.) and Pd(tBu$_3$P)$_2$ (45 mg, 0.09 mmol, 0.1 equiv.). The reaction mixture was stirred at 100 °C under nitrogen atmosphere for 12 h. After the consumption of the starting material, the

mixture was diluted with 54 mL EtOAc, washed with 0.5 M HCl and saturated *aq.* NaCl. The organic layer was dried over Na_2SO_4 and removed under reduced pressure to give the residue; the residue was purified by silica gel chromatography (15% EtOAc/hexane) to give the compound **60** (270 mg, 41% yield).

Compound 60
Physical State: amorphous solid
^{1}H NMR (600 MHz, $CDCl_3$): δ 7.69 (d, *J* = 8.3 Hz, 1H), 7.66 (d, *J* = 7.8 Hz, 1H), 7.26 (d, *J* = 7.8 Hz, 1H), 7.21 (t, *J* = 7.4 Hz, 1H), 6.76 (d, *J* = 6.5 Hz, 1H), 6.06 (d, *J* = 7.2 Hz, 1H), 5.21 (d, *J* = 8.2 Hz, 1H), 4.94 (ddd, *J* = 17.2, 8.5, 4.0 Hz, 2H), 4.16 (dtd, *J* = 22.5, 11.2, 10.6, 4.2 Hz, 3H), 3.38 (t, *J* = 12.7 Hz, 1H), 3.20 (dd, *J* = 13.0, 4.2 Hz, 1H), 2.64 (ddd, *J* = 14.6, 10.5, 4.4 Hz, 1H), 1.94 – 1.88 (m, 1H), 1.74 (ddd, *J* = 14.1, 9.5, 4.5 Hz, 1H), 1.63 (td, *J* = 13.5, 12.8, 5.2 Hz, 1H), 1.50 (s, 9H), 1.40 (ddd, *J* = 14.5, 10.6, 3.9 Hz, 1H), 1.24 (ddt, *J* = 15.5, 7.7, 3.9 Hz, 1H), 1.11 – 1.00 (m, 5H), 0.95 (tq, *J* = 13.6, 7.0 Hz, 18H), 0.88 (d, *J* = 6.9 Hz, 3H), 0.84 (t, *J* = 7.3 Hz, 3H), 0.70 (d, *J* = 6.6 Hz, 3H), 0.66 (d, *J* = 6.5 Hz, 3H), 0.06 (ddd, *J* = 14.7, 10.9, 3.9 Hz, 1H).
^{13}C NMR (151 MHz, $CDCl_3$): δ 174.48, 171.55, 171.51, 170.43, 156.31, 140.55, 135.79, 128.96, 127.39, 125.15, 122.22, 118.35, 113.93, 81.34, 59.21, 59.13, 52.83, 50.32, 39.28, 36.33, 35.61, 29.12, 28.42, 24.55, 24.48, 24.45, 23.49, 23.30, 22.12, 20.77, 15.75, 11.56, 7.77, 5.16.
HRMS (ESI-TOF): calculated for $C_{40}H_{65}N_5NaO_6Si^+$ $[M+Na]^+$: 762.4596, found: 762.4601.
TLC: R_f = 0.5 (3:1 Hexane:EtOAc, $Ce_2(SO_4)_3$ in phosphomolybdic acid).
$[\alpha]^{25}_D$: –99.5 (*c* = 1.0, $CHCl_3$)

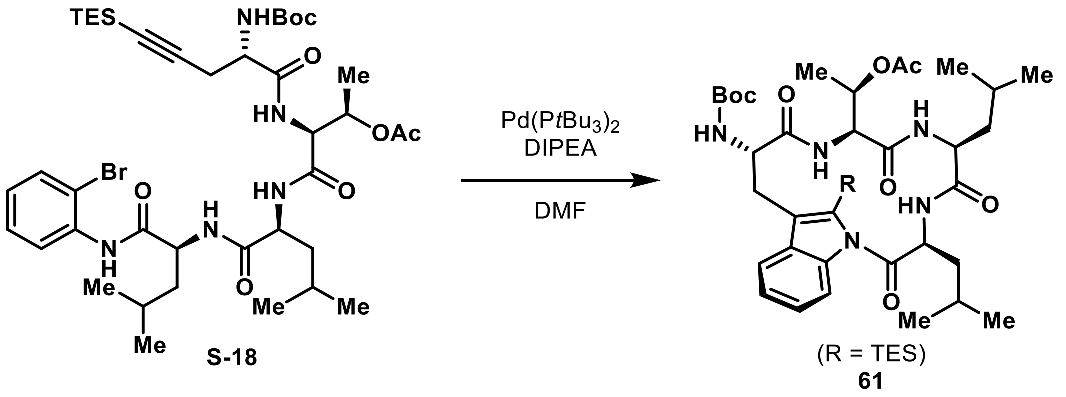

To a solution of compound **S-18** (589 mg, 0.693 mmol, 1.0 equiv.) in 14 mL anhydrous DMF was added DIPEA (0.37 mL, 2.1 mmol, 3.0 equiv.) and Pd(tBu$_3$P)$_2$ (35 mg, 0.07 mmol, 0.1 equiv.). The reaction mixture was stirred at 100 °C under nitrogen atmosphere for 12 h. After the consumption of the starting material, the mixture was diluted with 42 mL EtOAc, washed with 0.5 M HCl and saturated *aq.* NaCl. The organic layer was dried over Na_2SO_4 and removed under reduced pressure to give the residue; the residue was purified by silica gel chromatography (25% EtOAc/hexane) to give the compound **61** (203 mg, 38% yield).

Compound 61
Physical State: amorphous solid
^{1}H NMR (600 MHz, $CDCl_3$): δ 7.70 (d, *J* = 7.8 Hz, 1H), 7.65 (d, *J* = 8.3 Hz, 1H), 7.28 – 7.26 (m, 1H), 7.23 – 7.18 (m, 1H), 6.78 (d, *J* = 7.6 Hz, 1H), 5.87 (d, *J* = 7.3 Hz, 1H), 5.40 (qd, *J* = 6.4, 2.5 Hz, 1H), 5.20 (d, *J* = 8.3 Hz, 1H), 4.98 (ddd, *J* = 12.5, 8.3, 4.2 Hz, 1H), 4.79 (d, *J* = 10.0 Hz, 1H), 4.43 (dd, *J* = 7.6, 2.5 Hz, 1H), 4.27 – 4.16 (m, 1H), 4.09 (ddd, *J* = 11.3, 7.3, 4.5 Hz, 1H), 3.39 (t, *J* = 12.7 Hz, 1H), 3.20 (dd, *J* = 13.1, 4.2 Hz, 1H), 2.63 (ddd, *J* = 14.7, 10.7, 4.3 Hz, 1H), 1.90 (s, 3H), 1.72 (ddd, *J* = 14.3, 9.8, 4.4 Hz, 1H), 1.59 (dtd, *J* = 11.9, 6.2, 5.8, 2.9 Hz, 1H), 1.47 (s, 9H), 1.38 (ddd, *J* = 14.5, 10.8, 3.7 Hz, 1H), 1.21 (d, *J* = 6.5 Hz, 3H),

1.02 – 0.88 (m, 21H), 0.87 – 0.82 (m, 1H), 0.64 (d, J = 6.7 Hz, 3H), 0.60 (d, J = 6.5 Hz, 3H), -0.10 (ddd, J = 14.7, 11.3, 3.5 Hz, 1H).

^{13}C NMR (151 MHz, CDCl$_3$): δ 174.45, 171.72, 171.29, 169.18, 168.14, 156.06, 140.33, 135.24, 128.28, 127.33, 125.32, 122.30, 118.54, 114.06, 81.34, 69.57, 59.13, 57.63, 53.33, 50.16, 39.55, 36.30, 29.57, 28.40, 24.37, 24.17, 23.51, 23.39, 22.01, 20.84, 20.78, 16.65, 7.76, 5.18.

HRMS (ESI-TOF): calculated for $C_{40}H_{63}N_5NaO_8Si^+$ [M+Na]$^+$: 792.4338, found: 792.4339.

TLC: R$_f$ = 0.4 (3:1 Hexane:EtOAc, $Ce_2(SO_4)_3$ in phosphomolybdic acid).

[α]$^{25}_D$: –78.6 (c = 1.0, CHCl$_3$)

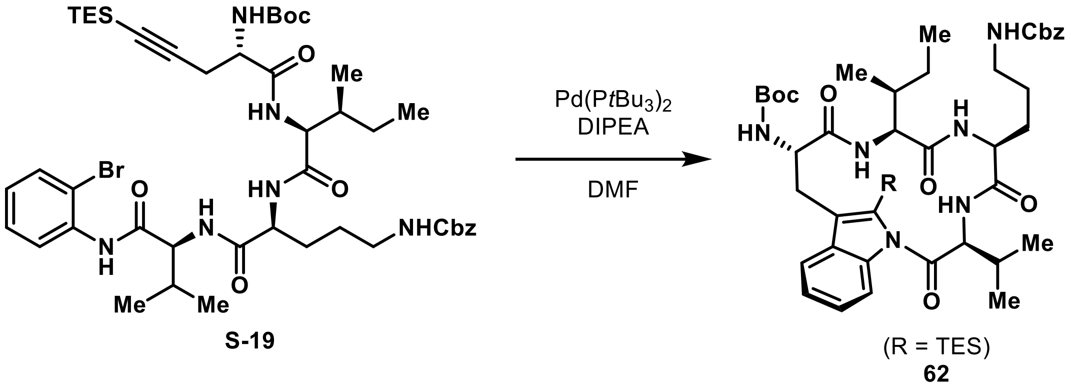

To a solution of compound **S-19** (543 mg, 0.535 mmol, 1.0 equiv.) in 11 mL anhydrous DMF was added DIPEA (0.28 mL, 1.6 mmol, 3.0 equiv.) and Pd(tBu$_3$P)$_2$ (25 mg, 0.05 mmol, 0.1 equiv.). The reaction mixture was stirred at 100 °C under nitrogen atmosphere for 12 h. After the consumption of the starting material, the mixture was diluted with 33 mL EtOAc, washed with 0.5 M HCl and saturated *aq.* NaCl. The organic layer was dried over Na_2SO_4 and removed under reduced pressure to give the residue; the residue was purified by silica gel chromatography (30% EtOAc/hexane) to give the compound **62** (220 mg, 44% yield).

Compound 62

Physical State: amorphous solid

^{1}H NMR (600 MHz, CDCl$_3$): δ 7.78 (d, J = 8.3 Hz, 1H), 7.63 (d, J = 7.8 Hz, 1H), 7.41 (d, J = 7.5 Hz, 2H), 7.38 – 7.32 (m, 2H), 7.32 – 7.29 (m, 1H), 7.18 (ddd, J = 8.4, 7.0, 1.2 Hz, 1H), 7.11 (t, J = 7.4 Hz, 1H), 6.23 (d, J = 7.6 Hz, 1H), 5.91 (d, J = 9.5 Hz, 1H), 5.25 (d, J = 12.4 Hz, 1H), 5.18 (t, J = 9.8 Hz, 2H), 4.90 (ddd, J = 12.4, 8.9, 3.6 Hz, 1H), 4.78 (t, J = 6.1 Hz, 1H), 4.49 – 4.23 (m, 2H), 3.80 (dd, J = 9.5, 7.6 Hz, 1H), 3.42 (t, J = 12.6 Hz, 1H), 3.17 – 3.03 (m, 2H), 2.74 – 2.56 (m, 1H), 2.31 (s, 1H), 1.96 – 1.85 (m, 1H), 1.46 (s, 9H), 1.39 – 1.32 (m, 1H), 1.21 (d, J = 6.7 Hz, 3H), 1.19 – 1.13 (m, 1H), 1.06 – 1.00 (m, 3H), 0.96 – 0.92 (m, 11H), 0.89 (d, J = 6.8 Hz, 3H), 0.84 (d, J = 6.9 Hz, 3H), 0.78 (t, J = 7.4 Hz, 3H).

^{13}C NMR (151 MHz, CDCl$_3$): δ 172.99, 171.64, 171.46, 170.73, 156.74, 155.68, 140.42, 136.70, 134.34, 129.77, 128.59, 128.19, 128.10, 125.05, 122.16, 118.62, 114.25, 80.66, 67.04, 64.89, 58.57, 53.75, 50.70, 40.29, 36.18, 29.71, 28.40, 27.21, 24.70, 24.47, 21.07, 19.72, 15.65, 11.59, 7.81, 5.70.

HRMS (ESI-TOF): calculated for $C_{46}H_{68}N_6NaO_8Si^+$ [M+Na]$^+$: 883.4760, found: 883.4756.

TLC: R$_f$ = 0.4 (2:1 Hexane:EtOAc, $Ce_2(SO_4)_3$ in phosphomolybdic acid).

[α]$^{25}_D$: –68.5 (c = 1.0, CHCl$_3$)

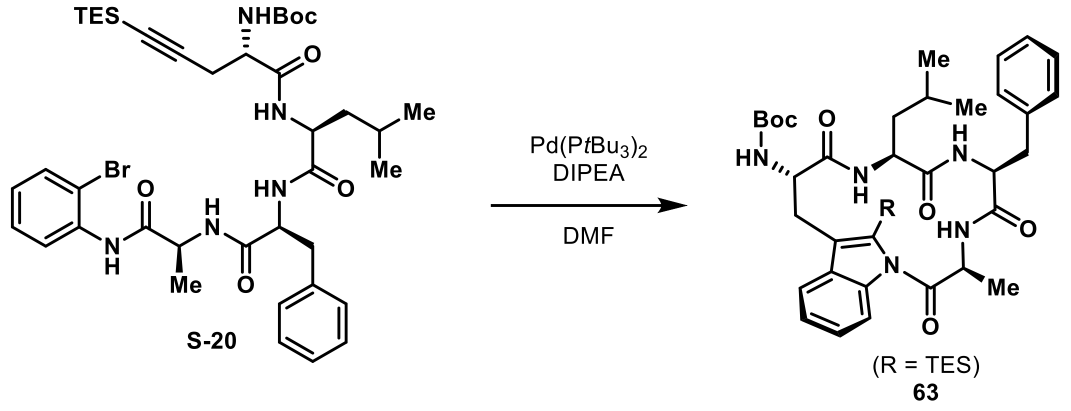

To a solution of compound **S-20** (678 mg, 0.835 mmol, 1.0 equiv.) in 17 mL anhydrous DMF was added DIPEA (0.43 mL, 2.5 mmol, 3.0 equiv.) and Pd(tBu$_3$P)$_2$ (40 mg, 0.08 mmol, 0.1 equiv.). The reaction mixture was stirred at 100 °C under nitrogen atmosphere for 12 h. After the consumption of the starting material, the mixture was diluted with 50 mL EtOAc, washed with 0.5 M HCl and saturated *aq.* NaCl. The organic layer was dried over Na_2SO_4 and removed under reduced pressure to give the residue; the residue was purified by silica gel chromatography (30% EtOAc/hexane) to give the compound **63** (318 mg, 52% yield).

Compound 63

Physical State: amorphous solid

^{1}H NMR (600 MHz, CDCl$_3$): δ 7.76 (dd, J = 14.7, 7.9 Hz, 2H), 7.35 (dt, J = 22.5, 7.3 Hz, 2H), 7.15 (t, J = 7.4 Hz, 2H), 7.09 (t, J = 7.4 Hz, 1H), 6.72 (d, J = 7.3 Hz, 2H), 6.28 (d, J = 4.9 Hz, 1H), 6.12 (d, J = 7.2 Hz, 1H), 5.12 (d, J = 8.6 Hz, 1H), 5.00 (ddd, J = 12.9, 8.6, 4.6 Hz, 1H), 4.67 (d, J = 9.7 Hz, 1H), 4.43 (ddd, J = 11.9, 9.6, 4.7 Hz, 1H), 4.23 (p, J = 6.9 Hz, 1H), 3.86 (dt, J = 11.3, 4.5 Hz, 1H), 3.31 (t, J = 12.8 Hz, 1H), 3.18 (td, J = 14.1, 13.4, 4.6 Hz, 2H), 1.74 (d, J = 6.8 Hz, 3H), 1.45 (s, 9H), 1.30 (dd, J = 14.7, 12.0 Hz, 1H), 1.24 – 1.19 (m, 1H), 0.94 (d, J = 3.5 Hz, 15H), 0.68 (d, J = 6.6 Hz, 3H), 0.64 (d, J = 6.5 Hz, 3H), 0.53 (p, J = 8.3 Hz, 1H), 0.35 (ddd, J = 15.0, 11.3, 4.5 Hz, 1H).

^{13}C NMR (151 MHz, CDCl$_3$): δ 174.25, 171.59, 171.26, 171.02, 156.10, 140.68, 137.13, 136.01, 129.14, 128.81, 128.56, 127.48, 126.67, 125.30, 122.42, 118.68, 113.98, 81.28, 56.39, 52.81, 52.66, 52.44, 39.25, 36.49, 29.44, 28.47, 28.32, 24.25, 22.97, 20.69, 13.94, 7.74, 4.98.

HRMS (ESI-TOF): calculated for $C_{40}H_{57}N_5NaO_6Si^+$ [M+Na]$^+$: 754.3970, found: 754.3976.

TLC: R_f = 0.6 (2:1 Hexane:EtOAc, $Ce_2(SO_4)_3$ in phosphomolybdic acid).

[α]$^{25}_{D}$: –79.7 (c = 1.0, CHCl$_3$)

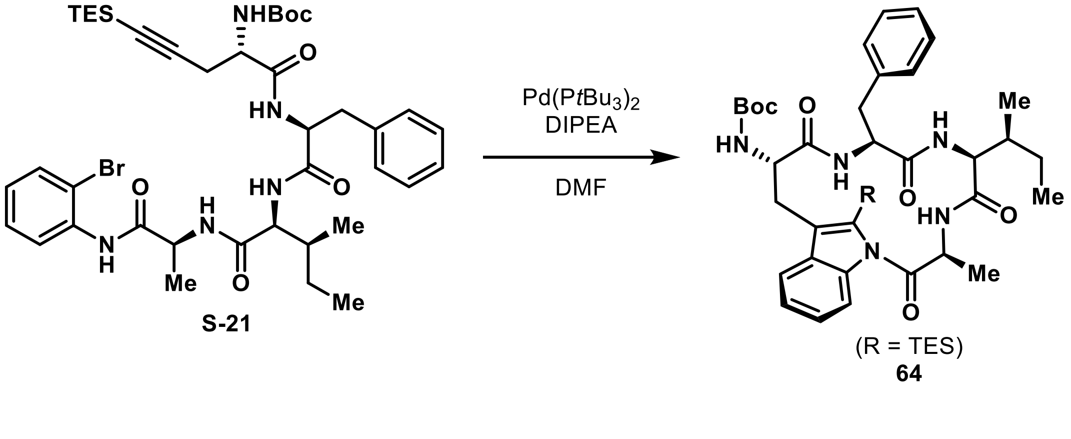

To a solution of compound **S-21** (667 mg, 0.822 mmol, 1.0 equiv.) in 17 mL anhydrous DMF was added DIPEA (0.43 mL, 2.5 mmol, 3.0 equiv.) and Pd(tBu$_3$P)$_2$ (40 mg, 0.08 mmol, 0.1 equiv.). The reaction mixture

was stirred at 100 °C under nitrogen atmosphere for 12 h. After the consumption of the starting material, the mixture was diluted with 50 mL EtOAc, washed with 0.5 M HCl and saturated *aq.* NaCl. The organic layer was dried over Na_2SO_4 and removed under reduced pressure to give the residue; the residue was purified by silica gel chromatography (30% EtOAc/hexane) to give the compound **64** (331 mg, 55% yield).

Compound 64

Physical State: amorphous solid

^{1}H NMR (600 MHz, $CDCl_3$): δ 7.91 (d, *J* = 8.3 Hz, 1H), 7.64 (d, *J* = 7.8 Hz, 1H), 7.30 (t, *J* = 7.7 Hz, 1H), 7.24 – 7.20 (m, 3H), 7.18 (t, *J* = 7.3 Hz, 1H), 7.13 – 7.10 (m, 2H), 6.63 (s, 1H), 6.30 (d, *J* = 56.8 Hz, 1H), 5.56 (d, *J* = 9.4 Hz, 1H), 5.22 (d, *J* = 8.3 Hz, 1H), 4.84 (ddd, *J* = 12.4, 8.3, 4.3 Hz, 1H), 4.54 (q, *J* = 6.6 Hz, 2H), 3.77 (s, 1H), 3.28 (t, *J* = 12.6 Hz, 1H), 3.19 (dd, *J* = 13.2, 4.3 Hz, 1H), 2.85 (qd, *J* = 14.1, 6.6 Hz, 2H), 1.90 (dtd, *J* = 9.7, 6.5, 2.8 Hz, 1H), 1.66 (d, *J* = 6.6 Hz, 3H), 1.46 (s, 9H), 0.98 – 0.83 (m, 15H), 0.51 – 0.41 (m, 6H), -0.08 (s, 1H), -0.18 (q, *J* = 11.4, 9.7 Hz, 1H).

^{13}C NMR (151 MHz, $CDCl_3$): δ 173.67, 170.73, 170.60, 170.28, 155.80, 140.46, 135.47, 135.37, 130.37, 129.16, 128.91, 128.05, 127.39, 125.80, 123.01, 118.53, 114.42, 81.03, 54.83, 54.74, 52.85, 37.20, 33.37, 30.48, 28.50, 22.90, 15.26, 14.37, 11.39, 7.77, 5.31.

HRMS (ESI-TOF): calculated for $C_{40}H_{57}N_5NaO_6Si^+$ $[M+Na]^+$: 754.3970, found: 754.3961.

TLC: R_f = 0.6 (2:1 Hexane:EtOAc, $Ce_2(SO_4)_3$ in phosphomolybdic acid).

$[\alpha]^{25}_D$: –59.1 (*c* = 1.0, $CHCl_3$)

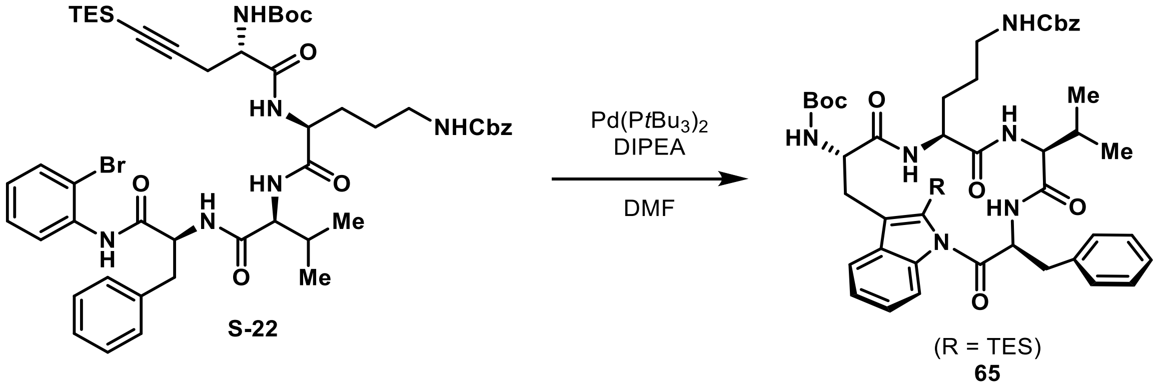

To a solution of compound **S-22** (617 mg, 0.633 mmol, 1.0 equiv.) in 13 mL anhydrous DMF was added DIPEA (0.33 mL, 1.9 mmol, 3.0 equiv.) and Pd(*t*$Bu_3P)_2$ (30 mg, 0.06 mmol, 0.1 equiv.). The reaction mixture was stirred at 100 °C under nitrogen atmosphere for 12 h. After the consumption of the starting material, the mixture was diluted with 40 mL EtOAc, washed with 0.5 M HCl and saturated *aq.* NaCl. The organic layer was dried over Na_2SO_4 and removed under reduced pressure to give the residue; the residue was purified by silica gel chromatography (40% EtOAc/hexane) to give the compound **65** (255 mg, 45% yield).

Compound 65

Physical State: amorphous solid

^{1}H NMR (600 MHz, $CDCl_3$): δ 7.86 (d, *J* = 8.3 Hz, 1H), 7.65 (d, *J* = 7.9 Hz, 1H), 7.35 – 7.26 (m, 10H), 7.22 (p, *J* = 4.3 Hz, 1H), 7.18 (t, *J* = 7.5 Hz, 1H), 6.99 – 6.67 (m, 1H), 6.47 – 6.09 (m, 1H), 5.81 – 5.40 (m, 1H), 5.23 (d, *J* = 8.3 Hz, 1H), 5.04 (s, 2H), 4.94 (t, *J* = 6.0 Hz, 1H), 4.85 (ddd, *J* = 12.6, 8.4, 3.7 Hz, 1H), 4.51 – 4.35 (m, 1H), 4.25 – 4.16 (m, 1H), 4.05 – 3.90 (m, 1H), 3.77 (dd, *J* = 13.9, 6.9 Hz, 1H), 3.39 (dd, *J* = 13.9, 6.6 Hz, 1H), 3.31 (t, *J* = 12.7 Hz, 1H), 3.22 – 3.06 (m, 3H), 2.22 (q, *J* = 6.6, 6.2 Hz, 1H), 1.82 (s, 1H), 1.54 (ddd, *J* = 15.6, 8.2, 3.6 Hz, 1H), 1.45 (s, 9H), 0.98 – 0.93 (m, 1H), 0.79 (t, *J* = 3.4 Hz, 12H), 0.75 – 0.66 (m, 3H), 0.60 – 0.55 (m, 3H), -0.03 – -0.13 (m, 3H).

^{13}C NMR (151 MHz, CDCl$_3$): δ 172.87, 171.41, 171.23, 170.38, 156.79, 156.04, 140.66, 137.91, 136.46, 135.20, 129.94, 129.84, 128.77, 128.65, 128.29, 128.17, 127.96, 127.01, 125.79, 122.76, 118.60, 114.10, 81.14, 66.86, 61.37, 53.90, 53.04, 40.00, 35.04, 29.65, 28.84, 28.41, 27.44, 26.57, 19.38, 15.48, 7.72, 7.58, 5.14.
HRMS (ESI-TOF): calculated for $C_{49}H_{66}N_6NaO_8Si^+$ $[M+Na]^+$: 917.4604, found: 917.4612.
TLC: R_f = 0.6 (1:1 Hexane:EtOAc, $Ce_2(SO_4)_3$ in phosphomolybdic acid).
$[\alpha]^{25}_D$: –98.7 (*c* = 1.0, $CHCl_3$)

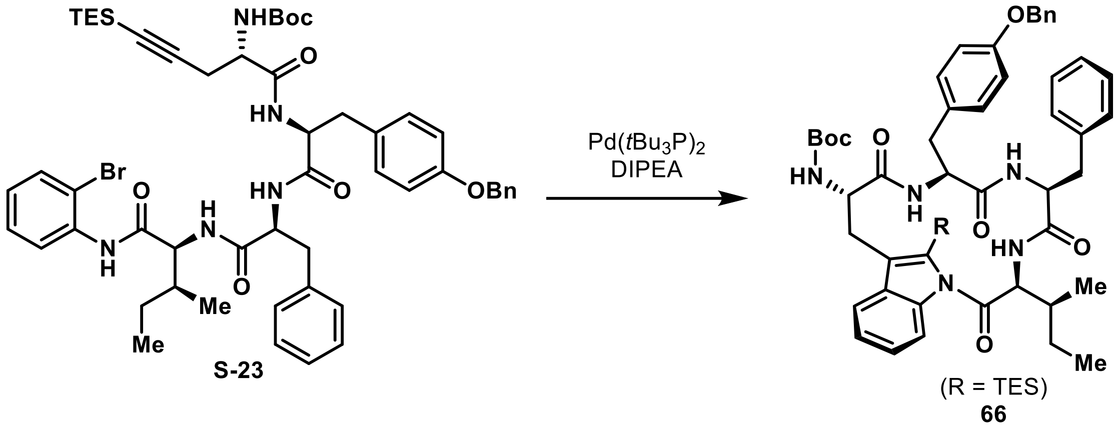

To a solution of compound **S-23** (4.24 g, 4.27 mmol, 1.0 equiv.) in 250 mL anhydrous DMF was added DIPEA (2.3 mL, 12.8 mmol, 3.0 equiv.) and Pd(tBu$_3$P)$_2$ (220 mg, 0.43 mmol, 0.1 equiv.). The reaction mixture was stirred at 100 °C under nitrogen atmosphere for 6 h. When complete, the mixture was diluted with 400 mL EtOAc, washed by 0.5 M HCl and saturated *aq.* NaCl. The organic layer was dried over Na_2SO_4 and removed under reduced pressure to give the residue; the residue was purified by silica gel chromatography (20% EtOAc/hexane) to give the compound **66** (1.6 g, 41% yield).

Compound 66
Physical State: amorphous solid
^{1}H NMR (600 MHz, CDCl$_3$): δ 7.77 – 7.71 (m, 1H), 7.64 (d, *J* = 8.0 Hz, 1H), 7.42 – 7.34 (m, 6H), 7.33 – 7.29 (m, 2H), 7.26 – 7.21 (m, 2H), 7.21 – 7.17 (m, 1H), 7.06 – 6.98 (m, 2H), 6.87 – 6.80 (m, 2H), 6.79 – 6.73 (m, 2H), 6.29 (d, *J* = 8.2 Hz, 1H), 5.80 (d, *J* = 9.4 Hz, 1H), 5.62 (d, *J* = 7.8 Hz, 1H), 5.56 (t, *J* = 9.2 Hz, 1H), 5.21 (d, *J* = 8.7 Hz, 1H), 4.97 (d, *J* = 2.3 Hz, 2H), 4.54 – 4.39 (m, 2H), 4.13 (q, *J* = 7.8 Hz, 1H), 3.49 – 3.34 (m, 1H), 3.07 (dd, *J* = 13.3, 3.9 Hz, 1H), 2.82 (dd, *J* = 13.7, 8.2 Hz, 1H), 2.72 (ddd, *J* = 18.4, 13.9, 6.6 Hz, 2H), 2.56 (dd, *J* = 14.2, 6.5 Hz, 1H), 2.06 (tddd, *J* = 8.9, 6.5, 4.4, 2.3 Hz, 1H), 1.61 (ddt, *J* = 14.3, 6.9, 3.3 Hz, 1H), 1.48 (s, 9H), 1.01 (t, *J* = 7.3 Hz, 3H), 0.92 – 0.84 (m, 15H), 0.74 (d, *J* = 6.7 Hz, 3H).
^{13}C NMR (151 MHz, CDCl$_3$): δ 172.40, 170.68, 170.23, 170.18, 157.91, 155.45, 139.99, 138.35, 137.00, 136.94, 132.38, 130.39, 129.91, 129.33, 128.78, 128.67, 128.19, 128.08, 127.61, 126.99, 124.93, 123.53, 119.71, 115.53, 115.03, 80.39, 77.34, 77.13, 76.92, 69.99, 57.61, 55.69, 54.63, 54.51, 37.49, 36.63, 36.24, 29.36, 28.50, 27.18, 14.56, 12.08, 8.22, 5.85.
HRMS (ESI-TOF): calculated for $C_{53}H_{67}N_5NaO_7Si^+$ $[M+Na]^+$: 936.4702, found: 936.4709.
TLC: R_f = 0.5 (4:1 Hexane:EtOAc, $Ce_2(SO_4)_3$ in phosphomolybdic acid).
$[\alpha]^{25}_D$: –59.6 (*c* = 1.0, $CHCl_3$)

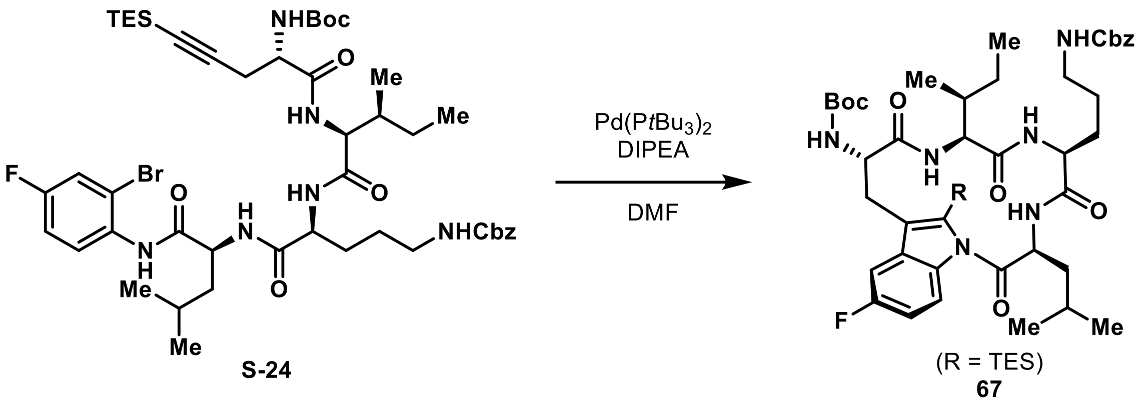

To a solution of compound **S-24** (512 mg, 0.524 mmol, 1.0 equiv.) in 11 mL anhydrous DMF was added DIPEA (0.26 mL, 1.5 mmol, 3.0 equiv.) and Pd(tBu$_3$P)$_2$ (25 mg, 0.05 mmol, 0.1 equiv.). The reaction mixture was stirred at 100 °C under nitrogen atmosphere for 12 h. After the consumption of the starting material, the mixture was diluted with 33 mL EtOAc, washed with 0.5 M HCl and saturated *aq.* NaCl. The organic layer was dried over Na_2SO_4 and removed under reduced pressure to give the residue; the residue was purified by silica gel chromatography (20% EtOAc/hexane) to give the compound **67** (170 mg, 37% yield).

Compound 67
Physical State: amorphous solid
^{1}H NMR (400 MHz, CDCl$_3$): δ 7.71 (dd, J = 9.1, 4.5 Hz, 1H), 7.54 (d, J = 9.2 Hz, 1H), 7.47 – 7.30 (m, 6H), 6.94 (td, J = 9.0, 2.5 Hz, 1H), 6.42 (d, J = 7.4 Hz, 1H), 6.34 (d, J = 9.3 Hz, 1H), 5.40 (d, J = 12.5 Hz, 1H), 5.25 – 5.13 (m, 2H), 4.93 (t, J = 6.1 Hz, 1H), 4.83 (ddd, J = 12.4, 9.1, 3.4 Hz, 1H), 4.47 (t, J = 8.1 Hz, 2H), 4.22 (ddd, J = 11.1, 7.3, 4.1 Hz, 1H), 3.40 (t, J = 12.6 Hz, 1H), 3.08 (dd, J = 13.0, 3.4 Hz, 1H), 2.64 – 2.38 (m, 2H), 2.11 – 1.96 (m, 1H), 1.79 (ddd, J = 14.2, 9.8, 4.2 Hz, 2H), 1.70 – 1.49 (m, 3H), 1.45 (s, 9H), 1.42 – 1.35 (m, 1H), 1.30 – 1.25 (m, 1H), 1.10 – 1.02 (m, 5H), 1.02 – 0.99 (m, 3H), 0.99 – 0.89 (m, 15H), 0.86 (d, J = 6.9 Hz, 3H), 0.79 (t, J = 7.3 Hz, 3H).
^{13}C NMR (151 MHz, CDCl$_3$): δ 174.57, 172.36, 171.40, 171.17, 158.06, 157.19, 155.42, 136.61, 136.24, 135.55, 128.87, 128.51, 128.14, 115.20, 115.14, 112.91, 112.74, 103.92, 103.77, 80.43, 67.40, 58.59, 57.62, 54.19, 50.10, 40.11, 36.68, 36.39, 30.00, 28.35, 24.62, 24.59, 23.60, 23.05, 21.93, 15.47, 11.38, 7.82, 5.50.
^{19}F NMR (565 MHz, CDCl$_3$): δ -120.86.
HRMS (ESI-TOF): calculated for $C_{47}H_{69}FN_6NaO_8Si^+$ [M+Na]$^+$: 915.4822, found: 915.4821.
TLC: R$_f$ = 0.5 (3:1 Hexane:EtOAc, $Ce_2(SO_4)_3$ in phosphomolybdic acid).
[α]$^{25}_D$: –110.4 (c = 1.0, CHCl$_3$)

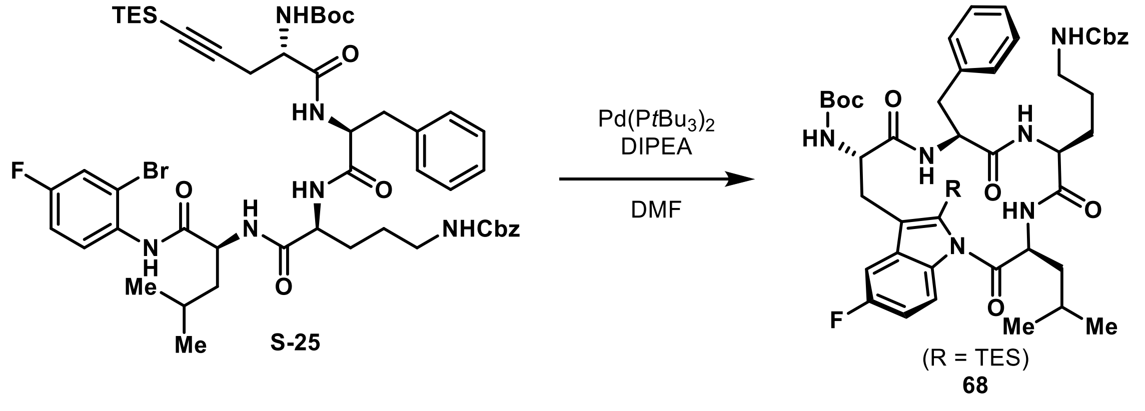

To a solution of compound **S-25** (612 mg, 0.608 mmol, 1.0 equiv.) in 12 mL anhydrous DMF was added DIPEA (0.33 mL, 1.9 mmol, 3.0 equiv.) and Pd(tBu$_3$P)$_2$ (30 mg, 0.06 mmol, 0.1 equiv.). The reaction mixture was stirred at 100 °C under nitrogen atmosphere for 12 h. After the consumption of the starting material, the mixture was diluted with 36 mL EtOAc, washed with 0.5 M HCl and saturated *aq.* NaCl. The organic layer was dried over Na_2SO_4 and removed under reduced pressure to give the residue; the residue was purified by silica gel chromatography (20% EtOAc/hexane) to give the compound **68** (220 mg, 39% yield).

Compound 68
Physical State: amorphous solid
^{1}H NMR (600 MHz, CDCl$_3$): δ 7.74 (d, J = 9.1 Hz, 1H), 7.65 (dd, J = 9.1, 4.5 Hz, 1H), 7.48 (d, J = 7.5 Hz, 2H), 7.43 – 7.36 (m, 3H), 7.34 – 7.29 (m, 1H), 7.11 (dd, J = 8.3, 4.3 Hz, 5H), 6.90 (td, J = 9.1, 2.5 Hz, 1H), 6.16 (d, J = 7.5 Hz, 1H), 5.94 (d, J = 9.7 Hz, 1H), 5.41 (d, J = 12.5 Hz, 1H), 5.22 (d, J = 12.4 Hz, 1H), 5.00 (d, J = 8.9 Hz, 1H), 4.92 – 4.76 (m, 3H), 4.39 (p, J = 4.4 Hz, 1H), 4.18 (ddd, J = 11.2, 7.5, 4.2 Hz, 1H), 3.32 (t, J = 12.6 Hz, 1H), 3.08 – 2.98 (m, 2H), 2.93 (dd, J = 13.9, 8.1 Hz, 1H), 2.48 (ddd, J = 14.6, 10.6, 4.3 Hz, 1H), 2.38 (q, J = 8.9, 8.1 Hz, 1H), 2.05 – 1.96 (m, 1H), 1.77 (ddd, J = 14.2, 9.8, 4.2 Hz, 1H), 1.63 – 1.56 (m, 1H), 1.44 (s, 9H), 1.42 – 1.39 (m, 1H), 1.26 – 1.22 (m, 1H), 1.02 – 0.97 (m, 6H), 0.92 (t, J = 7.2 Hz, 12H), 0.89 – 0.83 (m, 3H), 0.80 – 0.70 (m, 1H), 0.58 (ddt, J = 17.2, 11.7, 5.7 Hz, 1H).
^{13}C NMR (151 MHz, CDCl$_3$): δ 174.37, 171.34, 170.89, 170.81, 159.58, 158.00, 156.93, 155.18, 136.65, 136.25, 136.02, 135.43, 129.52, 129.24, 128.92, 128.55, 128.18, 127.07, 115.02, 114.96, 113.10, 112.93, 104.26, 104.10, 80.29, 67.39, 57.77, 54.34, 53.92, 50.59, 40.33, 37.51, 36.30, 30.52, 28.45, 24.52, 24.37, 23.59, 22.99, 21.94, 7.80, 5.45.
^{19}F NMR (565 MHz, CDCl$_3$): δ -120.75.
HRMS (ESI-TOF): calculated for $C_{50}H_{67}FN_6NaO_8Si^+$ [M+Na]$^+$: 949.4666, found: 949.4670.
TLC: R$_f$ = 0.5 (3:1 Hexane:EtOAc, $Ce_2(SO_4)_3$ in phosphomolybdic acid).
[α]$^{25}_D$: –74.1 (c = 1.0, CHCl$_3$)

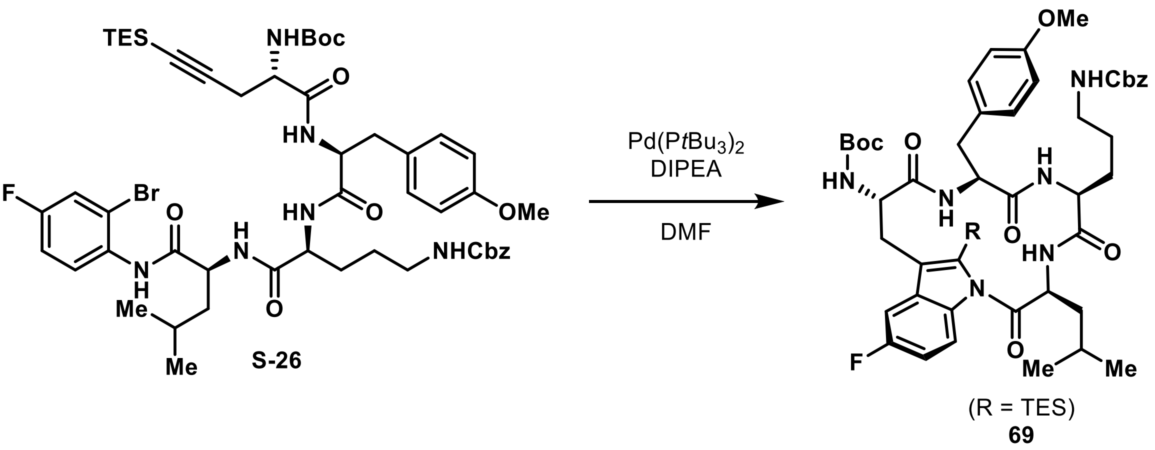

(R = TES)
69

To a solution of compound **S-26** (575 mg, 0.553 mmol, 1.0 equiv.) in 11 mL anhydrous DMF was added DIPEA (0.28 mL, 1.6 mmol, 3.0 equiv.) and Pd(tBu$_3$P)$_2$ (25 mg, 0.05 mmol, 0.1 equiv.). The reaction mixture was stirred at 100 °C under nitrogen atmosphere for 12 h. After the consumption of the starting material, the mixture was diluted with 33 mL EtOAc, washed with 0.5 M HCl and saturated *aq.* NaCl. The organic layer was dried over Na_2SO_4 and removed under reduced pressure to give the residue; the residue was purified by silica gel chromatography (15% EtOAc/hexane) to give the compound **69** (180 mg, 34% yield).

Compound 69
Physical State: amorphous solid

^{1}H NMR (400 MHz, CDCl$_3$): ^{1}H NMR (400 MHz, Chloroform-*d*) δ 7.73 (d, *J* = 9.1 Hz, 1H), 7.67 (dd, *J* = 9.1, 4.5 Hz, 1H), 7.51 (d, *J* = 7.4 Hz, 2H), 7.45 – 7.37 (m, 3H), 7.36 – 7.31 (m, 1H), 7.07 – 6.99 (m, 2H), 6.93 (td, *J* = 9.0, 2.5 Hz, 1H), 6.65 (d, *J* = 8.1 Hz, 2H), 6.17 (d, *J* = 7.5 Hz, 1H), 5.94 (d, *J* = 9.7 Hz, 1H), 5.46 (d, *J* = 12.5 Hz, 1H), 5.23 (d, *J* = 12.4 Hz, 1H), 5.07 (d, *J* = 8.9 Hz, 1H), 4.98 – 4.77 (m, 3H), 4.46 – 4.33 (m, 1H), 4.20 (ddd, *J* = 11.3, 7.4, 4.2 Hz, 1H), 3.66 (s, 3H), 3.35 (t, *J* = 12.5 Hz, 1H), 3.07 (dd, *J* = 13.1, 3.4 Hz, 1H), 2.98 – 2.85 (m, 2H), 2.50 (ddd, *J* = 14.6, 10.6, 4.3 Hz, 1H), 2.42 – 2.28 (m, 1H), 2.10 – 1.96 (m, 1H), 1.79 (ddd, *J* = 14.2, 9.8, 4.2 Hz, 1H), 1.65 – 1.56 (m, 1H), 1.47 (s, 9H), 1.45 – 1.40 (m, 1H), 1.29 – 1.22 (m, 1H), 1.03 – 0.87 (m, 21H), 0.81 – 0.69 (m, 1H), 0.58 (td, *J* = 14.2, 12.1, 5.8 Hz, 1H).
^{13}C NMR (101 MHz, CDCl$_3$): δ 173.66, 170.64, 170.18, 170.09, 159.24, 157.97, 156.87, 156.15, 154.50, 135.99, 135.52, 129.83, 127.82, 127.53, 127.46, 127.15, 114.30, 114.21, 113.22, 112.43, 112.17, 103.60, 103.37, 79.62, 66.62, 57.03, 54.49, 53.79, 53.22, 49.83, 39.59, 35.95, 35.58, 29.78, 27.71, 23.80, 23.64, 22.88, 22.27, 22.25, 21.23, 7.09, 4.74.
^{19}F NMR (565 MHz, CDCl$_3$): δ -120.82.
HRMS (ESI-TOF): calculated for $C_{51}H_{69}FN_6NaO_9Si^+$ [M+Na]$^+$: 979.4772, found: 979.4770.
TLC: R$_f$ = 0.5 (3:1 Hexane:EtOAc, $Ce_2(SO_4)_3$ in phosphomolybdic acid).
[α]$^{25}_{D}$: –92.6 (*c* = 1.0, CHCl$_3$)

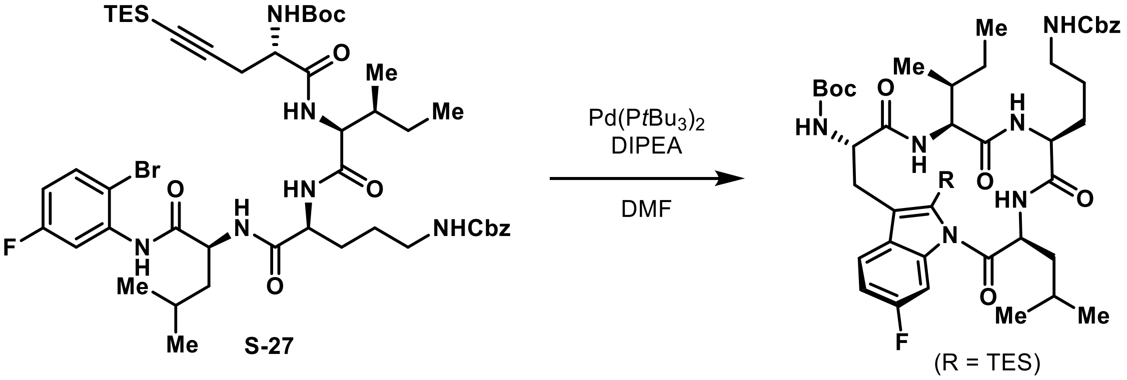

S-27
(R = TES)
70

To a solution of compound **S-27** (502 mg, 0.515 mmol, 1.0 equiv.) in 11 mL anhydrous DMF was added DIPEA (0.26 mL, 1.5 mmol, 3.0 equiv.) and Pd(*t*Bu$_3$P)$_2$ (25 mg, 0.05 mmol, 0.1 equiv.). The reaction mixture was stirred at 100 °C under nitrogen atmosphere for 12 h. After the consumption of the starting material, the mixture was diluted with 33 mL EtOAc, washed with 0.5 M HCl and saturated *aq.* NaCl. The organic layer was dried over Na_2SO_4 and removed under reduced pressure to give the residue; the residue was purified by silica gel chromatography (20% EtOAc/hexane) to give the compound **70** (230 mg, 50% yield).

Compound 70
Physical State: amorphous solid
^{1}H NMR (400 MHz, CDCl$_3$): δ 7.59 (dd, *J* = 8.7, 5.2 Hz, 1H), 7.54 – 7.41 (m, 4H), 7.41 – 7.31 (m, 4H), 6.83 (td, *J* = 8.9, 2.3 Hz, 1H), 6.43 (d, *J* = 7.4 Hz, 1H), 6.26 (d, *J* = 9.5 Hz, 1H), 5.35 – 5.17 (m, 3H), 4.96 (t, *J* = 6.0 Hz, 1H), 4.86 (ddd, *J* = 12.4, 9.1, 3.4 Hz, 1H), 4.47 (dd, *J* = 9.3, 5.9 Hz, 2H), 4.22 (ddd, *J* = 11.2, 7.4, 4.1 Hz, 1H), 3.39 (t, *J* = 12.5 Hz, 1H), 3.11 (dd, *J* = 13.0, 3.4 Hz, 1H), 2.62 (q, *J* = 7.2 Hz, 1H), 2.50 (ddd, *J* = 14.6, 10.7, 4.2 Hz, 1H), 2.10 – 1.99 (m, 1H), 1.87 – 1.73 (m, 2H), 1.66 – 1.58 (m, 2H), 1.47 (s, 9H), 1.39 – 1.32 (m, 1H), 1.27 – 1.23 (m, 1H), 1.06 – 1.02 (m, 2H), 1.01 – 0.99 (m, 3H), 0.97 – 0.92 (m, 15H), 0.85 (d, *J* = 6.9 Hz, 3H), 0.78 (t, *J* = 7.3 Hz, 3H), 0.64 – 0.49 (m, 3H).
^{13}C NMR (151 MHz, CDCl$_3$): δ 174.52, 172.20, 171.34, 171.21, 162.14, 160.54, 156.97, 155.49, 136.62, 129.29, 128.60, 128.21, 127.99, 119.56, 110.70, 110.53, 101.10, 100.92, 80.45, 67.18, 58.56, 57.52, 54.34,

49.94, 40.14, 36.68, 36.30, 30.16, 28.37, 24.56, 24.32, 23.60, 23.24, 21.92, 15.47, 11.44, 7.80, 6.88, 6.68, 6.49, 5.88, 5.48.

^{19}F NMR (565 MHz, CDCl$_3$): δ -117.25.

HRMS (ESI-TOF): calculated for $C_{47}H_{69}FN_6NaO_8Si^+$ $[M+Na]^+$: 915.4822, found: 915.4826.

TLC: R_f = 0.5 (3:1 Hexane:EtOAc, $Ce_2(SO_4)_3$ in phosphomolybdic acid).

$[\alpha]^{25}_D$: –125.8 (*c* = 1.0, $CHCl_3$)

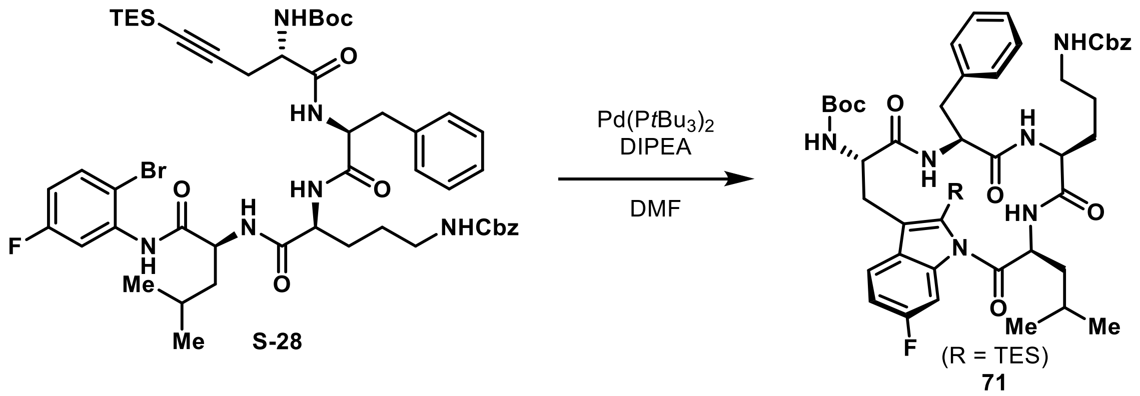

To a solution of compound **S-28** (780 mg, 0.773 mmol, 1.0 equiv.) in 15 mL anhydrous DMF was added DIPEA (0.38 mL, 2.2 mmol, 3.0 equiv.) and Pd(*t*Bu$_3$P)$_2$ (35 mg, 0.07 mmol, 0.1 equiv.). The reaction mixture was stirred at 100 °C under nitrogen atmosphere for 12 h. After the consumption of the starting material, the mixture was diluted with 45 mL EtOAc, washed with 0.5 M HCl and saturated *aq.* NaCl. The organic layer was dried over Na_2SO_4 and removed under reduced pressure to give the residue; the residue was purified by silica gel chromatography (20% EtOAc/hexane) to give the compound **71** (380 mg, 53% yield).

Compound 71

Physical State: amorphous solid

^{1}H NMR (400 MHz, CDCl$_3$): δ 7.69 (d, *J* = 9.1 Hz, 1H), 7.61 (dd, *J* = 8.7, 5.3 Hz, 1H), 7.52 (d, *J* = 7.6 Hz, 2H), 7.46 – 7.38 (m, 3H), 7.38 – 7.33 (m, 1H), 7.14 (q, *J* = 3.9, 3.2 Hz, 5H), 6.78 (td, *J* = 8.8, 2.3 Hz, 1H), 6.24 (d, *J* = 7.4 Hz, 1H), 5.95 (d, *J* = 9.7 Hz, 1H), 5.33 (q, *J* = 12.6 Hz, 2H), 5.06 (d, *J* = 8.9 Hz, 1H), 4.88 (t, *J* = 7.3 Hz, 3H), 4.44 (tt, *J* = 6.3, 3.0 Hz, 1H), 4.21 (ddd, *J* = 11.4, 7.5, 4.2 Hz, 1H), 3.33 (t, *J* = 12.5 Hz, 1H), 3.13 – 2.90 (m, 3H), 2.58 – 2.38 (m, 2H), 2.00 (q, *J* = 13.7, 12.6 Hz, 1H), 1.77 (ddd, *J* = 14.2, 9.8, 4.1 Hz, 1H), 1.62 – 1.54 (m, 2H), 1.48 (s, 9H), 1.27 – 1.21 (m, 1H), 1.01 – 0.84 (m, 21H), 0.79 – 0.71 (m, 1H), 0.59 – 0.49 (m, 1H).

^{13}C NMR (101 MHz, CDCl$_3$): δ 173.62, 170.54, 170.15, 170.03, 161.90, 159.49, 155.96, 154.50, 139.48, 139.36, 135.96, 135.31, 133.09, 128.90, 128.78, 127.92, 127.83, 127.53, 127.28, 126.34, 123.96, 119.18, 119.08, 110.01, 109.77, 100.28, 100.00, 79.56, 66.42, 56.88, 53.59, 53.44, 49.73, 39.63, 36.81, 35.51, 29.95, 27.76, 23.77, 23.31, 22.89, 22.20, 21.21, 7.09, 4.74.

^{19}F NMR (565 MHz, CDCl$_3$): δ -116.98.

HRMS (ESI-TOF): calculated for $C_{50}H_{67}FN_6NaO_8Si^+$ $[M+Na]^+$: 949.4666, found: 949.4669.

TLC: R_f = 0.5 (3:1 Hexane:EtOAc, $Ce_2(SO_4)_3$ in phosphomolybdic acid).

$[\alpha]^{25}_D$: –115.1 (*c* = 1.0, $CHCl_3$)

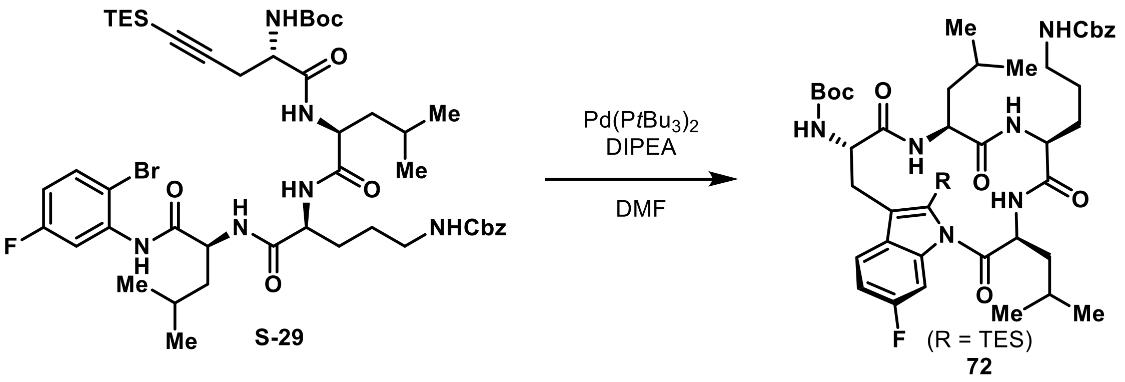

To a solution of compound **S-29** (631 mg, 0.646 mmol, 1.0 equiv.) in 13 mL anhydrous DMF was added DIPEA (0.33 mL, 1.9 mmol, 3.0 equiv.) and Pd(tBu$_3$P)$_2$ (30 mg, 0.06 mmol, 0.1 equiv.). The reaction mixture was stirred at 100 °C under nitrogen atmosphere for 12 h. After the consumption of the starting material, the mixture was diluted with 39 mL EtOAc, washed with 0.5 M HCl and saturated *aq.* NaCl. The organic layer was dried over Na_2SO_4 and removed under reduced pressure to give the residue; the residue was purified by silica gel chromatography (20% EtOAc/hexane) to give the compound **72** (330 mg, 57% yield).

Compound 72
Physical State: amorphous solid
^{1}H NMR (400 MHz, CDCl$_3$): δ 7.61 – 7.30 (m, 8H), 6.82 (td, J = 8.9, 2.3 Hz, 1H), 6.26 (d, J = 7.4 Hz, 1H), 5.99 (d, J = 9.7 Hz, 1H), 5.33 (d, J = 12.5 Hz, 1H), 5.27 – 5.15 (m, 2H), 5.03 – 4.82 (m, 2H), 4.51 (t, J = 9.4 Hz, 1H), 4.40 (dt, J = 10.0, 5.1 Hz, 1H), 4.19 (ddd, J = 11.1, 7.3, 4.2 Hz, 1H), 3.39 (t, J = 12.6 Hz, 1H), 3.10 (dd, J = 13.0, 3.6 Hz, 1H), 2.70 (dd, J = 15.1, 8.1 Hz, 1H), 2.53 (ddd, J = 14.6, 10.7, 4.2 Hz, 1H), 2.33 – 2.24 (m, 1H), 1.76 (ddd, J = 14.2, 9.8, 4.2 Hz, 1H), 1.62 (dtt, J = 13.7, 9.7, 5.1 Hz, 3H), 1.46 (s, 9H), 1.42 – 1.31 (m, 3H), 1.08 – 1.03 (m, 2H), 1.02 – 0.91 (m, 18H), 0.87 (td, J = 6.5, 3.9 Hz, 9H).
^{13}C NMR (101 MHz, CDCl$_3$): δ 173.79, 172.13, 170.38, 170.23, 161.82, 159.42, 156.21, 154.71, 135.88, 128.45, 127.90, 127.54, 127.43, 123.80, 118.89, 118.80, 110.04, 109.80, 100.35, 100.08, 79.82, 66.44, 57.16, 53.25, 51.73, 49.71, 39.91, 39.76, 35.46, 29.32, 27.61, 24.18, 23.79, 23.72, 23.01, 22.87, 22.46, 21.21, 20.55, 7.10, 4.69.
^{19}F NMR (565 MHz, CDCl$_3$): δ -117.00.
HRMS (ESI-TOF): calculated for $C_{47}H_{69}FN_6NaO_8Si^+$ [M+Na]$^+$: 915.4822, found: 915.4828.
TLC: R_f = 0.5 (3:1 Hexane:EtOAc, $Ce_2(SO_4)_3$ in phosphomolybdic acid).
[α]$^{25}_D$: –85.5 (c = 1.0, CHCl$_3$)

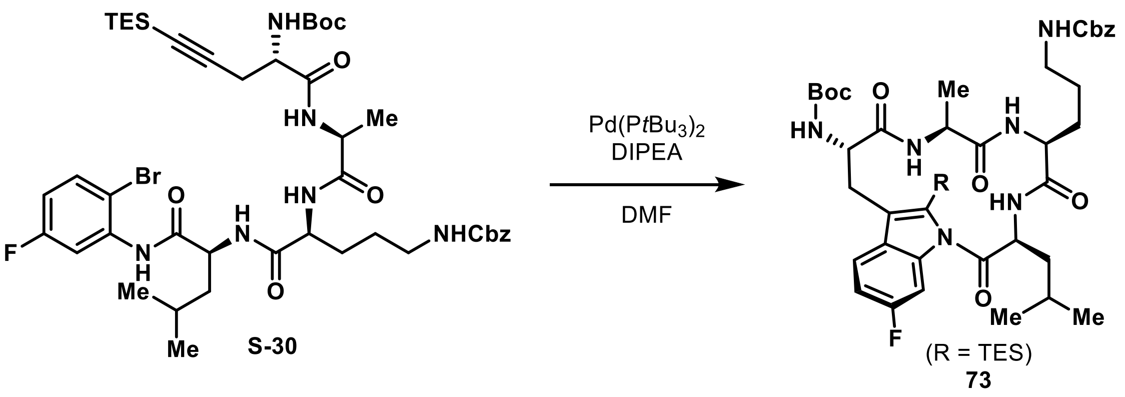

To a solution of compound **S-30** (400 mg, 0.427 mmol, 1.0 equiv.) in 9 mL anhydrous DMF was added DIPEA (0.26 mL, 1.5 mmol, 3.0 equiv.) and Pd(tBu$_3$P)$_2$ (20 mg, 0.04 mmol, 0.1 equiv.). The reaction mixture was

stirred at 100 °C under nitrogen atmosphere for 12 h. After the consumption of the starting material, the mixture was diluted with 27 mL EtOAc, washed with 0.5 M HCl and saturated *aq.* NaCl. The organic layer was dried over Na_2SO_4 and removed under reduced pressure to give the residue; the residue was purified by silica gel chromatography (30% EtOAc/hexane) to give the compound **73** (189 mg, 52% yield).

Compound 73

Physical State: amorphous solid

^{1}H NMR (400 MHz, CDCl$_3$): δ 7.65 (dd, *J* = 8.6, 5.1 Hz, 2H), 7.49 – 7.42 (m, 3H), 7.40 – 7.32 (m, 3H), 6.87 – 6.77 (m, 1H), 6.30 (d, *J* = 7.4 Hz, 1H), 5.92 (d, *J* = 9.7 Hz, 1H), 5.31 – 5.17 (m, 3H), 5.03 – 4.88 (m, 2H), 4.53 (t, *J* = 7.7 Hz, 1H), 4.42 – 4.31 (m, 1H), 4.19 (ddd, *J* = 11.2, 7.4, 4.2 Hz, 1H), 3.37 (t, *J* = 12.6 Hz, 1H), 3.12 (dd, *J* = 13.1, 3.5 Hz, 1H), 2.78 – 2.63 (m, 1H), 2.52 (ddd, *J* = 14.6, 10.6, 4.2 Hz, 1H), 2.38 – 2.27 (m, 1H), 1.77 (ddd, *J* = 14.2, 9.8, 4.2 Hz, 1H), 1.65 – 1.58 (m, 1H), 1.49 (s, 9H), 1.41 – 1.33 (m, 2H), 1.25 (d, *J* = 7.3 Hz, 3H), 1.05 – 1.02 (m, 1H), 1.00 – 0.87 (m, 21H), 0.84 – 0.76 (m, 1H).

^{13}C NMR (101 MHz, CDCl$_3$): δ 173.77, 172.06, 170.22, 169.75, 161.87, 159.47, 156.22, 154.77, 139.64, 139.51, 135.84, 128.71, 127.92, 127.55, 127.36, 123.81, 119.14, 119.04, 110.07, 109.83, 100.34, 100.06, 79.93, 66.50, 57.13, 53.28, 49.94, 48.62, 39.81, 35.56, 29.85, 27.69, 23.77, 23.14, 22.87, 21.21, 17.14, 7.07, 4.64.

^{19}F NMR (565 MHz, CDCl$_3$): δ -116.90.

HRMS (ESI-TOF): calculated for $C_{44}H_{63}FN_6NaO_8Si^+$ [M+Na]$^+$: 873.4353, found: 873.4346.

TLC: R$_f$ = 0.5 (2:1 Hexane:EtOAc, $Ce_2(SO_4)_3$ in phosphomolybdic acid).

[α]$^{25}_D$: –105.7 (*c* = 1.0, CHCl$_3$)

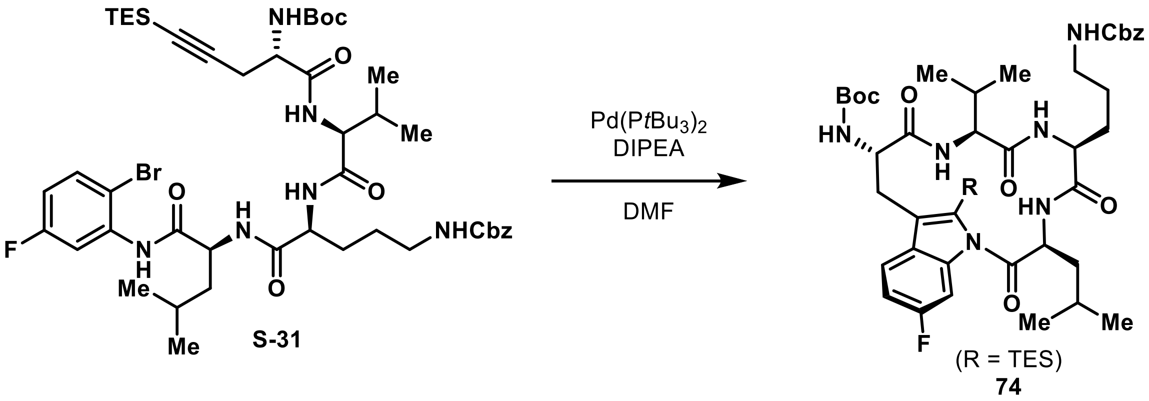

S-31 → 74

To a solution of compound **S-31** (397 mg, 0.413 mmol, 1.0 equiv.) in 9 mL anhydrous DMF was added DIPEA (0.23 mL, 1.3 mmol, 3.0 equiv.) and Pd(*t*Bu$_3$P)$_2$ (20 mg, 0.04 mmol, 0.1 equiv.). The reaction mixture was stirred at 100 °C under nitrogen atmosphere for 12 h. After the consumption of the starting material, the mixture was diluted with 27 mL EtOAc, washed with 0.5 M HCl and saturated *aq.* NaCl. The organic layer was dried over Na_2SO_4 and removed under reduced pressure to give the residue; the residue was purified by silica gel chromatography (20% EtOAc/hexane) to give the compound **74** (160 mg, 44% yield).

Compound 74

Physical State: amorphous solid

^{1}H NMR (400 MHz, CDCl$_3$): δ 7.58 (dt, *J* = 11.3, 5.9 Hz, 2H), 7.50 – 7.42 (m, 3H), 7.41 – 7.30 (m, 3H), 6.82 (td, *J* = 8.9, 2.4 Hz, 1H), 6.36 (d, *J* = 7.4 Hz, 1H), 6.19 (d, *J* = 9.6 Hz, 1H), 5.36 – 5.19 (m, 3H), 5.04 – 4.78 (m, 2H), 4.58 – 4.35 (m, 2H), 4.22 (ddd, *J* = 11.3, 7.4, 4.2 Hz, 1H), 3.40 (t, *J* = 12.5 Hz, 1H), 3.11 (dd, *J* = 13.0, 3.4 Hz, 1H), 2.72 – 2.39 (m, 2H), 2.16 (d, *J* = 8.9 Hz, 1H), 2.07 – 1.93 (m, 1H), 1.78 (ddd, *J* = 14.2, 9.8,

4.1 Hz, 1H), 1.65 – 1.56 (m, 2H), 1.46 (s, 9H), 1.39 – 1.30 (m, 1H), 1.06 – 1.02 (m, 2H), 1.01 – 0.99 (m, 3H), 0.99 – 0.91 (m, 15H), 0.91 – 0.83 (m, 6H), 0.73 (d, *J* = 6.8 Hz, 3H).
^{13}C NMR (101 MHz, $CDCl_3$): δ 173.76, 171.33, 170.65, 170.52, 161.84, 159.44, 156.20, 154.73, 139.47, 139.34, 135.93, 133.20, 128.65, 127.89, 127.48, 127.26, 124.11, 118.96, 118.86, 110.04, 109.79, 100.38, 100.11, 79.73, 66.47, 58.30, 56.79, 53.58, 49.24, 39.43, 35.56, 29.48, 29.37, 27.67, 23.82, 23.58, 22.89, 22.49, 21.19, 18.45, 16.70, 7.10, 4.77.
^{19}F NMR (565 MHz, $CDCl_3$): δ -117.18.
HRMS (ESI-TOF): calculated for $C_{46}H_{67}FN_6NaO_8Si^+$ $[M+Na]^+$: 901.4666, found: 901.4667.
TLC: R_f = 0.35 (3:1 Hexane:EtOAc, $Ce_2(SO_4)_3$ in phosphomolybdic acid).
$[\alpha]^{25}_D$: –91.2 (*c* = 1.0, $CHCl_3$)

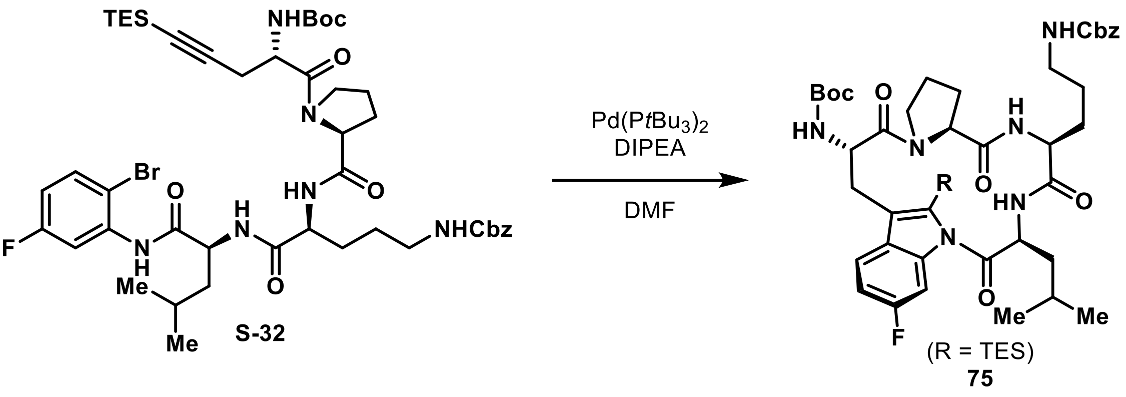

To a solution of compound **S-32** (442 mg, 0.461 mmol, 1.0 equiv.) in 9 mL anhydrous DMF was added DIPEA (0.25 mL, 1.4 mmol, 3.0 equiv.) and $Pd(tBu_3P)_2$ (25 mg, 0.05 mmol, 0.1 equiv.). The reaction mixture was stirred at 100 °C under nitrogen atmosphere for 12 h. After the consumption of the starting material, the mixture was diluted with 27 mL EtOAc, washed with 0.5 M HCl and saturated *aq.* NaCl. The organic layer was dried over Na_2SO_4 and removed under reduced pressure to give the residue; the residue was purified by silica gel chromatography (40% EtOAc/hexane) to give the compound **75** (251 mg, 62% yield).

Compound 75
Physical State: amorphous solid
^{1}H NMR (400 MHz, $CDCl_3$): δ 7.66 (dd, *J* = 8.7, 5.2 Hz, 1H), 7.45 (dd, *J* = 10.1, 2.2 Hz, 1H), 7.41 – 7.32 (m, 6H), 6.87 (td, *J* = 8.8, 2.3 Hz, 1H), 6.31 (d, *J* = 7.2 Hz, 1H), 5.40 (td, *J* = 11.0, 4.2 Hz, 1H), 5.19 – 5.02 (m, 4H), 4.90 (q, *J* = 5.5, 4.7 Hz, 2H), 4.48 (dd, *J* = 8.5, 3.2 Hz, 1H), 4.33 (dt, *J* = 10.5, 5.6 Hz, 1H), 4.17 (ddd, *J* = 11.2, 7.1, 4.3 Hz, 1H), 3.73 (td, *J* = 9.9, 7.0 Hz, 1H), 3.46 (ddd, *J* = 10.8, 8.0, 3.2 Hz, 1H), 3.33 (t, *J* = 12.4 Hz, 1H), 3.14 (dd, *J* = 13.1, 4.2 Hz, 1H), 2.95 (p, *J* = 6.7 Hz, 1H), 2.90 – 2.80 (m, 1H), 2.52 (ddd, *J* = 14.7, 10.6, 4.1 Hz, 1H), 2.06 (d, *J* = 7.0 Hz, 1H), 1.93 – 1.84 (m, 1H), 1.74 (td, *J* = 10.1, 5.2 Hz, 1H), 1.65 – 1.58 (m, 2H), 1.53 (s, 9H), 1.42 – 1.36 (m, 1H), 1.00 – 0.90 (m, 21H), 0.79 – 0.74 (m, 1H), 0.60 – 0.50 (m, 1H).
^{13}C NMR (101 MHz, $CDCl_3$): δ 173.37, 170.26, 169.61, 169.25, 161.83, 159.42, 155.65, 154.44, 139.89, 139.76, 136.08, 134.97, 128.91, 127.94, 127.54, 127.20, 123.37, 118.77, 118.67, 110.42, 110.18, 100.49, 100.22, 80.31, 65.89, 60.08, 57.67, 50.38, 50.09, 46.95, 39.84, 35.61, 30.68, 28.97, 27.73, 27.61, 26.52, 23.73, 23.68, 22.84, 21.24, 7.06, 4.41.
^{19}F NMR (565 MHz, $CDCl_3$): δ -116.25.
HRMS (ESI-TOF): calculated for $C_{46}H_{65}FN_6NaO_8Si^+$ $[M+Na]^+$: 899.4509, found: 899.4517.
TLC: R_f = 0.6 (1:1 Hexane:EtOAc, $Ce_2(SO_4)_3$ in phosphomolybdic acid).
$[\alpha]^{25}_D$: –88.3 (*c* = 1.0, $CHCl_3$)

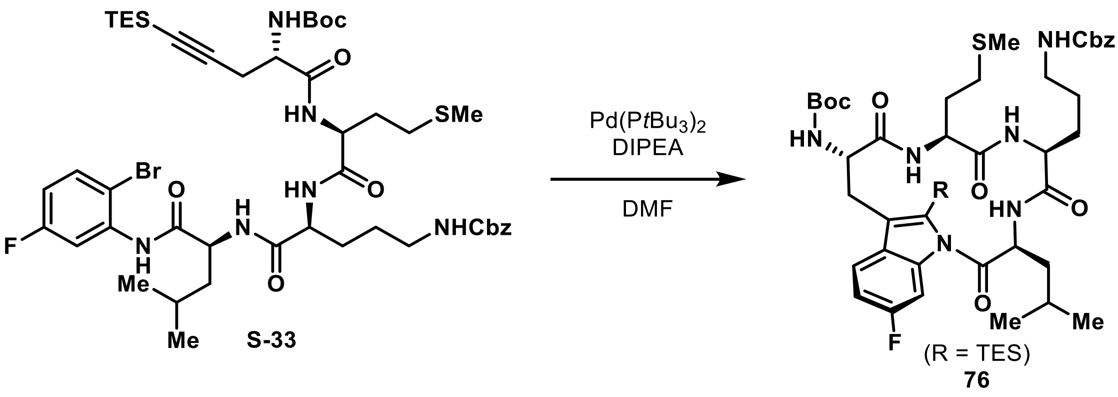

To a solution of compound **S-33** (494 mg, 0.498 mmol, 1.0 equiv.) in 10 mL anhydrous DMF was added DIPEA (0.26 mL, 1.5 mmol, 3.0 equiv.) and Pd(tBu$_3$P)$_2$ (25 mg, 0.05 mmol, 0.1 equiv.). The reaction mixture was stirred at 100 °C under nitrogen atmosphere for 12 h. After the consumption of the starting material, the mixture was diluted with 30 mL EtOAc, washed with 0.5 M HCl and saturated *aq.* NaCl. The organic layer was dried over Na_2SO_4 and removed under reduced pressure to give the residue; the residue was purified by silica gel chromatography (20% EtOAc/hexane) to give the compound **76** (150 mg, 33% yield).

Compound 76
Physical State: amorphous solid
^{1}H NMR (400 MHz, $CDCl_3$): δ 7.79 (d, J = 8.7 Hz, 1H), 7.53 (dd, J = 8.6, 5.2 Hz, 1H), 7.48 – 7.34 (m, 6H), 6.80 (t, J = 7.8 Hz, 1H), 6.28 (d, J = 7.4 Hz, 1H), 5.93 (d, J = 9.8 Hz, 1H), 5.35 – 5.16 (m, 3H), 5.00 – 4.83 (m, 2H), 4.73 – 4.58 (m, 1H), 4.40 (dd, J = 10.0, 5.1 Hz, 1H), 4.19 (ddd, J = 11.2, 7.2, 4.1 Hz, 1H), 3.38 (t, J = 12.6 Hz, 1H), 3.10 (dd, J = 13.1, 3.6 Hz, 1H), 2.76 – 2.63 (m, 1H), 2.59 – 2.48 (m, 2H), 2.42 (dt, J = 13.4, 7.9 Hz, 1H), 2.35 – 2.23 (m, 1H), 2.18 – 2.10 (m, 2H), 2.04 (s, 3H), 1.82 – 1.68 (m, 2H), 1.66 – 1.58 (m, 1H), 1.48 (s, 9H), 1.42 – 1.32 (m, 2H), 1.03 – 0.89 (m, 21H), 0.82 – 0.72 (m, 1H).
^{13}C NMR (101 MHz, $CDCl_3$): δ 173.80, 171.08, 170.45, 170.14, 161.85, 159.45, 156.26, 154.76, 139.47, 135.79, 128.32, 127.94, 127.62, 127.42, 123.74, 118.97, 118.86, 110.11, 109.86, 100.37, 100.10, 79.96, 66.50, 57.16, 53.33, 52.04, 49.91, 39.89, 35.50, 30.39, 29.46, 27.70, 23.77, 23.56, 23.07, 22.87, 21.18, 14.47, 7.08, 4.67.
^{19}F NMR (565 MHz, $CDCl_3$): δ -116.87.
HRMS (ESI-TOF): calculated for $C_{46}H_{67}FN_6NaO_8Si^+$ [M+Na]$^+$: 933.4387, found: 933.4392.
TLC: R_f = 0.45 (3:1 Hexane:EtOAc, $Ce_2(SO_4)_3$ in phosphomolybdic acid).
[α]$^{25}_{D}$: –52.6 (c = 1.0, $CHCl_3$)

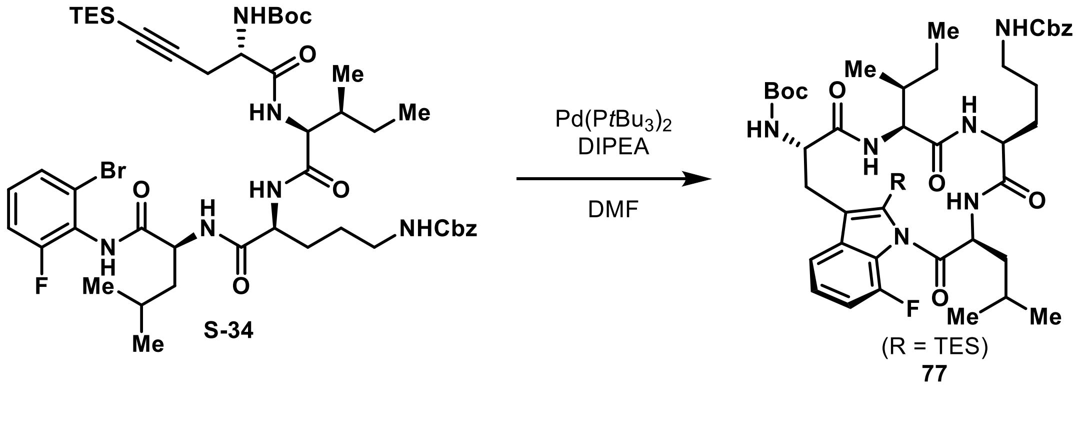

To a solution of compound **S-34** (800 mg, 0.822 mmol, 1.0 equiv.) in 17 mL anhydrous DMF was added DIPEA (0.43 mL, 2.5 mmol, 3.0 equiv.) and Pd(*t*$Bu_3P)_2$ (40 mg, 0.08 mmol, 0.1 equiv.). The reaction mixture was stirred at 100 °C under nitrogen atmosphere for 12 h. After the consumption of the starting material, the mixture was diluted with 50 mL EtOAc, washed with 0.5 M HCl and saturated *aq.* NaCl. The organic layer was dried over Na_2SO_4 and removed under reduced pressure to give the residue; the residue was purified by silica gel chromatography (35% EtOAc/hexane) to give the compound **77** (410 mg, 56% yield).

Compound 77

Physical State: amorphous solid

^{1}H NMR (400 MHz, $CDCl_3$): δ 7.50 – 7.29 (m, 6H), 7.21 (d, *J* = 8.4 Hz, 1H), 7.02 (dt, *J* = 8.0, 4.0 Hz, 1H), 6.90 (dd, *J* = 11.9, 7.8 Hz, 1H), 6.49 (d, *J* = 7.6 Hz, 1H), 5.99 (d, *J* = 9.2 Hz, 1H), 5.35 – 5.10 (m, 3H), 4.93 (h, *J* = 4.4 Hz, 2H), 4.30 (dq, *J* = 13.2, 7.0 Hz, 3H), 3.35 (t, *J* = 12.7 Hz, 1H), 3.15 (dd, *J* = 13.2, 3.9 Hz, 1H), 2.88 – 2.74 (m, 1H), 2.51 (d, *J* = 8.0 Hz, 1H), 2.39 (ddd, *J* = 14.7, 10.3, 4.2 Hz, 1H), 2.26 (s, 1H), 1.85 (ddd, *J* = 14.4, 9.8, 4.3 Hz, 2H), 1.68 – 1.53 (m, 2H), 1.47 (s, 9H), 1.33 – 1.18 (m, 2H), 1.10 – 1.03 (m, 2H), 1.00 – 0.87 (m, 21H), 0.85 (d, *J* = 6.9 Hz, 3H), 0.80 (t, *J* = 7.3 Hz, 3H).

^{13}C NMR (101 MHz, $CDCl_3$): δ 175.05, 171.10, 170.52, 169.89, 156.13, 154.93, 150.35, 147.83, 135.87, 131.08, 127.89, 127.51, 127.40, 127.15, 121.18, 113.81, 110.51, 110.31, 79.94, 66.37, 57.95, 52.97, 50.68, 39.64, 36.70, 35.60, 29.18, 27.67, 24.57, 24.01, 23.92, 22.75, 21.11, 14.85, 10.70, 7.08, 4.35.

^{19}F NMR (565 MHz, $CDCl_3$): δ -120.06.

HRMS (ESI-TOF): calculated for $C_{47}H_{69}FN_6NaO_8Si^+$ $[M+Na]^+$: 915.4822, found: 915.4830.

TLC: R_f = 0.5 (2:1 Hexane:EtOAc, $Ce_2(SO_4)_3$ in phosphomolybdic acid).

$[\alpha]^{25}_D$: –115.0 (*c* = 1.0, $CHCl_3$)

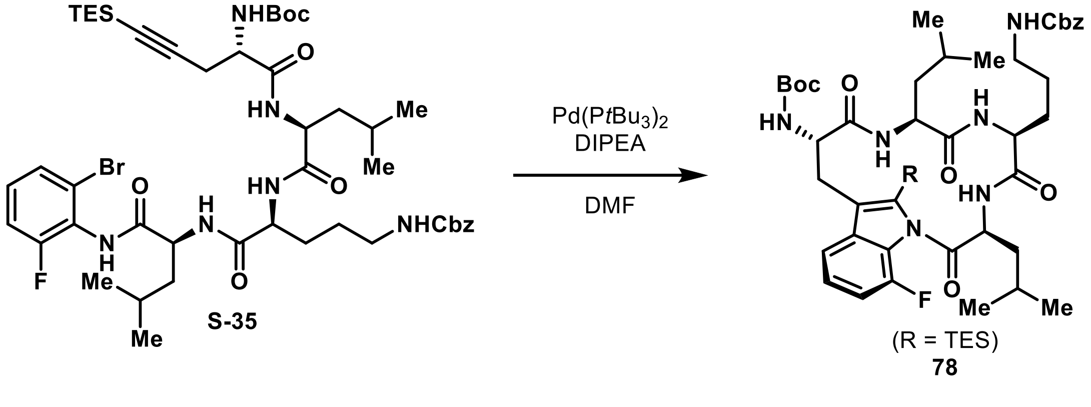

To a solution of compound **S-35** (480 mg, 0.490 mmol, 1.0 equiv.) in 10 mL anhydrous DMF was added DIPEA (0.26 mL, 1.5 mmol, 3.0 equiv.) and Pd(*t*$Bu_3P)_2$ (25 mg, 0.05 mmol, 0.1 equiv.). The reaction mixture was stirred at 100 °C under nitrogen atmosphere for 12 h. After the consumption of the starting material, the mixture was diluted with 30 mL EtOAc, washed with 0.5 M HCl and saturated *aq.* NaCl. The organic layer was dried over Na_2SO_4 and removed under reduced pressure to give the residue; the residue was purified by silica gel chromatography (40% EtOAc/hexane) to give the compound **78** (224 mg, 51% yield).

Compound 78

Physical State: amorphous solid

^{1}H NMR (400 MHz, $CDCl_3$): δ 7.48 – 7.27 (m, 7H), 7.01 (td, *J* = 7.9, 4.0 Hz, 1H), 6.91 (dd, *J* = 11.8, 7.8 Hz, 1H), 6.29 (d, *J* = 7.5 Hz, 1H), 5.70 (d, *J* = 9.5 Hz, 1H), 5.30 (d, *J* = 12.4 Hz, 1H), 5.24 – 5.13 (m, 2H), 5.00 (ddd, *J* = 12.7, 8.8, 4.1 Hz, 1H), 4.89 (d, *J* = 6.5 Hz, 1H), 4.46 – 4.32 (m, 1H), 4.30 – 4.13 (m, 2H), 3.36 (t, *J* = 12.8 Hz, 1H), 3.14 (dd, *J* = 13.2, 4.1 Hz, 1H), 2.95 – 2.77 (m, 1H), 2.72 – 2.56 (m, 1H), 2.49 – 2.29 (m, 2H),

1.83 (ddd, J = 14.3, 9.8, 4.2 Hz, 1H), 1.68 – 1.53 (m, 4H), 1.47 (s, 9H), 1.15 – 1.06 (m, 1H), 1.03 – 0.99 (m, 2H), 0.98 – 0.91 (m, 18H), 0.86 (dd, J = 11.7, 6.2 Hz, 9H).
^{13}C NMR (101 MHz, $CDCl_3$): δ 175.03, 171.75, 170.38, 169.69, 156.09, 154.90, 150.49, 147.97, 135.88, 130.83, 127.90, 127.54, 127.28, 121.15, 113.83, 110.57, 110.37, 80.02, 66.35, 58.43, 52.59, 51.78, 50.88, 39.80, 39.51, 36.40, 29.08, 27.61, 24.89, 24.25, 24.13, 23.92, 22.74, 22.51, 21.13, 20.43, 7.08, 4.27.
^{19}F NMR (565 MHz, $CDCl_3$): δ -120.13.
HRMS (ESI-TOF): calculated for $C_{47}H_{69}FN_6NaO_8Si^+$ $[M+Na]^+$: 915.4822, found: 915.4822.
TLC: R_f = 0.5 (2:1 Hexane:EtOAc, $Ce_2(SO_4)_3$ in phosphomolybdic acid).
$[\alpha]^{25}_D$: –125.9 (c = 1.0, $CHCl_3$)

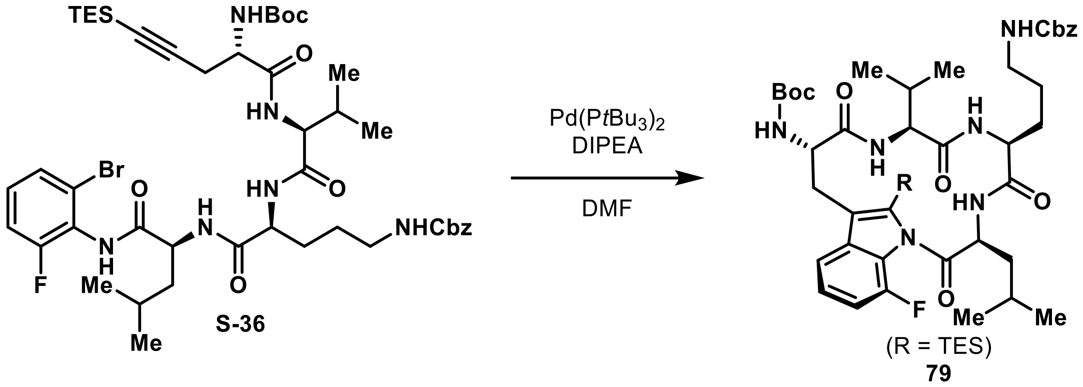

To a solution of compound **S-36** (587 mg, 0.613 mmol, 1.0 equiv.) in 13 mL anhydrous DMF was added DIPEA (0.33 mL, 1.9 mmol, 3.0 equiv.) and Pd($t$$Bu_3P)_2$ (30 mg, 0.06 mmol, 0.1 equiv.). The reaction mixture was stirred at 100 °C under nitrogen atmosphere for 12 h. After the consumption of the starting material, the mixture was diluted with 40 mL EtOAc, washed with 0.5 M HCl and saturated *aq.* NaCl. The organic layer was dried over Na_2SO_4 and removed under reduced pressure to give the residue; the residue was purified by silica gel chromatography (30% EtOAc/hexane) to give the compound **79** (286 mg, 53% yield).

Compound 79
Physical State: amorphous solid
^{1}H NMR (600 MHz, $CDCl_3$): δ 7.45 (d, J = 7.9 Hz, 1H), 7.40 (d, J = 7.5 Hz, 2H), 7.35 (t, J = 7.5 Hz, 2H), 7.29 (dd, J = 22.8, 7.8 Hz, 2H), 7.01 (td, J = 7.8, 3.6 Hz, 1H), 6.89 (dd, J = 11.8, 7.8 Hz, 1H), 6.40 (d, J = 7.6 Hz, 1H), 5.86 (d, J = 9.4 Hz, 1H), 5.28 – 5.11 (m, 3H), 4.95 (ddd, J = 12.5, 8.8, 4.0 Hz, 1H), 4.86 (d, J = 6.2 Hz, 1H), 4.43 – 4.05 (m, 3H), 3.34 (t, J = 12.7 Hz, 1H), 3.14 (dd, J = 13.2, 3.9 Hz, 1H), 2.95 – 2.70 (m, 1H), 2.57 – 2.45 (m, 1H), 2.39 (ddd, J = 14.6, 10.5, 4.3 Hz, 1H), 2.14 (tt, J = 13.4, 5.8 Hz, 1H), 2.02 (s, 2H), 1.84 (ddd, J = 14.3, 9.8, 4.1 Hz, 1H), 1.63 – 1.57 (m, 1H), 1.46 (s, 9H), 1.13 – 1.02 (m, 2H), 1.01 – 0.97 (m, 3H), 0.96 – 0.89 (m, 18H), 0.87 (d, J = 6.8 Hz, 3H), 0.75 (d, J = 6.8 Hz, 3H).
^{13}C NMR (151 MHz, $CDCl_3$): δ 175.74, 171.67, 171.43, 170.51, 156.82, 155.67, 136.61, 136.26, 128.63, 128.24, 128.14, 127.92, 121.89, 114.62, 111.23, 111.10, 80.69, 67.10, 59.11, 58.75, 53.65, 51.34, 40.41, 37.35, 29.94, 29.73, 28.40, 25.24, 24.72, 23.44, 21.81, 19.26, 17.52, 7.78, 5.07.
^{19}F NMR (565 MHz, $CDCl_3$): δ -120.00.
HRMS (ESI-TOF): calculated for $C_{46}H_{67}FN_6NaO_8Si^+$ $[M+Na]^+$: 901.4666, found: 901.4657.
TLC: R_f = 0.35 (2:1 Hexane:EtOAc, $Ce_2(SO_4)_3$ in phosphomolybdic acid).
$[\alpha]^{25}_D$: –105.3 (c = 1.0, $CHCl_3$)

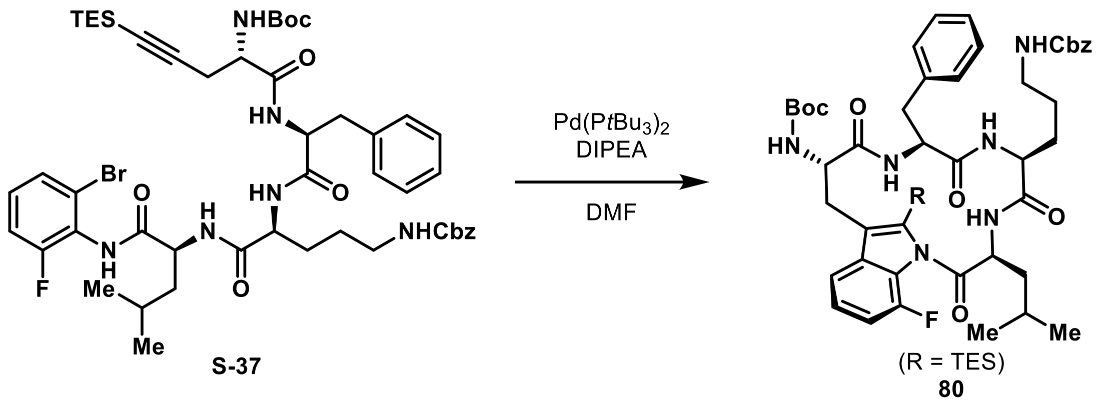

To a solution of compound **S-37** (404 mg, 0.400 mmol, 1.0 equiv.) in 8 mL anhydrous DMF was added DIPEA (0.21 mL, 1.2 mmol, 3.0 equiv.) and Pd(tBu$_3$P)$_2$ (20 mg, 0.04 mmol, 0.1 equiv.). The reaction mixture was stirred at 100 °C under nitrogen atmosphere for 12 h. After the consumption of the starting material, the mixture was diluted with 24 mL EtOAc, washed with 0.5 M HCl and saturated *aq.* NaCl. The organic layer was dried over Na_2SO_4 and removed under reduced pressure to give the residue; the residue was purified by silica gel chromatography (30% EtOAc/hexane) to give the compound **80** (182 mg, 49% yield).

Compound 80

Physical State: amorphous solid

^{1}H NMR (600 MHz, CDCl$_3$): δ 7.46 (dd, J = 7.9, 4.0 Hz, 3H), 7.38 (t, J = 7.3 Hz, 3H), 7.34 – 7.31 (m, 1H), 7.20 – 7.11 (m, 5H), 6.97 (td, J = 7.9, 3.7 Hz, 1H), 6.87 (dd, J = 11.8, 7.8 Hz, 1H), 6.29 (d, J = 7.7 Hz, 1H), 5.62 (d, J = 9.4 Hz, 1H), 5.31 (d, J = 12.5 Hz, 1H), 5.22 (d, J = 12.4 Hz, 1H), 5.06 (d, J = 8.9 Hz, 1H), 4.99 – 4.89 (m, 1H), 4.71 (q, J = 6.6 Hz, 2H), 4.36 – 4.12 (m, 2H), 3.28 (t, J = 12.7 Hz, 1H), 3.11 (dd, J = 13.4, 4.0 Hz, 1H), 3.00 (dd, J = 14.1, 4.8 Hz, 1H), 2.90 (dd, J = 14.1, 8.3 Hz, 1H), 2.67 (dd, J = 14.7, 7.8 Hz, 1H), 2.53 – 2.43 (m, 1H), 2.42 – 2.31 (m, 1H), 1.88 – 1.83 (m, 1H), 1.62 – 1.56 (m, 1H), 1.46 (s, 9H), 1.42 – 1.37 (m, 1H), 1.04 – 0.97 (m, 2H), 0.96 (d, J = 6.6 Hz, 3H), 0.94 – 0.89 (m, 15H), 0.89 – 0.84 (m, 3H), 0.71 – 0.60 (m, 1H).

^{13}C NMR (151 MHz, CDCl$_3$): δ 171.02, 170.82, 170.17, 156.70, 155.37, 136.64, 136.11, 129.40, 128.65, 128.28, 128.16, 127.89, 127.10, 121.90, 114.78, 111.20, 80.53, 67.11, 58.79, 54.50, 53.49, 51.97, 40.50, 37.55, 37.23, 30.50, 28.48, 25.12, 24.72, 24.51, 23.42, 21.82, 7.75, 4.99.

^{19}F NMR (565 MHz, CDCl$_3$): δ -120.32.

HRMS (ESI-TOF): calculated for $C_{50}H_{67}FN_6NaO_8Si^+$ [M+Na]$^+$: 949.4666, found: 949.4670.

TLC: R_f = 0.45 (2:1 Hexane:EtOAc, $Ce_2(SO_4)_3$ in phosphomolybdic acid).

$[\alpha]^{25}_D$: –96.9 (c = 1.0, $CHCl_3$)

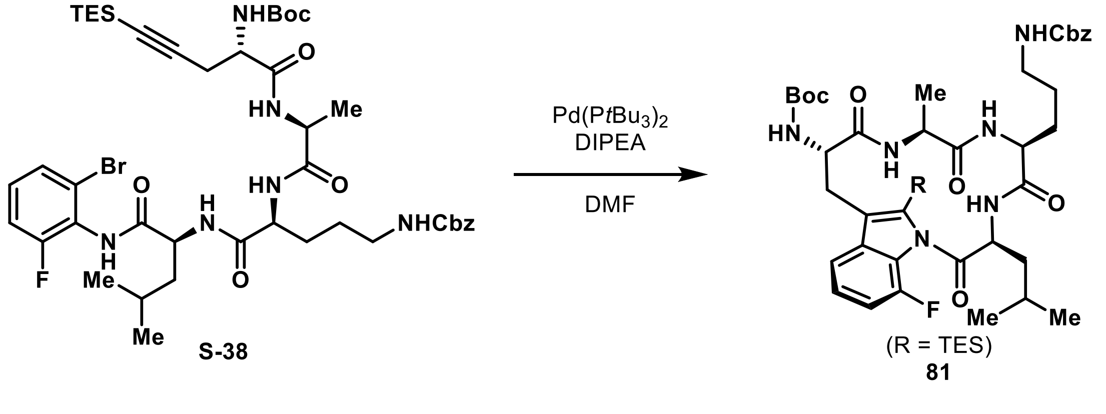

To a solution of compound **S-38** (411 mg, 0.440 mmol, 1.0 equiv.) in 9 mL anhydrous DMF was added DIPEA (0.25 mL, 1.4 mmol, 3.0 equiv.) and Pd(tBu$_3$P)$_2$ (20 mg, 0.04 mmol, 0.1 equiv.). The reaction mixture was stirred at 100 °C under nitrogen atmosphere for 12 h. After the consumption of the starting material, the mixture was diluted with 27 mL EtOAc, washed with 0.5 M HCl and saturated *aq.* NaCl. The organic layer was dried over Na_2SO_4 and removed under reduced pressure to give the residue; the residue was purified by silica gel chromatography (30% EtOAc/hexane) to give the compound **81** (150 mg, 40% yield).

Compound 81
Physical State: amorphous solid
^{1}H NMR (400 MHz, CDCl$_3$): δ 7.52 (d, J = 7.8 Hz, 1H), 7.47 – 7.41 (m, 2H), 7.41 – 7.29 (m, 5H), 7.03 (td, J = 8.0, 4.3 Hz, 1H), 6.93 (dd, J = 11.8, 7.8 Hz, 1H), 6.37 (d, J = 7.6 Hz, 1H), 5.72 (d, J = 9.4 Hz, 1H), 5.22 (t, J = 10.5 Hz, 3H), 5.03 (ddd, J = 12.7, 8.9, 4.2 Hz, 1H), 4.87 (t, J = 6.2 Hz, 1H), 4.49 – 4.37 (m, 1H), 4.33 – 4.15 (m, 2H), 3.33 (t, J = 12.7 Hz, 1H), 3.17 (dd, J = 13.3, 4.2 Hz, 1H), 2.97 – 2.83 (m, 1H), 2.78 – 2.62 (m, 1H), 2.40 (ddd, J = 14.9, 10.6, 4.4 Hz, 1H), 1.86 (ddd, J = 14.2, 9.8, 4.2 Hz, 1H), 1.67 – 1.57 (m, 2H), 1.50 (s, 9H), 1.26 (d, J = 7.3 Hz, 3H), 1.16 – 1.07 (m, 1H), 1.01 – 0.99 (m, 1H), 0.97 – 0.88 (m, 21H).
^{13}C NMR (101 MHz, CDCl$_3$): δ 175.36, 171.72, 169.87, 169.60, 156.11, 154.91, 150.40, 147.88, 135.83, 130.88, 127.93, 127.56, 127.45, 127.22, 121.18, 114.03, 110.48, 110.29, 80.10, 66.41, 58.27, 52.65, 51.43, 48.65, 39.82, 36.88, 29.65, 27.70, 24.98, 24.29, 23.99, 22.70, 21.12, 16.84, 7.04, 4.20.
^{19}F NMR (565 MHz, CDCl$_3$): δ -120.50.
HRMS (ESI-TOF): calculated for $C_{44}H_{63}FN_6NaO_8Si^+$ [M+Na]$^+$: 873.4353, found: 873.4359.
TLC: R$_f$ = 0.6 (1:1 Hexane:EtOAc, $Ce_2(SO_4)_3$ in phosphomolybdic acid).
[α]$^{25}_D$: –111.6 (c = 1.0, CHCl$_3$)

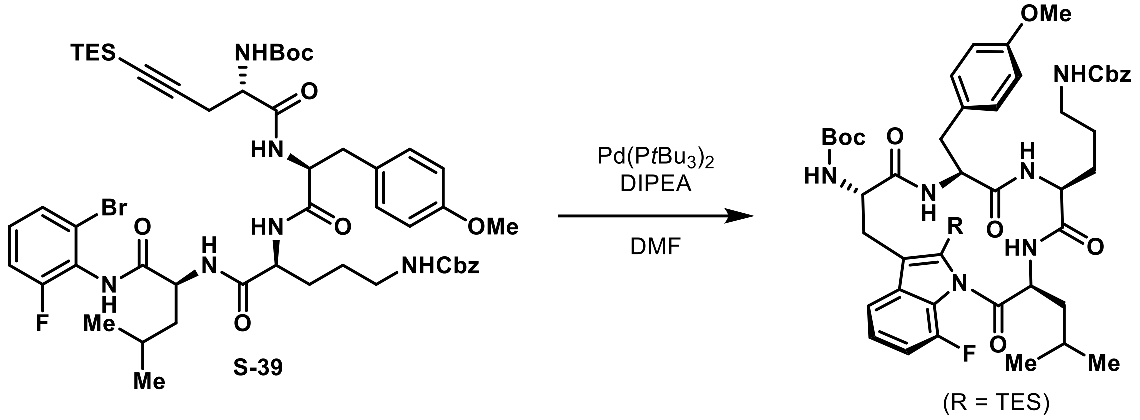

To a solution of compound **S-39** (479 mg, 0.461 mmol, 1.0 equiv.) in 9 mL anhydrous DMF was added DIPEA (0.25 mL, 1.4 mmol, 3.0 equiv.) and Pd(tBu$_3$P)$_2$ (25 mg, 0.05 mmol, 0.1 equiv.). The reaction mixture was stirred at 100 °C under nitrogen atmosphere for 12 h. After the consumption of the starting material, the mixture was diluted with 27 mL EtOAc, washed with 0.5 M HCl and saturated *aq.* NaCl. The organic layer was dried over Na_2SO_4 and removed under reduced pressure to give the residue; the residue was purified by silica gel chromatography (30% EtOAc/hexane) to give the compound **82** (212 mg, 48% yield).

Compound 82
Physical State: amorphous solid
^{1}H NMR (400 MHz, CDCl$_3$): δ 7.48 (dd, J = 7.9, 3.0 Hz, 3H), 7.42 – 7.33 (m, 4H), 7.05 (d, J = 8.3 Hz, 2H), 6.99 (q, J = 3.7 Hz, 1H), 6.89 (dd, J = 11.9, 7.8 Hz, 1H), 6.72 (d, J = 8.1 Hz, 2H), 6.34 (d, J = 7.6 Hz, 1H), 5.66 (d, J = 9.4 Hz, 1H), 5.34 (d, J = 12.5 Hz, 1H), 5.23 (d, J = 12.4 Hz, 1H), 5.12 (d, J = 8.8 Hz, 1H), 5.03 –

4.93 (m, 1H), 4.80 (t, *J* = 5.9 Hz, 1H), 4.67 (q, *J* = 7.3 Hz, 1H), 4.34 – 4.17 (m, 2H), 3.71 (s, 3H), 3.30 (t, *J* = 12.6 Hz, 1H), 3.14 (dd, *J* = 13.3, 4.0 Hz, 1H), 2.98 – 2.83 (m, 2H), 2.72 – 2.60 (m, 1H), 2.53 – 2.43 (m, 1H), 2.43 – 2.33 (m, 1H), 2.08 (s, 2H), 1.87 (ddd, *J* = 14.2, 9.9, 4.1 Hz, 1H), 1.64 – 1.57 (m, 1H), 1.48 (s, 9H), 1.42 – 1.36 (m, 1H), 0.99 – 0.88 (m, 21H), 0.74 – 0.62 (m, 1H).
^{13}C NMR (101 MHz, $CDCl_3$): δ 175.22, 170.38, 170.09, 169.50, 157.95, 155.96, 154.67, 150.28, 147.76, 135.95, 135.53, 130.94, 129.72, 127.92, 127.56, 127.47, 127.27, 121.18, 114.06, 113.31, 110.53, 110.33, 79.81, 66.35, 58.04, 54.52, 53.98, 52.78, 51.27, 39.77, 36.86, 35.68, 29.78, 27.74, 24.43, 23.99, 23.75, 22.72, 21.10, 7.05, 4.27.
^{19}F NMR (565 MHz, $CDCl_3$): δ -120.40.
HRMS (ESI-TOF): calculated for $C_{51}H_{69}FN_6NaO_9Si^+$ $[M+Na]^+$: 979.4772, found: 979.4779.
TLC: R_f = 0.5 (2:1 Hexane:EtOAc, $Ce_2(SO_4)_3$ in phosphomolybdic acid).
$[\alpha]^{25}_D$: –73.1 (*c* = 1.0, $CHCl_3$)

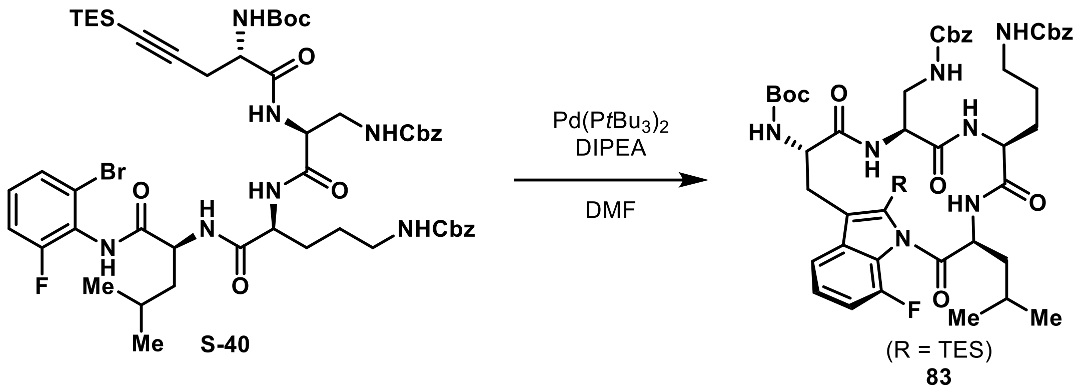

To a solution of compound **S-40** (605 mg, 0.560 mmol, 1.0 equiv.) in 11 mL anhydrous DMF was added DIPEA (0.30 mL, 1.7 mmol, 3.0 equiv.) and Pd(*t*$Bu_3P)_2$ (30 mg, 0.06 mmol, 0.1 equiv.). The reaction mixture was stirred at 100 °C under nitrogen atmosphere for 12 h. After the consumption of the starting material, the mixture was diluted with 33 mL EtOAc, washed with 0.5 M HCl and saturated *aq.* NaCl. The organic layer was dried over Na_2SO_4 and removed under reduced pressure to give the residue; the residue was purified by silica gel chromatography (40% EtOAc/hexane) to give the compound **83** (252 mg, 45% yield).

Compound 83
Physical State: amorphous solid
^{1}H NMR (600 MHz, $CDCl_3$): δ 7.92 – 7.75 (m, 1H), 7.50 (d, *J* = 7.8 Hz, 1H), 7.35 – 7.26 (m, 11H), 7.03 – 6.97 (m, 1H), 6.90 (dd, *J* = 11.8, 7.8 Hz, 1H), 6.39 (d, *J* = 7.5 Hz, 1H), 5.78 (s, 1H), 5.48 (d, *J* = 9.5 Hz, 1H), 5.22 (d, *J* = 8.1 Hz, 1H), 5.10 (d, *J* = 6.8 Hz, 2H), 5.03 (d, *J* = 3.0 Hz, 2H), 5.00 – 4.96 (m, 1H), 4.84 (s, 1H), 4.50 (s, 1H), 4.32 (s, 1H), 4.20 (s, 1H), 3.55 – 3.45 (m, 1H), 3.42 – 3.33 (m, 1H), 3.29 – 3.17 (m, 2H), 2.93 – 2.83 (m, 1H), 2.63 – 2.53 (m, 1H), 2.34 (t, *J* = 11.9 Hz, 1H), 1.86 (ddd, *J* = 14.3, 10.0, 4.2 Hz, 1H), 1.63 – 1.57 (m, 2H), 1.56 – 1.51 (m, 1H), 1.45 (s, 9H), 1.41 – 1.38 (m, 1H), 1.06 – 1.01 (m, 1H), 0.96 – 0.89 (m, 21H).
^{13}C NMR (151 MHz, $CDCl_3$): δ 176.41, 171.37, 170.11, 169.67, 156.87, 155.40, 136.49, 131.67, 128.62, 128.60, 128.28, 128.21, 122.06, 115.02, 110.90, 108.72, 80.76, 67.06, 58.90, 54.89, 53.77, 51.88, 42.46, 40.43, 37.97, 30.70, 28.43, 25.27, 24.75, 23.39, 21.78, 7.72, 7.55, 4.82.
^{19}F NMR (565 MHz, $CDCl_3$): δ -120.83.
HRMS (ESI-TOF): calculated for $C_{52}H_{70}FN_7NaO_{10}Si^+$ $[M+Na]^+$: 1022.4830, found: 1022.4815.
TLC: R_f = 0.6 (1:1 Hexane:EtOAc, $Ce_2(SO_4)_3$ in phosphomolybdic acid).
$[\alpha]^{25}_D$: –81.9 (*c* = 1.0, $CHCl_3$)

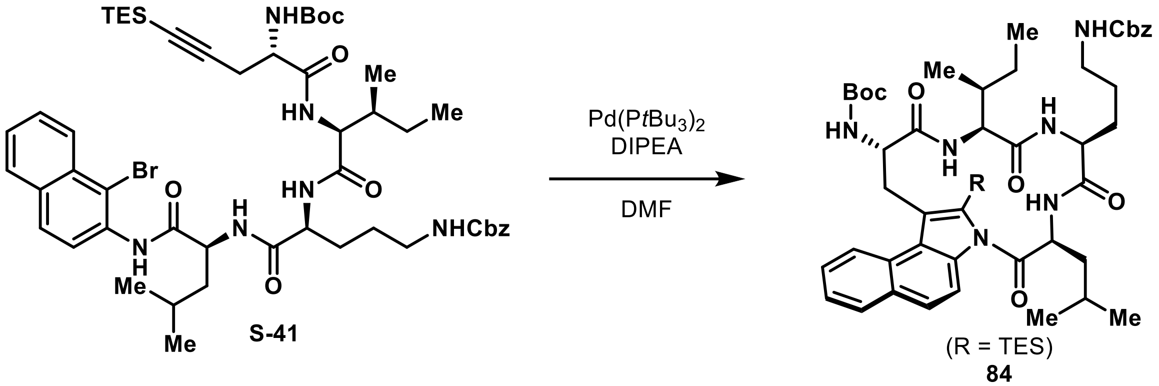

S-41

(R = TES)
84

To a solution of compound **S-41** (333 mg, 0.331 mmol, 1.0 equiv.) in 7 mL anhydrous DMF was added DIPEA (0.17 mL, 1.0 mmol, 3.0 equiv.) and Pd(tBu$_3$P)$_2$ (15 mg, 0.03 mmol, 0.1 equiv.). The reaction mixture was stirred at 100 °C under nitrogen atmosphere for 12 h. After the consumption of the starting material, the mixture was diluted with 20 mL EtOAc, washed with 0.5 M HCl and saturated *aq*. NaCl. The organic layer was dried over Na_2SO_4 and removed under reduced pressure to give the residue; the residue was purified by silica gel chromatography (20% EtOAc/hexane) to give the compound **84** (49 mg, 16% yield).

Compound 84
Physical State: amorphous solid
^{1}H NMR (400 MHz, CDCl$_3$): δ 8.34 (d, J = 8.4 Hz, 1H), 7.87 (dd, J = 8.8, 2.3 Hz, 2H), 7.59 (d, J = 9.1 Hz, 1H), 7.53 (t, J = 7.6 Hz, 1H), 7.43 – 7.28 (m, 7H), 6.50 (d, J = 7.3 Hz, 1H), 6.32 – 6.17 (m, 1H), 5.27 (d, J = 9.2 Hz, 1H), 5.04 (d, J = 12.7 Hz, 1H), 4.95 (td, J = 10.5, 9.0, 2.8 Hz, 1H), 4.66 (d, J = 12.8 Hz, 1H), 4.50 – 4.18 (m, 4H), 3.73 (t, J = 12.7 Hz, 1H), 3.51 (dd, J = 13.3, 2.9 Hz, 1H), 2.64 – 2.50 (m, 1H), 2.45 (s, 1H), 1.93 (s, 1H), 1.85 (ddd, J = 14.2, 9.8, 4.1 Hz, 1H), 1.75 (d, J = 6.1 Hz, 1H), 1.67 (d, J = 4.9 Hz, 2H), 1.50 (s, 9H), 1.41 – 1.35 (m, 2H), 1.30 – 1.24 (m, 1H), 1.13 (dt, J = 12.5, 6.2 Hz, 3H), 1.03 (d, J = 6.6 Hz, 3H), 1.00 – 0.93 (m, 15H), 0.93 – 0.87 (m, 2H), 0.81 (d, J = 6.8 Hz, 3H), 0.72 (t, J = 7.3 Hz, 3H).
^{13}C NMR (151 MHz, CDCl$_3$): δ 175.19, 172.36, 171.28, 171.28, 156.84, 156.50, 155.45, 142.59, 137.88, 130.89, 130.56, 129.02, 128.40, 127.90, 127.80, 127.48, 126.66, 125.89, 124.17, 123.14, 114.77, 80.49, 66.93, 58.93, 58.17, 53.95, 39.75, 36.31, 36.31, 31.56, 28.36, 27.05, 24.66, 24.52, 23.62, 21.95, 15.53, 11.25, 7.93, 5.85.
HRMS (ESI-TOF): calculated for $C_{51}H_{72}N_6NaO_8Si^+$ [M+Na]$^+$: 947.5073, found: 947.5059.
TLC: R_f = 0.4 (3:1 Hexane:EtOAc, $Ce_2(SO_4)_3$ in phosphomolybdic acid).
[α]$^{25}_D$: –111.2 (c = 1.0, CHCl$_3$)

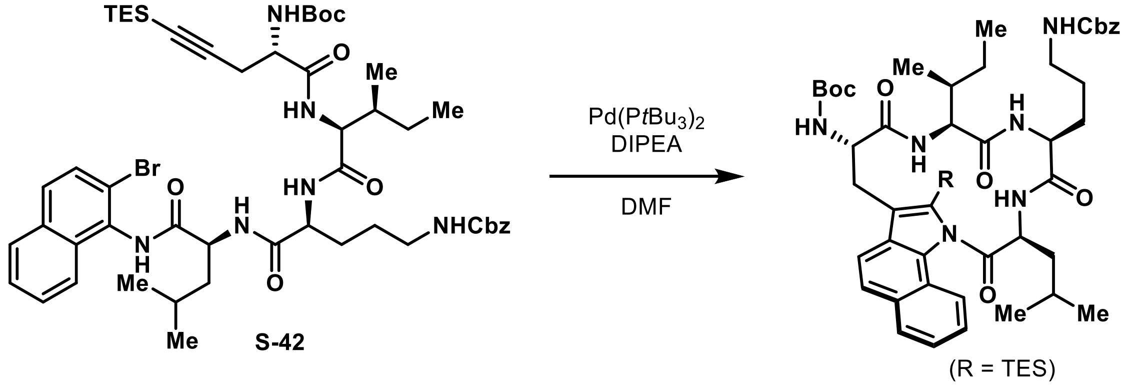

S-42

(R = TES)
85

To a solution of compound **S-42** (702 mg, 0.697 mmol, 1.0 equiv.) in 15 mL anhydrous DMF was added DIPEA (0.36 mL, 2.1 mmol, 3.0 equiv.) and Pd(*t*Bu$_3$P)$_2$ (35 mg, 0.07 mmol, 0.1 equiv.). The reaction mixture was stirred at 100 °C under nitrogen atmosphere for 12 h. After the consumption of the starting material, the mixture was diluted with 45 mL EtOAc, washed with 0.5 M HCl and saturated *aq.* NaCl. The organic layer was dried over Na_2SO_4 and removed under reduced pressure to give the residue; the residue was purified by silica gel chromatography (20% EtOAc/hexane) to give the compound **85** (310 mg, 48% yield).

Compound 85
Physical State: amorphous solid
^{1}H NMR (400 MHz, CDCl$_3$): δ 8.59 (d, *J* = 8.7 Hz, 1H), 7.80 (d, *J* = 8.0 Hz, 1H), 7.73 (d, *J* = 8.6 Hz, 1H), 7.52 (dd, *J* = 8.4, 3.4 Hz, 2H), 7.49 – 7.32 (m, 6H), 7.28 (d, *J* = 5.0 Hz, 1H), 6.21 (d, *J* = 7.3 Hz, 1H), 5.36 (d, *J* = 9.6 Hz, 1H), 5.20 (d, *J* = 11.5 Hz, 3H), 5.12 (td, *J* = 10.1, 8.1, 4.0 Hz, 1H), 4.35 (t, *J* = 5.9 Hz, 1H), 4.16 (dp, *J* = 22.7, 5.4 Hz, 3H), 3.42 (t, *J* = 12.6 Hz, 1H), 3.27 (dd, *J* = 13.3, 4.2 Hz, 1H), 2.65 (ddd, *J* = 14.7, 10.7, 4.3 Hz, 1H), 2.30 (dp, *J* = 25.4, 6.7 Hz, 2H), 1.92 (d, *J* = 8.7 Hz, 1H), 1.82 (ddd, *J* = 14.3, 10.0, 4.1 Hz, 1H), 1.61 – 1.54 (m, 1H), 1.51 (s, 9H), 1.28 – 1.17 (m, 1H), 1.08 – 1.04 (m, 1H), 1.03 – 0.91 (m, 21H), 0.88 (d, *J* = 6.9 Hz, 3H), 0.84 – 0.75 (m, 4H), 0.62 (p, *J* = 6.6 Hz, 1H), 0.44 (q, *J* = 8.0, 7.5 Hz, 1H), 0.16 (q, *J* = 7.6 Hz, 1H).
^{13}C NMR (101 MHz, CDCl$_3$): δ 179.58, 171.10, 170.15, 169.30, 155.76, 155.33, 136.05, 135.30, 134.51, 131.58, 128.86, 127.92, 127.52, 127.22, 125.12, 124.59, 124.04, 123.64, 122.85, 122.66, 116.91, 80.40, 66.19, 60.25, 58.67, 52.53, 49.81, 39.28, 35.62, 35.04, 28.66, 27.71, 24.33, 24.11, 23.88, 23.83, 22.77, 21.17, 15.09, 10.89, 7.15, 4.45.
HRMS (ESI-TOF): calculated for $C_{51}H_{72}N_6NaO_8Si^+$ [M+Na]$^+$: 947.5073, found: 947.5074.
TLC: R_f = 0.5 (3:1 Hexane:EtOAc, $Ce_2(SO_4)_3$ in phosphomolybdic acid).
$[\alpha]^{25}_D$: –98.5 (*c* = 1.0, CHCl$_3$)

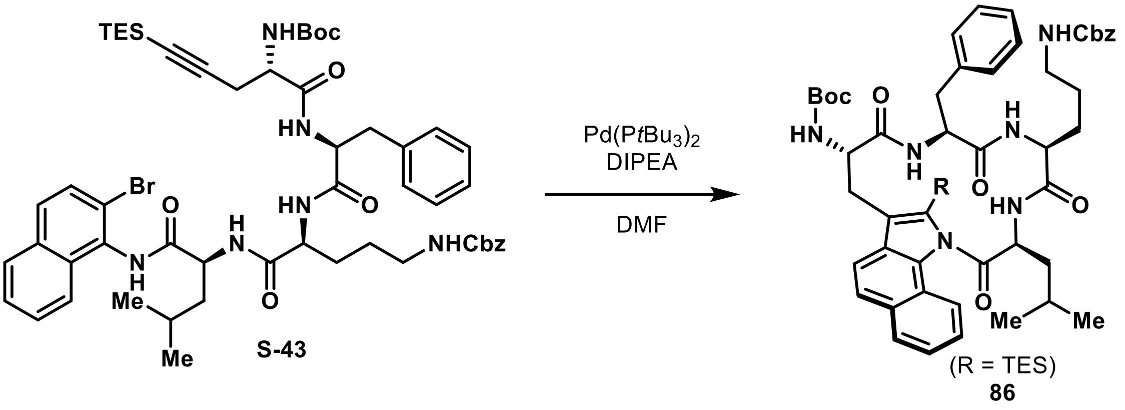

To a solution of compound **S-43** (496 mg, 0.493 mmol, 1.0 equiv.) in 10 mL anhydrous DMF was added DIPEA (0.26 mL, 1.5 mmol, 3.0 equiv.) and Pd(*t*Bu$_3$P)$_2$ (25 mg, 0.05 mmol, 0.1 equiv.). The reaction mixture was stirred at 100 °C under nitrogen atmosphere for 12 h. After the consumption of the starting material, the mixture was diluted with 30 mL EtOAc, washed with 0.5 M HCl and saturated *aq.* NaCl. The organic layer was dried over Na_2SO_4 and removed under reduced pressure to give the residue; the residue was purified by silica gel chromatography (20% EtOAc/hexane) to give the compound **86** (210 mg, 46% yield).

Compound 86
Physical State: amorphous solid
^{1}H NMR (400 MHz, CDCl$_3$): δ 8.55 (d, *J* = 8.7 Hz, 1H), 7.76 (d, *J* = 8.0 Hz, 1H), 7.70 (d, *J* = 8.6 Hz, 1H), 7.44 (ddt, *J* = 26.9, 19.7, 7.2 Hz, 9H), 7.25 – 7.11 (m, 5H), 6.13 (d, *J* = 7.3 Hz, 1H), 5.27 (dd, *J* = 20.5, 8.4

Hz, 3H), 5.08 (d, J = 5.0 Hz, 2H), 4.62 (q, J = 6.4 Hz, 1H), 4.34 – 4.03 (m, 3H), 3.37 (td, J = 10.8, 8.5, 4.8 Hz, 1H), 3.22 (dd, J = 13.3, 3.2 Hz, 1H), 2.99 (d, J = 6.2 Hz, 2H), 2.64 (ddd, J = 14.7, 10.7, 4.3 Hz, 1H), 2.27 (dq, J = 19.2, 6.8 Hz, 2H), 1.84 (ddd, J = 14.4, 10.0, 4.2 Hz, 1H), 1.62 – 1.55 (m, 1H), 1.50 (s, 9H), 0.96 (ddd, J = 13.2, 8.2, 4.6 Hz, 21H), 0.69 (s, 1H), 0.26 (dd, J = 12.1, 5.9 Hz, 1H), 0.13 (s, 2H).
^{13}C NMR (101 MHz, $CDCl_3$): δ 179.63, 170.54, 169.77, 169.01, 155.78, 155.02, 136.09, 135.09, 134.15, 131.61, 128.86, 128.55, 128.08, 127.95, 127.55, 127.50, 127.25, 126.57, 125.06, 124.56, 124.01, 123.52, 122.83, 122.53, 116.94, 80.19, 66.24, 60.18, 54.28, 52.56, 50.18, 39.33, 36.11, 35.69, 29.18, 27.79, 23.89, 23.81, 22.77, 21.20, 7.14, 4.42.
HRMS (ESI-TOF): calculated for $C_{54}H_{70}N_6NaO_8Si^+$ $[M+Na]^+$: 981.4917, found: 981.4918.
TLC: R_f = 0.5 (3:1 Hexane:EtOAc, $Ce_2(SO_4)_3$ in phosphomolybdic acid).
$[\alpha]^{25}_D$: –127.7 (c = 1.0, $CHCl_3$)

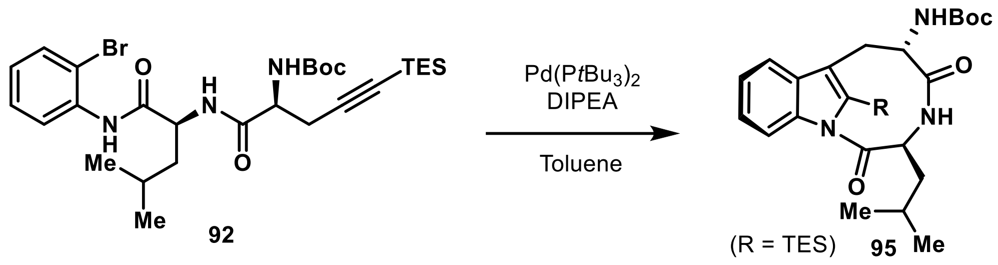

To a solution of compound **92** (150 mg, 0.252 mmol, 1.0 equiv.) in 25 mL toluene was added DIPEA (0.13 mL, 0.76 mmol, 3.0 equiv.) and Pd(tBu$_3$P)$_2$ (38 mg, 0.076 mmol, 0.3 equiv.). The reaction mixture was stirred at 110 °C under nitrogen atmosphere for 6 h. After the consumption of the starting material, the mixture was diluted with 30 mL EtOAc, washed with 0.5 M HCl and saturated *aq.* NaCl. The organic layer was dried over Na_2SO_4 and removed under reduced pressure to give the residue; the residue was purified by silica gel chromatography (20% EtOAc/hexane) to give the product **95** (61 mg, 47% yield).

Compound 95
Physical State: amorphous solid
^{1}H NMR (600 MHz, $CDCl_3$): δ 7.80 (s, 1H), 7.17 (t, J = 7.7 Hz, 1H), 7.08 (td, J = 7.5, 1.3 Hz, 1H), 7.00 (dd, J = 7.7, 1.6 Hz, 1H), 6.64 (d, J = 7.2 Hz, 1H), 6.61 – 6.44 (m, 2H), 6.00 (s, 1H), 4.77 (s, 1H), 1.85 – 1.72 (m, 3H), 1.59 (q, J = 9.4, 6.8 Hz, 1H), 1.48 (s, 9H), 0.99 (t, J = 6.2 Hz, 6H), 0.90 (t, J = 7.9 Hz, 9H), 0.75 – 0.64 (m, 6H).
^{13}C NMR (151 MHz, $CDCl_3$): δ 166.35, 152.36, 133.40, 128.39, 127.05, 124.48, 112.31, 81.66, 53.62, 28.32, 28.25, 25.04, 23.04, 21.85, 7.57, 4.19.
HRMS (ESI-TOF): calculated for $C_{28}H_{43}N_3NaO_4Si^+$ $[M+Na]^+$: 536.2915, found: 536.2913.
TLC: R_f = 0.3 (1:4 hexane:EtOAc, $Ce_2(SO_4)_3$ in phosphomolybdic acid).
$[\alpha]^{25}_D$: –78.0 (c = 0.1, $CHCl_3$)

LCMS analysis of compound 95 after separation:

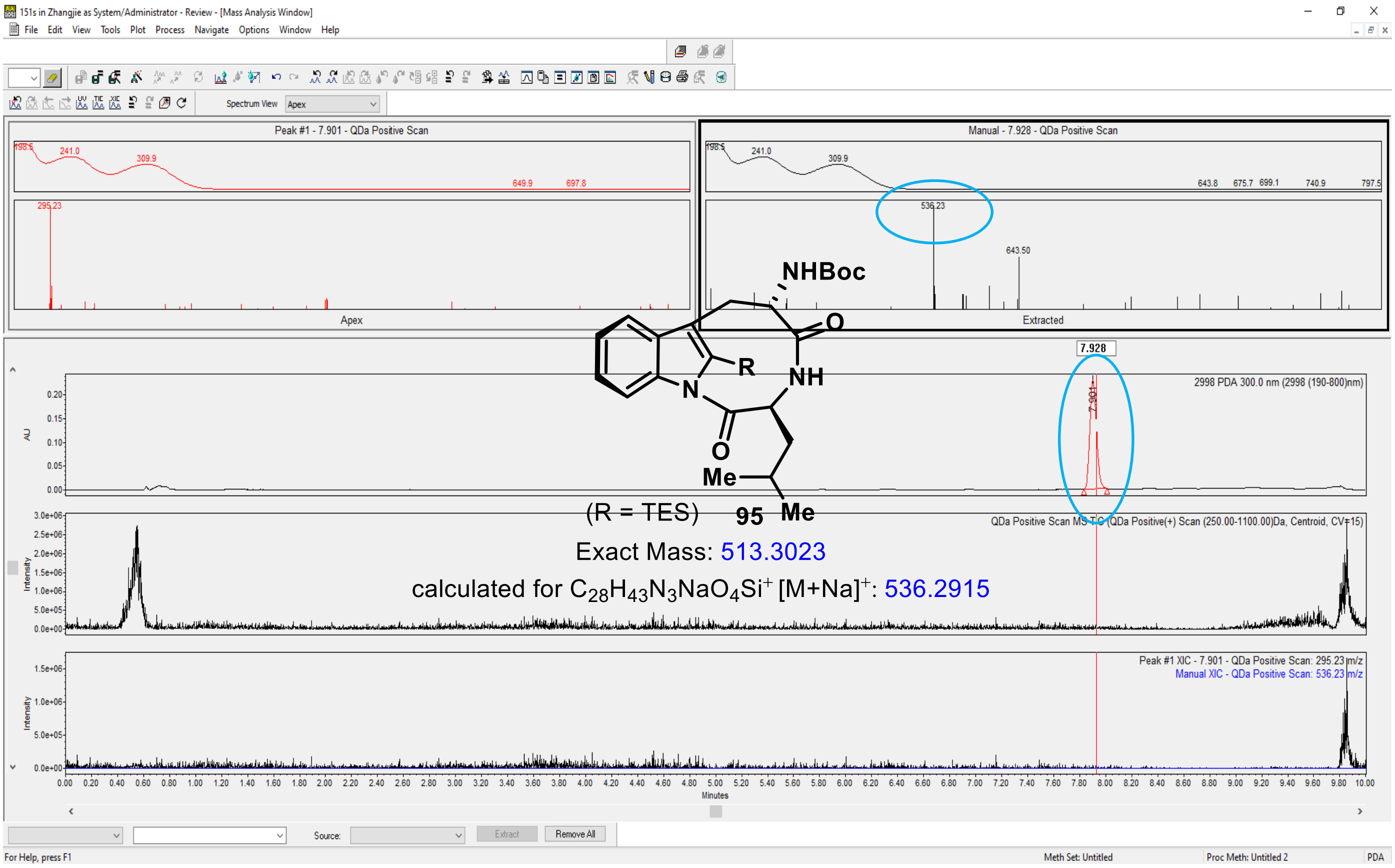

HRMS analysis of compound 95 after separation:

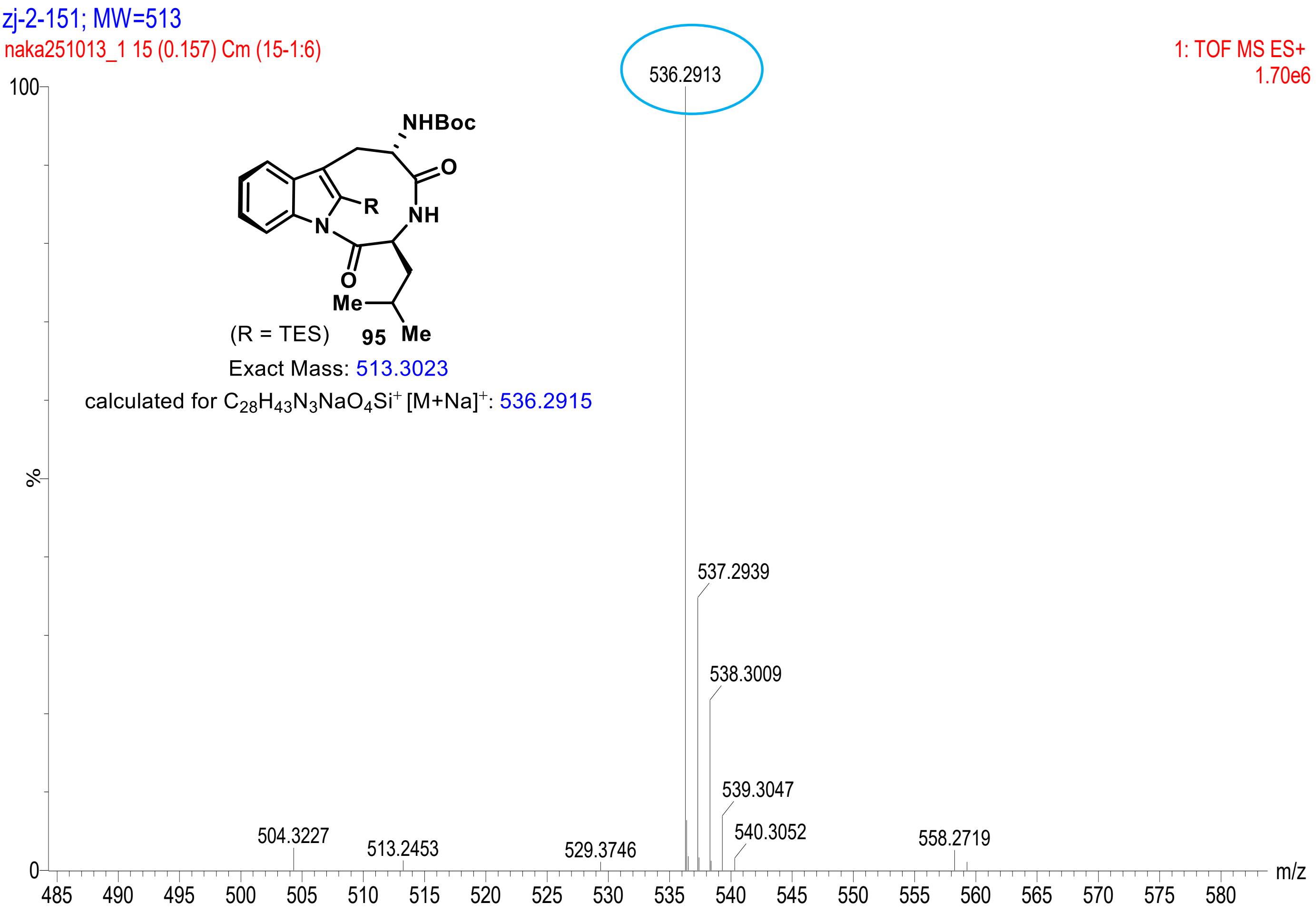

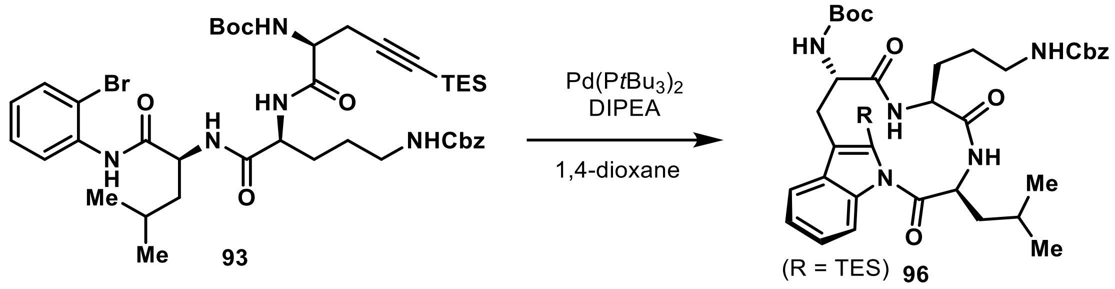

To a solution of compound **93** (168 mg, 0.2 mmol, 1.0 equiv.) in 20 mL 1,4-dioxane was added DIPEA (0.11 mL, 0.6 mmol, 3.0 equiv.) and Pd(tBu$_3$P)$_2$ (31 mg, 0.06 mmol, 0.3 equiv.). The reaction mixture was stirred at 110 °C under nitrogen atmosphere for 6 h. After the consumption of the starting material, the mixture was diluted with 30 mL EtOAc, washed with 0.5 M HCl and saturated *aq.* NaCl. The organic layer was dried over Na_2SO_4 and removed under reduced pressure to give the residue; the residue was purified by silica gel chromatography (40% EtOAc/hexane) to give the product **96** (67 mg, 44% yield).

Compound 96
Physical State: amorphous solid
^{1}H NMR (400 MHz, CDCl$_3$, 25 °C): mixture of conformational isomers (not an impurity) δ 8.36 – 7.50 (m, 2H), 7.40 – 7.28 (m, 5H), 7.20 – 6.94 (m, 3H), 6.92 – 6.40 (m, 2H), 5.30 – 4.95 (m, 3H), 4.89 – 4.28 (m, 1H), 4.25 – 3.57 (m, 1H), 3.34 – 2.80 (m, 2H), 2.00 – 1.67 (m, 5H), 1.66 – 1.48 (m, 4H), 1.48 – 1.35 (m, 9H), 1.35 – 1.30 (m, 1H), 1.00 – 0.79 (m, 15H), 0.77 – 0.49 (m, 6H).
^{13}C NMR (101 MHz, CDCl$_3$, 25 °C): mixture of conformational isomers (not an impurity) δ 170.91, 169.18, 167.01, 156.16, 150.88, 139.93, 139.11, 138.59, 135.83, 134.51, 132.26, 132.09, 127.95, 127.94, 127.87, 127.60, 127.49, 127.44, 126.10, 125.80, 122.85, 111.16, 81.18, 66.22, 66.00, 39.06, 38.11, 29.08, 29.00, 27.60, 27.52, 27.48, 25.57, 24.44, 24.21, 22.61, 20.98, 20.65, 7.06, 6.93, 4.07, 3.88.
^{1}H NMR (400 MHz, DMSO-*d*$_6$, 25 °C): δ 9.79 – 9.61 (m, 1H), 8.44 – 8.19 (m, 2H), 8.19 – 7.98 (m, 1H), 7.58 (d, *J* = 5.5 Hz, 1H), 7.36 – 7.28 (m, 6H), 7.18 – 6.78 (m, 4H), 6.74 – 6.26 (m, 2H), 4.99 – 4.96 (m, 2H), 4.27 – 4.15 (m, 1H), 4.10 – 4.01 (m, 1H), 2.97 (dd, *J* = 12.8, 6.6 Hz, 3H), 1.66 – 1.50 (m, 5H), 1.40 – 1.38 (m, 9H), 0.89 – 0.77 (m, 15H), 0.68 – 0.55 (m, 6H).
^{13}C NMR (151 MHz, DMSO-*d*$_6$, 25 °C): δ 174.17, 170.54, 164.04, 156.69, 152.24, 140.92, 138.76, 137.69, 137.65, 134.75, 133.25, 129.09, 128.91, 128.34, 128.24, 128.21, 126.82, 123.49, 119.90, 109.82, 80.63, 65.67, 54.45, 54.33, 29.40, 28.38, 26.67, 24.81, 23.25, 21.21, 8.01, 7.91, 4.58.
^{1}H NMR (400 MHz, DMSO-*d*$_6$, 60 °C): δ 9.53 (s, 1H), 8.32 (d, *J* = 10.0 Hz, 1H), 8.23 (d, *J* = 8.1 Hz, 1H), 8.15 – 8.01 (m, 1H), 7.55 – 7.41 (m, 1H), 7.32 (p, *J* = 7.4, 6.9 Hz, 6H), 7.13 (t, *J* = 7.8 Hz, 1H), 7.07 – 6.94 (m, 2H), 6.84 (d, *J* = 7.6 Hz, 1H), 6.69 (dd, *J* = 11.4, 3.5 Hz, 1H), 6.48 – 6.26 (m, 1H), 4.99 (d, *J* = 3.2 Hz, 2H), 4.39 – 4.17 (m, 1H), 4.10 (dt, *J* = 10.8, 5.3 Hz, 1H), 3.08 – 2.86 (m, 3H), 1.68 – 1.48 (m, 5H), 1.41 (s, 9H), 0.91 – 0.78 (m, 15H), 0.70 – 0.53 (m, 6H).
^{13}C NMR (101 MHz, DMSO-*d*$_6$, 60 °C): δ 173.19, 169.78, 163.34, 151.65, 140.33, 138.13, 137.83, 137.06, 134.26, 132.72, 128.32, 128.13, 127.52, 127.36, 126.02, 122.68, 119.33, 109.33, 79.96, 65.05, 53.83, 53.71, 28.82, 27.77, 25.94, 24.20, 22.43, 20.66, 7.18, 4.01.
HRMS (ESI-TOF): calculated for $C_{41}H_{59}N_5NaO_7Si^+$ [M+Na]$^+$: 784.4076, found: 784.4086.
TLC: R_f = 0.6 (1:1 hexane:EtOAc, $Ce_2(SO_4)_3$ in phosphomolybdic acid).
$[\alpha]^{25}_D$: -82.2 (*c* = 0.1, $CHCl_3$)

LCMS analysis of compound 96 after separation (negative scan):

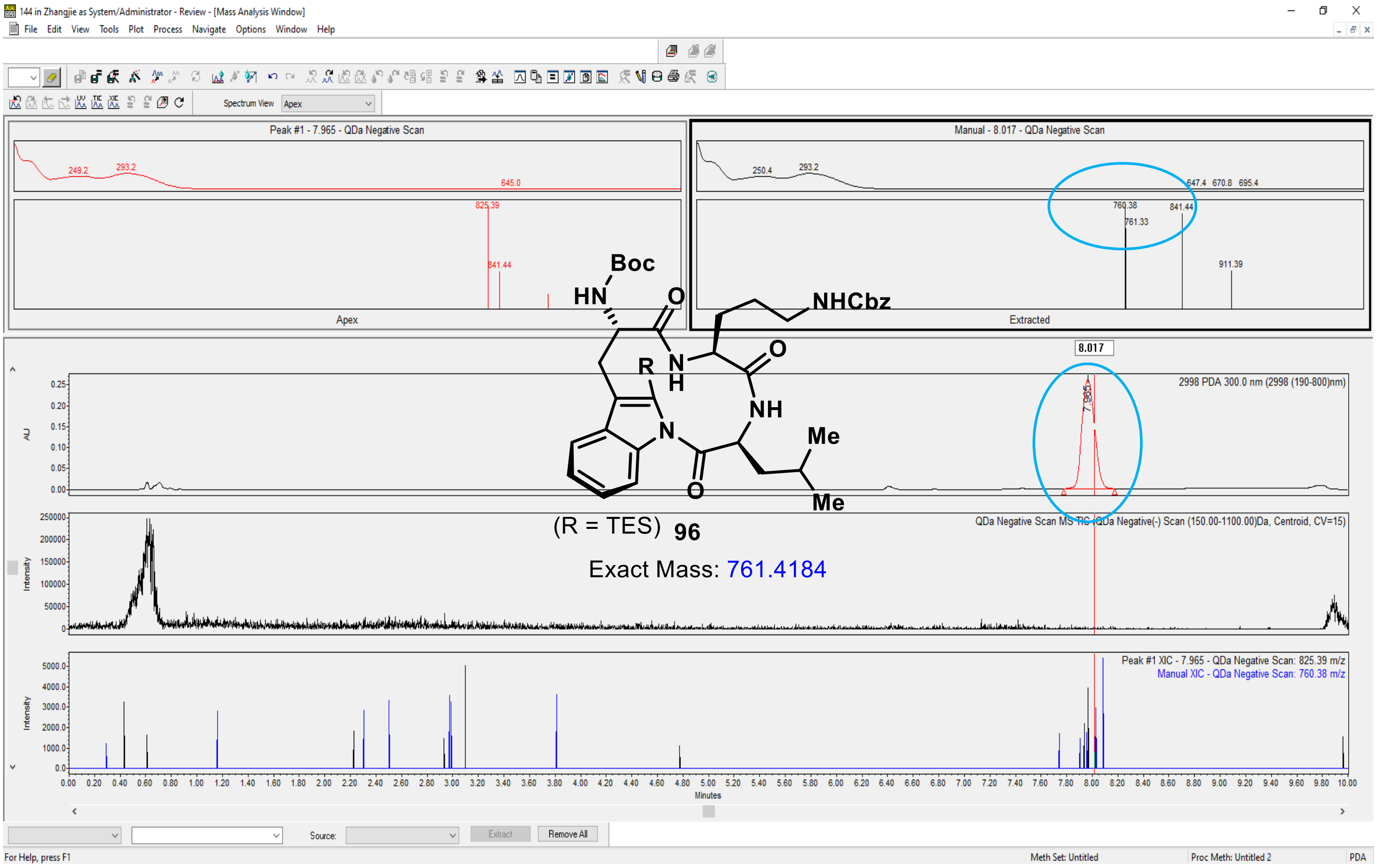

HRMS analysis of compound 96 after separation:

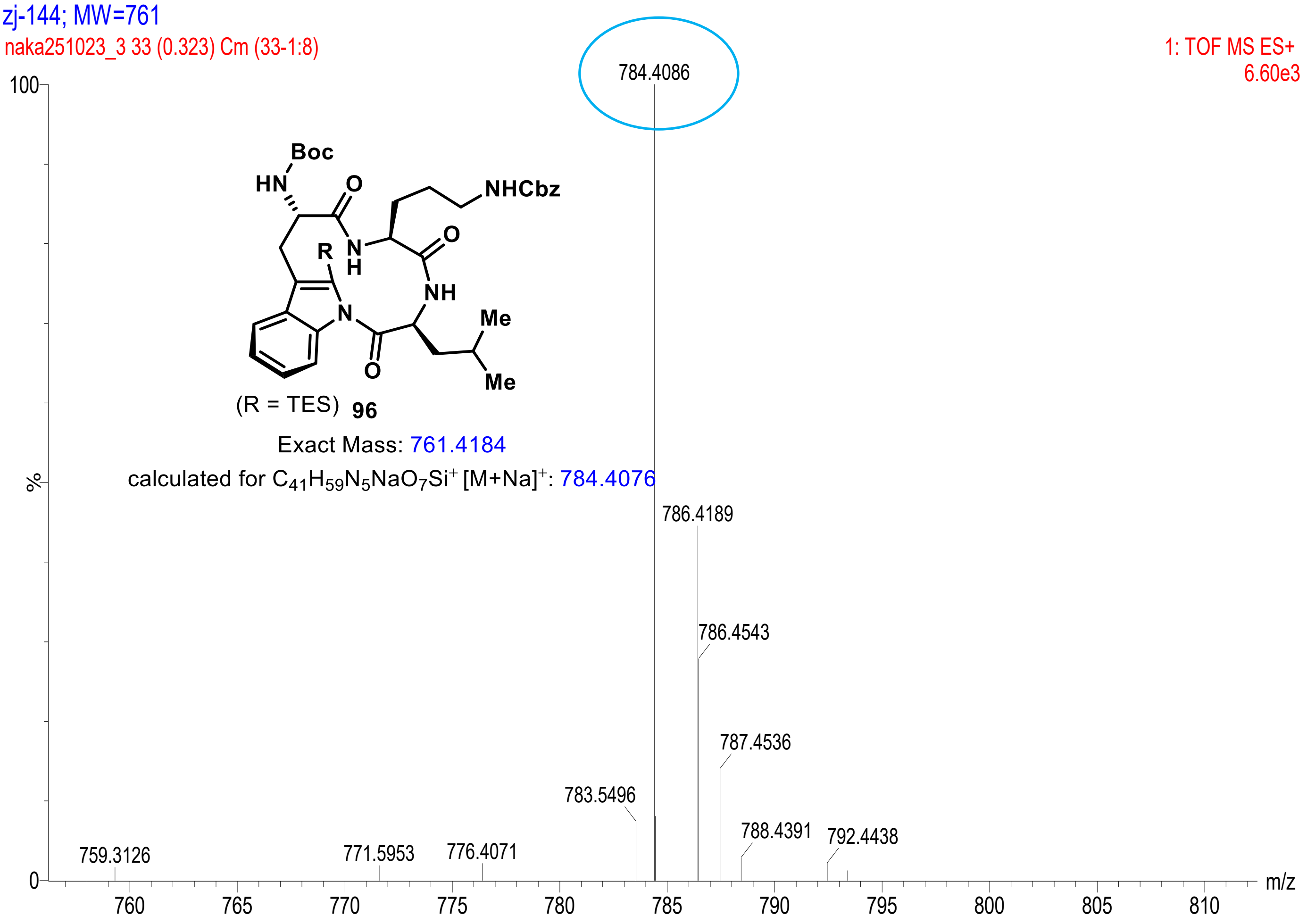

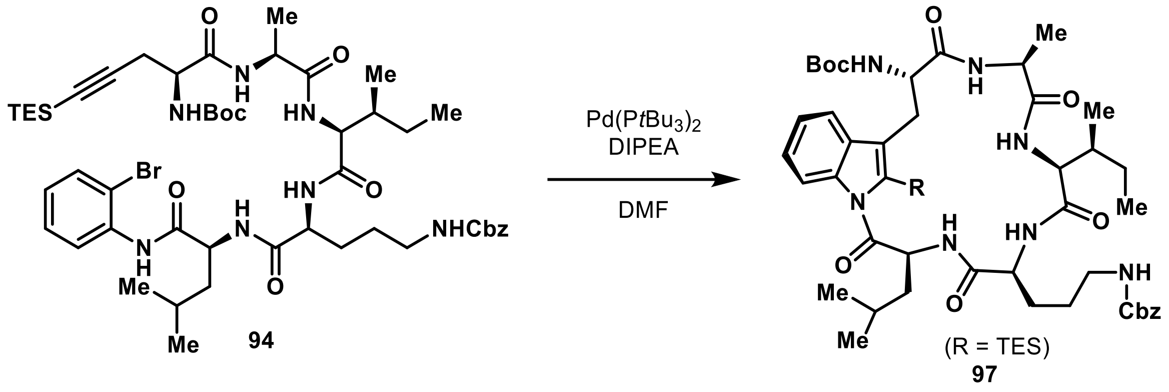

94 → **97**

To a solution of compound **94** (400 mg, 0.389 mmol, 1.0 equiv.) in 10 mL anhydrous DMF was added DIPEA (0.18 mL, 1.0 mmol, 3.0 equiv.) and Pd(tBu$_3$P)$_2$ (20 mg, 0.04 mmol, 0.1 equiv.). The reaction mixture was stirred at 100 °C under nitrogen atmosphere for 12 h. After the consumption of the starting material, the mixture was diluted with 60 mL EtOAc, washed with 0.5 M HCl and saturated *aq.* NaCl. The organic layer was dried over Na_2SO_4 and removed under reduced pressure to give the residue; the residue was purified by silica gel chromatography (50% EtOAc/hexane) to give the compound **97** (140 mg, 38% yield).

Compound 97
Physical State: amorphous solid
^{1}H NMR (400 MHz, DMSO-*d*$_6$): δ 8.25 (d, *J* = 7.2 Hz, 1H), 7.73 – 7.63 (m, 1H), 7.44 (t, *J* = 5.7 Hz, 2H), 7.40 – 7.29 (m, 7H), 7.15 – 6.89 (m, 4H), 5.02 (s, 2H), 4.30 (ddd, *J* = 11.2, 7.6, 2.9 Hz, 1H), 4.13 (td, *J* = 16.5, 14.3, 9.1 Hz, 2H), 3.73 (t, *J* = 5.5 Hz, 1H), 3.57 – 3.46 (m, 1H), 3.30 (d, *J* = 12.4 Hz, 1H), 3.04 – 2.91 (m, 1H), 2.62 (q, *J* = 6.8 Hz, 2H), 2.46 – 2.31 (m, 1H), 1.86 (q, *J* = 6.6, 5.4 Hz, 1H), 1.59 (ddd, *J* = 10.3, 8.1, 4.8 Hz, 1H), 1.47 (s, 9H), 1.30 (d, *J* = 7.1 Hz, 4H), 1.21 – 1.14 (m, 1H), 1.02 – 0.83 (m, 24H), 0.79 (dt, *J* = 7.4, 3.9 Hz, 6H), 0.60 – 0.49 (m, 1H), -0.06 – -0.21 (m, 1H).
^{13}C NMR (151 MHz, DMSO-*d*$_6$): δ 176.19, 173.34, 172.03, 170.89, 170.77, 156.43, 155.80, 139.98, 137.82, 133.65, 129.73, 129.05, 128.88, 128.32, 128.30, 124.30, 121.77, 119.15, 113.13, 79.72, 79.14, 65.65, 59.90, 57.12, 56.28, 53.51, 50.99, 40.58, 36.77, 35.50, 30.46, 28.72, 27.43, 26.88, 25.42, 24.21, 23.76, 21.54, 18.06, 16.15, 11.96, 8.13, 4.99.
HRMS (ESI-TOF): calculated for $C_{50}H_{75}N_7NaO_9Si^+$ $[M+Na]^+$: 968.5288, found: 968.5295.
TLC: R$_f$ = 0.4 (1:1 Hexane:EtOAc, $Ce_2(SO_4)_3$ in phosphomolybdic acid).
[α]$^{25}_D$: –117.4 (*c* = 1.0, $CHCl_3$)

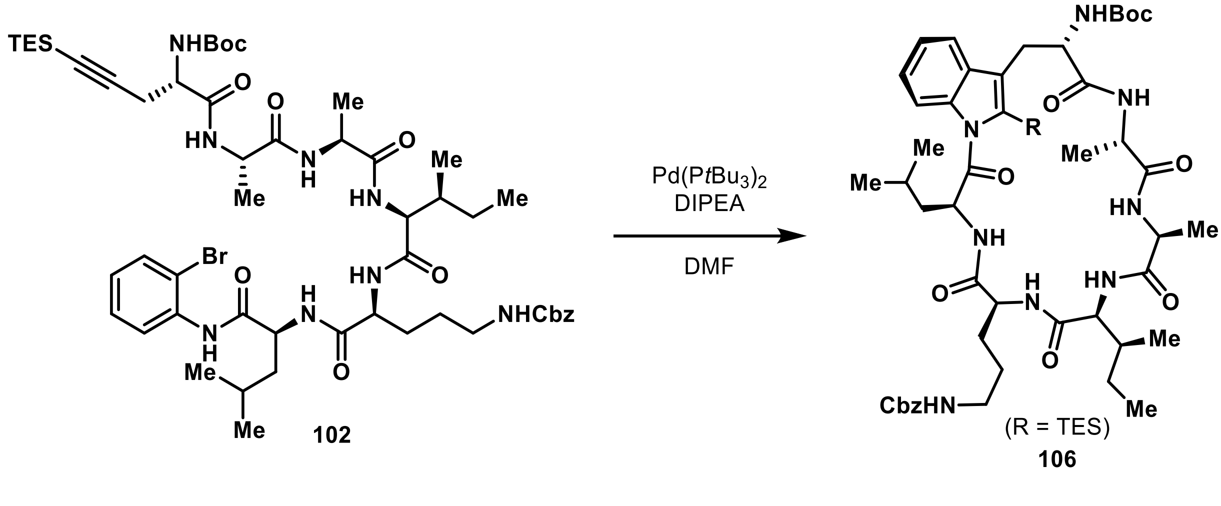

102 → **106**

To a solution of compound **102** (318 mg, 0.289 mmol, 1.0 equiv.) in 10 mL anhydrous DMF was added DIPEA (0.16 mL, 0.9 mmol, 3.0 equiv.) and Pd(*t*$Bu_3P)_2$ (15 mg, 0.03 mmol, 0.1 equiv.). The reaction mixture was stirred at 100 °C under nitrogen atmosphere for 12 h. After the consumption of the starting material, the mixture was diluted with 45 mL EtOAc, washed with 0.5 M HCl and saturated *aq.* NaCl. The organic layer was dried over Na_2SO_4 and removed under reduced pressure to give the residue; the residue was purified by silica gel chromatography (60% EtOAc/hexane) to give the compound **106** (100 mg, 34% yield).

Compound 106
Physical State: amorphous solid
^{1}H NMR (400 MHz, DMSO-*d*$_6$): δ 8.66 (s, 1H), 7.93 (d, *J* = 7.3 Hz, 1H), 7.88 (s, 1H), 7.43 – 7.24 (m, 10H), 7.09 (q, *J* = 7.4, 6.3 Hz, 2H), 7.00 (d, *J* = 7.7 Hz, 1H), 4.98 (s, 2H), 4.48 (s, 1H), 4.12 (s, 2H), 3.88 – 3.75 (m, 2H), 3.68 (s, 1H), 3.42 (t, *J* = 12.5 Hz, 1H), 2.97 (s, 1H), 2.85 (d, *J* = 12.8 Hz, 1H), 2.36 (t, *J* = 11.5 Hz, 2H), 1.73 (s, 2H), 1.43 (s, 9H), 1.37 – 1.32 (m, 4H), 1.14 – 1.12 (m, 1H), 1.04 – 0.96 (m, 3H), 0.92 (dd, *J* = 6.5, 4.0 Hz, 6H), 0.87 (dd, *J* = 13.3, 5.8 Hz, 15H), 0.80 – 0.74 (m, 6H), 0.71 (d, *J* = 6.8 Hz, 3H), 0.56 – 0.48 (m, 1H), 0.34 (s, 1H).
^{13}C NMR (151 MHz, DMSO-*d*$_6$): δ 176.22, 175.23, 174.03, 172.78, 170.18, 156.58, 155.91, 139.50, 137.76, 129.48, 128.84, 128.26, 128.13, 123.95, 121.52, 119.69, 113.52, 79.72, 78.76, 65.60, 59.00, 56.96, 54.61, 53.96, 53.03, 49.14, 40.58, 37.35, 35.95, 30.12, 29.12, 28.79, 26.50, 24.98, 24.59, 23.36, 22.50, 17.27, 16.55, 15.87, 12.45, 8.57, 8.13, 5.25.
HRMS (ESI-TOF): calculated for $C_{53}H_{80}N_8NaO_{10}Si^+$ $[M+Na]^+$: 1039.5659, found: 1039.5636.
TLC: R$_f$ = 0.3 (1:2 Hexane:EtOAc, $Ce_2(SO_4)_3$ in phosphomolybdic acid).
$[\alpha]^{25}_D$: –104.9 (*c* = 1.0, $CHCl_3$)

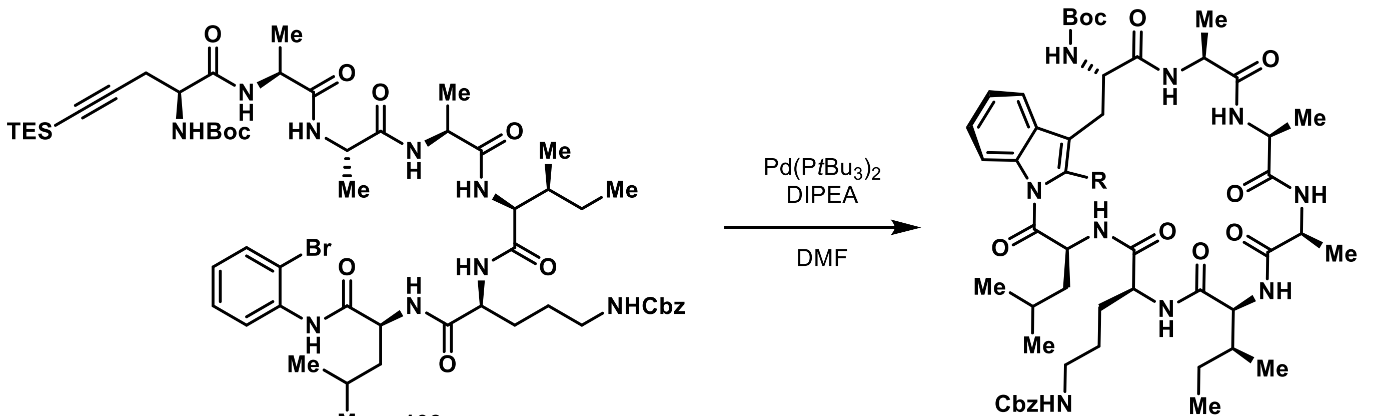

103

(R = TES)
107

To a solution of compound **103** (315 mg, 0.270 mmol, 1.0 equiv.) in 10 mL anhydrous DMF was added DIPEA (0.16 mL, 0.9 mmol, 3.0 equiv.) and Pd(*t*$Bu_3P)_2$ (15 mg, 0.03 mmol, 0.1 equiv.). The reaction mixture was stirred at 100 °C under nitrogen atmosphere for 12 h. After the consumption of the starting material, the mixture was diluted with 30 mL EtOAc, washed with 0.5 M HCl and saturated *aq.* NaCl. The organic layer was dried over Na_2SO_4 and removed under reduced pressure to give the residue; the residue was purified by silica gel chromatography (3% Methanol/EtOAc) to give the compound **107** (78 mg, 27% yield).

Compound 107
Physical State: amorphous solid
^{1}H NMR (600 MHz, DMSO-*d*$_6$): δ 8.47 (d, *J* = 5.4 Hz, 1H), 7.82 (d, *J* = 8.6 Hz, 1H), 7.64 (d, *J* = 4.8 Hz, 2H), 7.54 (d, *J* = 8.5 Hz, 1H), 7.45 (t, *J* = 9.2 Hz, 2H), 7.40 – 7.34 (m, 6H), 7.34 – 7.29 (m, 2H), 7.21 (t, *J* = 7.5

Hz, 1H), 7.03 (d, J = 8.6 Hz, 1H), 5.18 (s, 1H), 5.07 – 4.98 (m, 2H), 4.35 (d, J = 11.0 Hz, 1H), 4.21 (t, J = 7.8 Hz, 1H), 4.14 (p, J = 7.3 Hz, 1H), 3.91 (t, J = 7.7 Hz, 1H), 3.80 (dt, J = 10.9, 5.3 Hz, 1H), 3.19 – 2.91 (m, 4H), 2.03 (s, 1H), 1.98 – 1.88 (m, 2H), 1.83 (d, J = 11.5 Hz, 1H), 1.77 (t, J = 11.9 Hz, 1H), 1.49 (m, 1H), 1.44 (s, 9H), 1.36 (m, 1H), 1.25 – 1.21 (m, 4H), 1.18 – 1.14 (m, 6H), 1.09 (d, J = 6.5 Hz, 3H), 0.99 (d, J = 6.5 Hz, 3H), 0.91 (t, J = 7.7 Hz, 9H), 0.81 (q, J = 7.8, 7.3 Hz, 15H).
^{13}C NMR (151 MHz, DMSO-d_6): δ 173.25, 172.26, 171.93, 171.81, 171.34, 171.30, 156.75, 156.29, 137.73, 136.73, 136.14, 134.01, 131.22, 129.43, 128.87, 128.73, 128.30, 128.25, 127.75, 125.35, 122.73, 119.36, 114.54, 79.48, 65.72, 59.51, 59.08, 52.50, 51.26, 49.92, 49.30, 48.08, 40.58, 38.72, 35.67, 30.55, 29.15, 28.67, 26.97, 26.41, 26.27, 25.23, 24.93, 23.91, 21.16, 18.57, 18.13, 18.04, 16.17, 11.66, 8.91, 5.89.
HRMS (ESI-TOF): calculated for $C_{56}H_{85}N_9NaO_{11}Si^+$ [M+Na]$^+$: 1110.6030, found: 1110.6028.
TLC: R$_f$ = 0.5 (20:1 EtOAc:Methanol, $Ce_2(SO_4)_3$ in phosphomolybdic acid).
[α]$^{25}_D$: –99.4 (c = 1.0, $CHCl_3$)

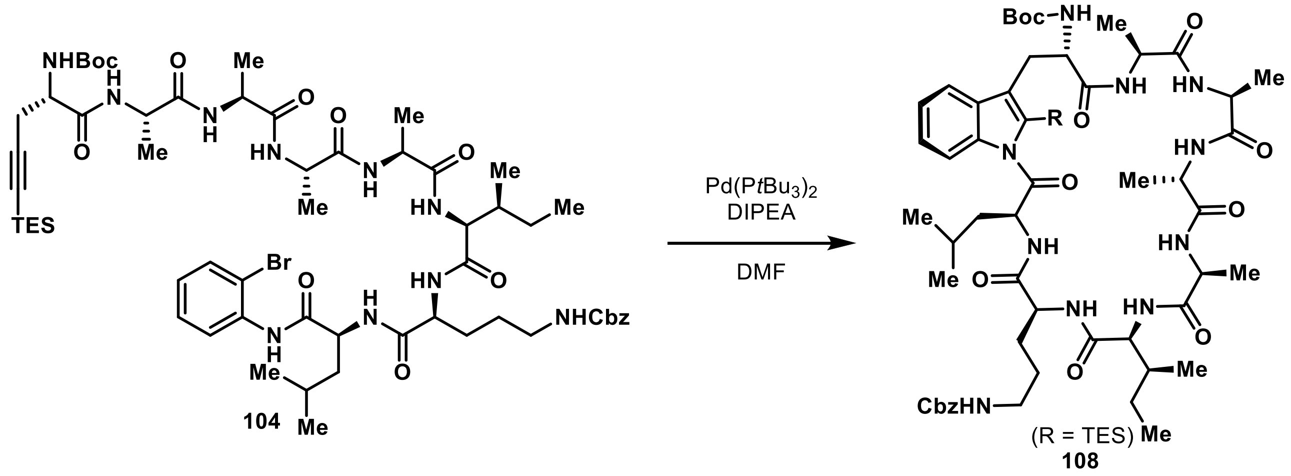

To a solution of compound **104** (290 mg, 0.235 mmol, 1.0 equiv.) in 10 mL anhydrous DMF was added DIPEA (0.13 mL, 0.7 mmol, 3.0 equiv.) and Pd(tBu$_3$P)$_2$ (15 mg, 0.3 mmol, 0.1 equiv.). The reaction mixture was stirred at 100 °C under nitrogen atmosphere for 12 h. After the consumption of the starting material, the mixture was diluted with 30 mL EtOAc, washed with 0.5 M HCl and saturated *aq.* NaCl. The organic layer was dried over Na_2SO_4 and removed under reduced pressure to give the residue; the residue was purified by silica gel chromatography (4% Methanol/EtOAc) to give the compound **108** (65 mg, 24% yield).

Compound 108
Physical State: amorphous solid
^{1}H NMR (400 MHz, DMSO-d_6): δ 8.50 (d, J = 6.8 Hz, 1H), 7.99 – 7.88 (m, 2H), 7.69 (s, 1H), 7.63 – 7.54 (m, 2H), 7.54 – 7.45 (m, 2H), 7.37 – 7.30 (m, 7H), 7.28 – 7.19 (m, 3H), 5.62 – 5.24 (m, 1H), 5.00 (s, 2H), 4.36 (s, 1H), 4.13 – 3.96 (m, 4H), 3.78 (s, 1H), 3.30 (s, 1H), 3.22 – 3.08 (m, 2H), 3.06 – 2.96 (m, 2H), 2.02 – 1.87 (m, 2H), 1.83 – 1.70 (m, 3H), 1.48 (s, 2H), 1.42 (s, 9H), 1.37 – 1.31 (m, 2H), 1.21 – 1.20 (m, 2H), 1.19 – 1.17 (m, 2H), 1.14 – 1.09 (m, 6H), 1.06 (d, J = 6.3 Hz, 3H), 0.95 (d, J = 6.5 Hz, 3H), 0.92 – 0.84 (m, 12H), 0.83 – 0.73 (m, 12H).
^{13}C NMR (151 MHz, DMSO-d_6): δ 173.40, 173.21, 172.25, 172.16, 171.93, 171.54, 170.61, 162.85, 156.68, 156.32, 137.72, 136.43, 135.93, 133.91, 131.41, 128.87, 128.29, 128.23, 125.59, 122.85, 119.55, 114.25, 79.72, 79.63, 65.71, 59.76, 58.28, 52.16, 51.88, 50.41, 50.01, 48.91, 48.60, 40.58, 36.70, 36.31, 31.30, 30.82, 29.16, 28.67, 26.90, 26.29, 25.06, 24.72, 23.76, 21.39, 18.66, 18.11, 17.93, 16.14, 11.64, 8.89, 5.85.
HRMS (ESI-TOF): calculated for $C_{59}H_{90}N_{10}NaO_{12}Si^+$ [M+Na]$^+$: 1181.6401, found: 1181.6404.
TLC: R$_f$ = 0.4 (20:1 EtOAc:Methanol, $Ce_2(SO_4)_3$ in phosphomolybdic acid).

$[\alpha]^{25}_{D}$: –97.7 (c = 1.0, $CHCl_3$)

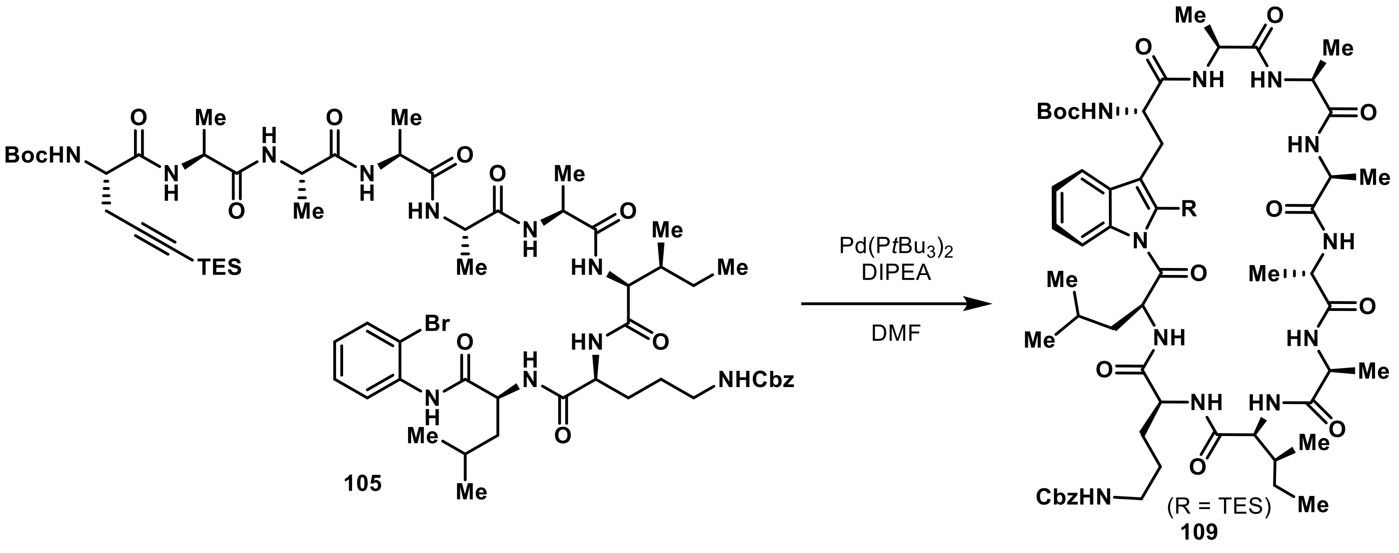

To a solution of compound **105** (178 mg, 0.135 mmol, 1.0 equiv.) in 8 mL anhydrous DMF was added DIPEA (0.07 mL, 0.4 mmol, 3.0 equiv.) and Pd($t$$Bu_3P)_2$ (7 mg, 0.14 mmol, 0.1 equiv.). The reaction mixture was stirred at 100 °C under nitrogen atmosphere for 12 h. After the consumption of the starting material, the mixture was diluted with 20 mL EtOAc, washed with 0.5 M HCl and saturated *aq.* NaCl. The organic layer was dried over Na_2SO_4 and removed under reduced pressure to give the residue; the residue was purified by silica gel chromatography (6% Methanol/EtOAc) to give the compound **109** (30 mg, 18% yield).

Compound 109

Physical State: amorphous solid

^{1}H NMR (400 MHz, DMSO-*d*$_6$): δ 8.54 (d, J = 6.1 Hz, 1H), 8.26 (s, 1H), 7.95 – 7.91 (m, 1H), 7.82 (d, J = 9.7 Hz, 1H), 7.70 (d, J = 7.4 Hz, 2H), 7.60 – 7.52 (m, 2H), 7.50 – 7.45 (m, 1H), 7.36 – 7.30 (m, 7H), 7.25 – 7.18 (m, 2H), 7.11 (s, 1H), 5.42 – 5.35 (m, 1H), 5.04 – 4.96 (m, 2H), 4.48 – 4.39 (m, 1H), 4.24 (t, J = 7.7 Hz, 2H), 4.16 – 3.99 (m, 4H), 3.95 – 3.79 (m, 2H), 3.23 – 3.11 (m, 2H), 3.07 – 2.94 (m, 3H), 1.89 – 1.76 (m, 3H), 1.73 – 1.67 (m, 1H), 1.58 (d, J = 10.1 Hz, 2H), 1.43 (s, 9H), 1.23 – 1.22 (m, 2H),1.20 – 1.19 (m, 3H), 1.06 (d, J = 7.2 Hz, 3H), 0.97 (d, J = 6.5 Hz, 3H), 0.92 – 0.82 (m, 18H), 0.81 – 0.72 (m, 12H), 0.65 (d, J = 6.8 Hz, 3H).

^{13}C NMR (151 MHz, DMSO-*d*$_6$): δ 179.79, 173.68, 172.71, 172.67, 172.45, 172.11, 171.95, 170.69, 156.67, 156.33, 137.73, 136.19, 136.02, 133.57, 130.84, 128.87, 128.29, 128.23, 125.54, 122.72, 119.63, 114.43, 79.72, 65.70, 58.23, 52.48, 51.73, 50.65, 49.90, 49.27, 48.40, 40.58, 36.86, 30.83, 28.66, 28.66, 25.84, 24.97, 24.83, 23.69, 23.69, 21.56, 18.56, 18.21, 17.93, 17.68, 16.73, 16.13, 11.57, 8.77, 5.63.

HRMS (ESI-TOF): calculated for $C_{62}H_{95}N_{11}NaO_{13}Si^+$ $[M+Na]^+$: 1252.6778, found: 1252.6770.

TLC: R_f = 0.5 (15:1 EtOAc:Methanol, $Ce_2(SO_4)_3$ in phosphomolybdic acid).

$[\alpha]^{25}_{D}$: –112.1 (c = 1.0, $CHCl_3$)

Synthesis of cyclization precursors

Synthesis of precursors 92–94 and 102–105

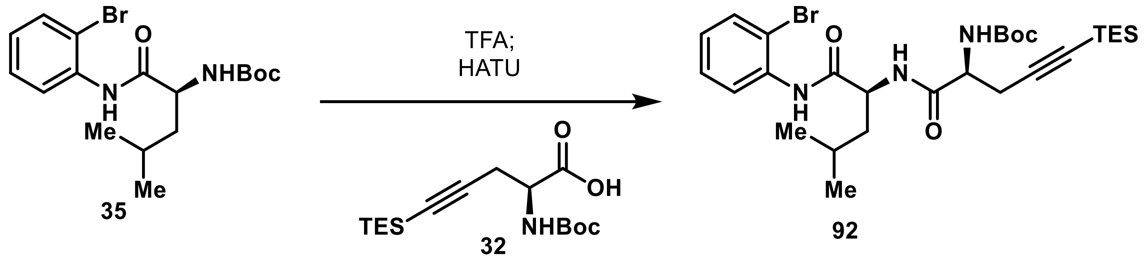

Compound **35** (307 mg, 0.8 mmol) was dissolved in DCM (3 mL), TFA (1.5 mL) was added dropwise, the reaction was stirred at rt for 1 h. Then 10 mL toluene was added and concentrated *in vacuo* (three times with toluene) to give the residue. The residue was used in the next step directly without further purification.

To a solution of the crude, compound **32** (314 mg, 0.96 mmol, 1.2 equiv.) and DIPEA (0.56 mL, 3.2 mmol, 4.0 equiv.) in DMF (8 mL) was added HATU (365 mg, 0.96 mmol, 1.2 equiv.) in portion wise. The reaction mixture was stirred at rt for 1 h then quenched by 0.5 M HCl (10 mL) and extracted with EtOAc (10 mL) three times. The organic layers were combined and washed with saturated *aq.* NaCl, dried over Na_2SO_4 and removed under reduced pressure to give the residue; the residue was purified by silica gel chromatography (10% EtOAc/hexane) to give the compound **92** (430 mg, 90% yield over 2 steps).

Compound 92

Physical State: white solid

^{1}H NMR (600 MHz, $CDCl_3$): δ 8.32 (s, 1H), 8.24 (d, *J* = 8.2 Hz, 1H), 7.51 (dt, *J* = 8.0, 1.3 Hz, 1H), 7.27 (ddd, *J* = 8.5, 7.4, 1.5 Hz, 1H), 7.06 – 6.86 (m, 2H), 5.30 (s, 1H), 4.60 (td, *J* = 8.6, 5.9 Hz, 1H), 4.36 – 4.14 (m, 1H), 2.89 (ddd, *J* = 17.2, 6.0, 1.5 Hz, 1H), 2.68 (dd, *J* = 17.2, 6.8 Hz, 1H), 1.85 (ddd, *J* = 13.9, 8.4, 5.8 Hz, 1H), 1.69 (dq, *J* = 8.2, 6.3 Hz, 1H), 1.63 (ddd, *J* = 14.4, 9.1, 5.8 Hz, 1H), 1.43 (s, 9H), 0.98 – 0.91 (m, 15H), 0.56 – 0.51 (m, 6H).

^{13}C NMR (151 MHz, $CDCl_3$): δ 170.98, 169.76, 155.82, 135.52, 132.42, 128.33, 125.61, 122.41, 114.09, 102.61, 86.30, 80.86, 52.95, 40.59, 28.30, 24.87, 23.15, 23.02, 21.99, 7.55, 4.37.

HRMS (ESI-TOF): calculated for $C_{28}H_{44}BrN_3NaO_4Si^+$ $[M+Na]^+$: 616.2177, found: 616.2181.

TLC: R_f = 0.6 (4:1 Hexane:EtOAc, $Ce_2(SO_4)_3$ in phosphomolybdic acid).

$[\alpha]^{25}_D$: –37.8 (*c* = 1.0, $CHCl_3$)

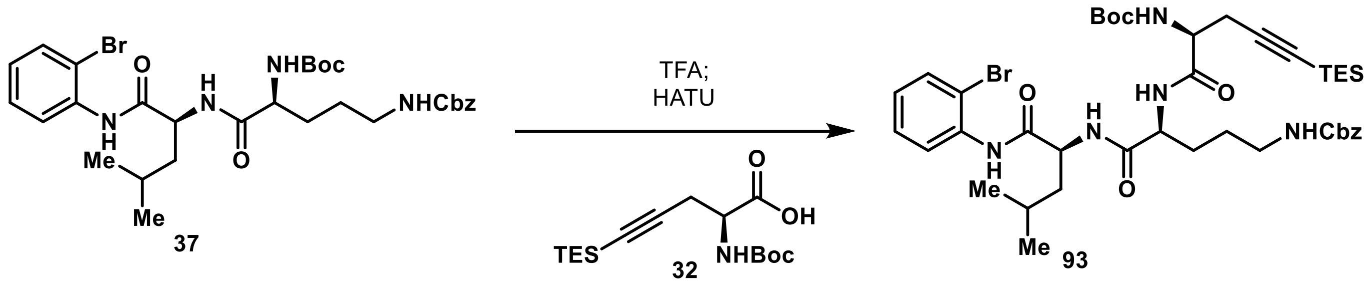

Compound **37** (634 mg, 0.6 mmol) was dissolved in DCM (3 mL), TFA (1.5 mL) was added dropwise, the reaction was stirred at rt for 1 h. Then 10 mL toluene was added and concentrated *in vacuo* (three times with toluene) to give the residue. The residue was used in the next step directly without further purification.

To a solution of the crude, compound **32** (236 mg, 0.72 mmol, 1.2 equiv.) and DIPEA (0.42 mL, 2.4 mmol, 4.0 equiv.) in DMF (6 mL) was added HATU (274 mg, 0.72 mmol, 1.2 equiv.) in portion wise. The reaction mixture was stirred at rt for 1 h then quenched by 0.5 M HCl (10 mL) and extracted with EtOAc (10 mL) three times. The organic layers were combined and washed with saturated *aq.* NaCl, dried over Na_2SO_4 and removed under reduced pressure to give the residue; the residue was purified by silica gel chromatography (30% EtOAc/hexane) to give the compound **93** (375 mg, 73% yield over 2 steps).

Compound 93
Physical State: white solid
^{1}H NMR (600 MHz, $CDCl_3$): δ 8.30 (s, 1H), 8.19 (d, *J* = 8.2 Hz, 1H), 7.50 (dd, *J* = 8.0, 1.4 Hz, 1H), 7.31 (d, *J* = 4.4 Hz, 5H), 7.29 – 7.26 (m, 1H), 7.16 (d, *J* = 7.9 Hz, 1H), 6.95 (td, *J* = 7.7, 1.6 Hz, 1H), 5.31 (s, 1H), 5.10 (d, *J* = 12.2 Hz, 1H), 5.08 – 5.00 (m, 2H), 4.71 (s, 1H), 4.58 – 4.51 (m, 1H), 4.26 (s, 1H), 3.49 (s, 1H), 3.17 – 3.04 (m, 1H), 2.82 (dd, *J* = 17.1, 6.3 Hz, 1H), 2.68 (dd, *J* = 17.2, 5.8 Hz, 1H), 1.96 – 1.88 (m, 1H), 1.84 – 1.78 (m, 2H), 1.78 – 1.70 (m, 1H), 1.69 – 1.53 (m, 3H), 1.51 – 1.46 (m, 1H), 1.43 (s, 9H), 0.98 – 0.91 (m, 15H), 0.55 (q, *J* = 7.9 Hz, 6H).
^{13}C NMR (151 MHz, $CDCl_3$): δ 172.08, 170.86, 170.37, 157.48, 155.74, 136.42, 135.63, 132.47, 128.62, 128.33, 128.25, 128.20, 125.68, 122.86, 114.32, 100.80, 86.15, 80.79, 67.01, 53.37, 53.03, 51.73, 40.49, 39.41, 30.28, 28.32, 26.58, 24.89, 23.49, 23.04, 22.01, 7.58, 4.41.
HRMS (ESI-TOF): calculated for $C_{41}H_{60}BrN_5NaO_7Si^+$ [M+Na]$^+$: 864.3338, found: 864.3332.
TLC: R_f = 0.4 (2:1 Hexane:EtOAc, $Ce_2(SO_4)_3$ in phosphomolybdic acid).
$[\alpha]^{25}_D$: –15.7 (*c* = 1.0, $CHCl_3$)

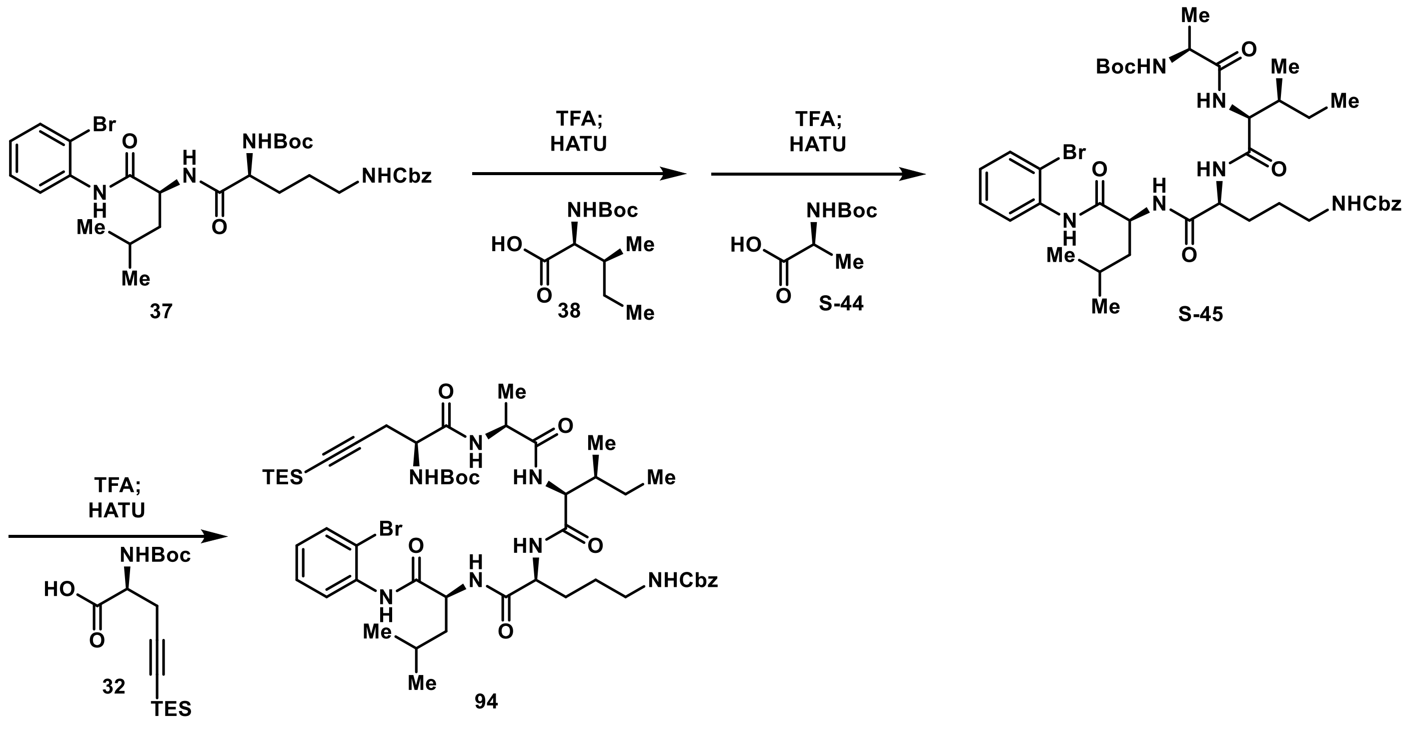

Compound **37** (634 mg, 1.0 mmol) was dissolved in DCM (3 mL), treated dropwise with TFA (1.5 mL), and stirred at room temperature for 1 h. The mixture co-evaporated with toluene (10 mL × 3) *in vacuo* to afford a residue, which was used directly in the next step without purification.
To a solution of the crude residue, Boc-*L*-isoleucine (278 mg, 1.2 mmol, 1.2 equiv.), and DIPEA (0.7 mL, 4.0 mmol, 4.0 equiv.) in DMF (10 mL) was added HATU (456 mg, 1.2 mmol, 1.2 equiv.) in one portion. After stirring at room temperature for 1 h, the reaction was quenched with 0.5 M HCl (20 mL) and extracted with

EtOAc (20 mL × 3). The combined organic layers were washed with saturated aqueous NaCl, dried over Na_2SO_4, and concentrated *in vacuo* to give a residue that was used directly in the subsequent step.

This residue was dissolved in DCM (3 mL), treated dropwise with TFA (1.5 mL), and stirred at room temperature for 1 h. The mixture was co-evaporated with toluene (10 mL × 3) *in vacuo* to afford a residue for direct use in the next step.
A mixture of the residue and Boc-*L*-alanine (260 mg, 1.2 mmol, 1.2 equiv.) in DMF (10 mL) was treated sequentially with DIPEA (0.7 mL, 4.0 mmol, 4.0 equiv.) and HATU (456 mg, 1.2 mmol, 1.2 equiv.). The reaction was stirred for 1 h, quenched with 0.5 M HCl (20 mL), and extracted with EtOAc (20 mL × 3). The combined organic extracts were washed with saturated aqueous NaCl, dried over Na_2SO_4, and concentrated *in vacuo* to give a residue **S-45**, which was used directly in the next step.

The residue **S-45** was dissolved in DCM (3 mL), treated dropwise with TFA (1.5 mL), and stirred at room temperature for 1 h. The mixture was co-evaporated with toluene (10 mL × 3) *in vacuo* to afford a residue.
To a solution of this crude residue, compound **32** (394 mg, 1.2 mmol, 1.2 equiv.), and DIPEA (0.7 mL, 4.0 mmol, 4.0 equiv.) in DMF (10 mL) was added HATU (456 mg, 1.2 mmol, 1.2 equiv.) in one portion. The reaction mixture was stirred at room temperature for 2.5 h, then water (30 mL) was added slowly to the reaction mixture to form a precipitate. The solid was filtered, washed successively with EtOH and EtOAc (twice each), and dried to yield the product **94** as a white solid (748 mg, 73% overall yield).

Compound 94
Physical State: white solid
^{1}H NMR (600 MHz, DMSO-*d*$_6$): δ 9.42 (s, 1H), 8.11 (d, J = 7.9 Hz, 1H), 8.00 – 7.87 (m, 2H), 7.79 (d, J = 8.7 Hz, 1H), 7.58 (dd, J = 17.6, 8.1 Hz, 2H), 7.35 – 7.16 (m, 7H), 7.08 (d, J = 7.8 Hz, 1H), 7.01 (d, J = 8.8 Hz, 1H), 4.95 (s, 2H), 4.49 (q, J = 8.0 Hz, 1H), 4.35 – 4.19 (m, 2H), 4.19 – 3.95 (m, 2H), 3.04 – 2.88 (m, 2H), 2.66 – 2.52 (m, 1H), 2.44 – 2.36 (m, 1H), 1.70 – 1.59 (m, 3H), 1.59 – 1.51 (m, 2H), 1.50 – 1.37 (m, 3H), 1.34 (s, 9H), 1.14 (d, J = 7.0 Hz, 4H), 1.05 – 0.97 (m, 1H), 0.96 – 0.85 (m, 12H), 0.83 (d, J = 6.4 Hz, 3H), 0.80 – 0.68 (m, 6H), 0.58 – 0.38 (m, 6H).
^{13}C NMR (151 MHz, DMSO-*d*$_6$): δ 172.32, 172.07, 171.52, 171.19, 170.49, 162.85, 156.66, 155.65, 137.74, 136.44, 133.19, 128.87, 128.58, 128.28, 127.52, 127.02, 118.09, 105.96, 83.09, 78.70, 65.69, 57.24, 53.88, 52.57, 52.03, 48.67, 41.16, 37.36, 36.31, 31.30, 29.86, 28.63, 26.48, 24.76, 24.69, 23.78, 23.64, 22.02, 18.76, 15.74, 11.62, 7.87, 4.48.
HRMS (ESI-TOF): calculated for $C_{50}H_{76}BrN_7NaO_9Si^+$ [M+Na]$^+$: 1048.4549, found: 1048.4541.
TLC: R_f = 0.4 (5:1 DCM: Acetone, $Ce_2(SO_4)_3$ in phosphomolybdic acid).
[α]$^{25}_D$: –20.1 (*c* = 1.0, $CHCl_3$)

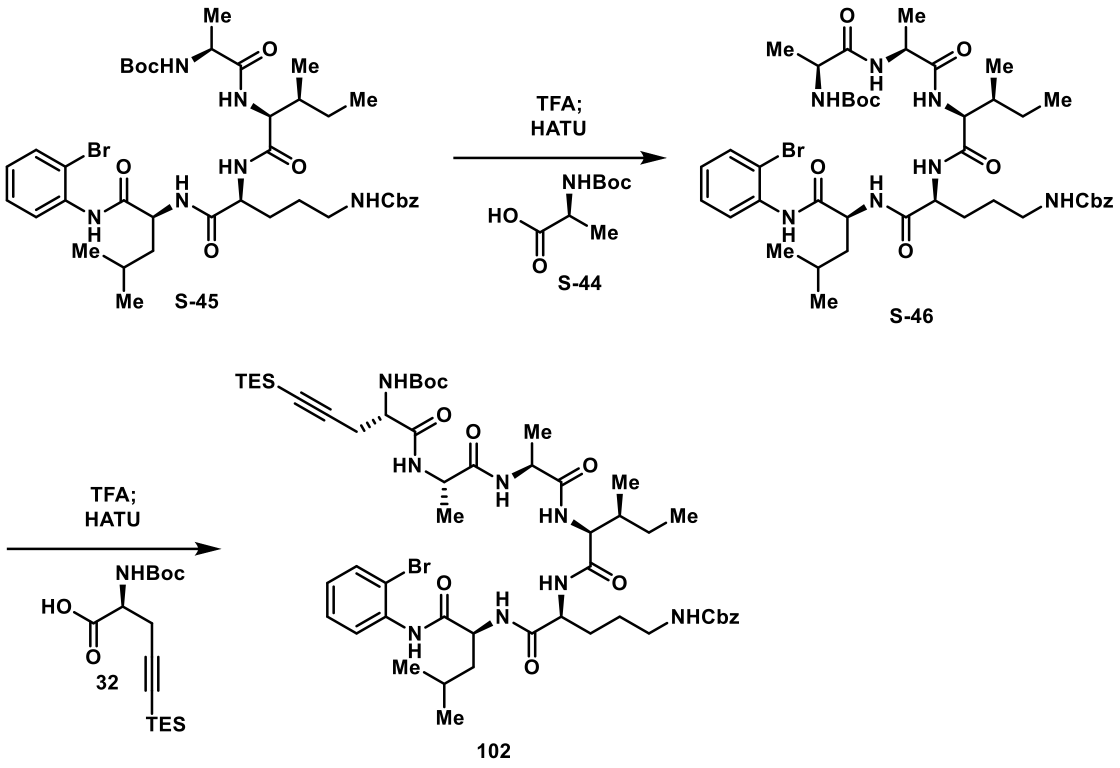

Crude product **S-45** (1.0 mmol) was dissolved in DCM (3 mL), treated dropwise with TFA (1.5 mL), and stirred at room temperature for 1 h. The mixture was co-evaporated with toluene (10 mL × 3) *in vacuo* to afford a residue for direct use in the next step.

A mixture of the residue and Boc-*L*-alanine (260 mg, 1.2 mmol, 1.2 equiv.) in DMF (10 mL) was treated sequentially with DIPEA (0.7 mL, 4.0 mmol, 4.0 equiv.) and HATU (456 mg, 1.2 mmol, 1.2 equiv.). The reaction was stirred for 1 h, then water (30 mL) was added slowly to the reaction mixture to form a precipitate. The solid was filtered, washed successively with EtOH and EtOAc (twice each), and dried to to give a residue **S-46**, which was used directly in the next step.

The residue was dissolved in DCM (3 mL), treated dropwise with TFA (1.5 mL), and stirred at room temperature for 1 h. The mixture was co-evaporated with toluene (10 mL × 3) *in vacuo* to afford a residue.

To a solution of this crude residue, compound **32** (394 mg, 1.2 mmol, 1.2 equiv.), and DIPEA (0.7 mL, 4.0 mmol, 4.0 equiv.) in DMF (10 mL) was added HATU (456 mg, 1.2 mmol, 1.2 equiv.) in one portion. The reaction mixture was stirred at room temperature for 2.5 h, then water (30 mL) was added slowly to the reaction mixture to form a precipitate. The solid was filtered, washed successively with EtOH and EtOAc (twice each), and dried to yield the product **102** as a white solid (707 mg, 64% overall yield).

Compound 102

Physical State: white solid

^{1}H NMR (600 MHz, DMSO-d_6): δ 9.42 (s, 1H), 8.09 (dd, *J* = 27.4, 7.7 Hz, 2H), 8.00 – 7.89 (m, 2H), 7.66 (d, *J* = 8.8 Hz, 1H), 7.58 (dd, *J* = 19.0, 8.0 Hz, 2H), 7.35 – 7.18 (m, 7H), 7.09 (t, *J* = 7.8 Hz, 1H), 6.99 (d, *J* = 8.7 Hz, 1H), 4.95 (s, 2H), 4.48 (q, *J* = 7.9 Hz, 1H), 4.34 – 4.05 (m, 5H), 3.01 – 2.89 (m, 2H), 2.58 (d, *J* = 16.2 Hz, 0H), 2.45 – 2.34 (m, 1H), 1.72 – 1.60 (m, 3H), 1.55 (t, *J* = 7.3 Hz, 2H), 1.51 – 1.40 (m, 3H), 1.34 (s, 9H), 1.20 – 1.09 (m, 7H), 1.05 – 0.96 (m, 1H), 0.94 – 0.85 (m, 12H), 0.85 – 0.79 (m, 3H), 0.80 – 0.69 (m, 6H), 0.48 (q, *J* = 8.0 Hz, 6H).

^{13}C NMR (151 MHz, DMSO-d_6): δ 172.35, 172.26, 172.07, 171.52, 171.17, 170.47, 162.85, 156.67, 155.66, 137.74, 136.44, 133.19, 128.86, 128.58, 128.27, 127.52, 127.01, 118.10, 105.93, 83.09, 78.73, 65.69, 57.16,

53.82, 52.59, 52.05, 48.62, 48.55, 41.15, 38.77, 37.49, 36.31, 31.30, 29.85, 28.64, 26.49, 24.69, 23.81, 23.63, 22.02, 18.88, 18.31, 15.73, 11.65, 7.87, 4.48.
HRMS (ESI-TOF): calculated for $C_{53}H_{81}BrN_8NaO_{10}Si^+$ $[M+Na]^+$: 1119.4921, found: 1119.4916.
TLC: $R_f = 0.5$ (15:1 DCM: Methanol, $Ce_2(SO_4)_3$ in phosphomolybdic acid).
$[\alpha]^{25}_D$: –11.4 (c = 1.0, $CHCl_3$)

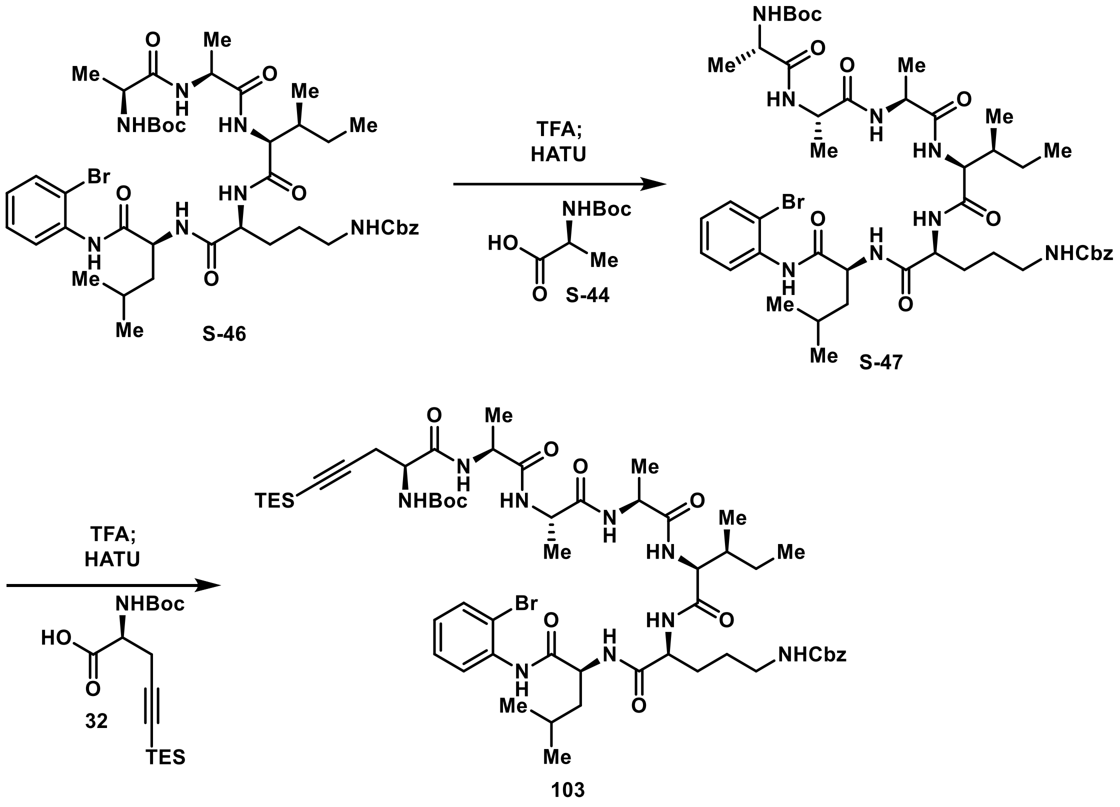

Crude product **S-46** (1.0 mmol) was dissolved in DCM (5 mL), treated dropwise with TFA (3 mL), and stirred at room temperature for 1 h. The mixture was co-evaporated with toluene (10 mL × 3) *in vacuo* to afford a residue for direct use in the next step.
A mixture of the residue and Boc-*L*-alanine (260 mg, 1.2 mmol, 1.2 equiv.) in DMF (15 mL) was treated sequentially with DIPEA (0.7 mL, 4.0 mmol, 4.0 equiv.) and HATU (456 mg, 1.2 mmol, 1.2 equiv.). The reaction was stirred for 2 h, then water (45 mL) was added slowly to the reaction mixture to form a precipitate. The solid was filtered, washed successively with EtOH and EtOAc (twice each), and dried to give a residue **S-47**, which was used directly in the next step.

The residue was dissolved in DCM (6 mL), treated dropwise with TFA (4 mL), and stirred at room temperature for 1 h. The mixture was co-evaporated with toluene (10 mL × 3) *in vacuo* to afford a residue.
To a solution of this crude residue, compound **32** (394 mg, 1.2 mmol, 1.2 equiv.), and DIPEA (0.7 mL, 4.0 mmol, 4.0 equiv.) in DMF (15 mL) was added HATU (456 mg, 1.2 mmol, 1.2 equiv.) in one portion. The reaction mixture was stirred at room temperature for 2.5 h, then water (45 mL) was added slowly to the reaction mixture to form a precipitate. The solid was filtered, washed successively with EtOH and EtOAc (twice each), and dried to yield the product **103** as a white solid (733 mg, 63% overall yield).

Compound 103
Physical State: white solid
^{1}H NMR (600 MHz, DMSO-d_6): δ 9.42 (s, 1H), 8.10 (d, J = 8.1 Hz, 1H), 8.03 (d, J = 7.2 Hz, 1H), 7.94 (dt, J = 19.9, 8.8 Hz, 3H), 7.68 (d, J = 8.6 Hz, 1H), 7.58 (dd, J = 23.6, 7.9 Hz, 2H), 7.43 – 7.16 (m, 7H), 7.09 (t, J

= 7.7 Hz, 1H), 6.99 (d, J = 8.6 Hz, 1H), 4.95 (s, 2H), 4.48 (q, J = 7.8 Hz, 1H), 4.37 – 4.03 (m, 6H), 3.06 – 2.83 (m, 2H), 2.59 (d, J = 17.0 Hz, 1H), 2.45 – 2.32 (m, 1H), 1.72 – 1.59 (m, 3H), 1.59 – 1.52 (m, 2H), 1.50 – 1.43 (m, 1H), 1.43 – 1.36 (m, 2H), 1.34 (s, 9H), 1.28 – 1.05 (m, 10H), 1.05 – 0.97 (m, 1H), 0.97 – 0.83 (m, 12H), 0.84 – 0.79 (m, 3H), 0.79 – 0.67 (m, 6H), 0.48 (q, J = 8.0 Hz, 6H).
^{13}C NMR (151 MHz, DMSO-d_6): δ 172.40, 172.33, 172.20, 172.07, 171.51, 171.19, 170.50, 156.67, 155.67, 137.73, 136.43, 133.20, 128.87, 128.58, 128.28, 127.54, 127.04, 118.13, 105.96, 83.09, 78.74, 65.70, 57.17, 53.82, 52.60, 52.04, 48.60, 48.50, 41.15, 37.43, 29.84, 28.64, 26.49, 24.69, 23.78, 23.64, 22.02, 18.82, 18.59, 18.43, 15.74, 11.64, 7.88, 4.48.
HRMS (ESI-TOF): calculated for $C_{56}H_{86}BrN_9NaO_{11}Si^+$ [M+Na]$^+$: 1190.5292, found: 1190.5295.
$[\alpha]^{25}_D$: –19.9 (c = 1.0, $CHCl_3$)

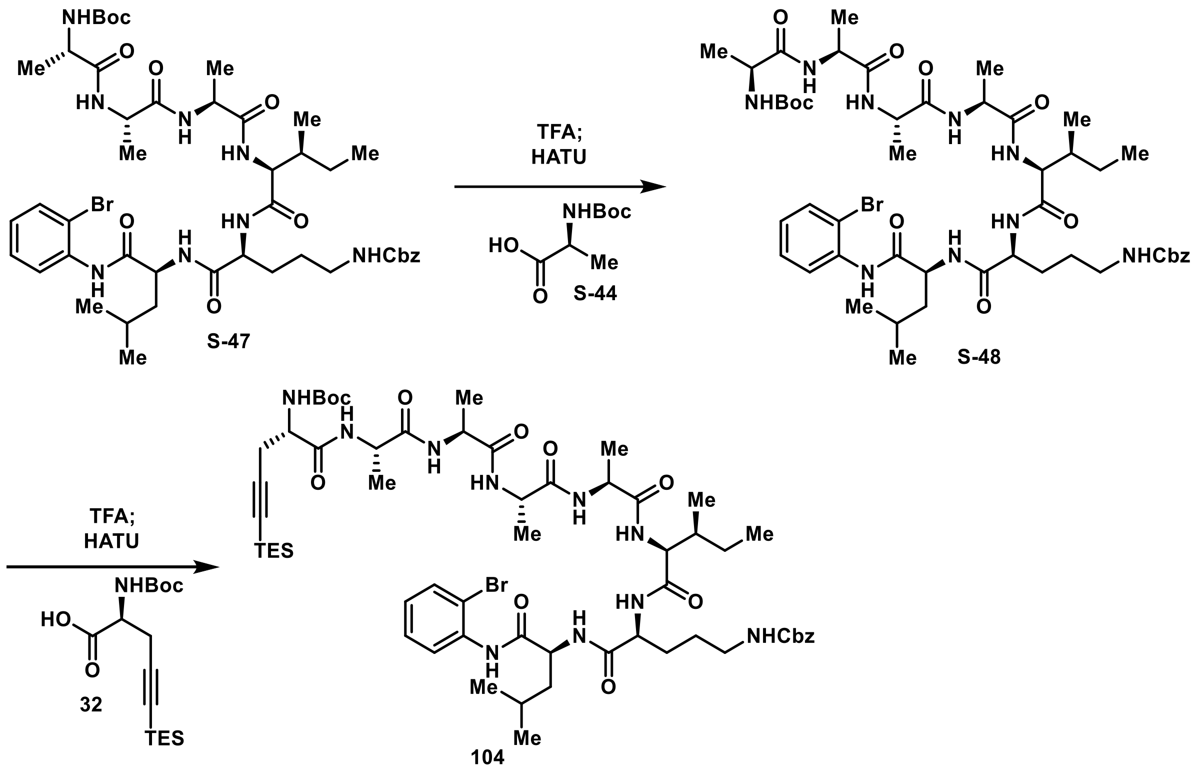

Crude product **S-47** (1.0 mmol) was dissolved in DCM (6 mL), treated dropwise with TFA (4 mL), and stirred at room temperature for 1 h. The mixture was co-evaporated with toluene (10 mL × 3) *in vacuo* to afford a residue for direct use in the next step.
A mixture of the residue and Boc-*L*-alanine (260 mg, 1.2 mmol, 1.2 equiv.) in DMF (15 mL) was treated sequentially with DIPEA (0.7 mL, 4.0 mmol, 4.0 equiv.) and HATU (456 mg, 1.2 mmol, 1.2 equiv.). The reaction was stirred for 1 h, then water (45 mL) was added slowly to the reaction mixture to form a precipitate. The solid was filtered, washed successively with EtOH and EtOAc (twice each), and dried to give a residue **S-48**, which was used directly in the next step.

The residue was dissolved in DCM (6 mL), treated dropwise with TFA (4 mL), and stirred at room temperature for 1 h. The mixture was co-evaporated with toluene (10 mL × 3) *in vacuo* to afford a residue.
To a solution of this crude residue, compound **32** (394 mg, 1.2 mmol, 1.2 equiv.), and DIPEA (0.7 mL, 4.0 mmol, 4.0 equiv.) in DMF (20 mL) was added HATU (456 mg, 1.2 mmol, 1.2 equiv.) in one portion. The reaction mixture was stirred at room temperature for 2.5 h, then water (60 mL) was added slowly to the reaction mixture to form a precipitate. The solid was filtered, washed successively with EtOH and EtOAc (twice each), and dried to yield the product **104** as a white solid (680 mg, 55% overall yield).

Compound 104

Physical State: white solid

^{1}H NMR (400 MHz, DMSO-d_6): δ 9.45 (s, 1H), 8.13 (d, J = 7.8 Hz, 1H), 8.08 – 7.86 (m, 6H), 7.71 (d, J = 8.5 Hz, 1H), 7.61 (dd, J = 14.7, 8.0 Hz, 2H), 7.40 – 7.18 (m, 7H), 7.12 (t, J = 7.5 Hz, 1H), 7.03 (d, J = 8.7 Hz, 1H), 4.98 (s, 2H), 4.51 (q, J = 7.9 Hz, 1H), 4.39 – 3.99 (m, 7H), 2.96 (s, 2H), 2.66 – 2.57 (m, 1H), 2.46 – 2.34 (m, 1H), 1.77 – 1.63 (m, 3H), 1.63 – 1.55 (m, 2H), 1.53 – 1.41 (m, 3H), 1.37 (s, 9H), 1.28 – 1.09 (m, 13H), 1.04 – 0.87 (m, 12H), 0.87 – 0.81 (m, 3H), 0.82 – 0.70 (m, 6H), 0.50 (q, J = 8.1 Hz, 6H).

^{13}C NMR (101 MHz, DMSO-d_6): δ 171.67, 171.56, 171.36, 170.81, 170.48, 169.84, 162.14, 155.95, 154.94, 137.01, 135.71, 132.48, 128.16, 127.87, 127.57, 126.84, 126.36, 121.04, 117.44, 105.26, 82.35, 78.01, 64.98, 56.45, 53.10, 51.88, 51.29, 47.89, 40.44, 36.72, 35.61, 30.58, 29.12, 27.93, 25.80, 23.97, 23.09, 22.94, 21.30, 18.07, 17.94, 17.82, 17.71, 15.02, 10.94, 7.18, 3.76.

HRMS (ESI-TOF): calculated for $C_{59}H_{91}BrN_{10}NaO_{12}Si^+$ $[M+Na]^+$: 1261.5663, found: 1261.5667.

$[\alpha]^{25}_D$: –25.2 (c = 1.0, $CHCl_3$)

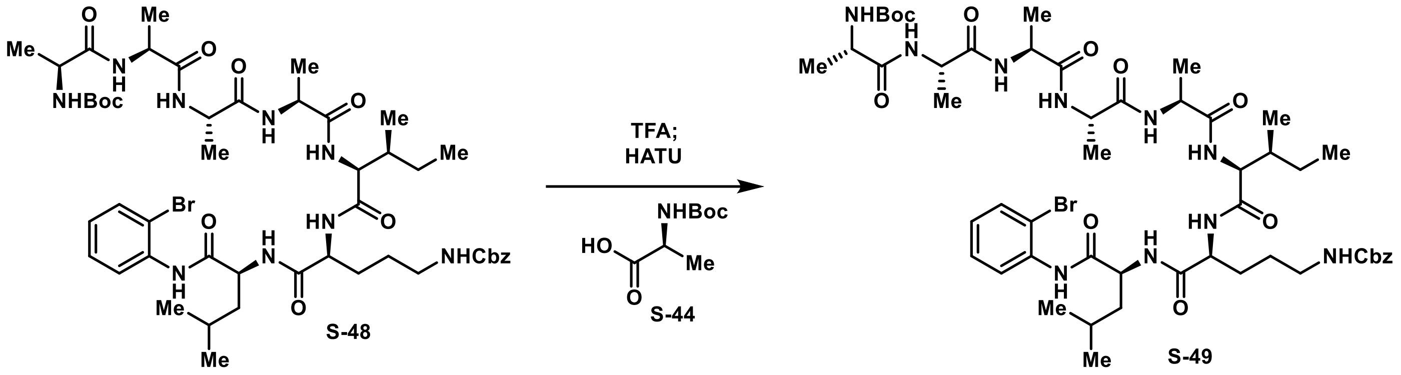

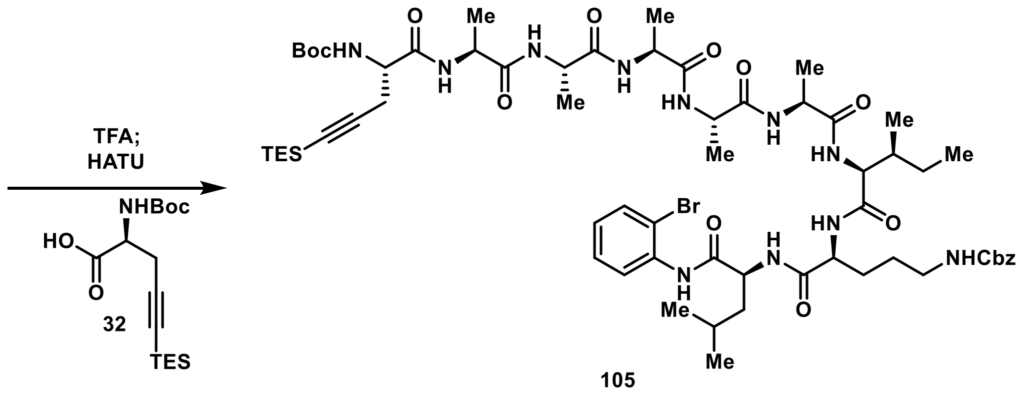

Crude product **S-48** (1.0 mmol) was dissolved in DCM (6 mL), treated dropwise with TFA (4 mL), and stirred at room temperature for 1 h. The mixture was co-evaporated with toluene (10 mL × 3) *in vacuo* to afford a residue for direct use in the next step.

A mixture of the residue and Boc-*L*-alanine (260 mg, 1.2 mmol, 1.2 equiv.) in DMF (20 mL) was treated sequentially with DIPEA (0.7 mL, 4.0 mmol, 4.0 equiv.) and HATU (456 mg, 1.2 mmol, 1.2 equiv.). The reaction was stirred for 2 h, then water (60 mL) was added slowly to the reaction mixture to form a precipitate. The solid was filtered, washed successively with EtOH and EtOAc (twice each), and dried to give a residue **S-49**, which was used directly in the next step.

The residue was dissolved in DCM (6 mL), treated dropwise with TFA (4 mL), and stirred at room temperature for 1 h. The mixture was co-evaporated with toluene (10 mL × 3) *in vacuo* to afford a residue.

To a solution of this crude residue, compound **32** (394 mg, 1.2 mmol, 1.2 equiv.), and DIPEA (0.7 mL, 4.0 mmol, 4.0 equiv.) in DMF (30 mL) was added HATU (456 mg, 1.2 mmol, 1.2 equiv.) in one portion. The reaction mixture was stirred at room temperature for 6 h, then water (90 mL) was added slowly to the reaction mixture to form a precipitate. The solid was filtered, washed successively with EtOH and EtOAc (twice each), and dried to yield the product **105** as a white solid (578 mg, 44% overall yield).

Compound 105

Physical State: white solid

^{1}H NMR (400 MHz, DMSO-*d*$_6$): δ 9.45 (s, 1H), 8.13 (d, *J* = 7.9 Hz, 1H), 8.05 (d, *J* = 6.9 Hz, 1H), 7.95 (q, *J* = 12.5, 9.3 Hz, 6H), 7.71 (d, *J* = 8.4 Hz, 1H), 7.61 (dd, *J* = 17.8, 7.9 Hz, 2H), 7.38 – 7.20 (m, 7H), 7.12 (t, *J* = 7.7 Hz, 1H), 7.03 (d, *J* = 8.8 Hz, 1H), 4.98 (s, 2H), 4.51 (d, *J* = 7.9 Hz, 1H), 4.37 – 4.09 (m, 8H), 3.02 – 2.92 (m, 2H), 2.68 – 2.58 (m, 1H), 2.46 – 2.35 (m, 1H), 1.68 (s, 3H), 1.59 (d, *J* = 7.0 Hz, 2H), 1.53 – 1.41 (m, 3H), 1.37 (s, 9H), 1.18 (d, *J* = 6.9 Hz, 16H), 1.01 – 0.87 (m, 12H), 0.85 (d, *J* = 6.1 Hz, 3H), 0.82 – 0.71 (m, 6H), 0.51 (q, *J* = 7.9 Hz, 6H).

^{13}C NMR (151 MHz, DMSO-*d*$_6$): δ 172.41, 172.34, 172.32, 172.07, 171.52, 170.54, 162.86, 156.67, 155.67, 137.74, 136.44, 133.20, 128.87, 128.59, 128.28, 127.54, 127.04, 118.13, 105.95, 83.10, 78.74, 65.70, 57.20, 53.82, 52.62, 52.05, 48.63, 48.60, 48.52, 41.15, 40.58, 37.41, 36.32, 31.30, 29.83, 28.65, 26.49, 24.69, 23.77, 23.64, 22.02, 18.78, 18.62, 18.58, 18.46, 18.39, 15.74, 11.64, 7.88, 4.47.

HRMS (ESI-TOF): calculated for $C_{62}H_{96}BrN_{11}NaO_{13}Si^{+}$ $[M+Na]^{+}$: 1332.6034, found: 1332.6033.

$[\alpha]^{25}_{D}$: –33.0 (*c* = 1.0, $CHCl_3$)

General synthetic procedure for Larock precursors S-4–43

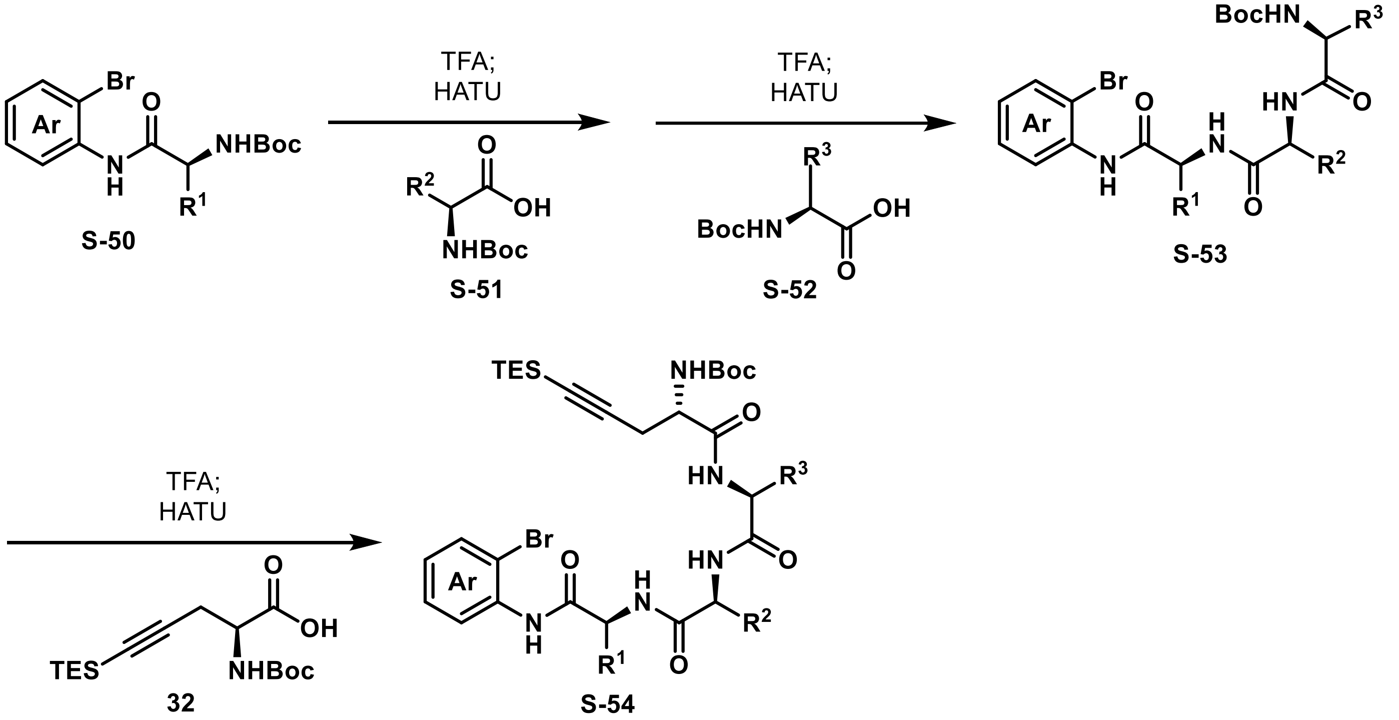

Compound **S-50** (1.0 mmol) was dissolved in DCM (3 mL), treated dropwise with TFA (1.5 mL), and stirred at room temperature for 1 h. The mixture co-evaporated with toluene (10 mL × 3) *in vacuo* to afford a residue, which was used directly in the next step without purification.
To a solution of the crude residue, **S-51** (1.2 mmol, 1.2 equiv.), and DIPEA (0.7 mL, 4.0 mmol, 4.0 equiv.) in DMF (10 mL) was added HATU (456 mg, 1.2 mmol, 1.2 equiv.) in one portion. After stirring at room temperature for 1 h, the reaction was quenched with 0.5 M HCl (20 mL) and extracted with EtOAc (20 mL × 3). The combined organic layers were washed with saturated aqueous NaCl, dried over Na_2SO_4, and concentrated *in vacuo* to give a residue that was used directly in the subsequent step.

This residue was dissolved in DCM (3 mL), treated dropwise with TFA (1.5 mL), and stirred at room temperature for 1 h. The mixture was co-evaporated with toluene (10 mL × 3) *in vacuo* to afford a residue for direct use in the next step.
A mixture of the residue and **S-52** (1.2 mmol, 1.2 equiv.) in DMF (10 mL) was treated sequentially with DIPEA (0.7 mL, 4.0 mmol, 4.0 equiv.) and HATU (456 mg, 1.2 mmol, 1.2 equiv.). The reaction was stirred for 1 h, quenched with 0.5 M HCl (20 mL), and extracted with EtOAc (20 mL × 3). The combined organic extracts were washed with saturated aqueous NaCl, dried over Na_2SO_4, and concentrated *in vacuo* to give a residue **S-53**, which was used directly in the next step.

The residue **S-53** was dissolved in DCM (3 mL), treated dropwise with TFA (1.5 mL), and stirred at room temperature for 1 h. The mixture was co-evaporated with toluene (10 mL × 3) *in vacuo* to afford a residue.
To a solution of this crude residue, compound **32** (394 mg, 1.2 mmol, 1.2 equiv.), and DIPEA (0.7 mL, 4.0 mmol, 4.0 equiv.) in DMF (10 mL) was added HATU (456 mg, 1.2 mmol, 1.2 equiv.) in one portion. After stirring at room temperature for 1 h, the reaction was quenched with 0.5 M HCl (20 mL) and extracted with EtOAc (20 mL × 3). The combined organic layers were washed with saturated aqueous NaCl, dried over Na_2SO_4, and concentrated *in vacuo*. The resulting residue was purified by recrystallization (DCM/hexane) or silica gel chromatography to afford the desired compound **S-4–43**.

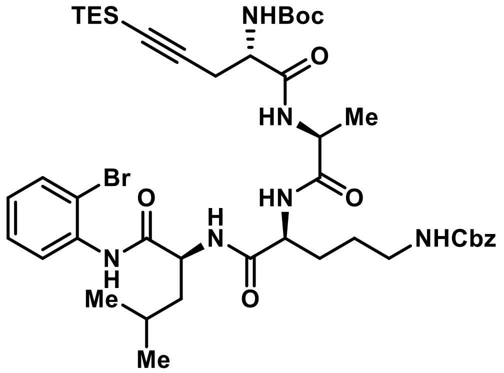

S-4

Purification by recrystallization (DCM/hexane) afforded compound **S-4** as a white solid (804 mg, 88% overall yield).

Compound S-4
Physical State: white solid
^{1}H NMR (400 MHz, CDCl$_3$): δ 8.47 (s, 1H), 8.17 (dd, *J* = 8.2, 1.6 Hz, 1H), 7.52 (dd, *J* = 8.0, 1.4 Hz, 1H), 7.38 – 7.28 (m, 7H), 7.27 – 7.23 (m, 1H), 6.97 (td, *J* = 7.7, 1.6 Hz, 1H), 6.80 (d, *J* = 6.0 Hz, 1H), 5.43 – 5.29 (m, 1H), 5.15 – 5.01 (m, 3H), 4.61 (ddd, *J* = 9.7, 7.9, 5.1 Hz, 2H), 4.48 – 4.35 (m, 1H), 4.12 (q, *J* = 5.8 Hz, 1H), 3.34 (d, *J* = 9.4 Hz, 1H), 3.15 (dd, *J* = 13.3, 6.4 Hz, 1H), 2.76 (t, *J* = 7.3 Hz, 2H), 2.04 (d, *J* = 13.6 Hz, 1H), 1.90 – 1.82 (m, 1H), 1.80 – 1.73 (m, 2H), 1.65 (s, 1H), 1.58 (d, *J* = 7.6 Hz, 2H), 1.45 (s, 9H), 1.42 (d, *J* = 7.2 Hz, 3H), 1.01 – 0.91 (m, 15H), 0.59 (q, *J* = 7.9 Hz, 6H).
^{13}C NMR (151 MHz, CDCl$_3$): δ 172.18, 171.26, 170.47, 144.21, 136.54, 135.74, 132.45, 128.60, 128.20, 128.16, 125.65, 123.21, 121.28, 101.59, 81.49, 66.87, 54.10, 53.05, 52.53, 50.11, 40.13, 29.10, 28.25, 26.55, 24.82, 23.22, 23.04, 21.63, 17.84, 7.57, 4.38.
HRMS (ESI-TOF): calculated for $C_{44}H_{65}BrN_6NaO_8Si^+$ [M+Na]$^+$: 935.3709, found: 935.3714.
TLC: R_f = 0.4 (10:1 DCM: Acetone, $Ce_2(SO_4)_3$ in phosphomolybdic acid).
$[\alpha]^{25}_D$: –7.5 (*c* = 1.0, $CHCl_3$)

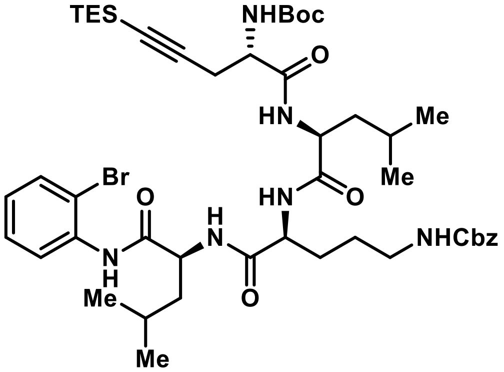

S-5

Purification by recrystallization (DCM/hexane) afforded compound **S-5** as a white solid (708 mg, 74% overall yield).

Compound S-5
Physical State: white solid
^{1}H NMR (400 MHz, CDCl$_3$): δ 8.46 (s, 1H), 8.17 (dd, *J* = 8.2, 1.6 Hz, 1H), 7.52 (dd, *J* = 8.0, 1.5 Hz, 1H), 7.37 – 7.28 (m, 7H), 7.27 – 7.22 (m, 1H), 6.97 (td, *J* = 7.7, 1.6 Hz, 1H), 6.78 (d, *J* = 6.8 Hz, 1H), 5.35 (s, 1H),

5.09 (t, J = 5.8 Hz, 3H), 4.70 – 4.52 (m, 2H), 4.41 (s, 1H), 4.14 (q, J = 6.0 Hz, 1H), 3.41 – 3.27 (m, 1H), 3.21 – 3.10 (m, 1H), 2.80 – 2.71 (m, 2H), 2.10 – 1.95 (m, 1H), 1.89 – 1.81 (m, 1H), 1.75 – 1.62 (m, 5H), 1.61 – 1.51 (m, 3H), 1.45 (s, 9H), 1.00 – 0.90 (m, 21H), 0.59 (q, J = 7.9 Hz, 6H).
^{13}C NMR (151 MHz, $CDCl_3$): δ 172.30, 172.01, 171.38, 170.49, 157.07, 156.40, 136.55, 135.74, 132.45, 128.60, 128.19, 125.65, 123.23, 114.85, 106.75, 101.75, 86.99, 81.44, 66.86, 53.97, 53.03, 52.58, 40.63, 40.12, 39.93, 28.97, 28.24, 26.59, 25.01, 24.82, 23.21, 23.10, 22.87, 21.86, 21.64, 7.56, 4.37.
HRMS (ESI-TOF): calculated for $C_{47}H_{71}BrN_6NaO_8Si^+$ [M+Na]$^+$: 977.4178, found: 977.4176.
TLC: R_f = 0.6 (10:1 DCM: Acetone, $Ce_2(SO_4)_3$ in phosphomolybdic acid).
$[\alpha]^{25}_D$: –22.3 (c = 1.0, $CHCl_3$)

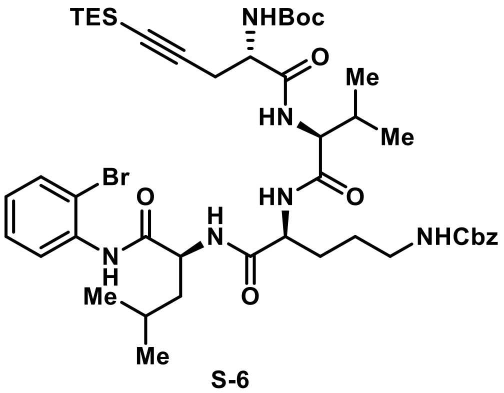

S-6

Purification by recrystallization (DCM/hexane) afforded compound **S-6** as a white solid (818 mg, 87% overall yield).

Compound S-6
Physical State: white solid
^{1}H NMR (400 MHz, $CDCl_3$): δ 8.49 (s, 1H), 8.15 (d, J = 8.4 Hz, 1H), 7.51 (dd, J = 8.0, 1.5 Hz, 1H), 7.38 – 7.28 (m, 7H), 7.26 – 7.21 (m, 1H), 6.97 (td, J = 7.7, 1.6 Hz, 1H), 6.88 (d, J = 6.7 Hz, 1H), 5.39 (s, 1H), 5.16 – 5.02 (m, 3H), 4.69 (s, 1H), 4.62 (q, J = 9.0, 8.4 Hz, 1H), 4.29 (s, 1H), 4.13 (q, J = 5.9 Hz, 1H), 3.44 – 3.26 (m, 1H), 3.22 – 3.05 (m, 1H), 2.78 (d, J = 6.2 Hz, 2H), 2.42 – 2.21 (m, 1H), 2.11 – 1.97 (m, 1H), 1.91 – 1.73 (m, 3H), 1.68 – 1.62 (m, 1H), 1.61 – 1.51 (m, 2H), 1.45 (s, 9H), 1.01 – 0.95 (m, 15H), 0.93 (dd, J = 6.7, 2.8 Hz, 6H), 0.59 (q, J = 7.9 Hz, 6H).
^{13}C NMR (151 MHz, $CDCl_3$): δ 172.33, 171.03, 170.52, 156.45, 155.69, 136.56, 135.76, 132.45, 128.59, 128.19, 128.13, 125.67, 123.34, 114.98, 101.58, 81.49, 66.86, 63.16, 59.47, 54.15, 53.04, 40.12, 39.90, 28.22, 26.64, 24.84, 23.22, 22.82, 21.62, 19.53, 17.60, 7.55, 4.37.
HRMS (ESI-TOF): calculated for $C_{46}H_{69}BrN_6NaO_8Si^+$ [M+Na]$^+$: 963.4022, found: 963.4051.
TLC: R_f = 0.5 (10:1 DCM: Acetone, $Ce_2(SO_4)_3$ in phosphomolybdic acid).
$[\alpha]^{25}_D$: –8.4 (c = 1.0, $CHCl_3$)

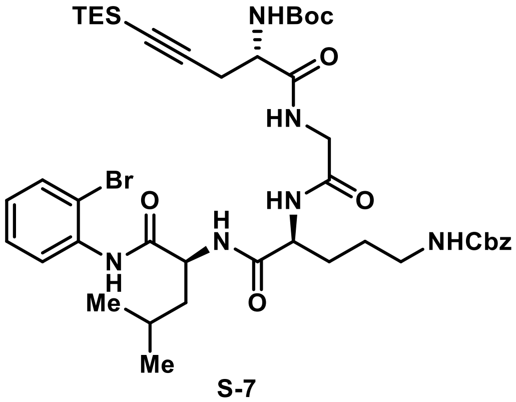

S-7

Purification by recrystallization (DCM/hexane) afforded compound **S-7** as a white solid (689 mg, 76% yield over 4 steps).

Compound S-7
Physical State: white solid
^{1}H NMR (600 MHz, CDCl$_3$): δ 8.61 (s, 1H), 8.04 (d, *J* = 8.0 Hz, 1H), 7.47 (d, *J* = 8.0 Hz, 3H), 7.35 – 7.25 (m, 6H), 7.21 (t, *J* = 7.7 Hz, 1H), 6.94 (t, *J* = 7.5 Hz, 1H), 5.40 (d, *J* = 6.4 Hz, 1H), 5.26 – 5.17 (m, 1H), 5.06 (q, *J* = 12.2 Hz, 2H), 4.82 – 4.56 (m, 2H), 4.28 – 4.13 (m, 1H), 4.03 (d, *J* = 5.3 Hz, 2H), 3.41 – 3.27 (m, 1H), 3.16 – 3.01 (m, 1H), 2.78 (s, 1H), 2.71 (d, *J* = 6.7 Hz, 2H), 1.81 (dt, *J* = 13.1, 6.7 Hz, 1H), 1.77 – 1.61 (m, 3H), 1.59 – 1.47 (m, 2H), 1.40 (s, 9H), 1.01 – 0.89 (m, 15H), 0.54 (q, *J* = 7.8 Hz, 6H).
^{13}C NMR (151 MHz, CDCl$_3$): δ 172.28, 171.47, 170.71, 168.85, 157.24, 136.55, 135.62, 132.56, 128.58, 128.20, 125.94, 123.71, 115.21, 102.32, 86.15, 80.95, 66.90, 53.91, 52.87, 52.17, 43.46, 40.73, 39.75, 38.70, 29.78, 28.33, 26.44, 24.88, 23.40, 23.11, 21.98, 7.55, 4.40.
HRMS (ESI-TOF): calculated for $C_{43}H_{63}BrN_6NaO_8Si^+$ [M+Na]$^+$: 921.3552, found: 921.3557.
TLC: R_f = 0.3 (10:1 DCM: Acetone, $Ce_2(SO_4)_3$ in phosphomolybdic acid).
$[\alpha]^{25}_D$: –15.8 (*c* = 1.0, $CHCl_3$)

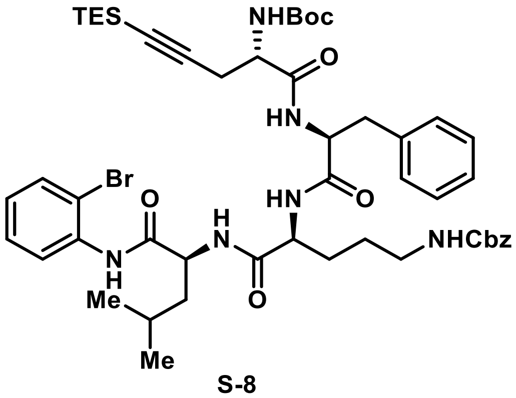

S-8

Purification by recrystallization (DCM/hexane) afforded compound **S-8** as a white solid (744 mg, 75% overall yield).

Compound S-8
Physical State: white solid
^{1}H NMR (600 MHz, CDCl$_3$): δ 8.47 (s, 1H), 8.20 (d, *J* = 8.2 Hz, 1H), 7.50 (d, *J* = 8.0 Hz, 1H), 7.36 – 7.31 (m, 4H), 7.30 – 7.26 (m, 3H), 7.25 – 7.21 (m, 2H), 7.20 – 7.10 (m, 3H), 7.03 (d, *J* = 8.2 Hz, 1H), 6.95 (t, *J* = 7.7 Hz, 1H), 6.83 (d, *J* = 6.7 Hz, 1H), 5.21 (s, 1H), 5.08 (s, 2H), 5.01 – 4.90 (m, 1H), 4.66 – 4.50 (m, 3H),

4.00 – 3.84 (m, 1H), 3.32 (dd, J = 13.8, 5.4 Hz, 1H), 3.21 (dd, J = 14.1, 7.1 Hz, 1H), 3.14 – 3.04 (m, 1H), 2.97 (dd, J = 14.2, 5.7 Hz, 1H), 2.76 (dd, J = 17.7, 5.2 Hz, 1H), 2.62 (dd, J = 17.3, 8.0 Hz, 1H), 2.04 – 1.93 (m, 1H), 1.87 – 1.81 (m, 1H), 1.70 – 1.61 (m, 1H), 1.57 – 1.48 (m, 1H), 1.45 – 1.34 (m, 2H), 1.29 (s, 9H), 0.97 (t, J = 7.9 Hz, 9H), 0.93 (d, J = 6.6 Hz, 3H), 0.89 (d, J = 6.5 Hz, 3H), 0.58 (q, J = 7.9 Hz, 6H).
^{13}C NMR (151 MHz, CDCl$_3$): δ 172.21, 171.49, 170.71, 170.41, 156.78, 136.60, 135.78, 135.68, 132.41, 129.27, 129.17, 128.61, 128.20, 128.14, 127.68, 125.54, 123.00, 114.82, 101.29, 87.27, 81.67, 66.81, 54.80, 54.74, 53.05, 52.88, 39.99, 39.87, 36.60, 28.63, 28.14, 26.50, 24.73, 23.26, 22.83, 21.42, 7.57, 4.38.
HRMS (ESI-TOF): calculated for $C_{50}H_{69}BrN_6NaO_8Si^+$ [M+Na]$^+$: 1011.4022, found: 1011.4020.
TLC: R$_f$ = 0.6 (10:1 DCM: Acetone, $Ce_2(SO_4)_3$ in phosphomolybdic acid).
[α]$^{25}_D$: –28.2 (c = 1.0, CHCl$_3$)

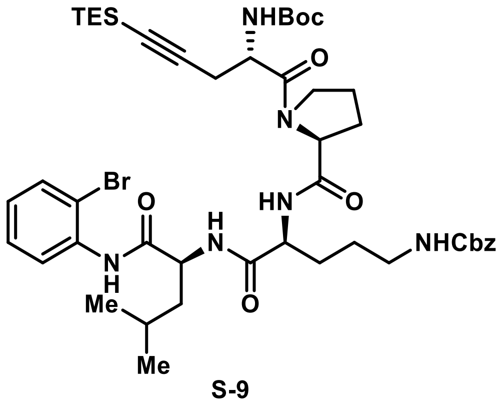

S-9

Purification by silica gel chromatography (40% EtOAc/hexane) afforded compound **S-9** as a white solid (580 mg, 62% overall yield).

Compound S-9
Physical State: white solid
^{1}H NMR (400 MHz, CDCl$_3$): δ 8.41 (s, 1H), 8.22 – 8.12 (m, 1H), 7.56 – 7.44 (m, 2H), 7.39 – 7.31 (m, 6H), 7.30 – 7.27 (m, 1H), 6.97 (td, J = 7.7, 1.6 Hz, 1H), 5.36 (t, J = 7.2 Hz, 1H), 5.24 (t, J = 6.0 Hz, 1H), 5.10 (s, 2H), 4.66 – 4.46 (m, 4H), 3.78 (t, J = 6.5 Hz, 2H), 3.36 – 3.10 (m, 2H), 2.65 (q, J = 9.3, 8.7 Hz, 2H), 2.18 – 1.93 (m, 5H), 1.85 (td, J = 10.4, 5.6 Hz, 1H), 1.70 (dq, J = 13.9, 8.6, 8.1 Hz, 3H), 1.57 (h, J = 6.7 Hz, 2H), 1.43 (s, 9H), 1.00 – 0.91 (m, 15H), 0.56 (qd, J = 7.9, 3.9 Hz, 6H).
^{13}C NMR (101 MHz, CDCl$_3$): δ 171.61, 170.83, 169.73, 156.46, 154.70, 135.81, 134.94, 131.75, 127.89, 127.50, 127.47, 125.00, 122.43, 101.42, 85.11, 79.67, 66.20, 60.39, 52.27, 51.20, 47.34, 39.49, 39.27, 27.87, 27.66, 27.64, 27.50, 25.90, 24.62, 24.16, 23.29, 22.42, 21.03, 6.80, 3.72, 3.65.
HRMS (ESI-TOF): calculated for $C_{46}H_{67}BrN_6NaO_8Si^+$ [M+Na]$^+$: 961.3865, found: 961.3862.
TLC: R$_f$ = 0.4 (1:1 Hexane:EtOAc, $Ce_2(SO_4)_3$ in phosphomolybdic acid).
[α]$^{25}_D$: +6.6 (c = 1.0, CHCl$_3$)

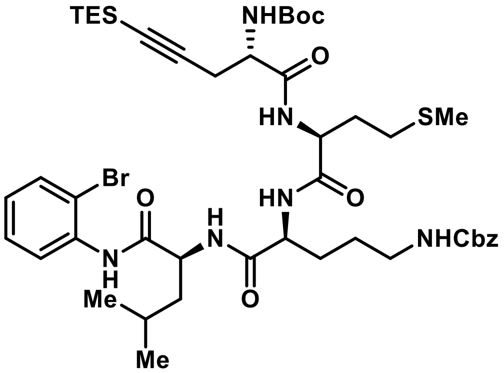

S-10

Purification by recrystallization (DCM/hexane) afforded compound **S-10** as a white solid (577 mg, 58% overall yield).

Compound S-10
Physical State: white solid
^{1}H NMR (600 MHz, CDCl$_3$): δ 8.43 (s, 1H), 8.18 (dd, *J* = 8.3, 1.7 Hz, 1H), 7.61 – 7.47 (m, 2H), 7.37 – 7.26 (m, 7H), 7.25 – 7.22 (m, 1H), 6.95 (td, *J* = 7.7, 1.6 Hz, 1H), 5.38 – 5.23 (m, 1H), 5.05 (dd, *J* = 18.9, 6.7 Hz, 3H), 4.69 – 4.49 (m, 3H), 4.07 (q, *J* = 5.9 Hz, 1H), 3.40 – 3.08 (m, 2H), 2.79 – 2.67 (m, 2H), 2.55 (ddq, *J* = 20.7, 13.8, 6.6 Hz, 2H), 2.12 (d, *J* = 6.5 Hz, 1H), 2.10 (s, 3H), 2.07 – 2.01 (m, 1H), 1.84 (ddd, *J* = 13.9, 9.2, 5.1 Hz, 1H), 1.76 (t, *J* = 11.3 Hz, 3H), 1.66 (d, *J* = 6.9 Hz, 1H), 1.60 – 1.50 (m, 2H), 1.43 (s, 9H), 0.96 (t, *J* = 7.9 Hz, 12H), 0.91 (d, *J* = 6.4 Hz, 3H), 0.57 (q, *J* = 7.9 Hz, 6H).
^{13}C NMR (151 MHz, CDCl$_3$): δ 172.23, 171.00, 170.43, 169.22, 150.48, 145.45, 136.54, 135.74, 132.43, 128.60, 128.20, 125.59, 123.06, 114.76, 107.58, 91.31, 81.53, 66.87, 54.40, 54.40, 53.05, 52.69, 40.06, 39.92, 30.38, 29.62, 28.97, 28.29, 26.61, 24.79, 23.24, 22.96, 21.57, 15.44, 7.58, 4.38.
HRMS (ESI-TOF): calculated for $C_{46}H_{69}BrN_6NaO_8SSi^+$ $[M+Na]^+$: 995.3742, found: 995.3752.
TLC: R_f = 0.5 (10:1 DCM: Acetone, $Ce_2(SO_4)_3$ in phosphomolybdic acid).
$[\alpha]^{25}_D$: –13.6 (*c* = 1.0, $CHCl_3$)

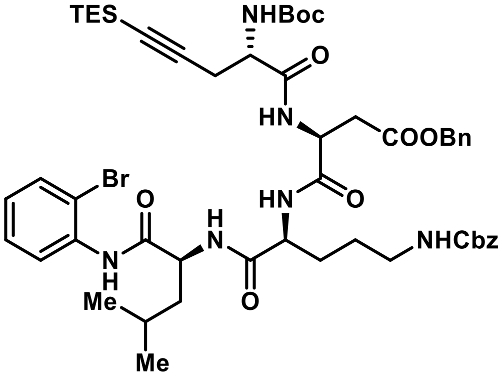

S-11

Purification by recrystallization (DCM/hexane) afforded compound **S-11** as a white solid (785 mg, 75% overall yield).

Compound S-11
Physical State: white solid
^{1}H NMR (400 MHz, CDCl$_3$): δ 8.46 (s, 1H), 8.24 (dd, *J* = 8.2, 1.6 Hz, 1H), 7.67 (d, *J* = 7.9 Hz, 1H), 7.54 – 7.43 (m, 2H), 7.37 – 7.28 (m, 10H), 7.17 (d, *J* = 7.9 Hz, 1H), 6.96 (td, *J* = 7.7, 1.6 Hz, 1H), 5.40 – 5.22 (m,

1H), 5.16 – 4.97 (m, 5H), 4.68 (s, 1H), 4.59 (ddd, *J* = 9.9, 8.0, 5.0 Hz, 2H), 4.12 – 3.95 (m, 1H), 3.32 – 3.08 (m, 3H), 2.84 – 2.65 (m, 3H), 2.10 (s, 1H), 1.92 – 1.83 (m, 1H), 1.79 – 1.64 (m, 4H), 1.59 – 1.50 (m, 2H), 1.46 (s, 9H), 1.00 – 0.91 (m, 15H), 0.58 (q, *J* = 7.9 Hz, 6H).
^{13}C NMR (151 MHz, CDCl$_3$): δ 172.06, 171.81, 171.46, 170.36, 156.80, 136.62, 135.77, 135.14, 132.39, 128.73, 128.63, 128.57, 128.32, 128.18, 128.15, 125.47, 122.76, 114.55, 101.36, 87.21, 81.70, 67.16, 66.77, 54.45, 53.03, 50.41, 40.10, 39.94, 34.71, 28.62, 28.25, 26.32, 24.78, 23.23, 22.88, 21.56, 7.54, 4.37.
HRMS (ESI-TOF): calculated for $C_{52}H_{71}BrN_6NaO_{10}Si^+$ $[M+Na]^+$: 1069.4077, found: 1069.4082.
TLC: R_f = 0.6 (10:1 DCM: Acetone, $Ce_2(SO_4)_3$ in phosphomolybdic acid).
$[\alpha]^{25}_D$: –17.8 (*c* = 1.0, $CHCl_3$)

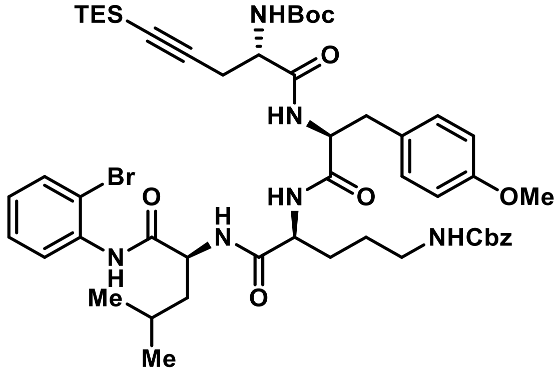

S-12

Purification by recrystallization (DCM/hexane) afforded compound **S-12** as a white solid (806 mg, 79% overall yield).

Compound S-12
Physical State: white solid
^{1}H NMR (600 MHz, CDCl$_3$): δ 8.47 (s, 1H), 8.20 (dd, *J* = 8.2, 1.6 Hz, 1H), 7.50 (dd, *J* = 8.0, 1.5 Hz, 1H), 7.36 – 7.25 (m, 6H), 7.18 (d, *J* = 7.9 Hz, 1H), 7.04 (dd, *J* = 23.0, 8.3 Hz, 3H), 6.95 (td, *J* = 7.7, 1.6 Hz, 1H), 6.84 – 6.76 (m, 3H), 5.22 (s, 1H), 5.13 – 5.03 (m, 2H), 4.98 (t, *J* = 5.9 Hz, 1H), 4.64 – 4.51 (m, 3H), 3.93 (s, 1H), 3.73 (s, 3H), 3.35 – 3.26 (m, 1H), 3.25 – 3.16 (m, 1H), 3.09 (dq, *J* = 12.9, 6.4 Hz, 1H), 2.90 (dd, *J* = 14.3, 5.7 Hz, 1H), 2.83 – 2.70 (m, 1H), 2.63 (dd, *J* = 17.3, 8.0 Hz, 1H), 2.08 – 1.96 (m, 1H), 1.88 – 1.81 (m, 1H), 1.80 – 1.74 (m, 2H), 1.67 (d, *J* = 8.5 Hz, 1H), 1.55 (dtd, *J* = 14.2, 9.0, 5.4 Hz, 1H), 1.46 – 1.37 (m, 2H), 1.29 (s, 9H), 0.97 (t, *J* = 7.9 Hz, 9H), 0.93 (d, *J* = 6.6 Hz, 3H), 0.89 (d, *J* = 6.5 Hz, 3H), 0.58 (q, *J* = 7.9 Hz, 6H).
^{13}C NMR (151 MHz, CDCl$_3$): δ 172.27, 171.51, 170.88, 170.43, 159.07, 156.76, 136.59, 135.81, 132.41, 130.31, 128.61, 128.20, 128.16, 128.13, 127.35, 125.52, 123.03, 114.87, 114.57, 101.24, 81.69, 66.80, 55.24, 54.90, 54.83, 53.09, 40.11, 39.80, 35.62, 28.59, 28.05, 26.56, 24.74, 23.26, 22.81, 21.39, 7.57, 4.38.
HRMS (ESI-TOF): calculated for $C_{51}H_{71}BrN_6NaO_9Si^+$ $[M+Na]^+$: 1041.4127, found: 1041.4133.
TLC: R_f = 0.5 (15:1 DCM: Acetone, $Ce_2(SO_4)_3$ in phosphomolybdic acid).
$[\alpha]^{25}_D$: –28.4 (*c* = 1.0, $CHCl_3$)

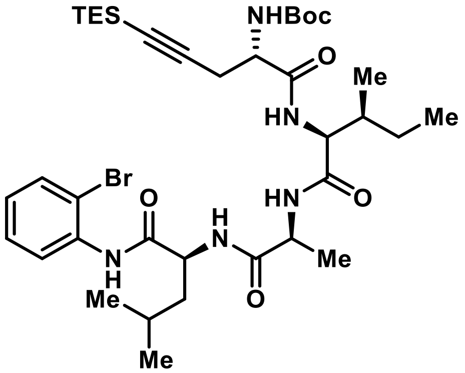

S-13

Purification by recrystallization (DCM/hexane) afforded compound **S-13** as a white solid (574 mg, 74% overall yield).

Compound S-13

Physical State: white solid

^{1}H NMR (600 MHz, CDCl$_3$): δ 8.53 (s, 1H), 8.15 (d, *J* = 8.3 Hz, 1H), 7.50 (d, *J* = 8.0 Hz, 1H), 7.31 (d, *J* = 7.7 Hz, 1H), 7.28 – 7.25 (m, 1H), 7.18 (d, *J* = 7.9 Hz, 1H), 6.94 (t, *J* = 7.7 Hz, 1H), 6.63 (d, *J* = 6.1 Hz, 1H), 5.50 – 5.33 (m, 1H), 4.75 – 4.44 (m, 2H), 4.23 (t, *J* = 5.1 Hz, 1H), 4.00 (d, *J* = 7.9 Hz, 1H), 2.82 (dd, *J* = 17.5, 5.5 Hz, 1H), 2.70 (dd, *J* = 17.4, 7.2 Hz, 1H), 2.05 (s, 1H), 1.89 – 1.79 (m, 2H), 1.74 – 1.69 (m, 1H), 1.46 (s, 3H), 1.45 (s, 9H), 1.13 – 1.08 (m, 2H), 1.01 – 0.94 (m, 15H), 0.91 (t, *J* = 6.2 Hz, 6H), 0.58 (q, *J* = 7.9 Hz, 6H).

^{13}C NMR (151 MHz, CDCl$_3$): δ 173.09, 172.17, 171.77, 170.66, 170.42, 156.75, 147.90, 135.86, 132.41, 128.06, 125.60, 123.50, 121.91, 101.03, 87.75, 81.92, 67.33, 59.19, 54.75, 53.14, 49.32, 39.89, 35.95, 28.20, 24.82, 24.72, 23.30, 22.71, 21.42, 17.55, 16.21, 11.88, 7.54, 4.36.

HRMS (ESI-TOF): calculated for $C_{37}H_{60}BrN_5NaO_6Si^+$ [M+Na]$^+$: 800.3388, found: 800.3398.

TLC: R$_f$ = 0.5 (10:1 DCM: Acetone, $Ce_2(SO_4)_3$ in phosphomolybdic acid).

[α]$^{25}_D$: –21.4 (*c* = 1.0, CHCl$_3$)

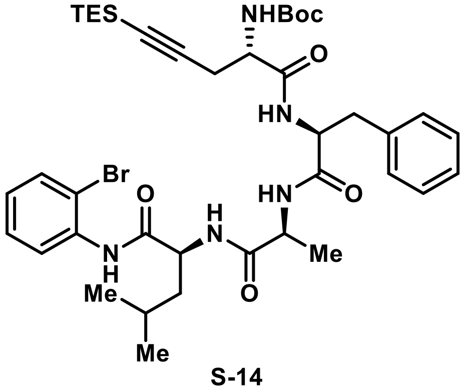

S-14

Purification by recrystallization (DCM/hexane) afforded compound **S-14** as a white solid (632 mg, 77% overall yield).

Compound S-14

Physical State: white solid

^{1}H NMR (600 MHz, CDCl$_3$): δ 8.54 (s, 1H), 8.20 (dd, *J* = 8.1, 1.6 Hz, 1H), 7.51 (dd, *J* = 8.0, 1.4 Hz, 1H), 7.34 – 7.29 (m, 2H), 7.27 (dd, *J* = 7.7, 1.8 Hz, 2H), 7.16 – 7.11 (m, 2H), 7.10 – 7.01 (m, 2H), 6.95 (td, *J* = 7.6, 1.6 Hz, 1H), 6.67 (d, *J* = 6.5 Hz, 1H), 5.22 (s, 1H), 4.64 (p, *J* = 7.4 Hz, 1H), 4.56 (ddt, *J* = 15.8, 7.8, 4.9 Hz,

2H), 3.87 (ddd, J = 8.1, 4.9, 2.6 Hz, 1H), 3.36 (dd, J = 14.2, 5.2 Hz, 1H), 2.96 (dd, J = 14.2, 5.4 Hz, 1H), 2.78 (dd, J = 17.2, 5.0 Hz, 1H), 2.57 (dd, J = 17.4, 8.3 Hz, 1H), 1.86 (ddd, J = 14.1, 9.7, 4.5 Hz, 1H), 1.77 (ddd, J = 14.2, 10.6, 4.7 Hz, 1H), 1.70 – 1.64 (m, 1H), 1.31 (d, J = 7.3 Hz, 3H), 1.27 (s, 9H), 0.98 (t, J = 7.9 Hz, 9H), 0.92 (d, J = 6.6 Hz, 3H), 0.88 (d, J = 6.5 Hz, 3H), 0.60 (q, J = 7.9 Hz, 6H).
^{13}C NMR (151 MHz, CDCl$_3$): δ 172.98, 171.56, 170.53, 170.41, 156.88, 135.84, 135.44, 132.39, 129.20, 129.15, 128.08, 127.80, 125.50, 123.11, 115.03, 101.02, 87.59, 81.91, 55.22, 54.50, 53.08, 49.19, 39.84, 36.42, 28.11, 24.68, 23.30, 22.80, 21.30, 17.33, 7.57, 4.38.
HRMS (ESI-TOF): calculated for $C_{40}H_{58}BrN_5NaO_6Si^+$ [M+Na]$^+$: 834.3232, found: 834.3228.
TLC: R$_f$ = 0.5 (10:1 DCM: Acetone, $Ce_2(SO_4)_3$ in phosphomolybdic acid).
[α]$^{25}_D$: –9.8 (c = 1.0, CHCl$_3$)

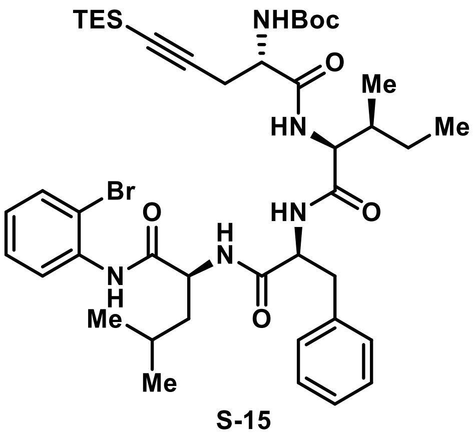

S-15

Purification by recrystallization (DCM/hexane) afforded compound **S-15** as a white solid (728 mg, 85% overall yield).

Compound S-15
Physical State: white solid
^{1}H NMR (600 MHz, CDCl$_3$): δ 8.51 (s, 1H), 8.11 (d, J = 8.2 Hz, 1H), 7.56 – 7.42 (m, 1H), 7.25 – 7.09 (m, 7H), 7.08 – 6.77 (m, 3H), 5.39 (s, 1H), 4.96 (s, 1H), 4.72 – 4.54 (m, 1H), 4.19 (s, 1H), 4.06 (s, 1H), 3.39 (d, J = 14.4 Hz, 1H), 2.95 (dd, J = 14.6, 9.6 Hz, 1H), 2.73 – 2.57 (m, 2H), 1.90 – 1.79 (m, 2H), 1.78 – 1.74 (m, 1H), 1.68 – 1.60 (m, 1H), 1.43 (s, 9H), 1.42 – 1.37 (m, 1H), 1.24 – 1.12 (m, 1H), 0.98 – 0.91 (m, 15H), 0.81 – 0.74 (m, 6H), 0.57 (q, J = 7.9 Hz, 6H).
^{13}C NMR (151 MHz, CDCl$_3$): δ 171.73, 170.59, 170.33, 156.61, 136.83, 135.79, 132.42, 128.97, 128.59, 128.04, 126.85, 125.66, 123.49, 115.26, 101.70, 98.82, 86.85, 81.43, 59.42, 54.22, 53.93, 53.02, 39.95, 37.32, 36.06, 28.29, 24.73, 24.55, 23.27, 22.68, 21.62, 15.80, 11.73, 7.56, 4.39.
HRMS (ESI-TOF): calculated for $C_{43}H_{64}BrN_5NaO_6Si^+$ [M+Na]$^+$: 876.3701, found: 876.3701.
TLC: R$_f$ = 0.5 (15:1 DCM: Acetone, $Ce_2(SO_4)_3$ in phosphomolybdic acid).
[α]$^{25}_D$: –27.1 (c = 1.0, CHCl$_3$)

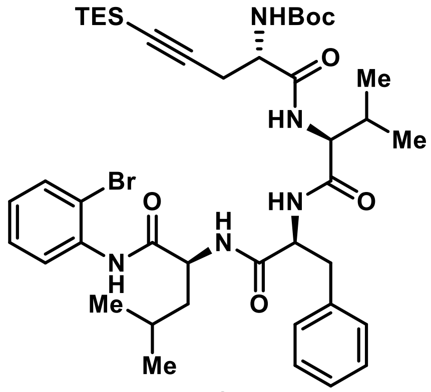

S-16

Purification by recrystallization (DCM/hexane) afforded compound **S-16** as a white solid (710 mg, 84% overall yield).

Compound S-16

Physical State: white solid

^{1}H NMR (600 MHz, CDCl$_3$): δ 8.48 (s, 1H), 8.11 (d, *J* = 8.1 Hz, 1H), 7.51 (dd, *J* = 8.0, 1.5 Hz, 1H), 7.21 – 7.07 (m, 7H), 7.03 – 6.87 (m, 3H), 5.42 – 5.21 (m, 1H), 4.92 (s, 1H), 4.67 – 4.57 (m, 1H), 4.15 (s, 1H), 4.08 (d, *J* = 6.5 Hz, 1H), 3.35 (d, *J* = 14.3 Hz, 1H), 2.99 (dd, *J* = 14.5, 8.8 Hz, 1H), 2.73 – 2.60 (m, 2H), 2.21 – 2.07 (m, 1H), 1.85 – 1.78 (m, 1H), 1.66 – 1.59 (m, 1H), 1.43 (s, 9H), 1.42 – 1.38 (m, 1H), 0.99 – 0.90 (m, 15H), 0.85 (d, *J* = 6.9 Hz, 3H), 0.75 – 0.67 (m, 3H), 0.57 (q, *J* = 7.9 Hz, 6H).

^{13}C NMR (151 MHz, CDCl$_3$): δ 171.72, 171.60, 170.69, 170.27, 156.58, 136.75, 135.79, 132.44, 128.99, 128.67, 128.07, 126.93, 125.70, 123.51, 115.23, 101.74, 86.87, 81.42, 59.78, 54.08, 54.00, 53.00, 39.98, 37.39, 29.60, 28.29, 28.22, 24.73, 23.21, 22.72, 21.67, 19.40, 17.26, 7.54, 4.39.

HRMS (ESI-TOF): calculated for $C_{42}H_{62}BrN_5NaO_6Si^+$ $[M+Na]^+$: 862.3545, found: 862.3560.

TLC: R_f = 0.5 (15:1 DCM: Acetone, $Ce_2(SO_4)_3$ in phosphomolybdic acid).

$[\alpha]^{25}_{D}$: –15.8 (*c* = 1.0, $CHCl_3$)

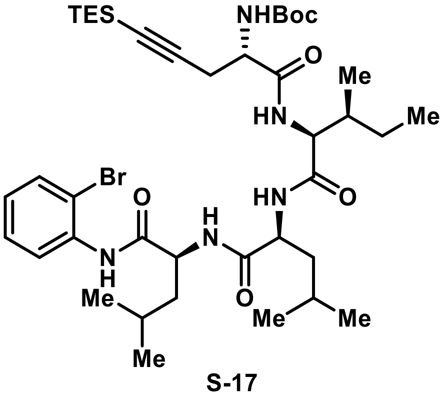

S-17

Purification by recrystallization (DCM/hexane) afforded compound **S-17** as a white solid (652 mg, 79% overall yield).

Compound S-17

Physical State: white solid

^{1}H NMR (600 MHz, CDCl$_3$): δ 8.55 (s, 1H), 8.15 (dq, *J* = 8.2, 1.6 Hz, 1H), 7.49 (dd, *J* = 8.0, 1.4 Hz, 1H), 7.28 – 7.25 (m, 1H), 7.25 – 7.23 (m, 1H), 7.14 – 7.01 (m, 1H), 6.94 (td, *J* = 7.7, 1.6 Hz, 1H), 6.67 (s, 1H), 5.38 (s, 1H), 4.68 – 4.54 (m, 2H), 4.29 – 4.20 (m, 1H), 4.05 – 3.90 (m, 1H), 2.81 (dd, *J* = 17.4, 5.2 Hz, 1H),

2.69 (dd, J = 17.4, 7.4 Hz, 1H), 2.07 – 1.95 (m, 1H), 1.92 – 1.77 (m, 3H), 1.72 – 1.62 (m, 4H), 1.45 (s, 9H), 1.17 – 1.09 (m, 1H), 0.99 – 0.87 (m, 27H), 0.58 (q, J = 7.9 Hz, 6H).
^{13}C NMR (151 MHz, CDCl$_3$): δ 173.06, 171.76, 170.73, 170.55, 156.81, 135.90, 132.39, 128.01, 125.55, 123.43, 115.28, 101.01, 87.70, 81.81, 59.38, 54.82, 53.09, 52.10, 40.00, 39.84, 36.01, 28.15, 24.97, 24.93, 24.77, 23.35, 22.74, 21.36, 20.83, 16.12, 11.85, 7.54, 4.35.
HRMS (ESI-TOF): calculated for $C_{43}H_{63}BrN_6NaO_8Si^+$ [M+Na]$^+$: 842.3858, found: 842.3864.
TLC: R$_f$ = 0.6 (10:1 DCM: Acetone, $Ce_2(SO_4)_3$ in phosphomolybdic acid).
[α]$^{25}_{D}$: –21.6 (c = 1.0, CHCl$_3$)

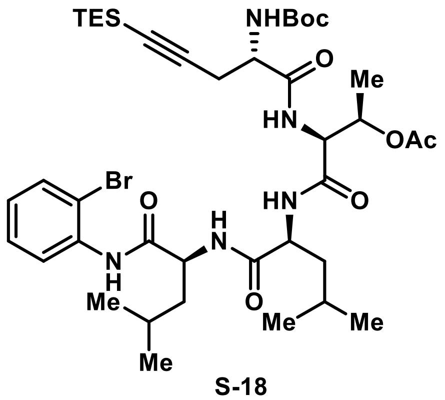

S-18

Purification by recrystallization (DCM/hexane) afforded compound **S-18** as a white solid (680 mg, 80% overall yield).

Compound S-18
Physical State: white solid
^{1}H NMR (600 MHz, CDCl$_3$): δ 8.51 (s, 1H), 8.20 (dd, J = 8.2, 1.7 Hz, 1H), 7.49 (dt, J = 8.0, 1.5 Hz, 1H), 7.30 – 7.23 (m, 2H), 7.17 (d, J = 7.0 Hz, 1H), 7.02 (d, J = 7.9 Hz, 1H), 6.94 (td, J = 7.6, 1.5 Hz, 1H), 5.50 – 5.31 (m, 2H), 4.72 – 4.53 (m, 2H), 4.43 – 4.28 (m, 1H), 4.05 – 3.90 (m, 1H), 2.87 (dd, J = 17.4, 5.0 Hz, 1H), 2.66 (dt, J = 17.5, 8.7 Hz, 1H), 1.96 (d, J = 1.3 Hz, 3H), 1.93 – 1.82 (m, 2H), 1.80 – 1.75 (m, 1H), 1.72 – 1.65 (m, 1H), 1.63 – 1.52 (m, 2H), 1.47 (d, J = 1.5 Hz, 9H), 1.32 (d, J = 6.7 Hz, 3H), 1.00 – 0.92 (m, 12H), 0.92 – 0.88 (m, 6H), 0.86 (d, J = 6.4 Hz, 3H), 0.58 (d, J = 7.9, 1.3 Hz, 6H).
^{13}C NMR (151 MHz, CDCl$_3$): δ 172.67, 172.00, 170.42, 169.40, 168.80, 157.13, 135.79, 132.38, 128.10, 125.47, 122.90, 114.82, 100.93, 87.79, 81.86, 69.97, 58.87, 55.05, 53.03, 52.02, 39.93, 28.20, 24.96, 24.70, 23.31, 23.27, 22.94, 21.39, 20.91, 20.83, 17.44, 7.53, 4.33.
HRMS (ESI-TOF): calculated for $C_{40}H_{64}BrN_5NaO_8Si^+$ [M+Na]$^+$: 872.3600, found: 872.3613.
TLC: R$_f$ = 0.5 (10:1 DCM: Acetone, $Ce_2(SO_4)_3$ in phosphomolybdic acid).
[α]$^{25}_{D}$: –19.9 (c = 1.0, CHCl$_3$)

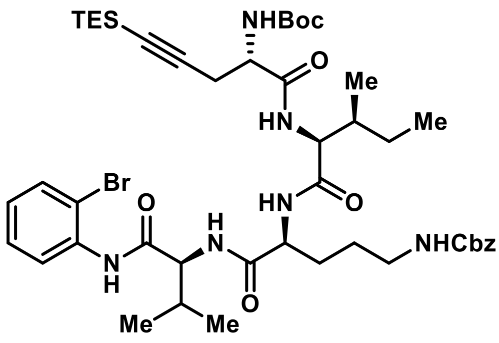

S-19

Purification by recrystallization (DCM/hexane) afforded compound **S-19** as a white solid (769 mg, 82% overall yield).

Compound S-19
Physical State: white solid
^{1}H NMR (400 MHz, DMSO-*d*$_6$): δ 9.55 (s, 1H), 8.23 – 7.83 (m, 2H), 7.78 – 7.48 (m, 3H), 7.40 – 7.27 (m, 5H), 7.27 – 7.21 (m, 1H), 7.19 – 6.98 (m, 2H), 4.98 (s, 2H), 4.53 – 3.94 (m, 4H), 3.05 – 2.86 (m, 2H), 2.69 – 2.47 (m, 4H), 2.20 – 2.03 (m, 1H), 1.78 – 1.59 (m, 2H), 1.56 – 1.42 (m, 3H), 1.36 (s, 9H), 1.09 – 1.00 (m, 1H), 1.00 – 0.83 (m, 15H), 0.82 – 0.70 (m, 6H), 0.58 – 0.36 (m, 6H).
^{13}C NMR (101 MHz, DMSO-*d*$_6$): δ 171.42, 170.42, 169.89, 155.96, 155.01, 145.24, 144.44, 137.01, 135.67, 132.52, 128.15, 127.81, 127.58, 126.95, 126.79, 117.58, 105.38, 92.82, 82.31, 78.05, 65.00, 57.67, 56.41, 53.29, 51.96, 37.00, 30.55, 29.11, 27.91, 25.85, 23.79, 22.70, 19.06, 17.70, 15.06, 10.94, 7.18, 3.77.
HRMS (ESI-TOF): calculated for $C_{46}H_{69}BrN_6NaO_8^+$ [M+Na]$^+$: 963.4022, found: 963.4028.
TLC: R$_f$ = 0.5 (10:1 DCM: Acetone, $Ce_2(SO_4)_3$ in phosphomolybdic acid).
[α]$^{25}_{D}$: –18.8 (*c* = 1.0, $CHCl_3$)

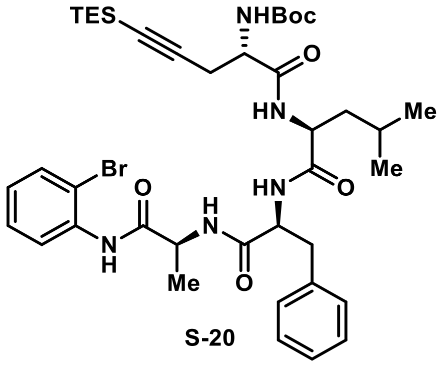

S-20

Purification by recrystallization (DCM/hexane) afforded compound **S-20** as a white solid (626 mg, 77% overall yield).

Compound S-20
Physical State: white solid
^{1}H NMR (400 MHz, CDCl$_3$): δ 8.52 (s, 1H), 8.17 (dd, *J* = 8.2, 1.6 Hz, 1H), 7.53 (dd, *J* = 8.0, 1.5 Hz, 1H), 7.33 – 7.28 (m, 1H), 7.25 – 7.16 (m, 6H), 7.13 (d, *J* = 6.4 Hz, 1H), 6.99 – 6.92 (m, 2H), 6.83 (d, *J* = 5.9 Hz, 1H), 5.30 (d, *J* = 5.2 Hz, 1H), 4.86 (q, *J* = 7.7 Hz, 1H), 4.66 (p, *J* = 7.1 Hz, 1H), 4.33 – 4.22 (m, 1H), 4.06 (dt, *J* = 7.0, 5.5 Hz, 1H), 3.34 (dd, *J* = 15.0, 5.3 Hz, 1H), 3.03 (dd, *J* = 14.4, 8.4 Hz, 1H), 2.70 – 2.57 (m, 2H), 1.58

– 1.51 (m, 2H), 1.46 (s, 9H), 0.99 (t, J = 7.9 Hz, 9H), 0.88 (d, J = 6.1 Hz, 3H), 0.84 (d, J = 5.4 Hz, 3H), 0.60 (q, J = 7.9 Hz, 6H).

^{13}C NMR (101 MHz, CDCl$_3$): δ 170.78, 170.72, 169.59, 155.87, 136.00, 135.02, 131.74, 128.52, 128.36, 127.94, 127.79, 127.40, 126.29, 124.93, 122.46, 114.24, 101.07, 86.16, 80.75, 53.06, 52.57, 49.44, 39.68, 36.57, 27.59, 27.51, 24.15, 22.27, 21.92, 21.17, 16.47, 6.87, 3.68.

HRMS (ESI-TOF): calculated for $C_{40}H_{58}BrN_5NaO_6Si^+$ [M+Na]$^+$: 834.3232, found: 834.3236.

TLC: R$_f$ = 0.5 (10:1 DCM: Acetone, $Ce_2(SO_4)_3$ in phosphomolybdic acid).

[α]$^{25}_{D}$: –28.8 (c = 1.0, CHCl$_3$)

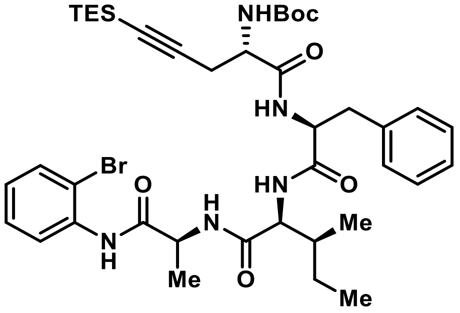

S-21

Purification by recrystallization (DCM/hexane) afforded compound **S-21** as a white solid (595 mg, 73% overall yield).

Compound S-21

Physical State: white solid

^{1}H NMR (600 MHz, CDCl$_3$): δ 8.66 (s, 1H), 8.05 (d, J = 8.1 Hz, 1H), 7.52 (d, J = 8.0 Hz, 1H), 7.32 – 7.15 (m, 7H), 7.13 (d, J = 7.5 Hz, 2H), 6.97 (t, J = 7.7 Hz, 1H), 5.40 (s, 1H), 4.80 (s, 2H), 4.55 (s, 1H), 4.17 (s, 1H), 3.33 – 3.10 (m, 1H), 2.99 (dd, J = 13.9, 6.5 Hz, 1H), 2.70 (dd, J = 17.7, 5.1 Hz, 1H), 2.60 (dd, J = 17.6, 7.7 Hz, 1H), 1.93 (s, 1H), 1.49 (d, J = 7.0 Hz, 3H), 1.34 (s, 9H), 1.14 – 1.06 (m, 2H), 0.96 (t, J = 7.9 Hz, 9H), 0.88 – 0.78 (m, 6H), 0.57 (p, J = 7.9, 7.0 Hz, 6H).

^{13}C NMR (151 MHz, CDCl$_3$): δ 171.34, 171.16, 170.81, 156.01, 135.94, 135.71, 132.57, 129.34, 128.91, 128.13, 127.41, 126.00, 124.13, 115.68, 109.30, 102.93, 101.94, 86.44, 80.92, 58.24, 54.76, 54.04, 49.73, 37.81, 36.86, 28.24, 24.55, 23.48, 18.01, 15.64, 11.62, 7.58, 4.42.

HRMS (ESI-TOF): calculated for $C_{40}H_{58}BrN_5NaO_6Si^+$ [M+Na]$^+$: 834.3232, found: 834.3237.

TLC: R$_f$ = 0.6 (10:1 DCM: Acetone, $Ce_2(SO_4)_3$ in phosphomolybdic acid).

[α]$^{25}_{D}$: –31.5 (c = 1.0, CHCl$_3$)

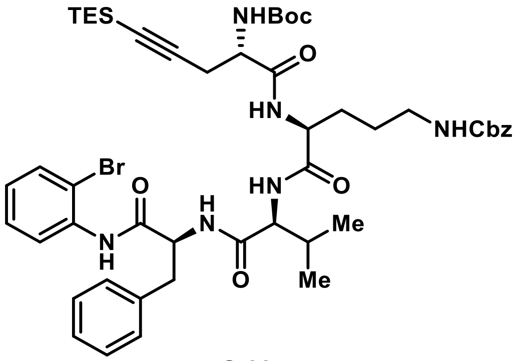

S-22

Purification by recrystallization (DCM/hexane) afforded compound **S-22** as a white solid (659 mg, 68% yield over 6 steps).

Compound S-22

Physical State: white solid

^{1}H NMR (400 MHz, DMSO-*d*$_6$): δ 9.55 (s, 1H), 8.30 (d, *J* = 7.9 Hz, 1H), 7.90 (d, *J* = 8.1 Hz, 1H), 7.74 (d, *J* = 8.9 Hz, 1H), 7.62 (dd, *J* = 8.0, 1.4 Hz, 1H), 7.53 (dd, *J* = 8.1, 1.7 Hz, 1H), 7.37 – 7.32 (m, 5H), 7.31 – 7.23 (m, 5H), 7.22 – 7.14 (m, 2H), 7.14 – 7.08 (m, 1H), 7.04 (d, *J* = 8.8 Hz, 1H), 4.99 (s, 2H), 4.80 (q, *J* = 7.8 Hz, 1H), 4.26 (d, *J* = 5.2 Hz, 1H), 4.15 (ddd, *J* = 22.0, 8.8, 5.2 Hz, 2H), 3.12 (dd, *J* = 13.9, 5.5 Hz, 1H), 2.99 – 2.89 (m, 3H), 2.61 (dd, *J* = 17.1, 3.9 Hz, 1H), 2.43 (dd, *J* = 17.1, 10.3 Hz, 1H), 1.99 – 1.88 (m, 1H), 1.62 – 1.52 (m, 1H), 1.47 – 1.41 (m, 1H), 1.36 (s, 9H), 1.34 – 1.31 (m, 1H), 1.28 – 1.17 (m, 1H), 0.92 (t, *J* = 7.9 Hz, 9H), 0.76 (t, *J* = 6.9 Hz, 6H), 0.51 (q, *J* = 7.9 Hz, 6H).

^{13}C NMR (101 MHz, DMSO-*d*$_6$): δ 170.97, 170.74, 170.08, 169.83, 155.94, 154.90, 137.19, 137.04, 135.67, 132.50, 129.01, 128.16, 127.94, 127.85, 127.59, 127.57, 126.81, 126.19, 117.16, 105.31, 89.65, 82.34, 77.99, 65.00, 57.02, 53.98, 53.25, 51.95, 37.05, 30.70, 29.30, 27.92, 25.59, 23.11, 18.98, 17.70, 7.19, 3.77.

HRMS (ESI-TOF): calculated for $C_{49}H_{67}BrN_6NaO_8Si^+$ [M+Na]$^+$: 997.3865, found: 997.3865.

TLC: R$_f$ = 0.4 (10:1 DCM: Acetone, $Ce_2(SO_4)_3$ in phosphomolybdic acid).

[α]$^{25}_D$: –17.1 (*c* = 1.0, $CHCl_3$)

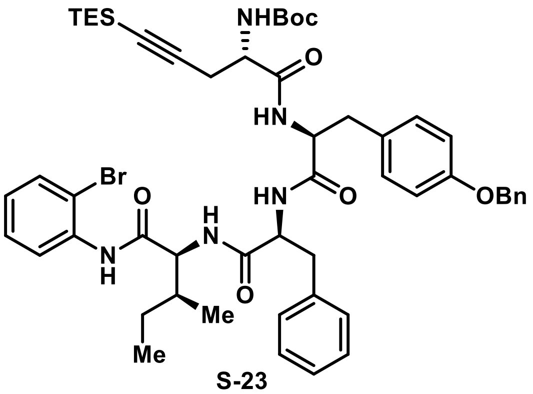

S-23

Starting from 10.0 mmol **S-50**. Purification by recrystallization (DCM/hexane) afforded compound **S-23** as a white solid (5.6 g, 60% overall yield).

Compound S-23

Physical State: white solid

^{1}H NMR (600 MHz, DMSO-*d*$_6$):δ 9.53 (d, *J* = 6.1 Hz, 1H), 8.31 – 8.15 (m, 2H), 7.73 (dd, *J* = 40.7, 8.2 Hz, 1H), 7.61 (ddd, *J* = 14.7, 8.0, 1.4 Hz, 1H), 7.52 (ddd, *J* = 13.7, 8.0, 1.6 Hz, 1H), 7.40 – 7.31 (m, 5H), 7.31 – 7.26 (m, 2H), 7.25 – 7.19 (m, 4H), 7.18 – 7.06 (m, 2H), 7.05 – 6.98 (m, 2H), 6.95 (t, *J* = 9.3 Hz, 1H), 6.81 – 6.74 (m, 2H), 4.91 (s, 2H), 4.77 (td, *J* = 8.4, 6.2 Hz, 1H), 4.64 – 4.52 (m, 1H), 4.47 – 4.33 (m, 1H), 4.05 (ddd, *J* = 10.5, 8.9, 3.7 Hz, 1H), 3.05 – 2.91 (m, 1H), 2.87 – 2.76 (m, 2H), 2.61 (ddd, *J* = 22.6, 12.9, 5.8 Hz, 1H), 2.28 (dd, *J* = 17.1, 10.5 Hz, 1H), 1.89 (tt, *J* = 16.8, 8.1 Hz, 1H), 1.32 (d, *J* = 3.6 Hz, 9H), 1.25 – 1.15 (m, 1H), 1.04 – 0.94 (m, 1H), 0.88 (t, *J* = 7.9 Hz, 9H), 0.85 – 0.74 (m, 6H), 0.47 (q, *J* = 7.9 Hz, 6H).

^{13}C NMR (151 MHz, DMSO-*d*$_6$): δ 171.75, 170.94, 170.84, 170.48, 157.42, 155.53, 137.82, 137.72, 136.48, 133.22, 130.87, 129.95, 129.79, 128.94, 128.91, 128.55, 128.53, 128.28, 128.11, 127.68, 127.59, 126.81, 118.68, 114.77, 106.12, 82.93, 78.66, 69.58, 56.56, 54.27, 53.92, 40.59, 38.87, 37.35, 28.63, 26.32, 23.77, 14.95, 12.18, 11.61, 7.88, 4.48.

HRMS (ESI-TOF): calculated for $C_{53}H_{68}BrN_5NaO_7Si^+$ [M+Na]$^+$: 1016.3964, found: 1016.3966.

TLC: R_f = 0.5 (40:1 DCM:Acetone, $Ce_2(SO_4)_3$ in phosphomolybdic acid).
$[\alpha]^{25}_D$: –15.6 (c = 1.0, $CHCl_3$)

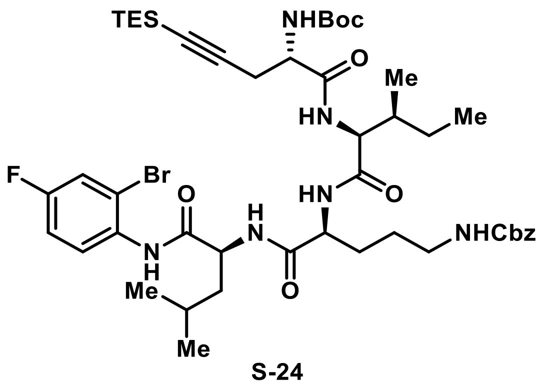

S-24

Purification by recrystallization (DCM/hexane) afforded compound **S-24** as a white solid (695 mg, 71% overall yield).

Compound S-24
Physical State: white solid
^{1}H NMR (400 MHz, DMSO-d_6): δ 9.60 (s, 1H), 8.11 (d, J = 7.8 Hz, 1H), 8.04 (d, J = 7.8 Hz, 1H), 7.86 (d, J = 8.2 Hz, 1H), 7.52 – 7.43 (m, 1H), 7.43 – 7.36 (m, 1H), 7.36 – 7.27 (m, 6H), 7.27 – 7.22 (m, 1H), 7.22 – 7.14 (m, 1H), 7.07 (d, J = 8.7 Hz, 1H), 4.98 (s, 2H), 4.51 (q, J = 7.6 Hz, 1H), 4.36 – 4.21 (m, 2H), 4.19 – 4.02 (m, 1H), 2.96 (q, J = 6.6 Hz, 2H), 2.69 – 2.53 (m, 1H), 2.48 – 2.37 (m, 1H), 1.74 – 1.63 (m, 2H), 1.62 – 1.53 (m, 3H), 1.53 – 1.39 (m, 5H), 1.38 (s, 9H), 1.00 – 0.75 (m, 21H), 0.67 – 0.25 (m, 6H).
^{13}C NMR (101 MHz, DMSO-d_6): δ 171.58, 171.41, 170.95, 170.01, 159.80, 157.38, 155.95, 154.96, 137.74, 137.00, 128.72, 128.63, 128.15, 127.57, 121.65, 112.84, 112.62, 105.35, 82.30, 78.00, 64.99, 53.23, 51.84, 51.40, 50.90, 40.88, 40.29, 28.97, 27.90, 25.82, 23.99, 23.84, 22.90, 21.47, 21.33, 7.17, 3.77.
^{19}F NMR (565 MHz, DMSO-d_6) δ -104.75.
HRMS (ESI-TOF): calculated for $C_{47}H_{70}BrFN_6NaO_8SSi^+$ $[M+Na]^+$: 995.4084, found: 995.4083.
TLC: R_f = 0.5 (10:1 DCM: Acetone, $Ce_2(SO_4)_3$ in phosphomolybdic acid).
$[\alpha]^{25}_D$: –19.8 (c = 1.0, $CHCl_3$)

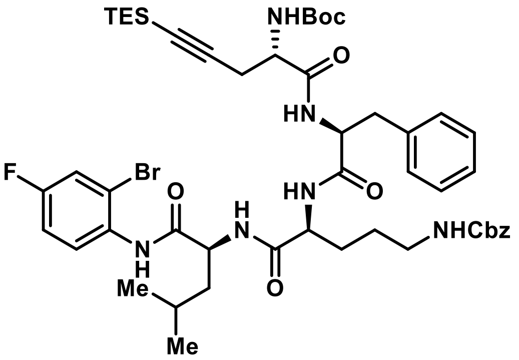

S-25

Purification by recrystallization (DCM/hexane) afforded compound **S-25** as a white solid (653 mg, 67% overall yield).

Compound S-25
Physical State: white solid

^{1}H NMR (400 MHz, DMSO-*d*$_6$): δ 9.66 (s, 1H), 8.20 (dd, *J* = 8.0, 3.6 Hz, 2H), 7.86 (d, *J* = 8.1 Hz, 1H), 7.51 – 7.43 (m, 1H), 7.42 – 7.36 (m, 1H), 7.35 – 7.29 (m, 5H), 7.28 – 7.25 (m, 1H), 7.23 – 7.15 (m, 6H), 7.00 (d, *J* = 8.9 Hz, 1H), 4.99 (s, 2H), 4.62 – 4.46 (m, 2H), 4.40 – 4.23 (m, 1H), 4.17 – 3.97 (m, 1H), 3.04 – 2.93 (m, 3H), 2.77 (dd, *J* = 13.9, 8.9 Hz, 1H), 2.35 (dd, *J* = 17.0, 10.5 Hz, 1H), 1.77 – 1.65 (m, 2H), 1.65 – 1.57 (m, 2H), 1.55 – 1.49 (m, 1H), 1.47 – 1.40 (m, 2H), 1.35 (s, 9H), 1.23 (s, 1H), 0.92 (q, *J* = 7.9, 6.7 Hz, 15H), 0.50 (q, *J* = 7.9 Hz, 6H).
^{13}C NMR (101 MHz, DMSO-*d*$_6$): δ 171.45, 170.99, 170.38, 170.00, 159.82, 157.40, 155.97, 154.83, 137.78, 137.18, 137.00, 129.17, 128.73, 128.64, 128.15, 127.77, 127.58, 127.56, 126.07, 121.77, 112.88, 112.66, 105.32, 82.26, 77.98, 65.01, 53.46, 53.27, 51.84, 51.41, 40.34, 37.46, 29.34, 27.91, 25.81, 24.10, 23.11, 22.90, 21.42, 7.18, 3.76.
^{19}F NMR (565 MHz, DMSO-*d*$_6$) δ -104.72.
HRMS (ESI-TOF): calculated for $C_{50}H_{68}BrFN_6NaO_8Si^+$ [M+Na]$^+$: 1029.3928, found: 1029.3922.
TLC: R$_f$ = 0.5 (10:1 DCM: Acetone, $Ce_2(SO_4)_3$ in phosphomolybdic acid).
[α]$^{25}_D$: –20.8 (*c* = 1.0, $CHCl_3$)

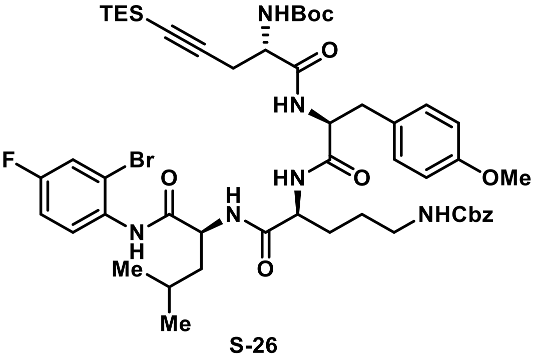

S-26

Purification by recrystallization (DCM/hexane) afforded compound **S-26** as a white solid (702 mg, 68% overall yield).

Compound S-26
Physical State: white solid
^{1}H NMR (600 MHz, CDCl$_3$): δ 8.43 (s, 1H), 8.07 (dd, *J* = 9.1, 5.6 Hz, 1H), 7.35 – 7.28 (m, 5H), 7.27 – 7.25 (m, 1H), 7.20 (d, *J* = 8.0 Hz, 1H), 7.10 – 7.02 (m, 3H), 6.99 (td, *J* = 8.5, 2.8 Hz, 1H), 6.81 (d, *J* = 8.0 Hz, 3H), 5.22 (s, 1H), 5.07 (s, 2H), 4.99 (s, 1H), 4.60 – 4.47 (m, 3H), 3.92 (s, 1H), 3.72 (s, 3H), 3.29 (dd, *J* = 14.3, 5.0 Hz, 1H), 3.24 – 3.14 (m, 1H), 3.09 (dq, *J* = 13.2, 6.5 Hz, 1H), 2.89 (dd, *J* = 14.3, 5.5 Hz, 1H), 2.77 (dd, *J* = 17.3, 5.1 Hz, 1H), 2.62 (dd, *J* = 17.4, 8.1 Hz, 1H), 2.01 (q, *J* = 11.5, 9.5 Hz, 1H), 1.85 – 1.81 (m, 1H), 1.78 – 1.72 (m, 1H), 1.65 (p, *J* = 7.1, 6.7 Hz, 1H), 1.59 – 1.50 (m, 1H), 1.45 – 1.36 (m, 2H), 1.29 (s, 9H), 0.97 (t, *J* = 7.9 Hz, 9H), 0.92 (d, *J* = 6.6 Hz, 3H), 0.88 (d, *J* = 6.6 Hz, 3H), 0.58 (q, *J* = 7.9 Hz, 6H).
^{13}C NMR (151 MHz, CDCl$_3$): δ 172.33, 171.68, 171.07, 170.59, 159.84, 159.11, 158.20, 156.84, 136.58, 132.31, 130.28, 128.61, 128.21, 128.15, 127.23, 124.67, 119.54, 119.37, 115.76, 115.00, 114.86, 114.61, 101.11, 87.47, 81.78, 66.80, 55.24, 55.01, 54.91, 53.34, 52.99, 40.15, 39.66, 35.55, 28.47, 28.05, 26.63, 24.77, 23.26, 22.76, 21.31, 7.57, 4.38.
^{19}F NMR (565 MHz, CDCl$_3$) δ -115.95.
HRMS (ESI-TOF): calculated for $C_{51}H_{70}BrFN_6NaO_9Si^+$ [M+Na]$^+$: 1059.4033, found: 1059.4042.
TLC: R$_f$ = 0.7 (10:1 DCM: Acetone, $Ce_2(SO_4)_3$ in phosphomolybdic acid).
[α]$^{25}_D$: –25.6 (*c* = 1.0, $CHCl_3$)

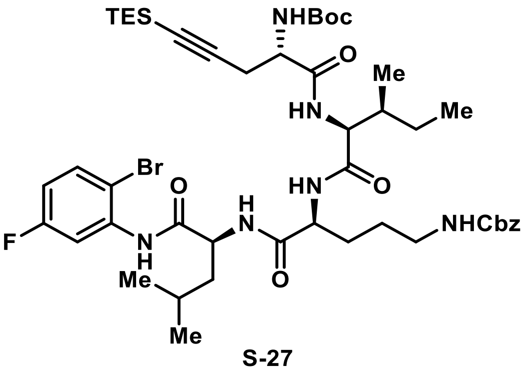

S-27

Purification by recrystallization (DCM/hexane) afforded compound **S-27** as a white solid (721 mg, 74% yield over 6 steps).

Compound S-27

Physical State: white solid

^{1}H NMR (400 MHz, DMSO-*d*$_6$): δ 9.47 (s, 1H), 8.22 (d, *J* = 7.8 Hz, 1H), 8.08 (d, *J* = 7.8 Hz, 1H), 7.64 (ddt, *J* = 19.2, 10.9, 4.4 Hz, 3H), 7.41 – 7.20 (m, 6H), 7.16 (d, *J* = 8.7 Hz, 1H), 7.01 (td, *J* = 8.6, 3.1 Hz, 1H), 4.98 (s, 2H), 4.53 (q, *J* = 7.8 Hz, 1H), 4.30 (q, *J* = 7.2 Hz, 1H), 4.17 (dt, *J* = 24.8, 8.9 Hz, 2H), 2.96 (q, *J* = 7.0, 6.4 Hz, 2H), 2.59 (dd, *J* = 17.1, 4.3 Hz, 1H), 2.49 – 2.34 (m, 2H), 1.68 (d, *J* = 11.0 Hz, 3H), 1.57 (p, *J* = 4.3 Hz, 2H), 1.54 – 1.40 (m, 3H), 1.37 (s, 9H), 1.10 – 1.00 (m, 1H), 1.00 – 0.87 (m, 12H), 0.86 – 0.81 (m, 3H), 0.81 – 0.72 (m, 6H), 0.50 (q, *J* = 7.9 Hz, 6H).

^{13}C NMR (101 MHz, DMSO-*d*$_6$): δ 171.53, 171.09, 170.34, 169.93, 162.06, 159.64, 155.93, 155.02, 137.13, 137.01, 133.69, 133.60, 128.14, 127.57, 113.51, 113.29, 112.21, 111.92, 110.75, 105.37, 82.31, 78.07, 64.98, 56.42, 53.30, 51.89, 51.40, 37.04, 29.07, 27.91, 25.85, 23.95, 23.84, 22.94, 22.65, 21.23, 14.97, 11.00, 7.18, 3.77.

^{19}F NMR (565 MHz, DMSO-*d*$_6$) δ -113.09.

HRMS (ESI-TOF): calculated for $C_{47}H_{70}BrFN_6NaO_8SSi^+$ [M+Na]$^+$: 995.4084, found: 995.4089.

TLC: R$_f$ = 0.6 (10:1 DCM: Acetone, $Ce_2(SO_4)_3$ in phosphomolybdic acid).

[α]$^{25}_D$: –19.6 (*c* = 1.0, $CHCl_3$)

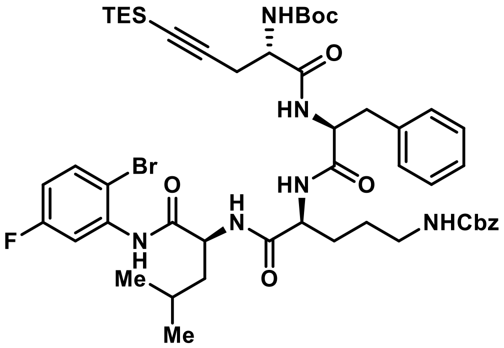

S-28

Purification by recrystallization (DCM/hexane) afforded compound **S-28** as a white solid (708 mg, 70% overall yield).

Compound S-28

Physical State: white solid

^{1}H NMR (400 MHz, DMSO-*d*$_6$): δ 9.52 (s, 1H), 8.22 (dd, *J* = 12.5, 8.0 Hz, 2H), 7.86 (d, *J* = 8.1 Hz, 1H), 7.68 (dd, *J* = 8.9, 5.9 Hz, 1H), 7.60 (dd, *J* = 10.6, 3.0 Hz, 1H), 7.38 – 7.27 (m, 5H), 7.27 – 7.23 (m, 1H), 7.23

– 7.11 (m, 5H), 7.08 – 6.87 (m, 2H), 4.99 (s, 2H), 4.53 (ddd, *J* = 16.6, 11.6, 5.9 Hz, 2H), 4.39 – 4.21 (m, 1H), 4.18 – 3.95 (m, 1H), 3.08 – 2.89 (m, 3H), 2.76 (dd, *J* = 13.9, 9.0 Hz, 1H), 2.58 – 2.51 (m, 1H), 2.34 (dd, *J* = 17.0, 10.5 Hz, 1H), 1.77 – 1.64 (m, 2H), 1.60 (t, *J* = 7.2 Hz, 2H), 1.51 (d, *J* = 9.2 Hz, 1H), 1.47 – 1.41 (m, 1H), 1.35 (s, 9H), 1.23 (s, 1H), 1.00 – 0.76 (m, 15H), 0.49 (q, *J* = 7.7 Hz, 6H).
^{13}C NMR (101 MHz, DMSO-*d*$_6$): δ 171.56, 171.09, 170.39, 170.00, 162.06, 159.64, 155.96, 154.83, 137.17, 137.02, 133.71, 133.61, 129.16, 128.15, 127.78, 127.58, 126.08, 113.35, 112.33, 105.32, 82.26, 77.98, 65.00, 53.44, 53.26, 51.85, 51.49, 37.46, 29.33, 27.92, 27.66, 25.82, 24.06, 23.11, 22.90, 21.37, 7.18, 3.76.
^{19}F NMR (565 MHz, DMSO-*d*$_6$) δ -113.10.
HRMS (ESI-TOF): calculated for $C_{50}H_{68}BrFN_6NaO_8Si^+$ [M+Na]$^+$: 1029.3928, found: 1029.3932.
TLC: R_f = 0.6 (10:1 DCM: Acetone, $Ce_2(SO_4)_3$ in phosphomolybdic acid).
[α]$^{25}_D$: –23.3 (*c* = 1.0, $CHCl_3$)

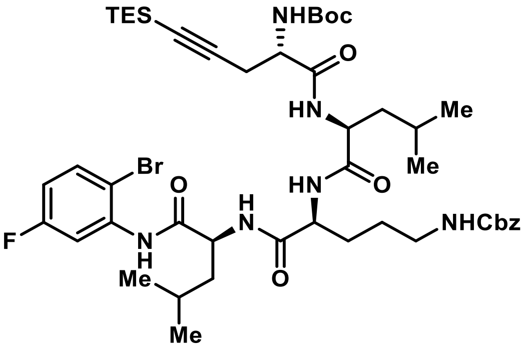

S-29

Purification by recrystallization (DCM/hexane) afforded compound **S-29** as a white solid (683 mg, 69% overall yield).

Compound S-29
Physical State: white solid
^{1}H NMR (400 MHz, DMSO-*d*$_6$): δ 9.47 (s, 1H), 8.24 – 8.09 (m, 1H), 8.05 (d, *J* = 7.6 Hz, 1H), 7.91 – 7.76 (m, 1H), 7.72 – 7.52 (m, 2H), 7.39 – 7.16 (m, 6H), 7.14 – 6.93 (m, 2H), 4.98 (s, 2H), 4.51 (s, 1H), 4.36 – 4.20 (m, 2H), 4.18 – 3.97 (m, 1H), 3.07 – 2.83 (m, 3H), 2.72 (s, 1H), 2.64 – 2.53 (m, 1H), 2.45 – 2.30 (m, 1H), 1.66 (s, 2H), 1.57 (s, 3H), 1.47 – 1.39 (m, 3H), 1.36 (s, 9H), 0.99 – 0.73 (m, 21H), 0.59 – 0.23 (m, 6H).
^{13}C NMR (101 MHz, DMSO-*d*$_6$): δ 171.53, 171.06, 170.00, 162.06, 159.63, 155.94, 154.96, 137.01, 133.68, 133.60, 131.28, 128.15, 127.57, 112.21, 110.76, 105.34, 94.89, 82.29, 78.00, 64.98, 53.23, 51.86, 51.48, 50.87, 40.89, 28.95, 27.90, 25.83, 23.95, 23.83, 22.90, 21.47, 21.28, 16.77, 7.18, 3.76.
^{19}F NMR (565 MHz, DMSO-*d*$_6$) δ -113.11.
HRMS (ESI-TOF): calculated for $C_{47}H_{70}BrFN_6NaO_8SSi^+$ [M+Na]$^+$: 995.4084, found: 995.4084.
TLC: R_f = 0.6 (10:1 DCM: Acetone, $Ce_2(SO_4)_3$ in phosphomolybdic acid).
[α]$^{25}_D$: –30.1 (*c* = 1.0, $CHCl_3$)

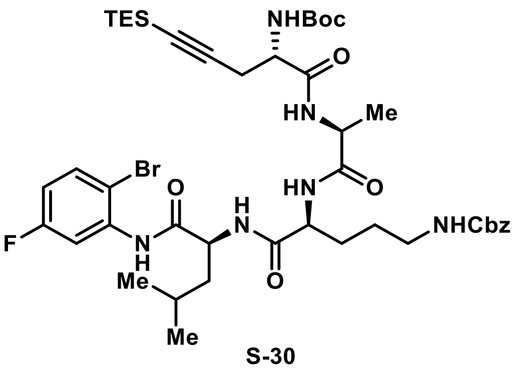

S-30

Purification by recrystallization (DCM/hexane) afforded compound **S-30** as a white solid (599 mg, 64% overall yield).

Compound S-30

Physical State: white solid

^{1}H NMR (400 MHz, DMSO-*d*$_6$): δ 9.48 (s, 1H), 8.16 (d, *J* = 7.7 Hz, 1H), 8.00 (dd, *J* = 13.9, 7.7 Hz, 2H), 7.67 (dd, *J* = 8.9, 5.9 Hz, 1H), 7.60 (dd, *J* = 10.6, 3.1 Hz, 1H), 7.38 – 7.27 (m, 5H), 7.24 (t, *J* = 5.8 Hz, 1H), 7.12 – 6.96 (m, 2H), 4.98 (s, 2H), 4.51 (q, *J* = 7.5 Hz, 1H), 4.26 (ddd, *J* = 14.5, 8.6, 4.8 Hz, 2H), 4.15 (td, *J* = 9.4, 3.8 Hz, 1H), 2.97 (q, *J* = 6.4 Hz, 2H), 2.63 (dd, *J* = 17.1, 4.0 Hz, 1H), 2.44 (dd, *J* = 17.1, 10.2 Hz, 1H), 1.75 – 1.63 (m, 2H), 1.63 – 1.55 (m, 2H), 1.54 – 1.39 (m, 3H), 1.37 (s, 9H), 1.18 (d, *J* = 6.9 Hz, 3H), 0.95 – 0.83 (m, 15H), 0.50 (q, *J* = 7.9 Hz, 6H).

^{13}C NMR (101 MHz, DMSO-*d*$_6$): δ 171.76, 171.57, 171.06, 169.86, 162.06, 159.64, 155.94, 154.98, 137.12, 137.02, 133.69, 133.59, 128.15, 127.57, 113.54, 113.31, 112.21, 111.95, 105.31, 82.33, 78.02, 64.98, 53.15, 51.85, 51.52, 48.08, 29.07, 27.93, 25.78, 24.01, 23.04, 22.87, 21.31, 18.09, 7.17, 3.77.

^{19}F NMR (565 MHz, DMSO-*d*$_6$) δ -113.08.

HRMS (ESI-TOF): calculated for $C_{44}H_{64}BrFN_6NaO_8Si^+$ $[M+Na]^+$: 953.3615, found: 953.3623.

TLC: R$_f$ = 0.3 (10:1 DCM: Acetone, $Ce_2(SO_4)_3$ in phosphomolybdic acid).

$[\alpha]^{25}_D$: –18.0 (*c* = 1.0, $CHCl_3$)

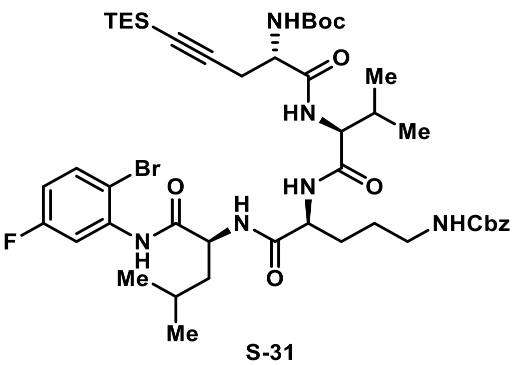

S-31

Purification by recrystallization (DCM/hexane) afforded compound **S-31** as a white solid (676 mg, 71% overall yield).

Compound S-31

Physical State: white solid

^{1}H NMR (400 MHz, DMSO-*d*$_6$): δ 9.46 (s, 1H), 8.22 (d, *J* = 7.8 Hz, 1H), 8.08 (d, *J* = 7.8 Hz, 1H), 7.76 – 7.53 (m, 3H), 7.33 (d, *J* = 6.6 Hz, 5H), 7.24 (d, *J* = 5.5 Hz, 1H), 7.17 (d, *J* = 8.7 Hz, 1H), 7.01 (td, *J* = 8.5, 3.0 Hz, 1H), 4.98 (s, 2H), 4.52 (q, *J* = 7.7 Hz, 1H), 4.30 (q, *J* = 7.5 Hz, 1H), 4.24 – 4.07 (m, 2H), 2.96 (q, *J* = 6.5

Hz, 2H), 2.60 (dd, J = 17.1, 4.2 Hz, 1H), 2.48 – 2.35 (m, 1H), 2.02 – 1.85 (m, 1H), 1.67 (q, J = 6.4 Hz, 2H), 1.62 – 1.53 (m, 2H), 1.54 – 1.42 (m, 2H), 1.37 (s, 9H), 1.20 – 1.05 (m, 1H), 0.97 – 0.74 (m, 21H), 0.50 (q, J = 7.9 Hz, 6H).
^{13}C NMR (101 MHz, DMSO-d_6): δ 171.59, 171.11, 170.31, 170.01, 162.06, 159.64, 155.94, 155.03, 137.12, 137.01, 133.69, 133.60, 128.14, 127.57, 112.16, 110.73, 105.35, 82.35, 78.08, 64.98, 56.92, 53.32, 51.89, 51.46, 35.61, 30.84, 29.04, 27.92, 25.87, 23.95, 22.93, 22.71, 21.23, 18.90, 17.45, 7.17, 3.77.
^{19}F NMR (565 MHz, DMSO-d_6) δ -113.09.
HRMS (ESI-TOF): calculated for $C_{46}H_{68}BrFN_6NaO_8SSi^+$ [M+Na]$^+$: 981.3928, found: 981.3929.
TLC: R$_f$ = 0.4 (10:1 DCM: Acetone, $Ce_2(SO_4)_3$ in phosphomolybdic acid).
[α]^{25}$_D$: –21.6 (c = 1.0, $CHCl_3$)

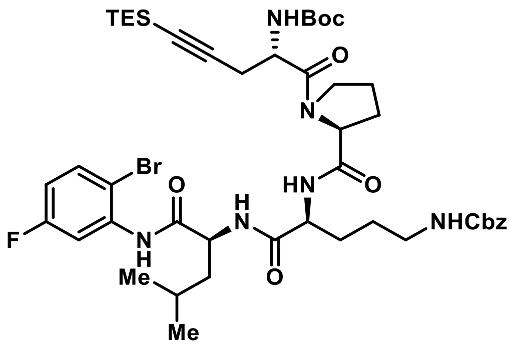

S-32

Purification by silica gel chromatography (40% EtOAc/hexane) to give the compound **S-32** (750 mg, 78% overall yield).

Compound S-32
Physical State: white solid
^{1}H NMR (400 MHz, CDCl$_3$): δ 8.52 (s, 1H), 8.12 (dd, J = 10.9, 3.0 Hz, 1H), 7.50 (d, J = 7.7 Hz, 1H), 7.45 (dd, J = 8.9, 5.8 Hz, 1H), 7.41 – 7.28 (m, 6H), 6.71 (ddd, J = 8.9, 7.6, 3.0 Hz, 1H), 5.33 (d, J = 7.5 Hz, 1H), 5.10 (s, 3H), 4.65 – 4.30 (m, 4H), 3.85 – 3.60 (m, 2H), 3.36 – 3.11 (m, 2H), 2.80 – 2.56 (m, 2H), 2.17 – 2.11 (m, 1H), 2.08 – 1.93 (m, 3H), 1.91 – 1.79 (m, 2H), 1.78 – 1.64 (m, 3H), 1.63 – 1.51 (m, 2H), 1.43 (s, 9H), 1.04 – 0.86 (m, 15H), 0.55 (q, J = 7.9 Hz, 6H).
^{13}C NMR (151 MHz, CDCl$_3$): δ 172.48, 171.48, 170.51, 162.90, 161.27, 157.18, 155.44, 137.01, 136.49, 132.96, 132.90, 128.62, 128.25, 128.17, 112.47, 112.32, 110.15, 108.21, 102.04, 86.01, 80.47, 66.95, 61.23, 53.01, 52.96, 52.13, 48.07, 39.98, 39.87, 28.46, 28.34, 28.19, 26.81, 25.37, 24.82, 23.95, 23.14, 21.66, 7.50, 4.36.
^{19}F NMR (565 MHz, CDCl$_3$) δ -111.73.
HRMS (ESI-TOF): calculated for $C_{47}H_{70}BrFN_6NaO_8SSi^+$ [M+Na]$^+$: 979.3771, found: 979.3711.
TLC: R$_f$ = 0.4 (1:1 Hexane:EtOAc, $Ce_2(SO_4)_3$ in phosphomolybdic acid).
[α]^{25}$_D$: –27.4 (c = 1.0, $CHCl_3$)

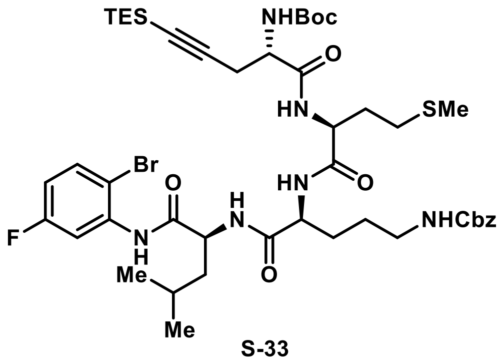

S-33

Purification by recrystallization (DCM/hexane) afforded compound **S-33** as a white solid (512 mg, 52% overall yield).

Compound S-33

Physical State: white solid

^{1}H NMR (400 MHz, DMSO-*d*$_6$): δ 9.48 (s, 1H), 8.21 (d, *J* = 7.9 Hz, 1H), 8.11 (d, *J* = 8.1 Hz, 1H), 7.97 – 7.84 (m, 1H), 7.67 (dd, *J* = 8.9, 5.9 Hz, 1H), 7.61 (dd, *J* = 10.5, 3.1 Hz, 1H), 7.37 – 7.27 (m, 5H), 7.27 – 7.21 (m, 1H), 7.11 (d, *J* = 8.6 Hz, 1H), 7.02 (td, *J* = 8.4, 3.1 Hz, 1H), 4.98 (s, 2H), 4.53 (q, *J* = 7.6 Hz, 1H), 4.39 – 4.22 (m, 2H), 4.19 – 4.02 (m, 1H), 2.97 (q, *J* = 6.3 Hz, 2H), 2.61 (dd, *J* = 17.1, 4.1 Hz, 1H), 2.47 – 2.35 (m, 3H), 2.00 (s, 3H), 1.93 – 1.84 (m, 1H), 1.82 – 1.74 (m, 1H), 1.73 – 1.62 (m, 2H), 1.62 – 1.55 (m, 2H), 1.52 – 1.42 (m, 2H), 1.37 (s, 9H), 1.17 – 1.08 (m, 1H), 0.98 – 0.81 (m, 15H), 0.51 (q, *J* = 7.9 Hz, 6H).

^{13}C NMR (101 MHz, DMSO-*d*$_6$): δ 171.58, 171.09, 170.48, 170.07, 162.06, 159.64, 155.95, 154.98, 137.12, 137.01, 133.69, 133.60, 128.15, 127.58, 113.32, 112.26, 105.27, 82.38, 78.06, 65.00, 53.23, 51.97, 51.64, 51.45, 32.13, 28.93, 27.93, 25.86, 23.99, 22.92, 21.27, 14.38, 7.18, 3.77.

^{19}F NMR (565 MHz, DMSO-*d*$_6$) δ -113.10.

HRMS (ESI-TOF): calculated for $C_{46}H_{68}BrFN_6NaO_8SSi^+$ [M+Na]$^+$: 1013.3648, found: 1013.3652.

TLC: R$_f$ = 0.5 (10:1 DCM: Acetone, $Ce_2(SO_4)_3$ in phosphomolybdic acid).

[α]$^{25}_D$: –22.9 (*c* = 1.0, $CHCl_3$)

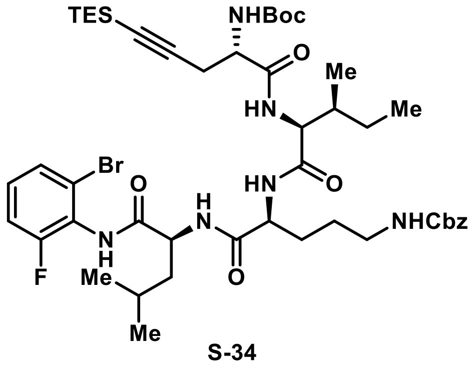

S-34

Purification by recrystallization (DCM/hexane) afforded compound **S-34** as a white solid (732 mg, 75% overall yield).

Compound S-34

Physical State: white solid

^{1}H NMR (400 MHz, DMSO-*d*$_6$): δ 9.79 (s, 1H), 8.04 (dd, *J* = 14.7, 8.2 Hz, 2H), 7.66 (d, *J* = 8.7 Hz, 1H), 7.50 (dd, *J* = 6.1, 3.1 Hz, 1H), 7.36 – 7.25 (m, 7H), 7.25 – 7.20 (m, 1H), 7.15 (d, *J* = 8.8 Hz, 1H), 4.98 (s, 2H), 4.58 (q, *J* = 7.8 Hz, 1H), 4.30 (q, *J* = 7.6 Hz, 1H), 4.25 – 4.08 (m, 2H), 2.96 (q, *J* = 6.4 Hz, 2H), 2.59 (dd, *J* =

17.1, 4.2 Hz, 1H), 2.44 (d, J = 19.3 Hz, 1H), 1.75 – 1.64 (m, 2H), 1.57 (t, J = 7.2 Hz, 2H), 1.51 – 1.44 (m, 1H), 1.37 (s, 9H), 1.10 – 0.99 (m, 1H), 0.96 – 0.85 (m, 15H), 0.83 – 0.72 (m, 6H), 0.51 (q, J = 7.9 Hz, 6H).
^{13}C NMR (101 MHz, DMSO-*d*$_6$): δ 171.01, 170.85, 170.28, 169.89, 159.29, 156.79, 155.94, 155.01, 137.02, 129.20, 128.15, 127.58, 127.55, 124.86, 124.71, 122.88, 115.43, 115.22, 105.40, 82.30, 78.05, 64.98, 56.41, 53.29, 51.80, 50.64, 41.03, 37.05, 29.14, 27.91, 25.76, 23.97, 23.84, 22.92, 22.69, 21.36, 14.99, 11.01, 7.18, 3.78.
^{19}F NMR (565 MHz, DMSO-*d*$_6$) δ -114.64.
HRMS (ESI-TOF): calculated for $C_{47}H_{70}BrFN_6NaO_8SSi^+$ [M+Na]$^+$: 995.4084, found: 995.4069.
TLC: R_f = 0.5 (10:1 DCM: Acetone, $Ce_2(SO_4)_3$ in phosphomolybdic acid).
$[\alpha]^{25}_D$: –27.3 (c = 1.0, $CHCl_3$)

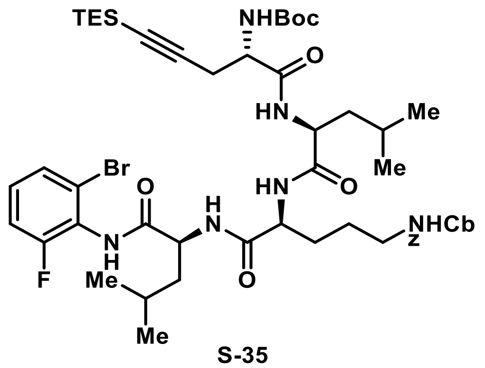

S-35

Purification by recrystallization (DCM/hexane) afforded compound **S-35** as a white solid (647 mg, 66% overall yield).

Compound S-35
Physical State: white solid
^{1}H NMR (600 MHz, DMSO-*d*$_6$): δ 9.75 (s, 1H), 7.96 (d, J = 8.2 Hz, 2H), 7.82 (d, J = 8.2 Hz, 1H), 7.47 (dd, J = 6.6, 2.6 Hz, 1H), 7.32 – 7.23 (m, 8H), 7.20 (t, J = 5.8 Hz, 1H), 7.03 (d, J = 8.8 Hz, 1H), 4.95 (s, 2H), 4.53 (td, J = 9.0, 5.6 Hz, 1H), 4.24 (q, J = 7.5 Hz, 2H), 4.10 (td, J = 9.6, 3.8 Hz, 1H), 2.93 (q, J = 6.7 Hz, 2H), 2.56 (dd, J = 17.0, 3.8 Hz, 1H), 2.41 (dd, J = 17.0, 10.3 Hz, 1H), 1.68 – 1.59 (m, 2H), 1.58 – 1.50 (m, 3H), 1.48 – 1.42 (m, 1H), 1.38 (t, J = 7.3 Hz, 3H), 1.34 (s, 9H), 0.91 – 0.87 (m, 12H), 0.84 (d, J = 6.4 Hz, 3H), 0.81 (d, J = 6.6 Hz, 3H), 0.78 (d, J = 6.6 Hz, 3H), 0.47 (q, J = 7.9 Hz, 6H).
^{13}C NMR (151 MHz, DMSO-*d*$_6$): δ 172.25, 171.74, 171.53, 170.67, 159.59, 157.92, 156.66, 155.66, 137.73, 130.01, 128.87, 128.29, 125.52, 123.58, 116.11, 106.08, 83.01, 78.71, 70.32, 65.69, 53.92, 52.49, 51.59, 51.42, 41.68, 41.60, 29.72, 28.62, 26.44, 24.68, 24.55, 23.59, 22.20, 22.12, 7.88, 4.48.
^{19}F NMR (565 MHz, DMSO-*d*$_6$) δ -114.60.
HRMS (ESI-TOF): calculated for $C_{47}H_{70}BrFN_6NaO_8SSi^+$ [M+Na]$^+$: 995.4084, found: 995.4084.
TLC: R_f = 0.6 (10:1 DCM: Acetone, $Ce_2(SO_4)_3$ in phosphomolybdic acid).
$[\alpha]^{25}_D$: –21.3 (c = 1.0, $CHCl_3$)

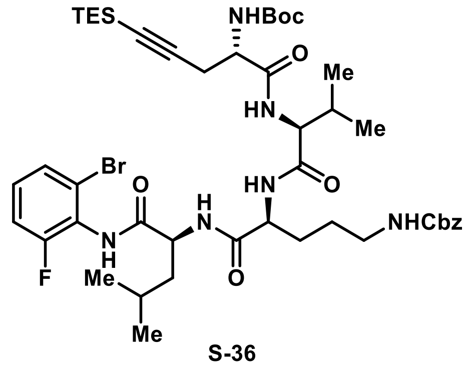

S-36

Purification by recrystallization (DCM/hexane) afforded compound **S-36** as a white solid (594 mg, 62% overall yield).

Compound S-36

Physical State: white solid

^{1}H NMR (400 MHz, DMSO-*d*$_6$): δ 9.78 (s, 1H), 8.14 – 7.94 (m, 2H), 7.74 – 7.56 (m, 1H), 7.55 – 7.45 (m, 1H), 7.43 – 7.03 (m, 10H), 4.98 (s, 2H), 4.68 – 4.46 (m, 1H), 4.35 – 4.09 (m, 3H), 3.04 – 2.90 (m, 2H), 2.64 – 2.53 (m, 1H), 2.44 (s, 1H), 1.95 (s, 1H), 1.73 – 1.52 (m, 4H), 1.51 – 1.42 (m, 2H), 1.37 (s, 9H), 0.97 – 0.74 (m, 21H), 0.59 – 0.33 (m, 6H).

^{13}C NMR (101 MHz, DMSO-*d*$_6$): δ 171.06, 170.86, 170.25, 169.99, 159.29, 156.79, 155.95, 155.01, 137.01, 129.28, 128.15, 127.57, 124.70, 122.88, 115.43, 115.22, 105.37, 82.34, 78.06, 64.99, 56.92, 53.31, 51.81, 50.68, 40.97, 35.61, 30.84, 29.09, 27.92, 25.77, 23.97, 22.91, 22.75, 21.36, 18.92, 17.45, 7.18, 3.77.

^{19}F NMR (565 MHz, DMSO-*d*$_6$) δ -114.63.

HRMS (ESI-TOF): calculated for $C_{46}H_{68}BrFN_6NaO_8Si^+$ $[M+Na]^+$: 981.3928, found: 981.3932.

TLC: R_f = 0.4 (10:1 DCM: Acetone, $Ce_2(SO_4)_3$ in phosphomolybdic acid).

$[\alpha]^{25}_{D}$: –17.8 (*c* = 1.0, $CHCl_3$)

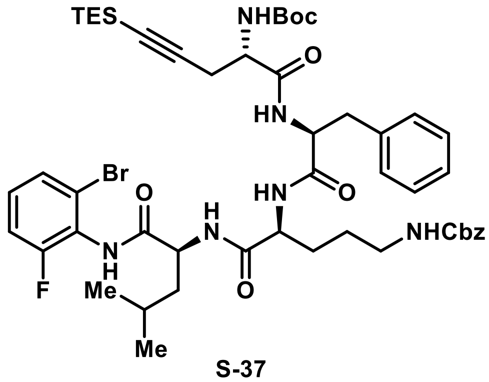

S-37

Purification by recrystallization (DCM/hexane) afforded compound **S-37** as a white solid (670 mg, 67% overall yield).

Compound S-37

Physical State: white solid

^{1}H NMR (600 MHz, DMSO-*d*$_6$): δ 9.79 (s, 1H), 8.13 (d, *J* = 8.0 Hz, 1H), 8.06 (d, *J* = 8.3 Hz, 1H), 7.81 (d, *J* = 8.1 Hz, 1H), 7.48 (d, *J* = 7.2 Hz, 1H), 7.33 – 7.23 (m, 8H), 7.15 (q, *J* = 7.7 Hz, 5H), 6.96 (d, *J* = 9.0 Hz, 1H), 4.96 (d, *J* = 7.2 Hz, 2H), 4.58 (q, *J* = 7.9 Hz, 1H), 4.52 – 4.42 (m, 1H), 4.32 – 4.25 (m, 1H), 4.08 (d, *J* = 10.4 Hz, 1H), 2.95 (dd, *J* = 15.8, 9.9 Hz, 3H), 2.74 (dd, *J* = 13.9, 8.8 Hz, 1H), 2.54 – 2.48 (m, 1H), 2.32 (dd,

J = 16.8, 10.6 Hz, 1H), 1.73 – 1.62 (m, 2H), 1.60 – 1.52 (m, 2H), 1.50 – 1.44 (m, 1H), 1.42 – 1.36 (m, 2H), 1.32 (s, 9H), 0.93 – 0.84 (m, 15H), 0.47 (q, J = 7.9 Hz, 6H).

^{13}C NMR (151 MHz, DMSO-*d*$_6$): δ 171.80, 171.57, 171.02, 170.65, 162.87, 157.95, 156.69, 155.54, 137.91, 137.74, 129.90, 128.86, 128.47, 128.29, 126.77, 125.56, 116.12, 115.98, 106.04, 82.98, 78.69, 65.72, 54.13, 53.98, 52.49, 51.45, 41.69, 38.18, 30.09, 28.63, 26.43, 24.78, 23.81, 23.58, 22.19, 7.88, 4.48.

^{19}F NMR (565 MHz, DMSO-*d*$_6$) δ -114.63.

HRMS (ESI-TOF): calculated for $C_{50}H_{68}BrFN_6NaO_8Si^+$ [M+Na]$^+$: 1029.3928, found: 1029.3931.

TLC: R_f = 0.5 (10:1 DCM: Acetone, $Ce_2(SO_4)_3$ in phosphomolybdic acid).

[α]$^{25}_D$: –21.6 (c = 1.0, $CHCl_3$)

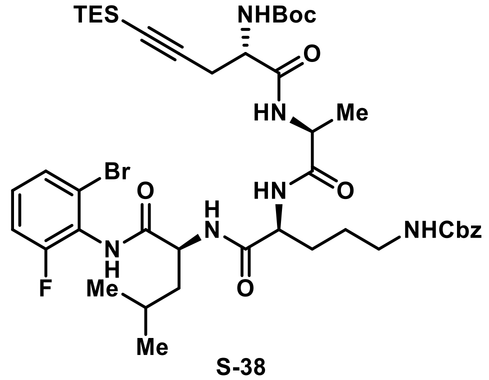

S-38

Purification by recrystallization (DCM/hexane) afforded compound **S-38** as a white solid (636 mg, 68% yield overall yield).

Compound S-38

Physical State: white solid

^{1}H NMR (600 MHz, DMSO-*d*$_6$): δ 9.76 (s, 1H), 8.01 – 7.88 (m, 3H), 7.47 (dd, J = 6.6, 2.6 Hz, 1H), 7.32 – 7.24 (m, 7H), 7.19 (t, J = 5.9 Hz, 1H), 7.01 (d, J = 8.9 Hz, 1H), 4.95 (s, 2H), 4.53 (td, J = 9.0, 5.3 Hz, 1H), 4.22 (tt, J = 14.5, 7.6 Hz, 2H), 4.11 (td, J = 9.7, 3.8 Hz, 1H), 2.92 (q, J = 6.6 Hz, 2H), 2.59 (dd, J = 16.9, 3.8 Hz, 1H), 2.40 (dd, J = 17.0, 10.2 Hz, 1H), 1.69 – 1.60 (m, 2H), 1.59 – 1.51 (m, 2H), 1.47 – 1.42 (m, 1H), 1.40 – 1.35 (m, 2H), 1.34 (s, 9H), 1.14 (d, J = 7.0 Hz, 3H), 0.89 (t, J = 7.9 Hz, 12H), 0.85 (d, J = 6.5 Hz, 3H), 0.47 (q, J = 7.9 Hz, 6H).

^{13}C NMR (151 MHz, DMSO-*d*$_6$): Unknown NMR (151 MHz, DMSO-*D*$_6$) δ 172.42, 171.75, 170.51, 159.60, 157.93, 156.67, 155.68, 137.74, 129.95, 128.87, 128.29, 125.42, 123.59, 115.98, 106.03, 88.92, 83.05, 78.73, 65.70, 53.84, 52.53, 51.43, 48.75, 41.69, 40.57, 40.45, 40.31, 40.17, 40.03, 39.89, 39.75, 39.61, 29.83, 28.64, 26.39, 24.74, 23.77, 23.54, 22.18, 18.83, 7.88, 4.48.

^{19}F NMR (565 MHz, DMSO-*d*$_6$) δ -114.64.

HRMS (ESI-TOF): calculated for $C_{44}H_{64}BrFN_6NaO_8Si^+$ [M+Na]$^+$: 953.3615, found: 953.3630.

TLC: R_f = 0.3 (10:1 DCM: Acetone, $Ce_2(SO_4)_3$ in phosphomolybdic acid).

[α]$^{25}_D$: –9.3 (c = 1.0, $CHCl_3$)

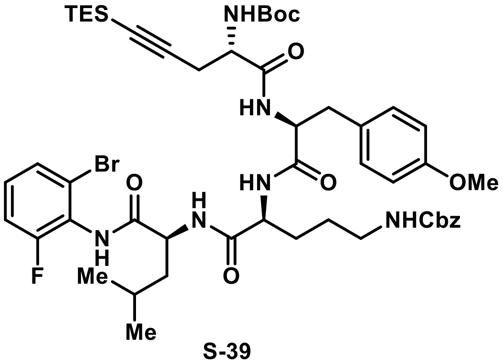

S-39

Purification by recrystallization (DCM/hexane) afforded compound **S-39** as a white solid (550 mg, 53% yield overall yield).

Compound S-39

Physical State: white solid

^{1}H NMR (400 MHz, DMSO-*d*$_6$): δ 9.83 (s, 1H), 8.12 (dd, *J* = 17.4, 8.1 Hz, 2H), 7.80 (d, *J* = 8.1 Hz, 1H), 7.56 – 7.45 (m, 1H), 7.40 – 7.19 (m, 9H), 7.10 (d, *J* = 8.0 Hz, 2H), 7.00 (d, *J* = 8.9 Hz, 1H), 6.75 (d, *J* = 8.1 Hz, 2H), 4.98 (d, *J* = 5.1 Hz, 2H), 4.61 (q, *J* = 8.3 Hz, 1H), 4.45 (t, *J* = 6.8 Hz, 1H), 4.37 – 4.26 (m, 1H), 4.17 – 4.04 (m, 1H), 3.68 (s, 3H), 2.95 (td, *J* = 14.7, 13.6, 8.5 Hz, 3H), 2.71 (q, *J* = 9.6, 8.5 Hz, 1H), 2.55 (dd, *J* = 18.8, 5.4 Hz, 1H), 2.36 (dd, *J* = 17.1, 10.6 Hz, 1H), 1.68 (dq, *J* = 14.8, 6.9 Hz, 2H), 1.61 (t, *J* = 6.9 Hz, 2H), 1.53 – 1.46 (m, 1H), 1.45 – 1.40 (m, 1H), 1.35 (s, 9H), 0.92 (q, *J* = 7.7, 6.3 Hz, 15H), 0.50 (q, *J* = 7.9 Hz, 6H).

^{13}C NMR (101 MHz, DMSO-*d*$_6$): δ 171.12, 170.86, 170.34, 169.92, 159.30, 157.58, 156.80, 155.98, 154.84, 137.00, 130.20, 129.23, 128.99, 128.16, 127.59, 124.85, 124.69, 122.88, 115.44, 115.24, 113.16, 105.37, 82.26, 77.98, 65.01, 54.65, 53.65, 53.24, 51.73, 50.72, 40.97, 36.60, 29.39, 27.92, 25.73, 24.08, 23.08, 22.87, 21.47, 7.18, 3.76.

^{19}F NMR (565 MHz, DMSO-*d*$_6$) δ -114.64.

HRMS (ESI-TOF): calculated for $C_{51}H_{70}BrFN_6NaO_9Si^+$ [M+Na]$^+$: 1059.4033, found: 1059.4034.

TLC: R$_f$ = 0.6 (10:1 DCM: Acetone, $Ce_2(SO_4)_3$ in phosphomolybdic acid).

[α]^{25}$_D$: –19.5 (*c* = 1.0, $CHCl_3$)

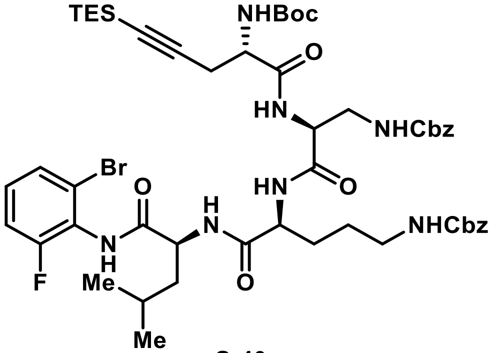

S-40

Purification by recrystallization (DCM/hexane) afforded compound **S-40** as a white solid (506 mg, 46% overall yield).

Compound S-40

Physical State: white solid

^{1}H NMR (400 MHz, DMSO-*d*$_6$): δ 9.79 (s, 1H), 8.07 (d, *J* = 7.9 Hz, 2H), 7.98 (d, *J* = 7.5 Hz, 1H), 7.53 – 7.45 (m, 1H), 7.43 – 7.22 (m, 13H), 7.13 (d, *J* = 6.1 Hz, 1H), 7.05 (d, *J* = 8.6 Hz, 1H), 4.99 (d, *J* = 11.4 Hz,

4H), 4.58 (q, J = 7.9 Hz, 1H), 4.24 (dt, J = 49.7, 11.9 Hz, 3H), 3.31 (s, 2H), 2.96 (q, J = 6.9, 6.4 Hz, 2H), 2.66 (d, J = 15.2 Hz, 1H), 2.48 – 2.36 (m, 1H), 1.76 – 1.64 (m, 2H), 1.64 – 1.54 (m, 2H), 1.53 – 1.44 (m, 2H), 1.37 (s, 9H), 1.19 – 1.03 (m, 1H), 0.99 – 0.81 (m, 15H), 0.50 (q, J = 7.9 Hz, 6H).
^{13}C NMR (101 MHz, DMSO-d_6): δ 171.21, 170.87, 170.20, 169.26, 162.15, 159.29, 156.80, 156.16, 155.97, 154.99, 137.00, 136.77, 129.30, 128.14, 127.59, 127.53, 124.84, 124.69, 122.89, 115.43, 115.22, 105.28, 82.36, 78.10, 65.34, 65.02, 53.24, 52.13, 50.81, 40.83, 35.62, 29.00, 27.93, 25.67, 24.01, 23.03, 22.81, 21.44, 7.18, 3.76.
^{19}F NMR (565 MHz, DMSO-d_6) δ -114.61.
HRMS (ESI-TOF): calculated for $C_{52}H_{71}BrFN_7NaO_{10}Si^+$ [M+Na]$^+$: 1102.4091, found: 1102.4099.
TLC: R_f = 0.5 (5:1 DCM: Acetone, $Ce_2(SO_4)_3$ in phosphomolybdic acid).
[α]^{25}$_D$: –19.7 (c = 1.0, $CHCl_3$)

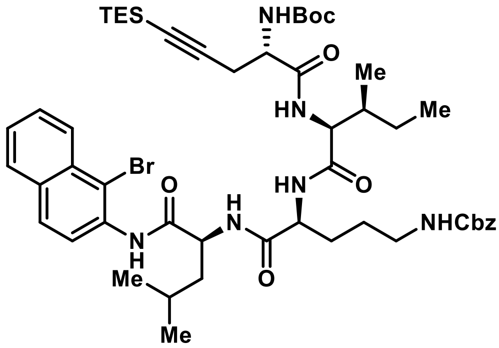

S-41

Purification by recrystallization (DCM/hexane) afforded compound **S-41** as a white solid (631 mg, 63% overall yield).

Compound S-41
Physical State: white solid
^{1}H NMR (400 MHz, DMSO-d_6): δ 9.77 (s, 1H), 8.27 – 8.03 (m, 3H), 8.01 – 7.87 (m, 2H), 7.76 – 7.51 (m, 4H), 7.44 – 7.03 (m, 7H), 4.97 (s, 2H), 4.59 (q, J = 7.7 Hz, 1H), 4.40 – 4.27 (m, 1H), 4.19 (dd, J = 18.9, 10.8 Hz, 2H), 3.05 – 2.92 (m, 2H), 2.60 (d, J = 16.6 Hz, 1H), 2.47 – 2.35 (m, 1H), 1.77 – 1.58 (m, 5H), 1.58 – 1.41 (m, 3H), 1.37 (s, 9H), 1.14 – 0.99 (m, 2H), 0.99 – 0.83 (m, 15H), 0.83 – 0.68 (m, 6H), 0.57 – 0.39 (m, 6H).
^{13}C NMR (101 MHz, DMSO-d_6): δ 171.38, 171.04, 170.35, 169.97, 155.94, 155.02, 136.99, 134.43, 131.66, 131.48, 128.12, 128.11, 127.79, 127.78, 127.53, 126.14, 124.64, 124.61, 115.78, 105.37, 82.29, 78.04, 64.99, 56.46, 53.31, 51.89, 51.36, 40.49, 37.03, 29.15, 29.12, 27.89, 25.84, 24.03, 22.95, 22.69, 21.36, 15.00, 11.00, 7.16, 3.77.
HRMS (ESI-TOF): calculated for $C_{51}H_{73}BrN_6NaO_8Si^+$ [M+Na]$^+$: 1027.4335, found: 1027.4309.
TLC: R_f = 0.4 (10:1 DCM: Acetone, $Ce_2(SO_4)_3$ in phosphomolybdic acid).
[α]^{25}$_D$: –30.2 (c = 1.0, $CHCl_3$)

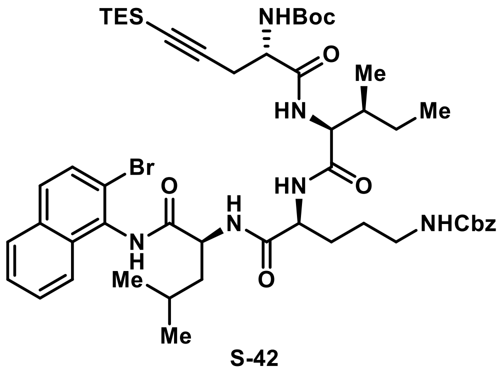

S-42

Purification by recrystallization (DCM/hexane) afforded compound **S-42** as a white solid (643 mg, 64% overall yield).

Compound S-42
Physical State: white solid
^{1}H NMR (400 MHz, DMSO-*d*$_6$): δ 10.06 (s, 1H), 8.22 – 8.04 (m, 2H), 8.02 – 7.79 (m, 4H), 7.78 – 7.63 (m, 2H), 7.61 – 7.47 (m, 2H), 7.42 – 7.11 (m, 6H), 4.95 (s, 2H), 4.62 (s, 1H), 4.41 – 4.05 (m, 3H), 3.02 – 2.81 (m, 4H), 2.78 – 2.66 (m, 2H), 2.61 – 2.54 (m, 1H), 1.86 – 1.60 (m, 5H), 1.57 – 1.46 (m, 2H), 1.36 (s, 9H), 1.07 – 0.69 (m, 21H), 0.60 – 0.31 (m, 6H).
^{13}C NMR (101 MHz, DMSO-*d*$_6$): δ 171.24, 170.39, 169.94, 164.71, 162.13, 155.91, 142.77, 136.97, 134.01, 132.45, 131.49, 129.28, 128.59, 128.13, 127.91, 127.56, 127.06, 126.40, 123.54, 120.28, 105.39, 82.32, 78.06, 64.98, 53.31, 51.84, 51.24, 40.54, 37.03, 35.59, 29.10, 27.91, 25.75, 24.07, 23.91, 22.95, 22.68, 21.41, 15.03, 11.01, 7.18, 3.77.
HRMS (ESI-TOF): calculated for $C_{51}H_{73}BrN_6NaO_8Si^+$ $[M+Na]^+$: 1027.4335, found: 1027.4319.
TLC: R$_f$ = 0.5 (10:1 DCM: Acetone, $Ce_2(SO_4)_3$ in phosphomolybdic acid).
$[\alpha]^{25}_D$: –31.8 (*c* = 1.0, $CHCl_3$)

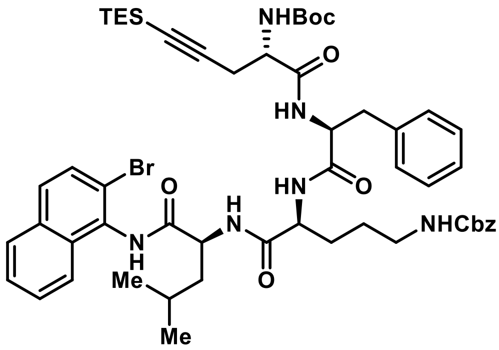

S-43

Purification by recrystallization (DCM/hexane) afforded compound **S-43** as a white solid (628 mg, 61% overall yield).

Compound S-43
Physical State: white solid
^{1}H NMR (400 MHz, DMSO-*d*$_6$): δ 10.10 (s, 1H), 8.22 (d, *J* = 7.5 Hz, 2H), 8.10 – 7.77 (m, 5H), 7.77 – 7.67 (m, 1H), 7.54 (d, *J* = 7.3 Hz, 2H), 7.40 – 7.11 (m, 11H), 7.01 (d, *J* = 8.9 Hz, 1H), 4.95 (s, 2H), 4.72 – 4.59 (m, 1H), 4.59 – 4.44 (m, 1H), 4.43 – 4.23 (m, 1H), 4.22 – 3.96 (m, 1H), 3.04 – 2.91 (m, 3H), 2.82 – 2.75 (m, 1H), 2.60 – 2.52 (m, 1H), 2.42 – 2.26 (m, 1H), 1.86 – 1.62 (m, 4H), 1.57 – 1.48 (m, 1H), 1.35 (s, 9H), 1.23 (s, 1H), 1.01 – 0.79 (m, 15H), 0.58 – 0.36 (m, 6H).

^{13}C NMR (101 MHz, DMSO-d_6): δ 171.46, 171.25, 170.36, 170.00, 162.18, 155.96, 155.96, 154.84, 137.16, 136.96, 132.47, 131.50, 129.18, 128.62, 128.14, 127.92, 127.78, 127.56, 127.08, 126.42, 126.09, 123.54, 120.31, 105.32, 82.29, 78.01, 65.00, 53.45, 53.28, 51.81, 51.32, 40.49, 37.45, 35.62, 29.34, 27.92, 25.69, 24.17, 23.09, 22.90, 21.54, 7.18, 3.76.
HRMS (ESI-TOF): calculated for $C_{51}H_{73}BrN_6NaO_8Si^+$ $[M+Na]^+$: 1061.4178, found: 1061.4189.
TLC: R_f = 0.5 (10:1 DCM: Acetone, $Ce_2(SO_4)_3$ in phosphomolybdic acid).
$[\alpha]^{25}_D$: –29.1 (c = 1.0, $CHCl_3$)

Synthesis of acylated aromatic amine derivatives

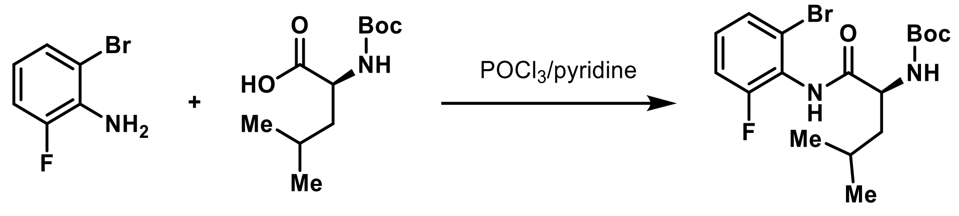

To a solution of 2-bromo-6-fluoroaniline **S-55** (1.90 g, 10.0 mmol, 1.0 equiv.) and Boc-*L*-leucine **34** (2.31 g, 10.0 mmol, 1.0 equiv.) in 30 mL pyridine was added $POCl_3$ (1.0 mL, 11.0 mmol, 1.1 equiv.) dropwise over 30 min at –10 °C. Then the reaction mixture was allowed to stir at –10 °C for another 30 min. Once the starting material was consumed completely, the mixture was quenched by saturated *aq.* $NaHCO_3$ (50 mL) and extracted with DCM (3 X 50 mL). The organic layers were combined, washed by saturated *aq.* NaCl, dried over Na_2SO_4 and removed under reduced pressure to give the residue; the residue was purified by silica gel chromatography (10% EtOAc/hexane) to give the compound **S-56** (3.6 g, 89% yield).

Compound S-56
Physical State: white solid
^{1}H NMR (600 MHz, $CDCl_3$): δ 8.27 – 7.69 (m, 1H), 7.34 (d, *J* = 7.9 Hz, 1H), 7.20 – 6.99 (m, 2H), 5.51 – 4.93 (m, 1H), 4.61 – 4.04 (m, 1H), 1.84 – 1.72 (m, 2H), 1.65 – 1.52 (m, 1H), 1.43 (s, 9H), 0.96 (dd, *J* = 12.2, 6.1 Hz, 6H).
^{13}C NMR (151 MHz, $CDCl_3$): δ 171.19, 159.09, 157.41, 156.17, 128.78, 128.73, 128.22, 128.19, 124.37, 124.27, 122.24, 115.66, 115.52, 80.47, 53.08, 40.73, 28.40, 24.85, 22.96, 22.27.
^{19}F NMR (565 MHz, $CDCl_3$) δ -113.29.
HRMS (ESI-TOF): calculated for $C_{17}H_{24}FBrN_2NaO_3^+$ $[M+Na]^+$: 425.0847, found: 425.0850.
TLC: R_f = 0.5 (10:1 Hexane:EtOAc, $Ce_2(SO_4)_3$ in phosphomolybdic acid).
$[\alpha]^{25}_D$: –80.2 (*c* = 1.0, $CHCl_3$)

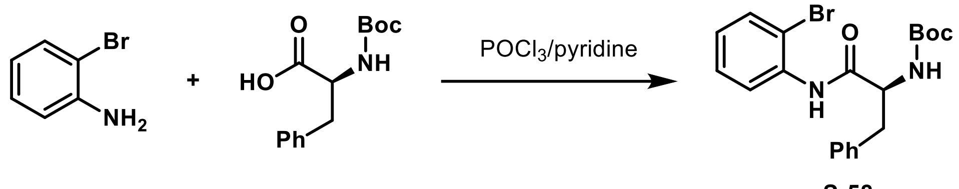

To a solution of 2-bromoaniline **33** (688 mg, 4.0 mmol, 1.0 equiv.) and Boc-*L*-phenylalanine **S-57** (925 mg, 4.0 mmol, 1.0 equiv.) in 12 mL pyridine was added $POCl_3$ (0.4 mL, 4.4 mmol, 1.1 equiv.) dropwise over 30 min at –10 °C. Then the reaction mixture was allowed to stir at –10 °C for another 30 min. Once the starting material was consumed completely, the mixture was quenched by saturated *aq.* $NaHCO_3$ (20 mL) and extracted with DCM (3 X 20 mL). The organic layers were combined, washed by saturated *aq.* NaCl, dried over Na_2SO_4 and removed under reduced pressure to give the residue; the residue was purified by silica gel chromatography (5% EtOAc/hexane) to give the compound **S-58** (1.3 g, 77% yield).

Compound S-58
Physical State: white solid
^{1}H NMR (400 MHz, $CDCl_3$): δ 8.34 (t, *J* = 7.8 Hz, 2H), 7.50 (dd, *J* = 8.0, 1.4 Hz, 1H), 7.35 – 7.22 (m, 6H), 6.98 (td, *J* = 7.7, 1.6 Hz, 1H), 5.12 (s, 1H), 4.58 (s, 1H), 3.38 – 2.92 (m, 2H), 1.43 (s, 9H).

^{13}C NMR (101 MHz, CDCl$_3$): δ 169.14, 154.82, 135.66, 134.61, 131.66, 128.63, 128.27, 127.70, 126.56, 124.79, 121.13, 112.97, 80.04, 56.05, 37.39, 27.66.
HRMS (ESI-TOF): calculated for $C_{17}H_{25}BrN_2NaO_3^+$ [M+Na]$^+$: 441.0784, found: 441.0790.
TLC: R$_f$ = 0.5 (15:1 Hexane:EtOAc, $Ce_2(SO_4)_3$ in phosphomolybdic acid).
[α]$^{25}_D$: –81.4 (*c* = 1.0, $CHCl_3$)

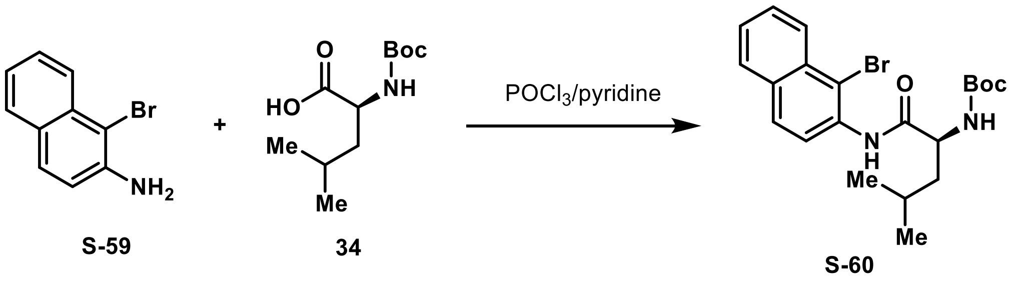

To a solution of 1-bromonaphthalen-2-amine **S-59** (888 mg, 4.0 mmol, 1.0 equiv.) and Boc-*L*-leucine **34** (925 mg, 4.0 mmol, 1.0 equiv.) in 12 mL pyridine was added $POCl_3$ (0.4 mL, 4.4 mmol, 1.1 equiv.) dropwise over 30 min at –10 °C. Then the reaction mixture was allowed to stir at –10 °C for another 30 min. Once the starting material was consumed completely, the mixture was quenched by saturated *aq.* $NaHCO_3$ (20 mL) and extracted with DCM (3 X 20 mL). The organic layers were combined, washed by saturated *aq.* NaCl, dried over Na_2SO_4 and removed under reduced pressure to give the residue; the residue was purified by silica gel chromatography (10% EtOAc/hexane) to give the compound **S-60** (1.17 g, 67% yield).

Compound S-60
Physical State: white solid
^{1}H NMR (400 MHz, CDCl$_3$): δ 8.82 (s, 1H), 8.48 (d, *J* = 9.0 Hz, 1H), 8.16 (d, *J* = 8.5 Hz, 1H), 7.80 (d, *J* = 8.9 Hz, 2H), 7.57 (ddd, *J* = 8.3, 6.8, 1.3 Hz, 1H), 7.46 (ddd, *J* = 8.1, 6.9, 1.2 Hz, 1H), 5.07 (d, *J* = 7.7 Hz, 1H), 4.54 – 4.09 (m, 1H), 1.96 – 1.75 (m, 2H), 1.63 (ddd, *J* = 13.5, 9.6, 5.2 Hz, 1H), 1.49 (s, 9H), 1.02 (t, *J* = 6.2 Hz, 6H).
^{13}C NMR (151 MHz, CDCl$_3$): δ 171.27, 155.84, 134.19, 132.06, 131.70, 128.39, 128.24, 127.81, 126.72, 125.67, 120.83, 112.04, 80.71, 54.35, 41.00, 28.46, 25.04, 23.12, 21.99.
HRMS (ESI-TOF): calculated for $C_{21}H_{27}BrN_2NaO_3^+$ [M+Na]$^+$: 457.1097, found: 457.1109.
TLC: R$_f$ = 0.5 (10:1 Hexane:EtOAc, $Ce_2(SO_4)_3$ in phosphomolybdic acid).
[α]$^{25}_D$: –77.1 (*c* = 1.0, $CHCl_3$)

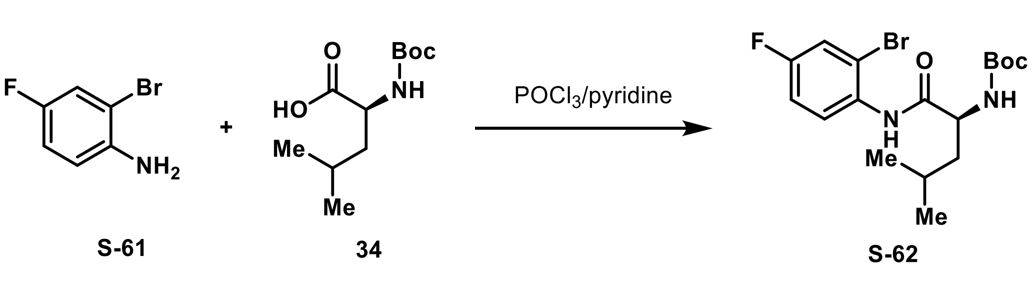

To a solution of 2-bromo-4-fluoroaniline **S-61** (760 mg, 4.0 mmol, 1.0 equiv.) and Boc-*L*-leucine **34** (925 mg, 4.0 mmol, 1.0 equiv.) in 12 mL pyridine was added $POCl_3$ (0.4 mL, 4.4 mmol, 1.1 equiv.) dropwise over 30 min at –10 °C. Then the reaction mixture was allowed to stir at –10 °C for another 30 min. Once the starting material was consumed completely, the mixture was quenched by saturated *aq.* $NaHCO_3$ (20 mL) and extracted with DCM (3 X 20 mL). The organic layers were combined, washed by saturated *aq.* NaCl, dried

over Na_2SO_4 and removed under reduced pressure to give the residue; the residue was purified by silica gel chromatography (10% EtOAc/hexane) to give the compound **S-62** (1.37 g, 85% yield).

Compound S-62

Physical State: white solid

^{1}H NMR (400 MHz, CDCl$_3$): δ 8.47 (s, 1H), 8.27 (dd, J = 9.1, 5.6 Hz, 1H), 7.28 (dd, J = 8.0, 2.7 Hz, 1H), 7.03 (ddd, J = 9.1, 7.8, 2.9 Hz, 1H), 5.27 – 4.81 (m, 1H), 4.30 (s, 1H), 1.87 – 1.68 (m, 2H), 1.62 – 1.53 (m, 1H), 1.46 (s, 9H), 0.98 (t, J = 6.7 Hz, 6H).

^{13}C NMR (101 MHz, CDCl$_3$): δ 170.33, 159.17, 156.70, 155.13, 131.39, 122.45, 118.84, 118.59, 114.60, 114.38, 113.21, 80.02, 53.37, 40.06, 27.70, 27.34, 24.25, 22.35, 21.20.

^{19}F NMR (565 MHz, CDCl$_3$) δ -115.89.

HRMS (ESI-TOF): calculated for $C_{17}H_{24}FBrN_2NaO_3^+$ [M+Na]$^+$: 425.0847, found: 425.0850.

TLC: R$_f$ = 0.6 (10:1 Hexane:EtOAc, $Ce_2(SO_4)_3$ in phosphomolybdic acid).

[α]$^{25}_D$: –73.9 (c = 1.0, CHCl$_3$)

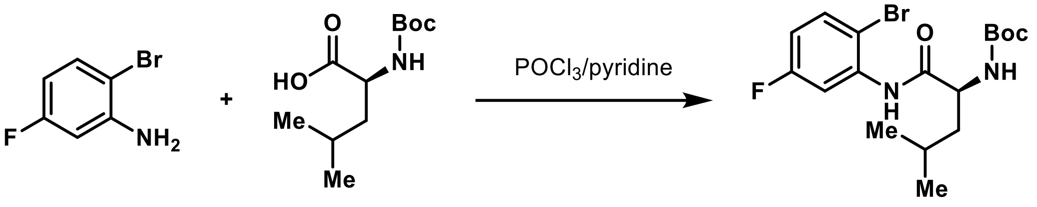

S-63 34 S-64

To a solution of 2-bromo-5-fluoroaniline **S-63** (760 mg, 4.0 mmol, 1.0 equiv.) and Boc-*L*-leucine **34** (925 mg, 4.0 mmol, 1.0 equiv.) in 12 mL pyridine was added $POCl_3$ (0.4 mL, 4.4 mmol, 1.1 equiv.) dropwise over 30 min at −10 °C. Then the reaction mixture was allowed to stir at −10 °C for another 30 min. Once the starting material was consumed completely, the mixture was quenched by saturated *aq.* $NaHCO_3$ (20 mL) and extracted with DCM (3 X 20 mL). The organic layers were combined, washed by saturated *aq.* NaCl, dried over Na_2SO_4 and removed under reduced pressure to give the residue; the residue was purified by silica gel chromatography (5% EtOAc/hexane) to give the compound **S-64** (1.25 g, 78% yield).

Compound S-64

Physical State: white solid

^{1}H NMR (400 MHz, CDCl$_3$): δ 8.63 (s, 1H), 8.25 (dd, J = 11.0, 3.0 Hz, 1H), 7.46 (dd, J = 8.9, 5.7 Hz, 1H), 6.72 (ddd, J = 8.9, 7.6, 3.0 Hz, 1H), 5.00 (s, 1H), 4.30 (s, 1H), 1.85 – 1.71 (m, 2H), 1.57 (ddd, J = 13.4, 9.6, 5.1 Hz, 1H), 1.46 (s, 9H), 0.98 (t, J = 6.8 Hz, 6H).

^{13}C NMR (101 MHz, CDCl$_3$): δ 170.49, 162.71, 160.27, 136.02, 132.19, 132.10, 111.59, 111.36, 108.54, 108.25, 106.59, 106.56, 80.15, 53.56, 39.95, 27.68, 24.26, 22.34, 21.15.

^{19}F NMR (565 MHz, CDCl$_3$) δ -111.18.

HRMS (ESI-TOF): calculated for $C_{17}H_{24}FBrN_2NaO_3^+$ [M+Na]$^+$: 425.0847, found: 425.0854.

TLC: R$_f$ = 0.6 (10:1 Hexane:EtOAc, $Ce_2(SO_4)_3$ in phosphomolybdic acid).

[α]$^{25}_D$: –78.3 (c = 1.0, CHCl$_3$)

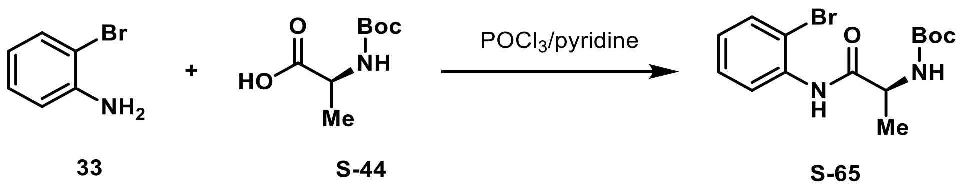

To a solution of 2-bromoaniline **33** (688 mg, 4.0 mmol, 1.0 equiv.) and Boc-*L*-alanine **S-44** (756 mg, 4.0 mmol, 1.0 equiv.) in 12 mL pyridine was added $POCl_3$ (0.4 mL, 4.4 mmol, 1.1 equiv.) dropwise over 30 min at −10 °C. Then the reaction mixture was allowed to stir at −10 °C for another 30 min. Once the starting material was consumed completely, the mixture was quenched by saturated *aq.* $NaHCO_3$ (20 mL) and extracted with DCM (3 X 20 mL). The organic layers were combined, washed by saturated *aq.* NaCl, dried over Na_2SO_4 and removed under reduced pressure to give the residue; the residue was purified by silica gel chromatography (5% EtOAc/hexane) to give the compound **S-65** (1.03 g, 75% yield).

Compound S-65
Physical State: white solid
^{1}H NMR (400 MHz, $CDCl_3$): δ 8.57 (s, 1H), 8.34 (dd, *J* = 8.3, 1.6 Hz, 1H), 7.52 (dd, *J* = 8.0, 1.5 Hz, 1H), 7.37 – 7.24 (m, 1H), 6.97 (td, *J* = 7.7, 1.6 Hz, 1H), 5.29 – 5.02 (m, 1H), 4.37 (s, 1H), 1.50 – 1.44 (m, 12H).
^{13}C NMR (101 MHz, $CDCl_3$): δ 170.38, 154.91, 134.87, 131.67, 127.70, 124.69, 121.19, 113.01, 80.03, 27.73, 27.37, 17.27.
HRMS (ESI-TOF): calculated for $C_{14}H_{19}BrN_2NaO_3^+$ [M+Na]$^+$: 365.0471, found: 365.0475.
TLC: R_f = 0.4 (10:1 Hexane:EtOAc, $Ce_2(SO_4)_3$ in phosphomolybdic acid).
$[\alpha]^{25}_D$: –75.6 (*c* = 1.0, $CHCl_3$)

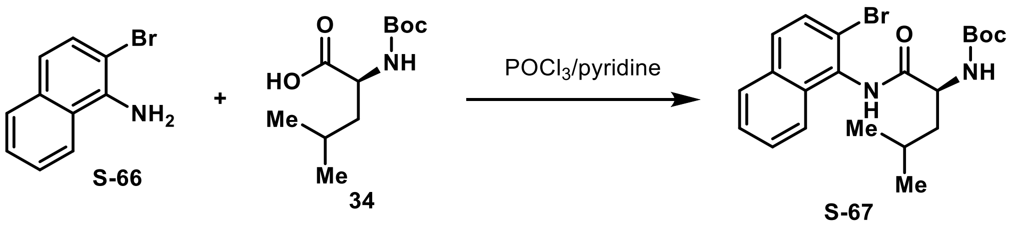

To a solution of 2-bromonaphthalen-1-amine **S-66** (888 mg, 4.0 mmol, 1.0 equiv.) and Boc-*L*-leucine **34** (925 mg, 4.0 mmol, 1.0 equiv.) in 12 mL pyridine was added $POCl_3$ (0.4 mL, 4.4 mmol, 1.1 equiv.) dropwise over 30 min at −10 °C. Then the reaction mixture was allowed to stir at −10 °C for another 30 min. Once the starting material was consumed completely, the mixture was quenched by saturated *aq.* $NaHCO_3$ (20 mL) and extracted with DCM (3 X 20 mL). The organic layers were combined, washed by saturated *aq.* NaCl, dried over Na_2SO_4 and removed under reduced pressure to give the residue; the residue was purified by silica gel chromatography (20% EtOAc/hexane) to give the compound **S-67** (913 mg, 53% yield).

Compound S-67
Physical State: white solid
^{1}H NMR (400 MHz, $CDCl_3$): δ 8.42 (s, 1H), 7.74 (dd, *J* = 25.3, 8.2 Hz, 2H), 7.62 – 7.49 (m, 2H), 7.46 – 7.29 (m, 2H), 5.89 – 5.07 (m, 1H), 4.71 – 4.14 (m, 1H), 1.93 – 1.82 (m, 2H), 1.68 (td, *J* = 9.5, 6.7 Hz, 1H), 1.50 (s, 9H), 1.10 – 0.90 (m, 6H).
^{13}C NMR (101 MHz, $CDCl_3$): δ 171.27, 155.62, 132.28, 130.74, 130.67, 128.68, 128.24, 127.33, 126.55, 125.71, 122.89, 119.62, 79.73, 52.80, 39.99, 27.78, 24.21, 22.36, 21.57.
HRMS (ESI-TOF): calculated for $C_{21}H_{27}BrN_2NaO_3^+$ [M+Na]$^+$: 457.1097, found: 457.1098.

TLC: R_f = 0.6 (3:1 Hexane:EtOAc, $Ce_2(SO_4)_3$ in phosphomolybdic acid).
$[\alpha]^{25}_D$: –78.6 (c = 1.0, $CHCl_3$)

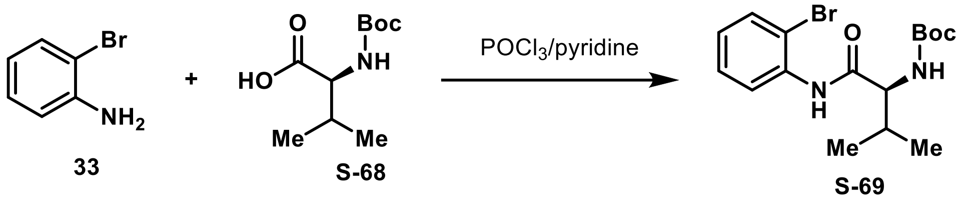

To a solution of 2-bromoaniline **33** (688 mg, 4.0 mmol, 1.0 equiv.) and Boc-*L*-valine **S-68** (869 mg, 4.0 mmol, 1.0 equiv.) in 12 mL pyridine was added $POCl_3$ (0.4 mL, 4.4 mmol, 1.1 equiv.) dropwise over 30 min at −10 °C. Then the reaction mixture was allowed to stir at −10 °C for another 30 min. Once the starting material was consumed completely, the mixture was quenched by saturated *aq.* $NaHCO_3$ (20 mL) and extracted with DCM (3 X 20 mL). The organic layers were combined, washed by saturated *aq.* NaCl, dried over Na_2SO_4 and removed under reduced pressure to give the residue; the residue was purified by silica gel chromatography (5% EtOAc/hexane) to give the compound **S-69** (1.22 g, 83% yield).

Compound S-69
Physical State: white solid
^{1}H NMR (400 MHz, $CDCl_3$): δ 8.53 – 8.20 (m, 2H), 7.54 (dd, J = 8.1, 1.5 Hz, 1H), 7.36 – 7.28 (m, 1H), 6.99 (td, J = 7.7, 1.6 Hz, 1H), 5.10 (s, 1H), 4.16 (s, 1H), 2.43 – 2.28 (m, 1H), 1.47 (s, 9H), 1.06 (d, J = 6.9 Hz, 3H), 0.99 (d, J = 6.9 Hz, 3H).
^{13}C NMR (151 MHz, $CDCl_3$): δ 170.11, 155.95, 135.39, 132.37, 128.46, 125.49, 121.94, 113.71, 80.51, 61.01, 30.53, 28.41, 19.50, 17.65.
HRMS (ESI-TOF): calculated for $C_{16}H_{23}BrN_2NaO_3^+$ $[M+Na]^+$: 393.0784, found: 393.0792.
TLC: R_f = 0.6 (10:1 Hexane:EtOAc, $Ce_2(SO_4)_3$ in phosphomolybdic acid).
$[\alpha]^{25}_D$: –22.4 (c = 1.0, $CHCl_3$)

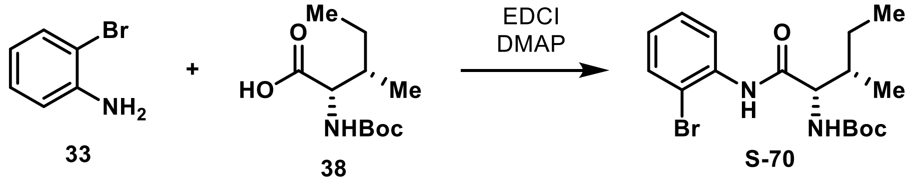

To a solution of compound **33** (3.44 g, 20.0 mmol, 1.0 equiv.), **38** (5.54 g, 24 mmol, 1.2 equiv.) and DAMP (976 mg, 8.0 mmol, 0.4 equiv.) in DCM (100 mL) was added EDCI (4.61 g, 24 mmol, 1.2 equiv.) in portion wise at 0 °C. The reaction mixture was allowed to stir at rt for 4 h and quenched by 0.5 M HCl (100 mL). The mixture was extracted with DCM (100 mL) three times, and the organic layers were combined, washed by saturated *aq.* NaCl, dried over Na_2SO_4 and removed under reduced pressure to give the residue. The residue was purified by silica gel chromatography (5% EtOAc/Hexane) to give the compound **S-70** (7.1 g, 80% yield).

Compound S-70
Physical State: white solid
^{1}H NMR (600 MHz, $CDCl_3$): δ 8.46 – 8.27 (m, 2H), 7.52 (dd, J = 8.1, 1.5 Hz, 1H), 7.30 (td, J = 7.7, 2.0 Hz, 1H), 6.97 (td, J = 7.7, 1.6 Hz, 1H), 5.15 – 4.80 (m, 1H), 4.24 (d, J = 71.5 Hz, 1H), 2.23 – 1.98 (m, 1H), 1.52 (ddd, J = 13.3, 7.4, 4.1 Hz, 1H), 1.45 (d, J = 3.9 Hz, 9H), 1.36 – 1.13 (m, 1H), 1.05 – 0.88 (m, 6H).

^{13}C NMR (151 MHz, $CDCl_3$): δ 170.10, 155.94, 135.40, 132.66, 132.36, 132.34, 128.54, 128.49, 128.46, 125.47, 125.44, 122.94, 121.78, 113.66, 80.56, 60.43, 59.11, 37.09, 36.82, 28.41, 26.58, 24.84, 15.95, 14.51, 11.82, 11.74.
HRMS (ESI-TOF): calculated for $C_{17}H_{25}BrN_2NaO_3^+$ $[M+Na]^+$: 407.0941, found: 407.0944.
TLC: R_f = 0.6 (15:1 Hexane:EtOAc, $Ce_2(SO_4)_3$ in phosphomolybdic acid).
$[\alpha]^{25}_D$: +5.5 (c = 1.0, $CHCl_3$)

Copies of NMR

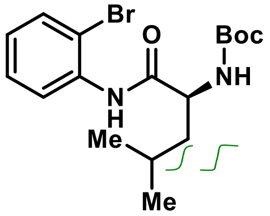

35

^{1}H NMR (600 MHz, CDCl$_3$)

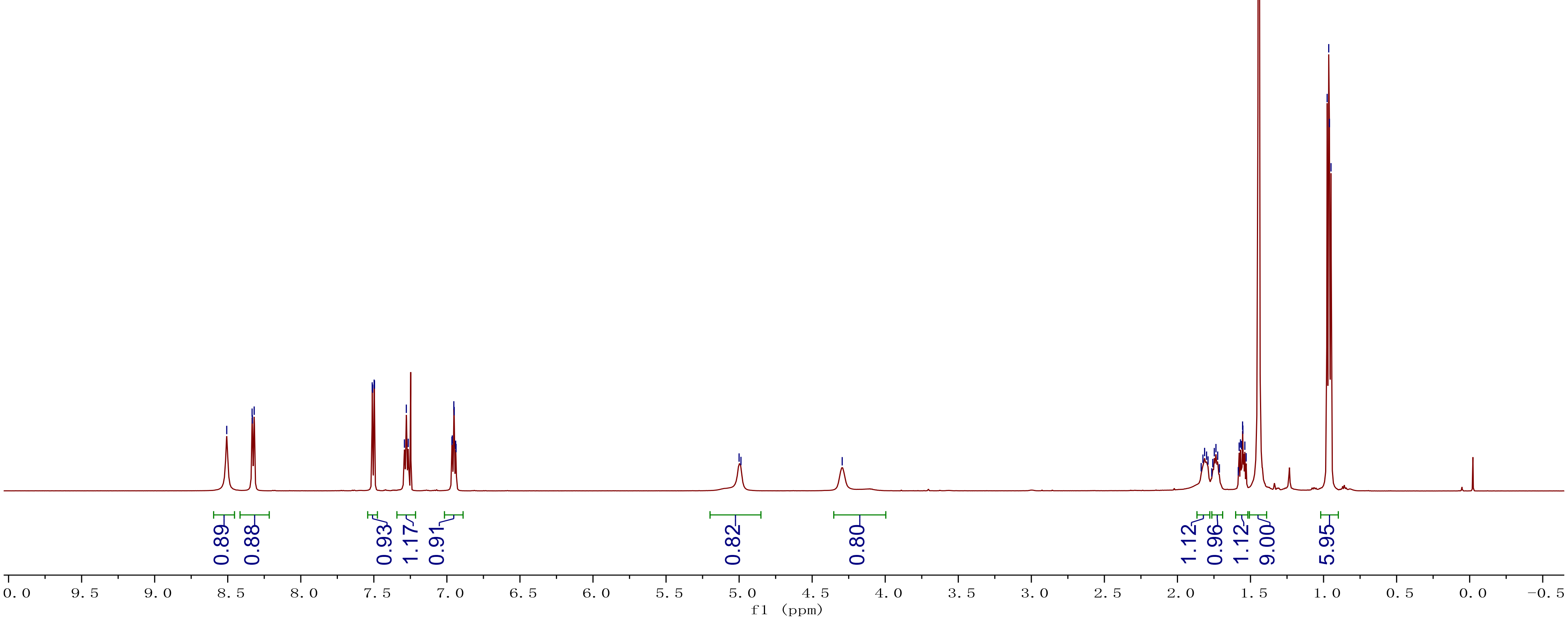

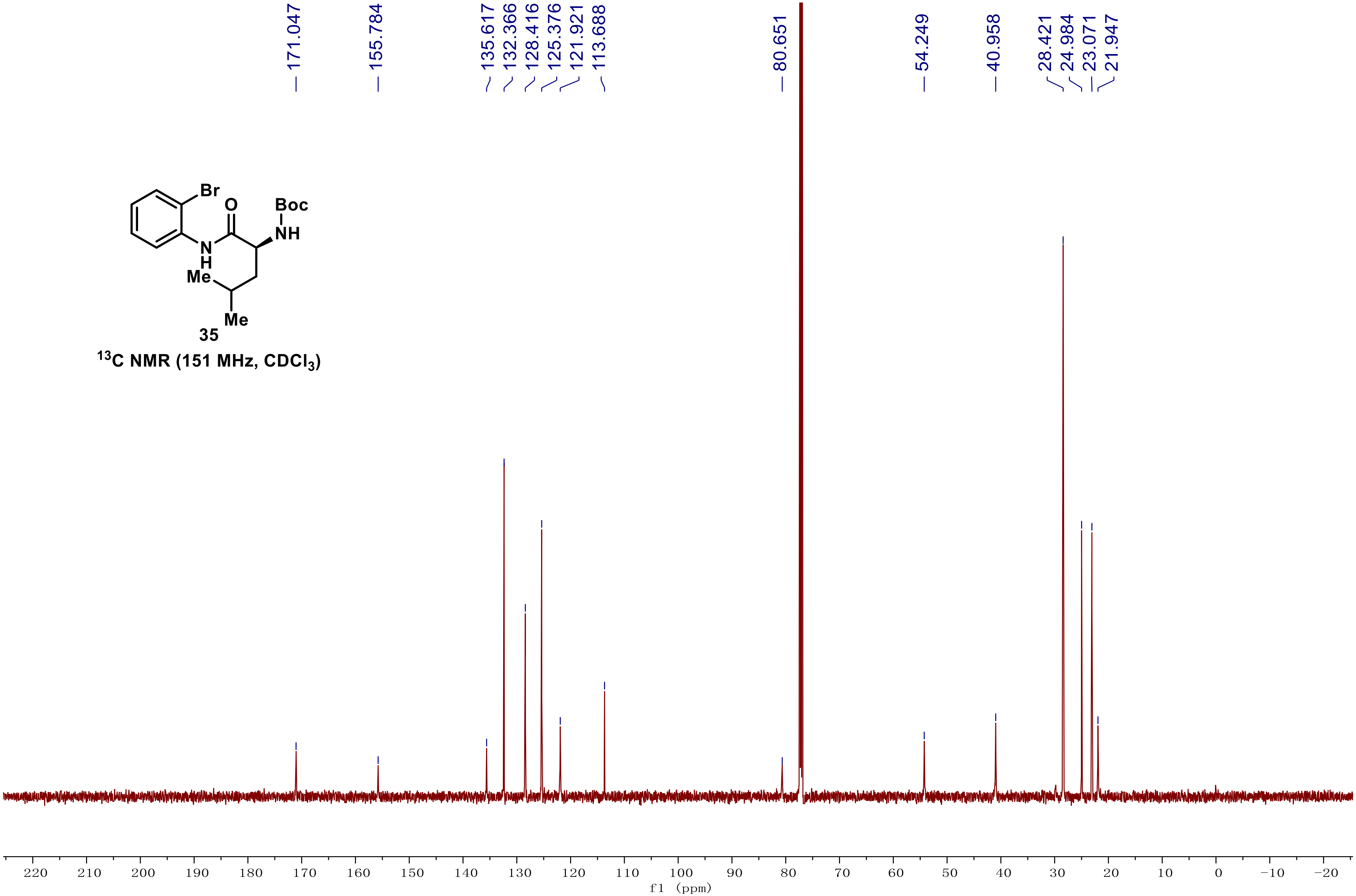

35

^{13}C NMR (151 MHz, $CDCl_3$)

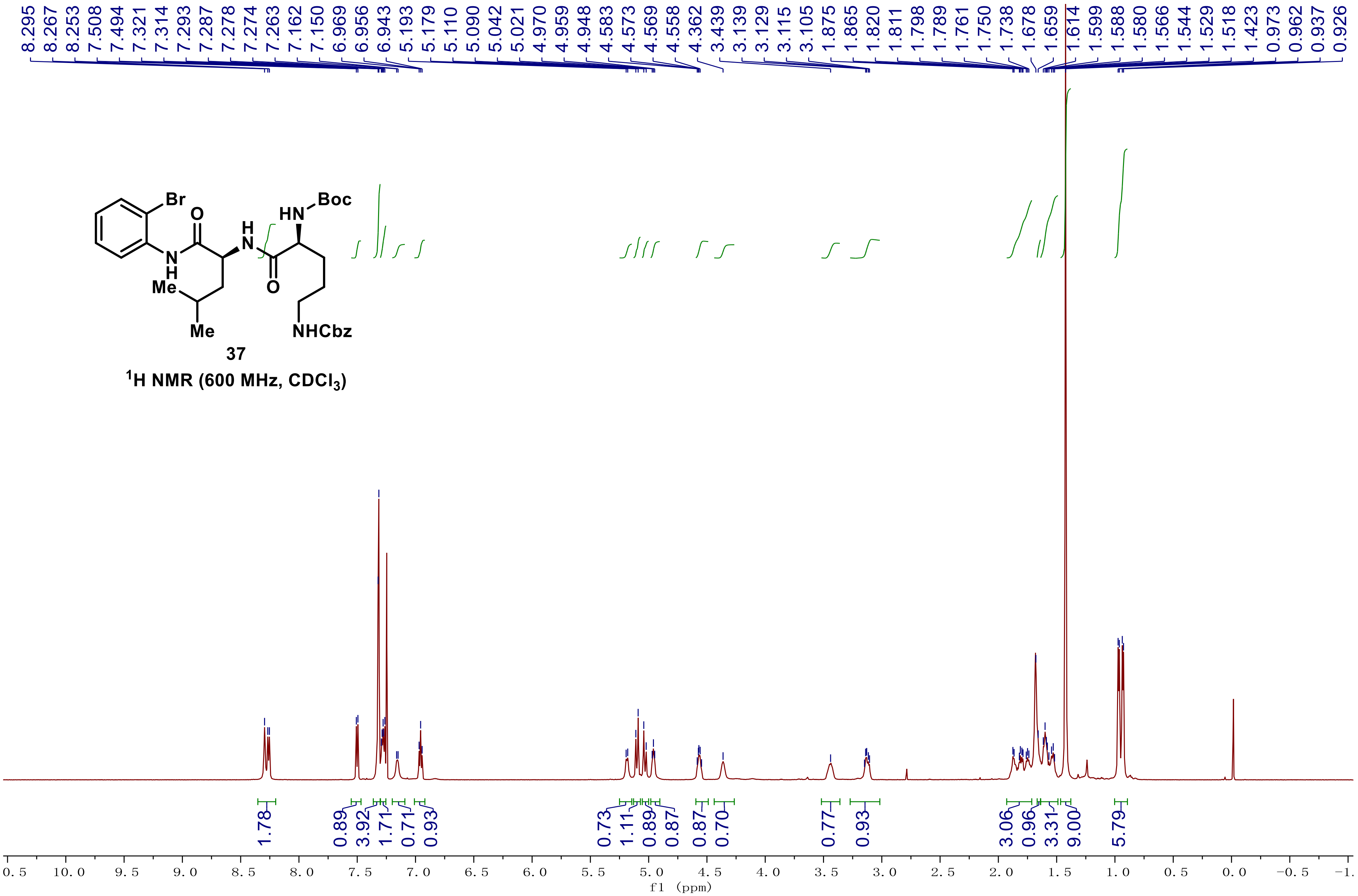

37

^{1}H NMR (600 MHz, $CDCl_3$)

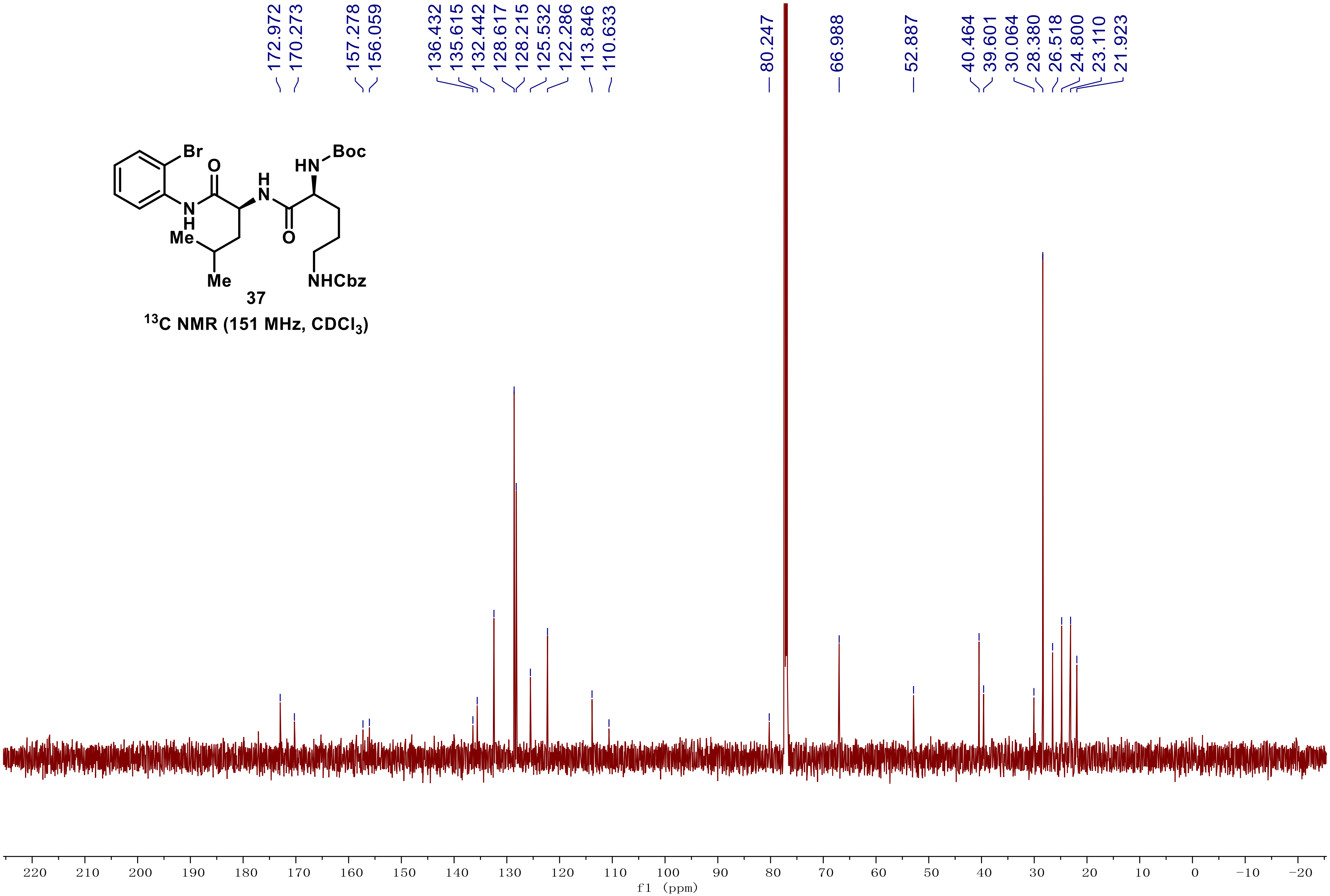
172.972
170.273
157.278
156.059
136.432
135.615
132.442
128.617
128.215
125.532
122.286
113.846
110.633
80.247
66.988
52.887
40.464
39.601
30.064
28.380
26.518
24.800
23.110
21.923
Br
O
HN
Boc
H
N
N
H
Me
O
Me
NHCbz
37
^{13}C NMR (151 MHz, $CDCl_3$)
220 210 200 190 180 170 160 150 140 130 120 110 100 90 80 70 60 50 40 30 20 10 0 −10 −20
f1 (ppm)

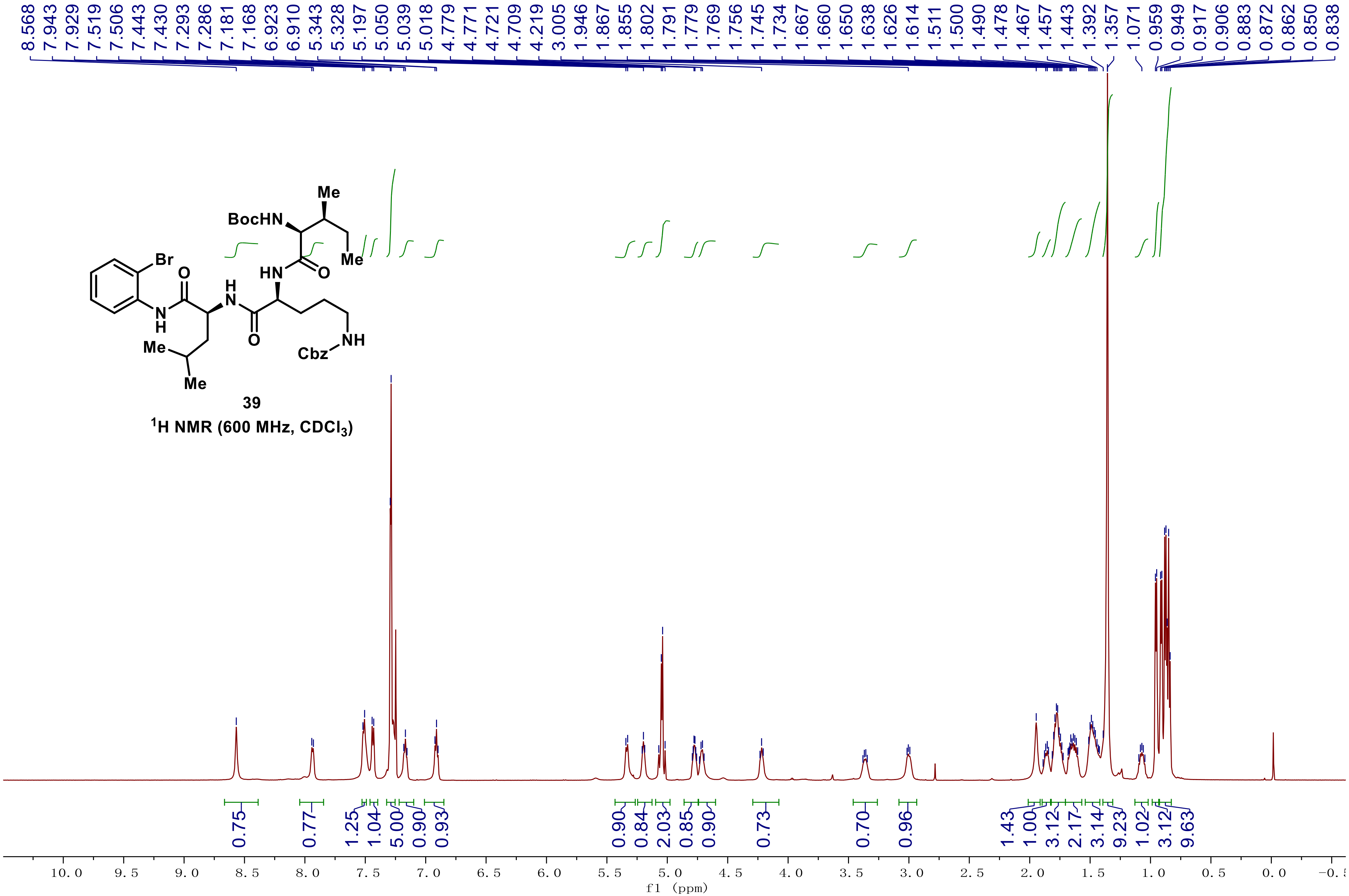

39

^{1}H NMR (600 MHz, $CDCl_3$)

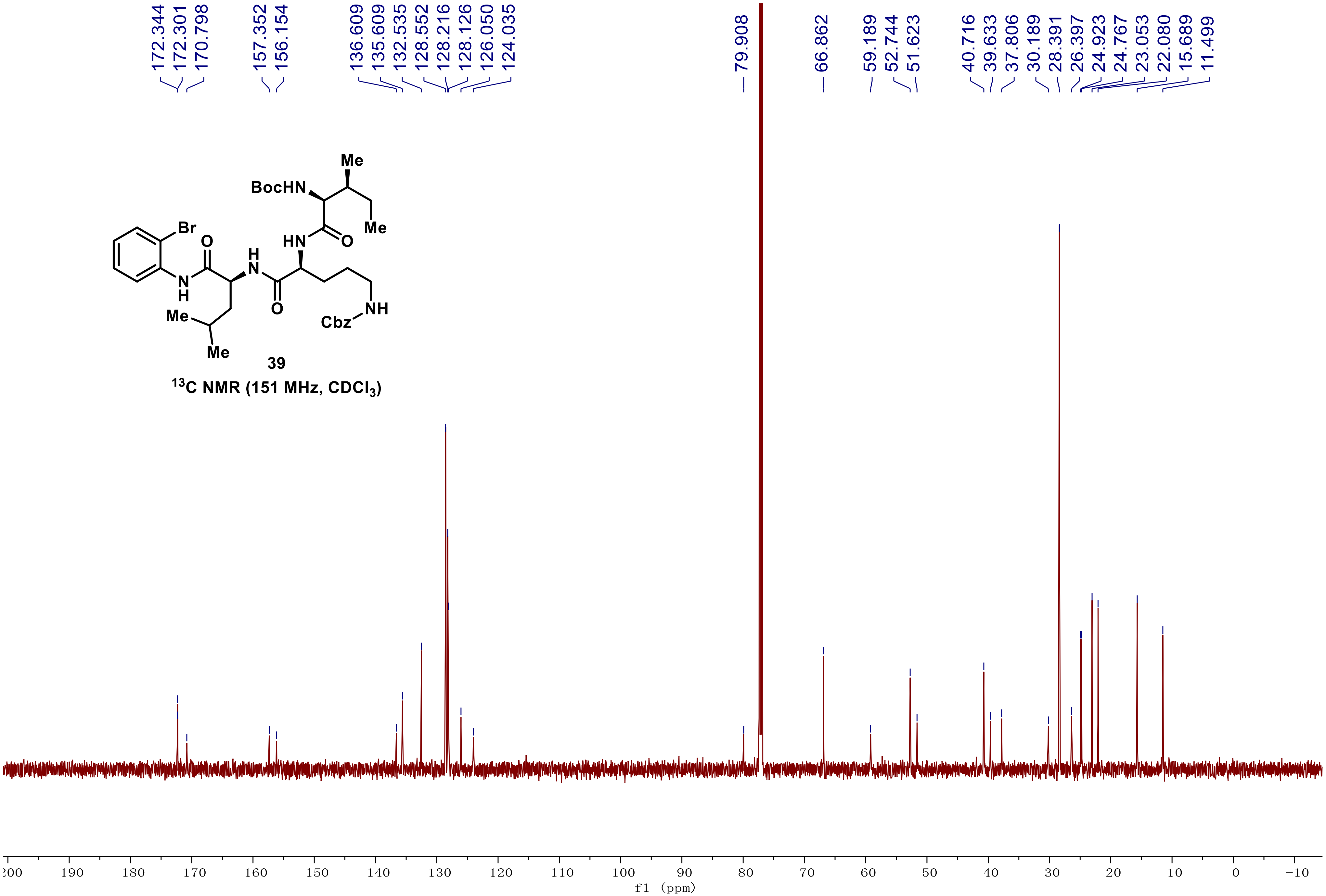

39

^{13}C NMR (151 MHz, $CDCl_3$)

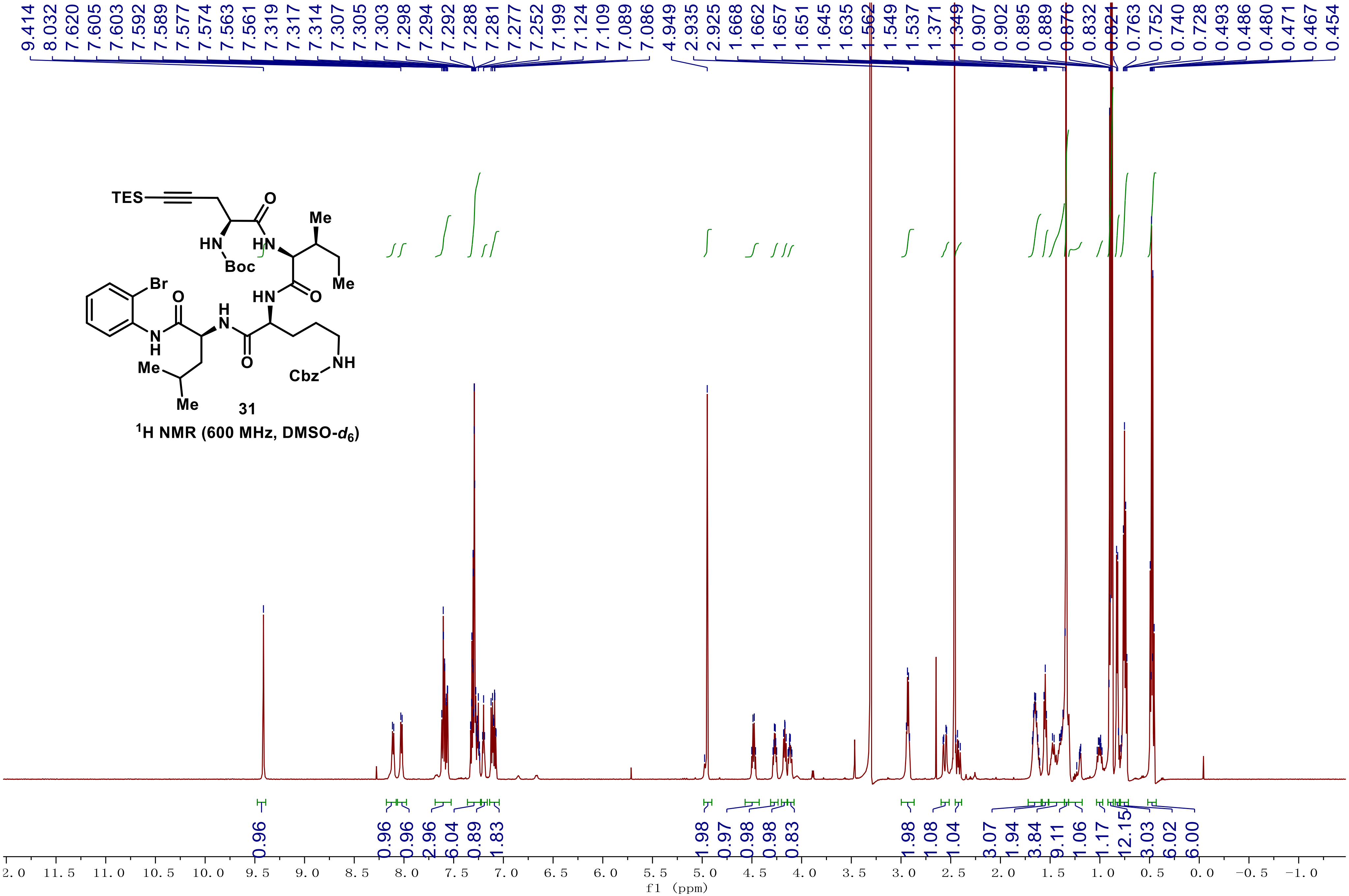

31

^{1}H NMR (600 MHz, DMSO-d_6)

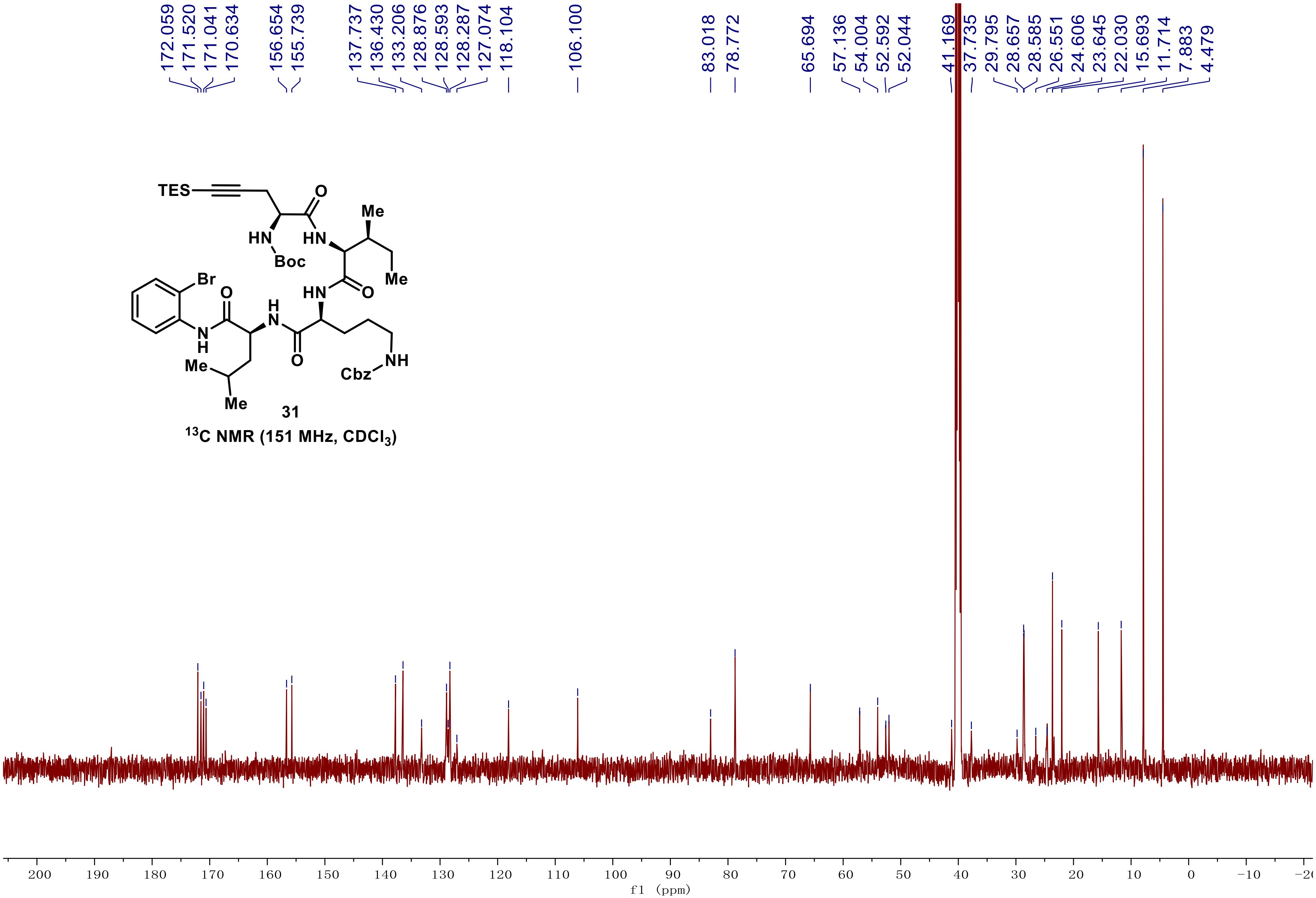
172.059
171.520
171.041
170.634
156.654
155.739
137.737
136.430
133.206
128.876
128.593
128.287
127.074
118.104
106.100
83.018
78.772
65.694
57.136
54.004
52.592
52.044
41.169
37.735
29.795
28.657
28.585
26.551
24.606
23.645
22.030
15.693
11.714
7.883
4.479
TES
O
Me
HN
Boc
HN
Me
Br
O
H
N
HN
O
N
H
O
Me
NH
Cbz
Me
31
^{13}C NMR (151 MHz, $CDCl_3$)
f1 (ppm)

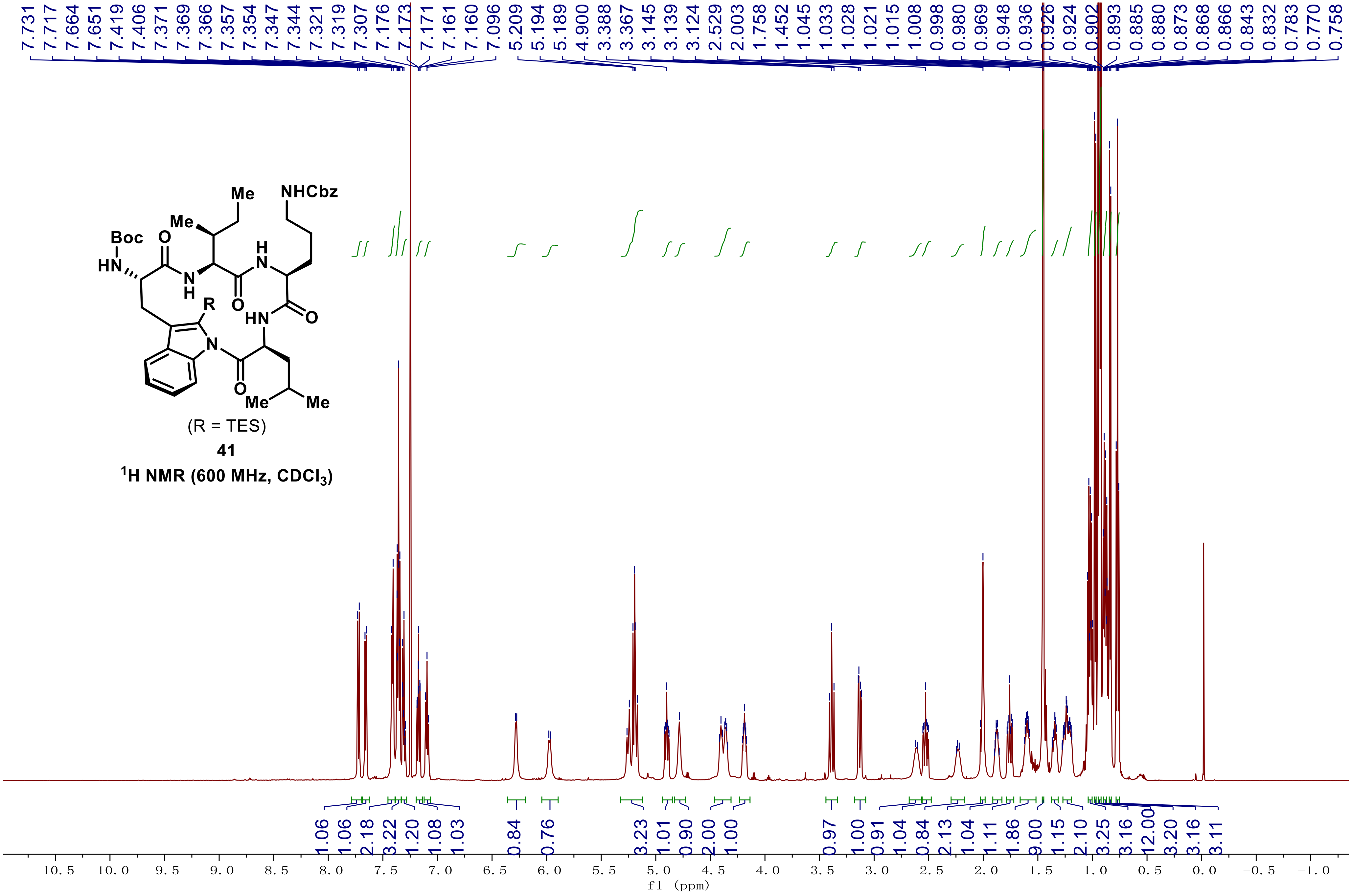

NHCbz
Me
Me
Boc
HN
O
N
H
H
N
R
O
HN
O
N
O
Me
Me
(R = TES)
41
¹H NMR (600 MHz, CDCl3)
f1 (ppm)

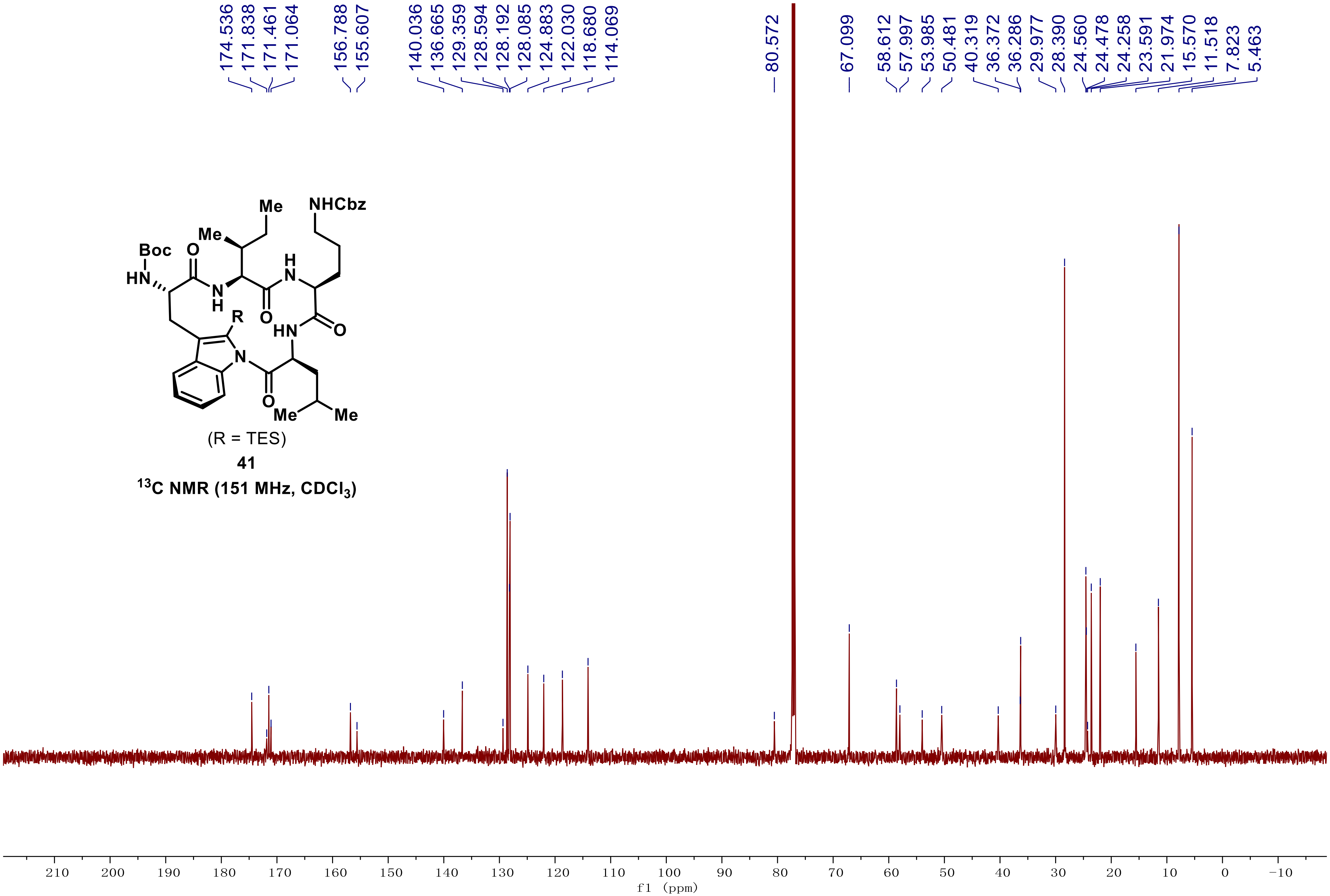

(R = TES)

41

^{13}C NMR (151 MHz, $CDCl_3$)

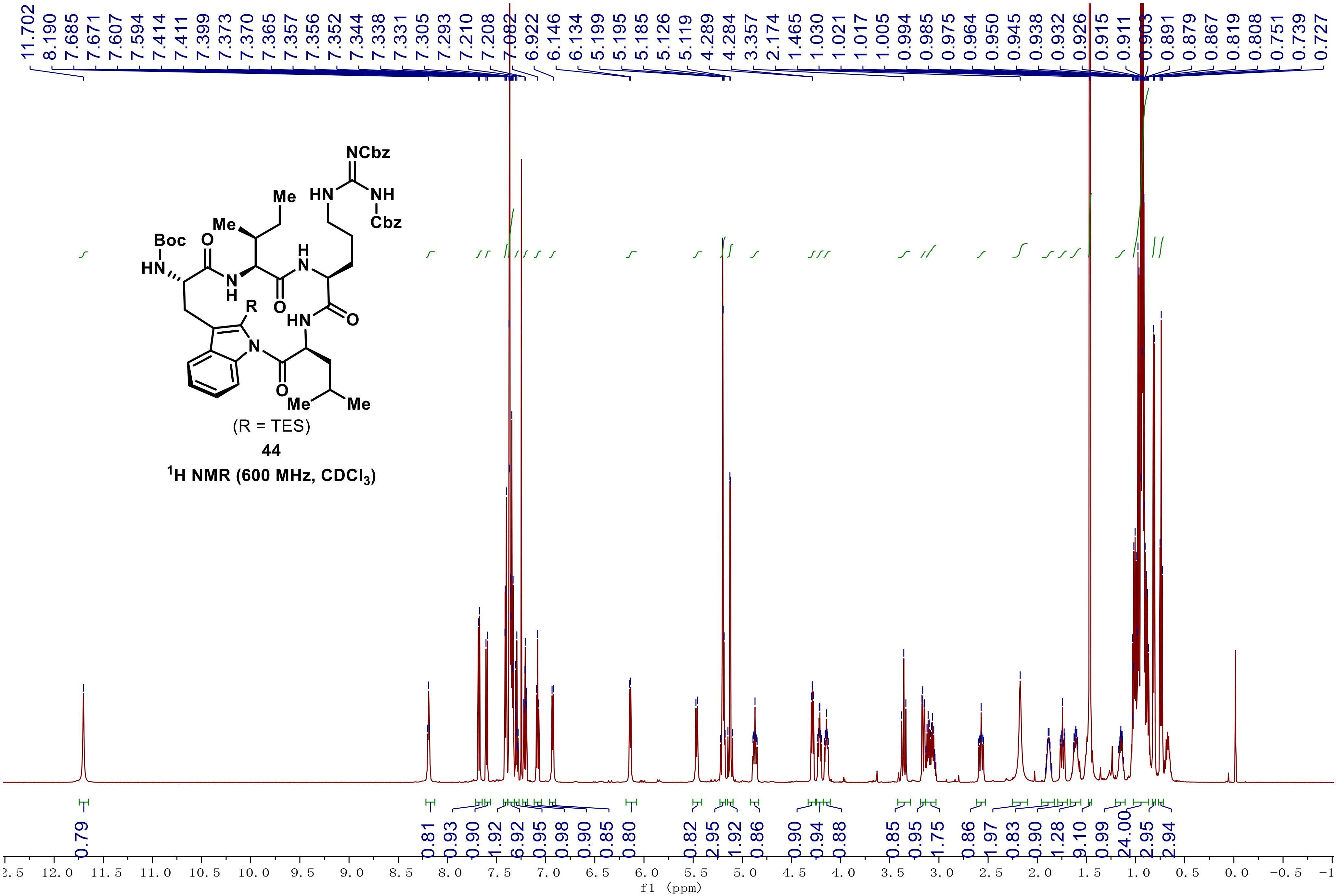

(R = TES)

44

^{1}H NMR (600 MHz, $CDCl_3$)

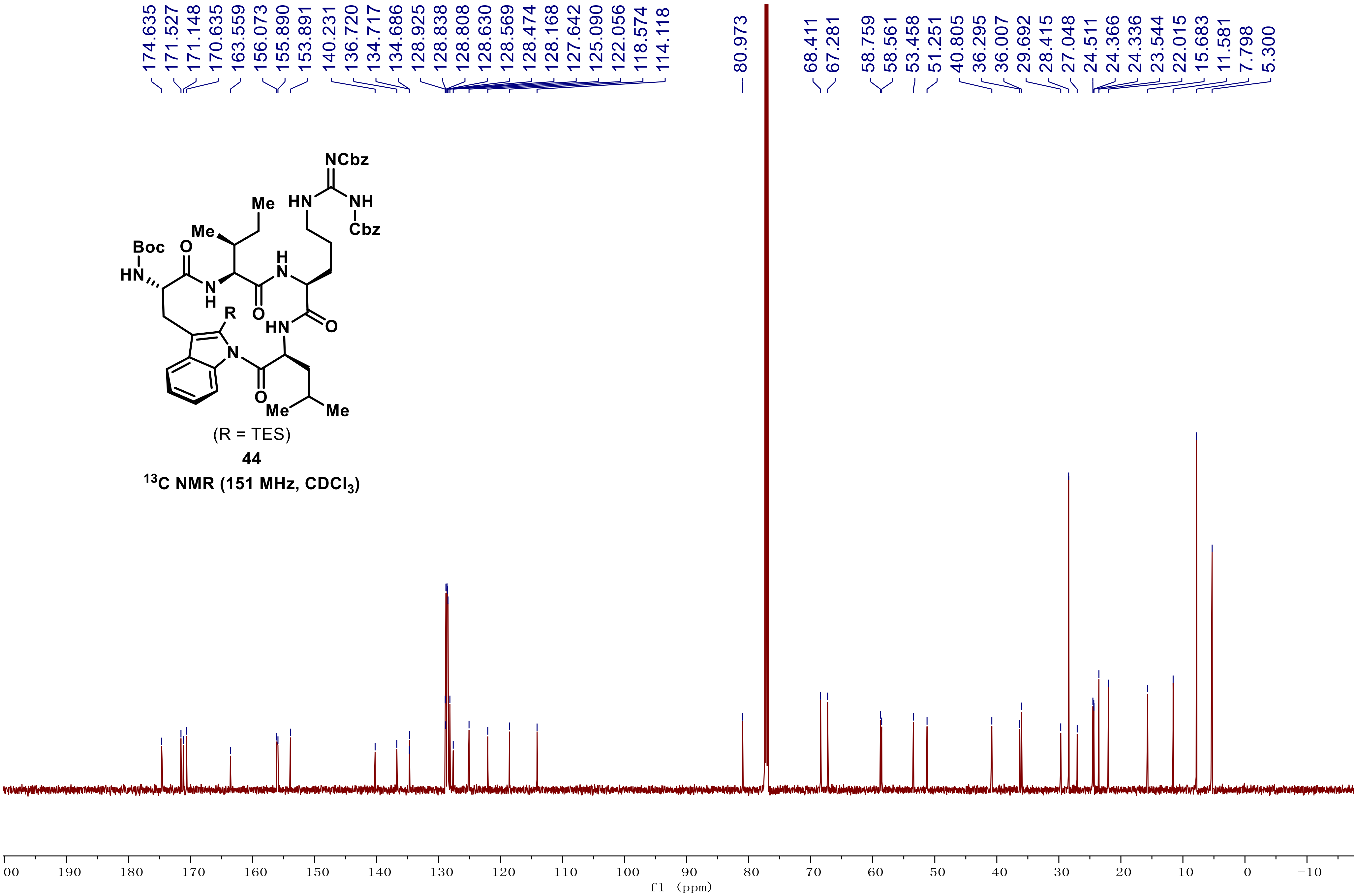

(R = TES)

44

^{13}C NMR (151 MHz, $CDCl_3$)

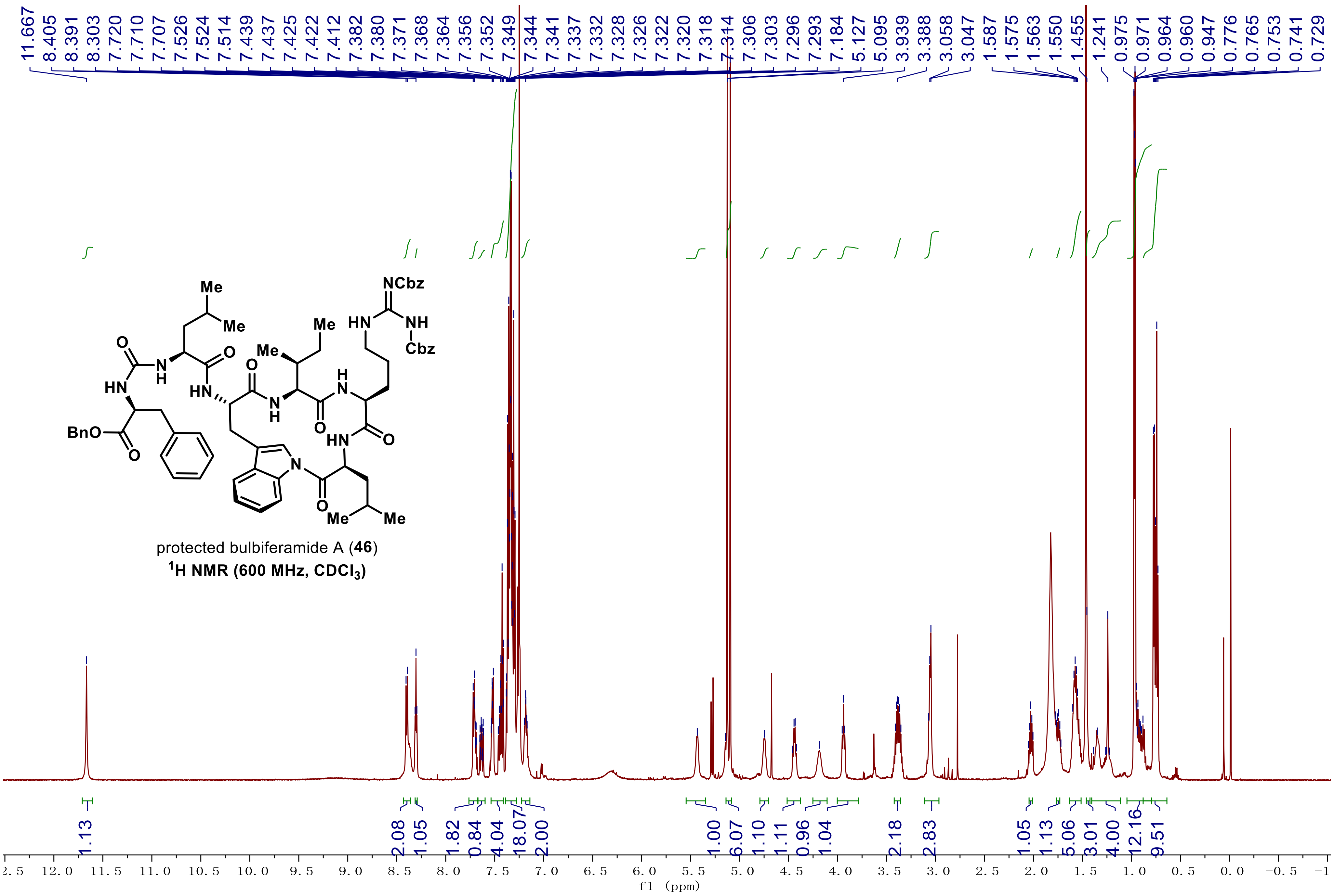

protected bulbiferamide A (**46**)

^{1}H NMR (600 MHz, CDCl$_3$)

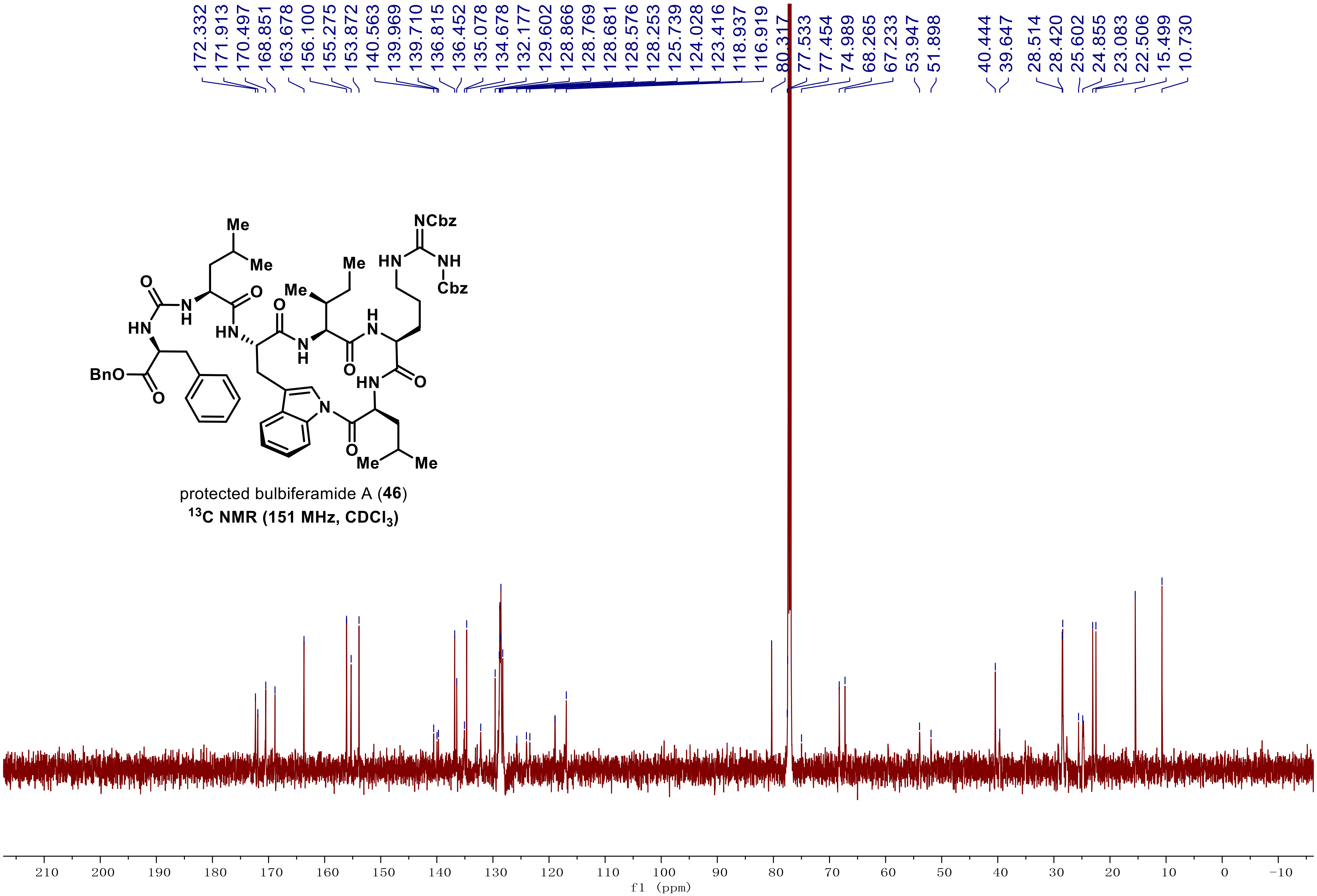

protected bulbiferamide A (**46**)
^{13}C NMR (151 MHz, CDCl$_3$)

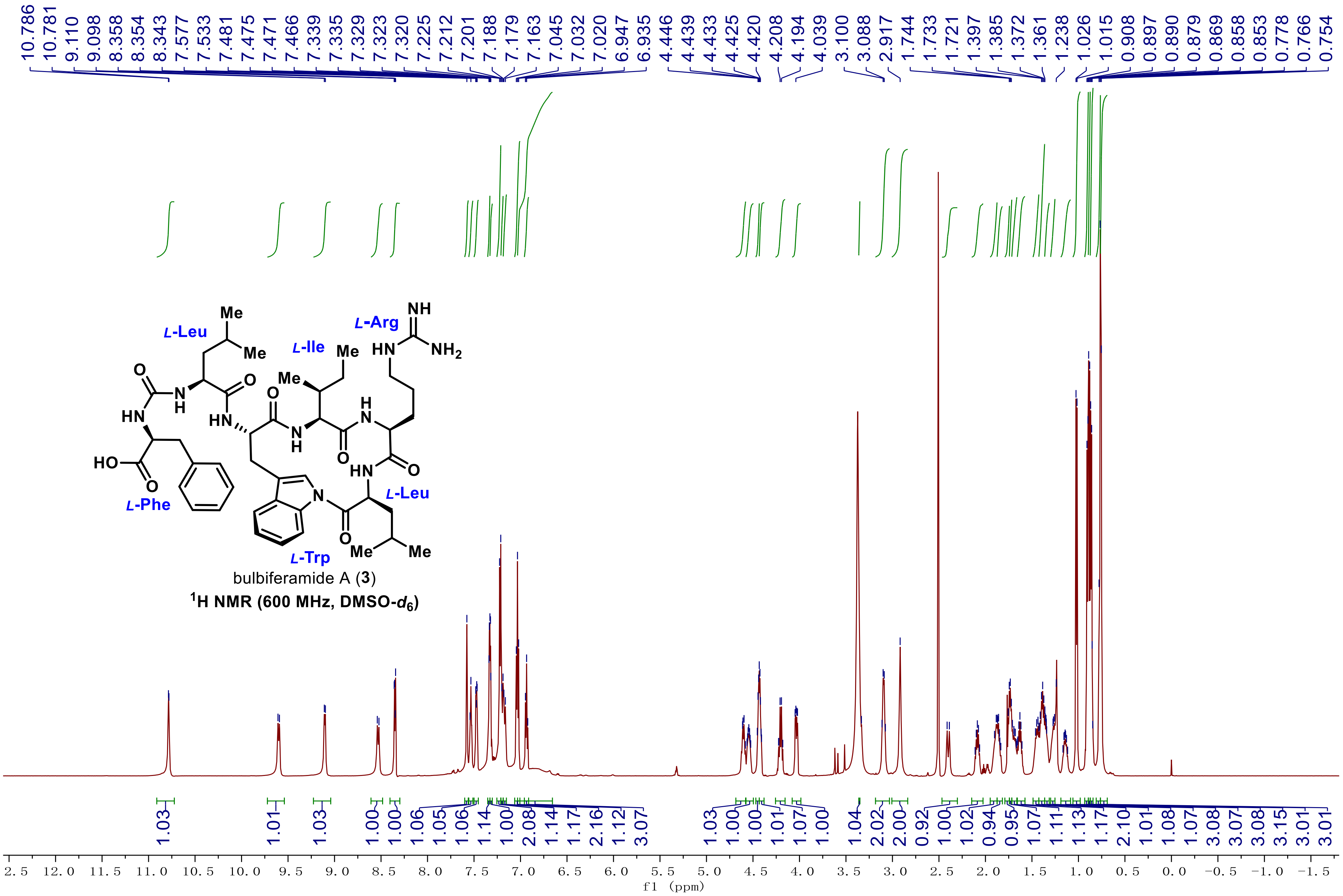

bulbiferamide A (**3**)

^{1}H NMR (600 MHz, DMSO-d_6)

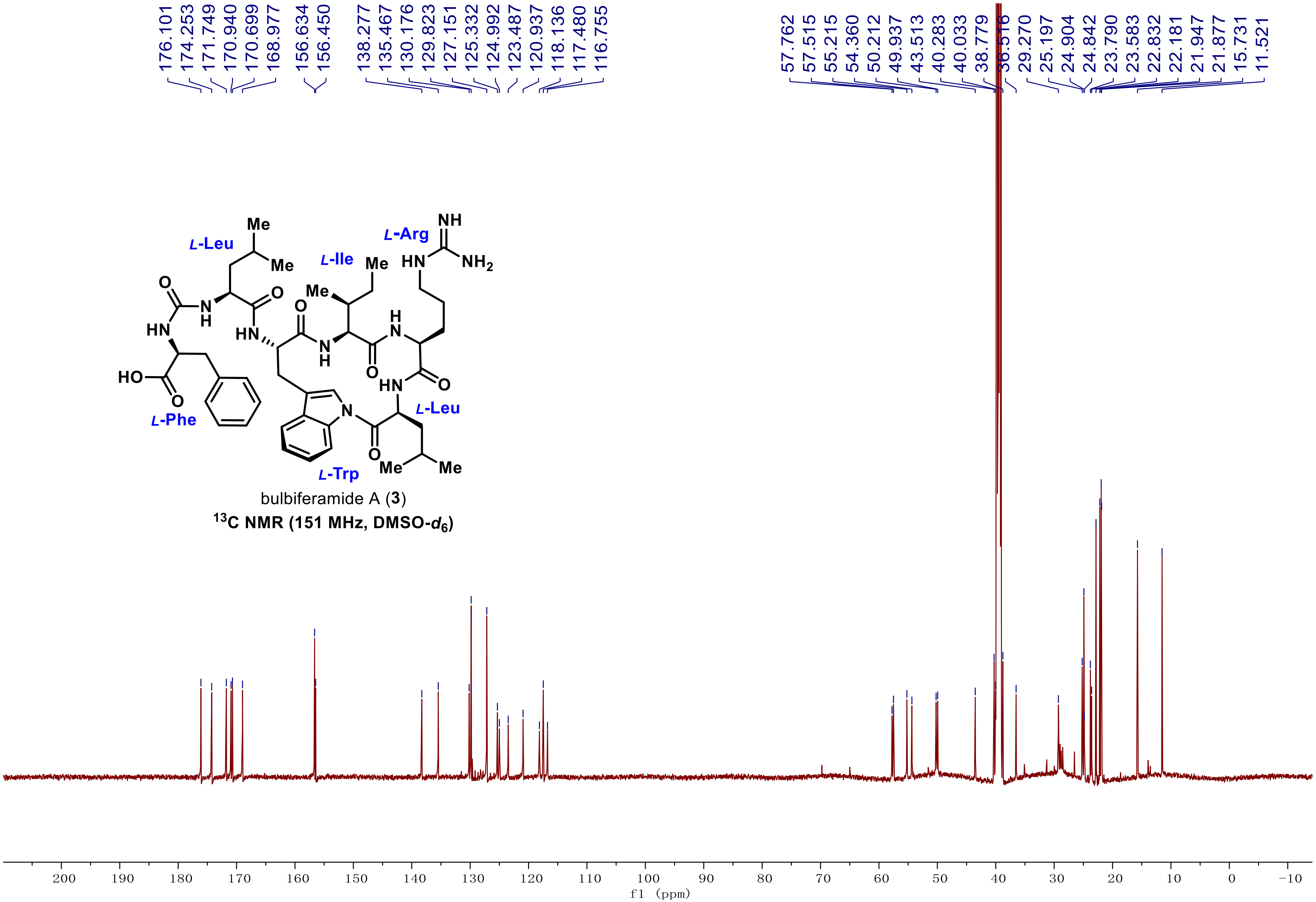

bulbiferamide A (**3**)

^{13}C NMR (151 MHz, DMSO-d_6)

^{1}HNMR Comparison of Bulbiferamide (3)

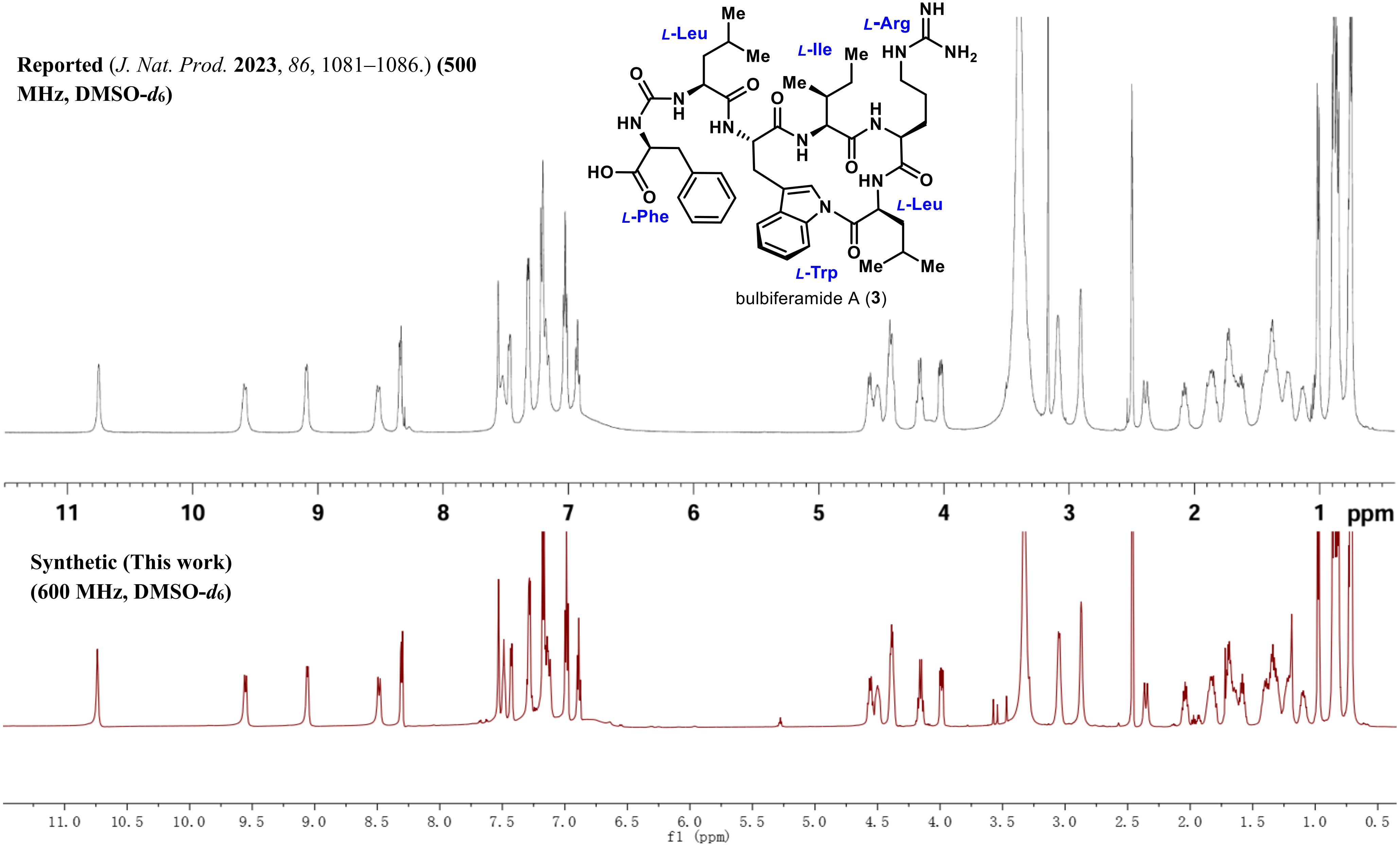

^{13}CNMR Comparison of Bulbiferamide (3)

Reported (*J. Nat. Prod.* **2023**, *86*, 1081–1086.) **(125 MHz, DMSO-*d*6)**

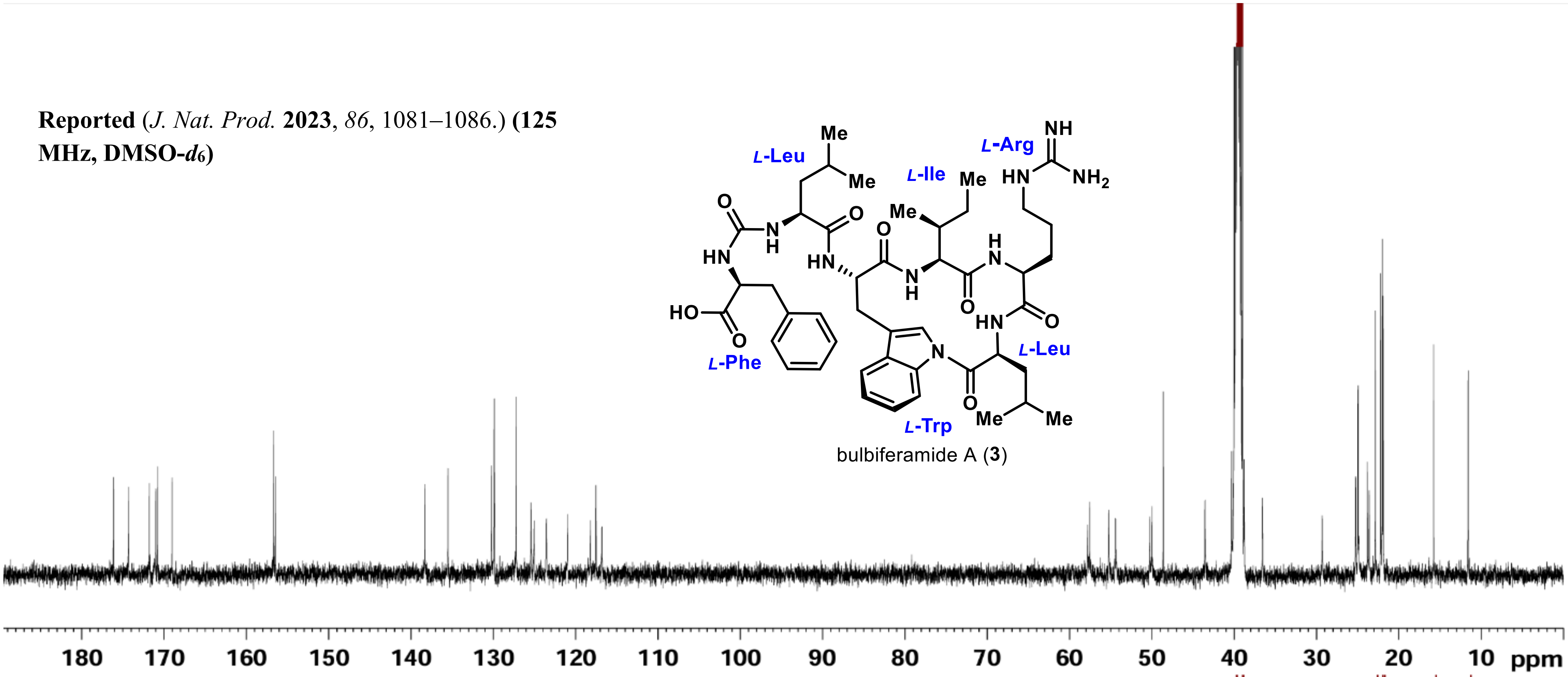

Synthetic (This work)
(151 MHz, DMSO-*d*6)

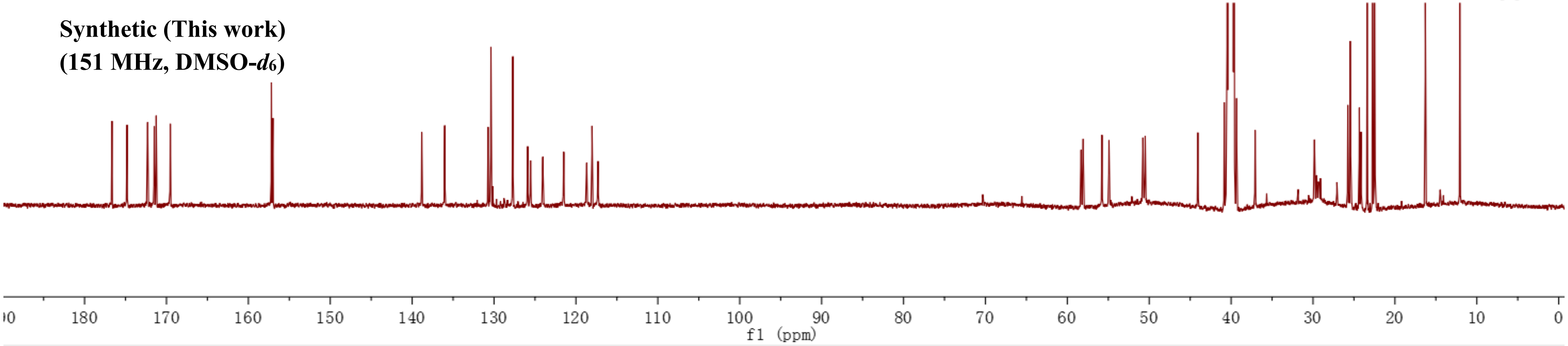

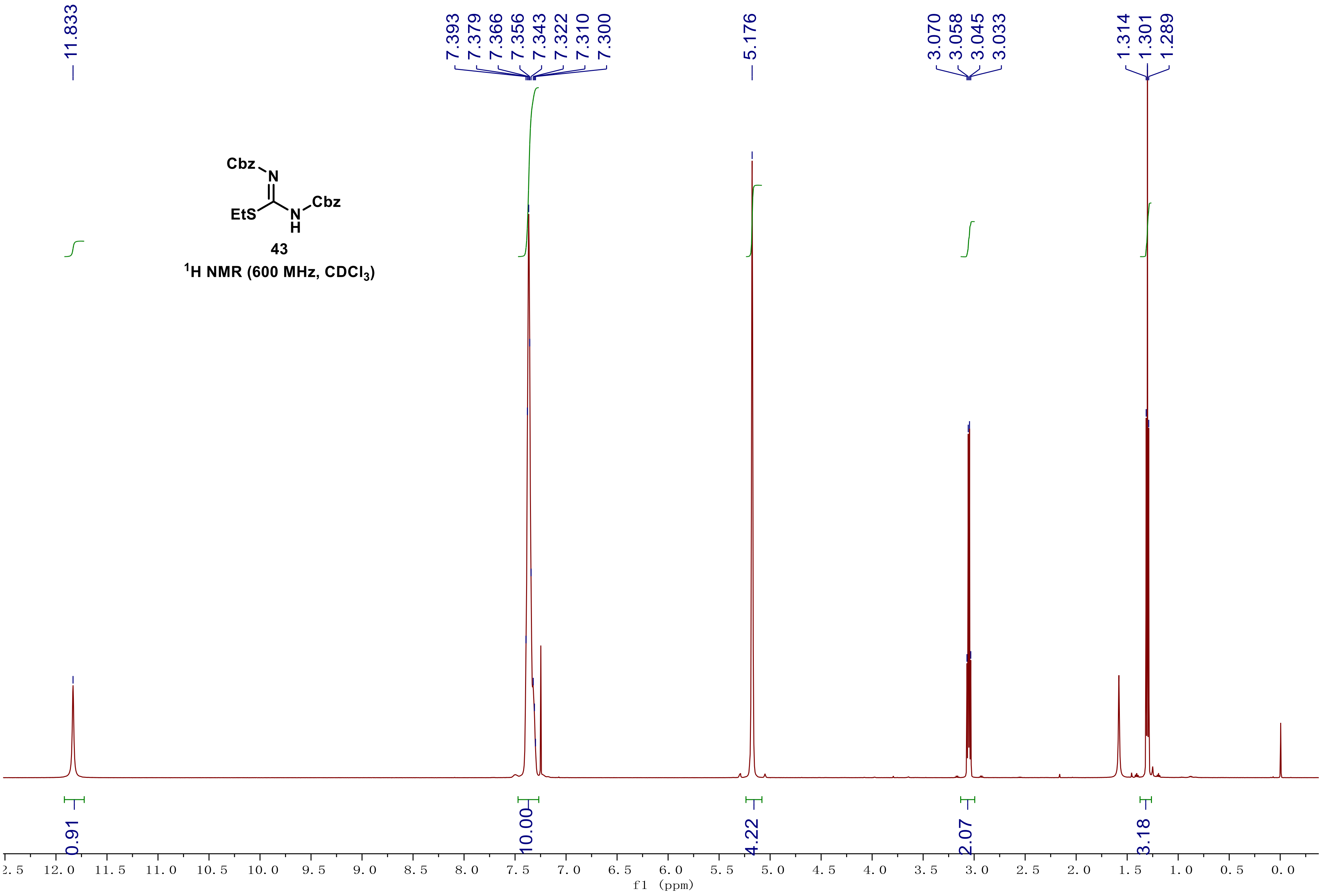
11.833
7.393
7.379
7.366
7.356
7.343
7.322
7.310
7.300
5.176
3.070
3.058
3.045
3.033
1.314
1.301
1.289
Cbz
N
EtS
N
H
Cbz
43
1H NMR (600 MHz, CDCl3)
0.91
10.00
4.22
2.07
3.18
12.0
11.5
11.0
10.5
10.0
9.5
9.0
8.5
8.0
7.5
7.0
6.5
6.0
5.5
5.0
4.5
4.0
3.5
3.0
2.5
2.0
1.5
1.0
0.5
0.0
f1 (ppm)

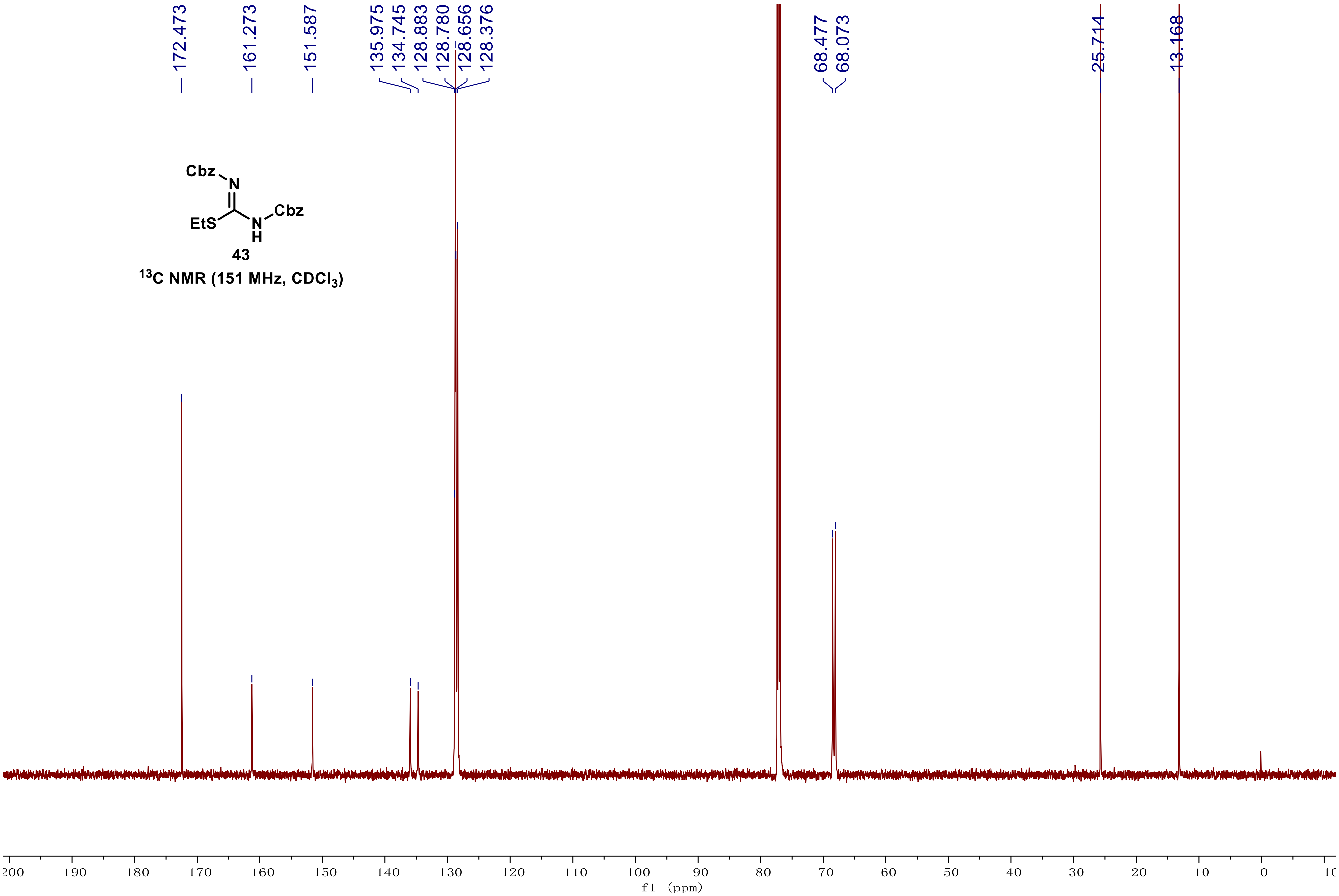

172.473
161.273
151.587
135.975
134.745
128.883
128.780
128.656
128.376
68.477
68.073
25.714
13.168
Cbz
N
EtS
N
H
Cbz
43
13C NMR (151 MHz, CDCl3)
200
190
180
170
160
150
140
130
120
110
100
90
80
70
60
50
40
30
20
10
0
f1 (ppm)

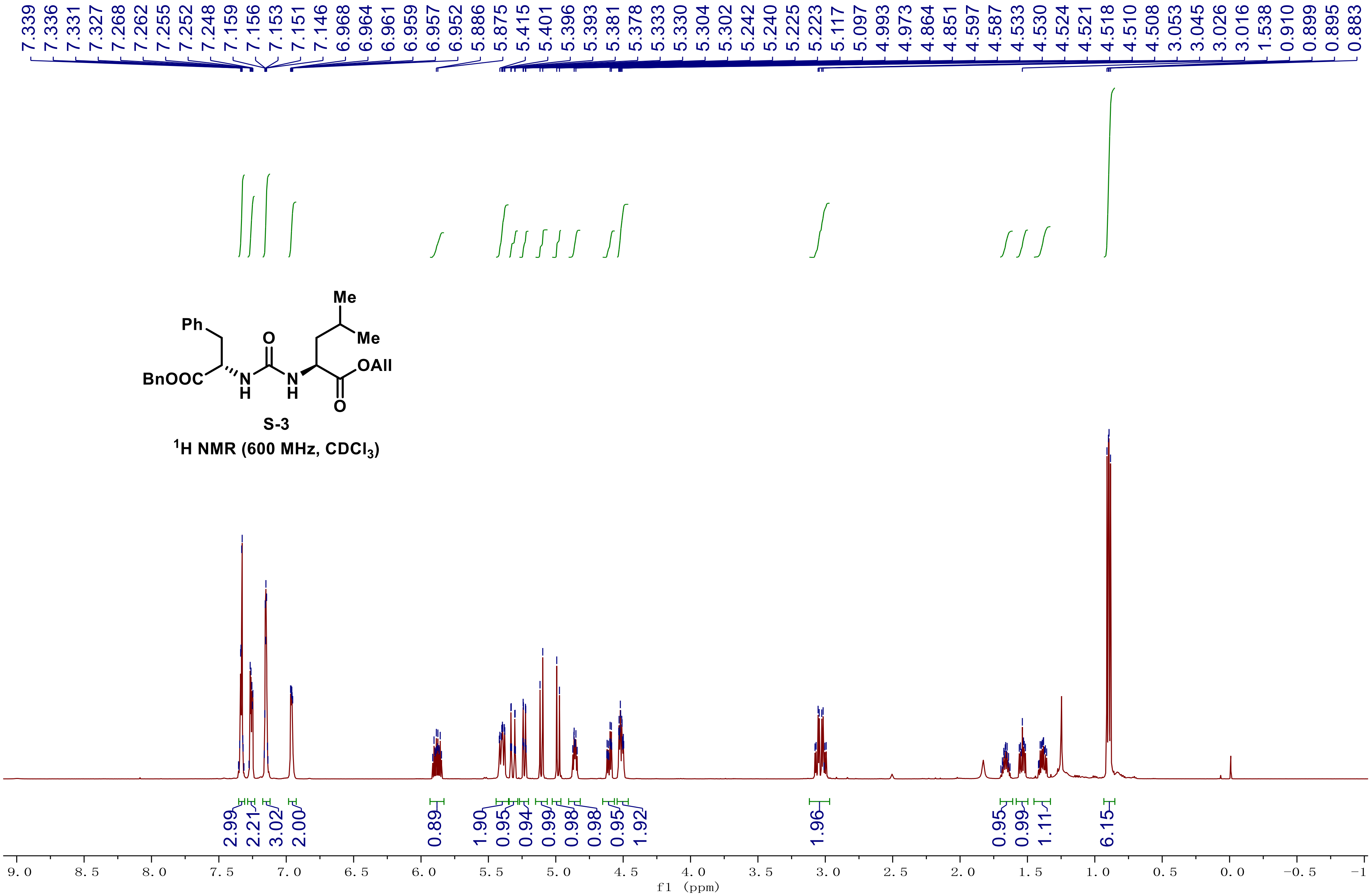

7.339 7.336 7.331 7.327 7.268 7.262 7.255 7.252 7.248 7.159 7.156 7.153 7.151 7.146 6.968 6.964 6.961 6.959 6.957 6.952 5.886 5.875 5.415 5.401 5.396 5.393 5.381 5.378 5.333 5.330 5.304 5.302 5.242 5.240 5.225 5.223 5.117 5.097 4.993 4.973 4.864 4.851 4.597 4.587 4.533 4.530 4.524 4.521 4.518 4.510 4.508 3.053 3.045 3.026 3.016 1.538 0.910 0.899 0.895 0.883
Me
Ph
O
Me
OAll
BnOOC
N
H
N
H
O
S-3
^{1}H NMR (600 MHz, CDCl$_3$)
2.99 2.21 3.02 2.00 0.89 1.90 0.95 0.94 0.99 0.98 0.98 0.95 1.92 1.96 0.95 0.99 1.11 6.15
9.0 8.5 8.0 7.5 7.0 6.5 6.0 5.5 5.0 4.5 4.0 3.5 3.0 2.5 2.0 1.5 1.0 0.5 0.0 -0.5 -1
f1 (ppm)

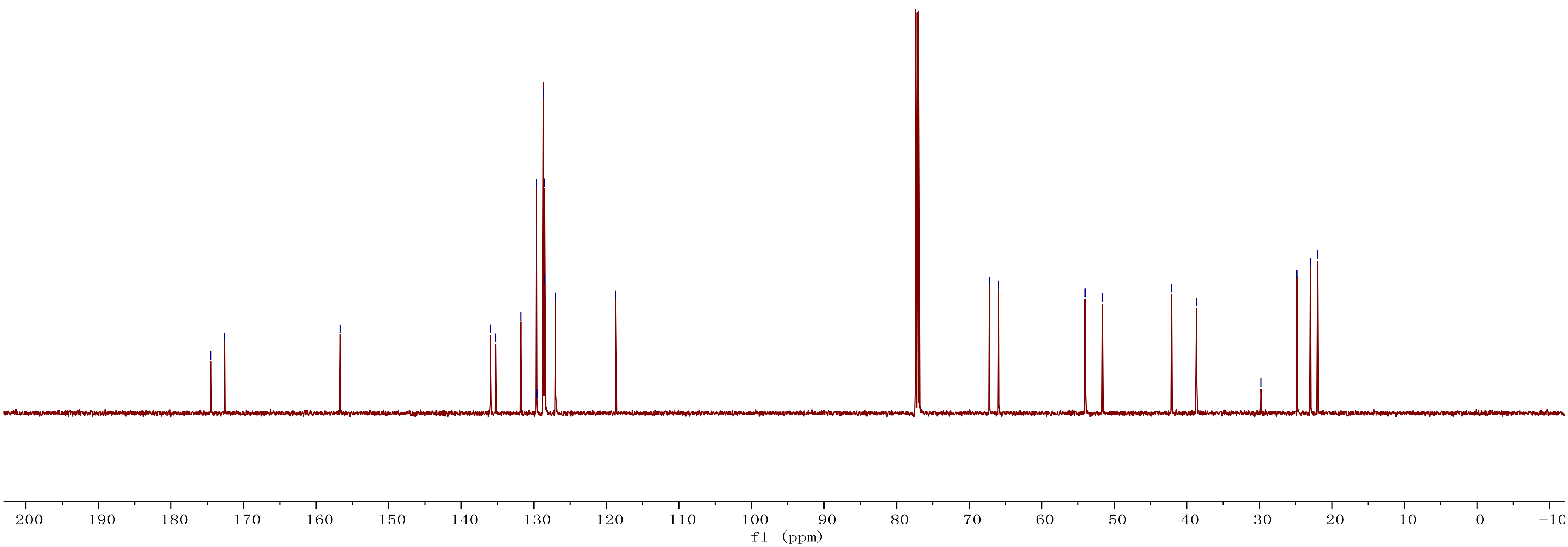

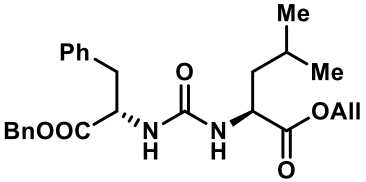

S-3

^{13}C NMR (151 MHz, $CDCl_3$)

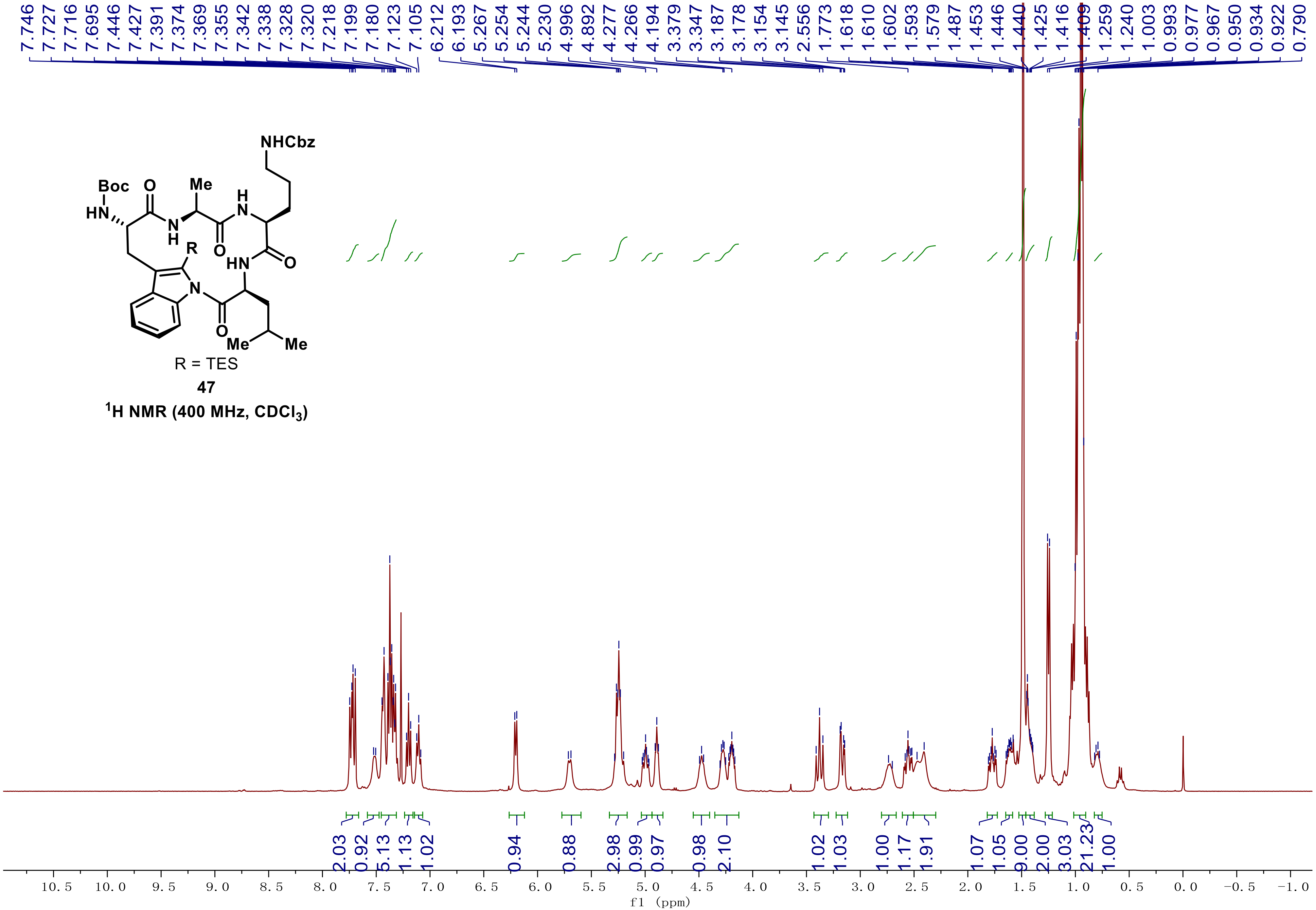
7.746 7.727 7.716 7.695 7.446 7.427 7.391 7.374 7.369 7.355 7.342 7.338 7.328 7.320 7.218 7.199 7.180 7.123 7.105 6.212 6.193 5.267 5.254 5.244 5.230 4.996 4.892 4.277 4.266 4.194 3.379 3.347 3.187 3.178 3.154 3.145 2.556 1.773 1.618 1.610 1.602 1.593 1.579 1.487 1.453 1.446 1.440 1.425 1.416 1.409 1.259 1.240 1.003 0.993 0.977 0.967 0.950 0.934 0.922 0.790
NHCbz
Boc
O
Me
H
HN
N
N
H
R
O
HN
O
N
O
Me
Me
R = TES
47
^{1}H NMR (400 MHz, CDCl$_3$)
2.03 0.92 5.13 1.13 1.02 0.94 0.88 2.98 0.99 0.97 0.98 2.10 1.02 1.03 1.00 1.17 1.91 1.07 1.05 9.00 2.00 3.03 21.23 1.00
10.5 10.0 9.5 9.0 8.5 8.0 7.5 7.0 6.5 6.0 5.5 5.0 4.5 4.0 3.5 3.0 2.5 2.0 1.5 1.0 0.5 0.0 -0.5 -1.0
f1 (ppm)

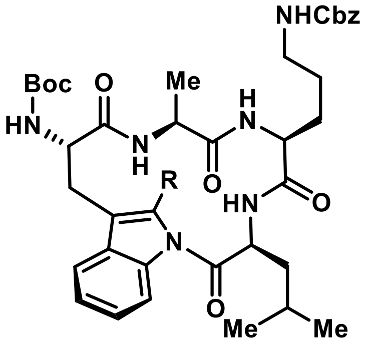

R = TES

47

^{13}C NMR (101 MHz, CDCl$_3$)

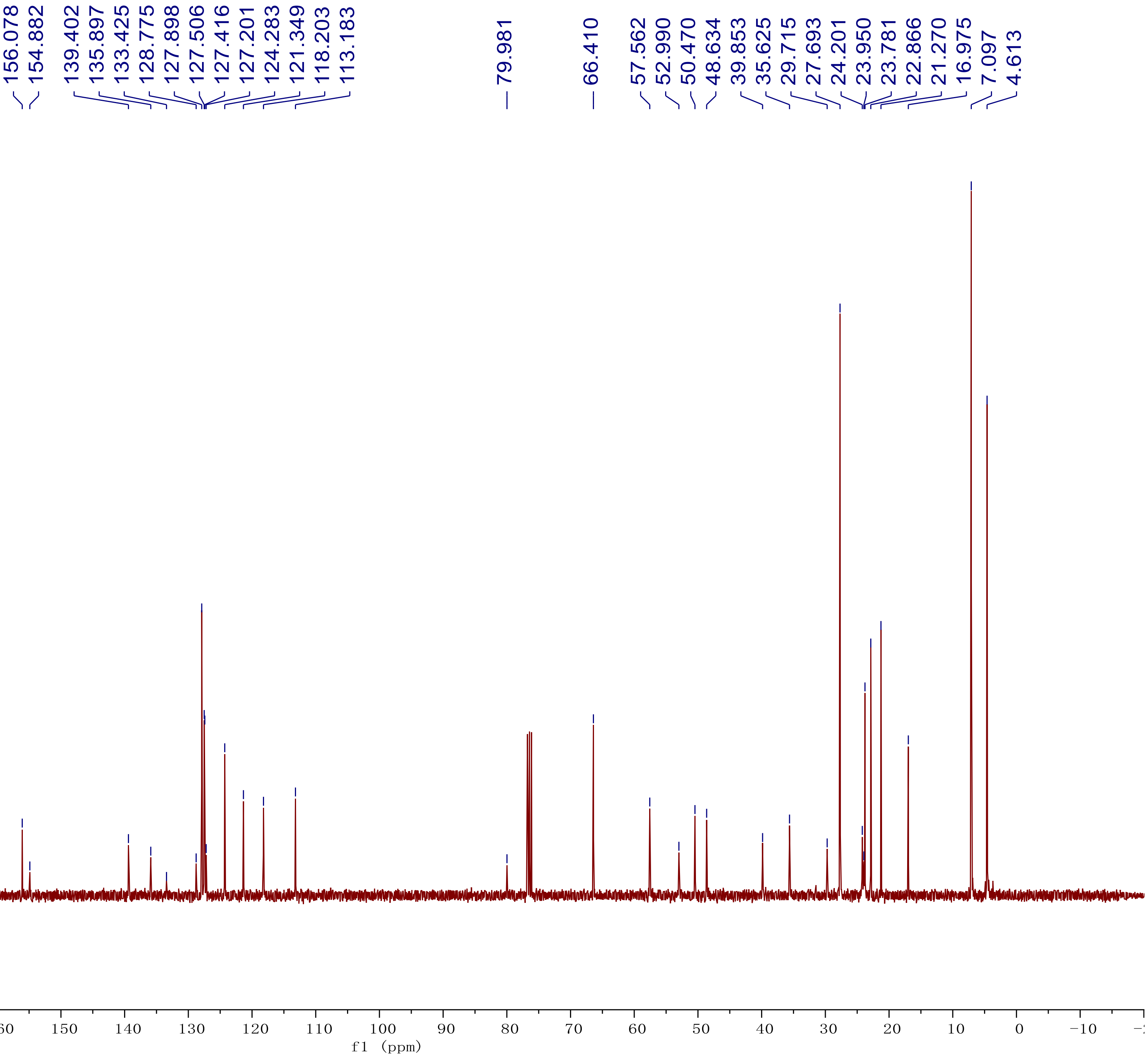

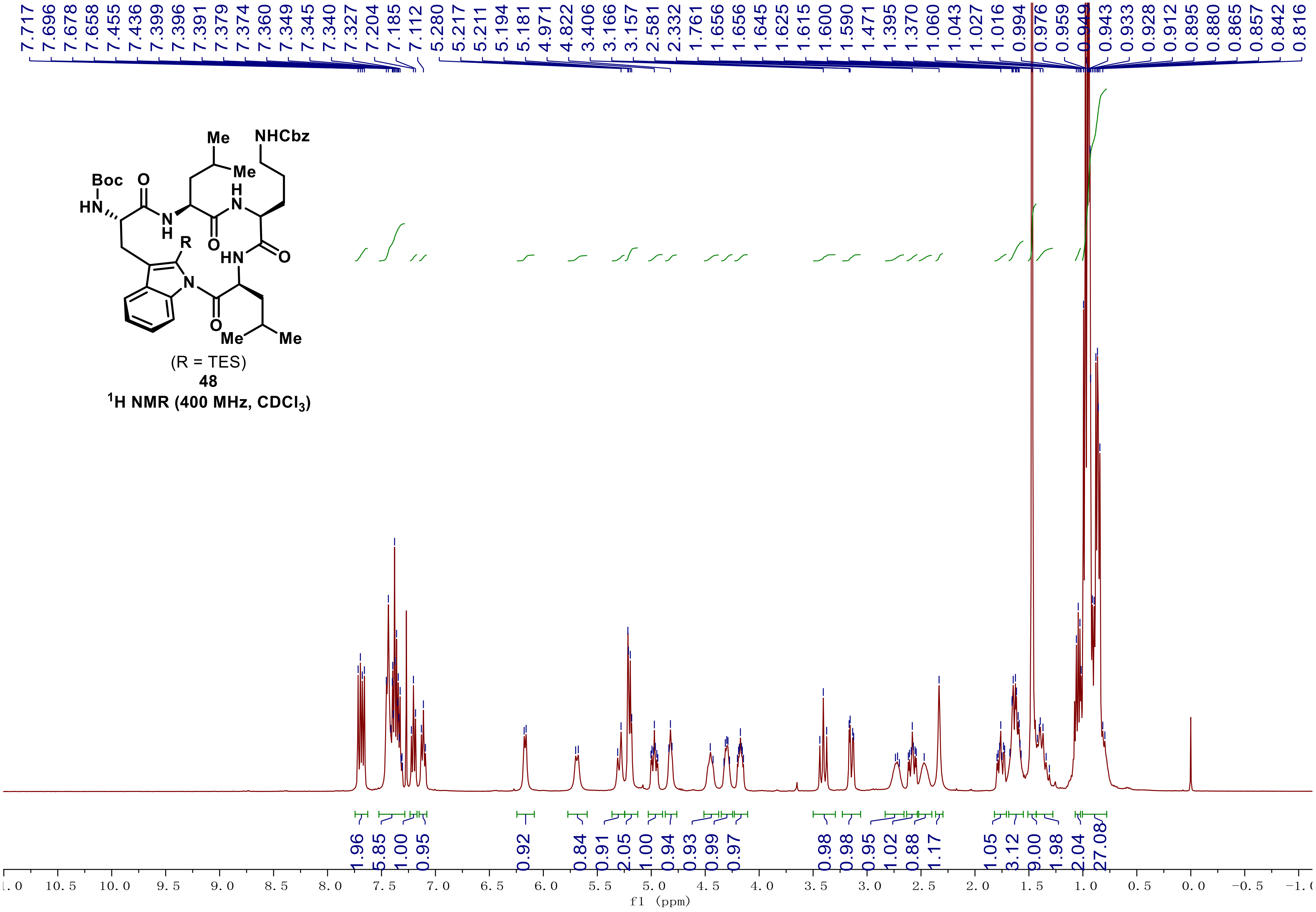

(R = TES)
48
^{1}H NMR (400 MHz, $CDCl_3$)

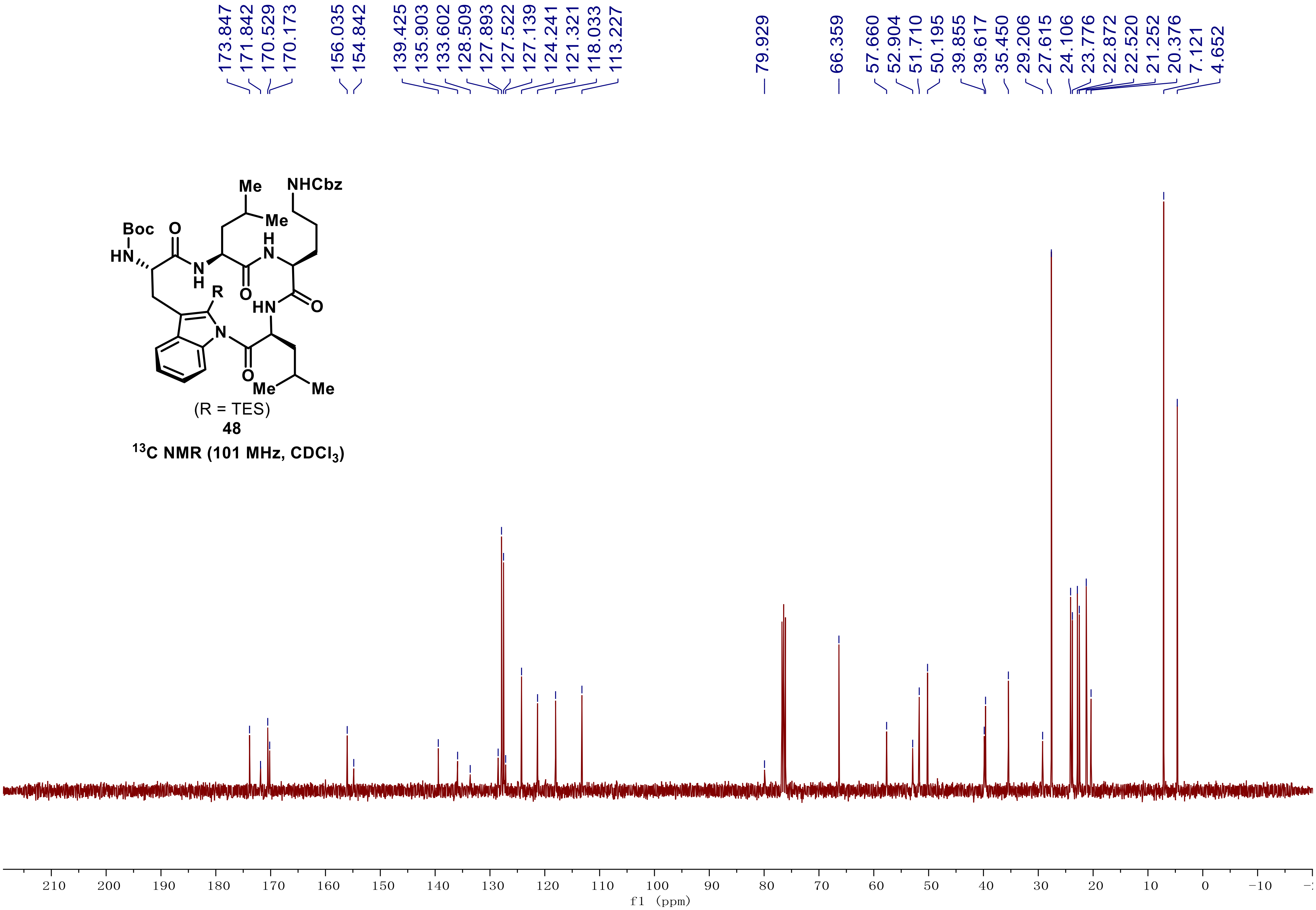

(R = TES)
48
^{13}C NMR (101 MHz, CDCl$_3$)

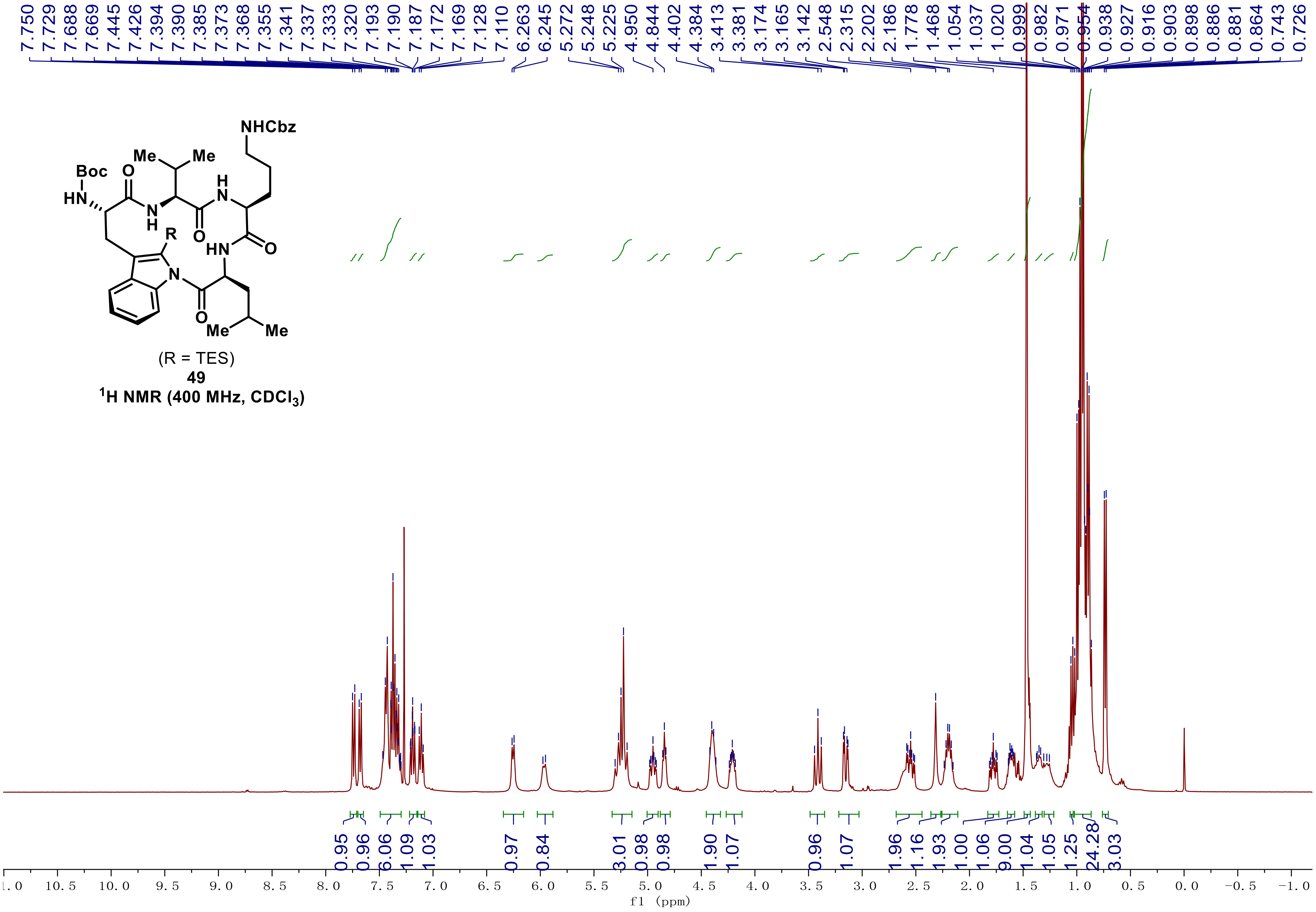

(R = TES)
49
^{1}H NMR (400 MHz, $CDCl_3$)

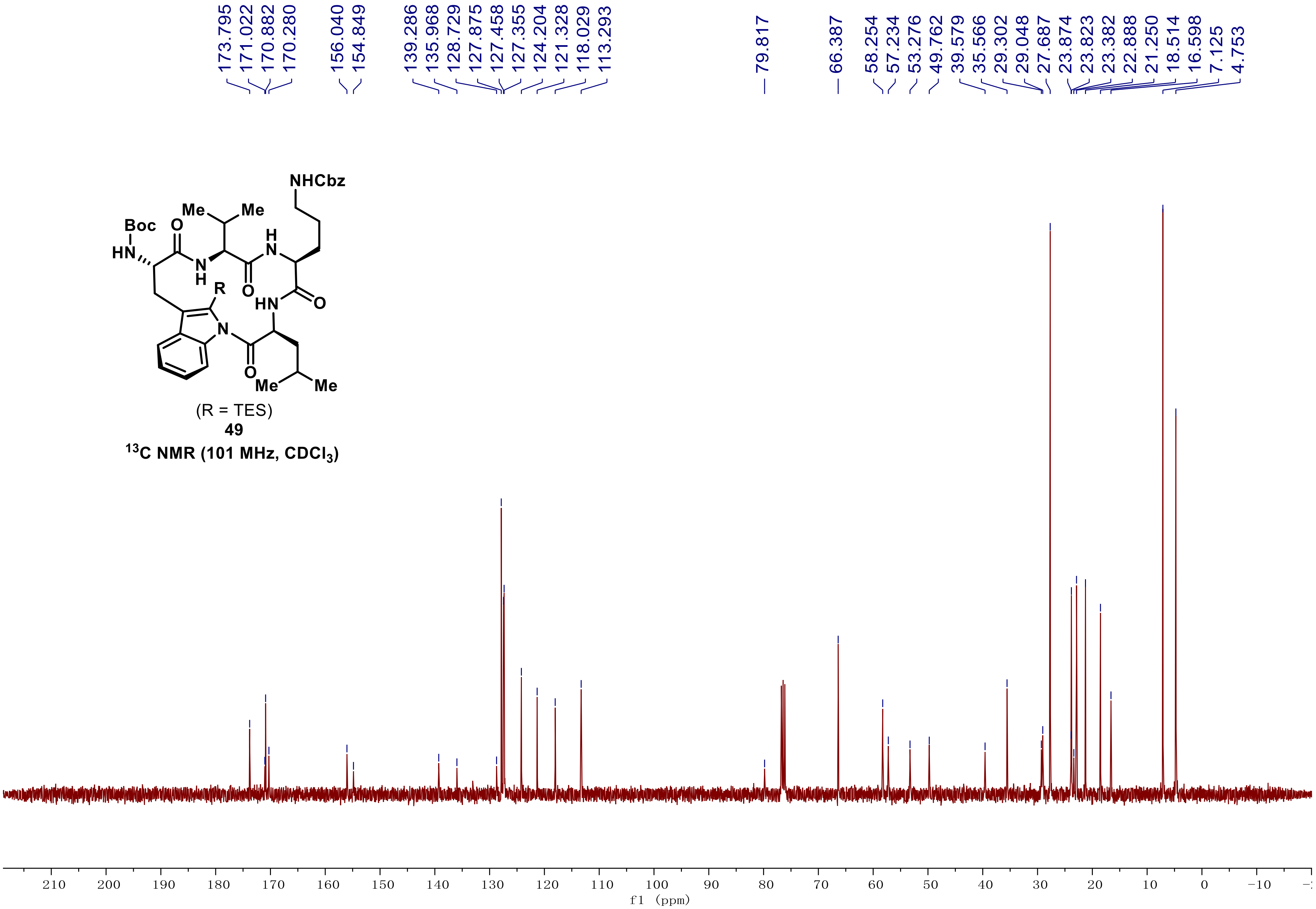

(R = TES)
49
^{13}C NMR (101 MHz, $CDCl_3$)

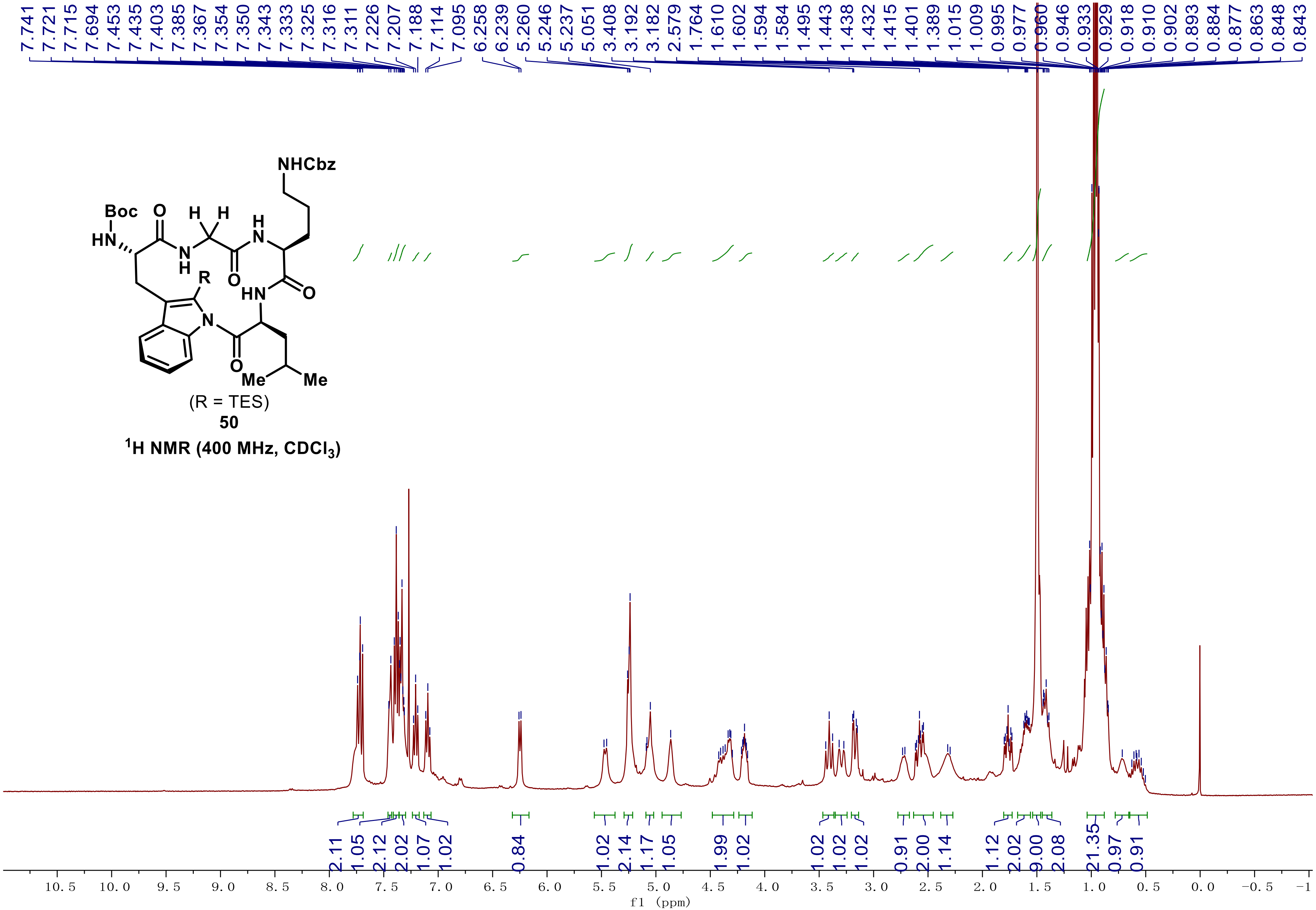
7.741 7.721 7.715 7.694 7.453 7.435 7.403 7.385 7.367 7.354 7.350 7.343 7.333 7.325 7.316 7.311 7.226 7.207 7.188 7.114 7.095 6.258 6.239 5.260 5.246 5.237 5.051 3.408 3.192 3.182 2.579 1.764 1.610 1.602 1.594 1.584 1.495 1.443 1.438 1.432 1.415 1.401 1.389 1.015 1.009 0.995 0.977 0.960 0.946 0.933 0.929 0.918 0.910 0.902 0.893 0.884 0.877 0.863 0.848 0.843
NHCbz
Boc
HN
O
H H
N
H
R
N
O
HN
O
N
O
Me
Me
(R = TES)
50
^{1}H NMR (400 MHz, $CDCl_3$)
2.11 1.05 2.12 2.02 1.07 1.02 0.84 1.02 2.14 1.17 1.05 1.99 1.02 1.02 1.02 1.02 0.91 2.00 1.14 1.12 2.02 9.00 2.08 21.35 0.97 0.91
10.5 10.0 9.5 9.0 8.5 8.0 7.5 7.0 6.5 6.0 5.5 5.0 4.5 4.0 3.5 3.0 2.5 2.0 1.5 1.0 0.5 0.0 −0.5 −1
f1 (ppm)

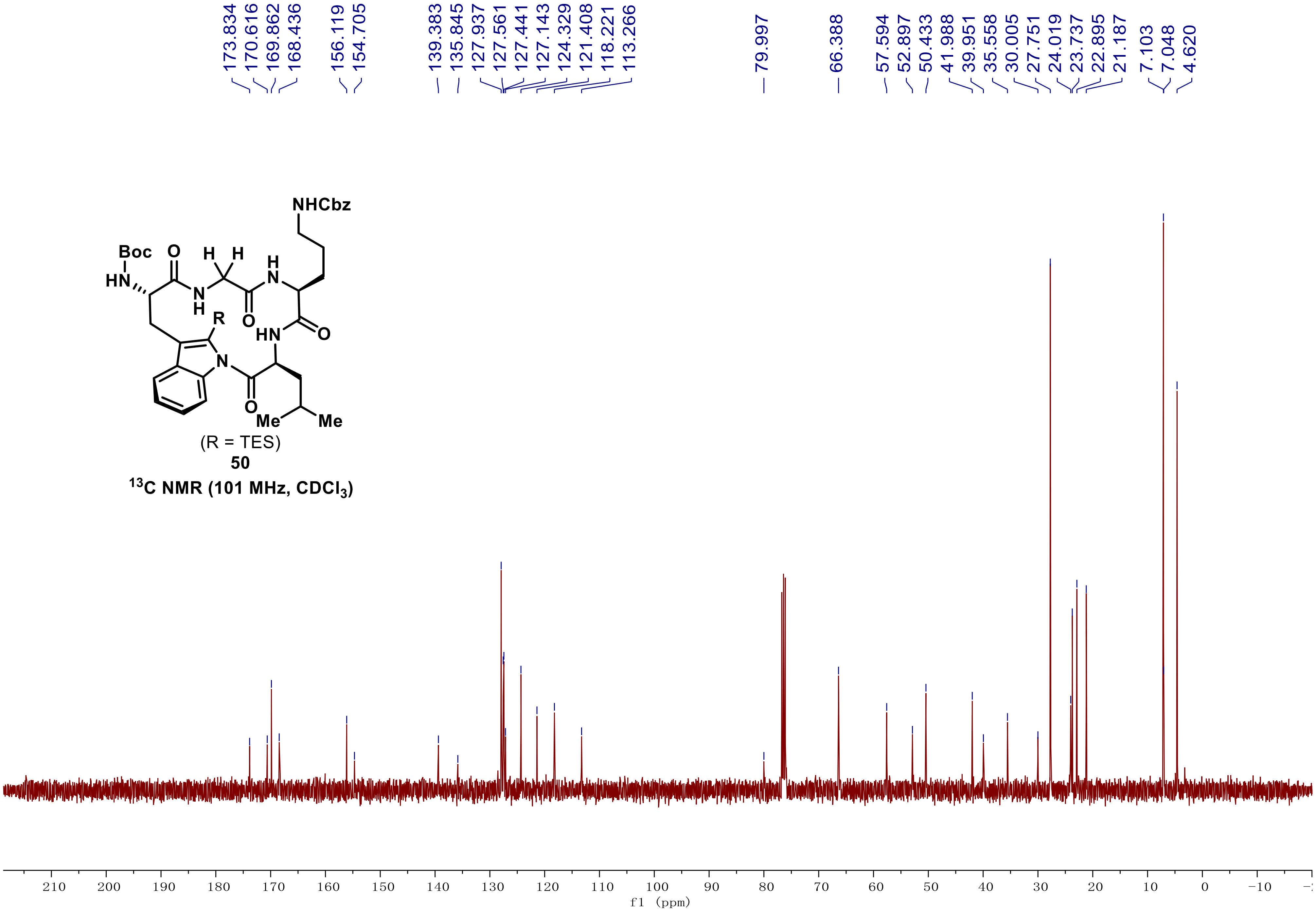

(R = TES)
50
^{13}C NMR (101 MHz, $CDCl_3$)

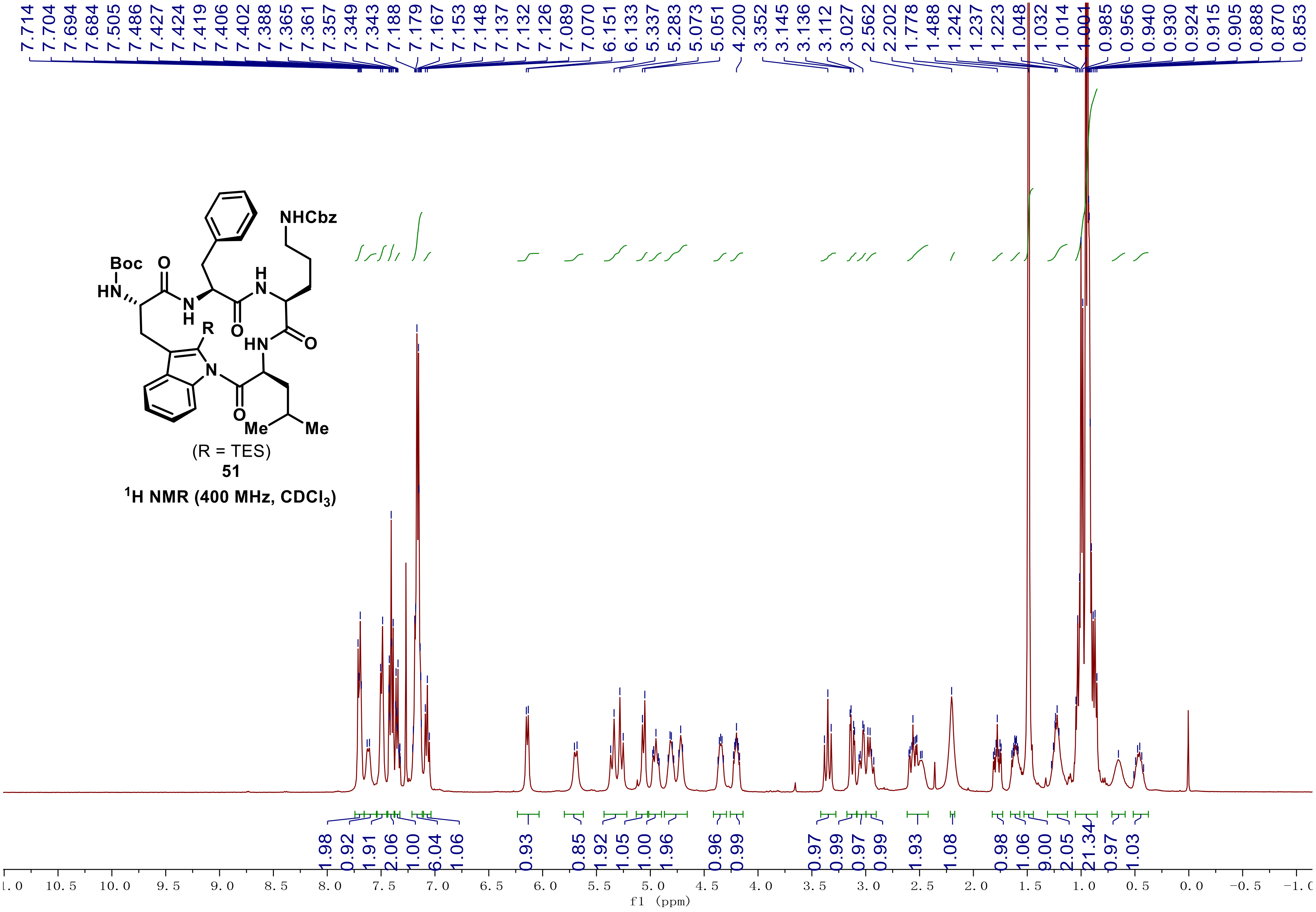

NHCbz
Boc
HN
O
N
H
R
N
HN
O
N
O
O
Me
Me
(R = TES)
51
1H NMR (400 MHz, CDCl3)
f1 (ppm)

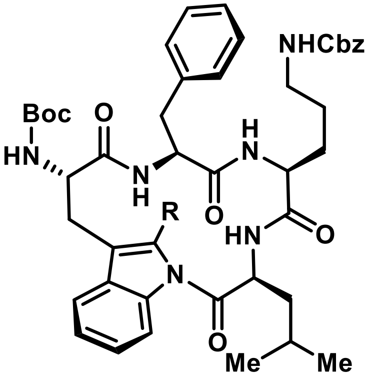

(R = TES)

51

^{13}C NMR (101 MHz, $CDCl_3$)

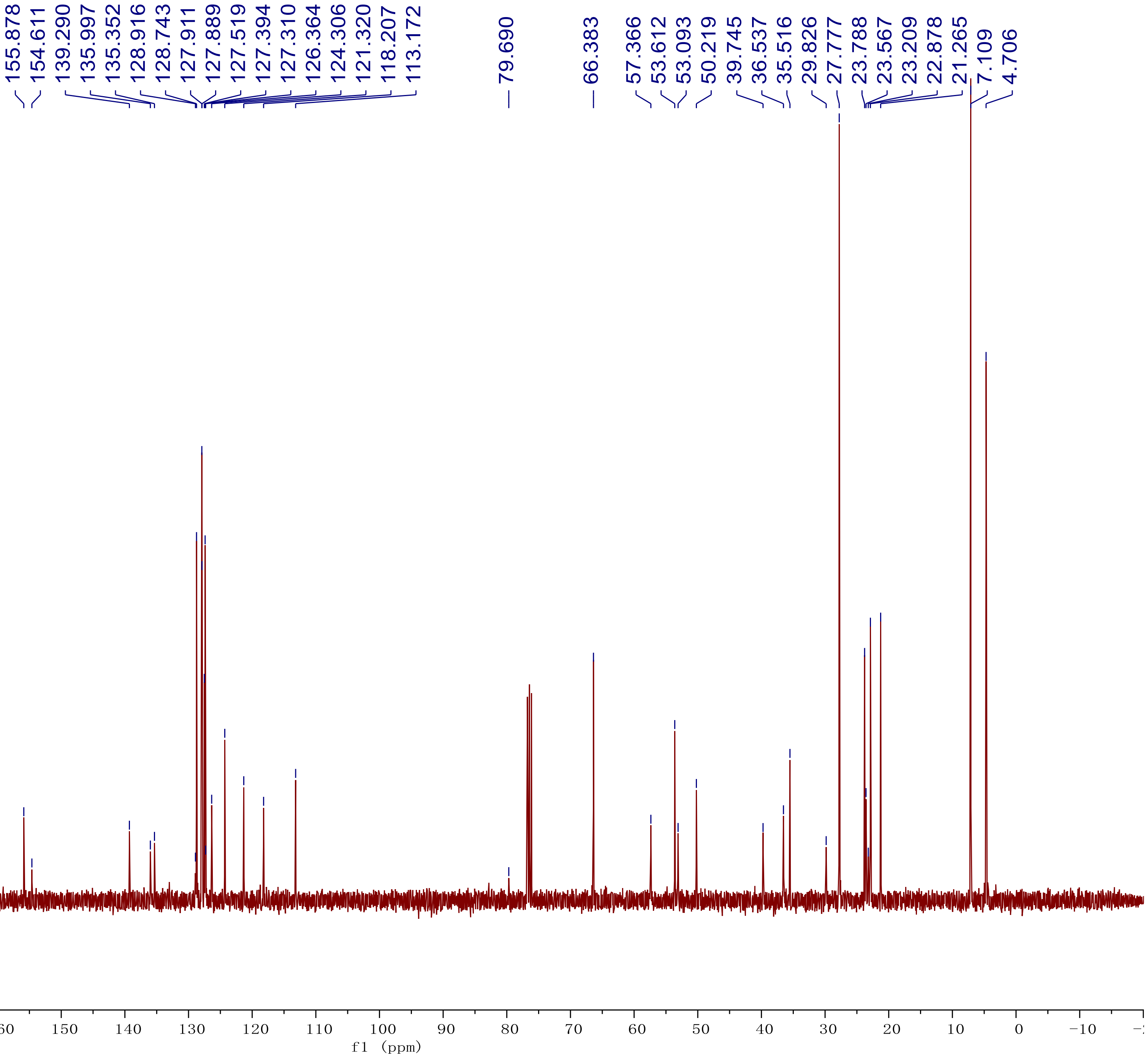

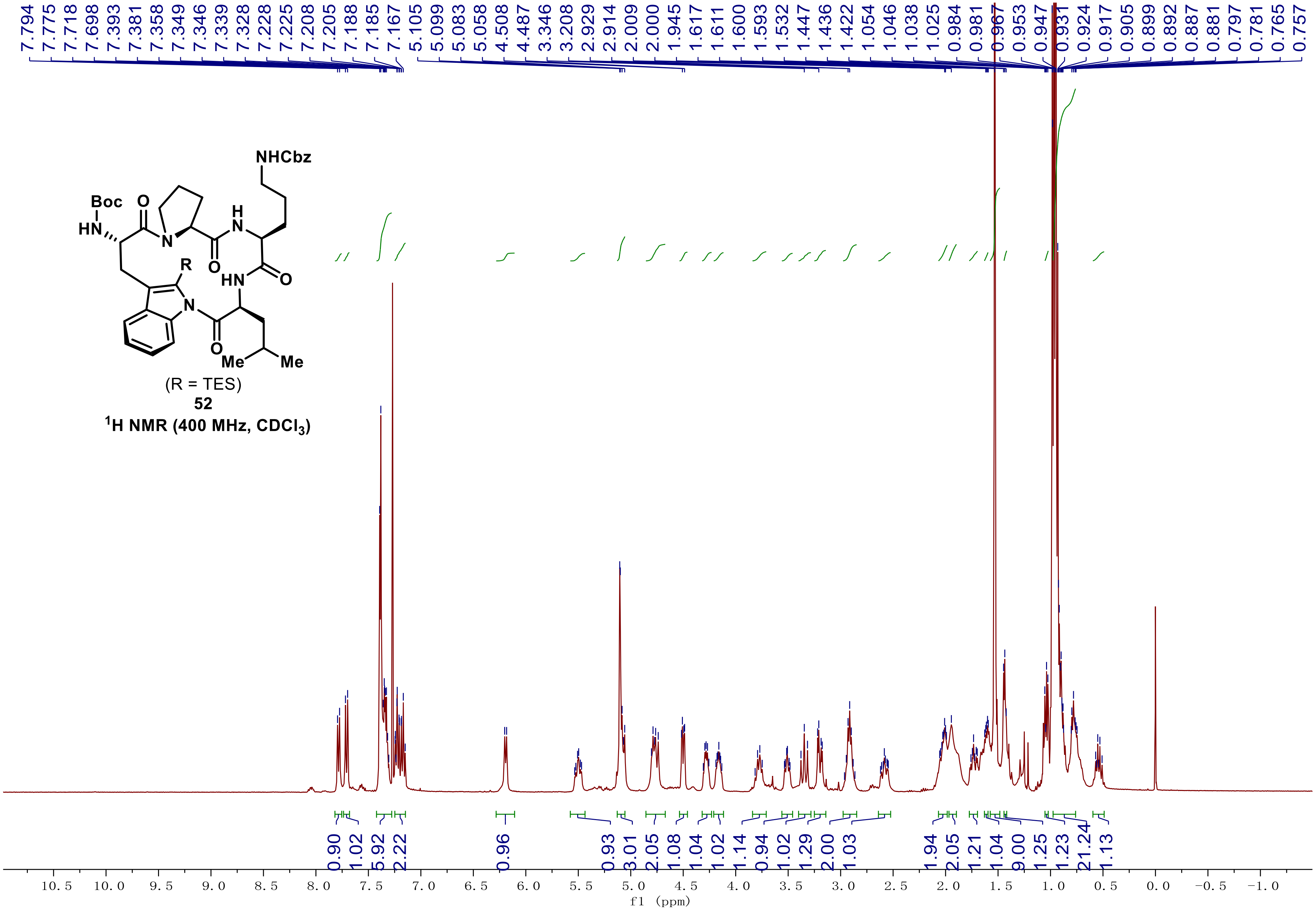
7.794 7.775 7.718 7.698 7.393 7.381 7.358 7.349 7.346 7.339 7.328 7.228 7.225 7.208 7.205 7.188 7.185 7.167 5.105 5.099 5.083 5.058 4.508 4.487 3.346 3.208 2.929 2.914 2.009 2.000 1.945 1.617 1.611 1.600 1.593 1.532 1.447 1.436 1.422 1.054 1.046 1.038 1.025 0.984 0.981 0.967 0.953 0.947 0.931 0.924 0.917 0.905 0.899 0.892 0.887 0.881 0.797 0.781 0.765 0.757
NHCbz
Boc
HN
O
N
H
N
R
O
HN
O
N
O
Me
Me
(R = TES)
52
1H NMR (400 MHz, CDCl3)
0.90 1.02 5.92 2.22 0.96 0.93 3.01 2.05 1.08 1.04 1.02 1.14 0.94 1.02 1.29 2.00 1.03 1.94 2.05 1.21 1.04 9.00 1.25 1.23 21.24 1.13
10.5 10.0 9.5 9.0 8.5 8.0 7.5 7.0 6.5 6.0 5.5 5.0 4.5 4.0 3.5 3.0 2.5 2.0 1.5 1.0 0.5 0.0 -0.5 -1.0
f1 (ppm)

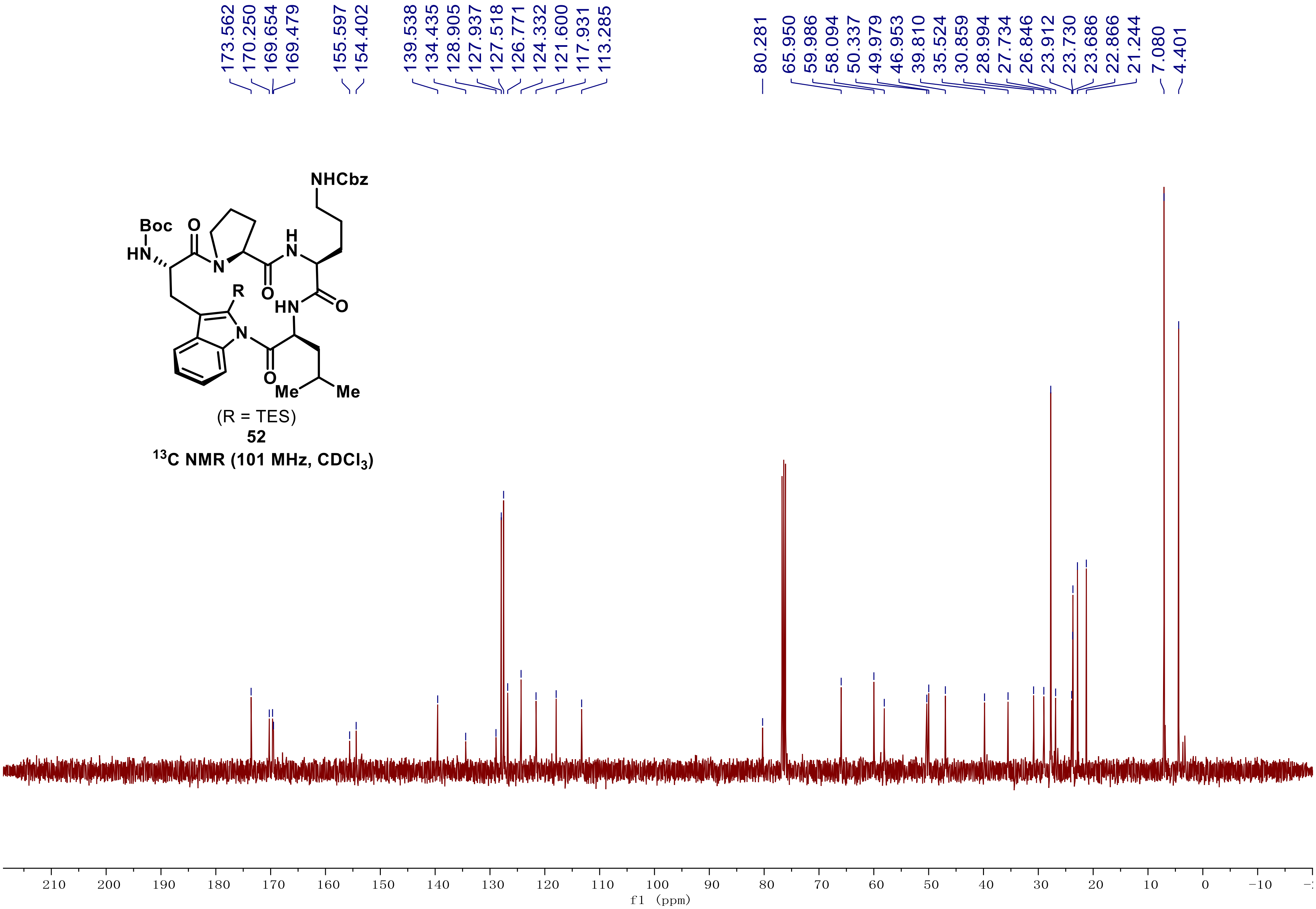
173.562
170.250
169.654
169.479
155.597
154.402
139.538
134.435
128.905
127.937
127.518
126.771
124.332
121.600
117.931
113.285
80.281
65.950
59.986
58.094
50.337
49.979
46.953
39.810
35.524
30.859
28.994
27.734
26.846
23.912
23.730
23.686
22.866
21.244
7.080
4.401
NHCbz
Boc
HN
O
N
H
N
R
O
HN
O
N
O
Me
Me
(R = TES)
52
13C NMR (101 MHz, CDCl3)
f1 (ppm)

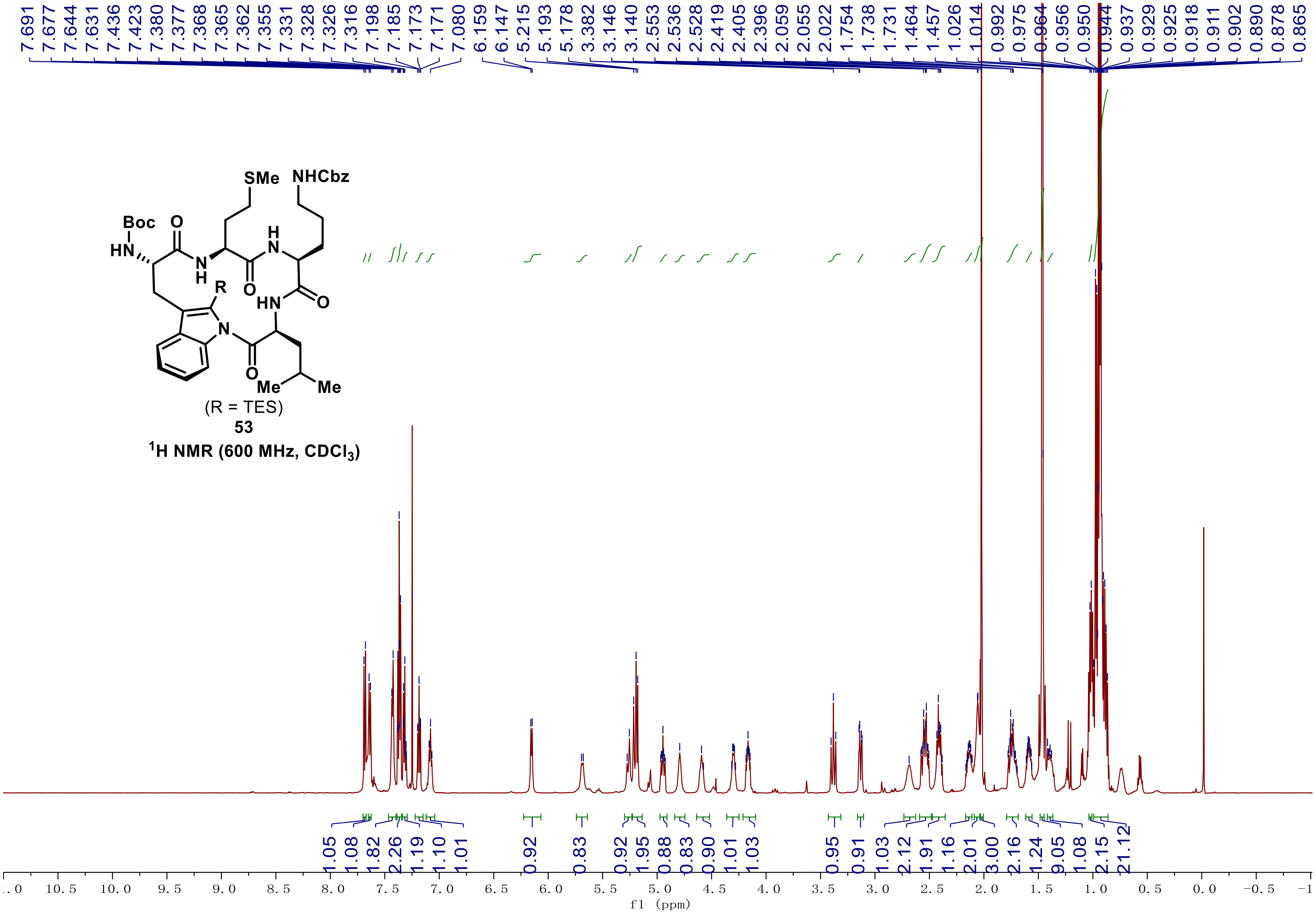

(R = TES)
53
^{1}H NMR (600 MHz, $CDCl_3$)

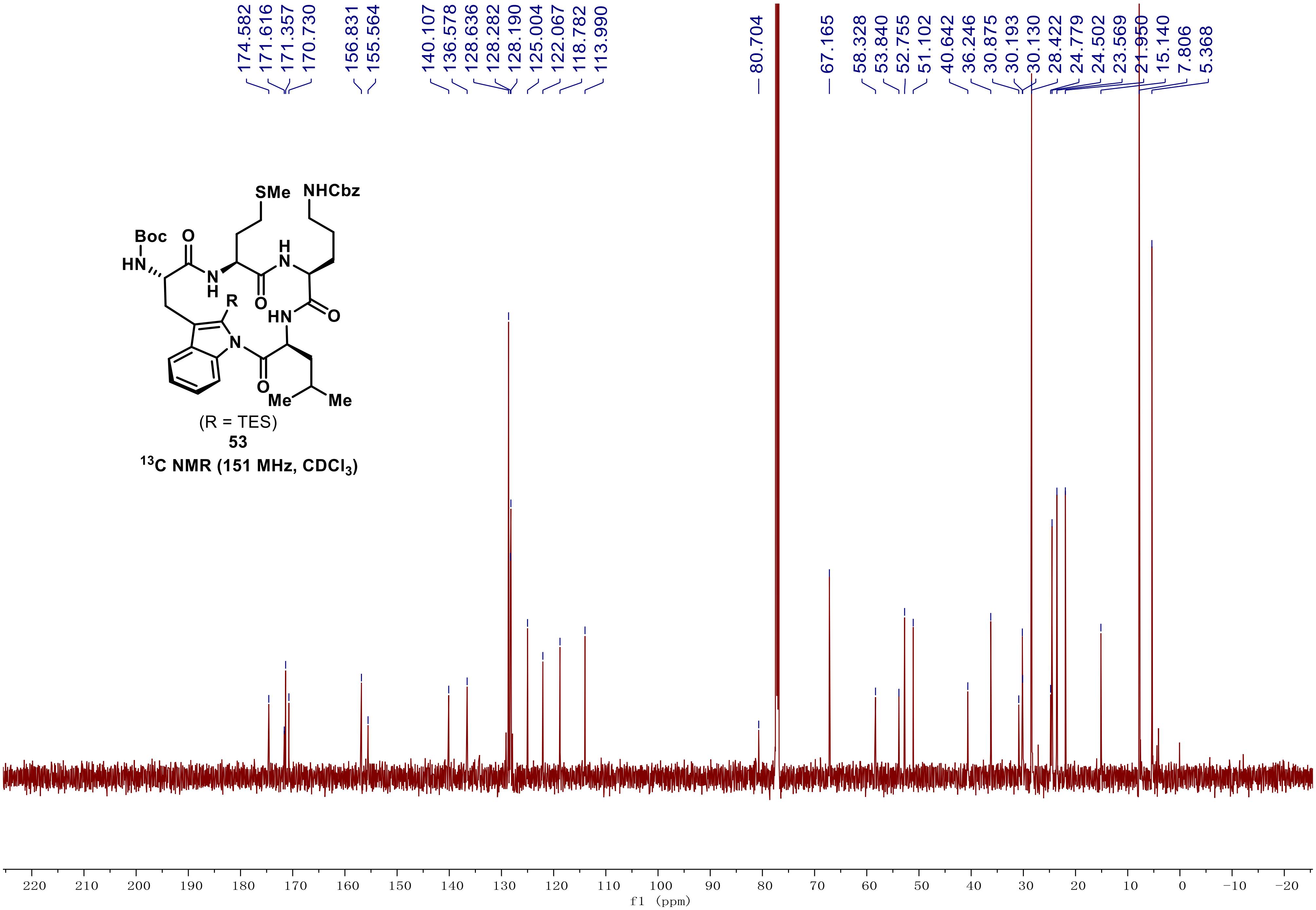

174.582
171.616
171.357
170.730
156.831
155.564
140.107
136.578
128.636
128.282
128.190
125.004
122.067
118.782
113.990
80.704
67.165
58.328
53.840
52.755
51.102
40.642
36.246
30.875
30.193
30.130
28.422
24.779
24.502
23.569
21.950
15.140
7.806
5.368
SMe
NHCbz
Boc
HN
O
N
H
R
N
HN
Me
(R = TES)
53
^{13}C NMR (151 MHz, $CDCl_3$)
220 210 200 190 180 170 160 150 140 130 120 110 100 90 80 70 60 50 40 30 20 10 0 −10 −20
f1 (ppm)

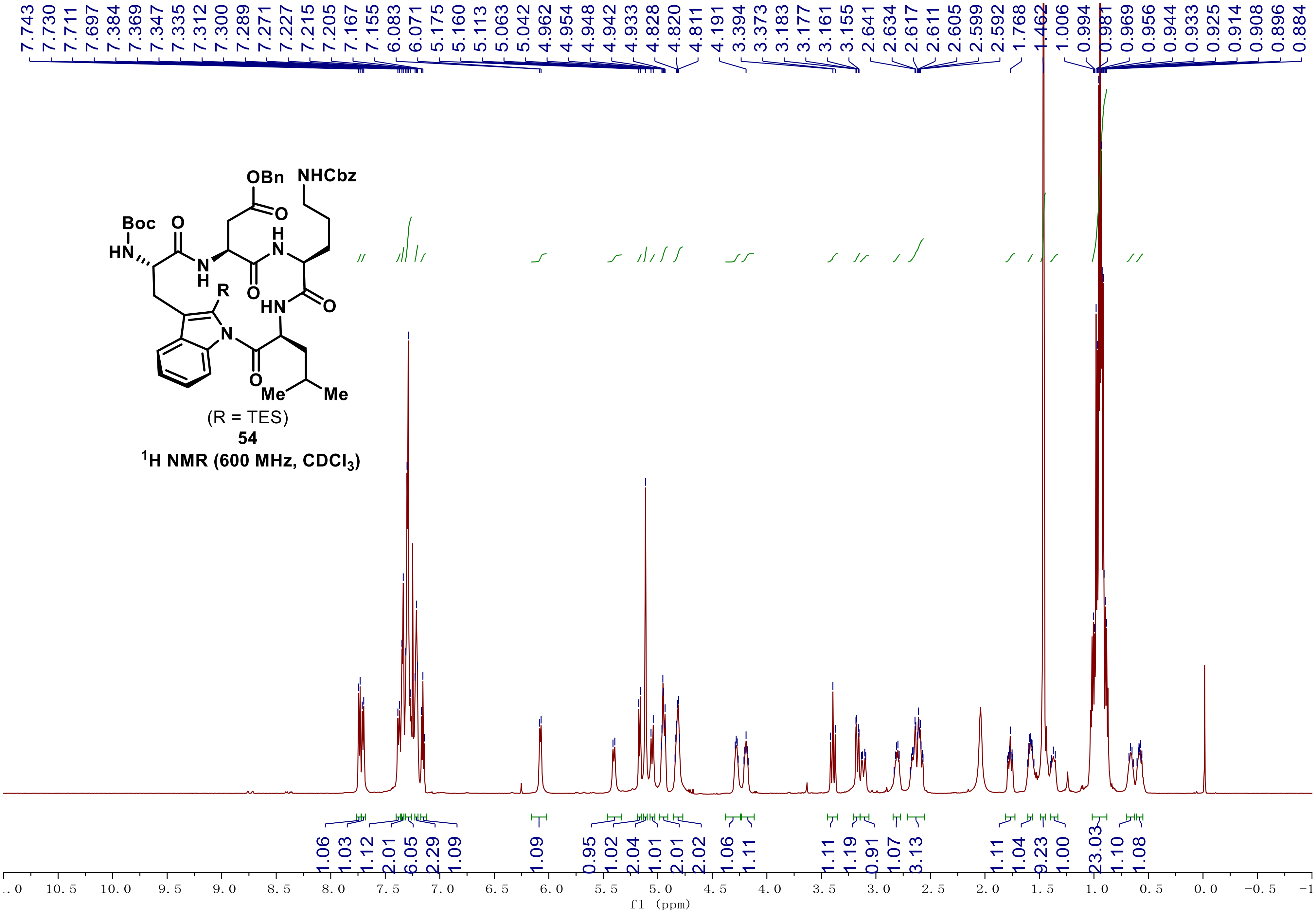
OBn
NHCbz
Boc
HN
O
H
N
R
(R = TES)
54
1H NMR (600 MHz, CDCl3)
f1 (ppm)

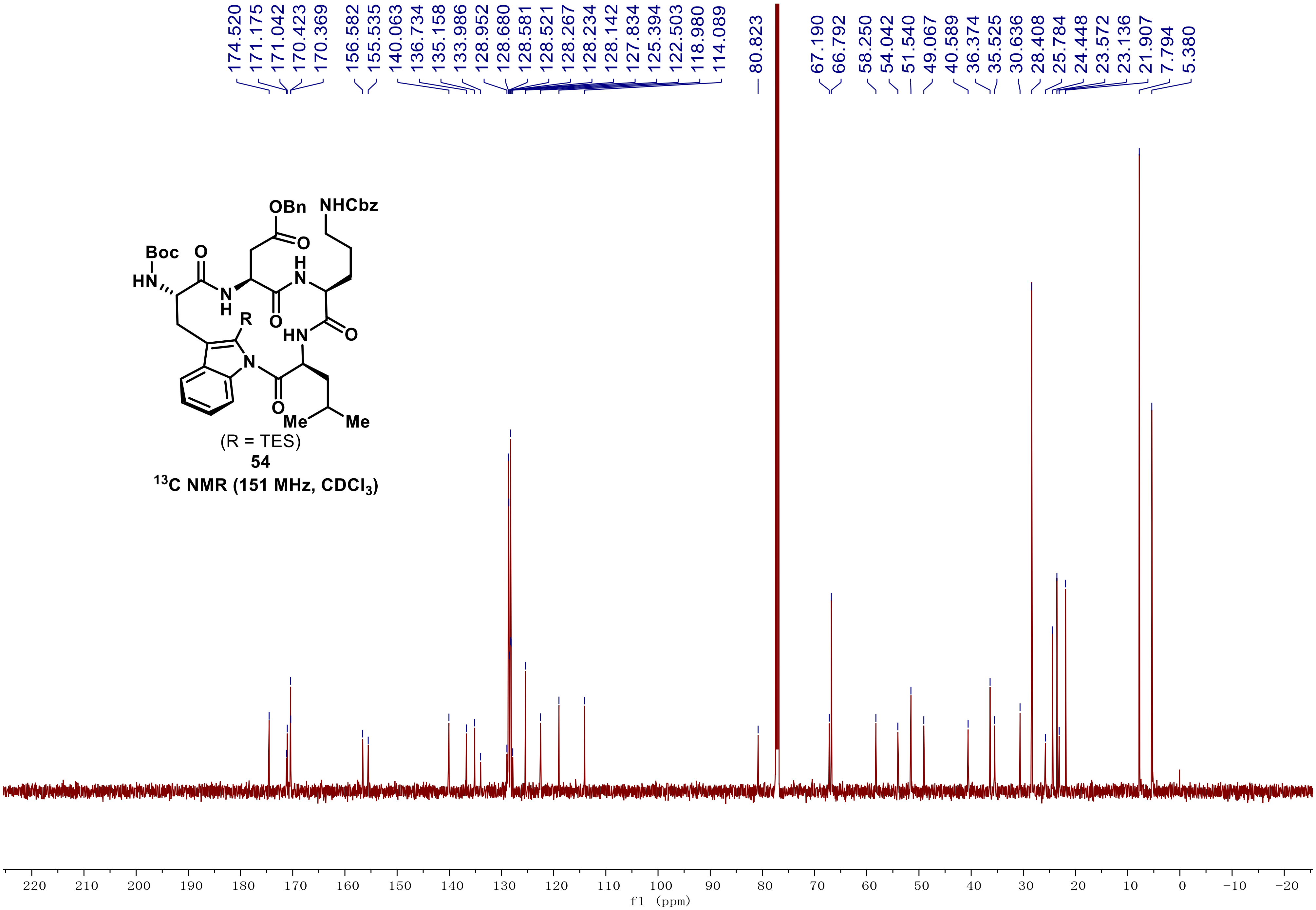

(R = TES)

54

^{13}C NMR (151 MHz, $CDCl_3$)

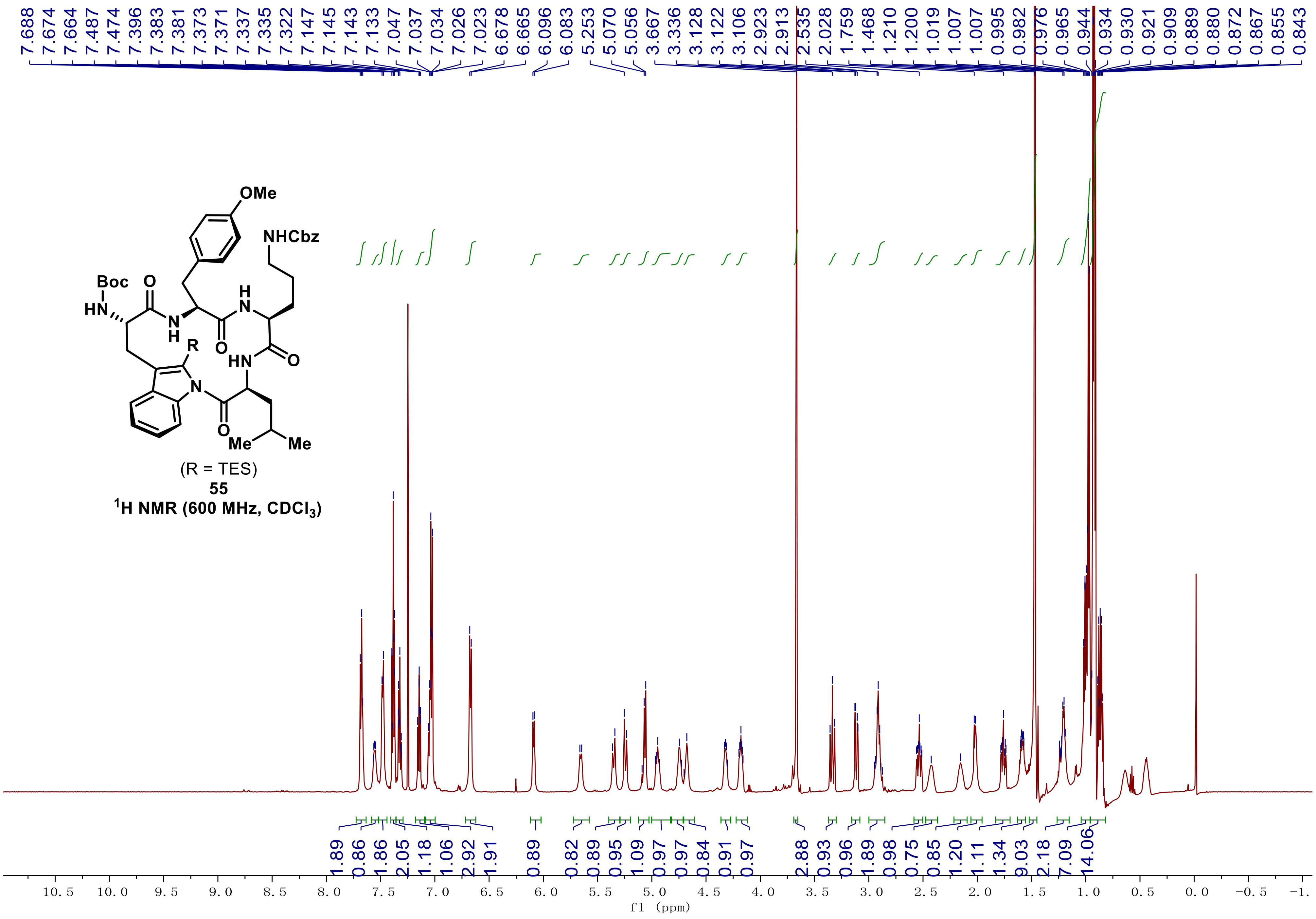

(R = TES)

55

^{1}H NMR (600 MHz, $CDCl_3$)

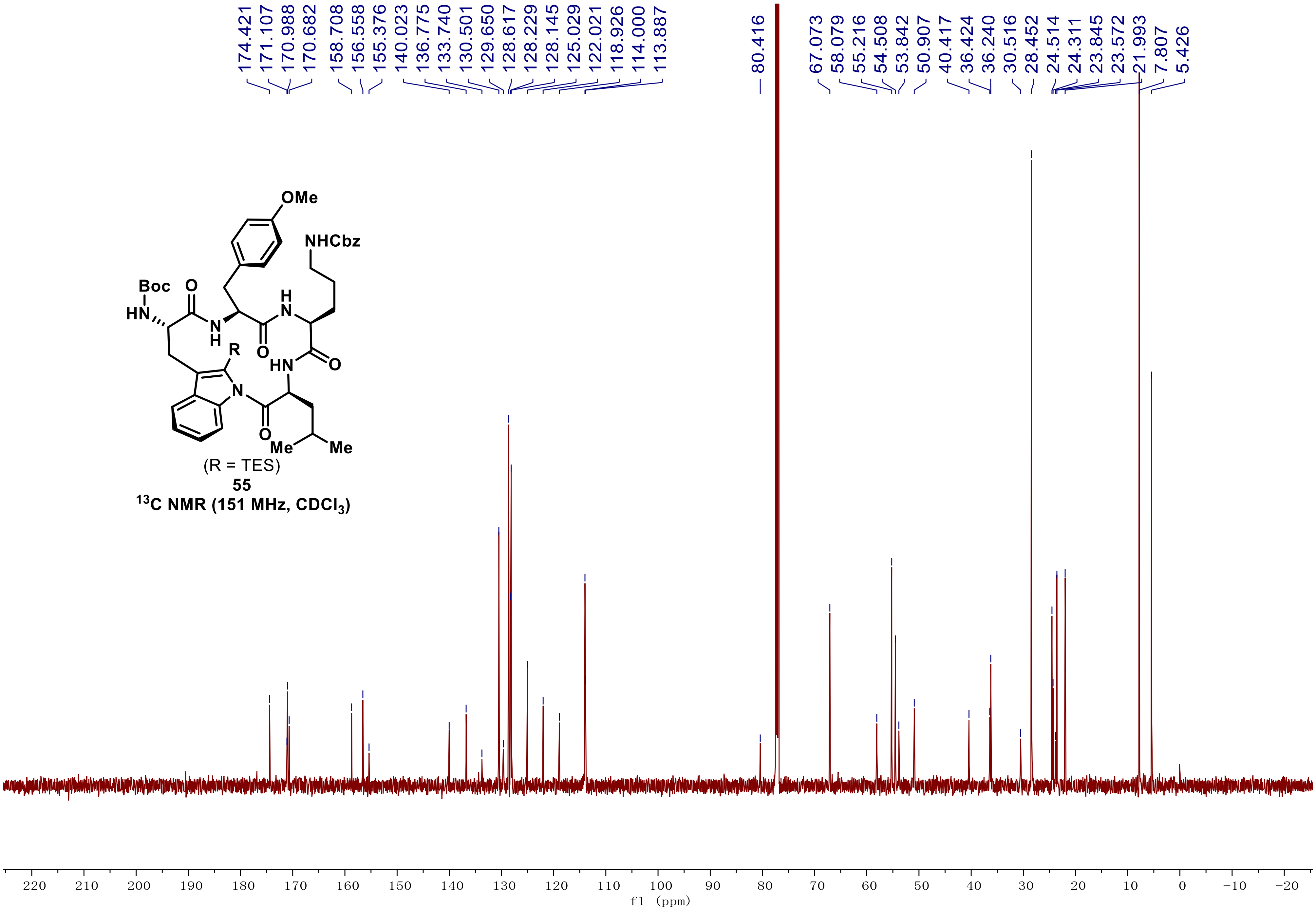
174.421
171.107
170.988
170.682
158.708
156.558
155.376
140.023
136.775
133.740
130.501
129.650
128.617
128.229
128.145
125.029
122.021
118.926
114.000
113.887
80.416
67.073
58.079
55.216
54.508
53.842
50.907
40.417
36.424
36.240
30.516
28.452
24.514
24.311
23.845
23.572
21.993
7.807
5.426
OMe
NHCbz
Boc
HN
O
N
H
R
HN
N
Me
Me
(R = TES)
55
^{13}C NMR (151 MHz, $CDCl_3$)
220 210 200 190 180 170 160 150 140 130 120 110 100 90 80 70 60 50 40 30 20 10 0 −10 −20
f1 (ppm)

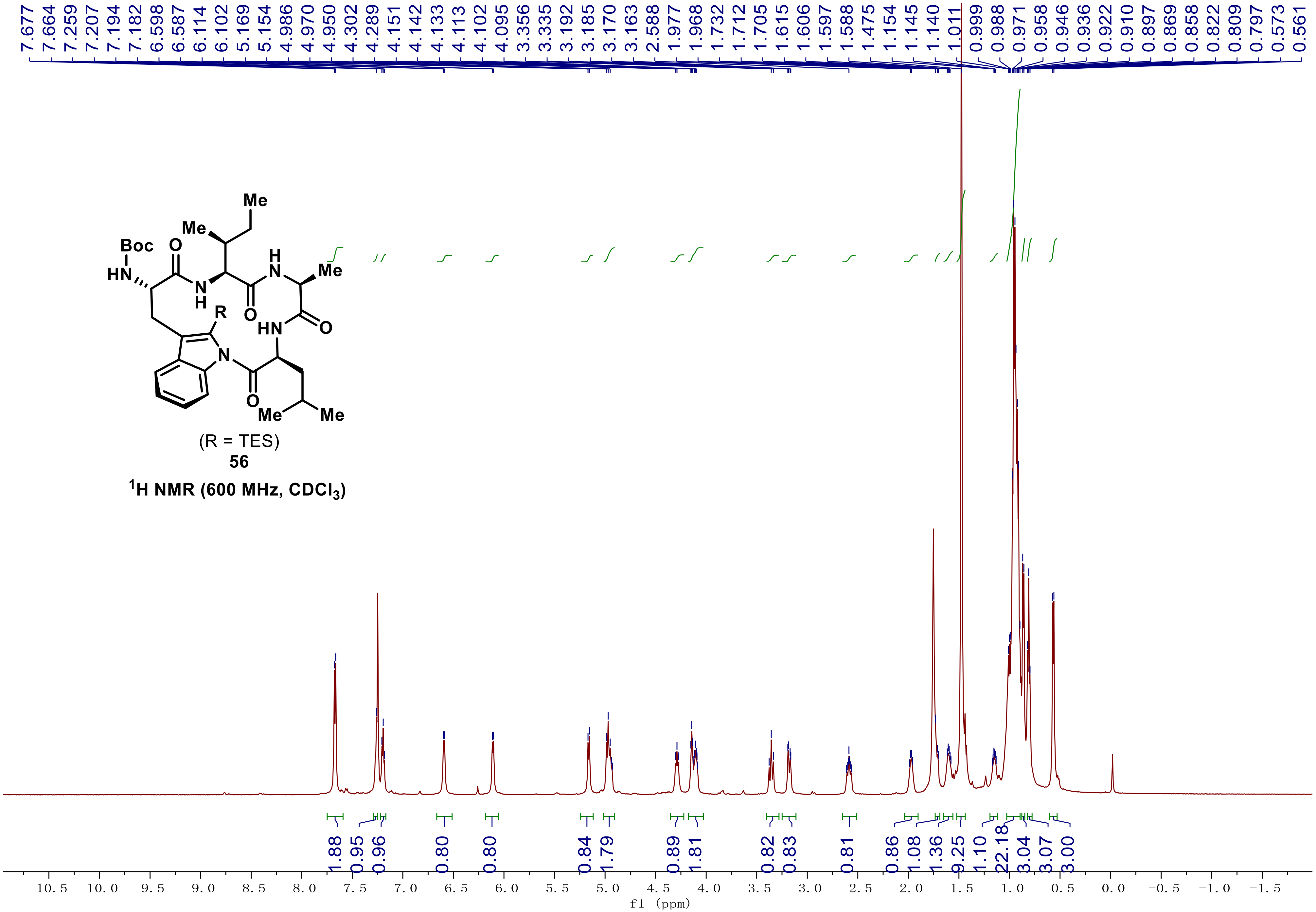

(R = TES)
56
^{1}H NMR (600 MHz, CDCl$_3$)

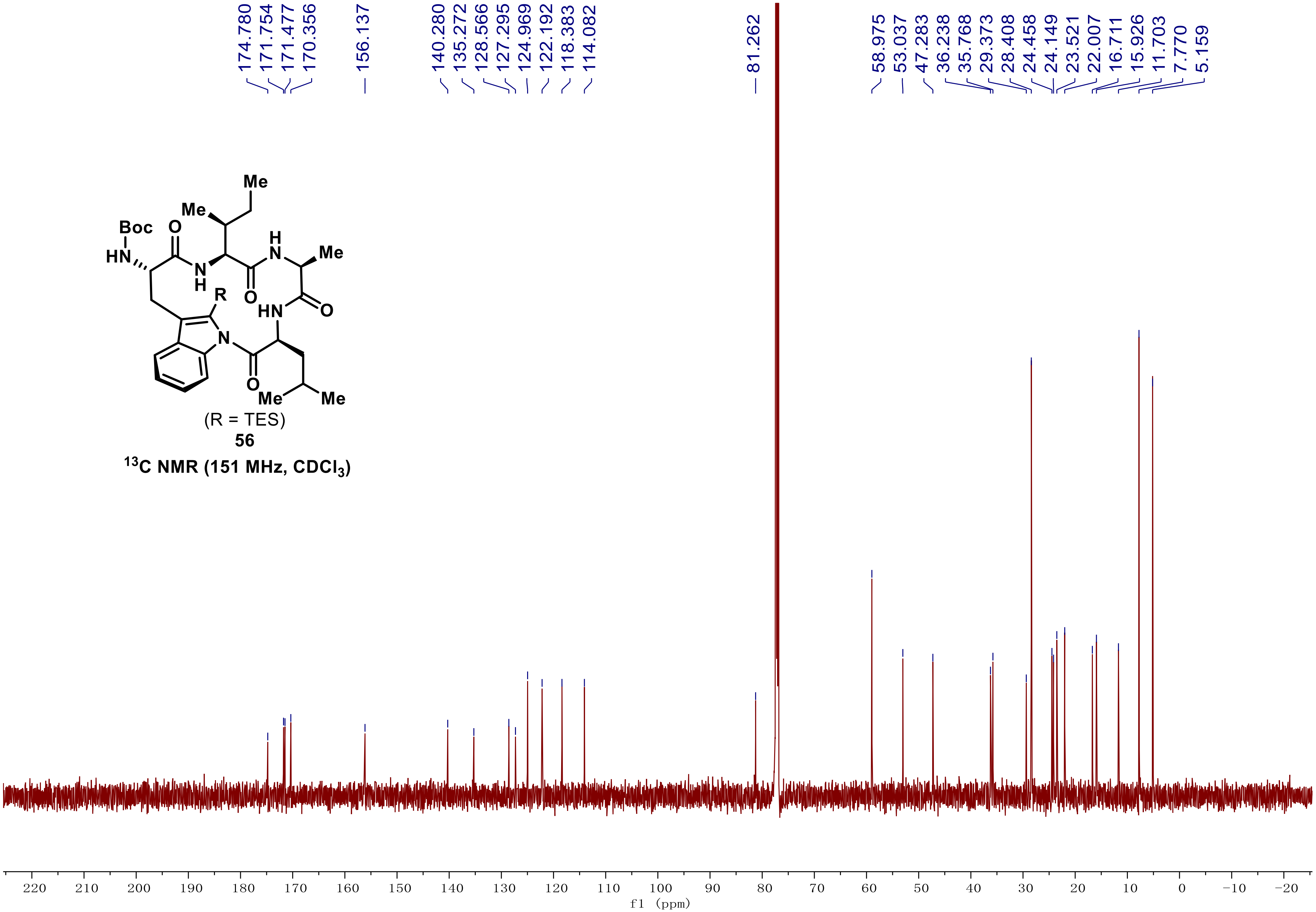

(R = TES)
56
^{13}C NMR (151 MHz, CDCl$_3$)

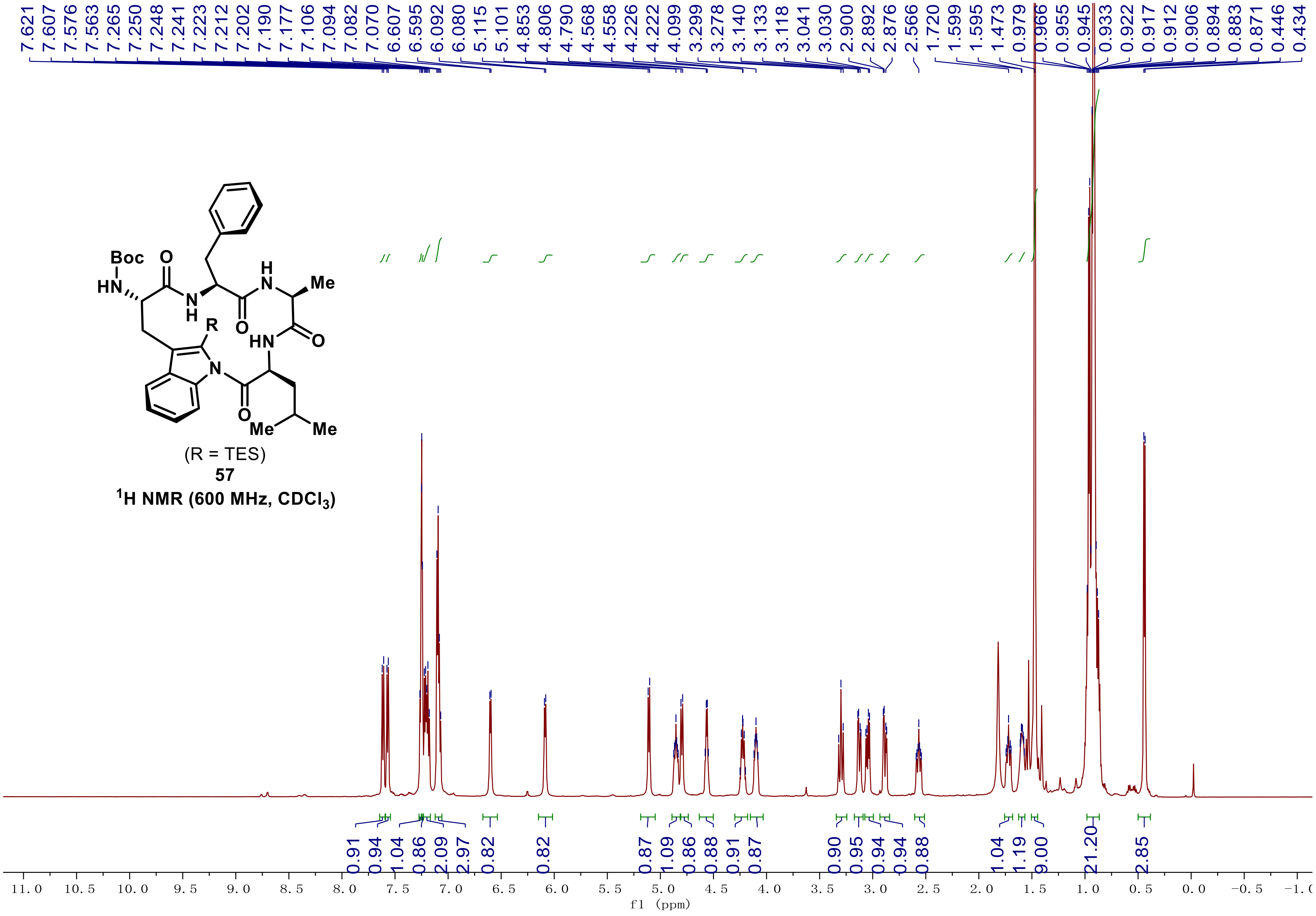

7.621 7.607 7.576 7.563 7.265 7.250 7.248 7.241 7.223 7.212 7.202 7.190 7.177 7.106 7.094 7.082 7.070 6.607 6.595 6.092 6.080 5.115 5.101 4.853 4.806 4.790 4.568 4.558 4.226 4.222 4.099 3.299 3.278 3.140 3.133 3.118 3.041 3.030 2.900 2.892 2.876 2.566 1.720 1.599 1.595 1.473 0.979 0.966 0.955 0.945 0.933 0.922 0.917 0.912 0.906 0.894 0.883 0.871 0.446 0.434
Boc
HN
O
N
H
H
N
Me
R
O
HN
O
N
O
Me
Me
(R = TES)
57
1H NMR (600 MHz, CDCl3)
0.91 0.94 1.04 0.86 2.09 2.97 0.82 0.82 0.87 1.09 0.86 0.88 0.91 0.87 0.90 0.95 0.94 0.94 0.88 1.04 1.19 9.00 21.20 2.85
11.0 10.5 10.0 9.5 9.0 8.5 8.0 7.5 7.0 6.5 6.0 5.5 5.0 4.5 4.0 3.5 3.0 2.5 2.0 1.5 1.0 0.5 0.0 -0.5 -1.0
f1 (ppm)

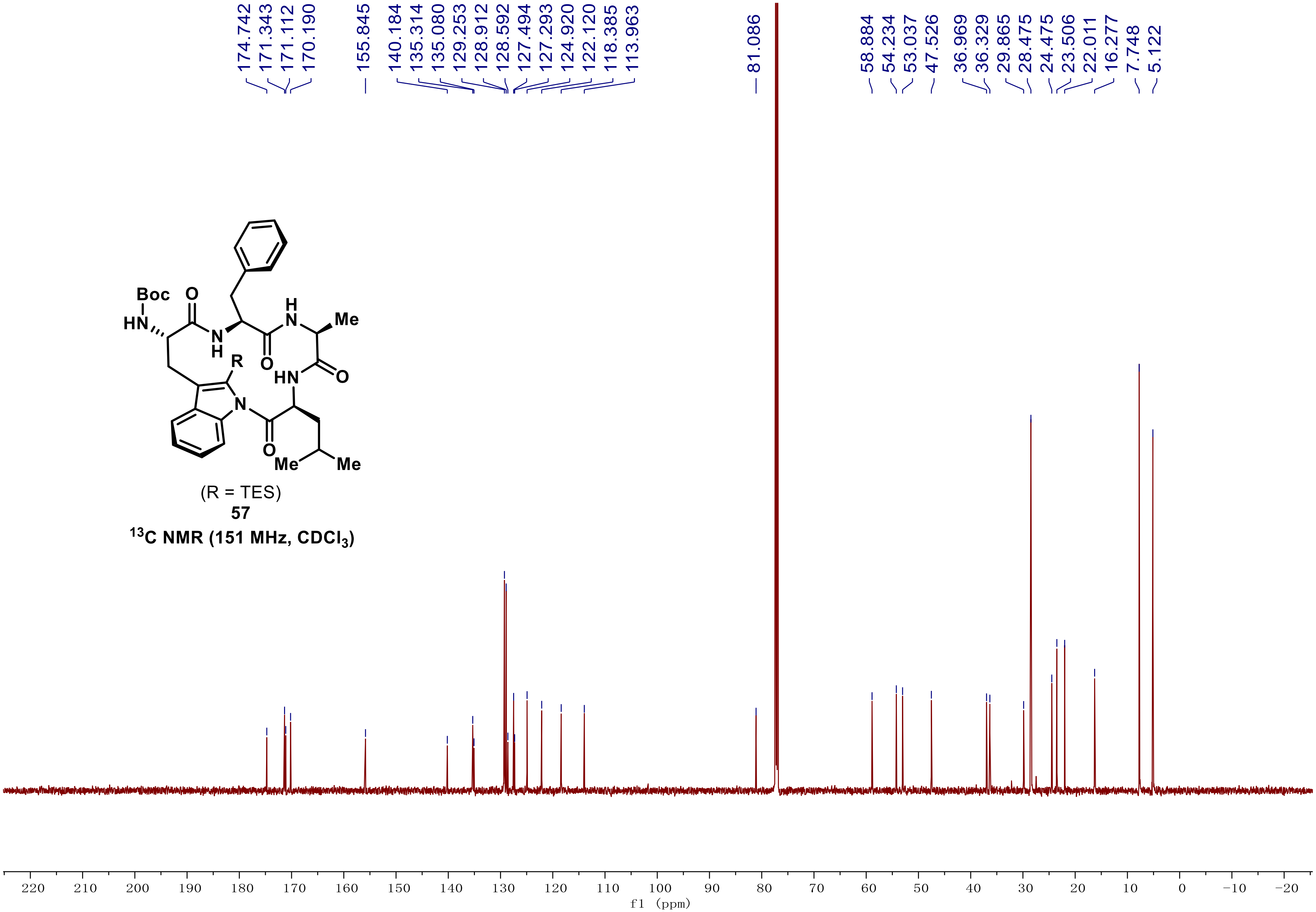

(R = TES)
57
^{13}C NMR (151 MHz, $CDCl_3$)

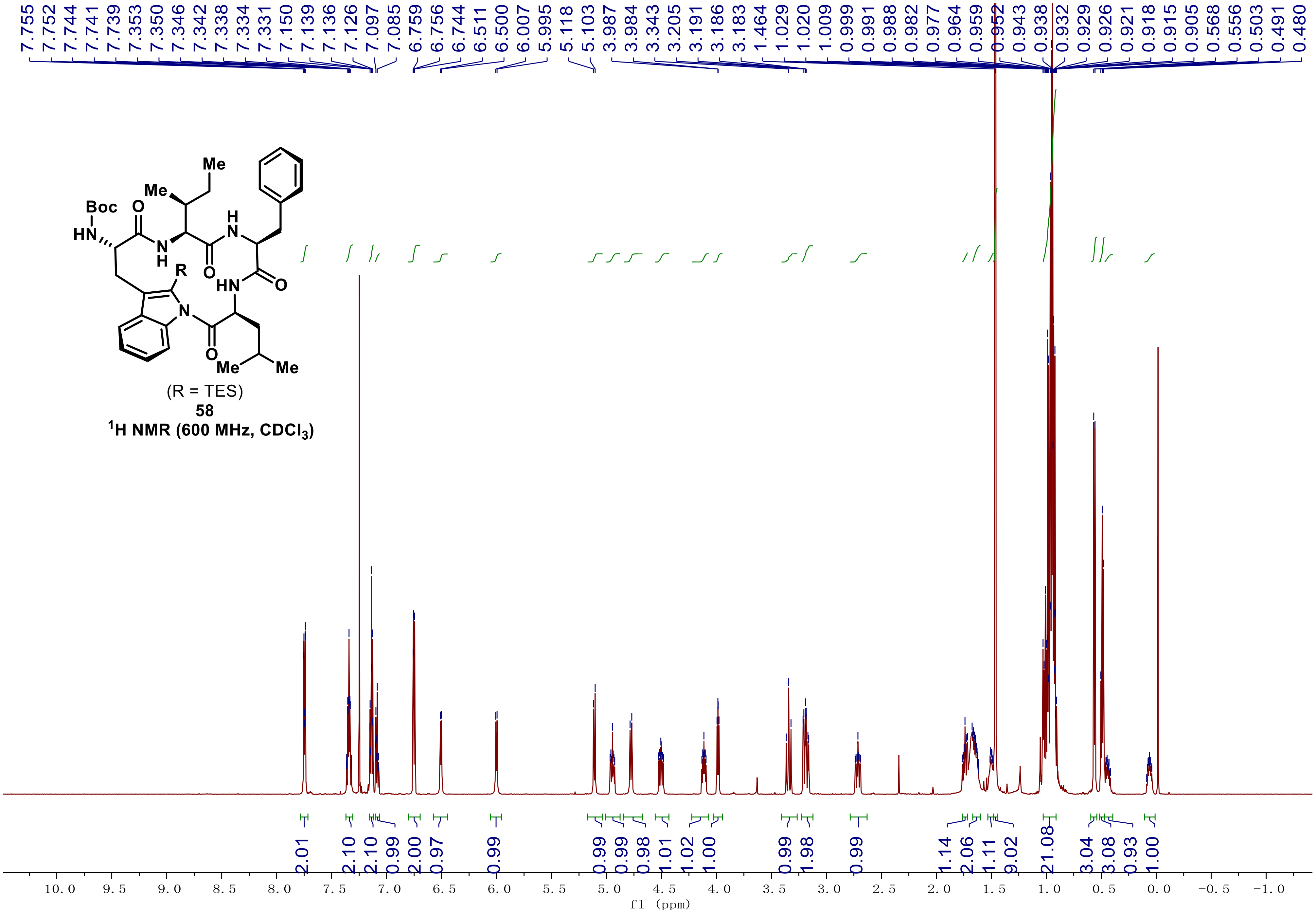
7.755 7.752 7.744 7.741 7.739 7.353 7.350 7.346 7.342 7.338 7.334 7.331 7.150 7.139 7.136 7.126 7.097 7.085 6.759 6.756 6.744 6.511 6.500 6.007 5.995 5.118 5.103 3.987 3.984 3.343 3.205 3.191 3.186 3.183 1.464 1.029 1.020 1.009 0.999 0.991 0.988 0.982 0.977 0.964 0.959 0.952 0.943 0.938 0.932 0.929 0.926 0.921 0.918 0.915 0.905 0.568 0.556 0.503 0.491 0.480
Boc
HN
O
N
H
Me
Me
H
N
O
R
N
HN
O
O
Me
Me
(R = TES)
58
^{1}H NMR (600 MHz, $CDCl_3$)
2.01 2.10 2.10 0.99 2.00 0.97 0.99 0.99 0.99 0.98 1.01 1.02 1.00 0.99 1.98 0.99 1.14 2.06 1.11 9.02 21.08 3.04 3.08 0.93 1.00
10.0 9.5 9.0 8.5 8.0 7.5 7.0 6.5 6.0 5.5 5.0 4.5 4.0 3.5 3.0 2.5 2.0 1.5 1.0 0.5 0.0 −0.5 −1.0
f1 (ppm)

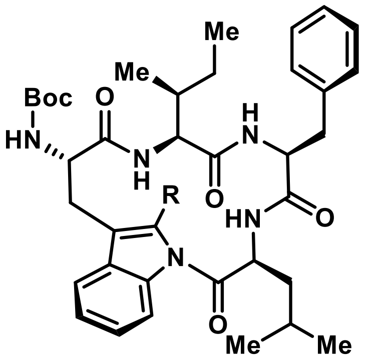

(R = TES)

58

^{13}C NMR (151 MHz, $CDCl_3$)

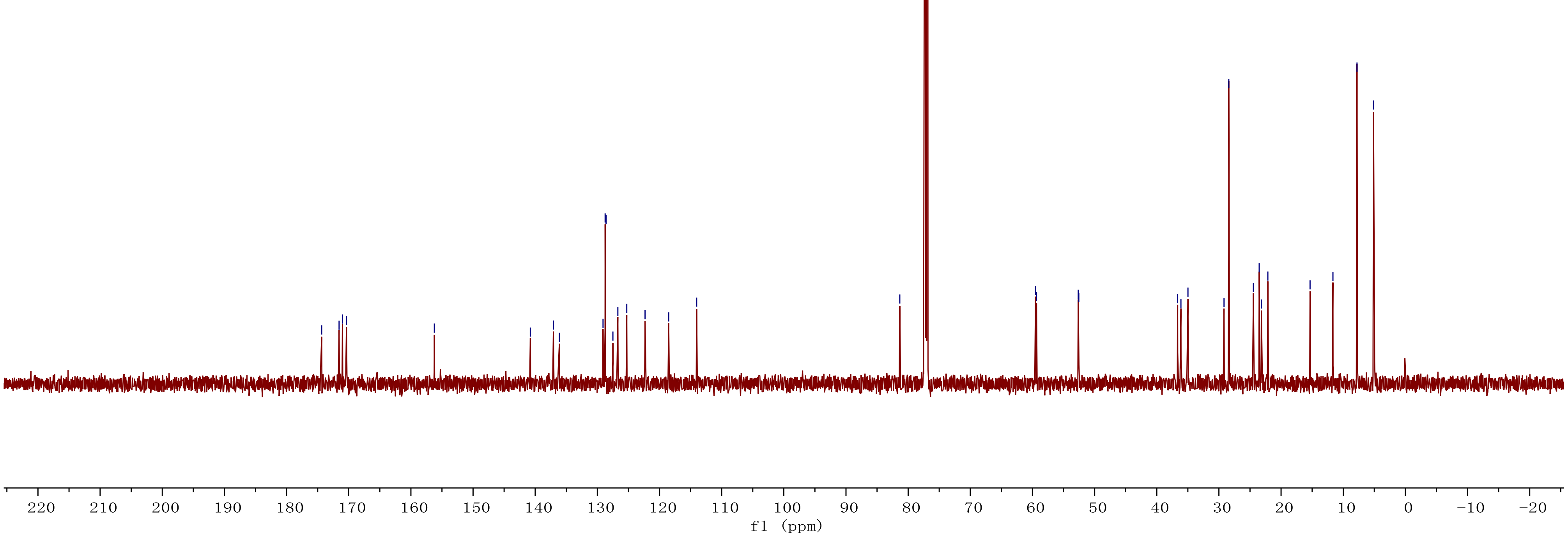

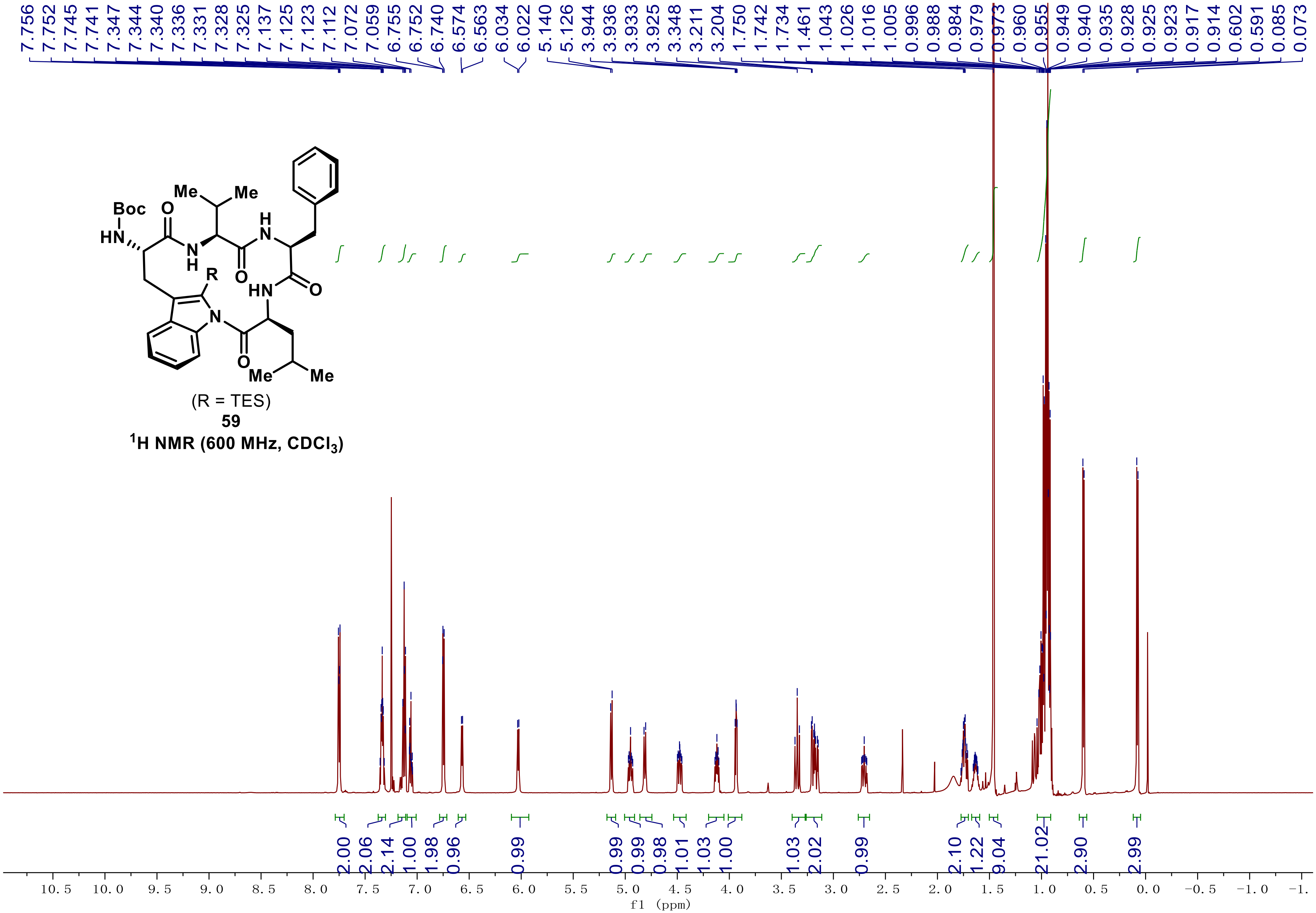

(R = TES)

59

^{1}H NMR (600 MHz, $CDCl_3$)

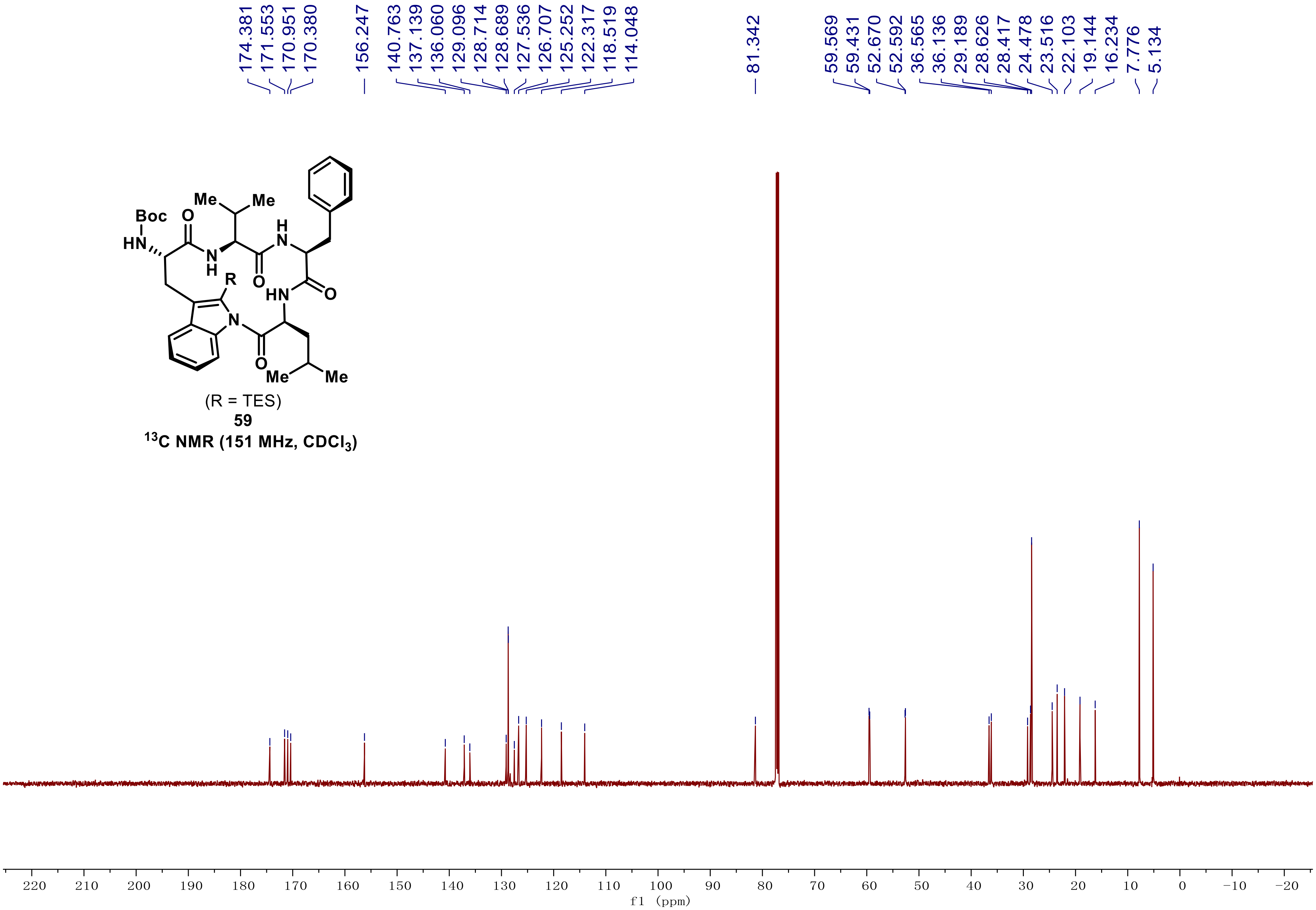

174.381
171.553
170.951
170.380
156.247
140.763
137.139
136.060
129.096
128.714
128.689
127.536
126.707
125.252
122.317
118.519
114.048
81.342
59.569
59.431
52.670
52.592
36.565
36.136
29.189
28.626
28.417
24.478
23.516
22.103
19.144
16.234
7.776
5.134
Boc
HN
O
Me
Me
N
H
H
N
O
R
HN
O
N
O
Me
Me
(R = TES)
59
^{13}C NMR (151 MHz, CDCl$_3$)
220 210 200 190 180 170 160 150 140 130 120 110 100 90 80 70 60 50 40 30 20 10 0 −10 −20
f1 (ppm)

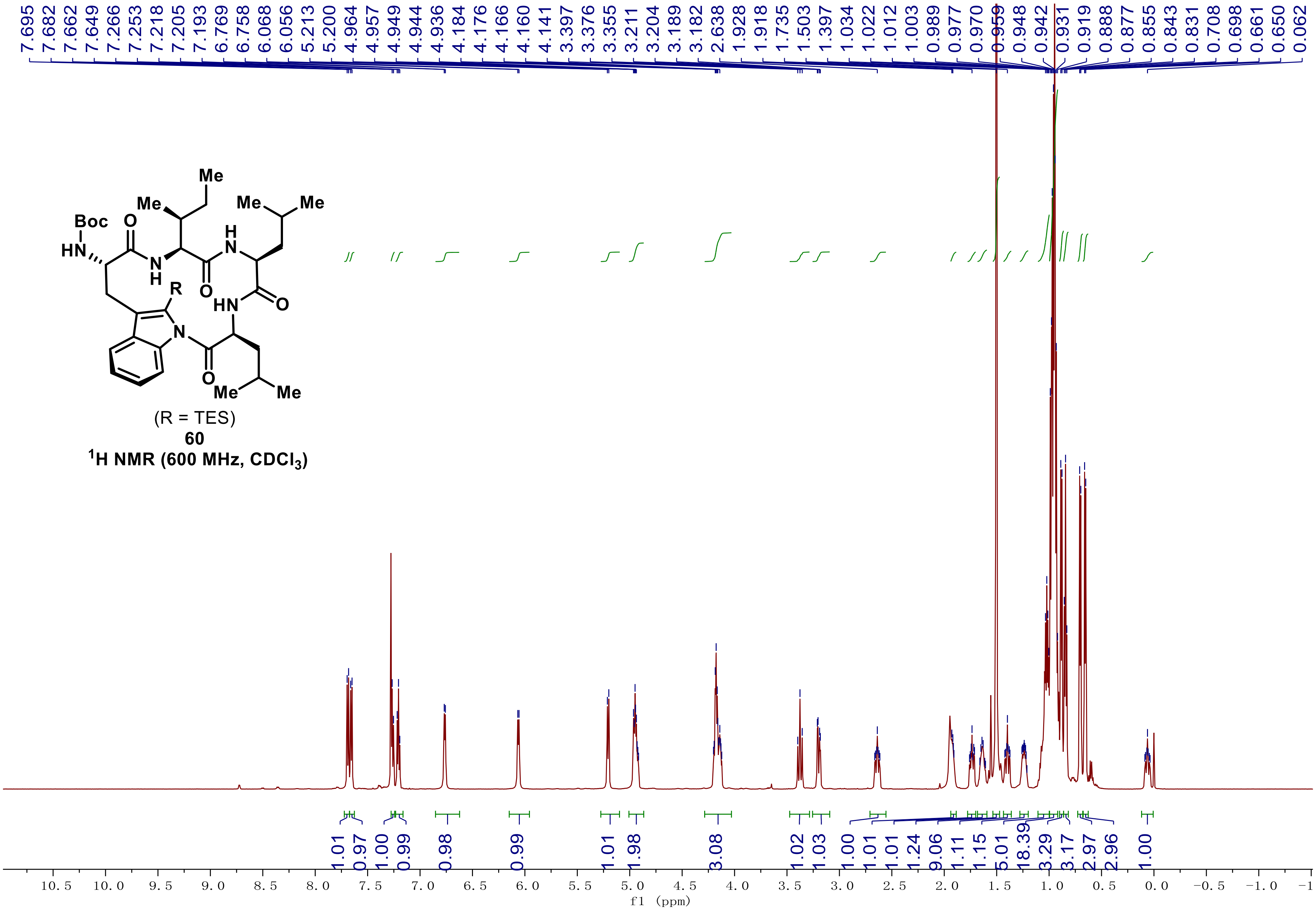

(R = TES)

60

^{1}H NMR (600 MHz, $CDCl_3$)

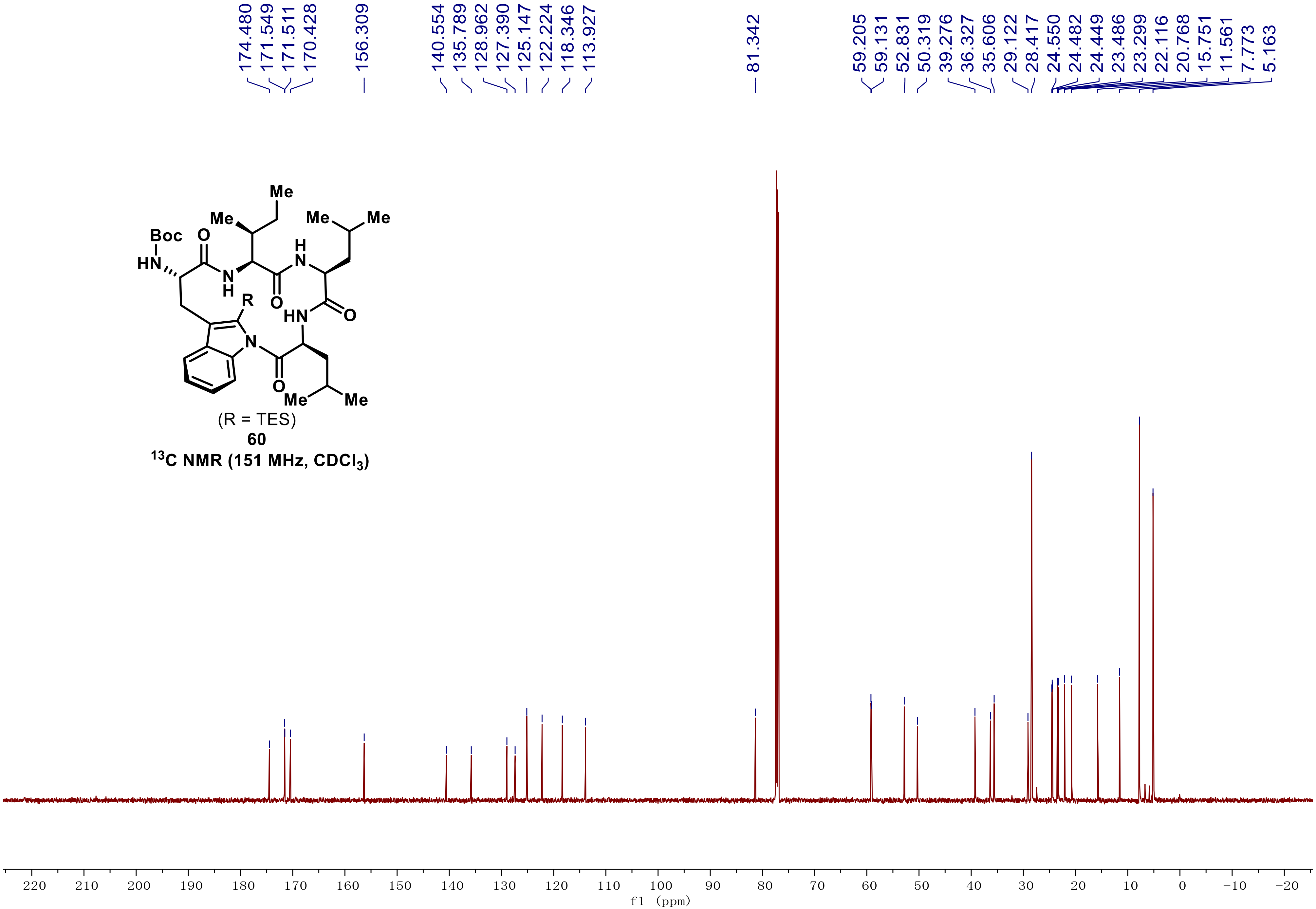
174.480
171.549
171.511
170.428
156.309
140.554
135.789
128.962
127.390
125.147
122.224
118.346
113.927
81.342
59.205
59.131
52.831
50.319
39.276
36.327
35.606
29.122
28.417
24.550
24.482
24.449
23.486
23.299
22.116
20.768
15.751
11.561
7.773
5.163
Boc
HN
O
N
H
Me
Me
H
N
Me
Me
R
O
HN
O
N
O
Me
Me
(R = TES)
60
^{13}C NMR (151 MHz, CDCl$_3$)
220 210 200 190 180 170 160 150 140 130 120 110 100 90 80 70 60 50 40 30 20 10 0 −10 −20
f1 (ppm)

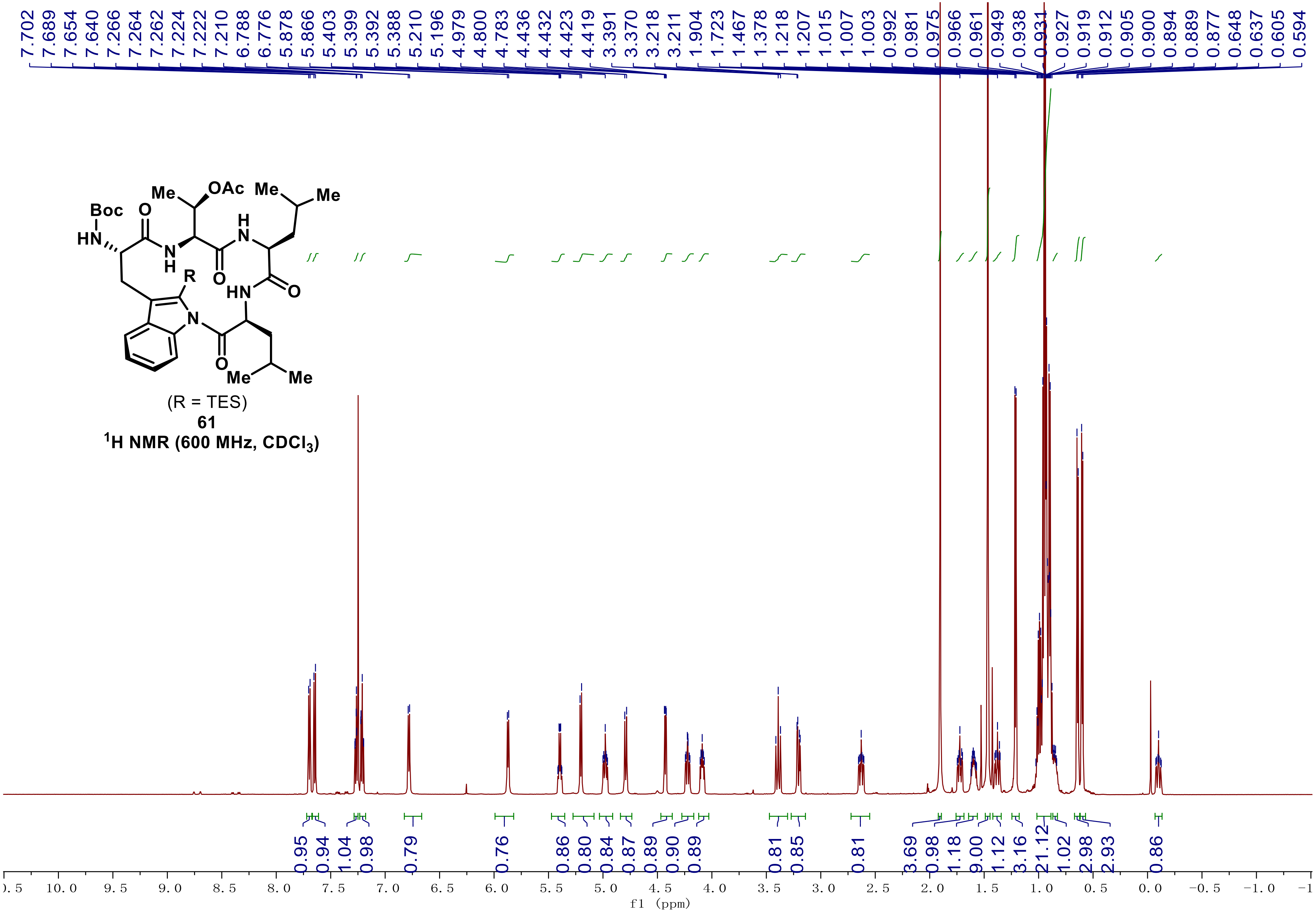

(R = TES)

61

^{1}H NMR (600 MHz, $CDCl_3$)

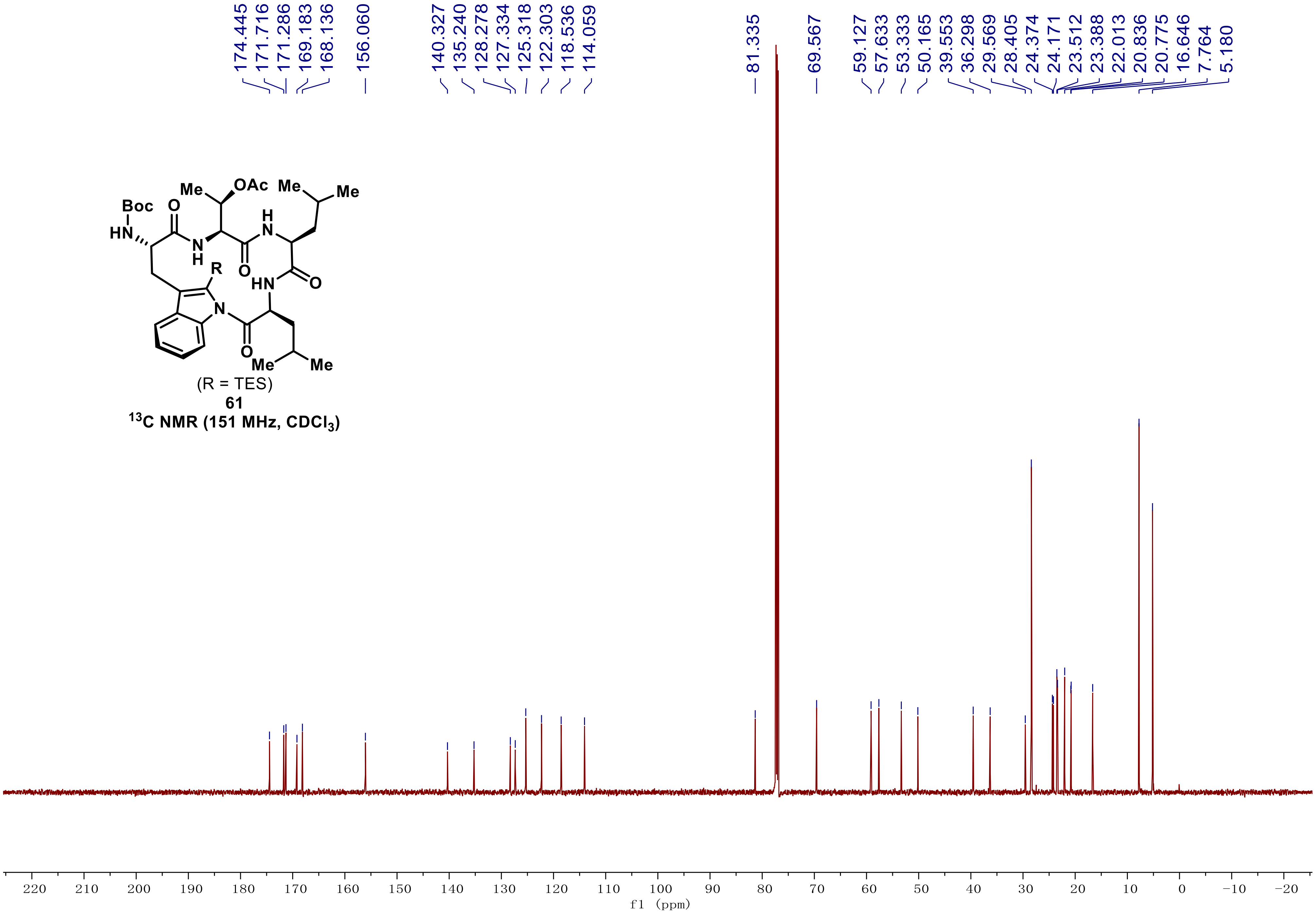

(R = TES)

61

^{13}C NMR (151 MHz, $CDCl_3$)

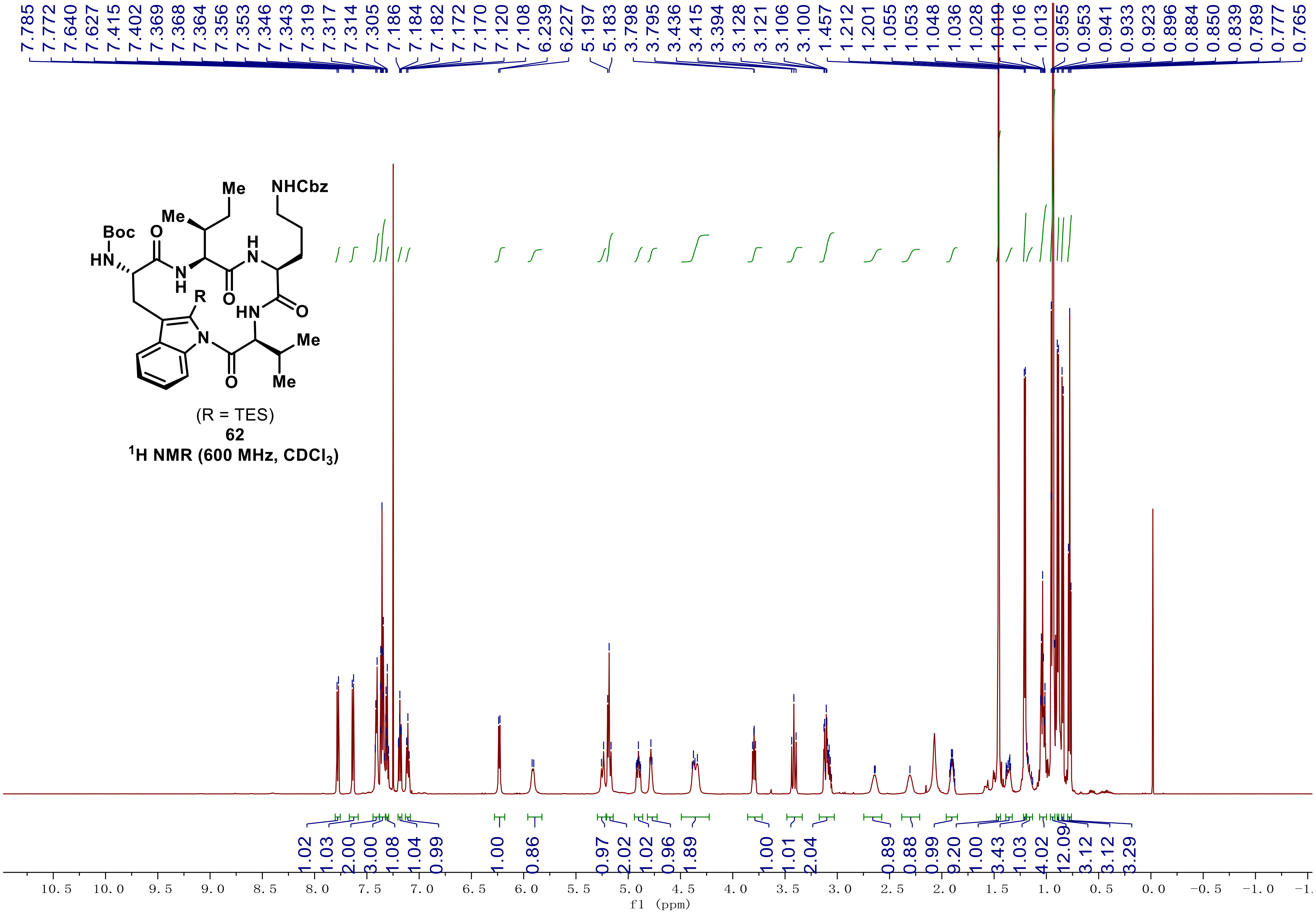
7.785
7.772
7.640
7.627
7.415
7.402
7.369
7.368
7.364
7.356
7.353
7.346
7.343
7.319
7.317
7.314
7.305
7.186
7.184
7.182
7.172
7.170
7.120
7.108
6.239
6.227
5.197
5.183
3.798
3.795
3.436
3.415
3.394
3.128
3.121
3.106
3.100
1.457
1.212
1.201
1.055
1.053
1.048
1.036
1.028
1.019
1.016
1.013
0.955
0.953
0.941
0.933
0.923
0.896
0.884
0.850
0.839
0.789
0.777
0.765
Me
NHCbz
Me
Boc
HN
H
N
N
H
R
O
HN
O
N
Me
O
Me
(R = TES)
62
¹H NMR (600 MHz, CDCl₃)
1.02
1.03
2.00
3.00
1.08
1.04
0.99
1.00
0.86
0.97
2.02
1.02
0.96
1.89
1.00
1.01
2.04
0.89
0.88
0.99
9.20
1.00
3.43
1.03
4.02
12.09
3.12
3.12
3.29
f1 (ppm)

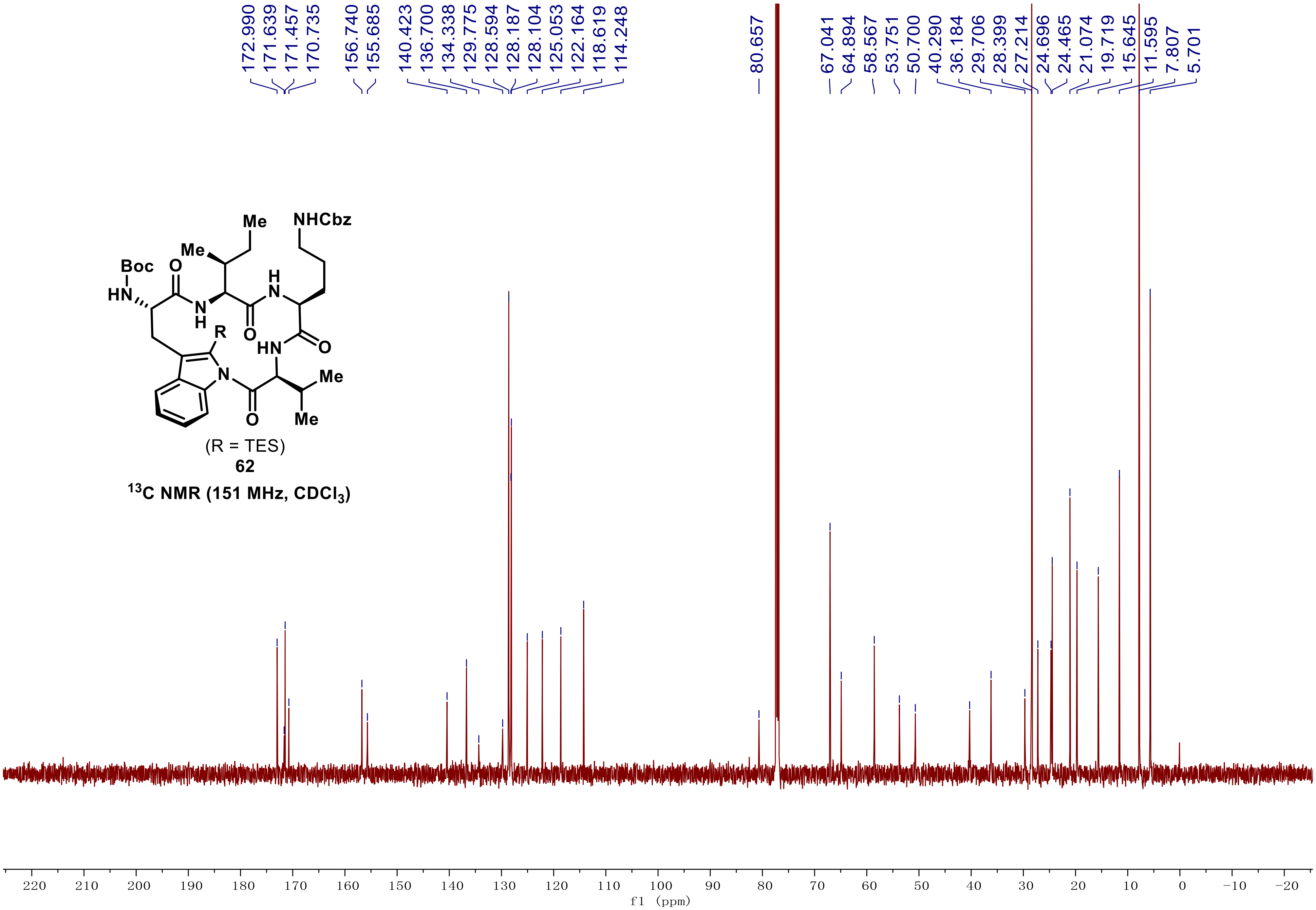
172.990
171.639
171.457
170.735
156.740
155.685
140.423
136.700
134.338
129.775
128.594
128.187
128.104
125.053
122.164
118.619
114.248
80.657
67.041
64.894
58.567
53.751
50.700
40.290
36.184
29.706
28.399
27.214
24.696
24.465
21.074
19.719
15.645
11.595
7.807
5.701
Me
NHCbz
Me
Boc
O
H
N
HN
N
H
R
O
HN
O
N
Me
O
Me
(R = TES)
62
^{13}C NMR (151 MHz, CDCl$_3$)
f1 (ppm)

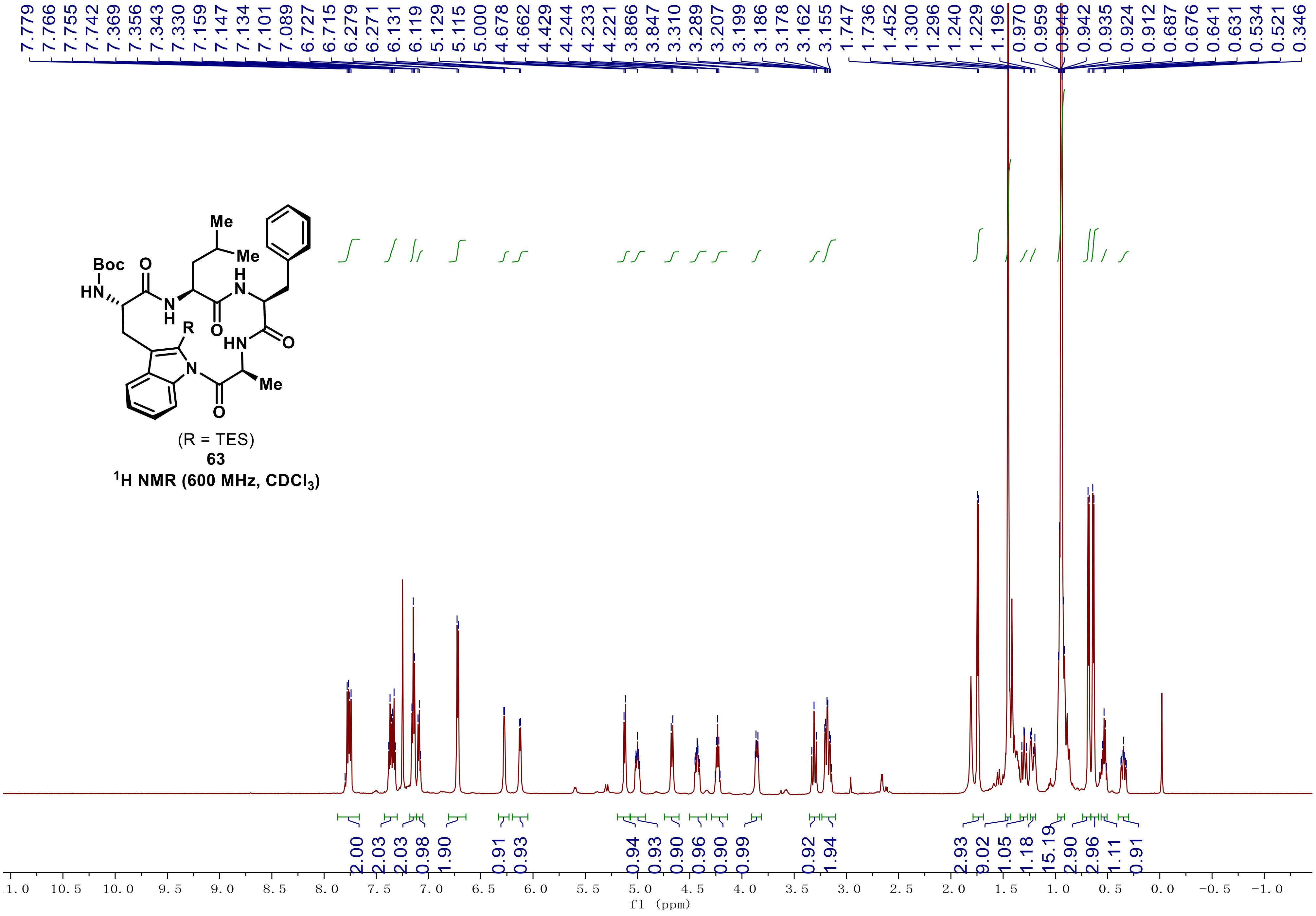

(R = TES)
63
^{1}H NMR (600 MHz, $CDCl_3$)

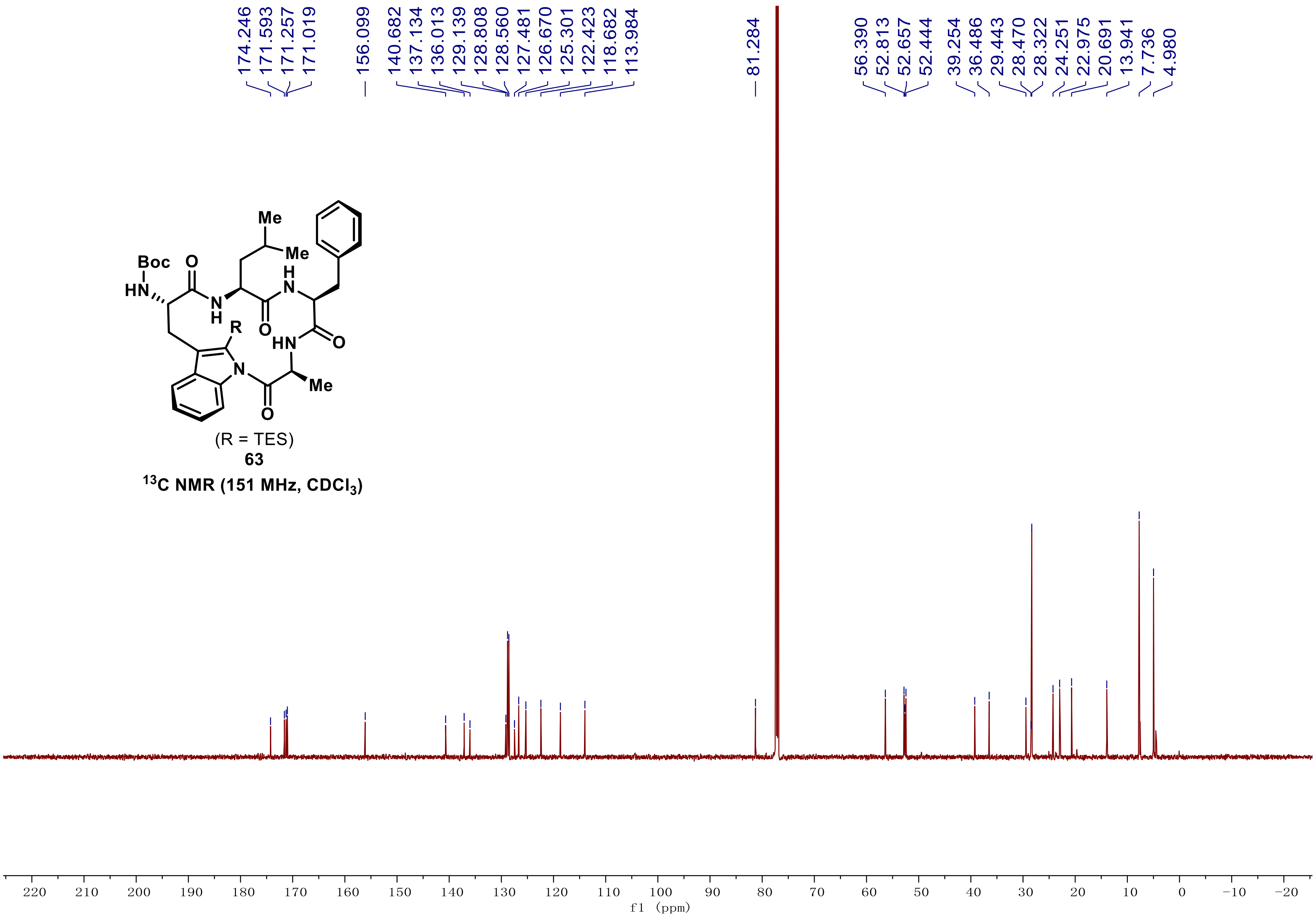

(R = TES)

63

^{13}C NMR (151 MHz, CDCl$_3$)

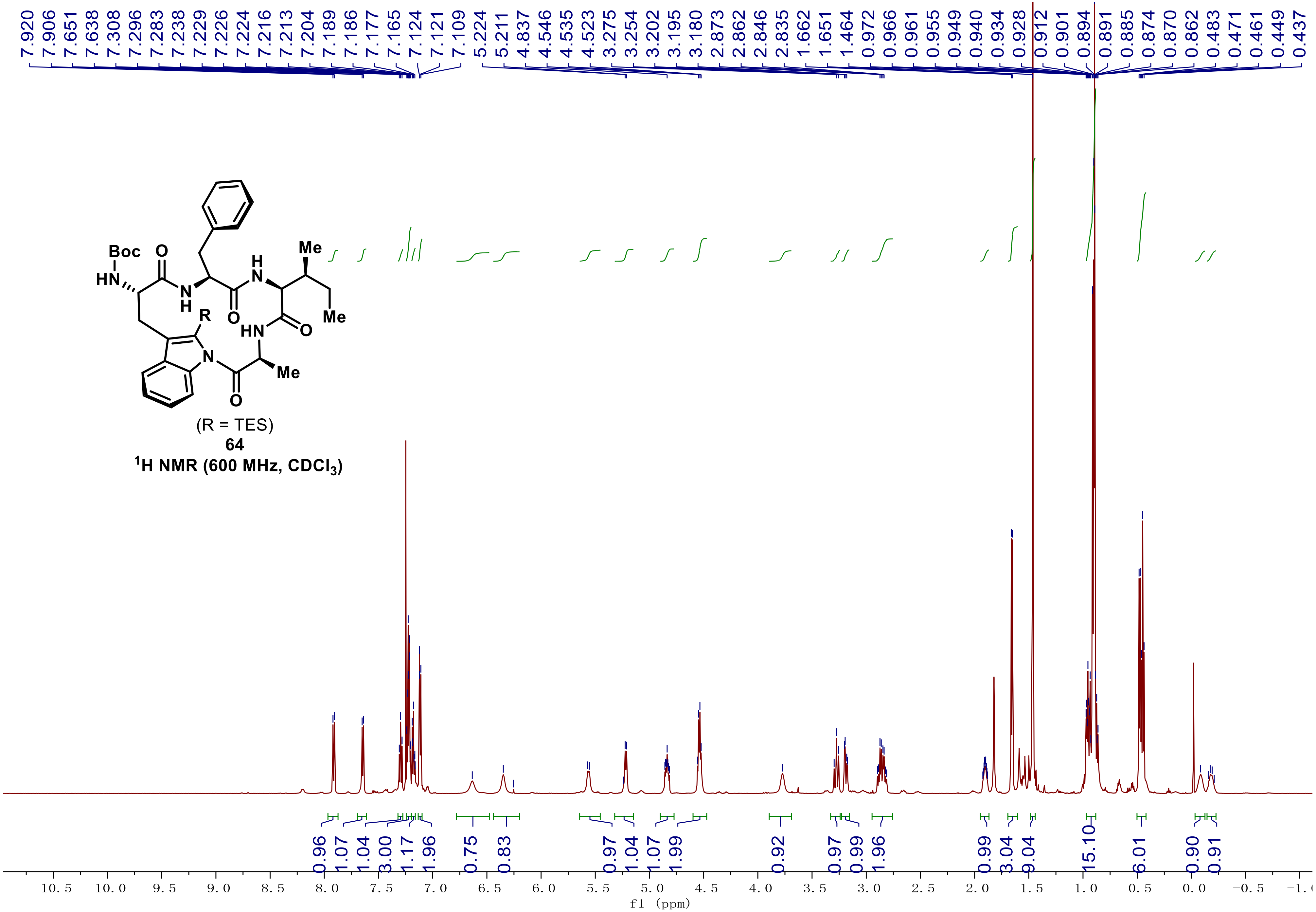
7.920 7.906 7.651 7.638 7.308 7.296 7.283 7.238 7.229 7.226 7.224 7.216 7.213 7.204 7.189 7.186 7.177 7.165 7.124 7.121 7.109 5.224 5.211 4.837 4.546 4.535 4.523 3.275 3.254 3.202 3.195 3.180 2.873 2.862 2.846 2.835 1.662 1.651 1.464 0.972 0.966 0.961 0.955 0.949 0.940 0.934 0.928 0.912 0.901 0.894 0.891 0.885 0.874 0.870 0.862 0.483 0.471 0.461 0.449 0.437
Boc
HN
O
N
H
H
N
Me
Me
O
R
HN
O
N
Me
O
(R = TES)
64
^{1}H NMR (600 MHz, CDCl$_3$)
0.96 1.07 1.04 3.00 1.17 1.96 0.75 0.83 0.97 1.04 1.07 1.99 0.92 0.97 0.99 1.96 0.99 3.04 9.04 15.10 6.01 0.90 0.91
10.5 10.0 9.5 9.0 8.5 8.0 7.5 7.0 6.5 6.0 5.5 5.0 4.5 4.0 3.5 3.0 2.5 2.0 1.5 1.0 0.5 0.0 −0.5 −1.0
f1 (ppm)

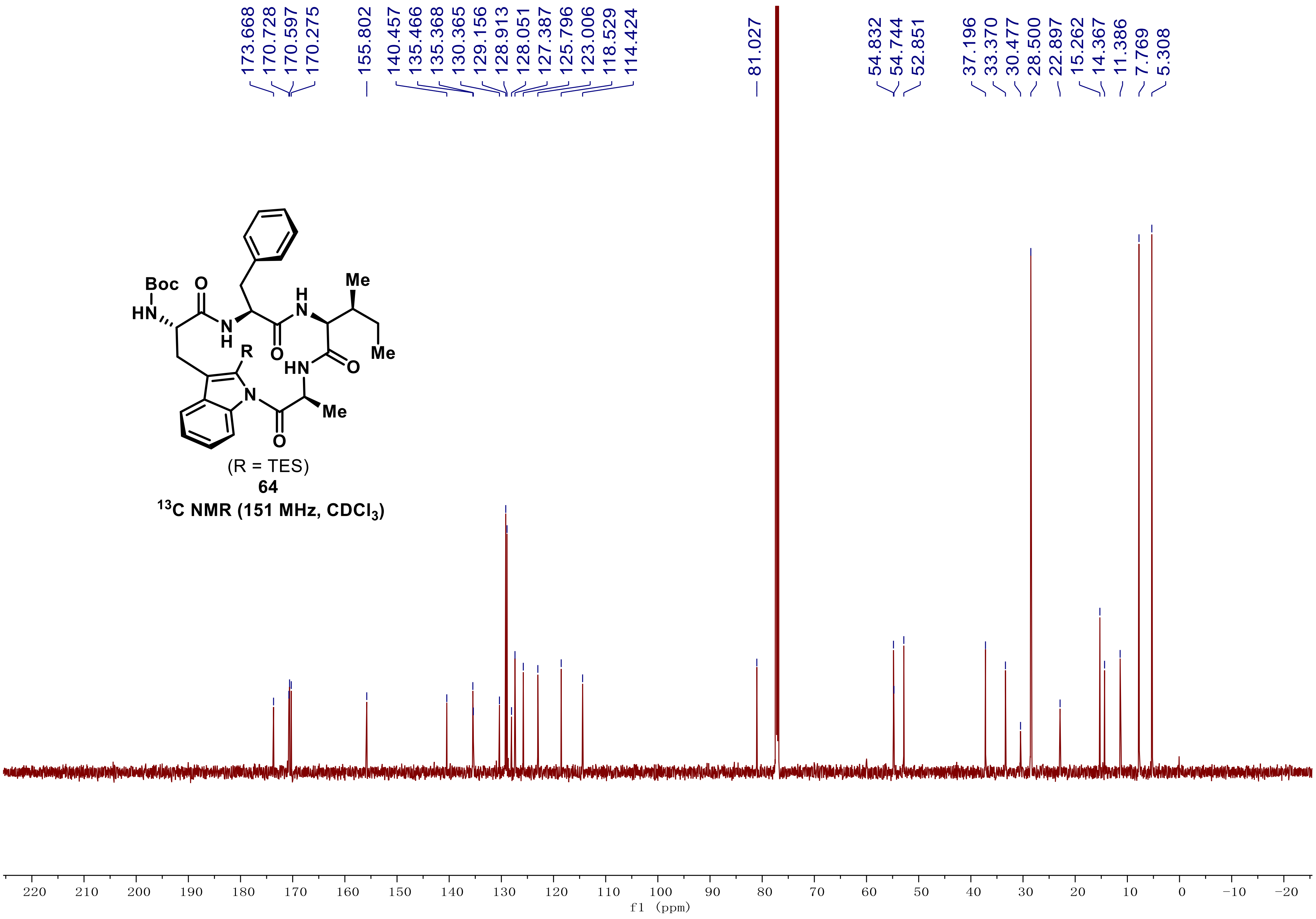
173.668
170.728
170.597
170.275
155.802
140.457
135.466
135.368
130.365
129.156
128.913
128.051
127.387
125.796
123.006
118.529
114.424
81.027
54.832
54.744
52.851
37.196
33.370
30.477
28.500
22.897
15.262
14.367
11.386
7.769
5.308
Boc
HN
O
N
H
R
O
H
N
Me
Me
HN
O
N
O
Me
(R = TES)
64
^{13}C NMR (151 MHz, $CDCl_3$)
220 210 200 190 180 170 160 150 140 130 120 110 100 90 80 70 60 50 40 30 20 10 0 −10 −20
f1 (ppm)

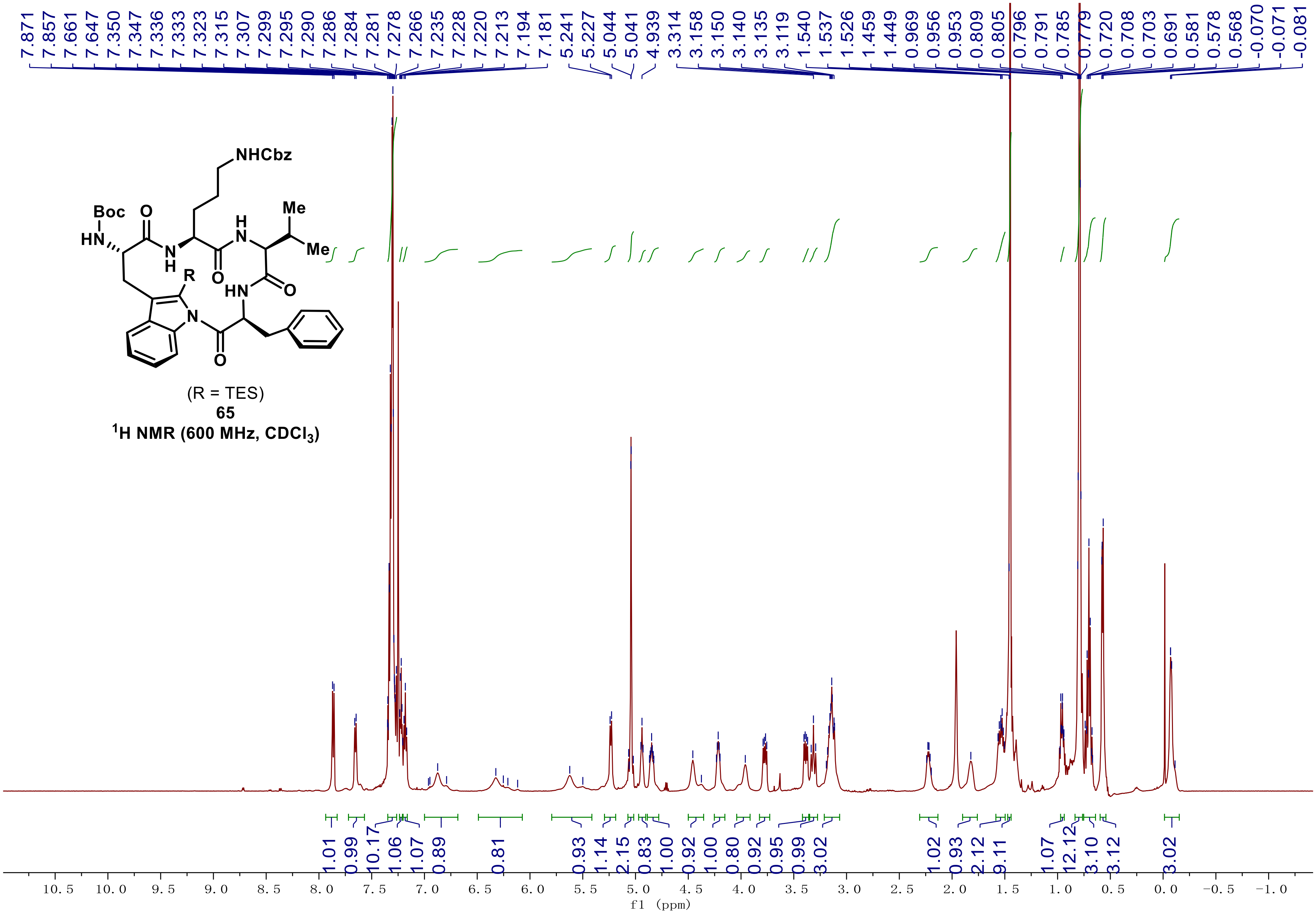

(R = TES)
65
^{1}H NMR (600 MHz, $CDCl_3$)

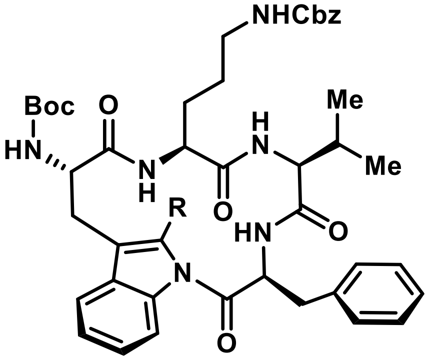

(R = TES)

65

^{13}C NMR (151 MHz, $CDCl_3$)

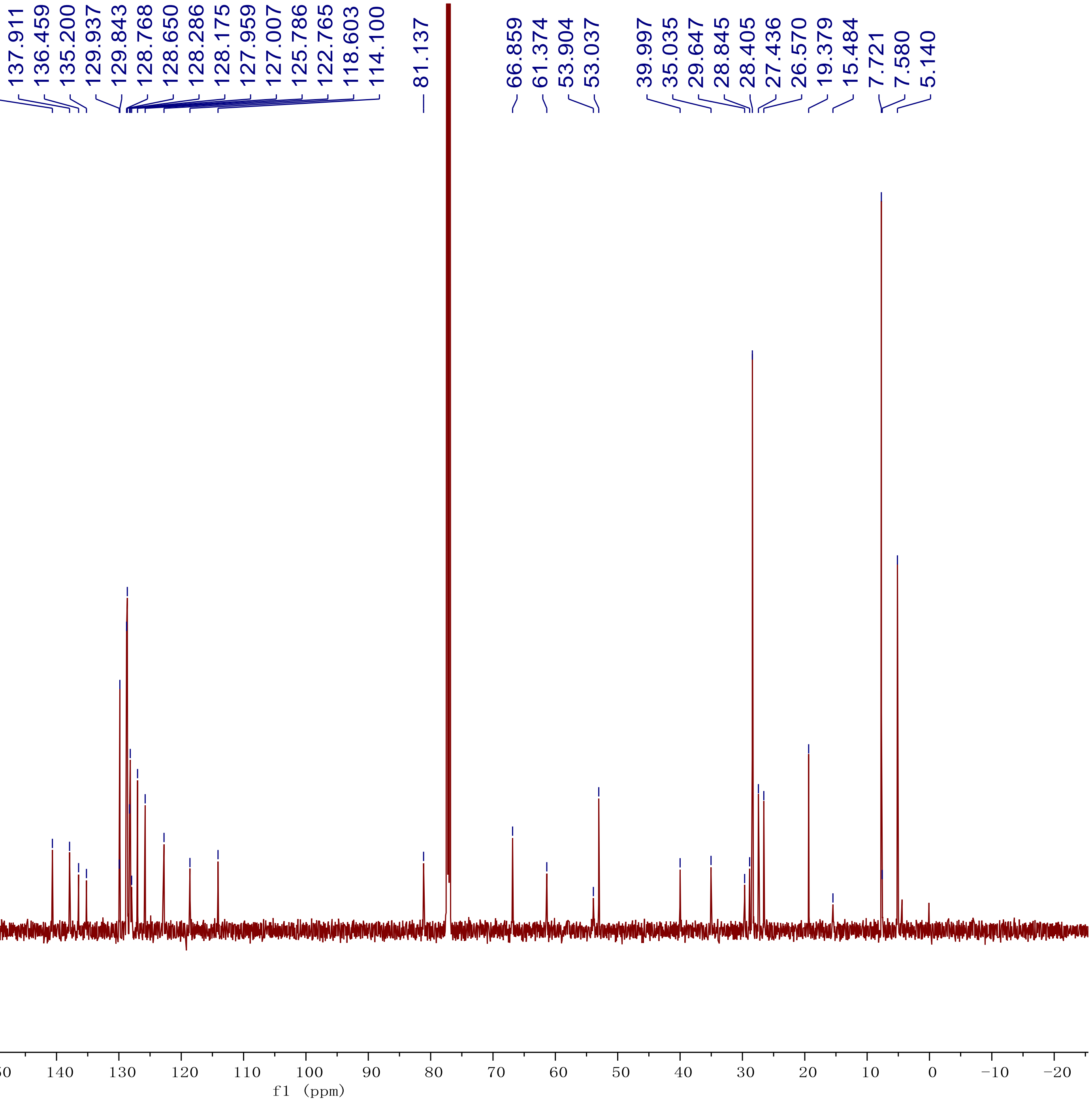

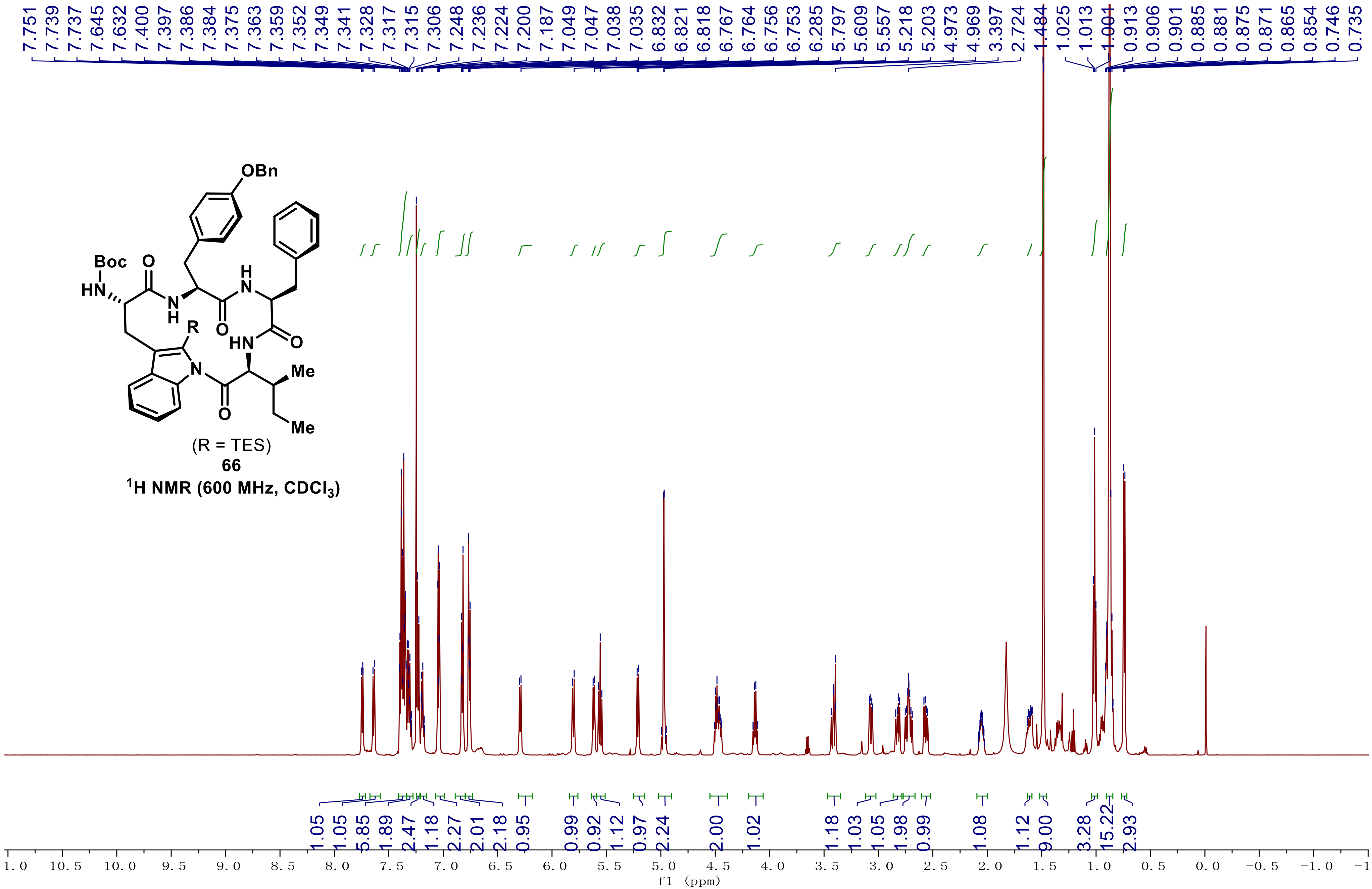
OBn
Boc
HN
O
N
H
H
N
R
O
HN
O
N
Me
O
Me
(R = TES)
66
¹H NMR (600 MHz, CDCl₃)
f1 (ppm)

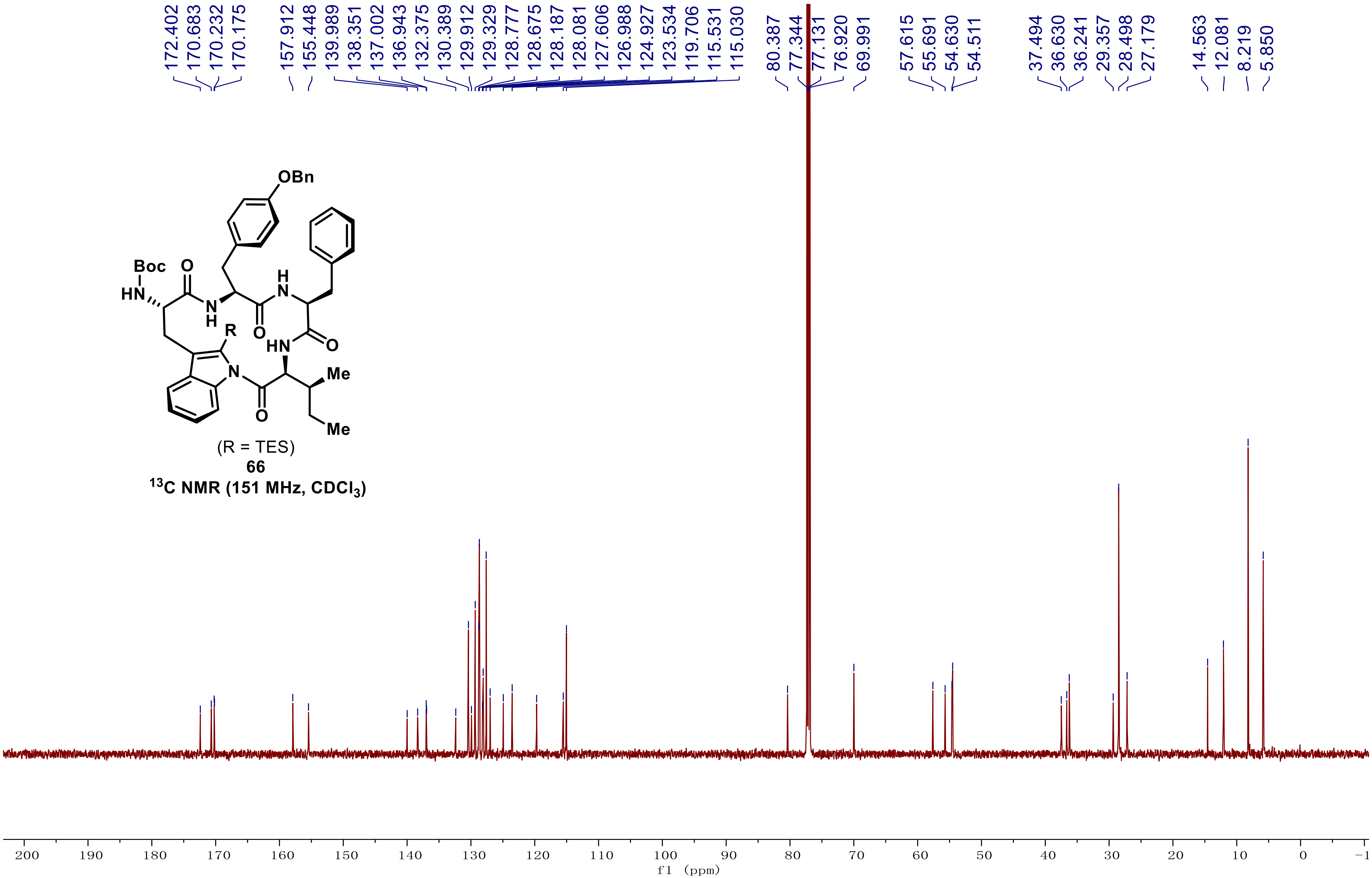

(R = TES)

66

^{13}C NMR (151 MHz, $CDCl_3$)

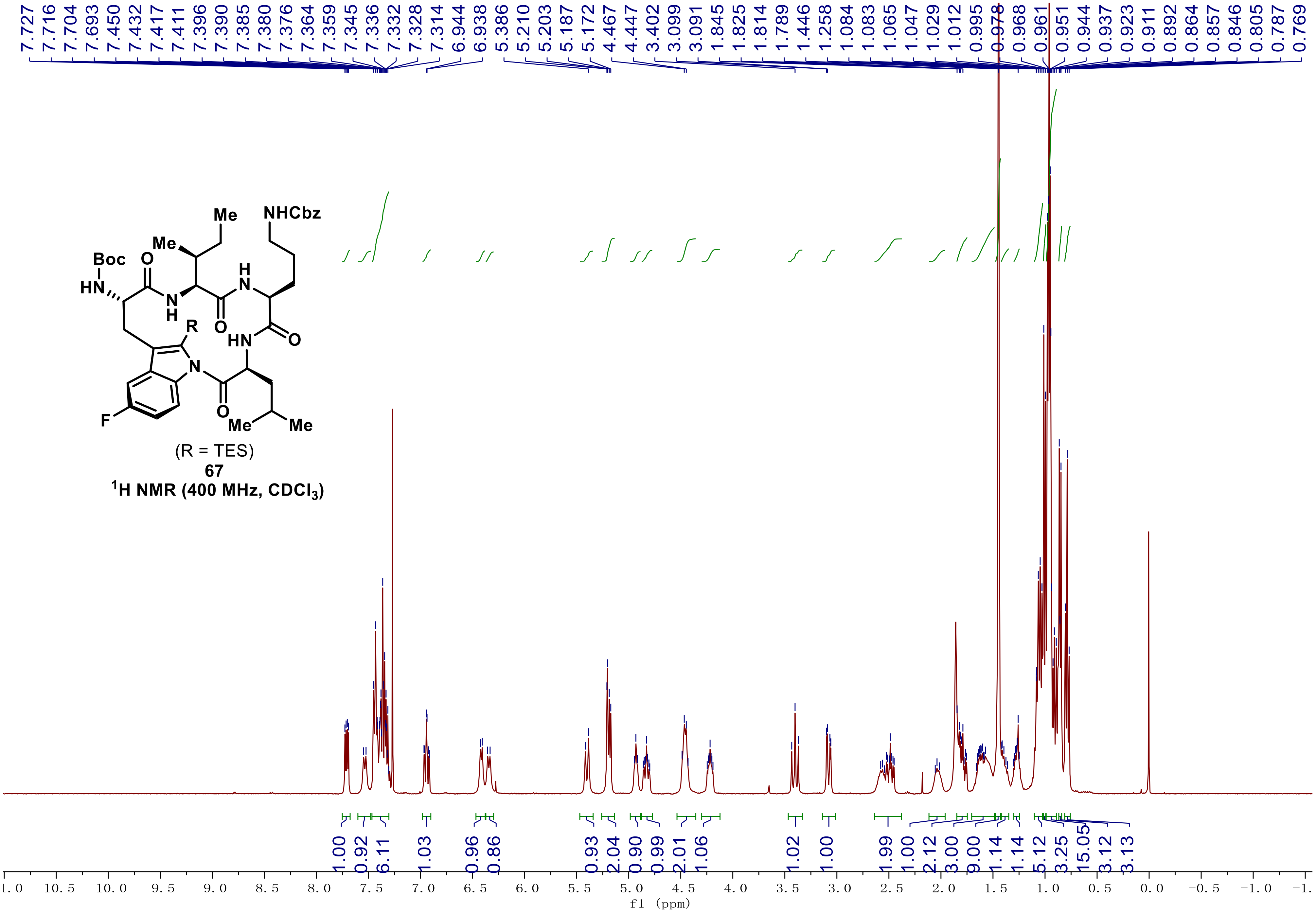
7.727 7.716 7.704 7.693 7.450 7.432 7.417 7.411 7.396 7.390 7.385 7.380 7.376 7.364 7.359 7.345 7.336 7.332 7.328 7.314 6.944 6.938 5.386 5.210 5.203 5.187 5.172 4.467 4.447 3.402 3.099 3.091 1.845 1.825 1.814 1.789 1.446 1.258 1.084 1.083 1.065 1.047 1.029 1.012 0.995 0.978 0.968 0.961 0.951 0.944 0.937 0.923 0.911 0.892 0.864 0.857 0.846 0.805 0.787 0.769
Me
NHCbz
Me
Boc
O
HN
N
H
H
N
R
O
HN
O
N
F
O
Me
Me
(R = TES)
67
1H NMR (400 MHz, CDCl3)
1.00 0.92 6.11 1.03 0.96 0.86 0.93 2.04 0.90 0.99 2.01 1.06 1.02 1.00 1.99 1.00 2.12 3.00 9.00 1.14 1.14 5.12 3.25 15.05 3.12 3.13
10.5 10.0 9.5 9.0 8.5 8.0 7.5 7.0 6.5 6.0 5.5 5.0 4.5 4.0 3.5 3.0 2.5 2.0 1.5 1.0 0.5 0.0 −0.5 −1.0
f1 (ppm)

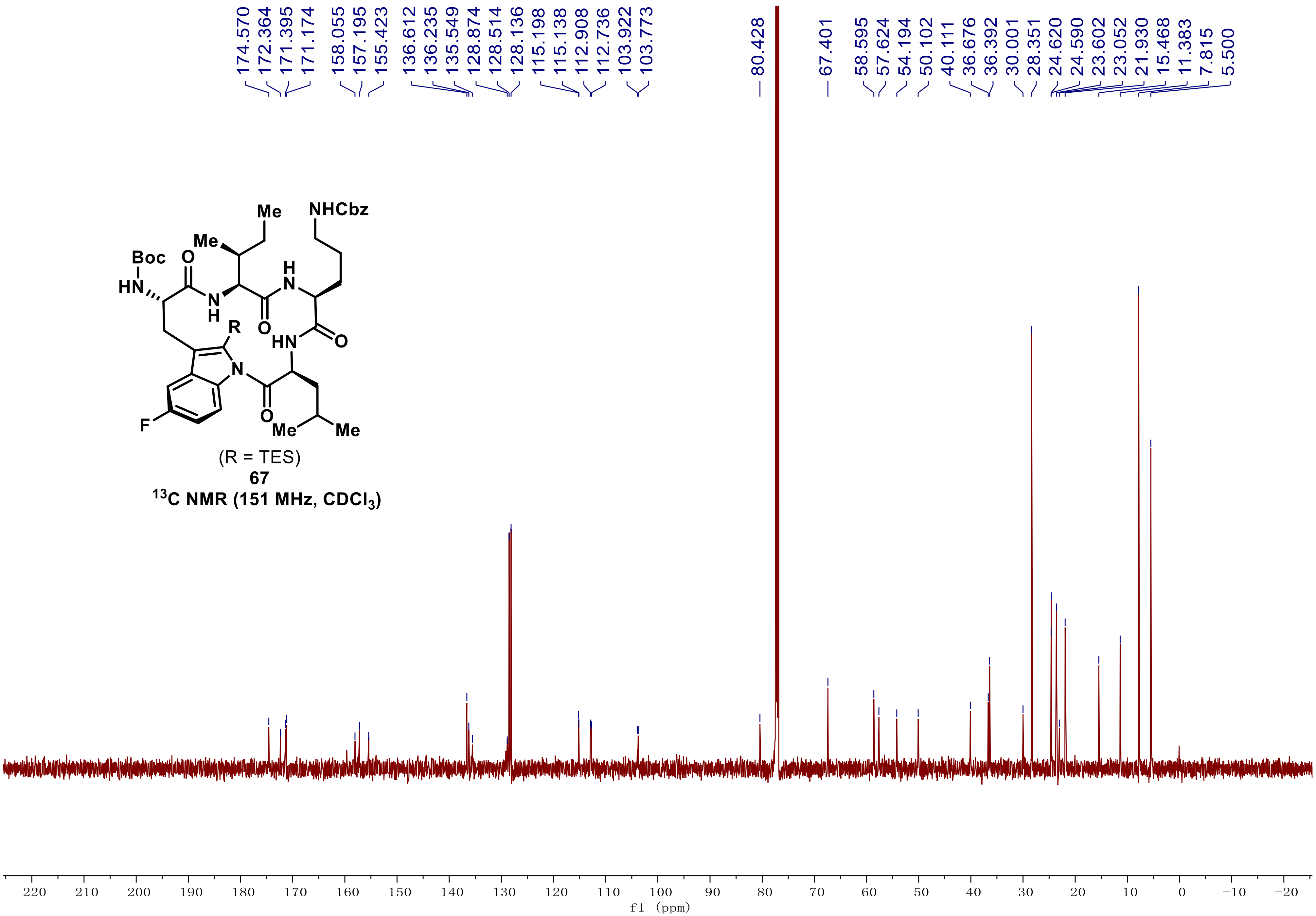

(R = TES)

67

^{13}C NMR (151 MHz, CDCl$_3$)

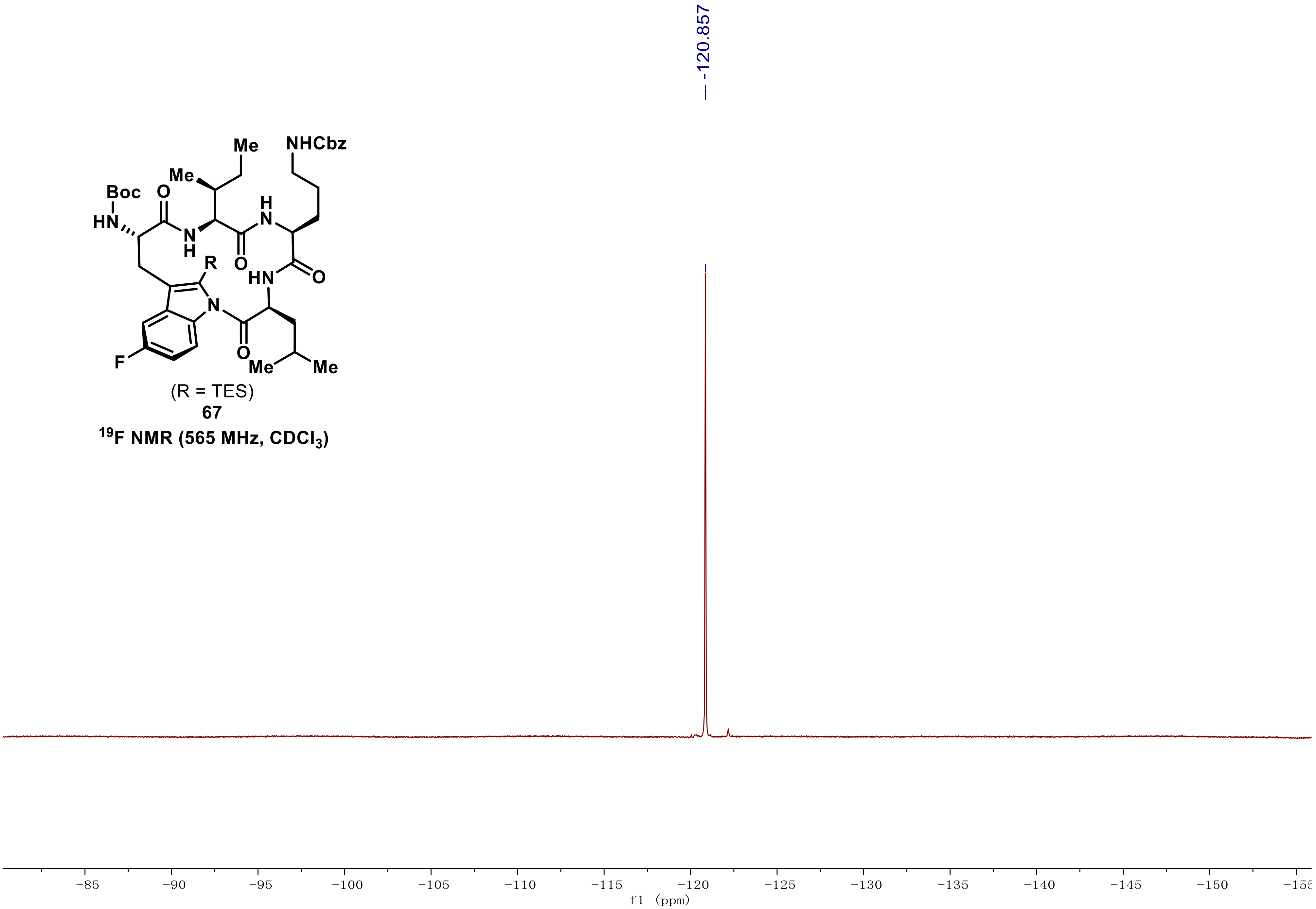

(R = TES)
67
^{19}F NMR (565 MHz, $CDCl_3$)

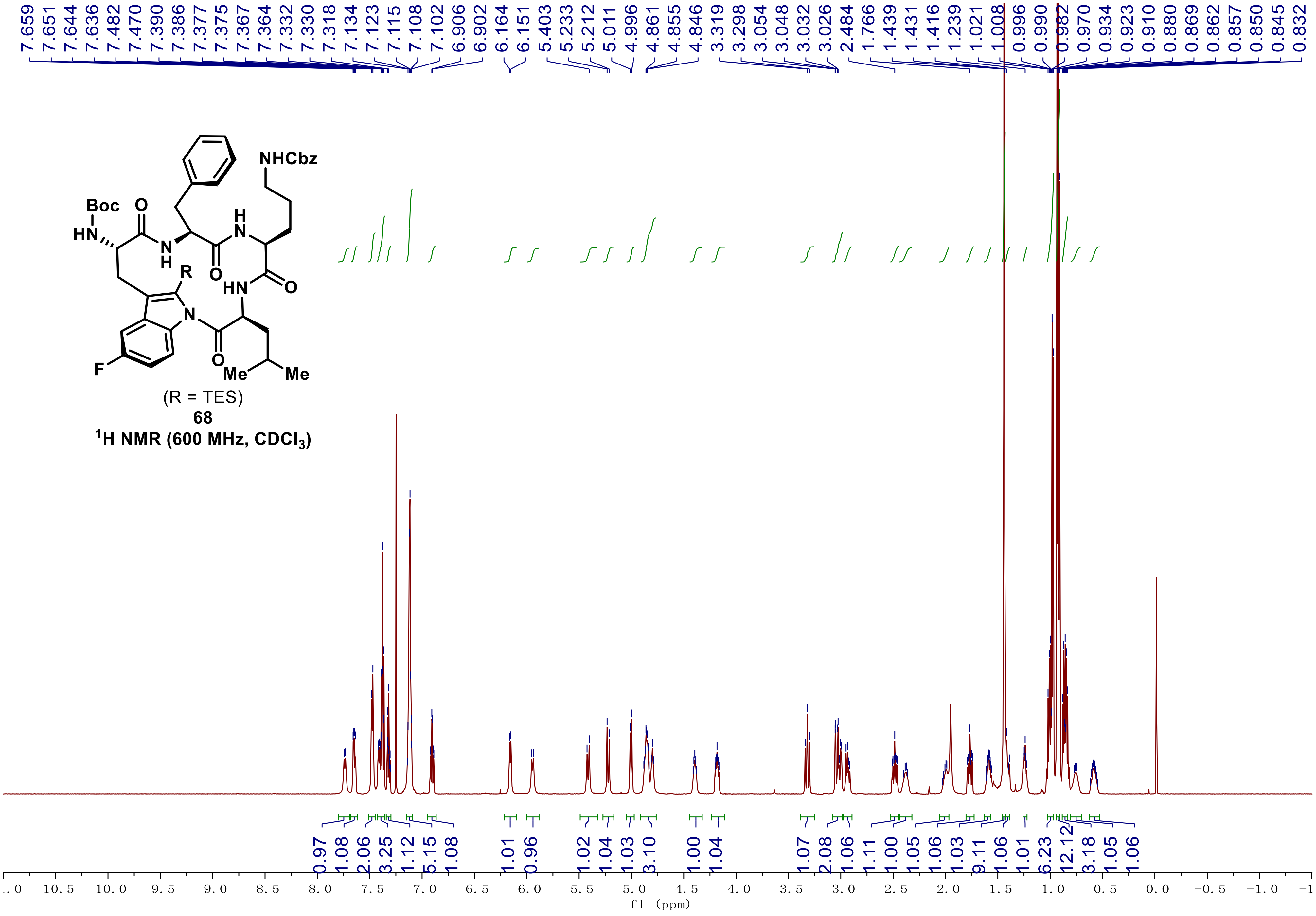
NHCbz
Boc
HN
O
N
H
R
F
O
Me
Me
(R = TES)
68
1H NMR (600 MHz, CDCl3)
f1 (ppm)

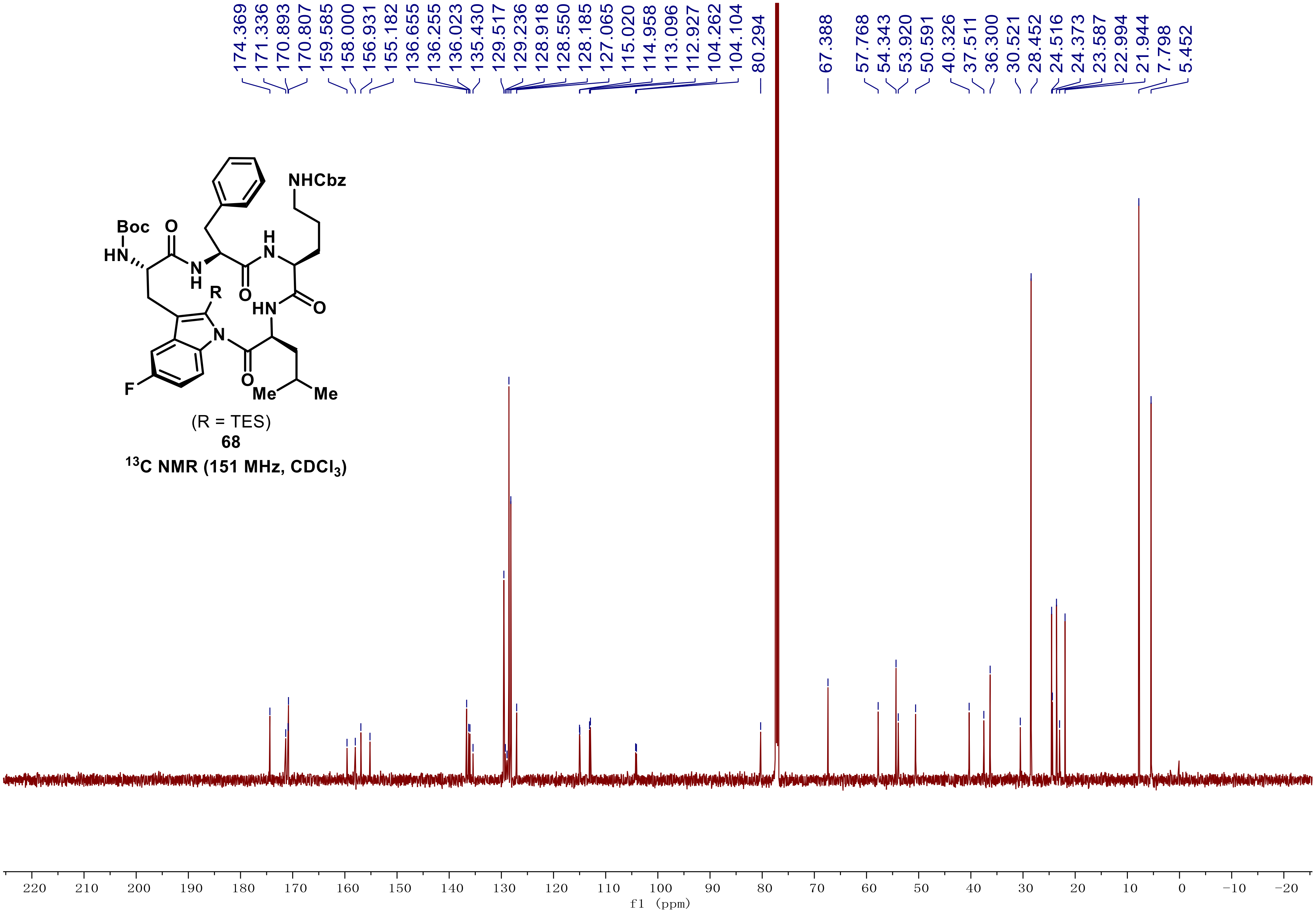

174.369
171.336
170.893
170.807
159.585
158.000
156.931
155.182
136.655
136.255
136.023
135.430
129.517
129.236
128.918
128.550
128.185
127.065
115.020
114.958
113.096
112.927
104.262
104.104
80.294
67.388
57.768
54.343
53.920
50.591
40.326
37.511
36.300
30.521
28.452
24.516
24.373
23.587
22.994
21.944
7.798
5.452
NHCbz
Boc
HN
O
H
N
R
F
Me
Me
(R = TES)
68
^{13}C NMR (151 MHz, CDCl$_3$)
220 210 200 190 180 170 160 150 140 130 120 110 100 90 80 70 60 50 40 30 20 10 0 −10 −20
f1 (ppm)

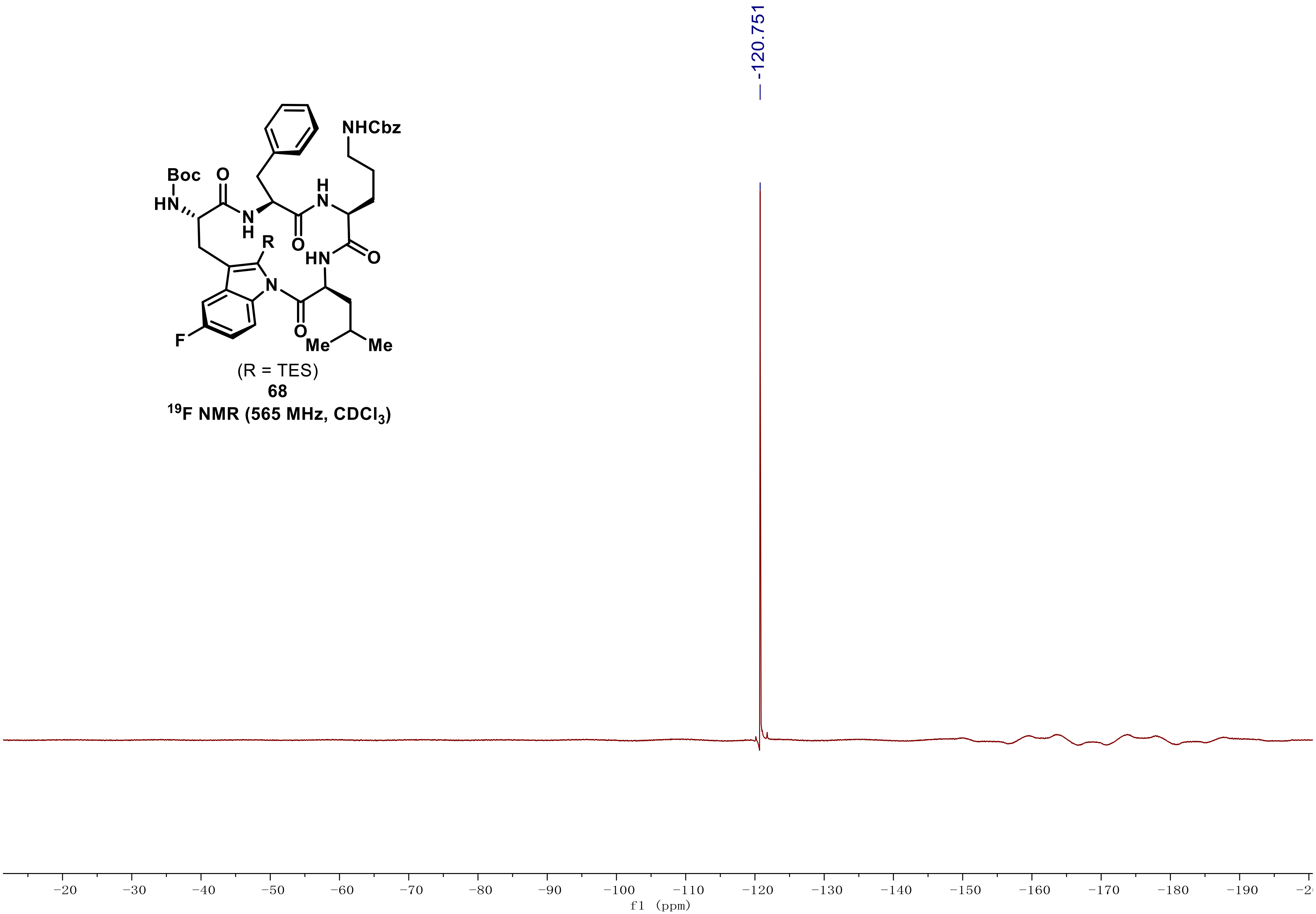

-120.751
NHCbz
Boc
O
HN
N
H
R
O
HN
N
O
F
O
Me
Me
(R = TES)
68
19F NMR (565 MHz, CDCl3)
-20
-30
-40
-50
-60
-70
-80
-90
-100
-110
-120
-130
-140
-150
-160
-170
-180
-190
f1 (ppm)

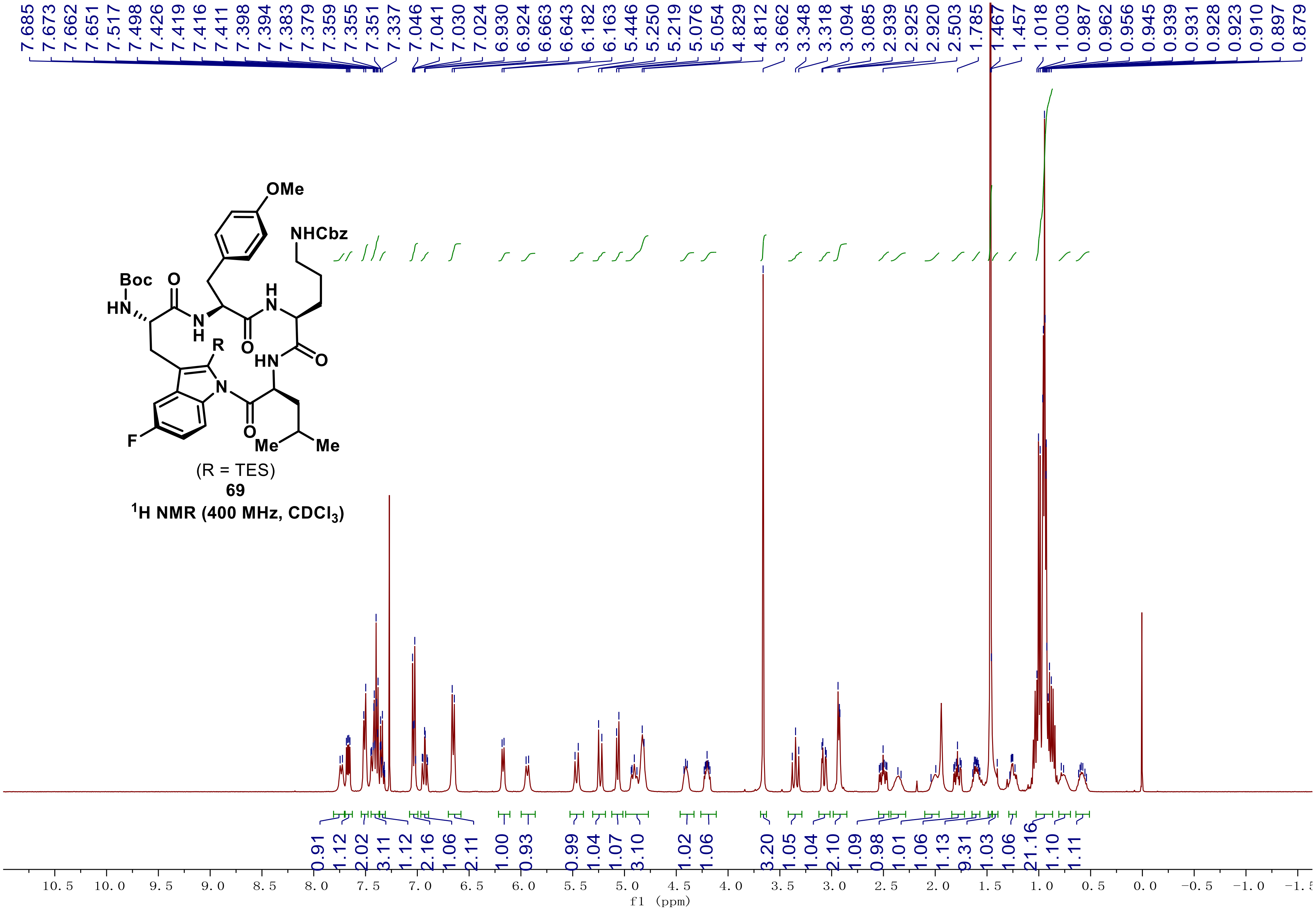

7.685
7.673
7.662
7.651
7.517
7.498
7.426
7.419
7.416
7.411
7.398
7.394
7.383
7.379
7.359
7.355
7.351
7.337
7.046
7.041
7.030
7.024
6.930
6.924
6.663
6.643
6.182
6.163
5.446
5.250
5.219
5.076
5.054
4.829
4.812
3.662
3.348
3.318
3.094
3.085
2.939
2.925
2.920
2.503
1.785
1.467
1.457
1.018
1.003
0.987
0.962
0.956
0.945
0.939
0.931
0.928
0.923
0.910
0.897
0.879
OMe
NHCbz
Boc
O
HN
N
H
H
N
R
O
HN
O
N
F
O
Me
Me
(R = TES)
69
¹H NMR (400 MHz, CDCl₃)
0.91
1.12
2.02
3.11
1.12
2.16
1.06
2.11
1.00
0.93
0.99
1.04
1.07
3.10
1.02
1.06
3.20
1.05
1.04
2.10
1.09
0.98
1.01
1.06
1.13
9.31
1.03
1.06
21.16
1.10
1.11
10.5
10.0
9.5
9.0
8.5
8.0
7.5
7.0
6.5
6.0
5.5
5.0
4.5
4.0
3.5
3.0
2.5
2.0
1.5
1.0
0.5
0.0
−0.5
−1.0
f1 (ppm)

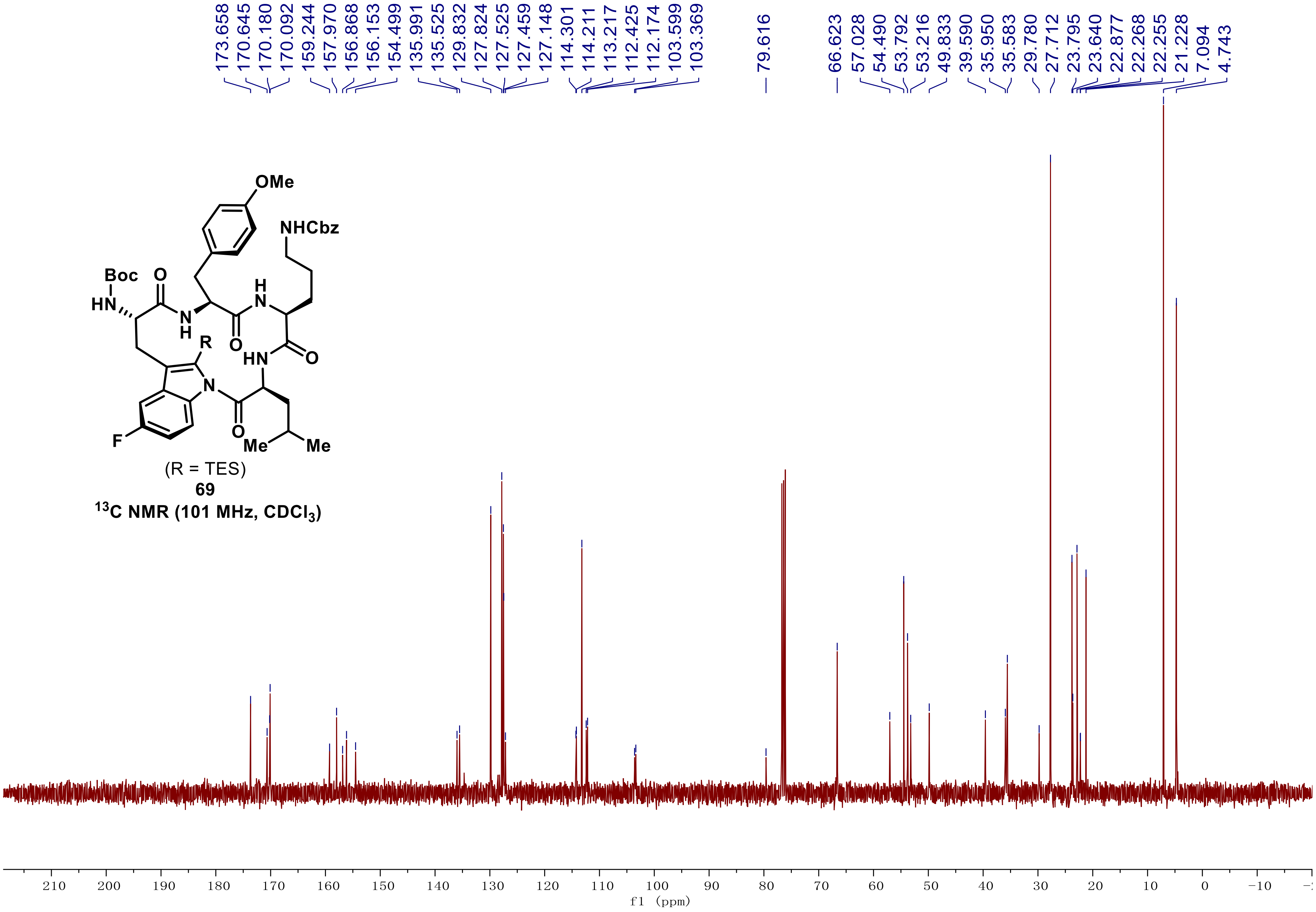

(R = TES)

69

^{13}C NMR (101 MHz, $CDCl_3$)

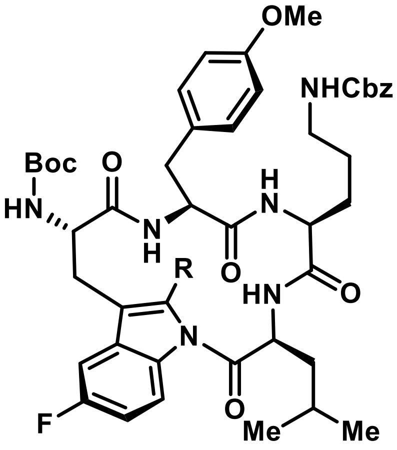

(R = TES)
69
^{19}F NMR (565 MHz, $CDCl_3$)

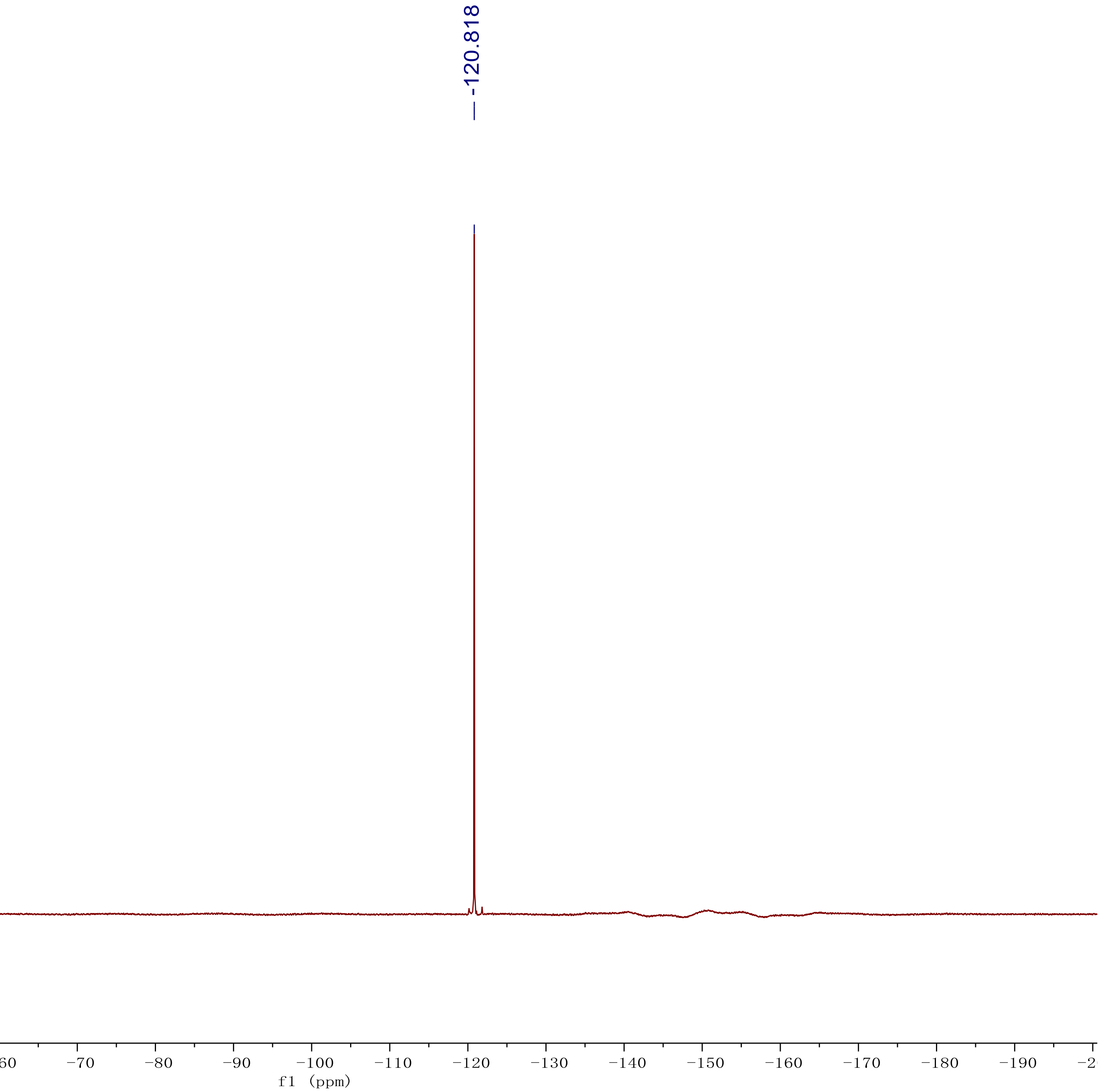

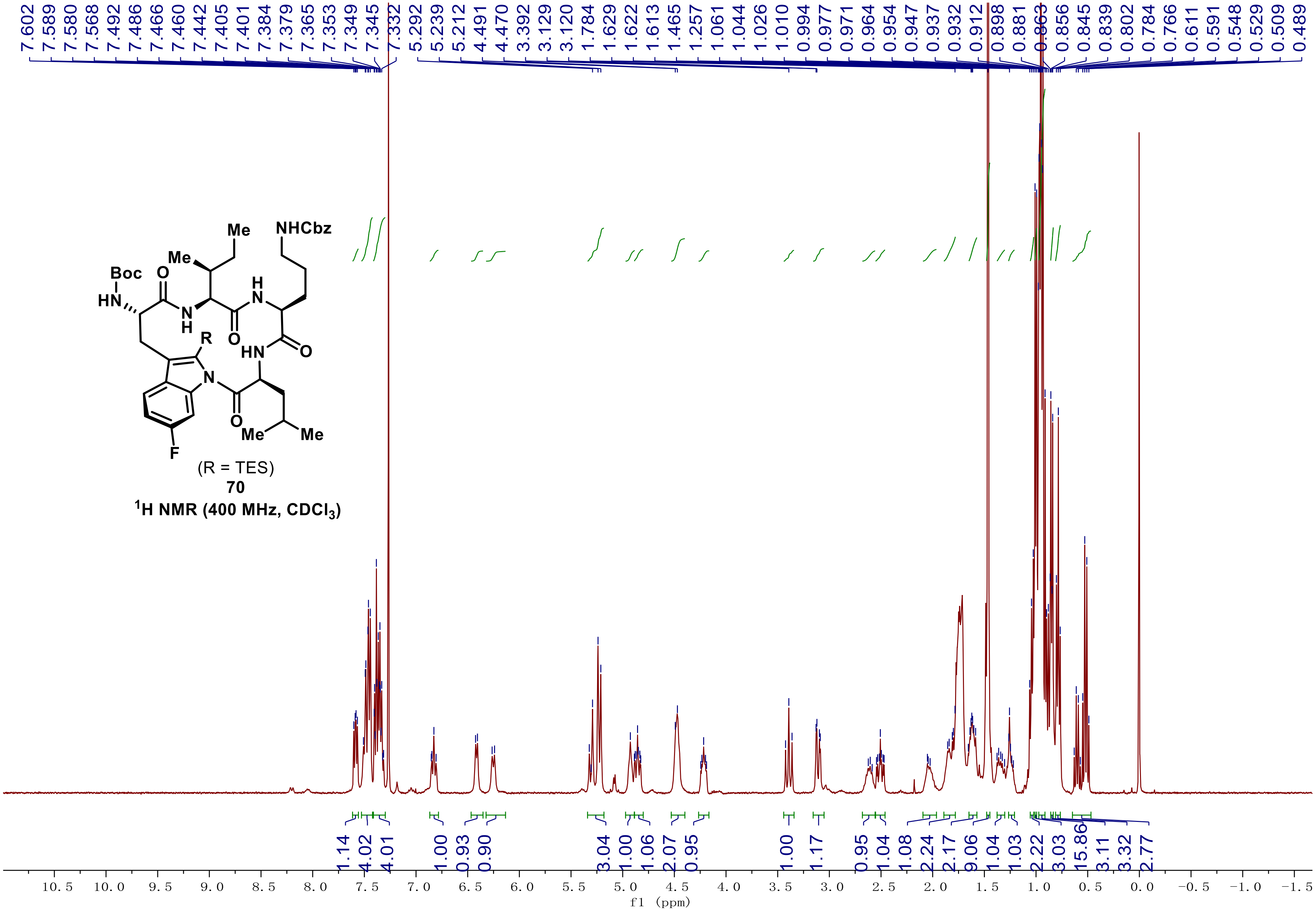
7.602
7.589
7.580
7.568
7.492
7.486
7.466
7.460
7.442
7.405
7.401
7.384
7.379
7.365
7.353
7.349
7.345
7.332
5.292
5.239
5.212
4.491
4.470
3.392
3.129
3.120
1.784
1.629
1.622
1.613
1.465
1.257
1.061
1.044
1.026
1.010
0.994
0.977
0.971
0.964
0.954
0.947
0.937
0.932
0.912
0.898
0.881
0.863
0.856
0.845
0.839
0.802
0.784
0.766
0.611
0.591
0.548
0.529
0.509
0.489
Me
NHCbz
Me
Boc
O
H
N
HN
N
H
R
O
HN
O
N
O
Me
Me
F
(R = TES)
70
^{1}H NMR (400 MHz, $CDCl_3$)
1.14
4.02
4.01
1.00
0.93
0.90
3.04
1.00
1.06
2.07
0.95
1.00
1.17
0.95
1.04
1.08
2.24
2.17
9.06
1.04
1.03
2.22
3.03
15.86
3.11
3.32
2.77
10.5
10.0
9.5
9.0
8.5
8.0
7.5
7.0
6.5
6.0
5.5
5.0
4.5
4.0
3.5
3.0
2.5
2.0
1.5
1.0
0.5
0.0
-0.5
-1.0
-1.5
f1 (ppm)

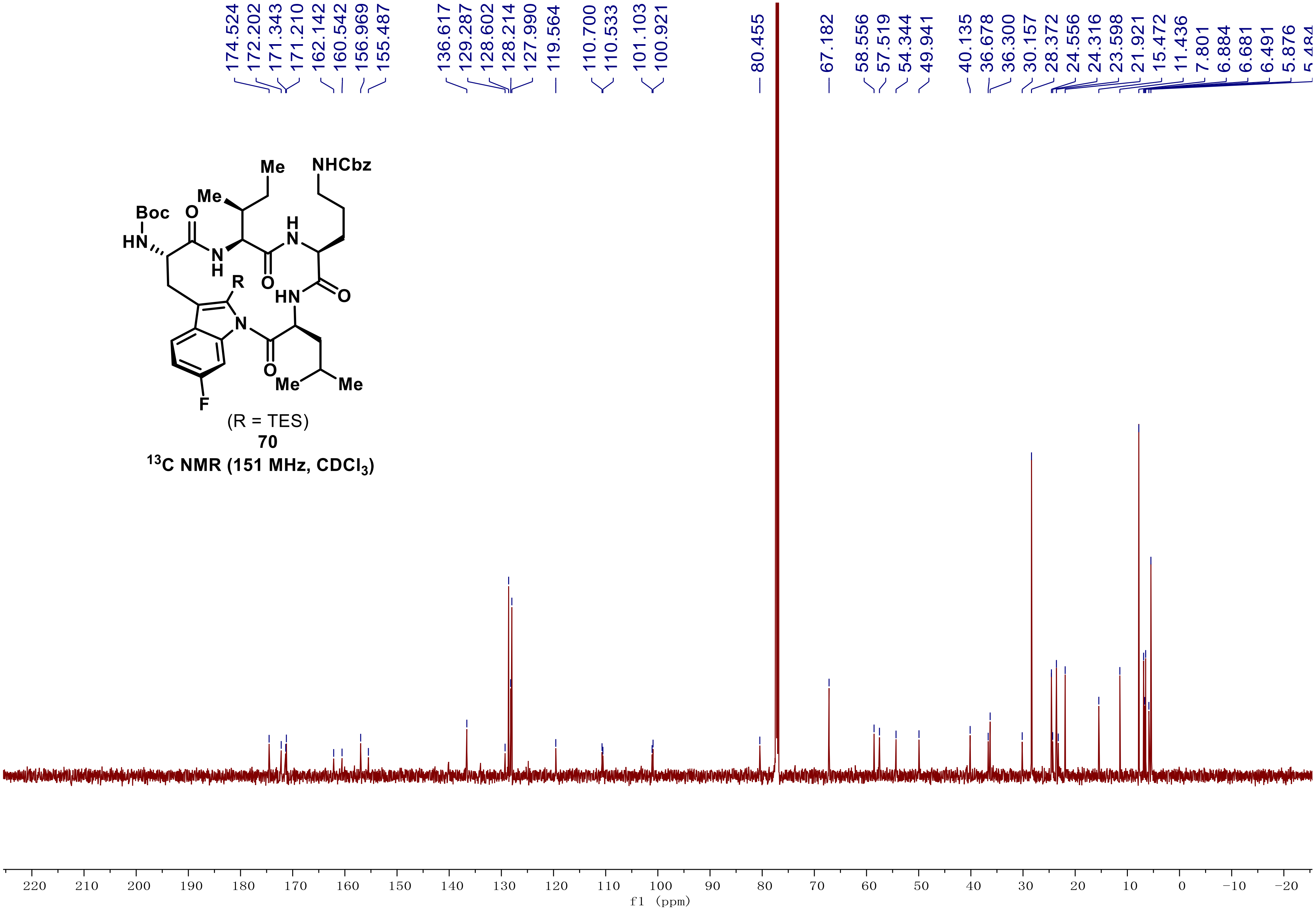
174.524
172.202
171.343
171.210
162.142
160.542
156.969
155.487
136.617
129.287
128.602
128.214
127.990
119.564
110.700
110.533
101.103
100.921
80.455
67.182
58.556
57.519
54.344
49.941
40.135
36.678
36.300
30.157
28.372
24.556
24.316
23.598
21.921
15.472
11.436
7.801
6.884
6.681
6.491
5.876
Me
NHCbz
Me
Boc
O
H
HN
N
N
H
R
O
HN
O
N
O
Me
Me
F
(R = TES)
70
13C NMR (151 MHz, CDCl3)
220 210 200 190 180 170 160 150 140 130 120 110 100 90 80 70 60 50 40 30 20 10 0 −10 −20
f1 (ppm)

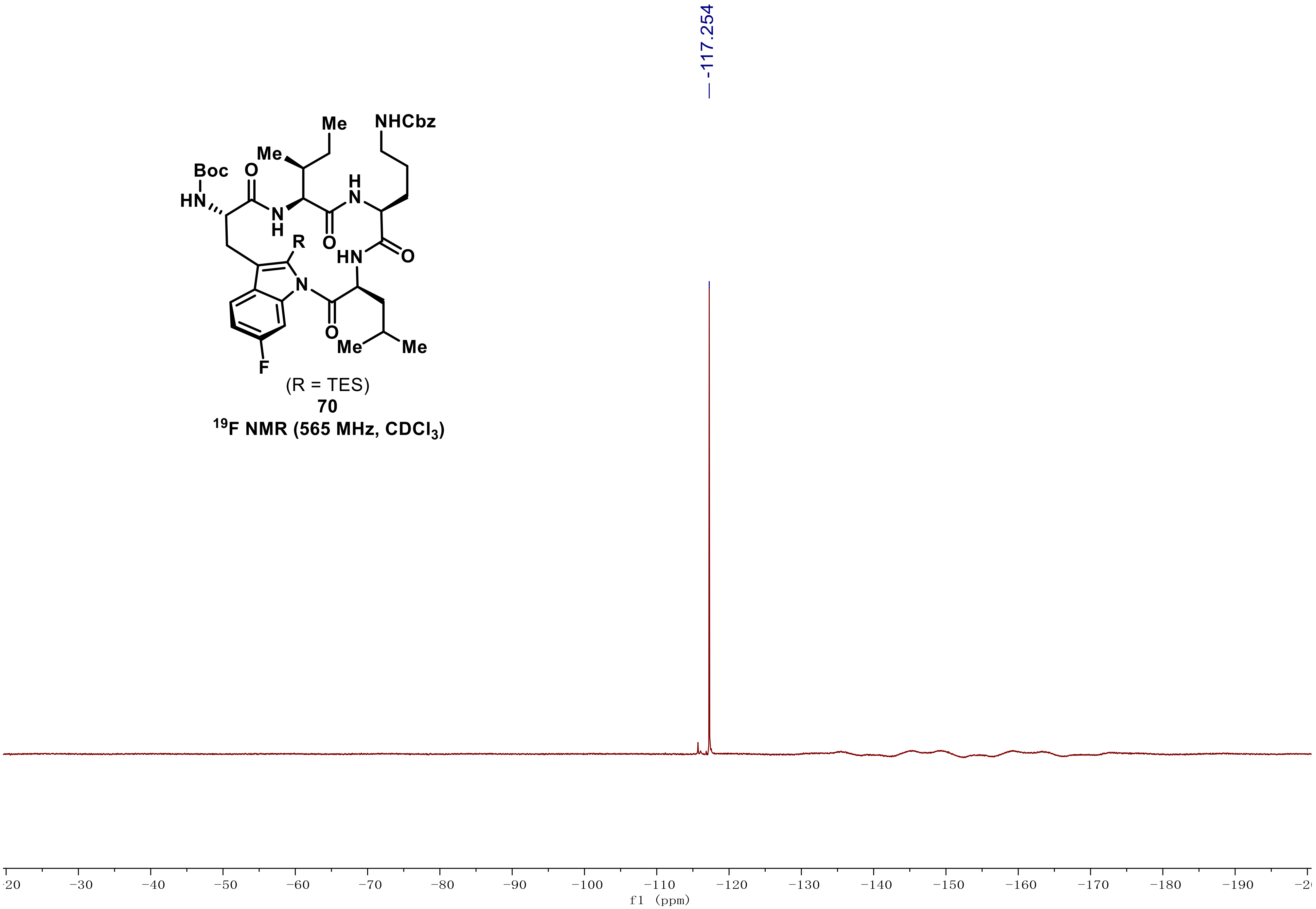

70

^{19}F NMR (565 MHz, $CDCl_3$)

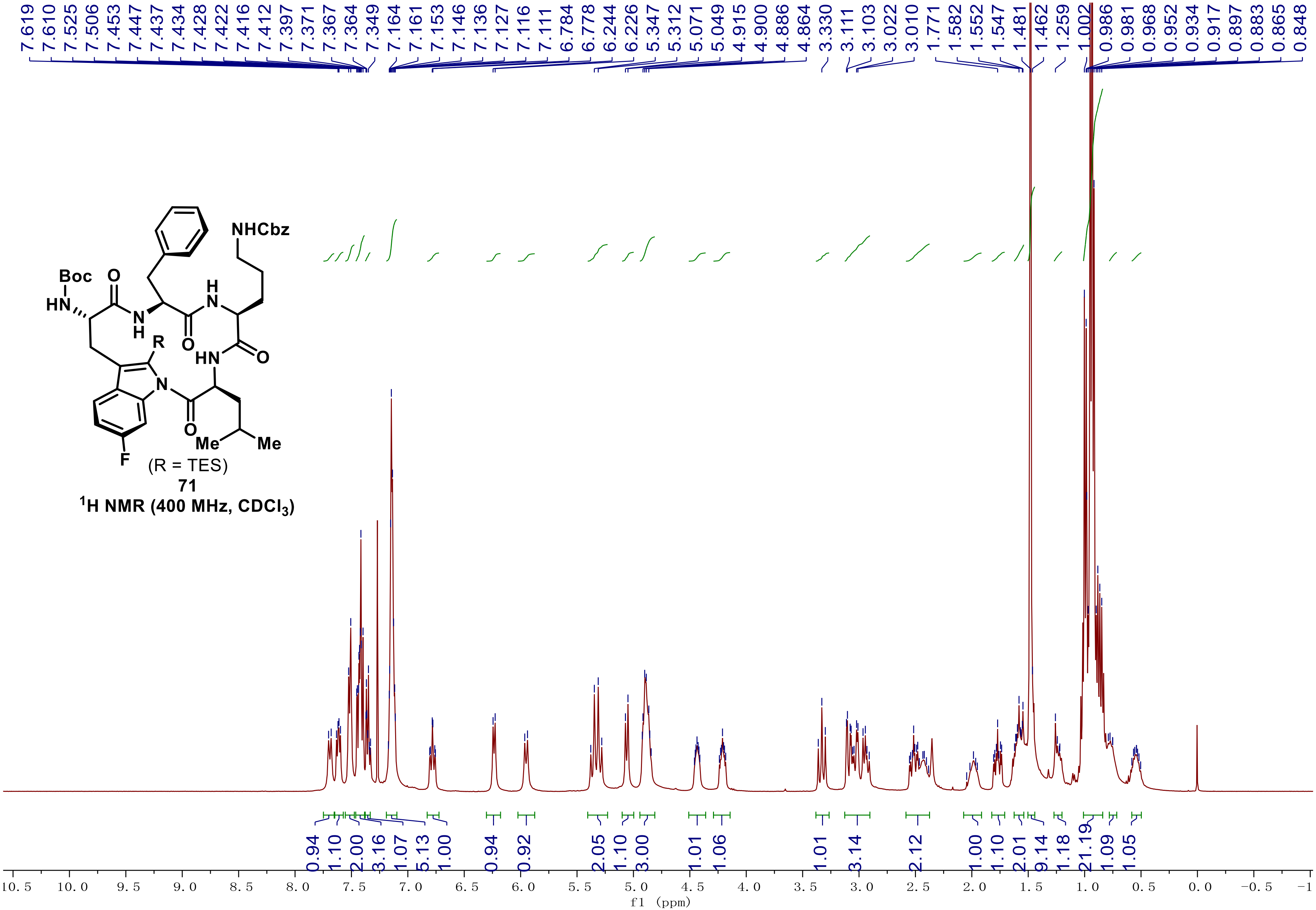

7.619 7.610 7.525 7.506 7.453 7.447 7.437 7.434 7.428 7.422 7.416 7.412 7.397 7.371 7.367 7.364 7.349 7.164 7.161 7.153 7.146 7.136 7.127 7.116 7.111 6.784 6.778 6.244 6.226 5.347 5.312 5.071 5.049 4.915 4.900 4.886 4.864 3.330 3.111 3.103 3.022 3.010 1.771 1.582 1.552 1.547 1.481 1.462 1.259 1.002 0.986 0.981 0.968 0.952 0.934 0.917 0.897 0.883 0.865 0.848
NHCbz
Boc
HN
O
N
H
H
N
R
O
HN
O
N
O
Me
Me
F
(R = TES)
71
^{1}H NMR (400 MHz, $CDCl_3$)
0.94 1.10 2.00 3.16 1.07 5.13 1.00 0.94 0.92 2.05 1.10 3.00 1.01 1.06 1.01 3.14 2.12 1.00 1.10 2.01 9.14 1.18 21.19 1.09 1.05
10.5 10.0 9.5 9.0 8.5 8.0 7.5 7.0 6.5 6.0 5.5 5.0 4.5 4.0 3.5 3.0 2.5 2.0 1.5 1.0 0.5 0.0 −0.5 −1
f1 (ppm)

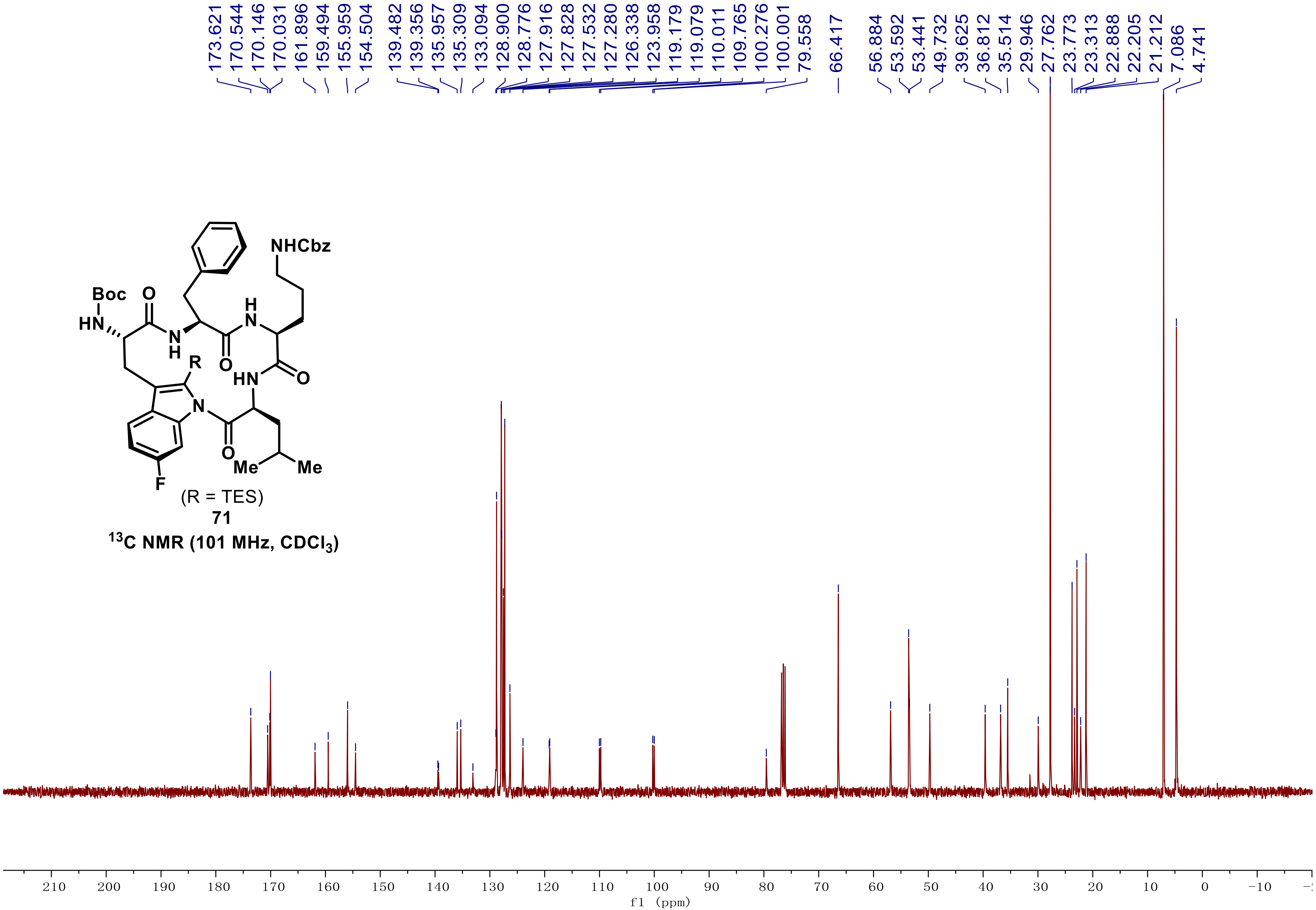
173.621
170.544
170.146
170.031
161.896
159.494
155.959
154.504
139.482
139.356
135.957
135.309
133.094
128.900
128.776
127.916
127.828
127.532
127.280
126.338
123.958
119.179
119.079
110.011
109.765
100.276
100.001
79.558
66.417
56.884
53.592
53.441
49.732
39.625
36.812
35.514
29.946
27.762
23.773
23.313
22.888
22.205
21.212
7.086
4.741
NHCbz
Boc
HN
O
N
H
H
N
R
O
HN
O
N
O
Me
Me
F
(R = TES)
71
^{13}C NMR (101 MHz, $CDCl_3$)
f1 (ppm)

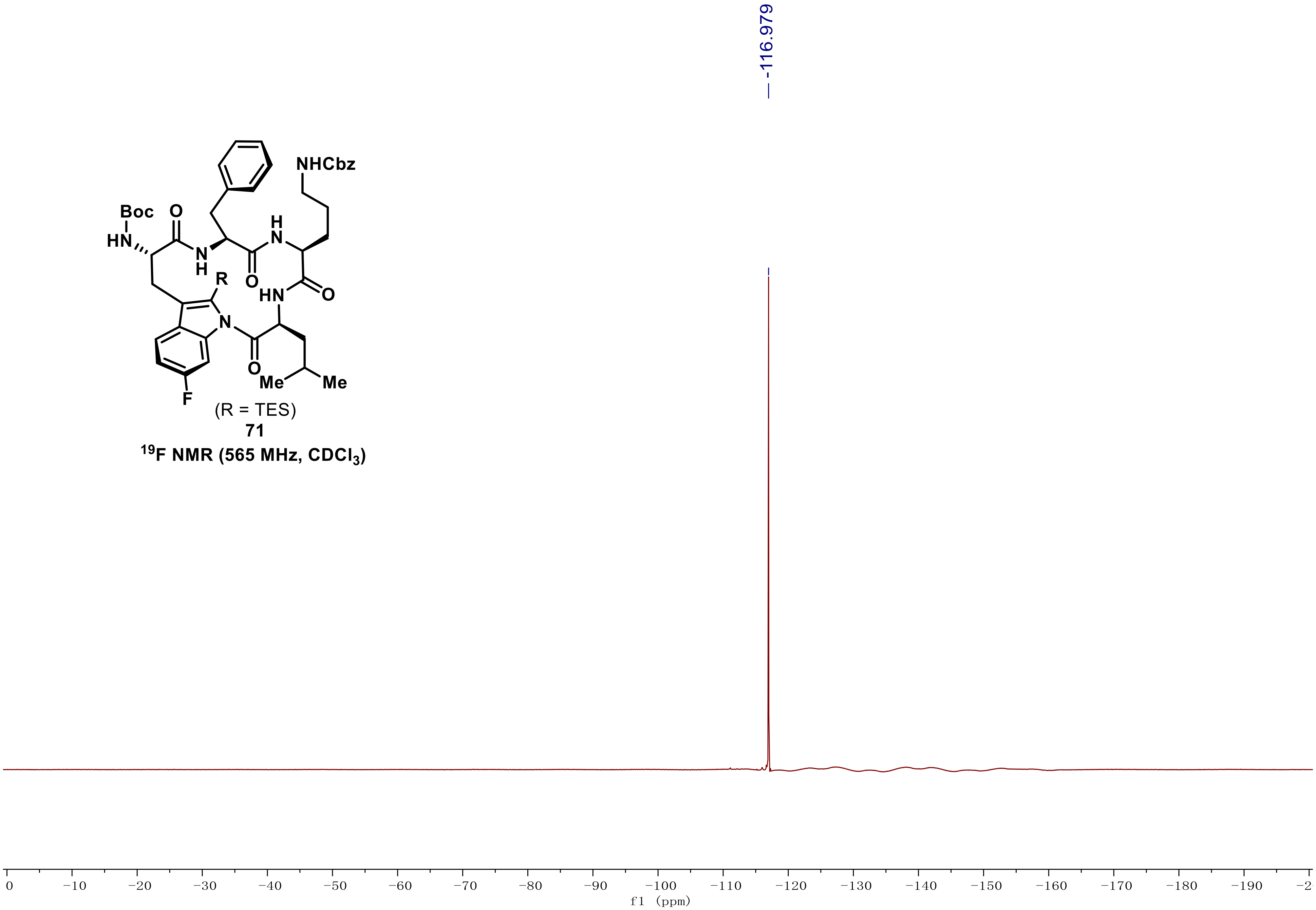

(R = TES)
71
^{19}F NMR (565 MHz, $CDCl_3$)

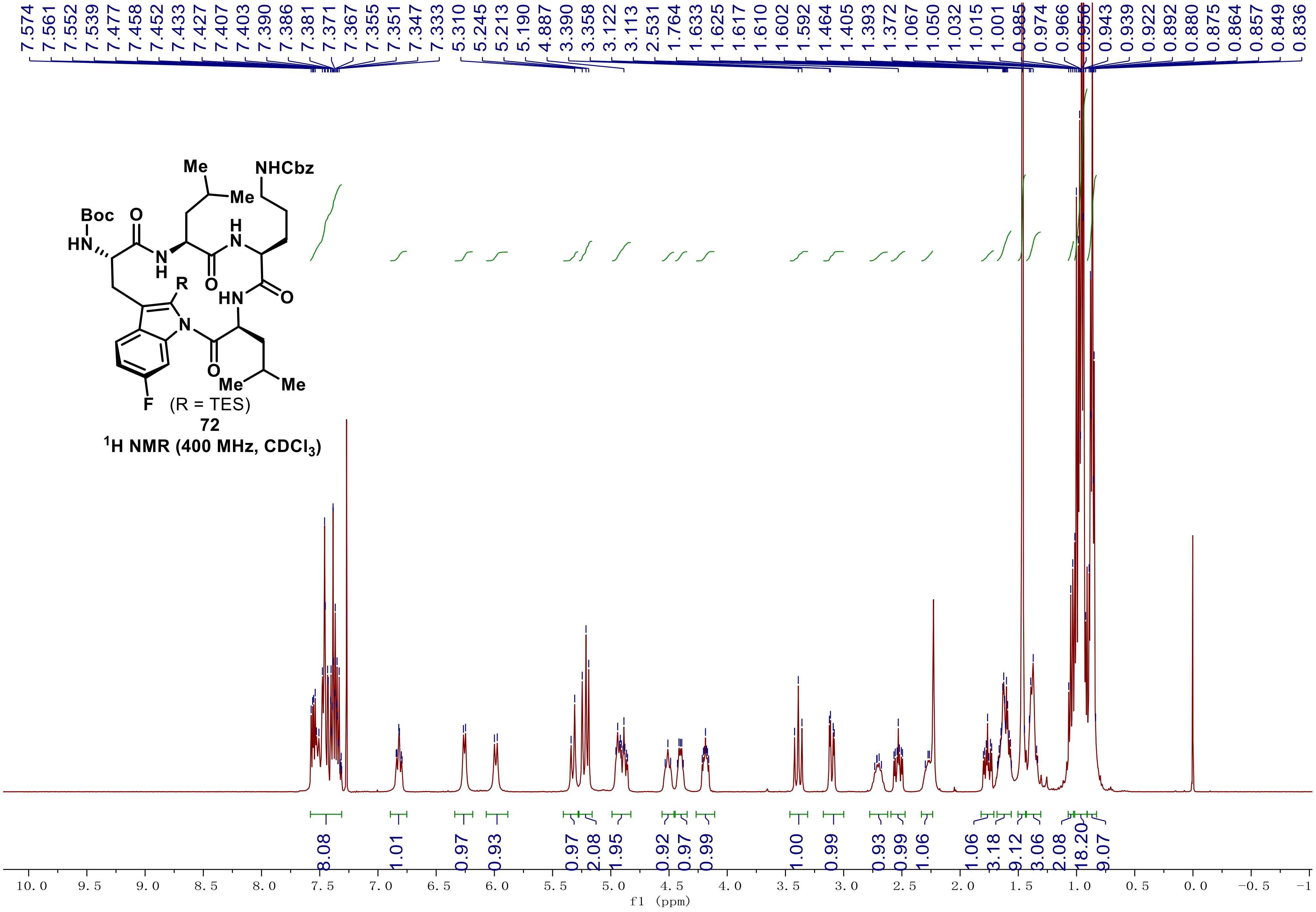
7.574 7.561 7.552 7.539 7.477 7.458 7.452 7.433 7.427 7.407 7.403 7.390 7.386 7.381 7.371 7.367 7.355 7.351 7.347 7.333 5.310 5.245 5.213 5.190 4.887 3.390 3.358 3.122 3.113 2.531 1.764 1.633 1.625 1.617 1.610 1.602 1.592 1.464 1.405 1.393 1.372 1.067 1.050 1.032 1.015 1.001 0.985 0.974 0.966 0.956 0.943 0.939 0.922 0.892 0.880 0.875 0.864 0.857 0.849 0.836
Me
NHCbz
Me
Boc
O
H
HN
N
N
H
R
O
HN
O
N
O
Me
Me
F
(R = TES)
72
¹H NMR (400 MHz, CDCl₃)
8.08 1.01 0.97 0.93 0.97 2.08 1.95 0.92 0.97 0.99 1.00 0.99 0.93 0.99 1.06 1.06 3.18 9.12 3.06 2.08 18.20 9.07
10.0 9.5 9.0 8.5 8.0 7.5 7.0 6.5 6.0 5.5 5.0 4.5 4.0 3.5 3.0 2.5 2.0 1.5 1.0 0.5 0.0 −0.5 −1
f1 (ppm)

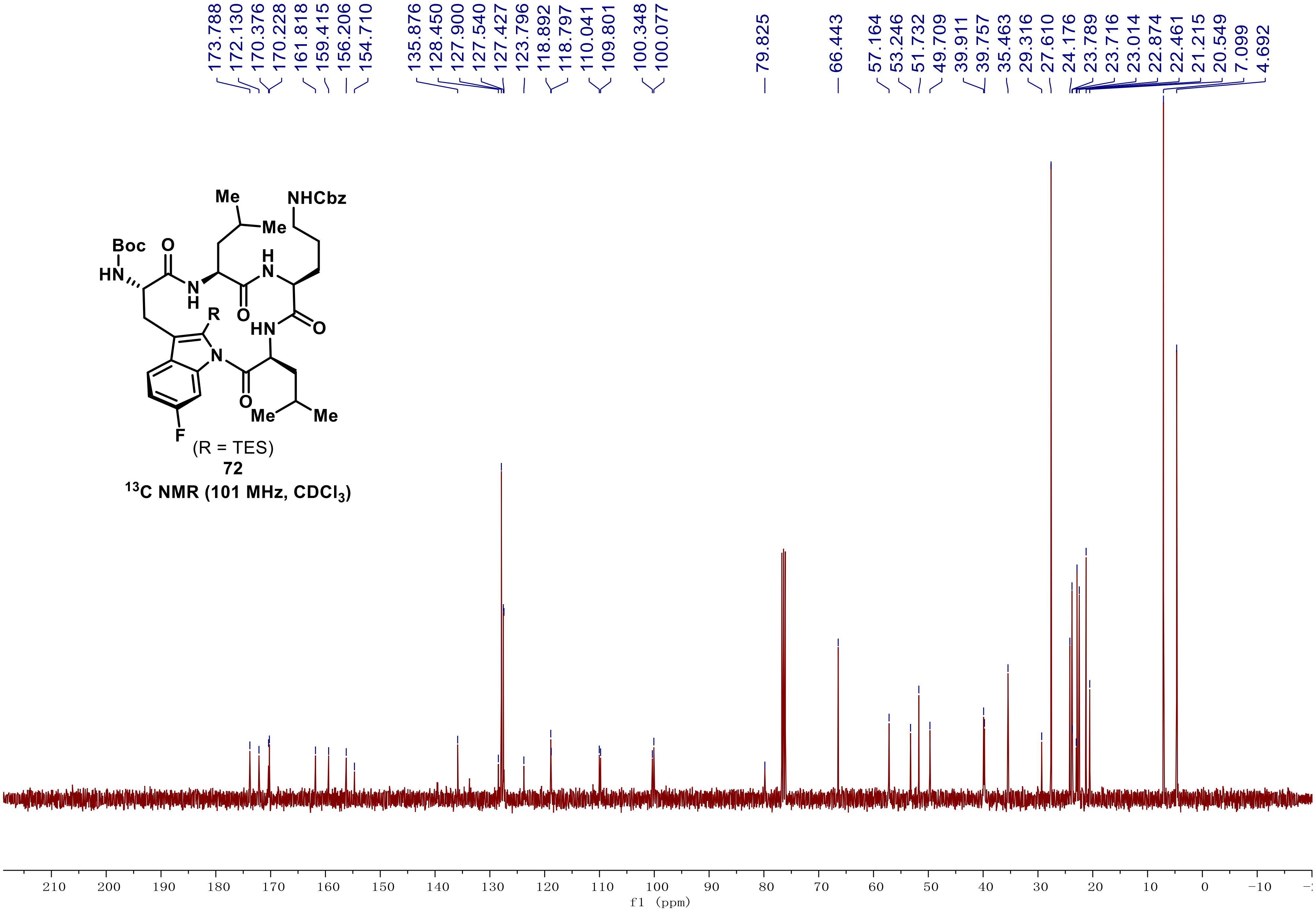
173.788
172.130
170.376
170.228
161.818
159.415
156.206
154.710
135.876
128.450
127.900
127.540
127.427
123.796
118.892
118.797
110.041
109.801
100.348
100.077
79.825
66.443
57.164
53.246
51.732
49.709
39.911
39.757
35.463
29.316
27.610
24.176
23.789
23.716
23.014
22.874
22.461
21.215
20.549
7.099
4.692
Me
NHCbz
Me
Boc
O
HN
H
N
N
H
R
O
HN
O
N
O
Me
Me
F
(R = TES)
72
^{13}C NMR (101 MHz, CDCl$_3$)
f1 (ppm)

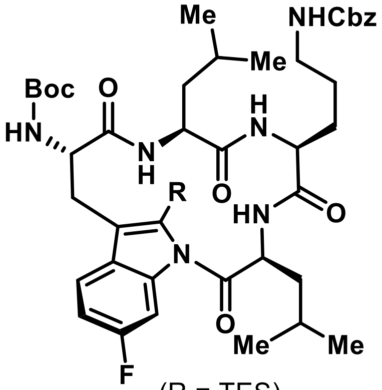

(R = TES)

72

^{19}F NMR (565 MHz, CDCl$_3$)

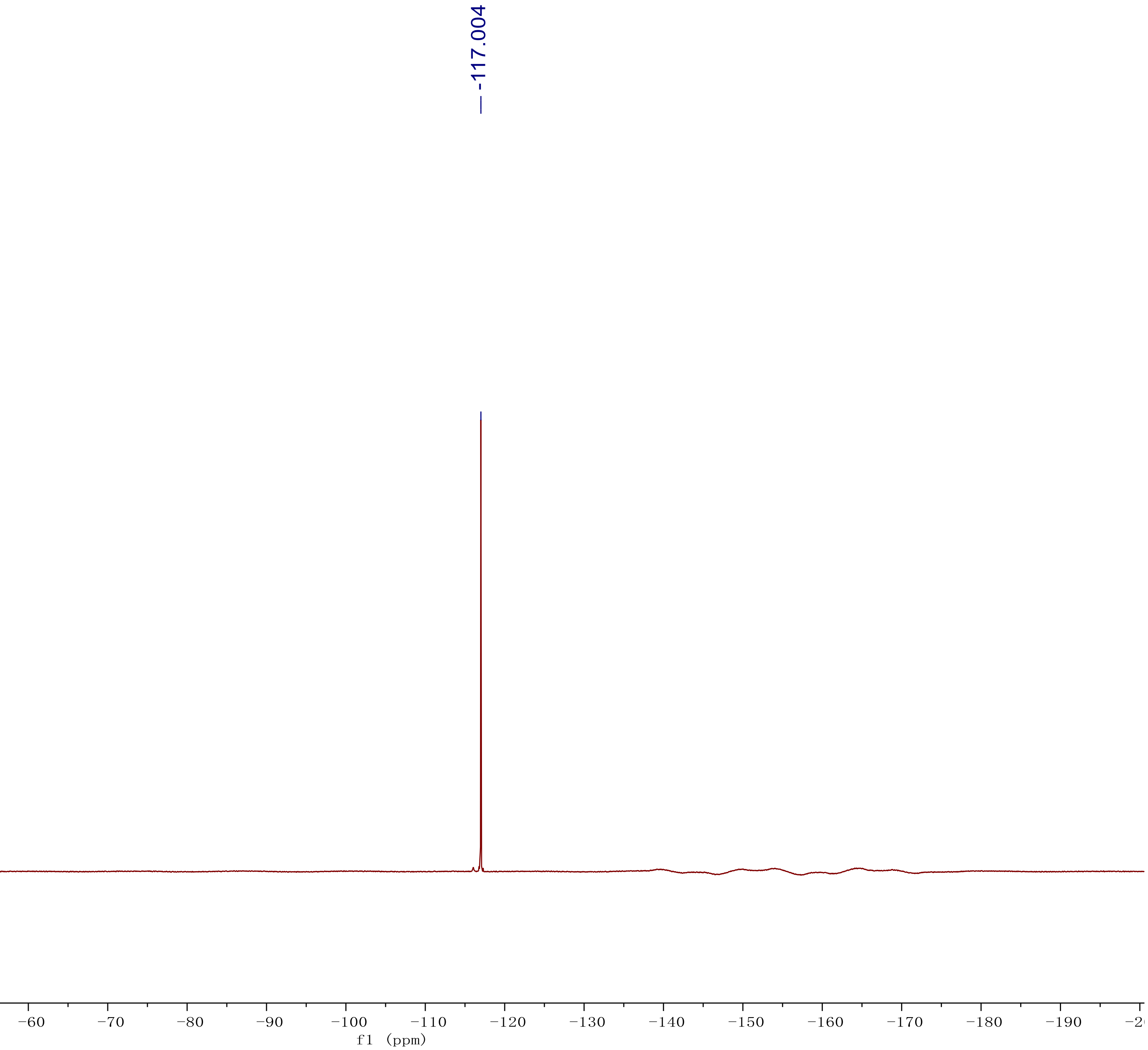

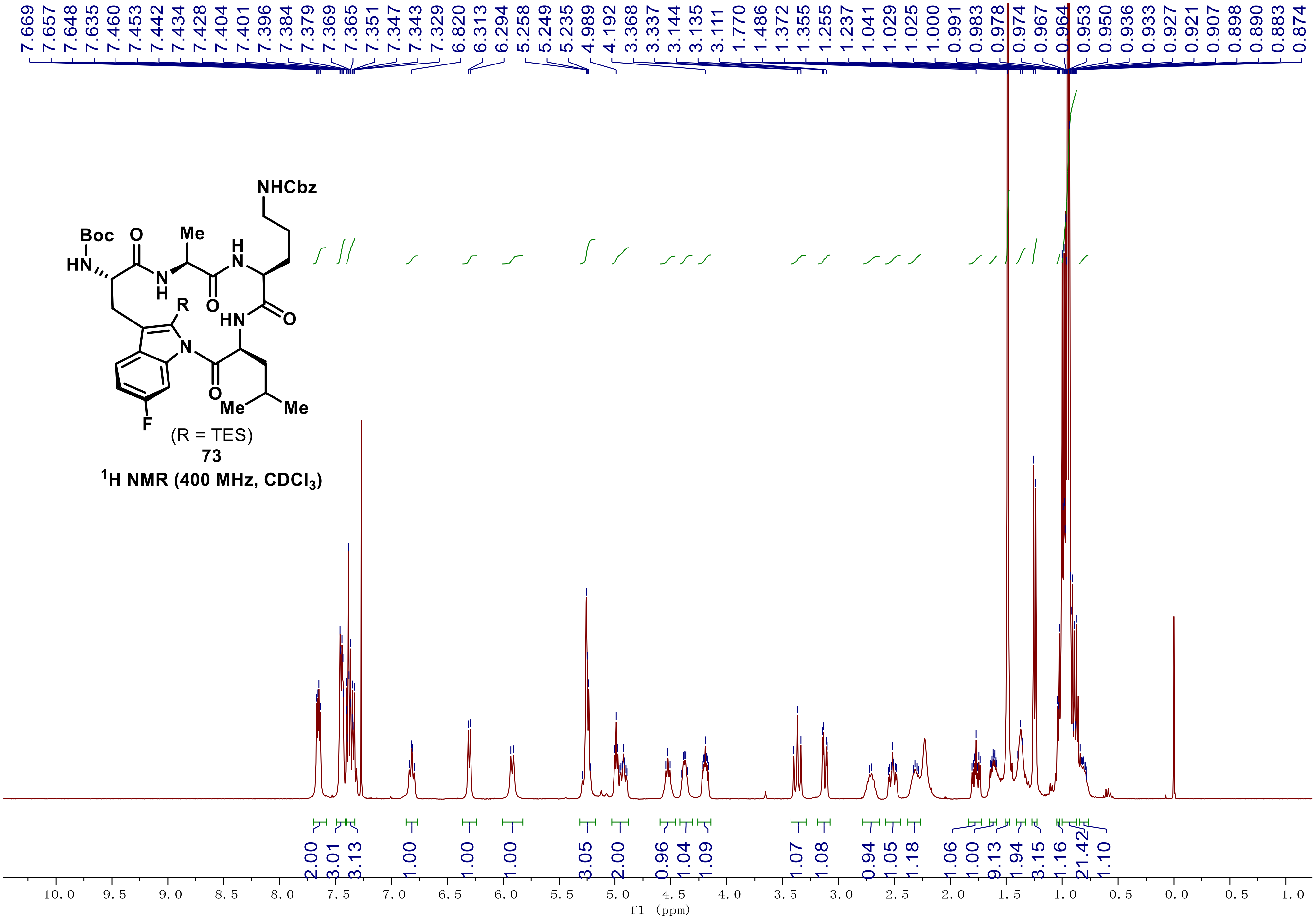
7.669 7.657 7.648 7.635 7.460 7.453 7.442 7.434 7.428 7.404 7.401 7.396 7.384 7.379 7.369 7.365 7.351 7.347 7.343 7.329 6.820 6.313 6.294 5.258 5.249 5.235 4.989 4.192 3.368 3.337 3.144 3.135 3.111 1.770 1.486 1.372 1.355 1.255 1.237 1.041 1.029 1.025 1.000 0.991 0.983 0.978 0.974 0.967 0.964 0.953 0.950 0.936 0.933 0.927 0.921 0.907 0.898 0.890 0.883 0.874
NHCbz
Boc
HN
O
Me
H
N
R
HN
N
F
Me
Me
(R = TES)
73
1H NMR (400 MHz, CDCl3)
2.00 3.01 3.13 1.00 1.00 1.00 3.05 2.00 0.96 1.04 1.09 1.07 1.08 0.94 1.05 1.18 1.06 1.00 9.13 1.94 3.15 1.16 21.42 1.10
10.0 9.5 9.0 8.5 8.0 7.5 7.0 6.5 6.0 5.5 5.0 4.5 4.0 3.5 3.0 2.5 2.0 1.5 1.0 0.5 0.0 -0.5 -1.0
f1 (ppm)

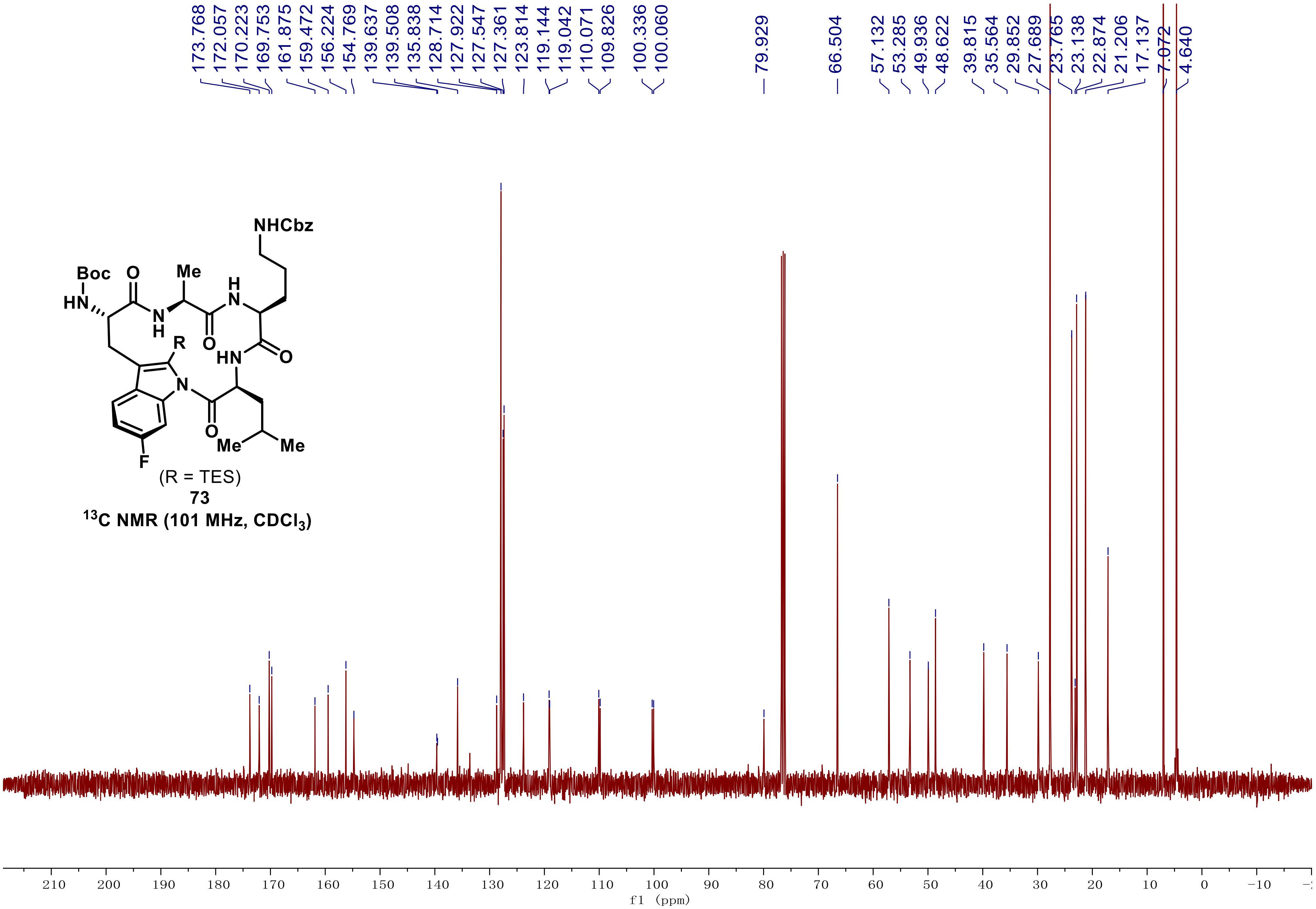
173.768
172.057
170.223
169.753
161.875
159.472
156.224
154.769
139.637
139.508
135.838
128.714
127.922
127.547
127.361
123.814
119.144
119.042
110.071
109.826
100.336
100.060
79.929
66.504
57.132
53.285
49.936
48.622
39.815
35.564
29.852
27.689
23.765
23.138
22.874
21.206
17.137
7.072
4.640
NHCbz
Boc
O
Me
HN
N
H
R
HN
O
N
O
Me
Me
F
(R = TES)
73
^{13}C NMR (101 MHz, CDCl$_3$)
210
200
190
180
170
160
150
140
130
120
110
100
90
80
70
60
50
40
30
20
10
0
−10
f1 (ppm)

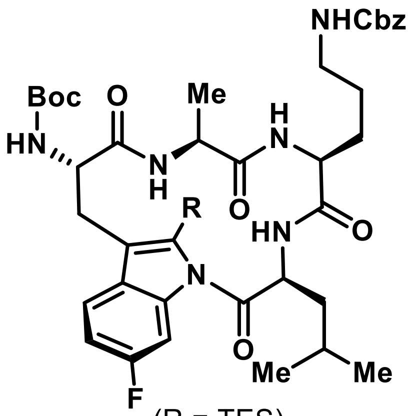

(R = TES)

73

^{19}F NMR (565 MHz, $CDCl_3$)

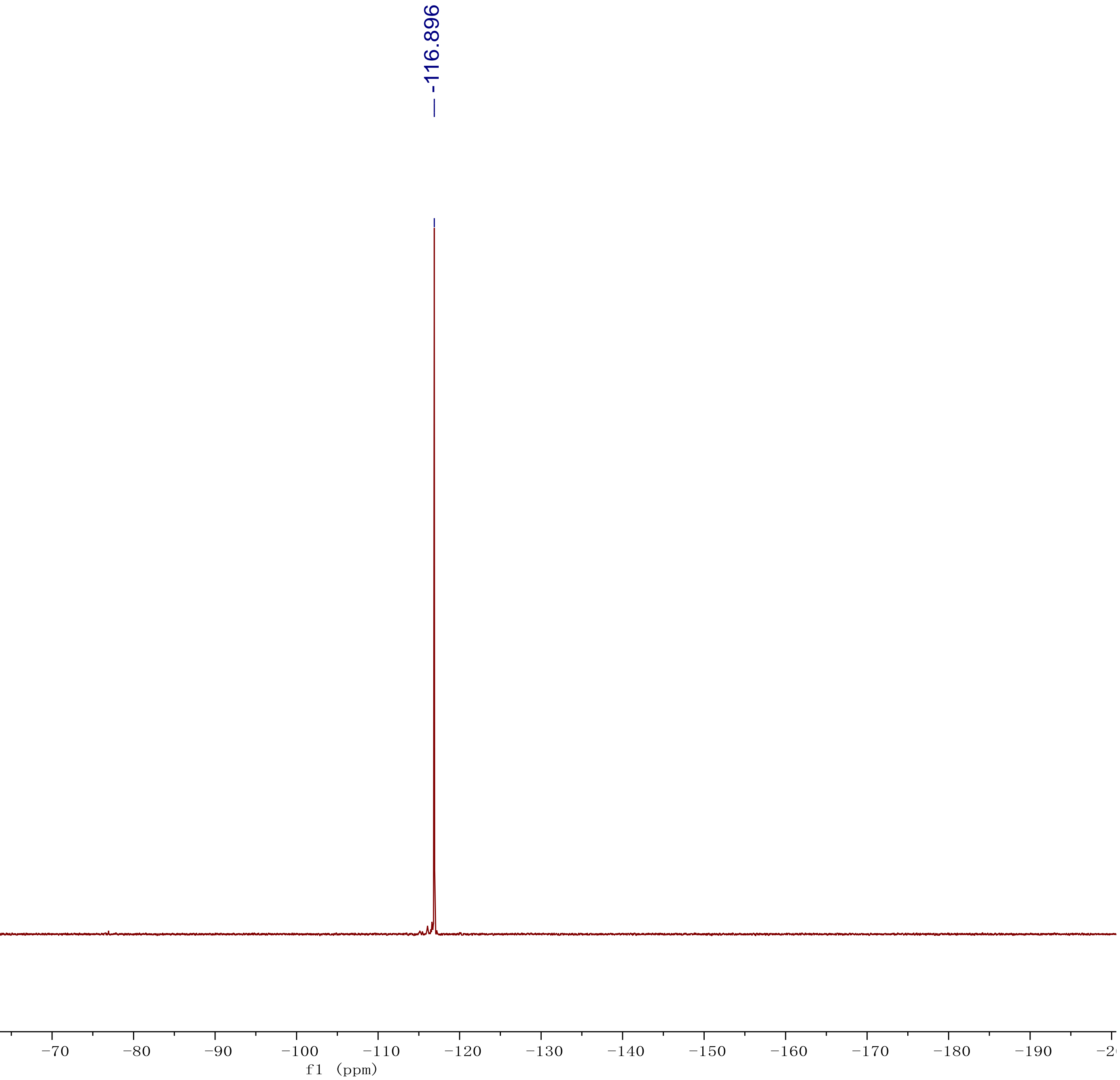

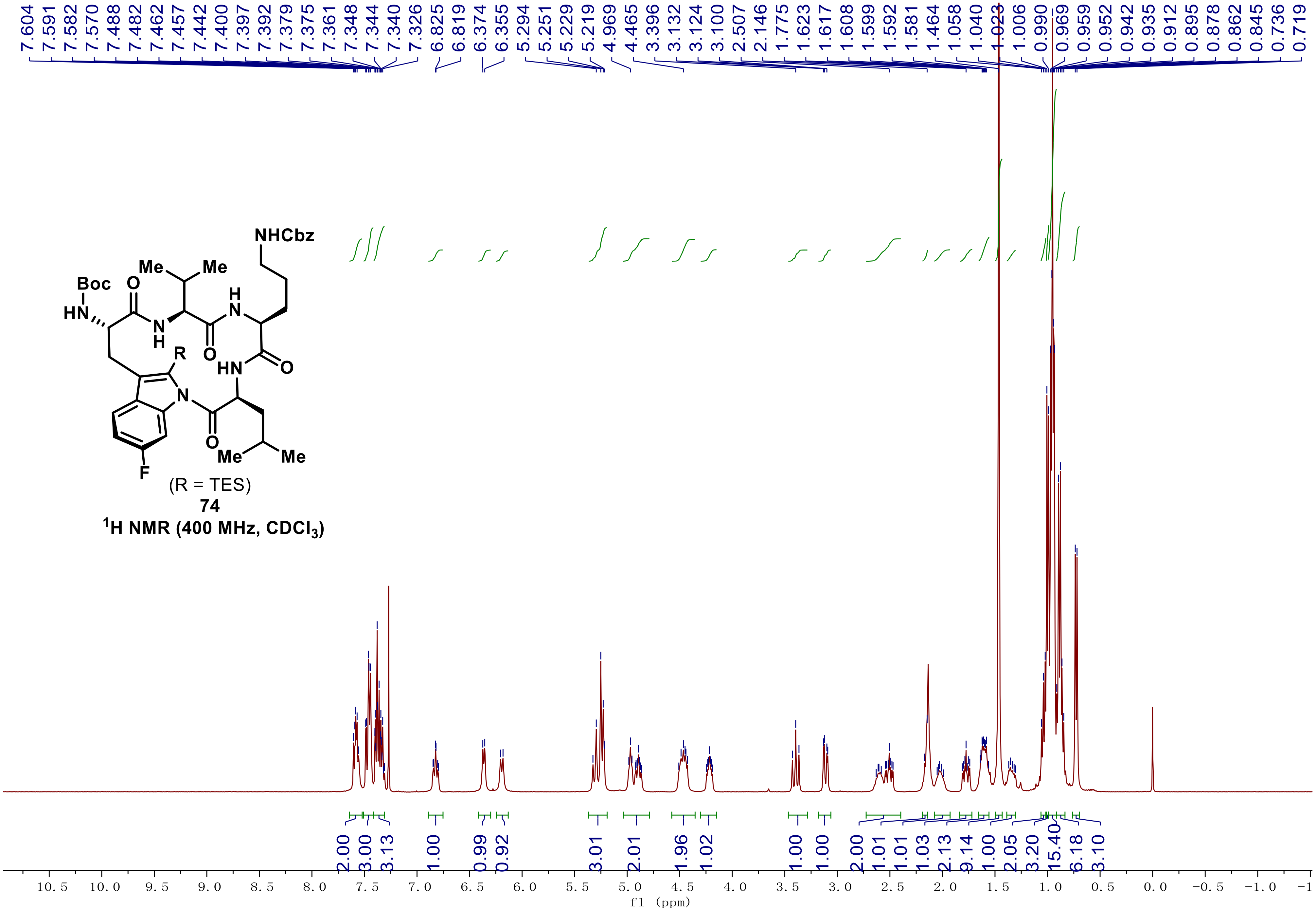

(R = TES)
74
^{1}H NMR (400 MHz, $CDCl_3$)

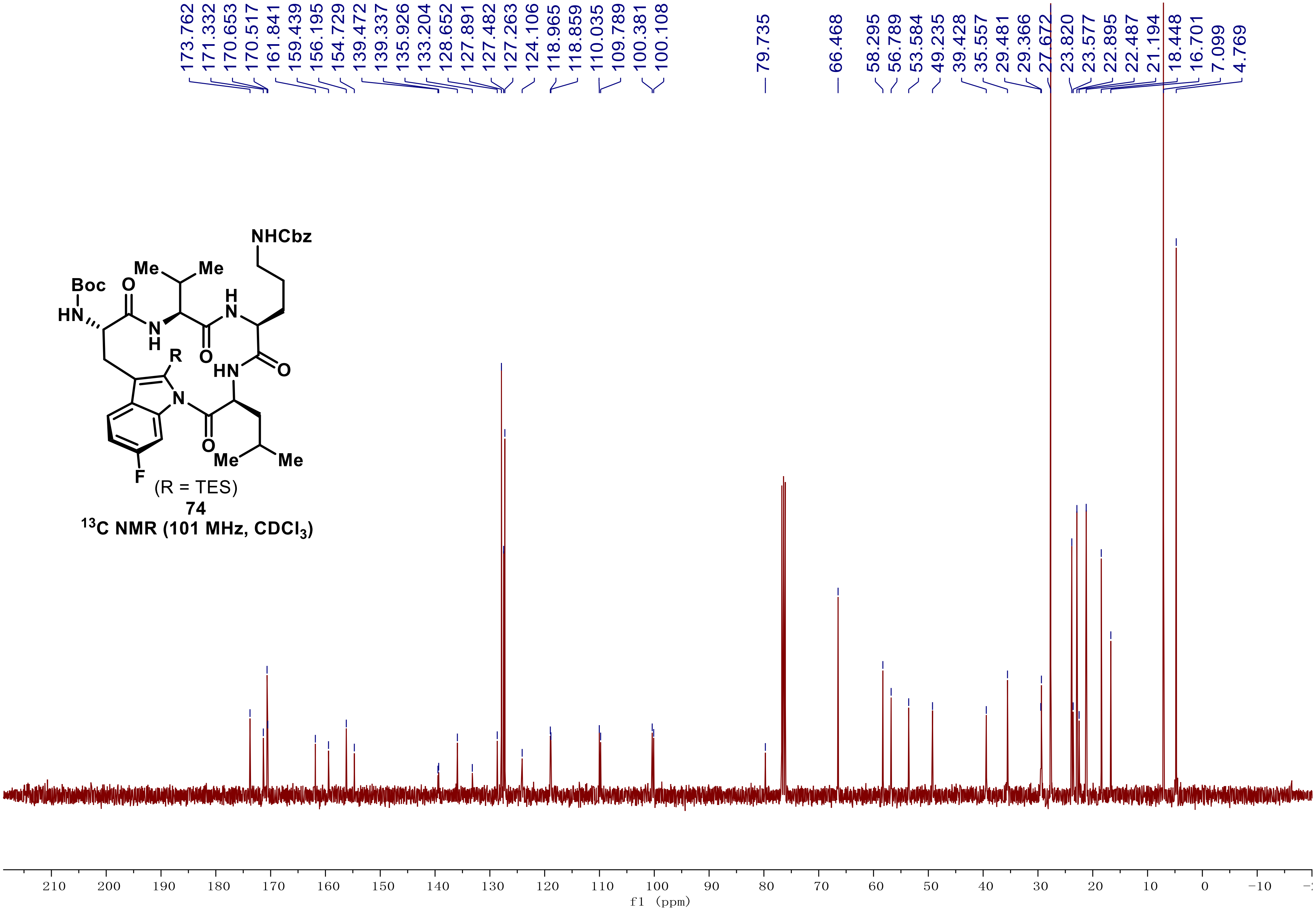
173.762
171.332
170.653
170.517
161.841
159.439
156.195
154.729
139.472
139.337
135.926
133.204
128.652
127.891
127.482
127.263
124.106
118.965
118.859
110.035
109.789
100.381
100.108
79.735
66.468
58.295
56.789
53.584
49.235
39.428
35.557
29.481
29.366
27.672
23.820
23.577
22.895
22.487
21.194
18.448
16.701
7.099
4.769
NHCbz
Me
Me
Boc
O
HN
N
H
H
N
R
O
HN
O
N
O
Me
Me
F
(R = TES)
74
^{13}C NMR (101 MHz, $CDCl_3$)
210 200 190 180 170 160 150 140 130 120 110 100 90 80 70 60 50 40 30 20 10 0 −10
f1 (ppm)

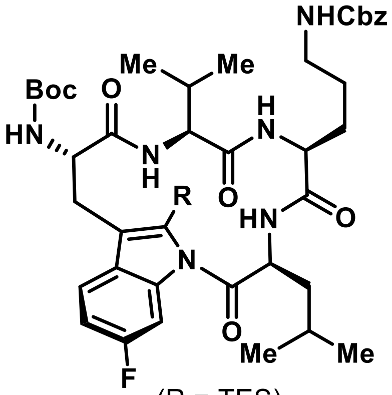

(R = TES)

74

^{19}F NMR (565 MHz, $CDCl_3$)

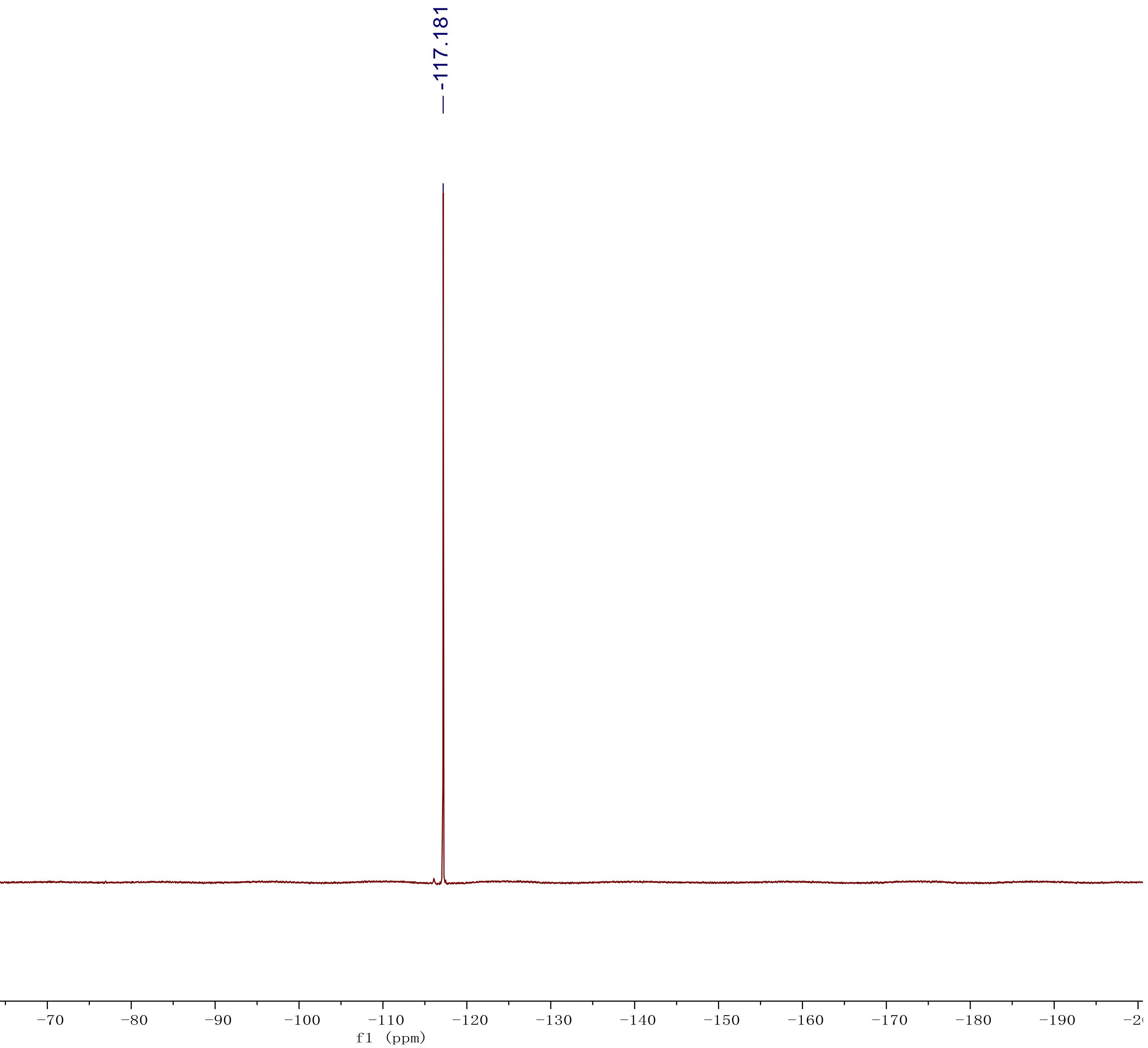

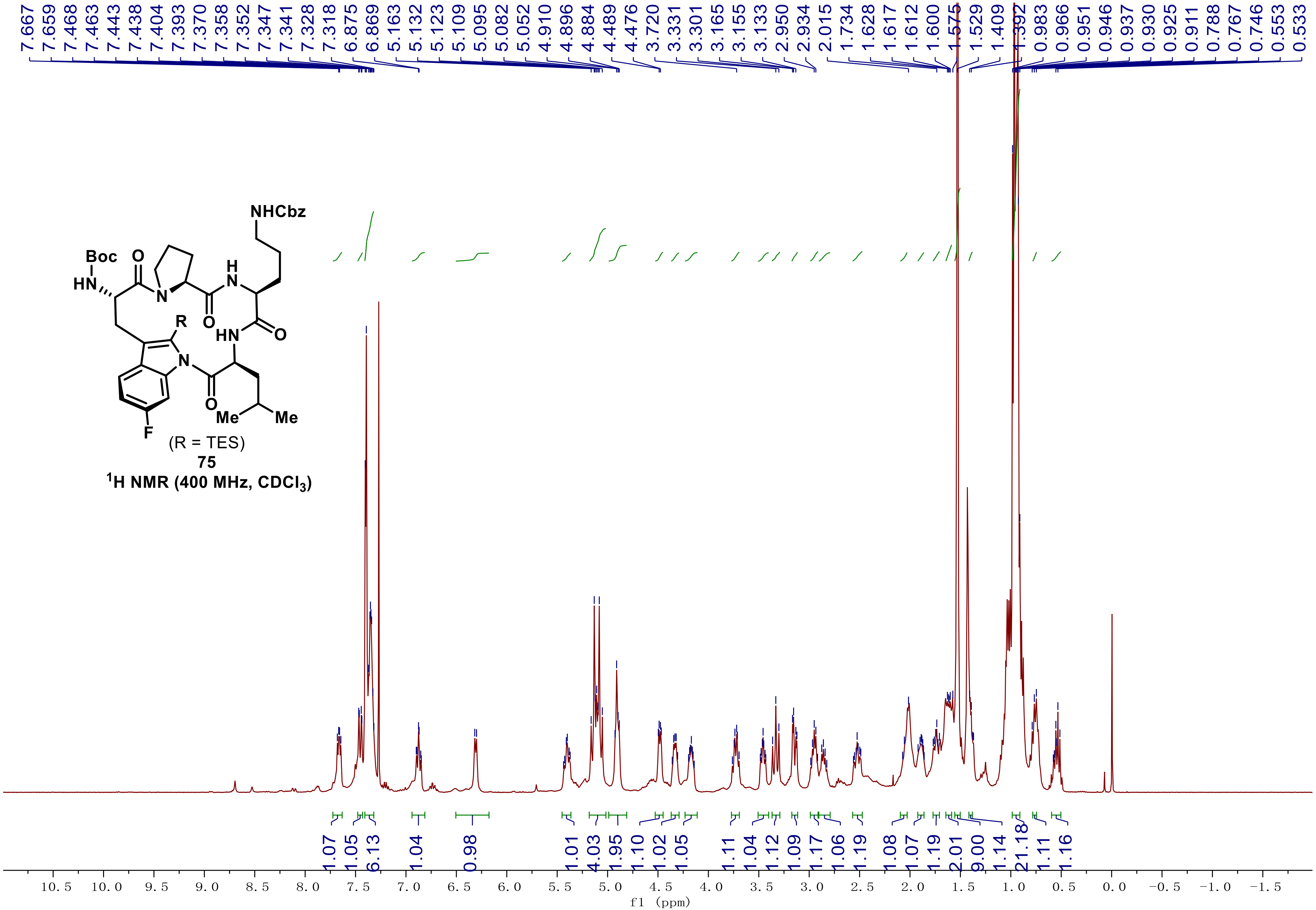
NHCbz
Boc
HN
O
N
H
N
O
R
HN
O
N
O
Me
Me
F
(R = TES)
75
1H NMR (400 MHz, CDCl3)
f1 (ppm)

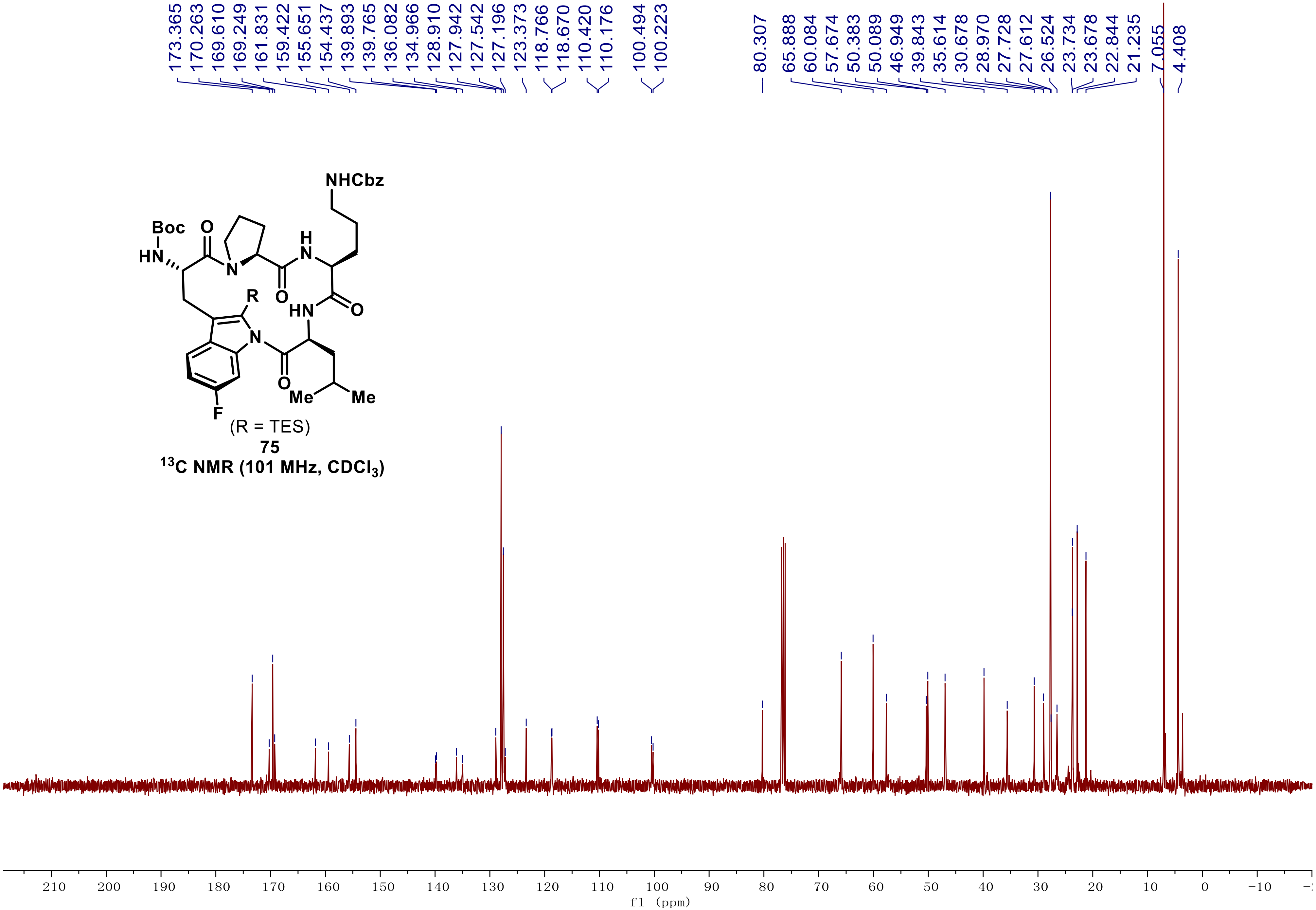
173.365
170.263
169.610
169.249
161.831
159.422
155.651
154.437
139.893
139.765
136.082
134.966
128.910
127.942
127.542
127.196
123.373
118.766
118.670
110.420
110.176
100.494
100.223
80.307
65.888
60.084
57.674
50.383
50.089
46.949
39.843
35.614
30.678
28.970
27.728
27.612
26.524
23.734
23.678
22.844
21.235
7.055
4.408
NHCbz
Boc
O
HN
N
H
N
R
O
HN
O
N
O
Me
Me
F
(R = TES)
75
13C NMR (101 MHz, CDCl3)
210 200 190 180 170 160 150 140 130 120 110 100 90 80 70 60 50 40 30 20 10 0 −10
f1 (ppm)

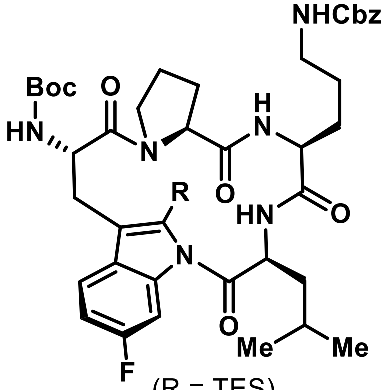

(R = TES)

75

^{19}F NMR (565 MHz, $CDCl_3$)

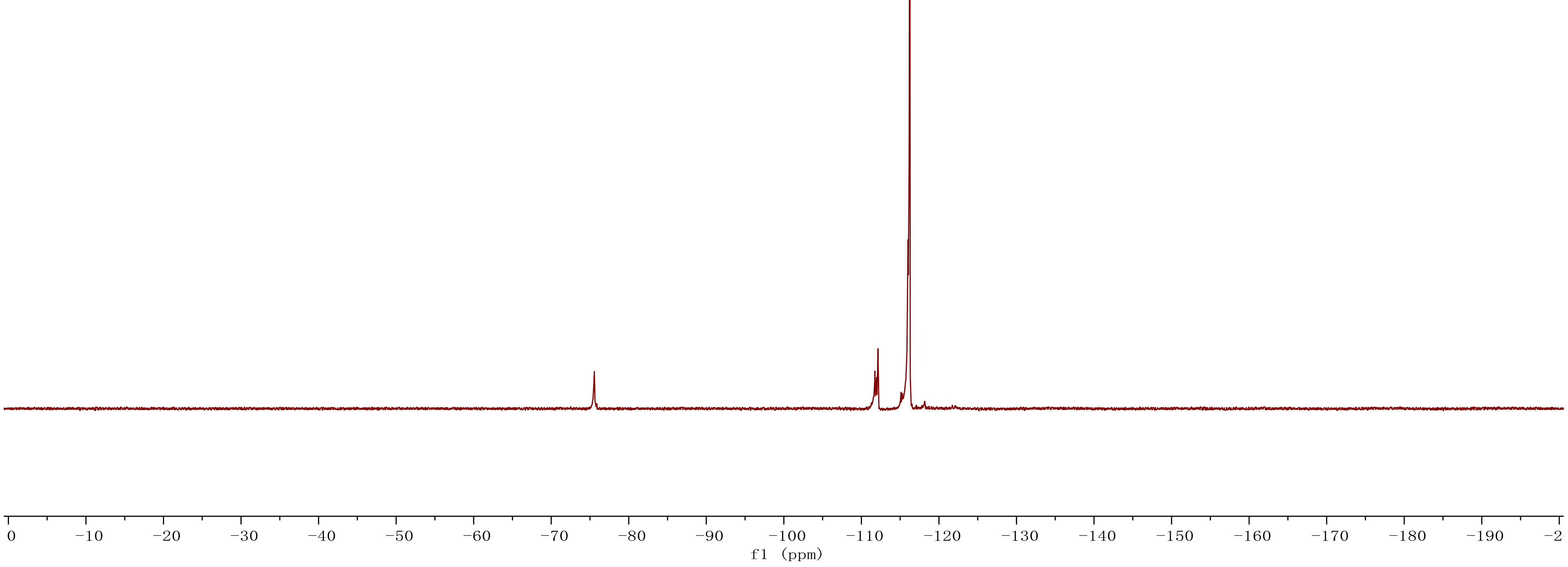

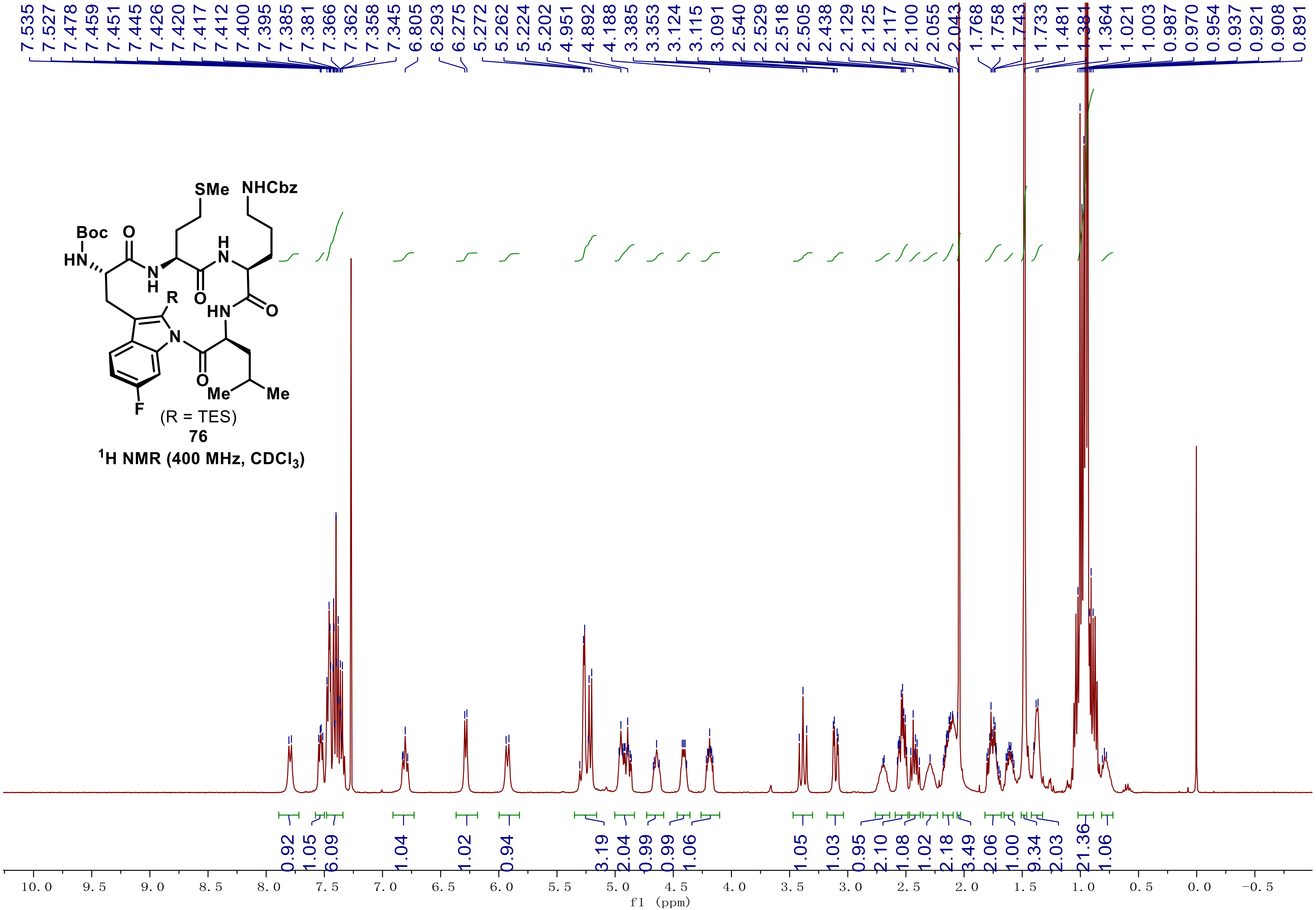

(R = TES)

76

^{1}H NMR (400 MHz, CDCl$_3$)

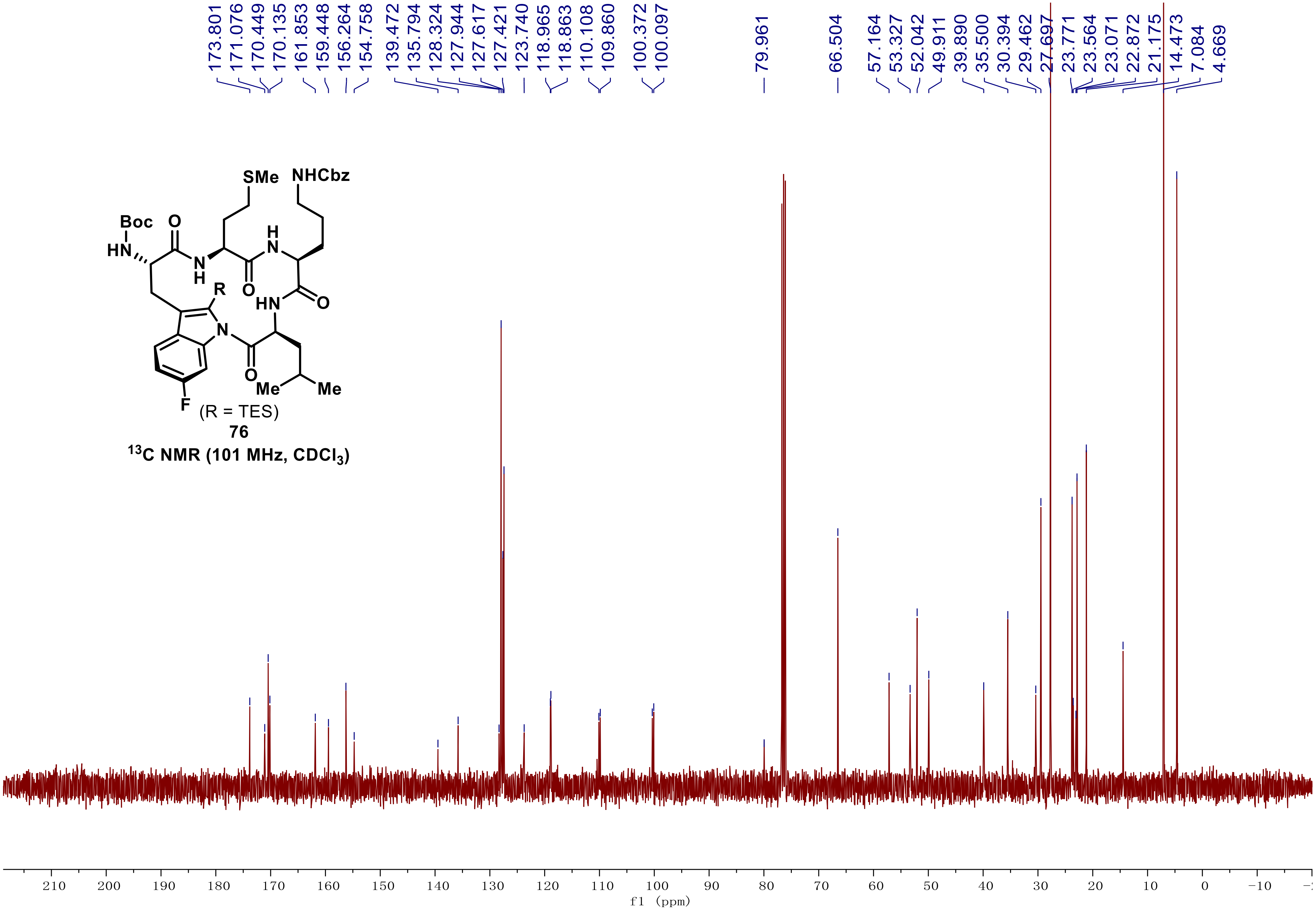

173.801
171.076
170.449
170.135
161.853
159.448
156.264
154.758
139.472
135.794
128.324
127.944
127.617
127.421
123.740
118.965
118.863
110.108
109.860
100.372
100.097
79.961
66.504
57.164
53.327
52.042
49.911
39.890
35.500
30.394
29.462
27.697
23.771
23.564
23.071
22.872
21.175
14.473
7.084
4.669
SMe
NHCbz
Boc
HN
O
N
H
R
N
HN
O
Me
Me
F
(R = TES)
76
13C NMR (101 MHz, CDCl3)
f1 (ppm)

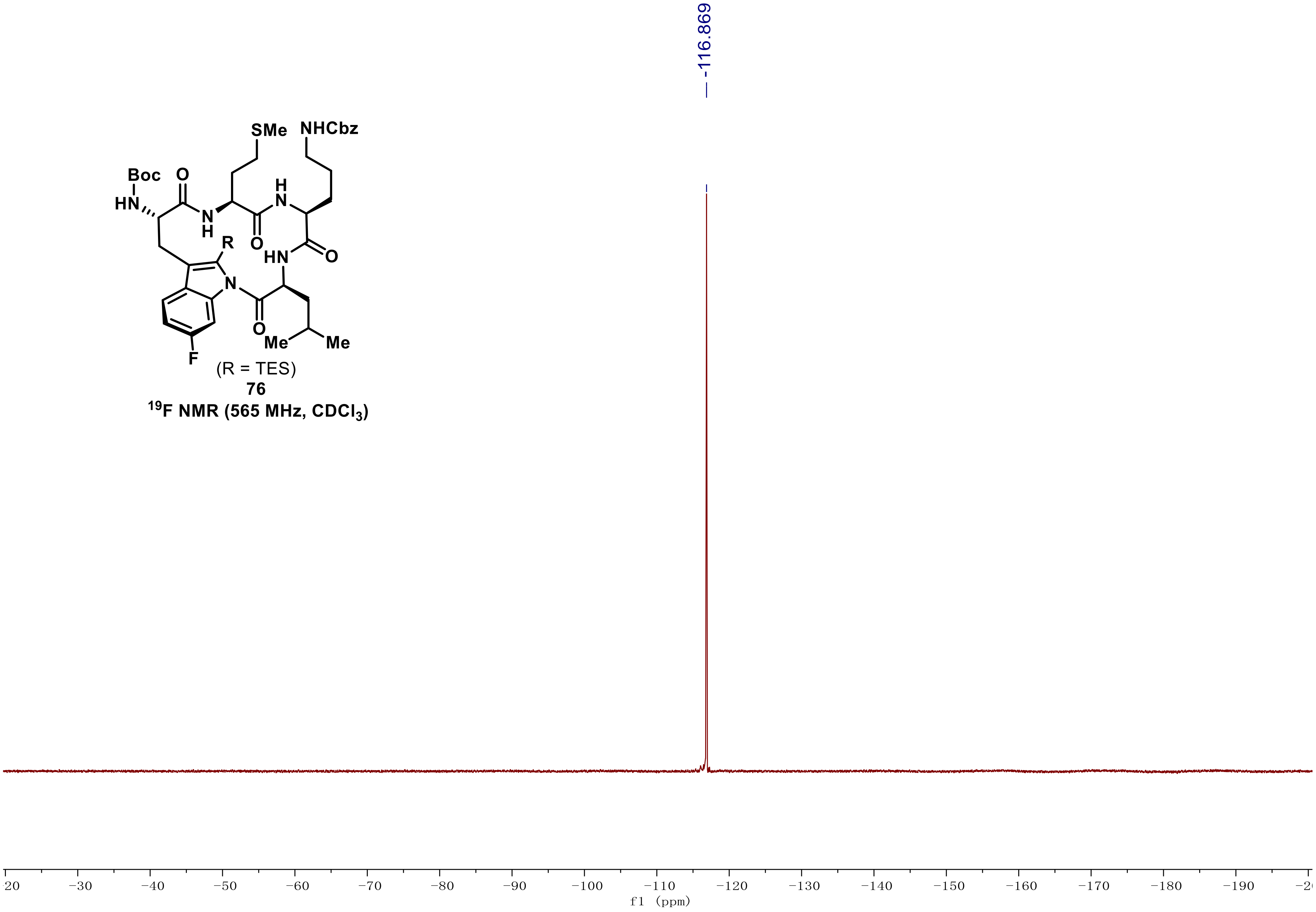
(R = TES)
76
19F NMR (565 MHz, CDCl3)
-116.869
20
−30
−40
−50
−60
−70
−80
−90
−100
−110
−120
−130
−140
−150
−160
−170
−180
−190
f1 (ppm)

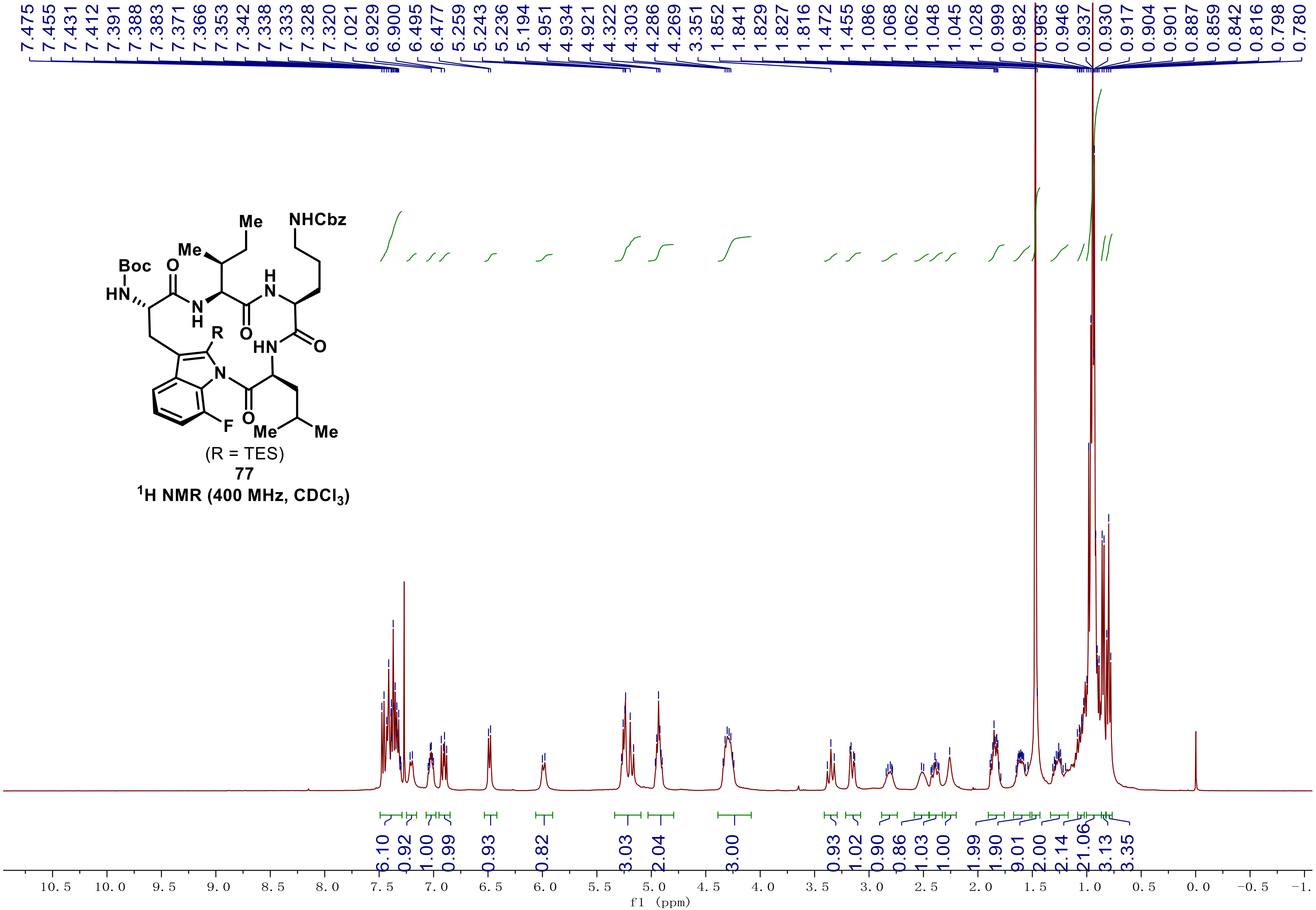

(R = TES)

77

^{1}H NMR (400 MHz, $CDCl_3$)

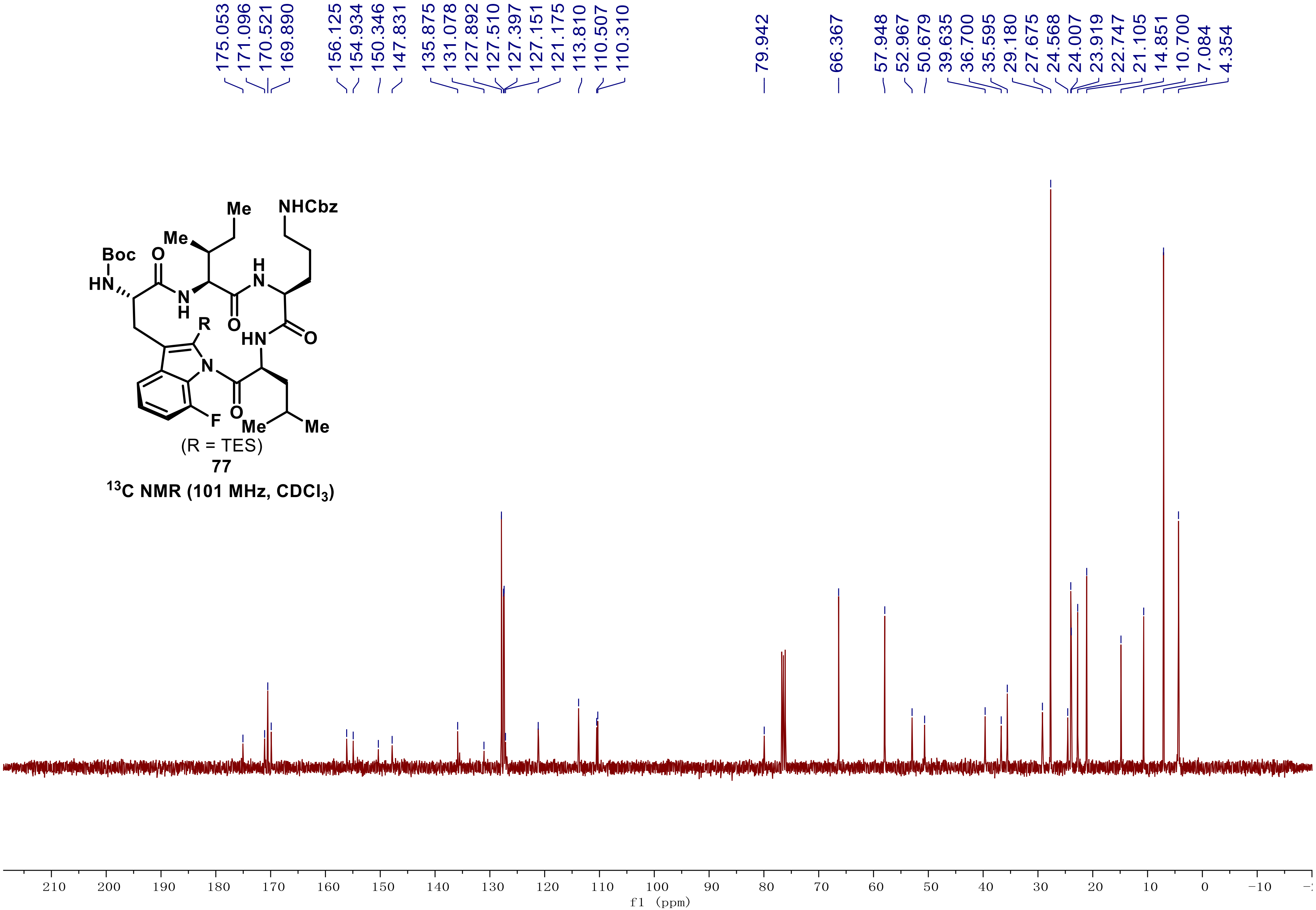

175.053
171.096
170.521
169.890
156.125
154.934
150.346
147.831
135.875
131.078
127.892
127.510
127.397
127.151
121.175
113.810
110.507
110.310
79.942
66.367
57.948
52.967
50.679
39.635
36.700
35.595
29.180
27.675
24.568
24.007
23.919
22.747
21.105
14.851
10.700
7.084
4.354
Me
NHCbz
Me
Boc
O
H
HN
N
N
H
R
O
HN
O
N
O
F
Me
Me
(R = TES)
77
^{13}C NMR (101 MHz, $CDCl_3$)
210
200
190
180
170
160
150
140
130
120
110
100
90
80
70
60
50
40
30
20
10
0
−10
f1 (ppm)

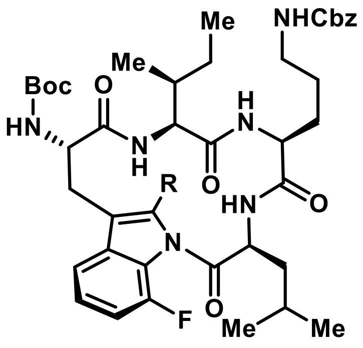

(R = TES)

77

^{19}F NMR (565 MHz, $CDCl_3$)

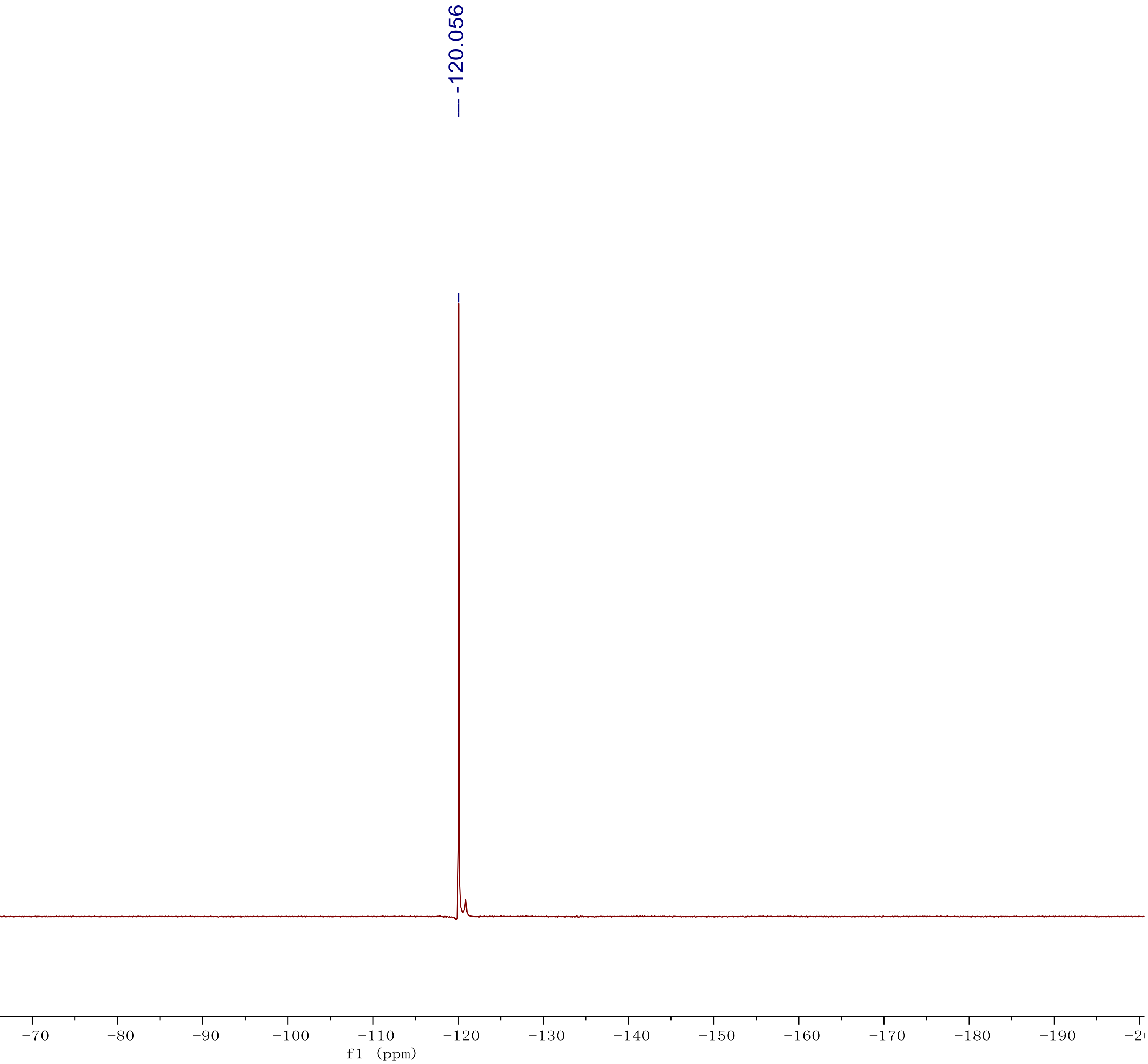

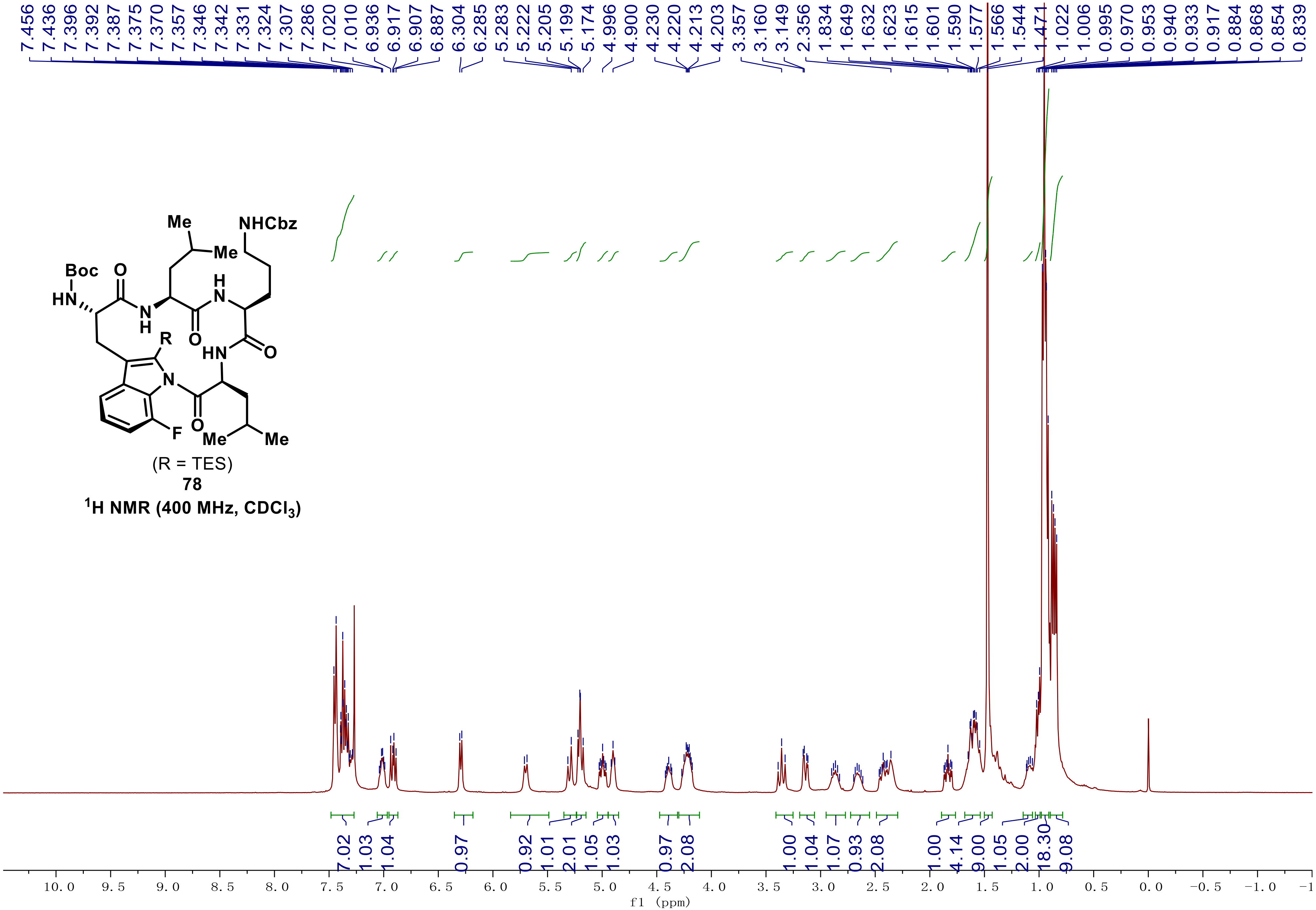

(R = TES)

78

^{1}H NMR (400 MHz, $CDCl_3$)

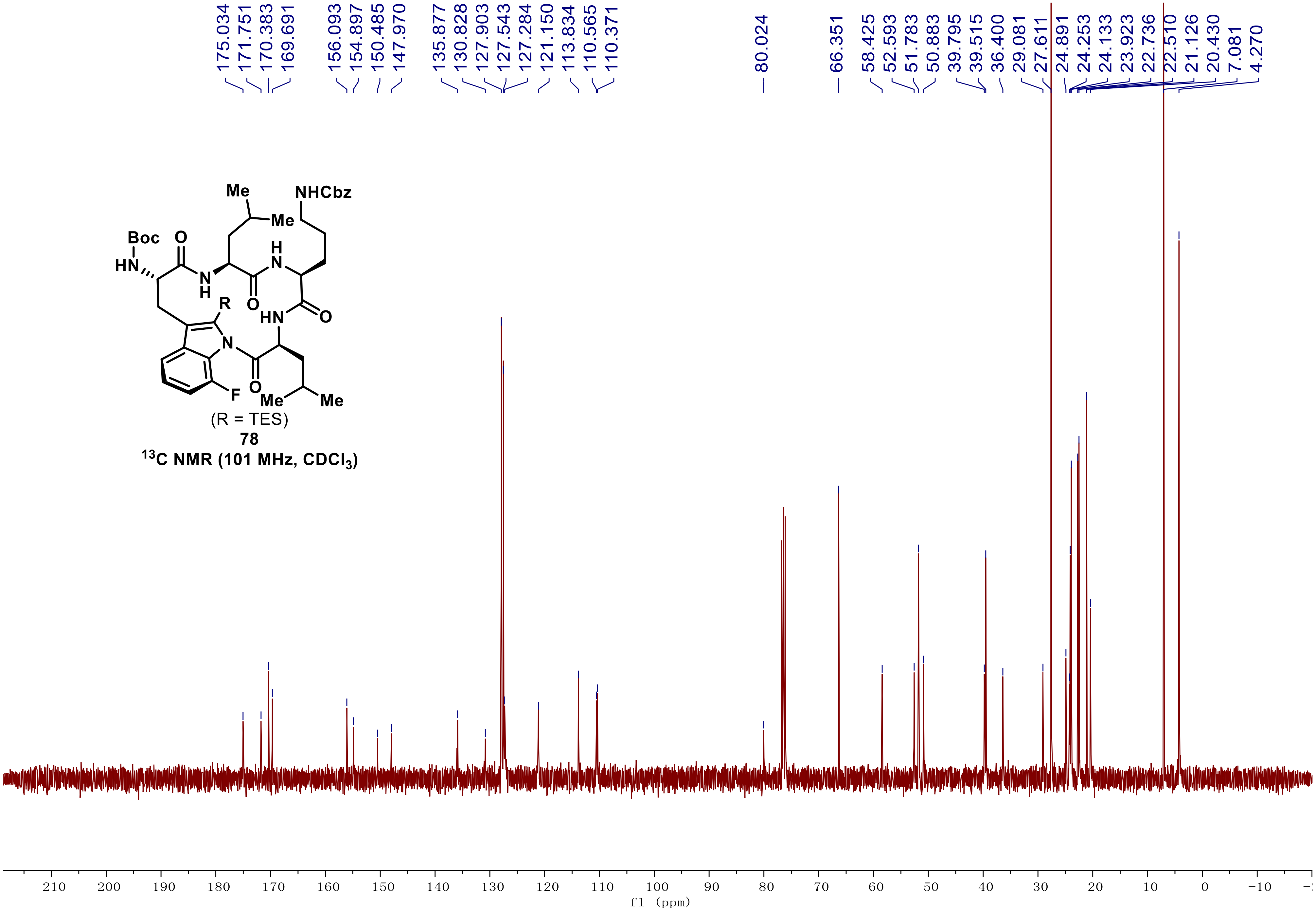
175.034
171.751
170.383
169.691
156.093
154.897
150.485
147.970
135.877
130.828
127.903
127.543
127.284
121.150
113.834
110.565
110.371
80.024
66.351
58.425
52.593
51.783
50.883
39.795
39.515
36.400
29.081
27.611
24.891
24.253
24.133
23.923
22.736
22.510
21.126
20.430
7.081
4.270
Boc
HN
N
H
O
Me
Me
NHCbz
H
N
O
R
HN
O
N
F
O
Me
Me
(R = TES)
78
^{13}C NMR (101 MHz, $CDCl_3$)
f1 (ppm)

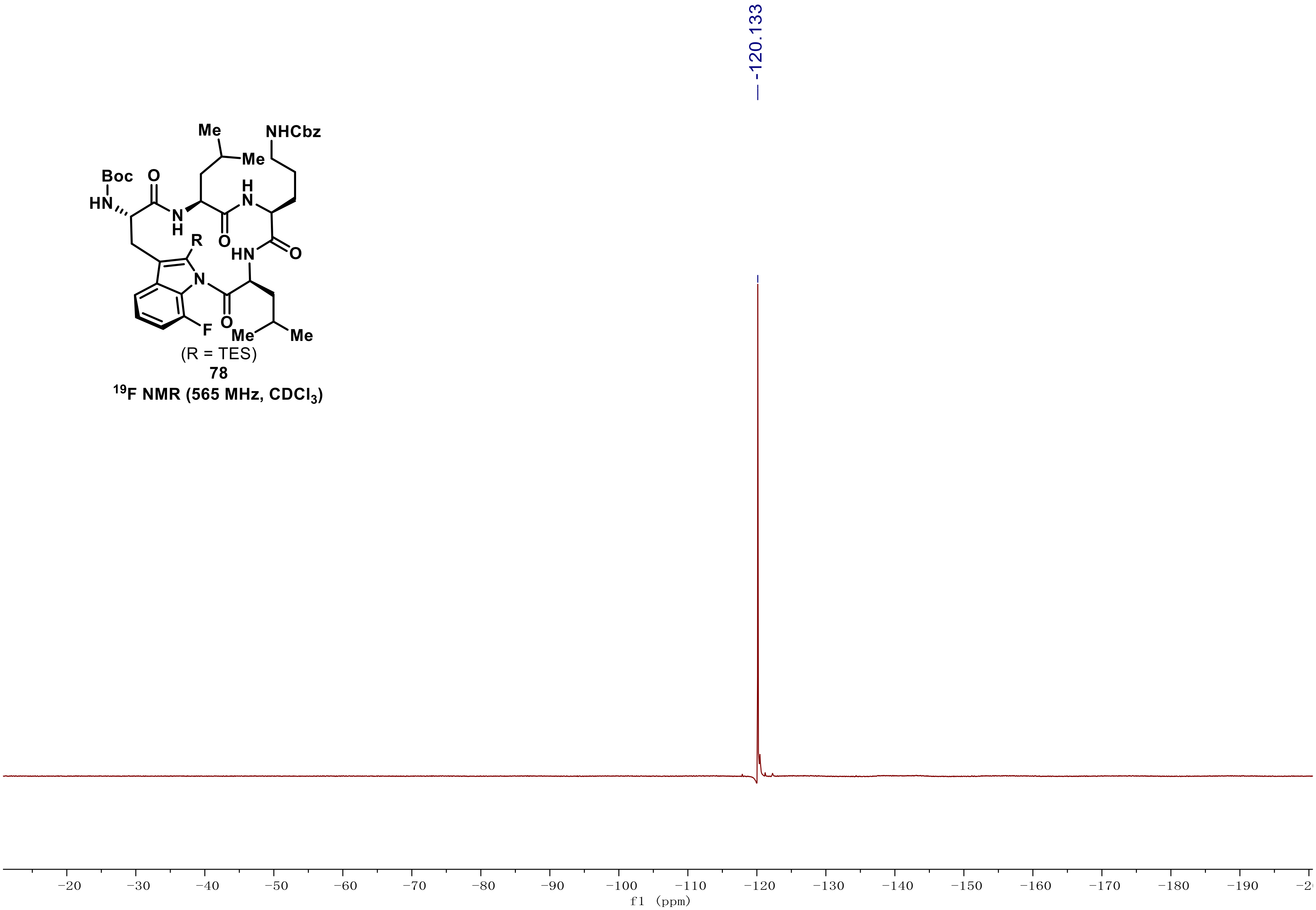

(R = TES)

78

^{19}F NMR (565 MHz, $CDCl_3$)

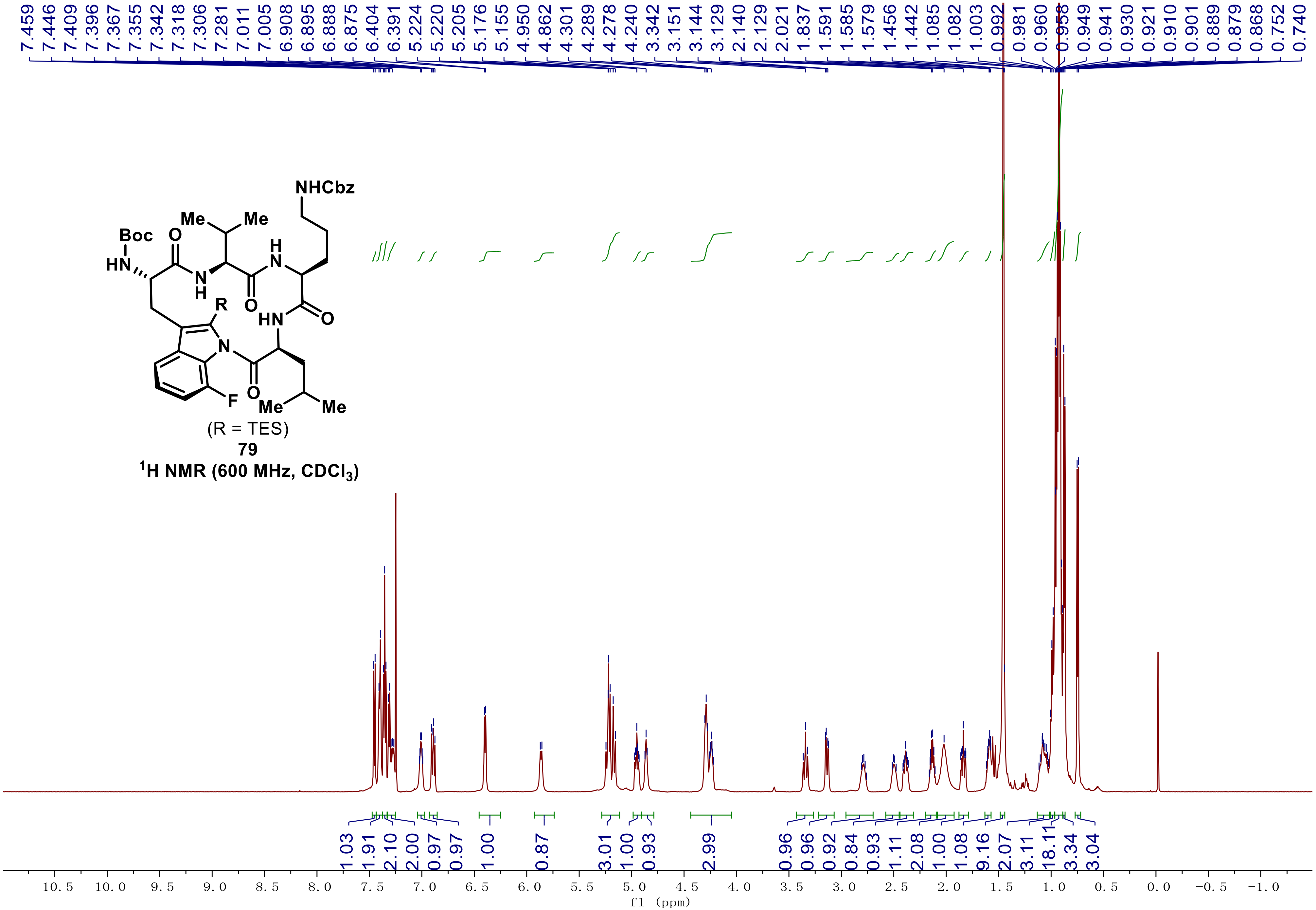

7.459 7.446 7.409 7.396 7.367 7.355 7.342 7.318 7.306 7.281 7.011 7.005 6.908 6.895 6.888 6.875 6.404 6.391 5.224 5.220 5.205 5.176 5.155 4.950 4.862 4.301 4.289 4.278 4.240 3.342 3.151 3.144 3.129 2.140 2.129 2.021 1.837 1.591 1.585 1.579 1.456 1.442 1.085 1.082 1.003 0.992 0.981 0.960 0.958 0.949 0.941 0.930 0.921 0.910 0.901 0.889 0.879 0.868 0.752 0.740
NHCbz
Me
Me
Boc
O
H
N
HN
N
H
R
O
HN
O
N
F
O
Me
Me
(R = TES)
79
^{1}H NMR (600 MHz, CDCl$_3$)
1.03 1.91 2.10 2.00 0.97 0.97 1.00 0.87 3.01 1.00 0.93 2.99 0.96 0.96 0.92 0.84 0.93 1.11 2.08 1.00 1.08 9.16 2.07 3.11 18.11 3.34 3.04
10.5 10.0 9.5 9.0 8.5 8.0 7.5 7.0 6.5 6.0 5.5 5.0 4.5 4.0 3.5 3.0 2.5 2.0 1.5 1.0 0.5 0.0 -0.5 -1.0
f1 (ppm)

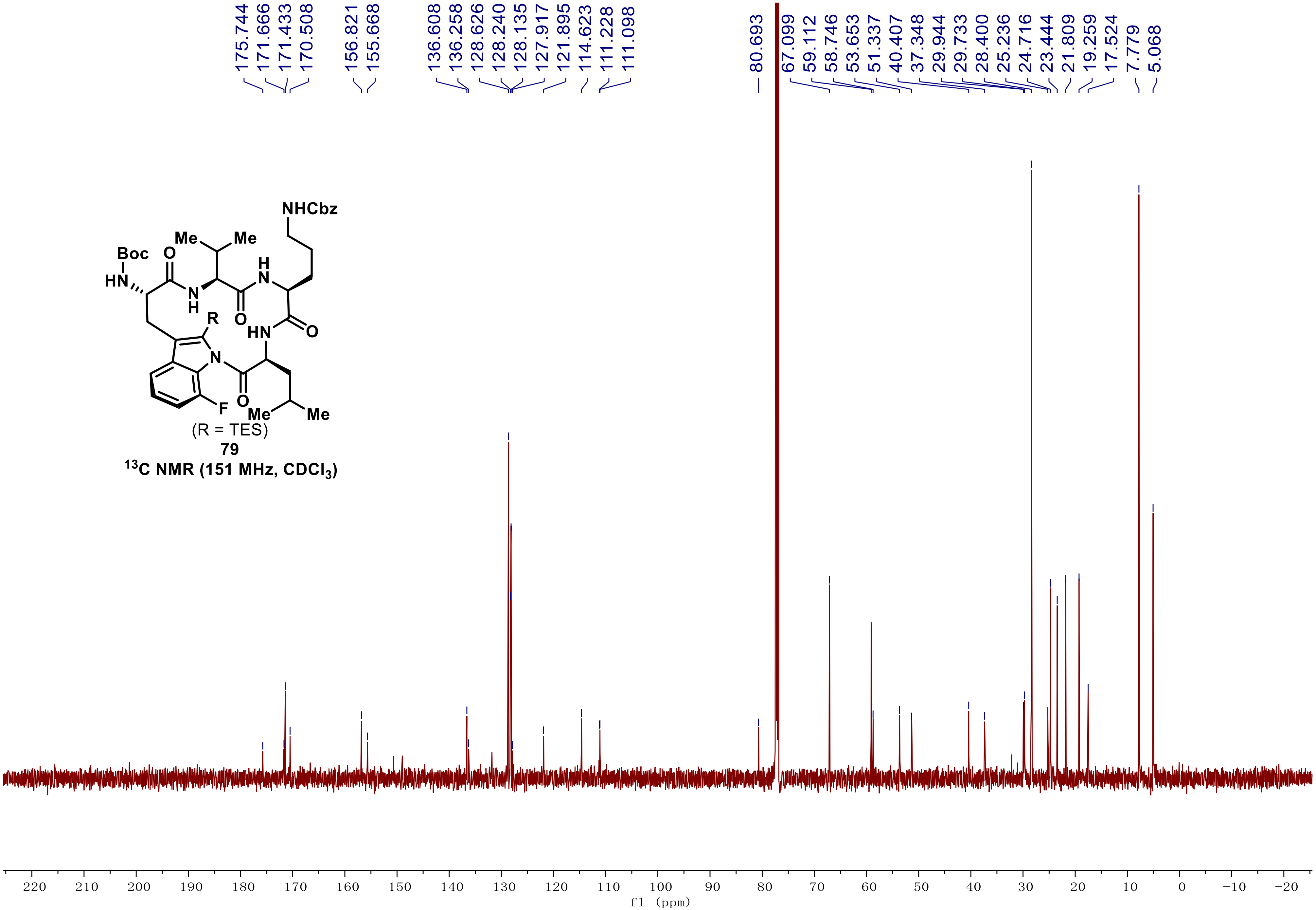

175.744
171.666
171.433
170.508
156.821
155.668
136.608
136.258
128.626
128.240
128.135
127.917
121.895
114.623
111.228
111.098
80.693
67.099
59.112
58.746
53.653
51.337
40.407
37.348
29.944
29.733
28.400
25.236
24.716
23.444
21.809
19.259
17.524
7.779
5.068
NHCbz
Me
Me
Boc
O
HN
N
H
R
O
HN
O
N
F
O
Me
Me
(R = TES)
79
^{13}C NMR (151 MHz, CDCl$_3$)
f1 (ppm)

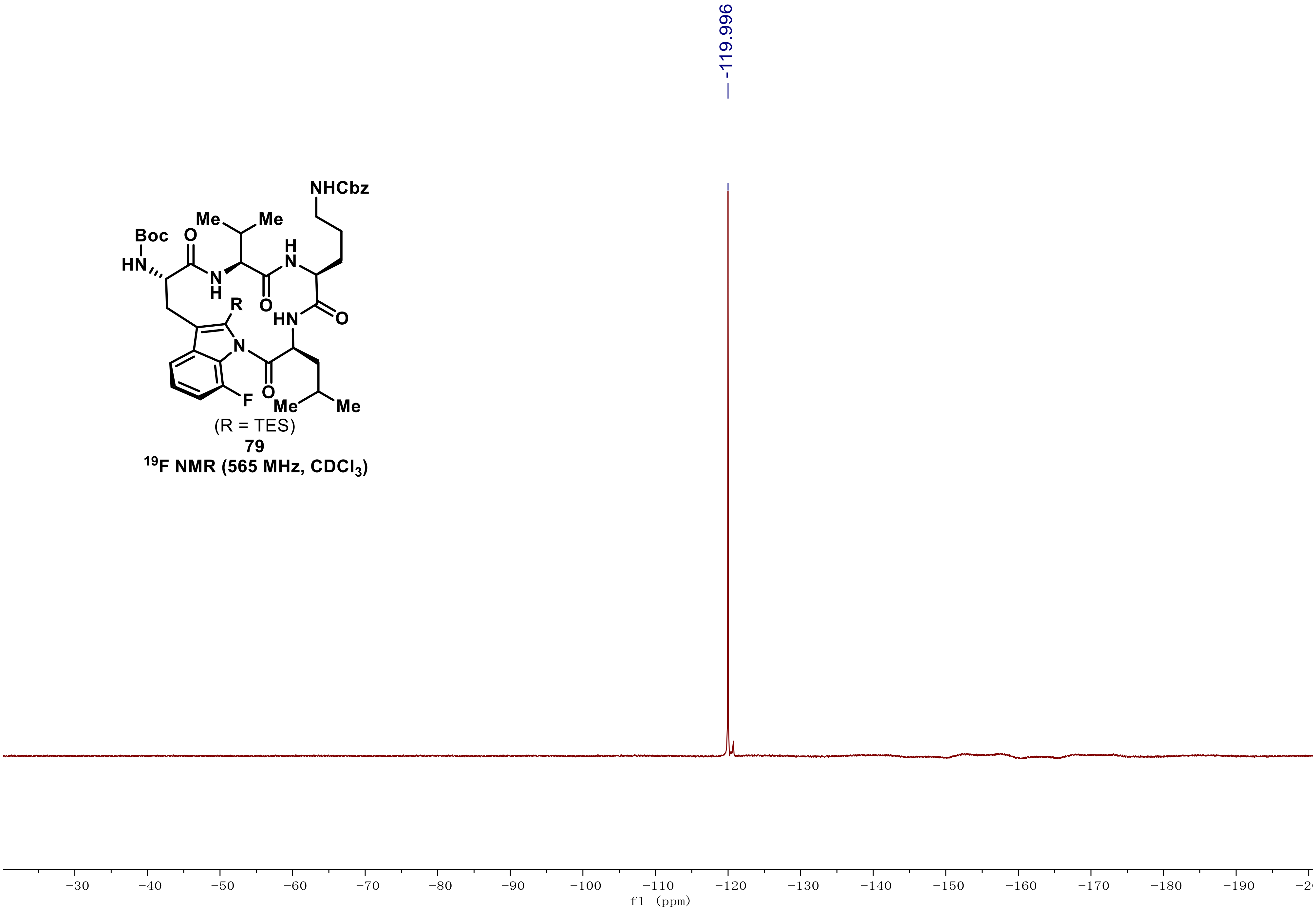

(R = TES)

79

^{19}F NMR (565 MHz, CDCl$_3$)

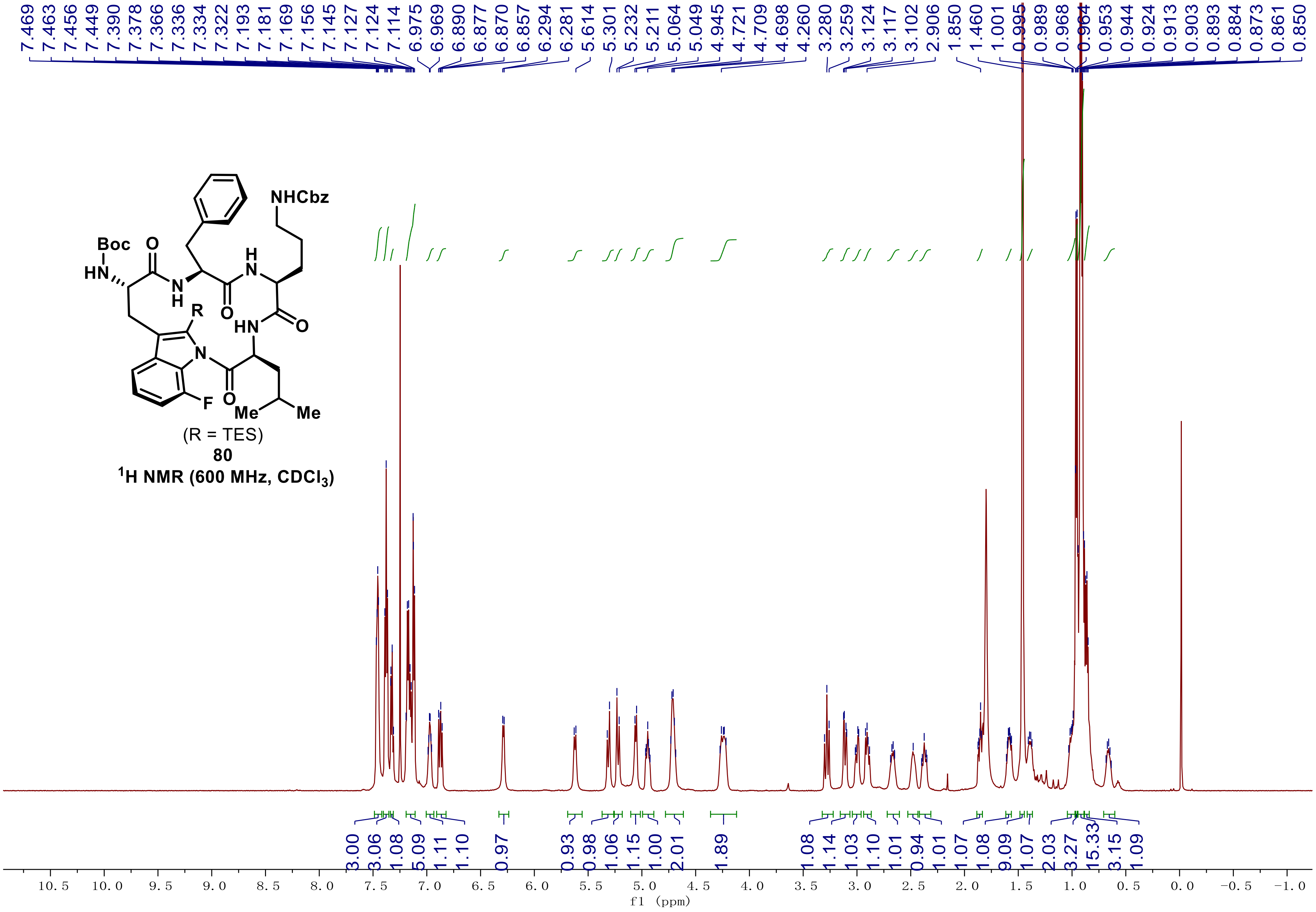

NHCbz
Boc
HN
O
N
H
H
N
R
O
HN
O
N
F
O
Me
Me
(R = TES)
80
1H NMR (600 MHz, CDCl3)
f1 (ppm)

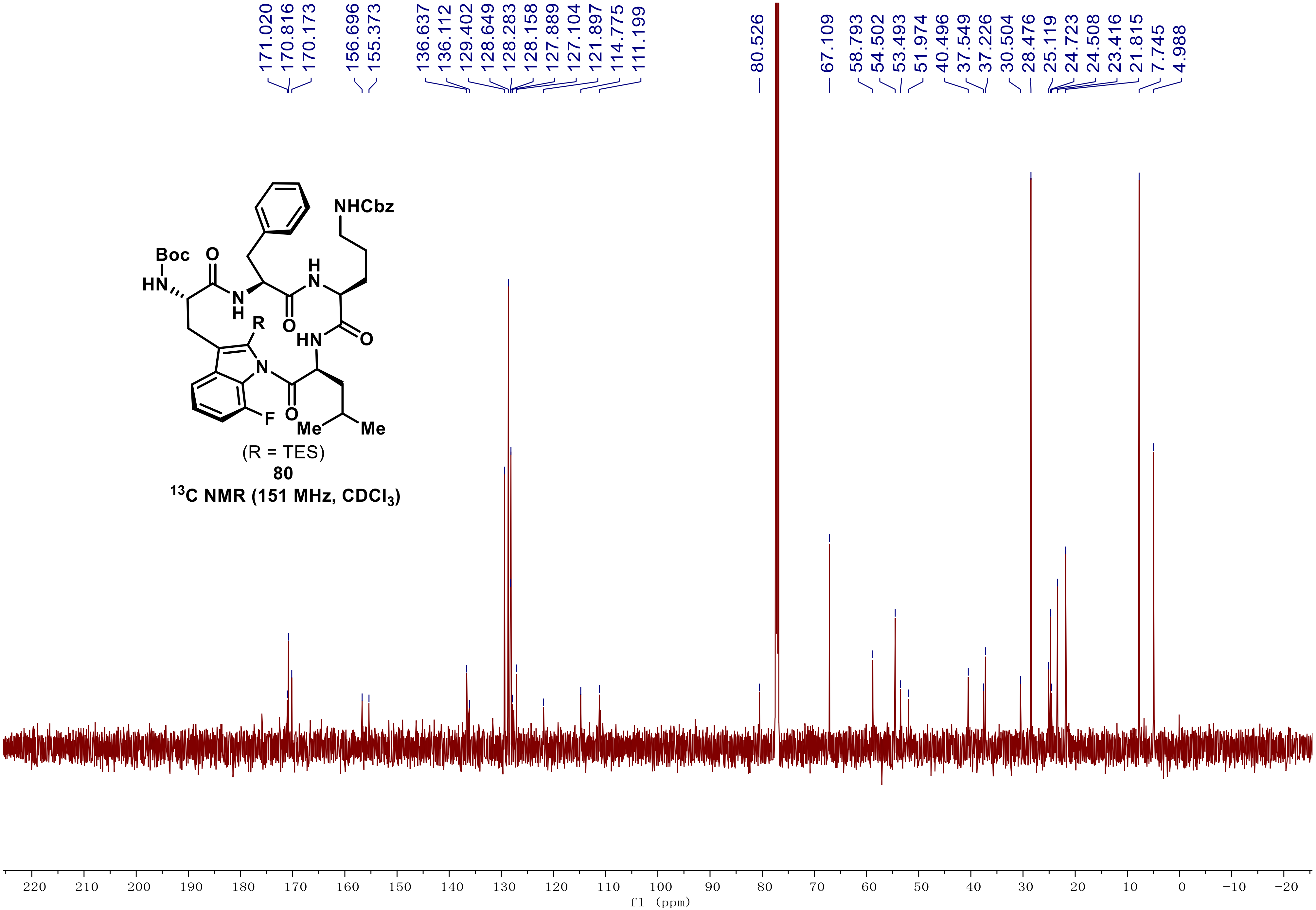

(R = TES)

80

^{13}C NMR (151 MHz, CDCl$_3$)

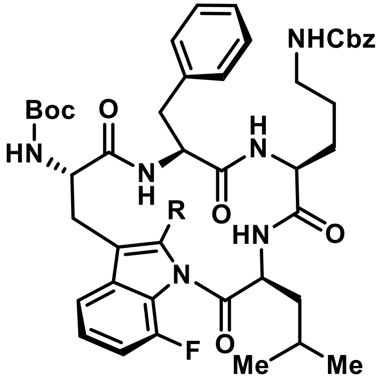

(R = TES)

80

^{19}F NMR (565 MHz, $CDCl_3$)

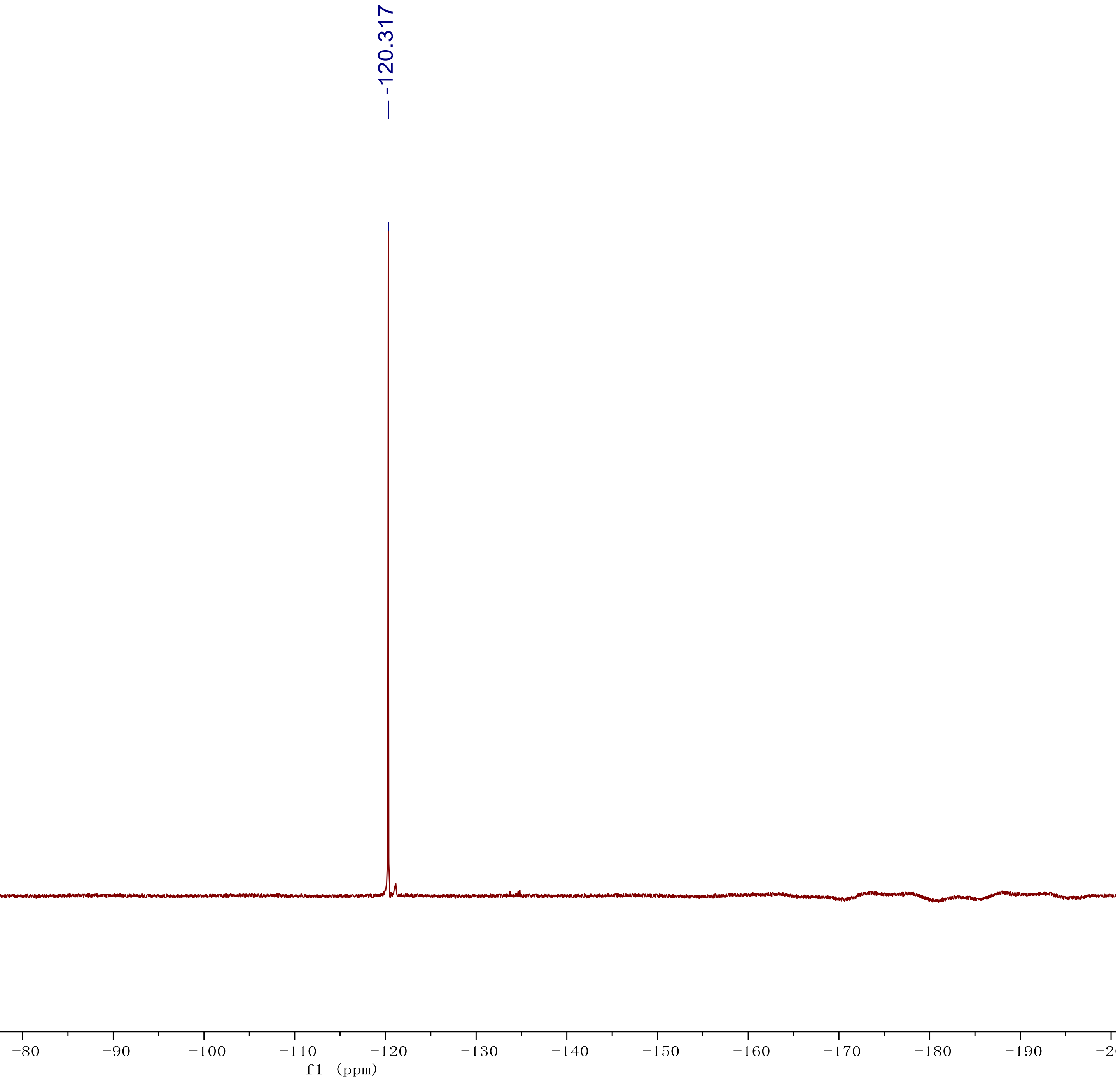

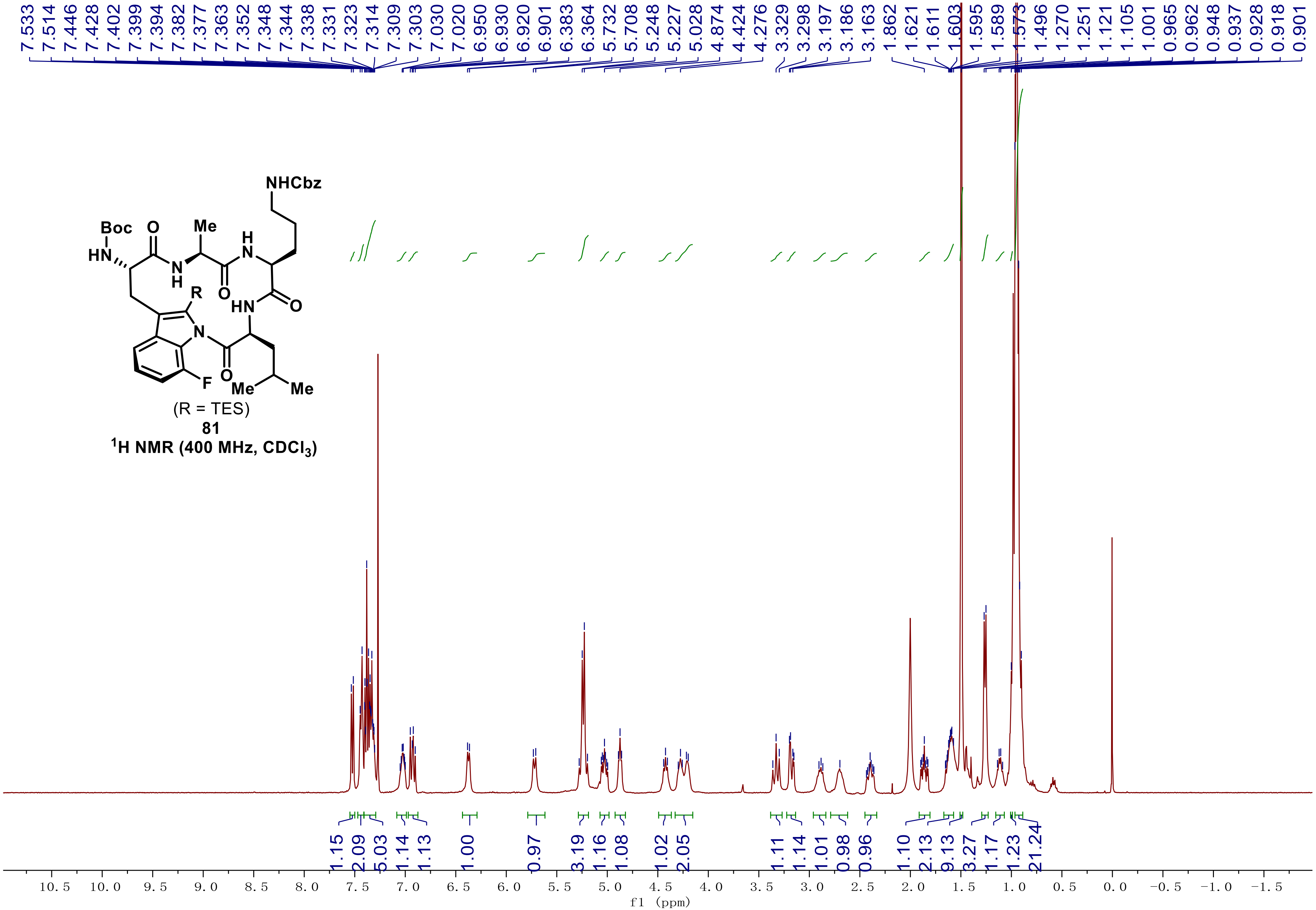
7.533
7.514
7.446
7.428
7.402
7.399
7.394
7.382
7.377
7.363
7.352
7.348
7.344
7.338
7.331
7.323
7.314
7.309
7.303
7.030
7.020
6.950
6.930
6.920
6.901
6.383
6.364
5.732
5.708
5.248
5.227
5.028
4.874
4.424
4.276
3.329
3.298
3.197
3.186
3.163
1.862
1.621
1.611
1.603
1.595
1.589
1.573
1.496
1.270
1.251
1.121
1.105
1.001
0.965
0.962
0.948
0.937
0.928
0.918
0.901
NHCbz
Boc
O
Me
HN
H
N
N
H
R
O
HN
O
N
F
O
Me
Me
(R = TES)
81
^{1}H NMR (400 MHz, $CDCl_3$)
1.15
2.09
5.03
1.14
1.13
1.00
0.97
3.19
1.16
1.08
1.02
2.05
1.11
1.14
1.01
0.98
0.96
1.10
2.13
9.13
3.27
1.17
1.23
21.24
10.5
10.0
9.5
9.0
8.5
8.0
7.5
7.0
6.5
6.0
5.5
5.0
4.5
4.0
3.5
3.0
2.5
2.0
1.5
1.0
0.5
0.0
-0.5
-1.0
-1.5
f1 (ppm)

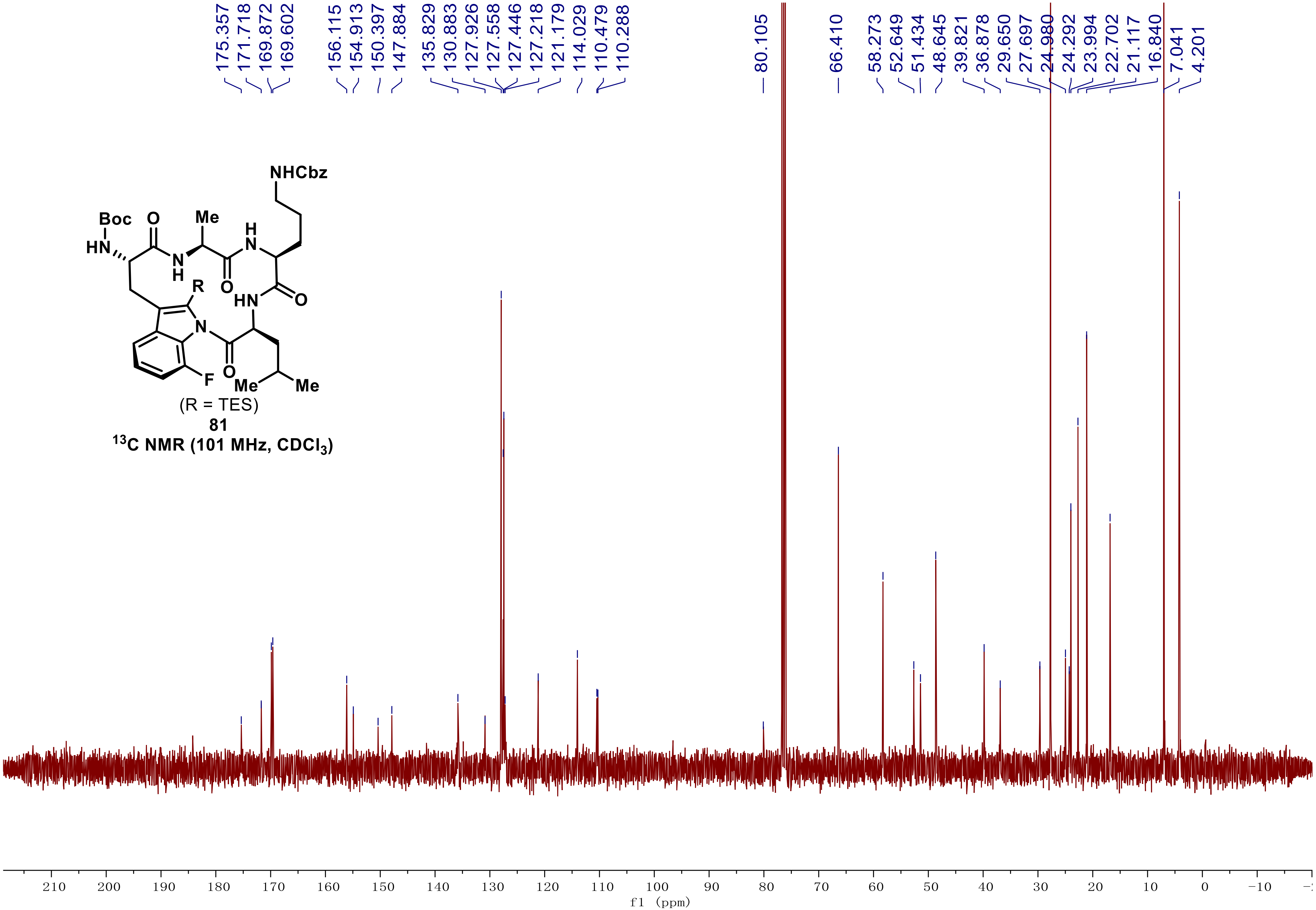
175.357
171.718
169.872
169.602
156.115
154.913
150.397
147.884
135.829
130.883
127.926
127.558
127.446
127.218
121.179
114.029
110.479
110.288
80.105
66.410
58.273
52.649
51.434
48.645
39.821
36.878
29.650
27.697
24.980
24.292
23.994
22.702
21.117
16.840
7.041
4.201
NHCbz
Boc
HN
O
Me
H
N
N
H
O
R
HN
O
N
O
F
Me
Me
(R = TES)
81
^{13}C NMR (101 MHz, CDCl$_3$)
210
200
190
180
170
160
150
140
130
120
110
100
90
80
70
60
50
40
30
20
10
0
−10
f1 (ppm)

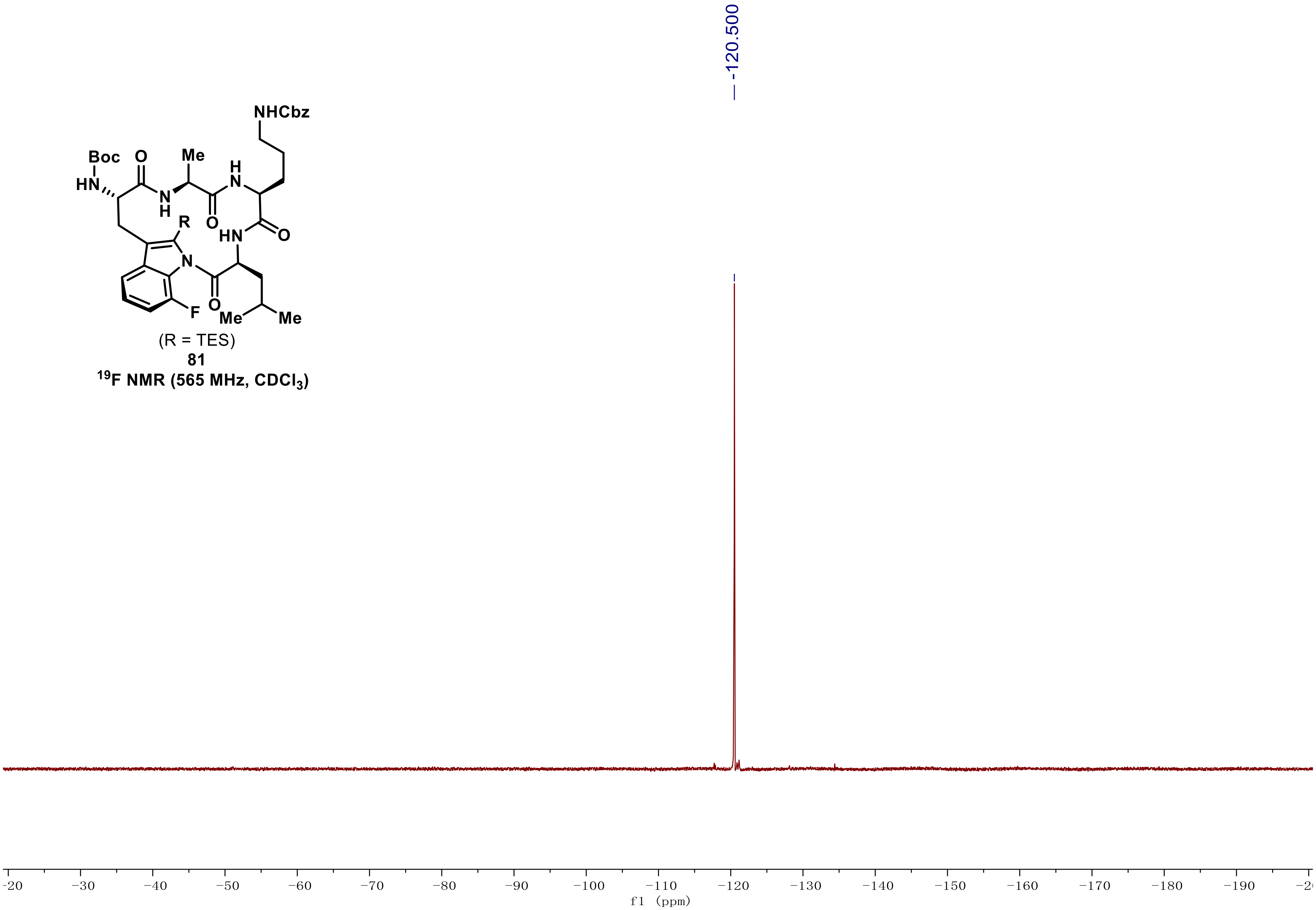

NHCbz
Boc
O
Me
H
N
HN
N
H
R
O
HN
O
N
F
O
Me
Me
(R = TES)
81
19F NMR (565 MHz, CDCl3)
-120.500
-20
-30
-40
-50
-60
-70
-80
-90
-100
-110
-120
-130
-140
-150
-160
-170
-180
-190
f1 (ppm)

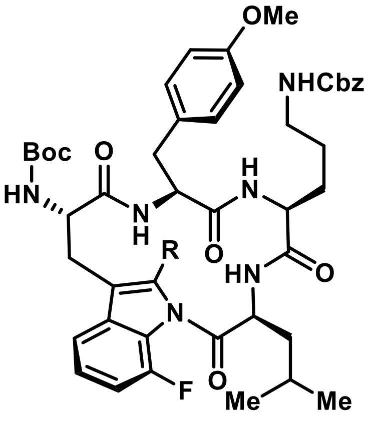

(R = TES)

82

^{1}H NMR (400 MHz, $CDCl_3$)

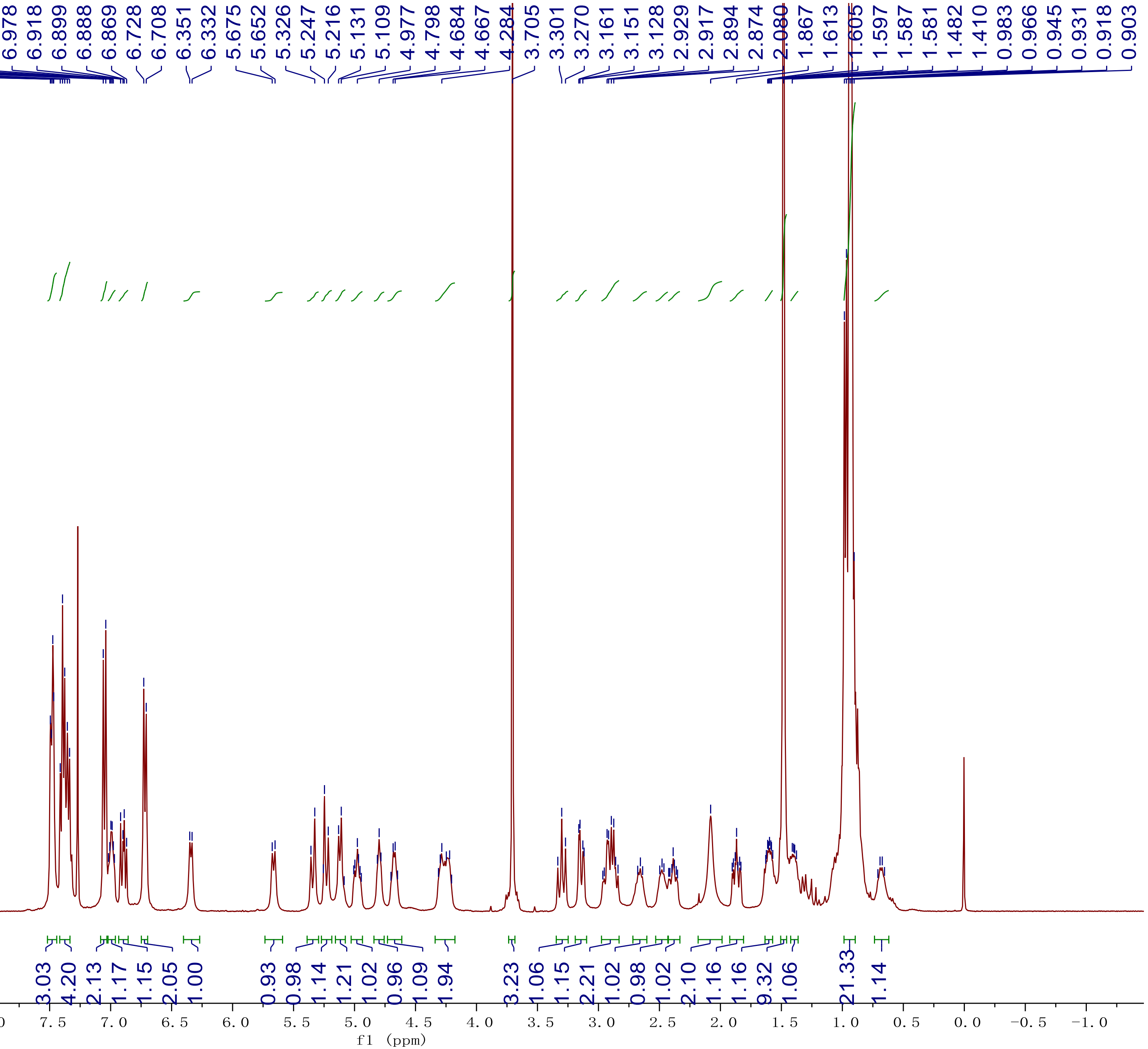

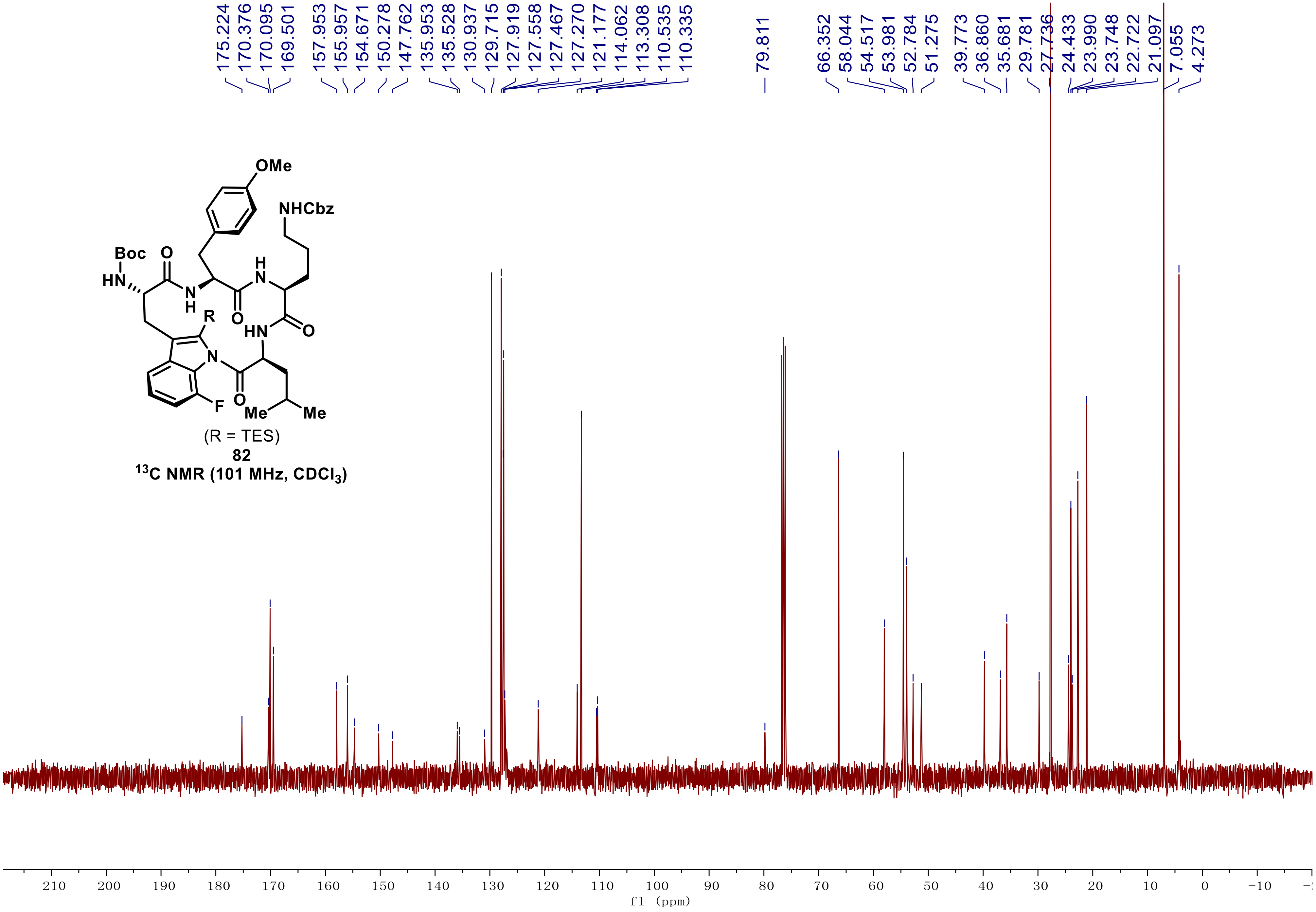
175.224
170.376
170.095
169.501
157.953
155.957
154.671
150.278
147.762
135.953
135.528
130.937
129.715
127.919
127.558
127.467
127.270
121.177
114.062
113.308
110.535
110.335
79.811
66.352
58.044
54.517
53.981
52.784
51.275
39.773
36.860
35.681
29.781
27.736
24.433
23.990
23.748
22.722
21.097
7.055
4.273
OMe
NHCbz
Boc
HN
O
N
H
R
N
F
O
HN
O
Me
Me
(R = TES)
82
^{13}C NMR (101 MHz, CDCl$_3$)
210 200 190 180 170 160 150 140 130 120 110 100 90 80 70 60 50 40 30 20 10 0 −10
f1 (ppm)

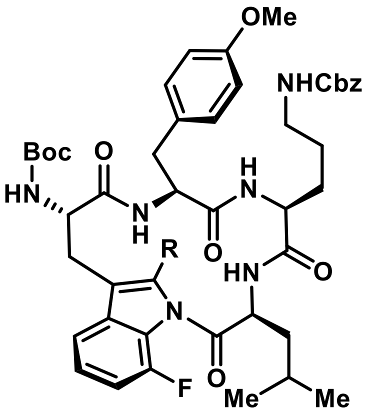

(R = TES)

82

^{19}F NMR (565 MHz, CDCl$_3$)

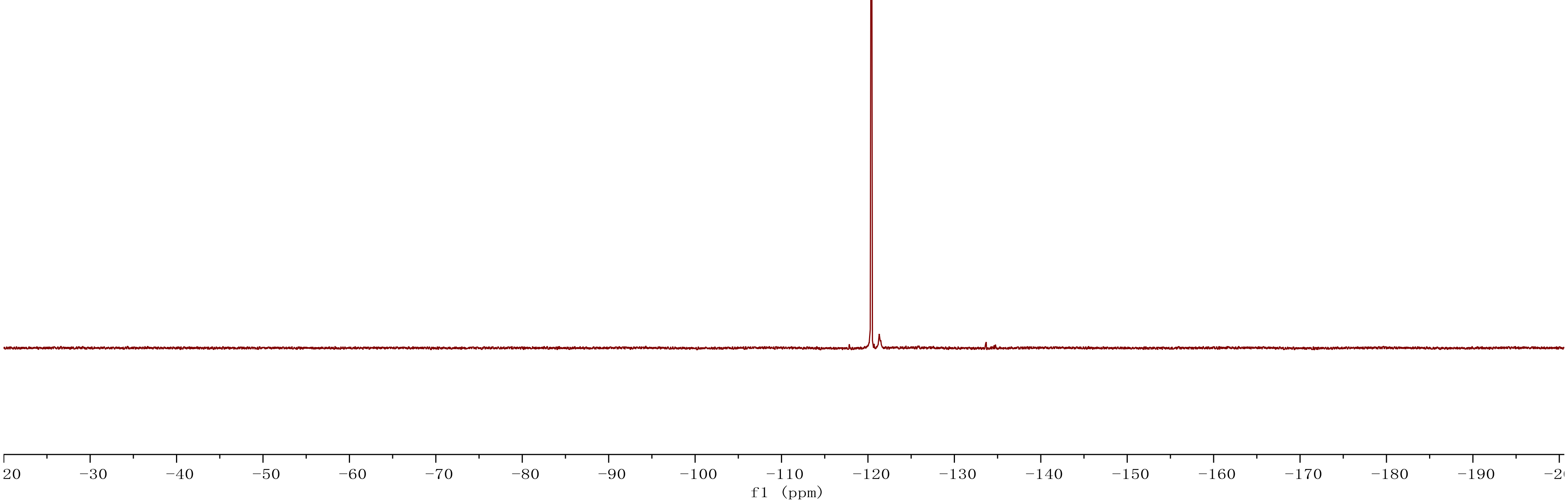

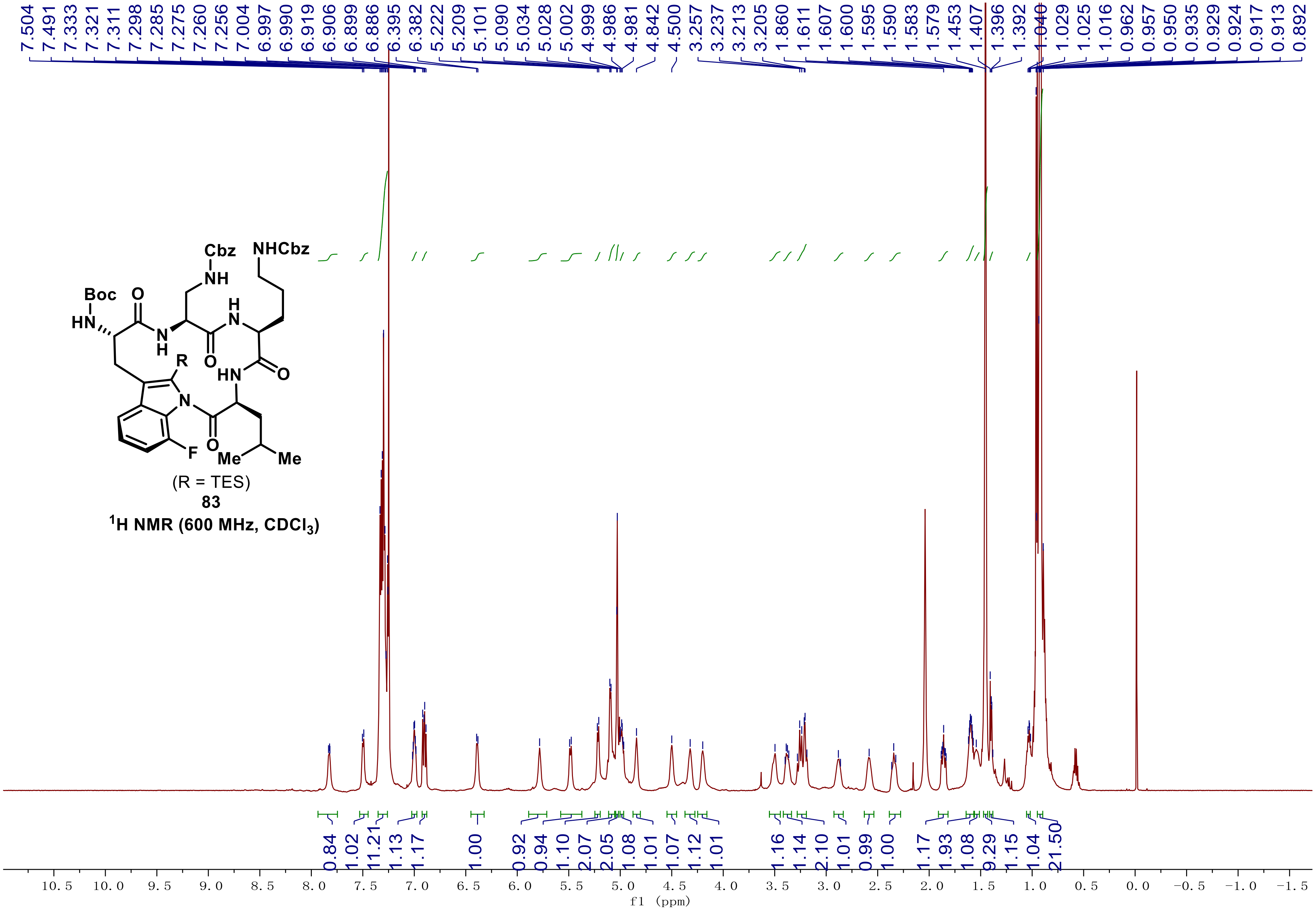

7.504
7.491
7.333
7.321
7.311
7.298
7.285
7.275
7.260
7.256
7.004
6.997
6.990
6.919
6.906
6.899
6.886
6.395
6.382
5.222
5.209
5.101
5.090
5.034
5.028
5.002
4.999
4.986
4.981
4.842
4.500
3.257
3.237
3.213
3.205
1.860
1.611
1.607
1.600
1.595
1.590
1.583
1.579
1.453
1.407
1.396
1.392
1.040
1.029
1.025
1.016
0.962
0.957
0.950
0.935
0.929
0.924
0.917
0.913
0.892
Cbz
NH
NHCbz
Boc
HN
O
H
N
R
HN
O
F
Me
Me
(R = TES)
83
^{1}H NMR (600 MHz, CDCl$_3$)
0.84
1.02
11.21
1.13
1.17
1.00
0.92
0.94
1.10
2.07
2.05
1.08
1.01
1.07
1.12
1.01
1.16
1.14
2.10
1.01
0.99
1.00
1.17
1.93
1.08
9.29
1.15
1.04
21.50
f1 (ppm)

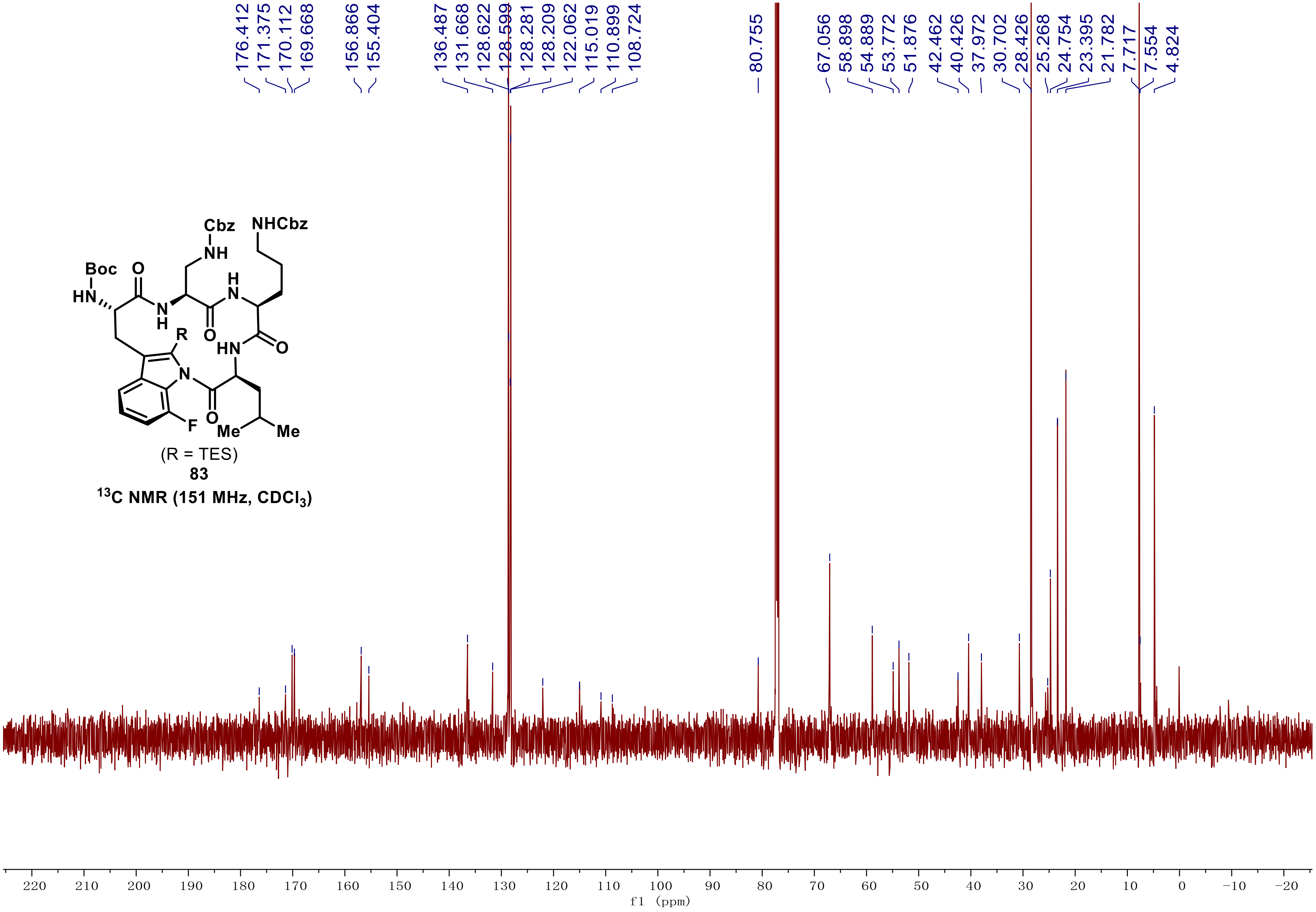

(R = TES)

83

^{13}C NMR (151 MHz, $CDCl_3$)

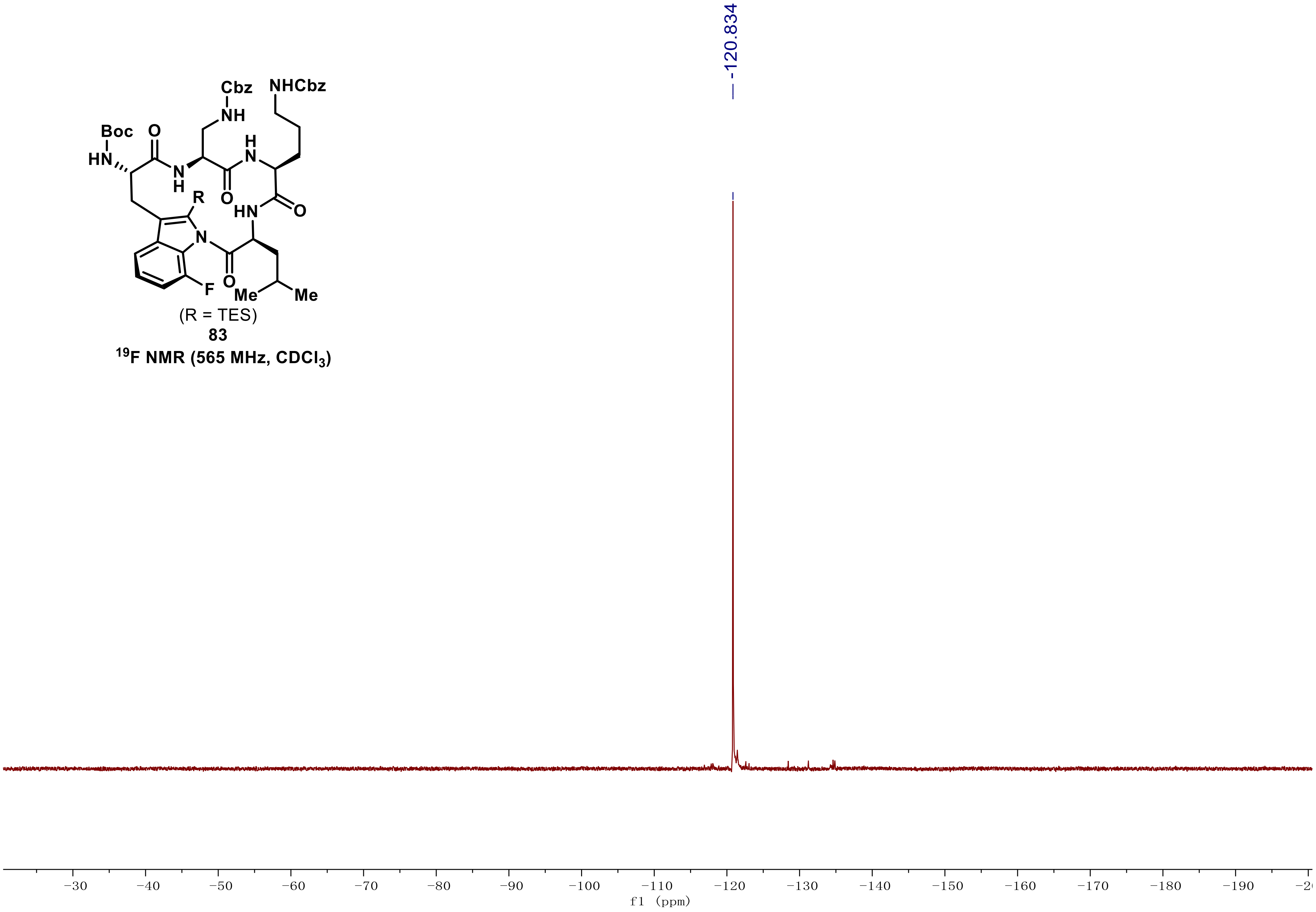

Boc
HN
O
N
H
Cbz
NH
H
N
O
NHCbz
R
HN
O
N
O
F
Me
Me
(R = TES)
83
19F NMR (565 MHz, CDCl3)
-120.834
f1 (ppm)

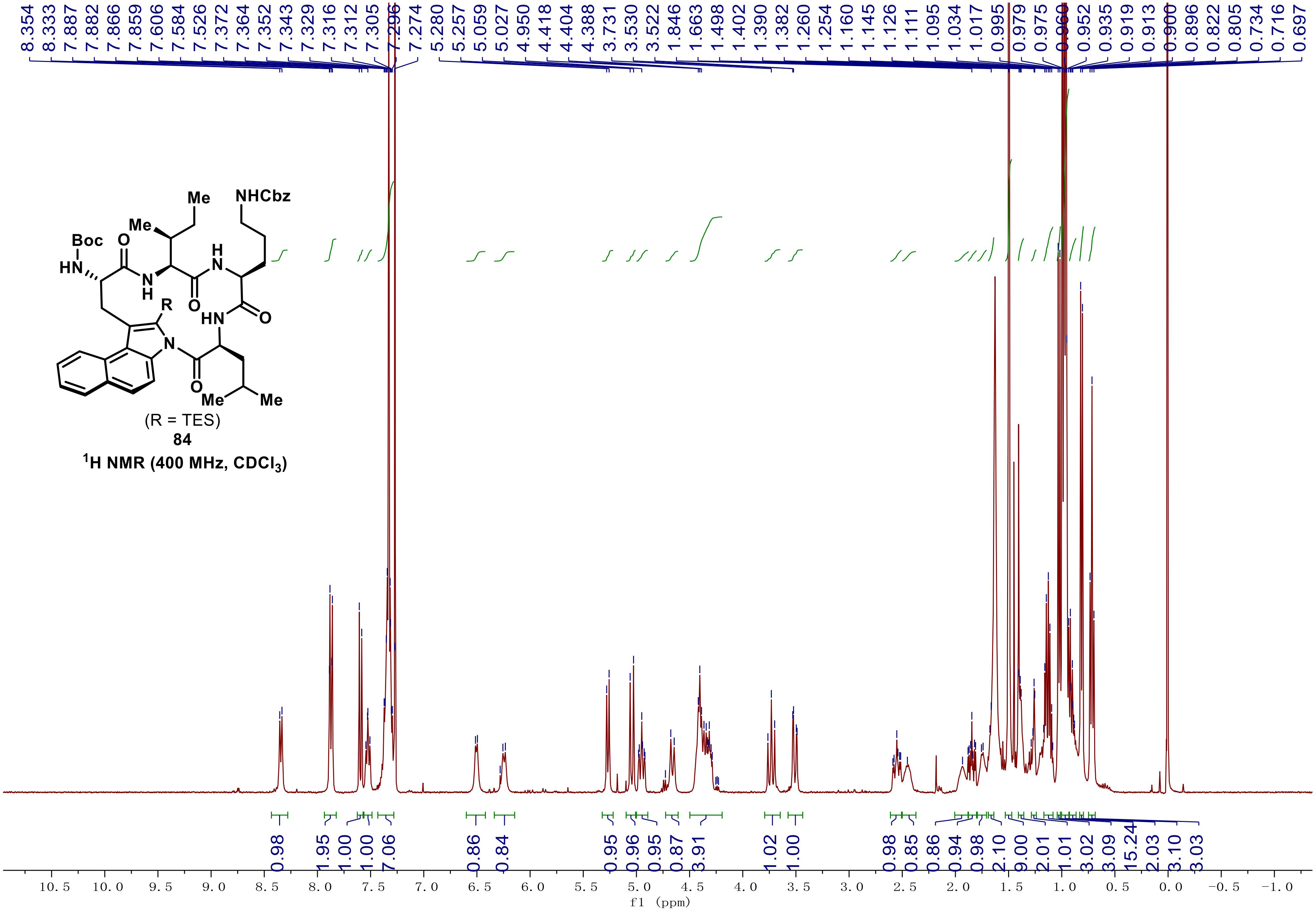

(R = TES)
84
^{1}H NMR (400 MHz, $CDCl_3$)

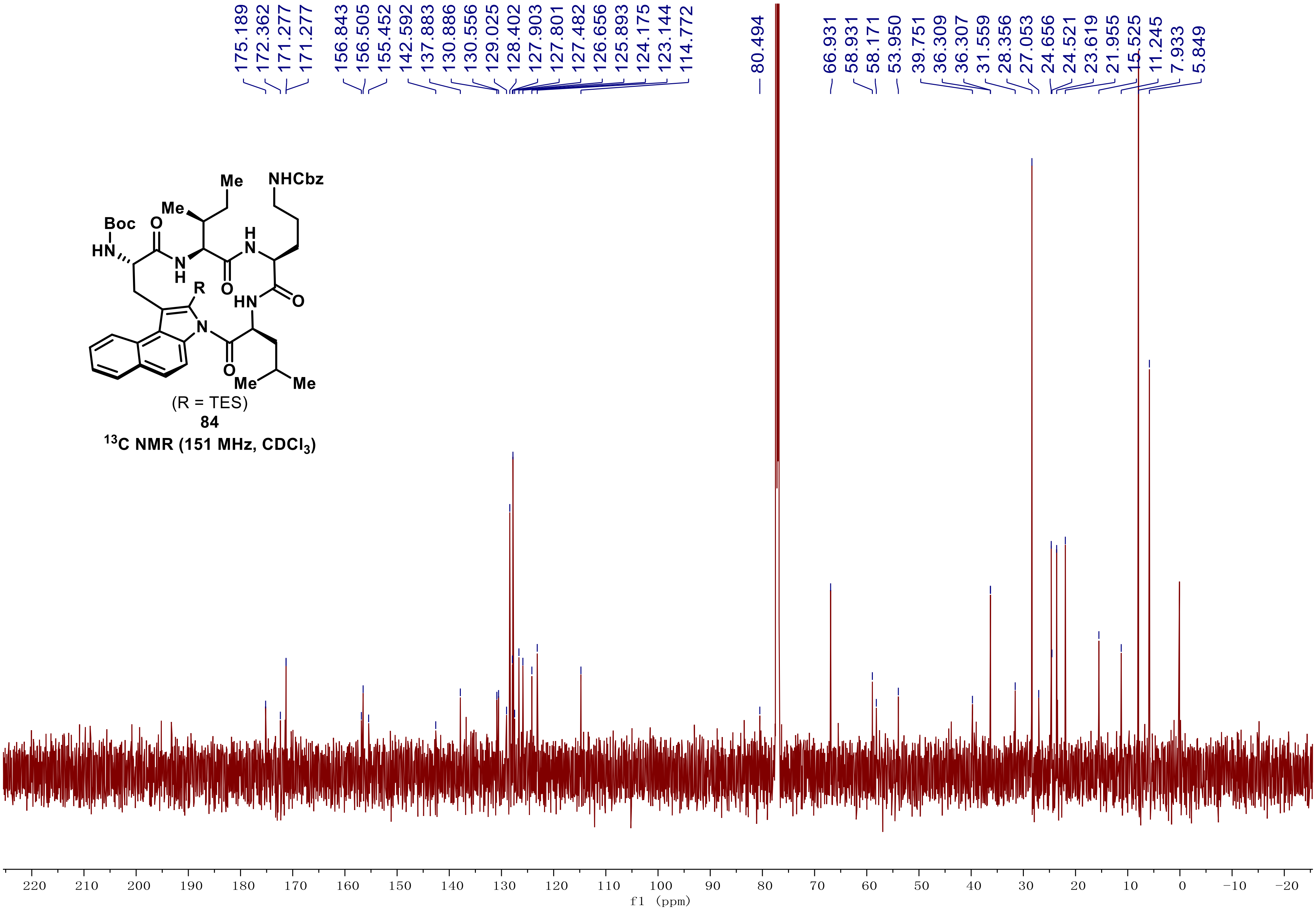

(R = TES)

84

^{13}C NMR (151 MHz, CDCl$_3$)

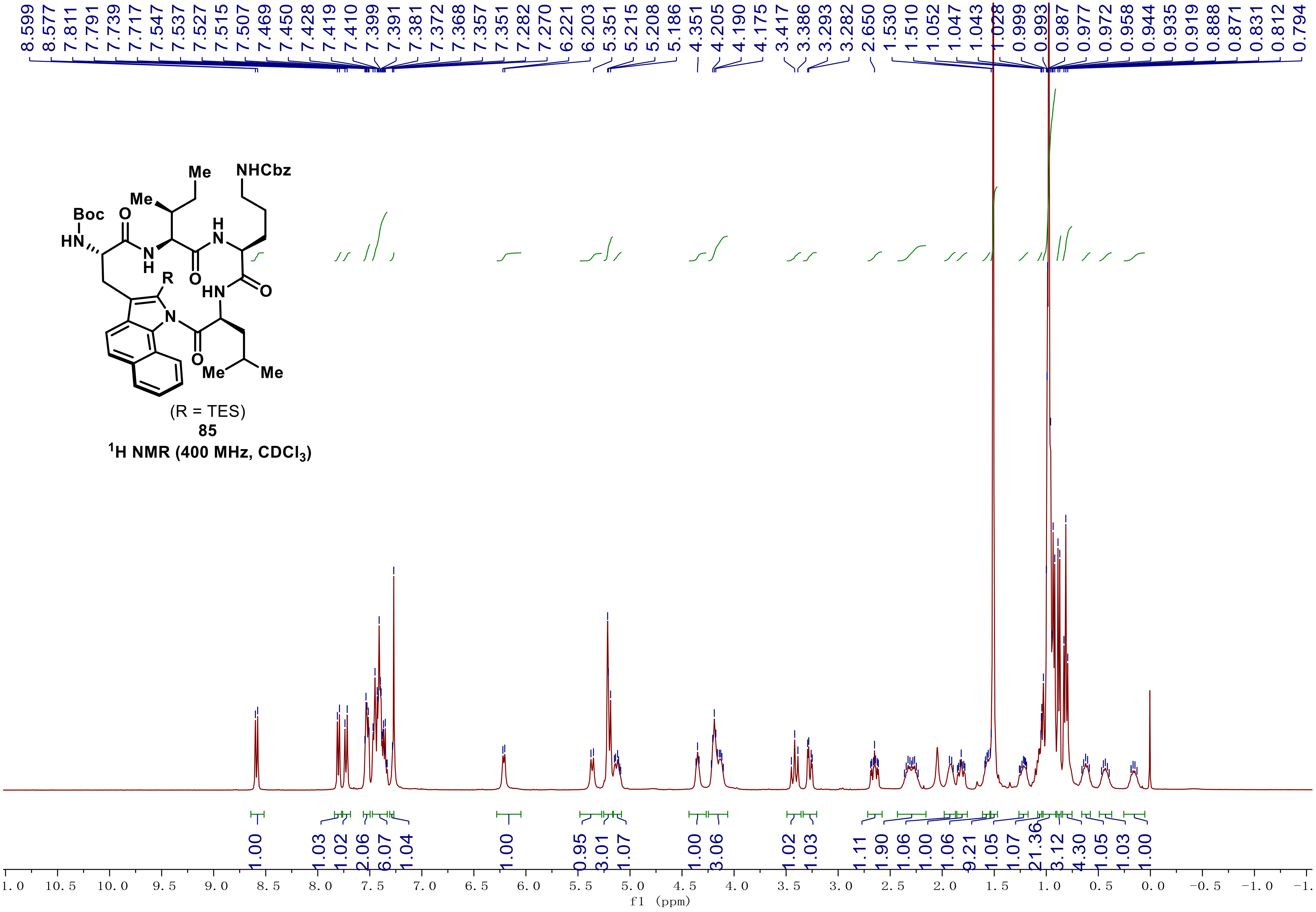

Boc
NHCbz
(R = TES)
85
¹H NMR (400 MHz, CDCl₃)
f1 (ppm)

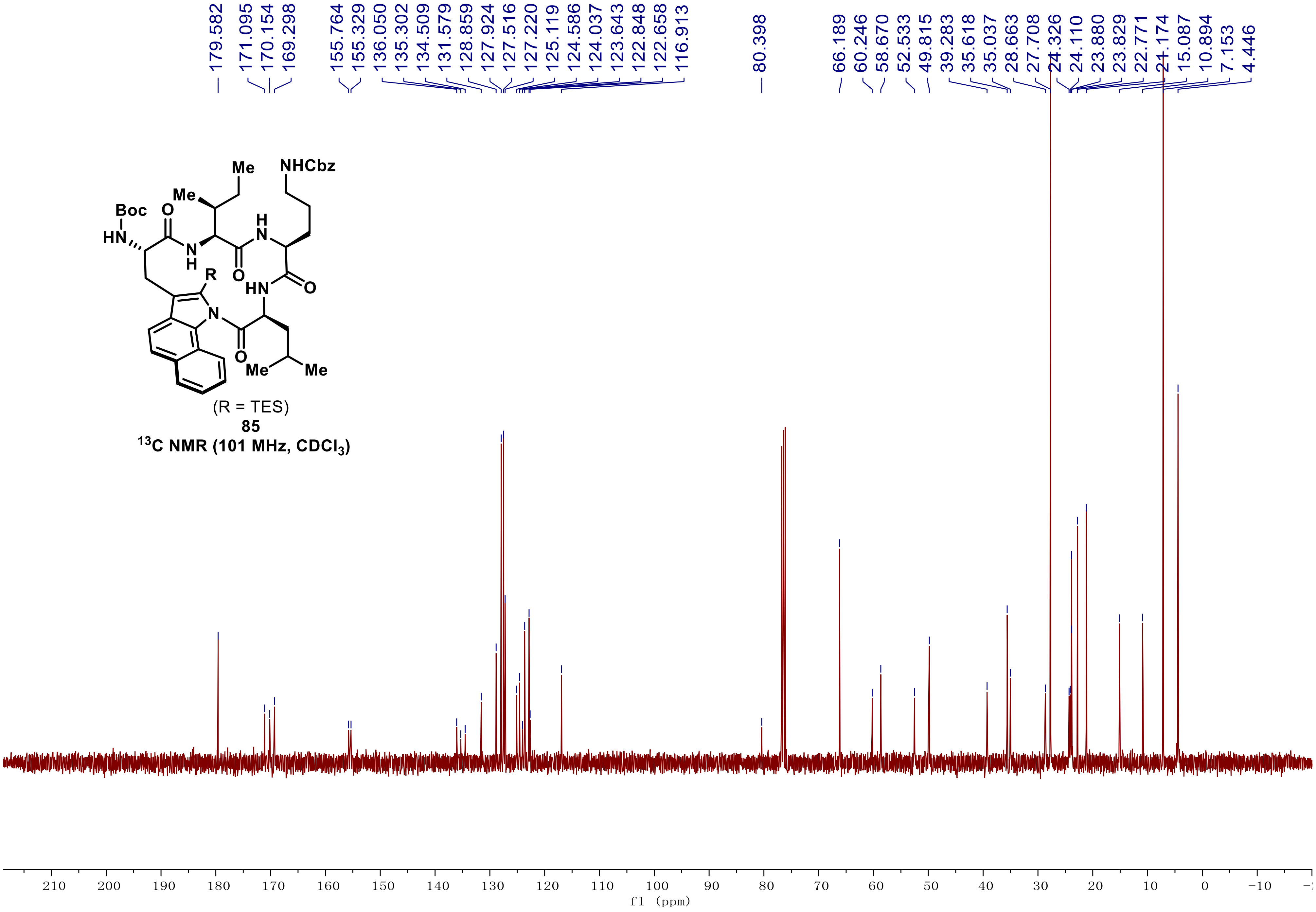

(R = TES)

85

^{13}C NMR (101 MHz, $CDCl_3$)

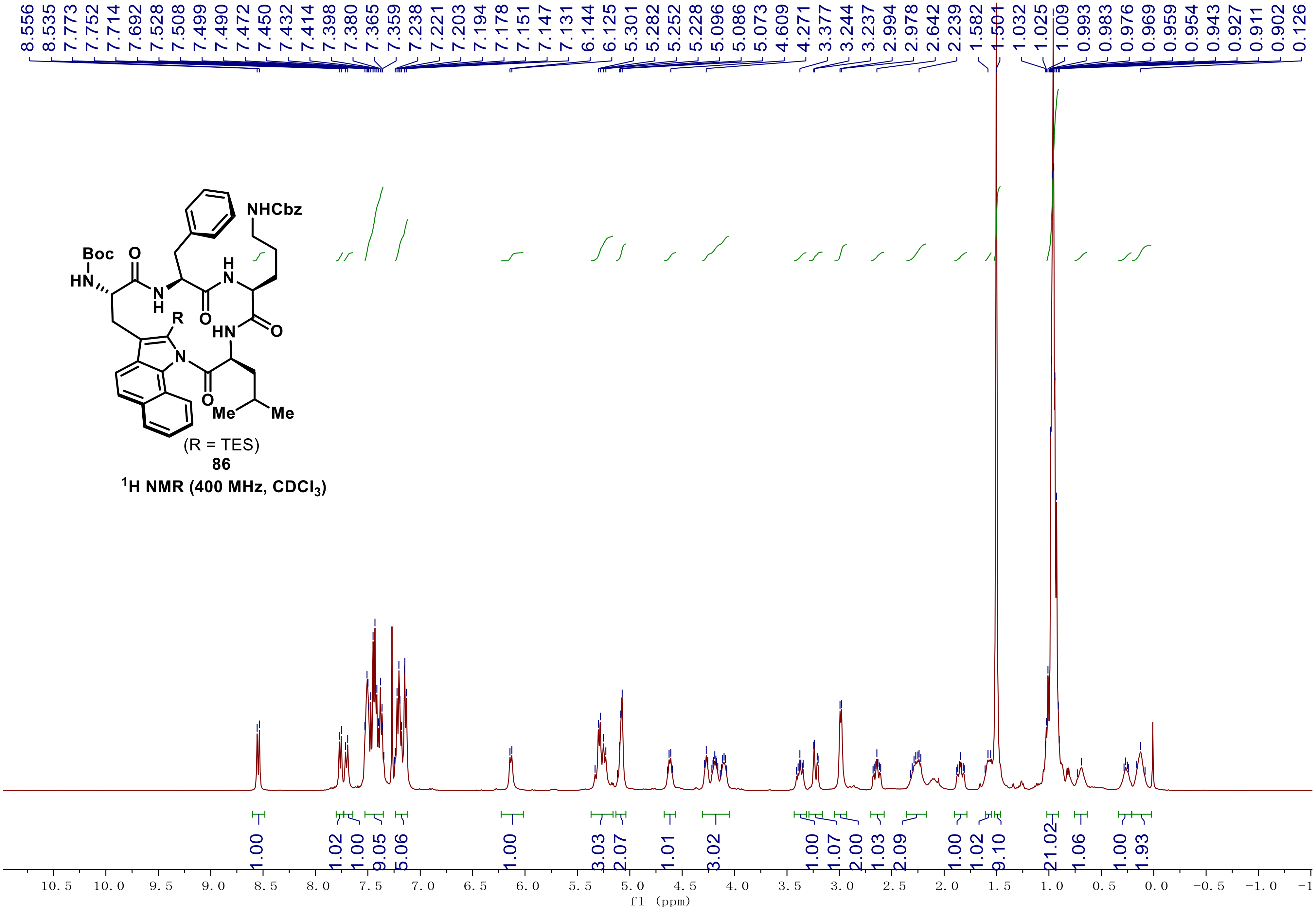
8.556 8.535 7.773 7.752 7.714 7.692 7.528 7.508 7.499 7.490 7.472 7.450 7.432 7.414 7.398 7.380 7.365 7.359 7.238 7.221 7.203 7.194 7.178 7.151 7.147 7.131 6.144 6.125 5.301 5.282 5.252 5.228 5.096 5.086 5.073 4.609 4.271 3.377 3.244 3.237 2.994 2.978 2.642 2.239 1.582 1.501 1.032 1.025 1.009 0.993 0.983 0.976 0.969 0.959 0.954 0.943 0.927 0.911 0.902 0.126
NHCbz
Boc
HN
O
N
H
R
O
HN
O
N
Me
Me
O
(R = TES)
86
^{1}H NMR (400 MHz, CDCl$_3$)
1.00 1.02 1.00 9.05 5.06 1.00 3.03 2.07 1.01 3.02 1.00 1.07 2.00 1.03 2.09 1.00 1.02 9.10 21.02 1.06 1.00 1.93
10.5 10.0 9.5 9.0 8.5 8.0 7.5 7.0 6.5 6.0 5.5 5.0 4.5 4.0 3.5 3.0 2.5 2.0 1.5 1.0 0.5 0.0 −0.5 −1.0
f1 (ppm)

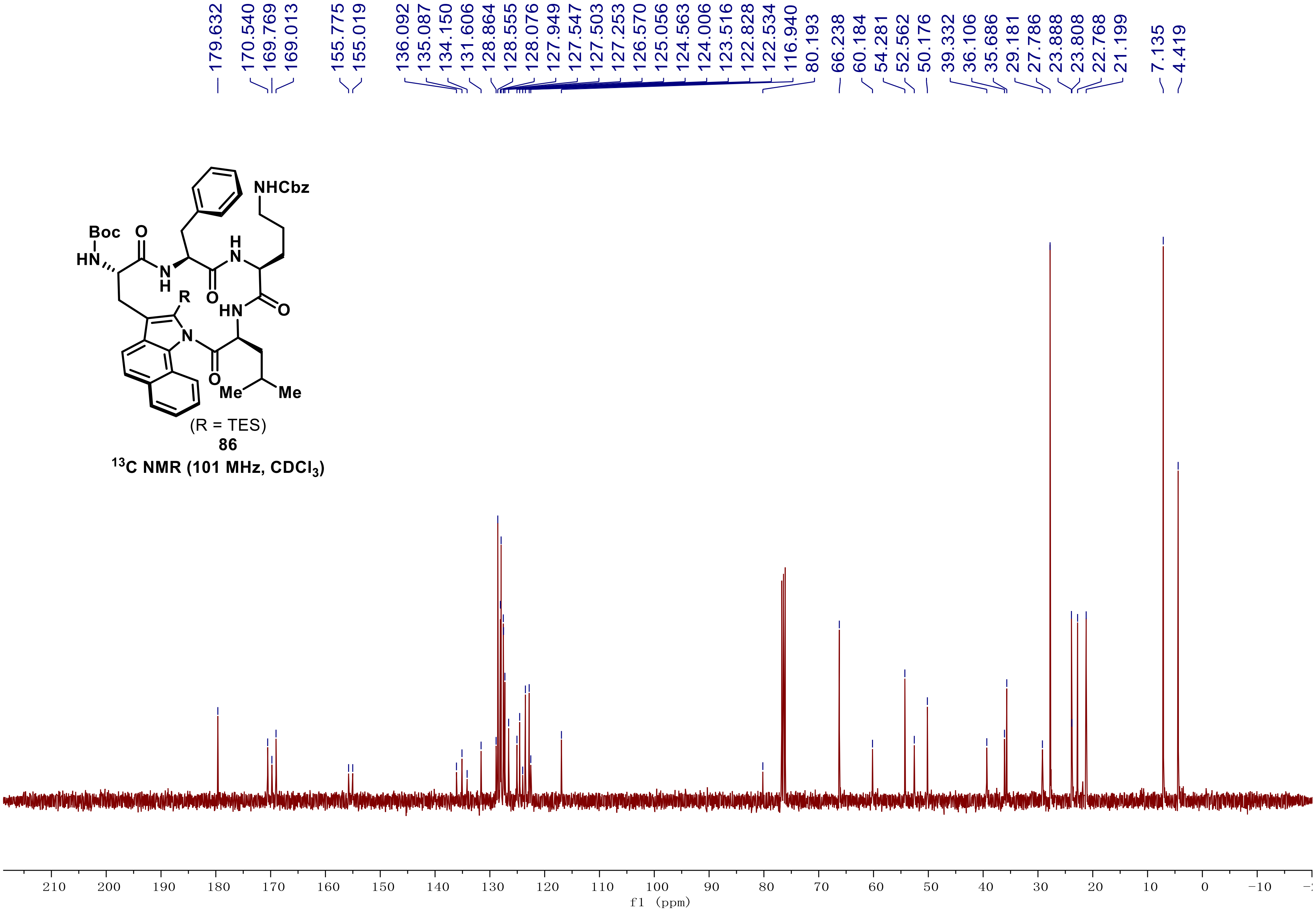
179.632
170.540
169.769
169.013
155.775
155.019
136.092
135.087
134.150
131.606
128.864
128.555
128.076
127.949
127.547
127.503
127.253
126.570
125.056
124.563
124.006
123.516
122.828
122.534
116.940
80.193
66.238
60.184
54.281
52.562
50.176
39.332
36.106
35.686
29.181
27.786
23.888
23.808
22.768
21.199
7.135
4.419
NHCbz
Boc
O
HN
H
N
R
N
O
HN
O
N
O
Me
Me
(R = TES)
86
^{13}C NMR (101 MHz, CDCl$_3$)
f1 (ppm)

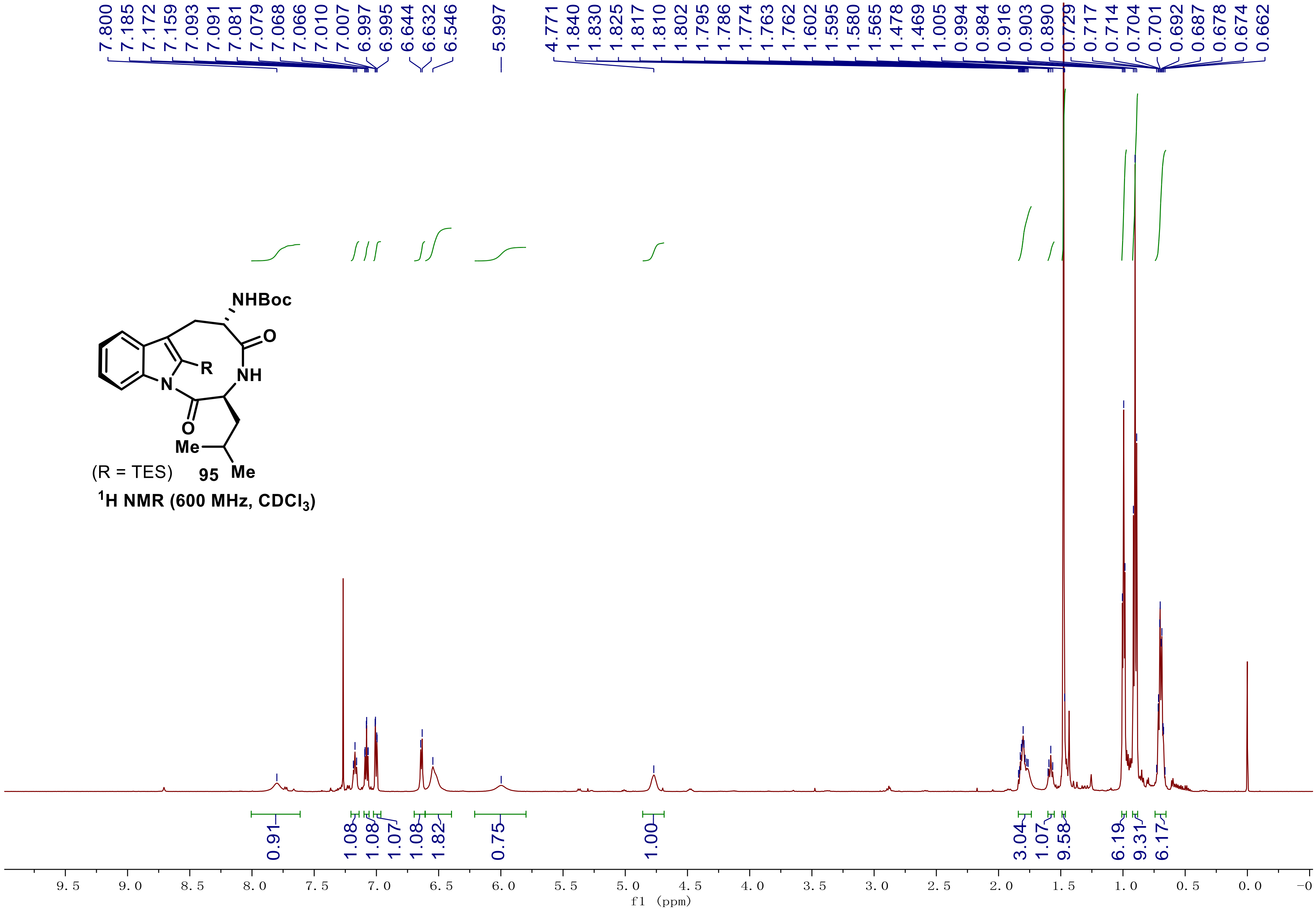
7.800
7.185
7.172
7.159
7.093
7.091
7.081
7.079
7.068
7.066
7.010
7.007
6.997
6.995
6.644
6.632
6.546
5.997
4.771
1.840
1.830
1.825
1.817
1.810
1.802
1.795
1.786
1.774
1.763
1.762
1.602
1.595
1.580
1.565
1.478
1.469
1.005
0.994
0.984
0.916
0.903
0.890
0.729
0.717
0.714
0.704
0.701
0.692
0.687
0.678
0.674
0.662
NHBoc
O
R
NH
N
O
Me
(R = TES)
95
Me
^{1}H NMR (600 MHz, $CDCl_3$)
0.91
1.08
1.08
1.07
1.08
1.82
0.75
1.00
3.04
1.07
9.58
6.19
9.31
6.17
9.5
9.0
8.5
8.0
7.5
7.0
6.5
6.0
5.5
5.0
4.5
4.0
3.5
3.0
2.5
2.0
1.5
1.0
0.5
0.0
f1 (ppm)

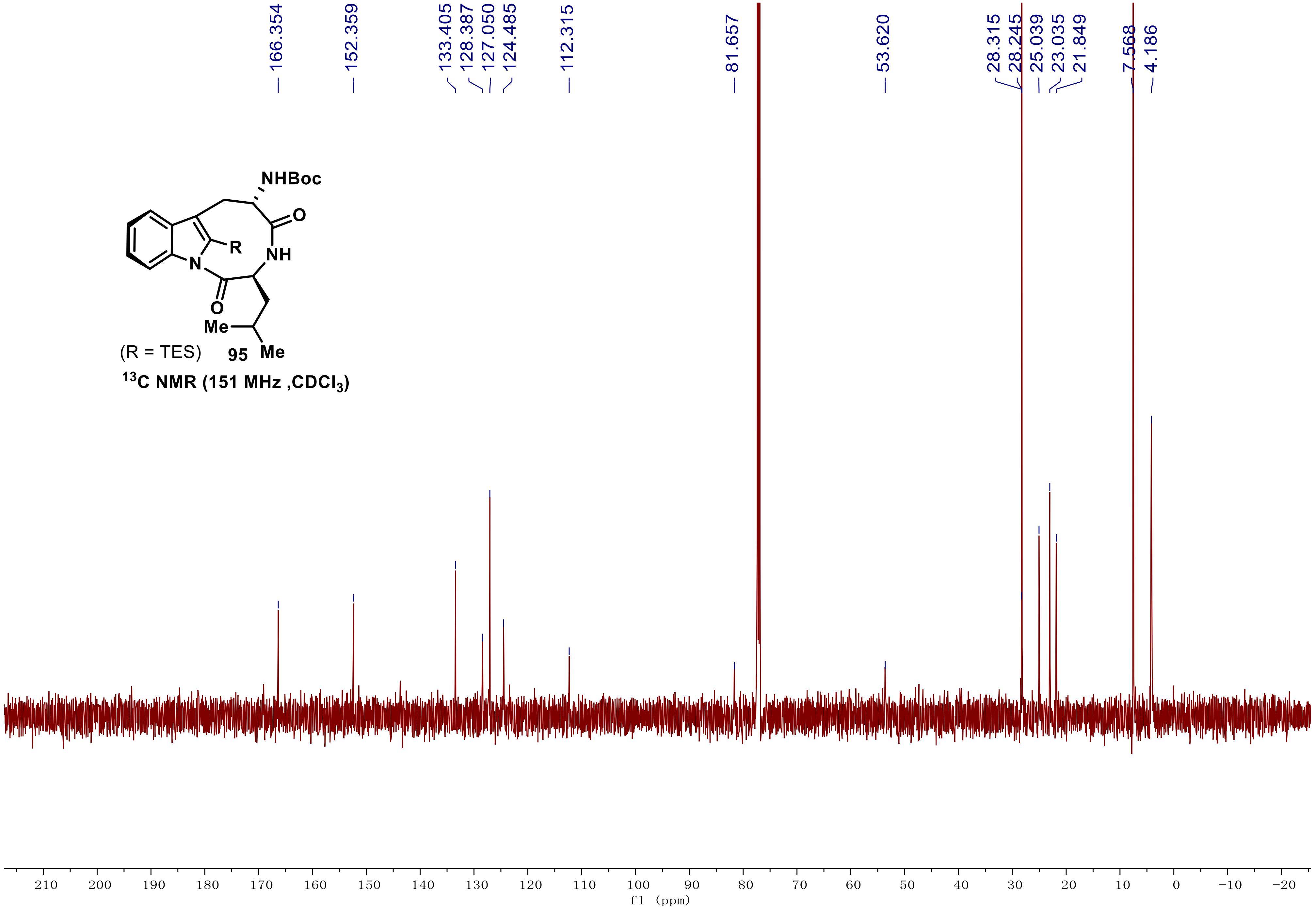
166.354
152.359
133.405
128.387
127.050
124.485
112.315
81.657
53.620
28.315
28.245
25.039
23.035
21.849
7.568
4.186
NHBoc
O
R
NH
N
O
Me
Me
(R = TES)
95
^{13}C NMR (151 MHz ,CDCl$_3$)
f1 (ppm)

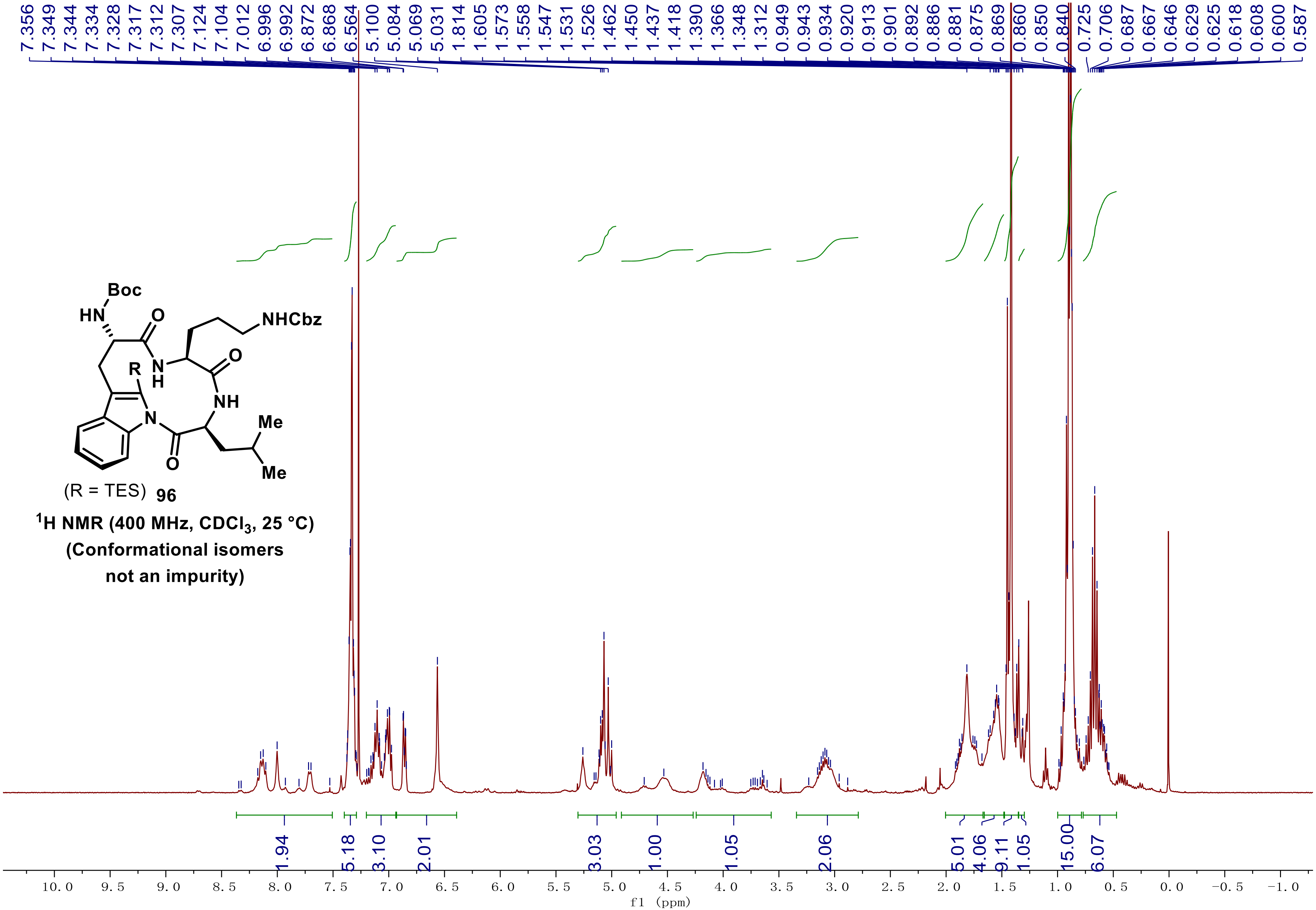

(R = TES) **96**

^{1}H NMR (400 MHz, $CDCl_3$, 25 °C)

(Conformational isomers not an impurity)

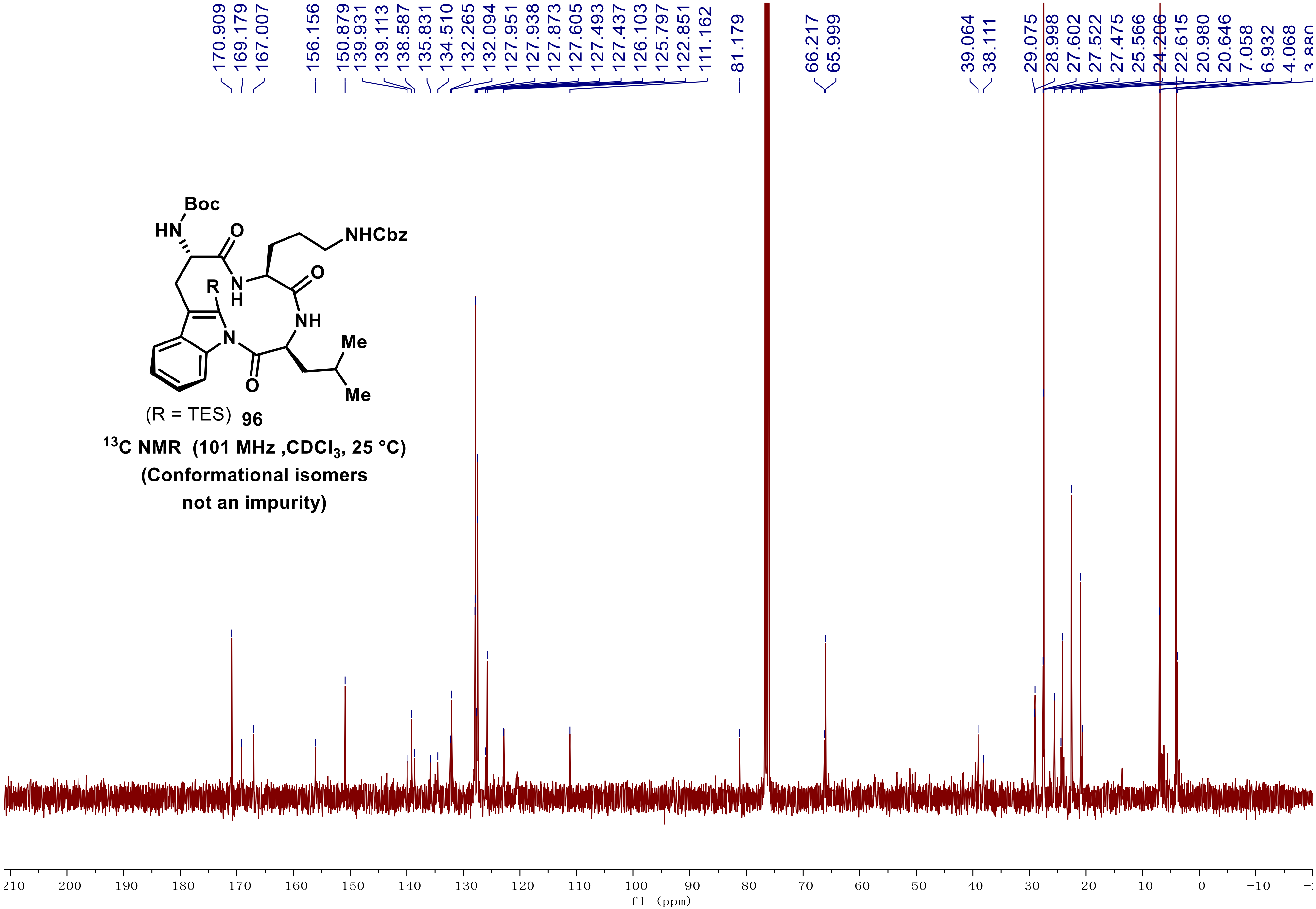
170.909
169.179
167.007
156.156
150.879
139.931
139.113
138.587
135.831
134.510
132.265
132.094
127.951
127.938
127.873
127.605
127.493
127.437
126.103
125.797
122.851
111.162
81.179
66.217
65.999
39.064
38.111
29.075
28.998
27.602
27.522
27.475
25.566
24.206
22.615
20.980
20.646
7.058
6.932
4.068
Boc
HN
O
NHCbz
R
N
H
O
NH
N
Me
O
Me
(R = TES) 96
^{13}C NMR (101 MHz ,CDCl$_3$, 25 °C)
(Conformational isomers
not an impurity)
f1 (ppm)

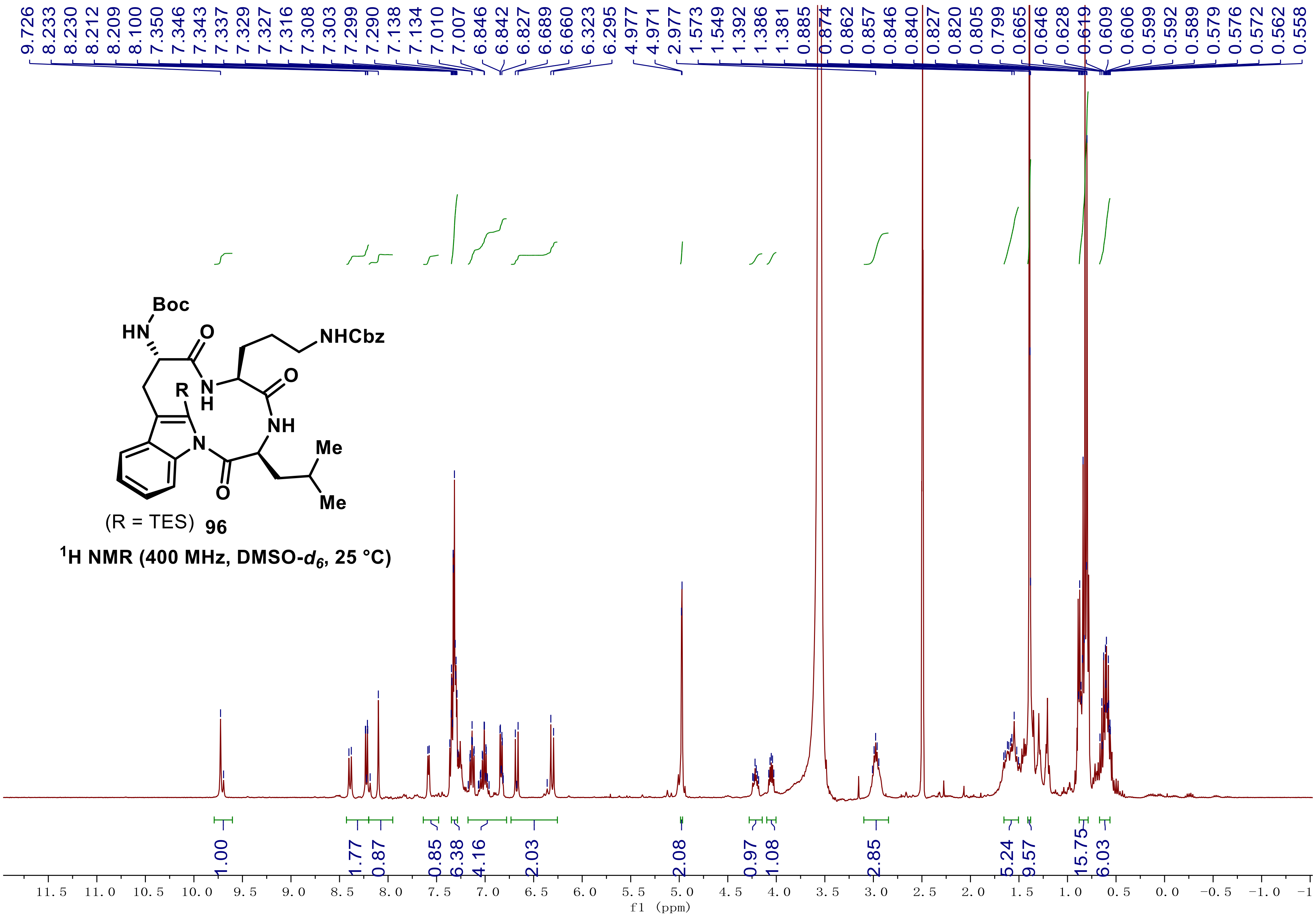
9.726
8.233
8.230
8.212
8.209
8.100
7.350
7.346
7.343
7.337
7.329
7.327
7.316
7.308
7.303
7.299
7.290
7.138
7.134
7.010
7.007
6.846
6.842
6.827
6.689
6.660
6.323
6.295
4.977
4.971
2.977
1.573
1.549
1.392
1.386
1.381
0.885
0.874
0.862
0.857
0.846
0.840
0.827
0.820
0.805
0.799
0.665
0.646
0.628
0.619
0.609
0.606
0.599
0.592
0.589
0.579
0.576
0.572
0.562
0.558
Boc
HN
O
NHCbz
O
R
N
H
NH
N
Me
O
Me
(R = TES) 96
1H NMR (400 MHz, DMSO-d6, 25 °C)
1.00
1.77
0.87
0.85
6.38
4.16
2.03
2.08
0.97
1.08
2.85
5.24
9.57
15.75
6.03
11.5
11.0
10.5
10.0
9.5
9.0
8.5
8.0
7.5
7.0
6.5
6.0
5.5
5.0
4.5
4.0
3.5
3.0
2.5
2.0
1.5
1.0
0.5
0.0
-0.5
-1.0
-1
f1 (ppm)

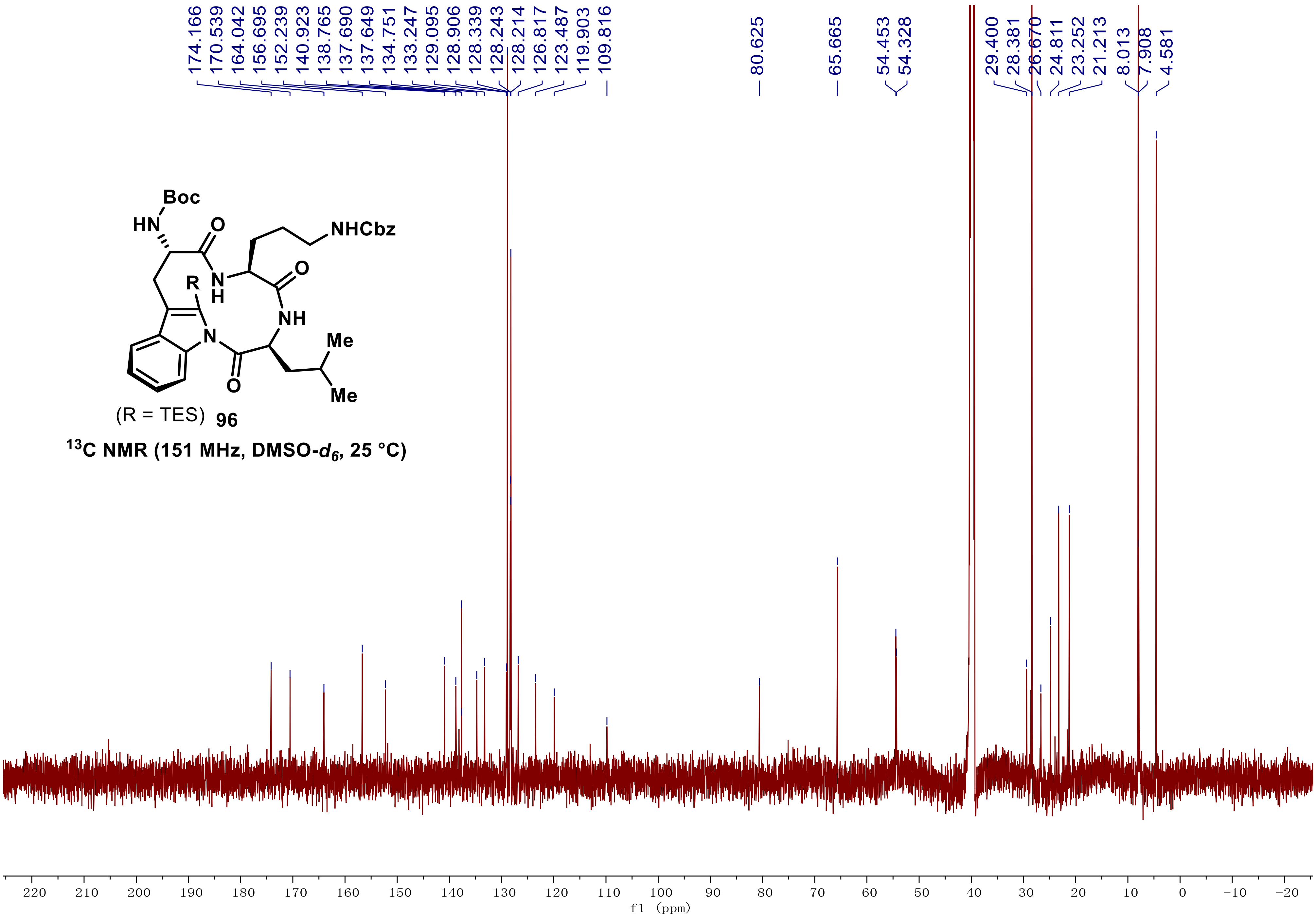

(R = TES) **96**

^{13}C NMR (151 MHz, DMSO-d_6, 25 °C)

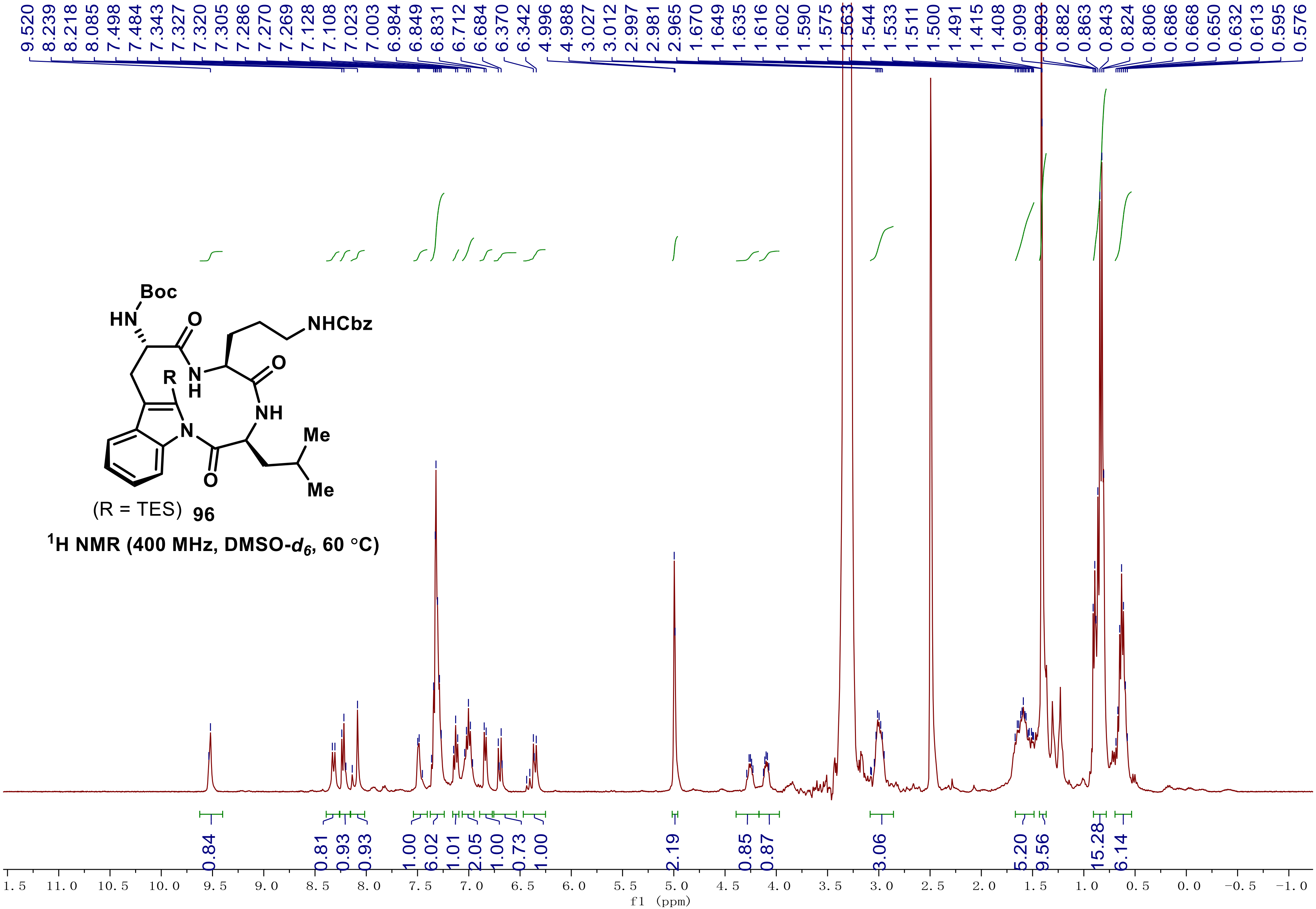
9.520 8.239 8.218 8.085 7.498 7.484 7.343 7.327 7.320 7.305 7.286 7.270 7.269 7.128 7.108 7.023 7.003 6.984 6.849 6.831 6.712 6.684 6.370 6.342 4.996 4.988 3.027 3.012 2.997 2.981 2.965 1.670 1.649 1.635 1.616 1.602 1.590 1.575 1.563 1.544 1.533 1.511 1.500 1.491 1.415 1.408 0.909 0.893 0.882 0.863 0.843 0.824 0.806 0.686 0.668 0.650 0.632 0.613 0.595 0.576
Boc
HN
O
NHCbz
O
R
N
H
NH
N
Me
Me
O
(R = TES)
96
^{1}H NMR (400 MHz, DMSO-d_6, 60 °C)
0.84 0.81 0.93 0.93 1.00 6.02 1.01 2.05 1.00 0.73 1.00 2.19 0.85 0.87 3.06 5.20 9.56 15.28 6.14
f1 (ppm)

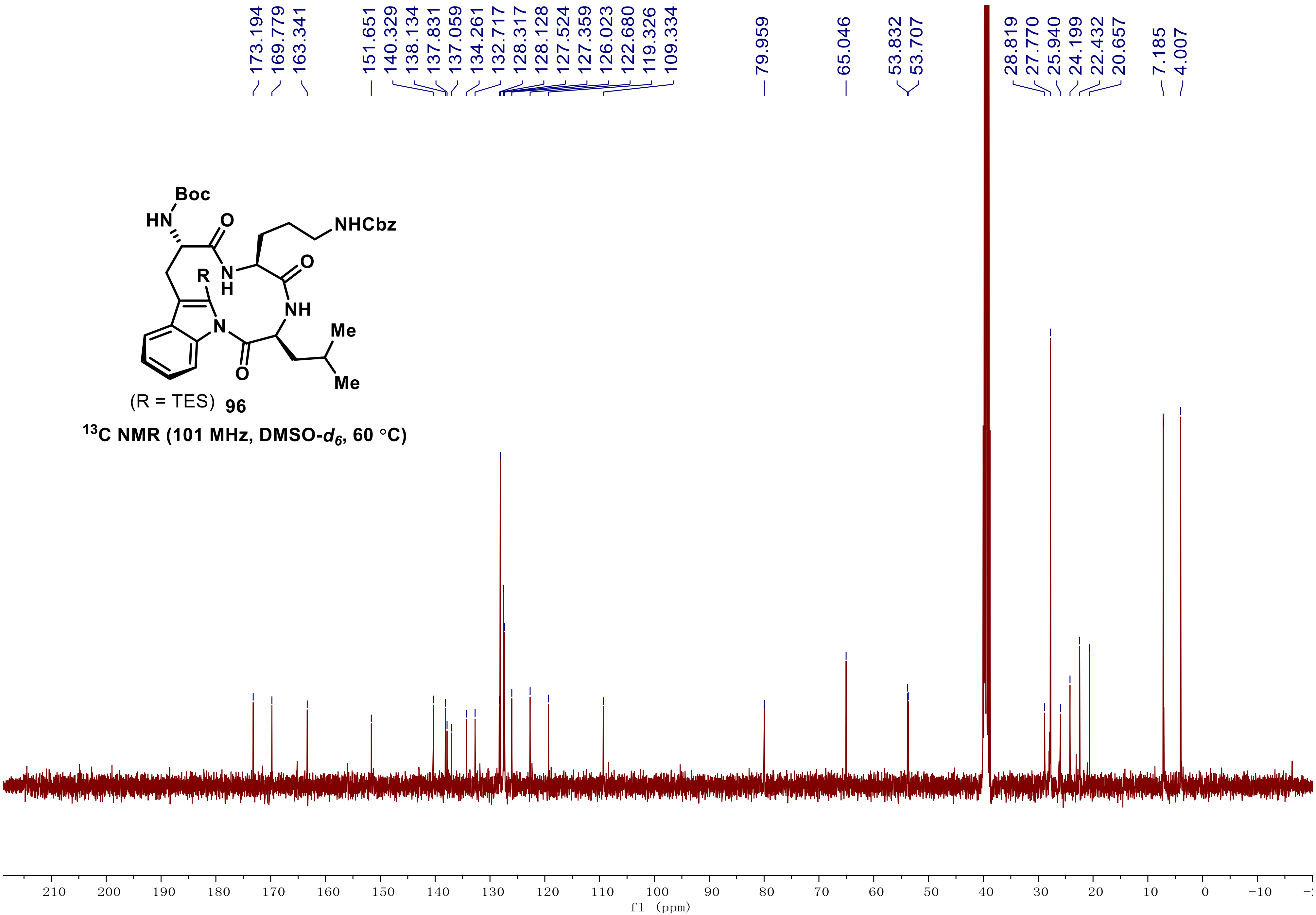

(R = TES) **96**

^{13}C NMR (101 MHz, DMSO-d_6, 60 °C)

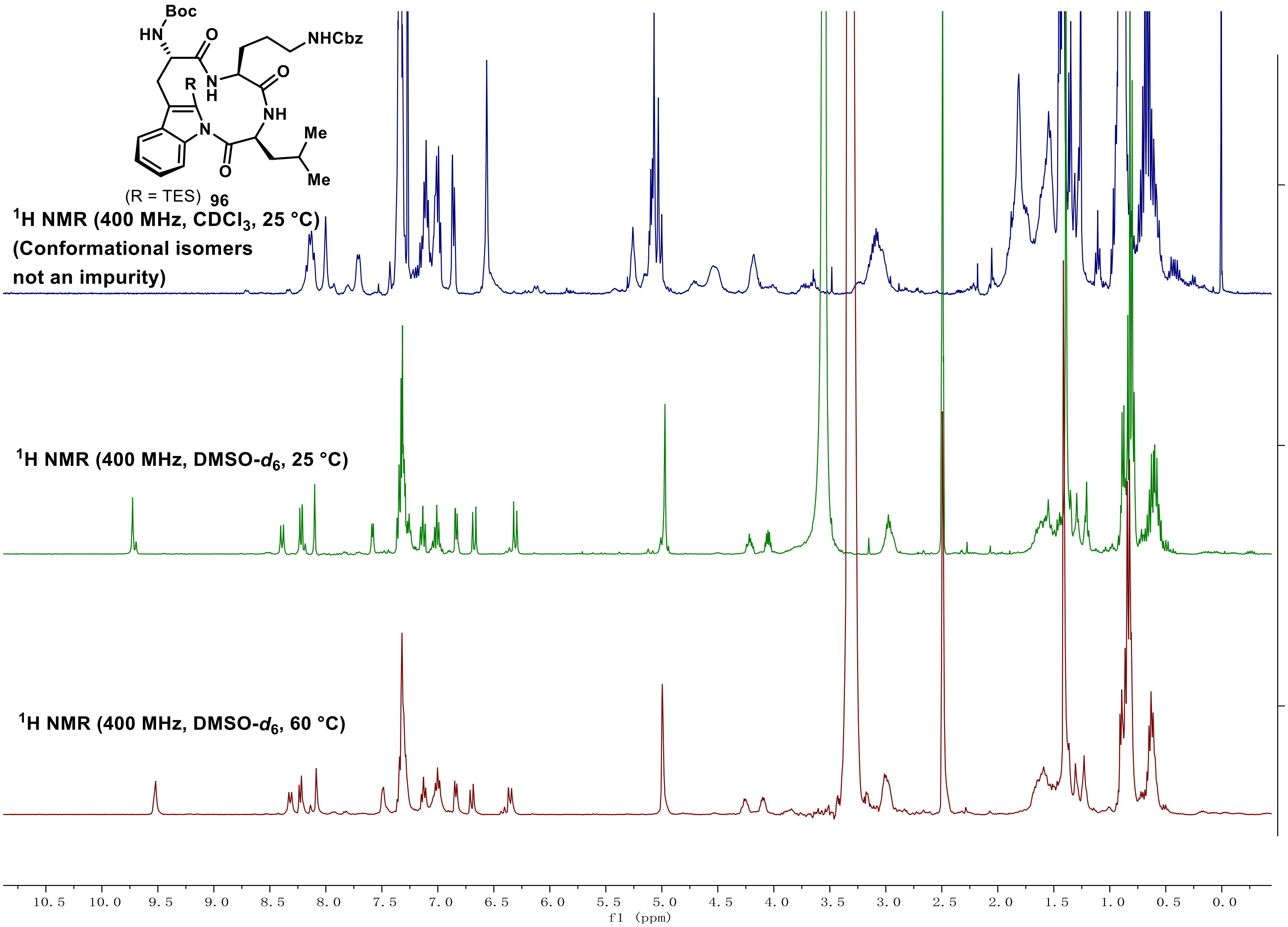
Boc
HN
O
NHCbz
R
N
H
O
NH
N
Me
O
Me
(R = TES) 96
1H NMR (400 MHz, CDCl3, 25 °C)
(Conformational isomers
not an impurity)
1H NMR (400 MHz, DMSO-d6, 25 °C)
1H NMR (400 MHz, DMSO-d6, 60 °C)
10.5 10.0 9.5 9.0 8.5 8.0 7.5 7.0 6.5 6.0 5.5 5.0 4.5 4.0 3.5 3.0 2.5 2.0 1.5 1.0 0.5 0.0
f1 (ppm)
3
2
1

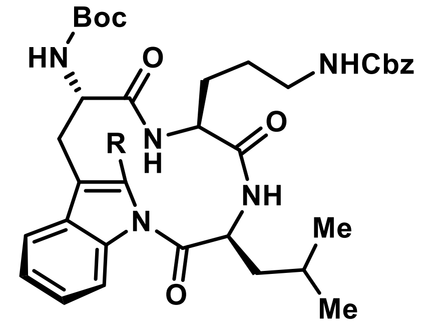

(R = TES) **96**

^{13}C NMR (101 MHz, $CDCl_3$, 25 °C)

^{13}C NMR (101 MHz, DMSO-d_6, 60 °C)

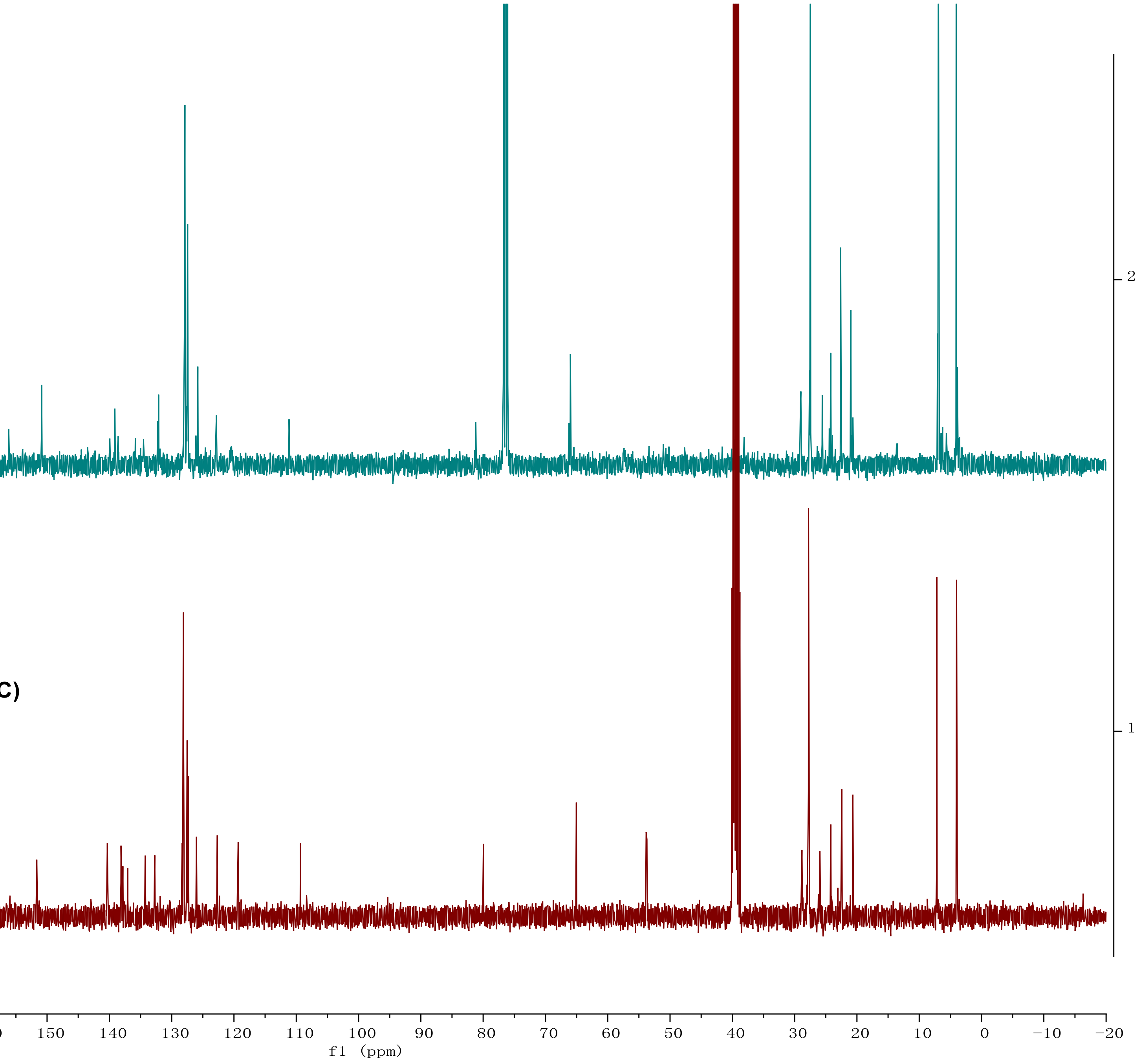

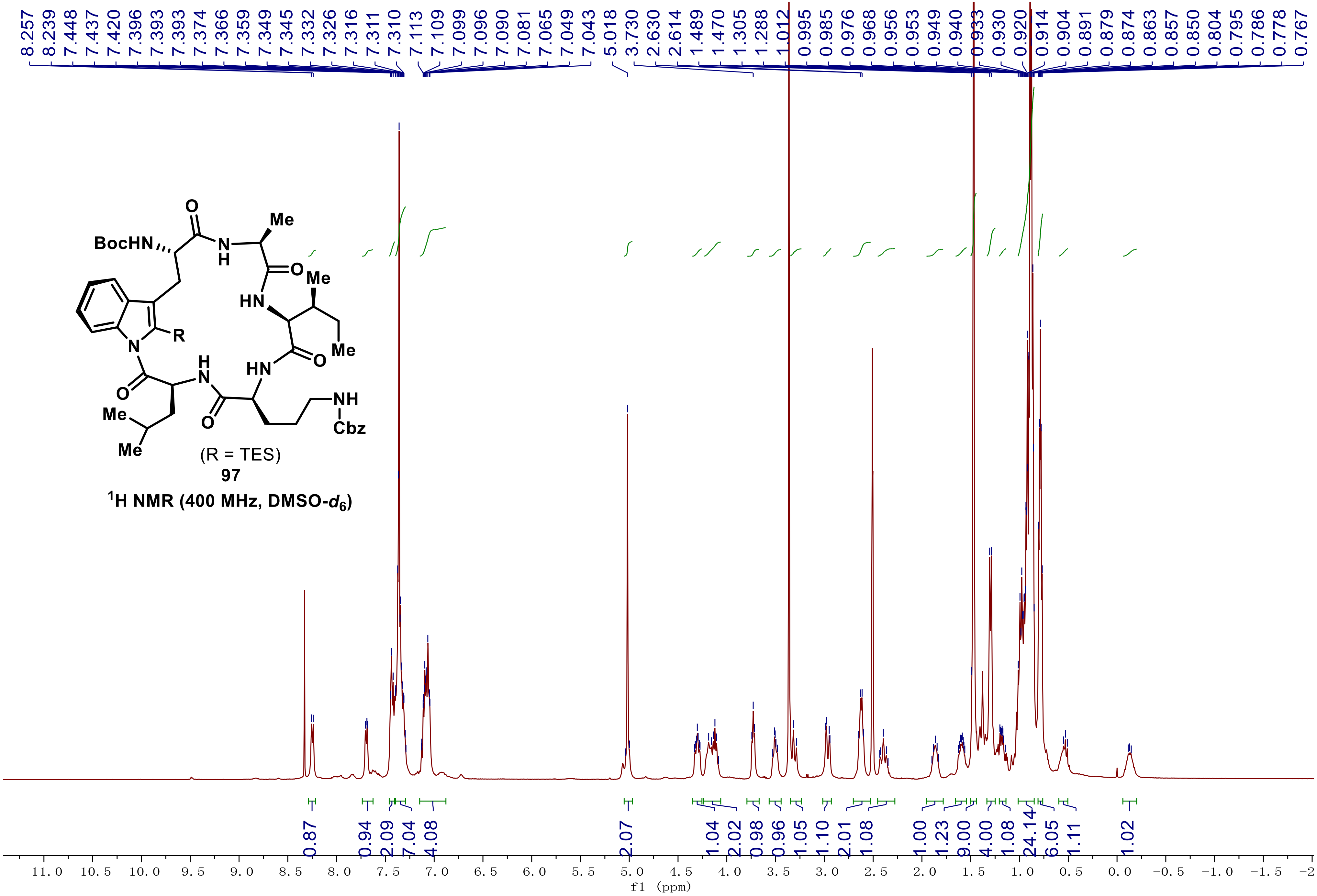

(R = TES)

97

^{1}H NMR (400 MHz, DMSO-d_6)

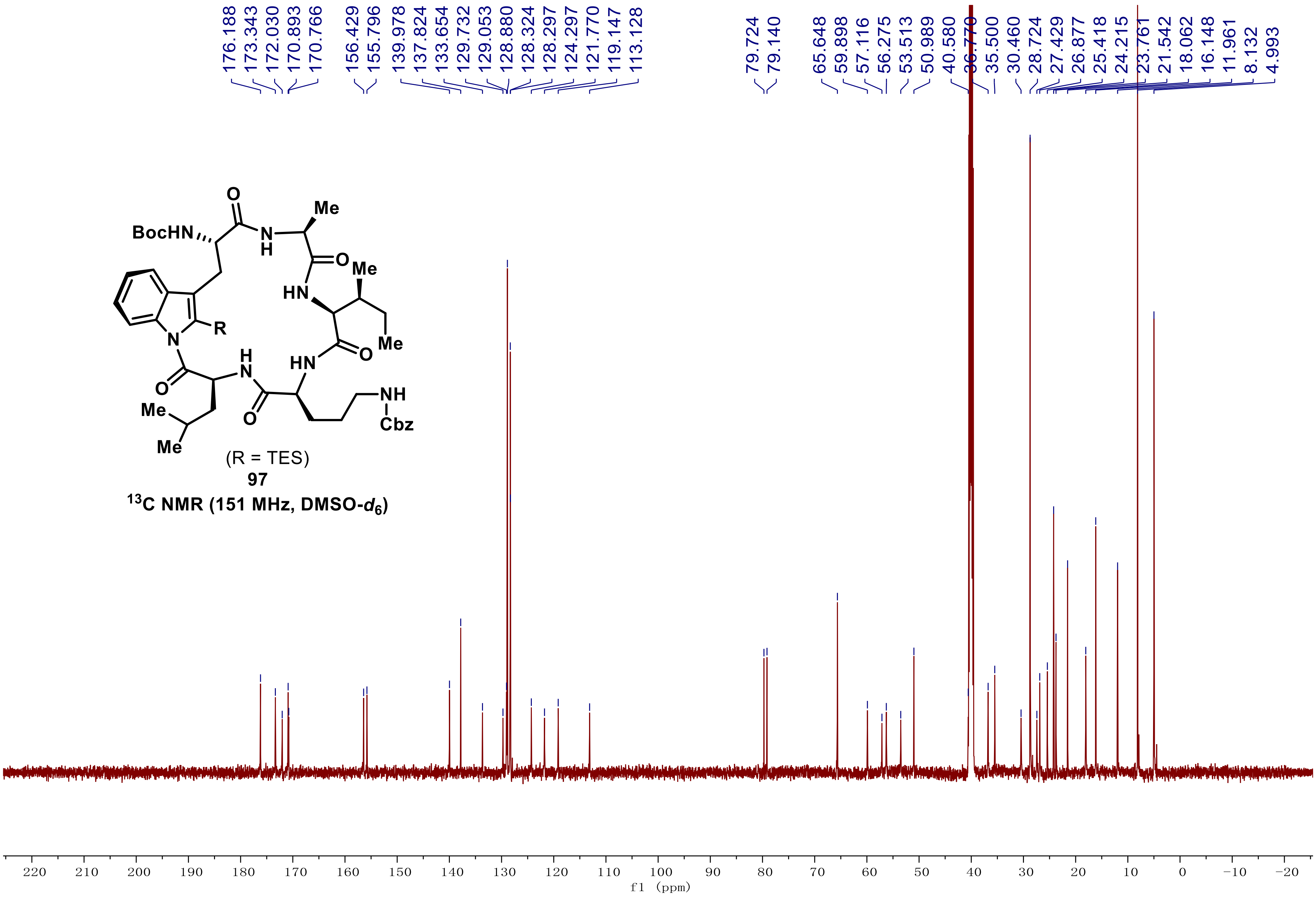

(R = TES)

97

^{13}C NMR (151 MHz, DMSO-d_6)

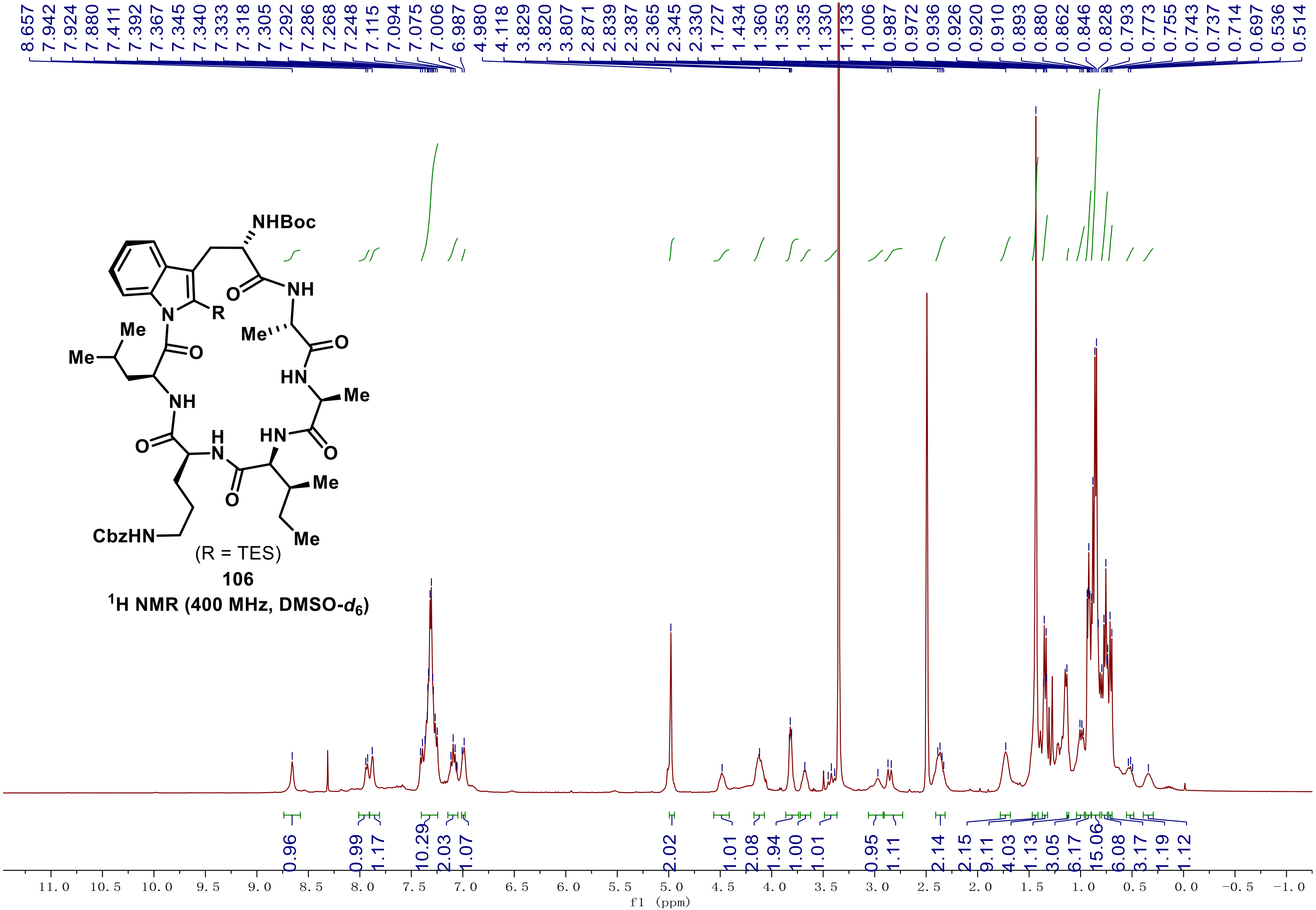

NHBoc
NH
O
N
R
Me
Me
O
Me
O
HN
Me
NH
O
H
N
HN
O
Me
O
CbzHN
Me
(R = TES)
106
^{1}H NMR (400 MHz, DMSO-d_6)
f1 (ppm)

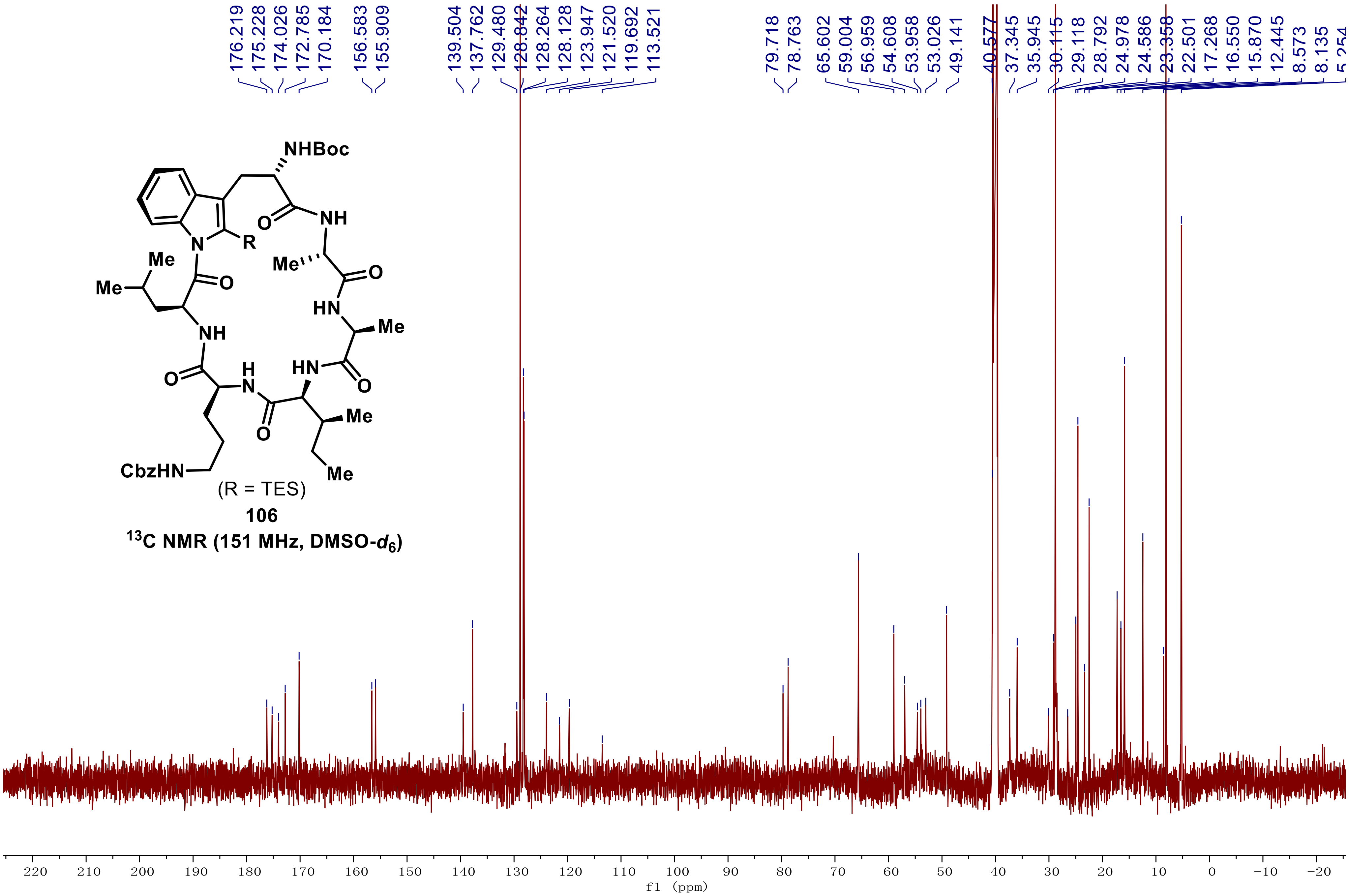

(R = TES)

106

^{13}C NMR (151 MHz, DMSO-d_6)

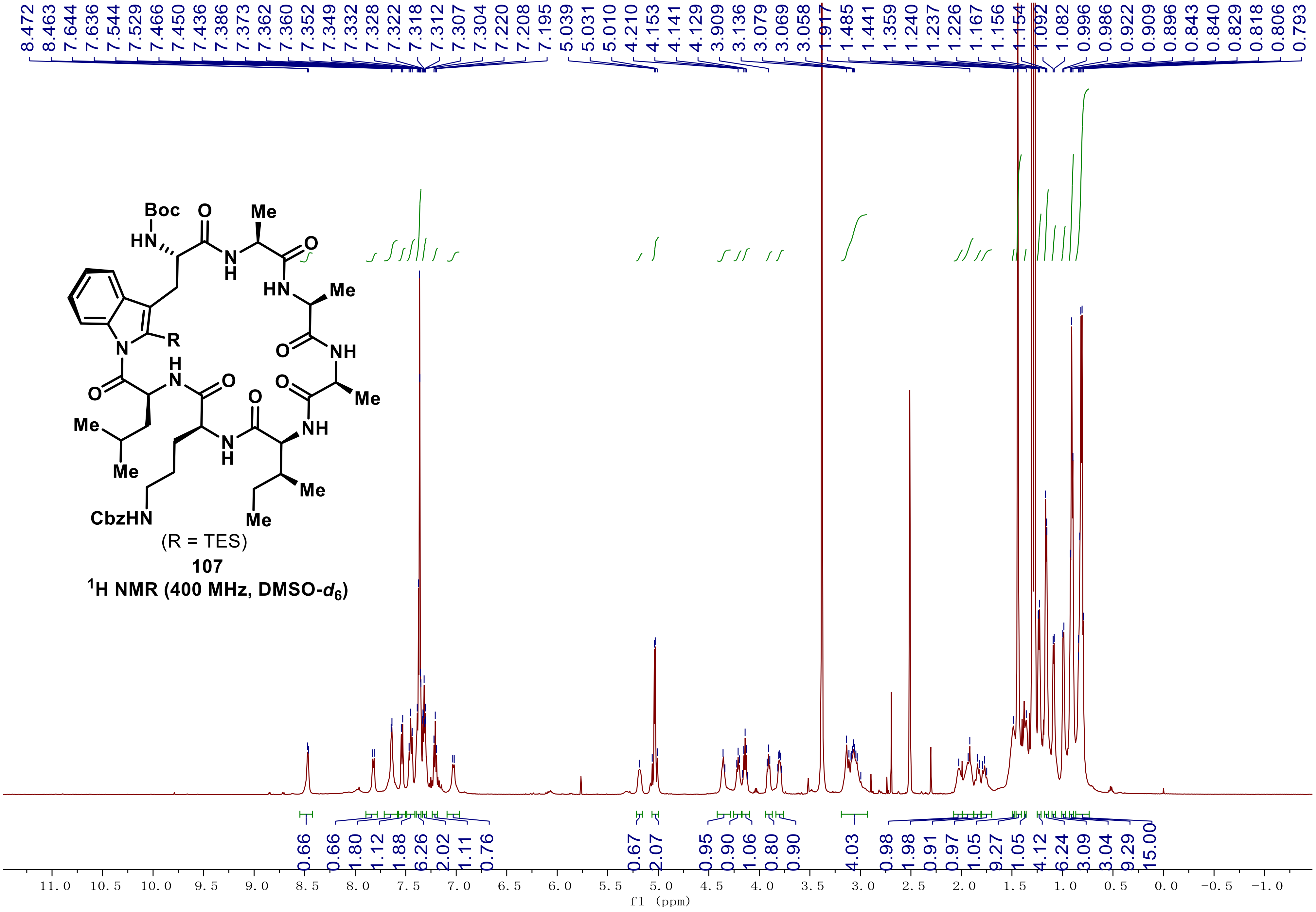

8.472 8.463 7.644 7.636 7.544 7.529 7.466 7.450 7.436 7.386 7.373 7.362 7.360 7.352 7.349 7.332 7.328 7.322 7.318 7.312 7.307 7.304 7.220 7.208 7.195 5.039 5.031 5.010 4.210 4.153 4.141 4.129 3.909 3.136 3.079 3.069 3.058 1.917 1.485 1.441 1.359 1.240 1.237 1.226 1.167 1.156 1.154 1.092 1.082 0.996 0.986 0.922 0.909 0.896 0.843 0.840 0.829 0.818 0.806 0.793
Boc
O
Me
HN
N
H
O
HN
Me
R
N
O
NH
H
N
O
O
O
Me
Me
N
H
NH
Me
Me
Me
CbzHN
Me
(R = TES)
107
1H NMR (400 MHz, DMSO-d6)
0.66 0.66 1.80 1.12 1.88 6.26 2.02 1.11 0.76 0.67 2.07 0.95 0.90 1.06 0.80 0.90 4.03 0.98 1.98 0.91 0.97 1.05 9.27 1.05 4.12 6.24 3.09 3.04 9.29 15.00
11.0 10.5 10.0 9.5 9.0 8.5 8.0 7.5 7.0 6.5 6.0 5.5 5.0 4.5 4.0 3.5 3.0 2.5 2.0 1.5 1.0 0.5 0.0 -0.5 -1.0
f1 (ppm)

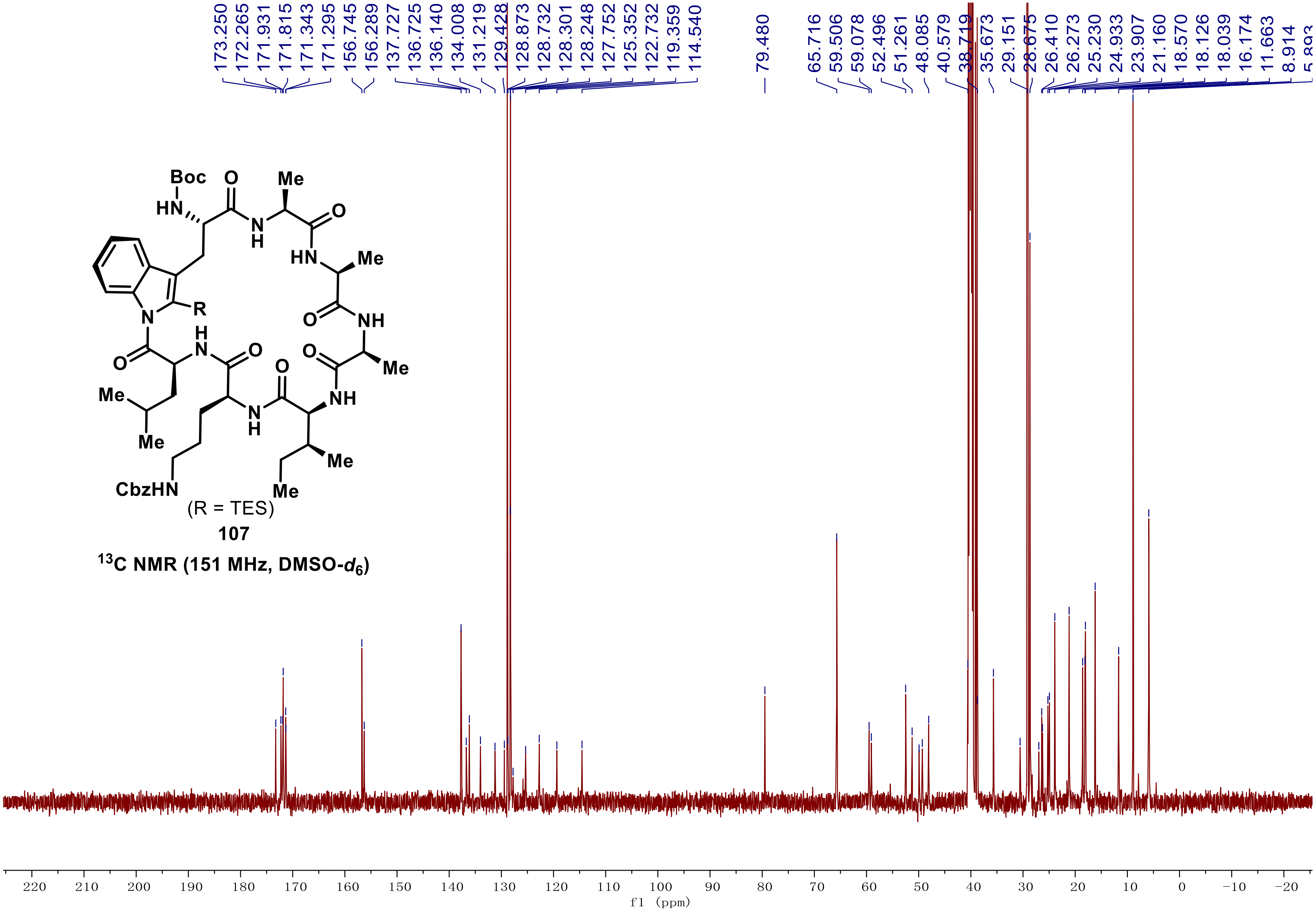

(R = TES)

107

^{13}C NMR (151 MHz, DMSO-d_6)

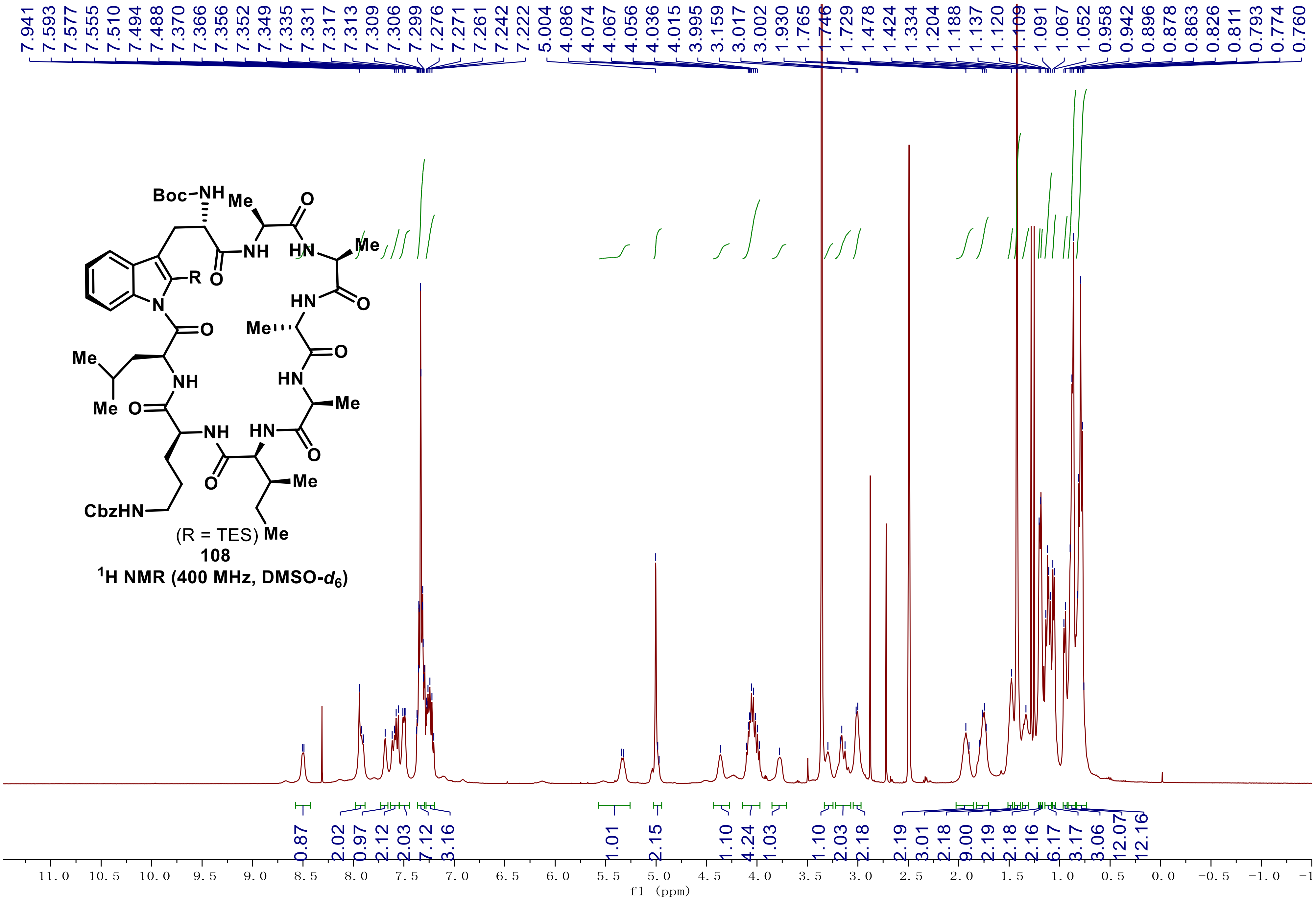
7.941 7.593 7.577 7.555 7.510 7.494 7.488 7.370 7.366 7.356 7.352 7.349 7.335 7.331 7.317 7.313 7.309 7.306 7.299 7.276 7.271 7.261 7.242 7.222 5.004 4.086 4.074 4.067 4.056 4.036 4.015 3.995 3.159 3.017 3.002 1.930 1.765 1.746 1.729 1.478 1.424 1.334 1.204 1.188 1.137 1.120 1.109 1.091 1.067 1.052 0.958 0.942 0.896 0.878 0.863 0.826 0.811 0.793 0.774 0.760
Boc–NH
Me
O
NH
HN
Me
R
O
N
HN
O
O
Me
Me
O
HN
Me
NH
Me
O
HN
Me
NH
HN
O
O
Me
CbzHN
(R = TES) Me
108
^{1}H NMR (400 MHz, DMSO-d_6)
0.87 2.02 0.97 2.12 2.03 7.12 3.16 1.01 2.15 1.10 4.24 1.03 1.10 2.03 2.18 2.19 3.01 2.18 9.00 2.19 2.18 2.16 6.17 3.17 3.06 12.07 12.16
11.0 10.5 10.0 9.5 9.0 8.5 8.0 7.5 7.0 6.5 6.0 5.5 5.0 4.5 4.0 3.5 3.0 2.5 2.0 1.5 1.0 0.5 0.0 −0.5 −1.0 −1
f1 (ppm)

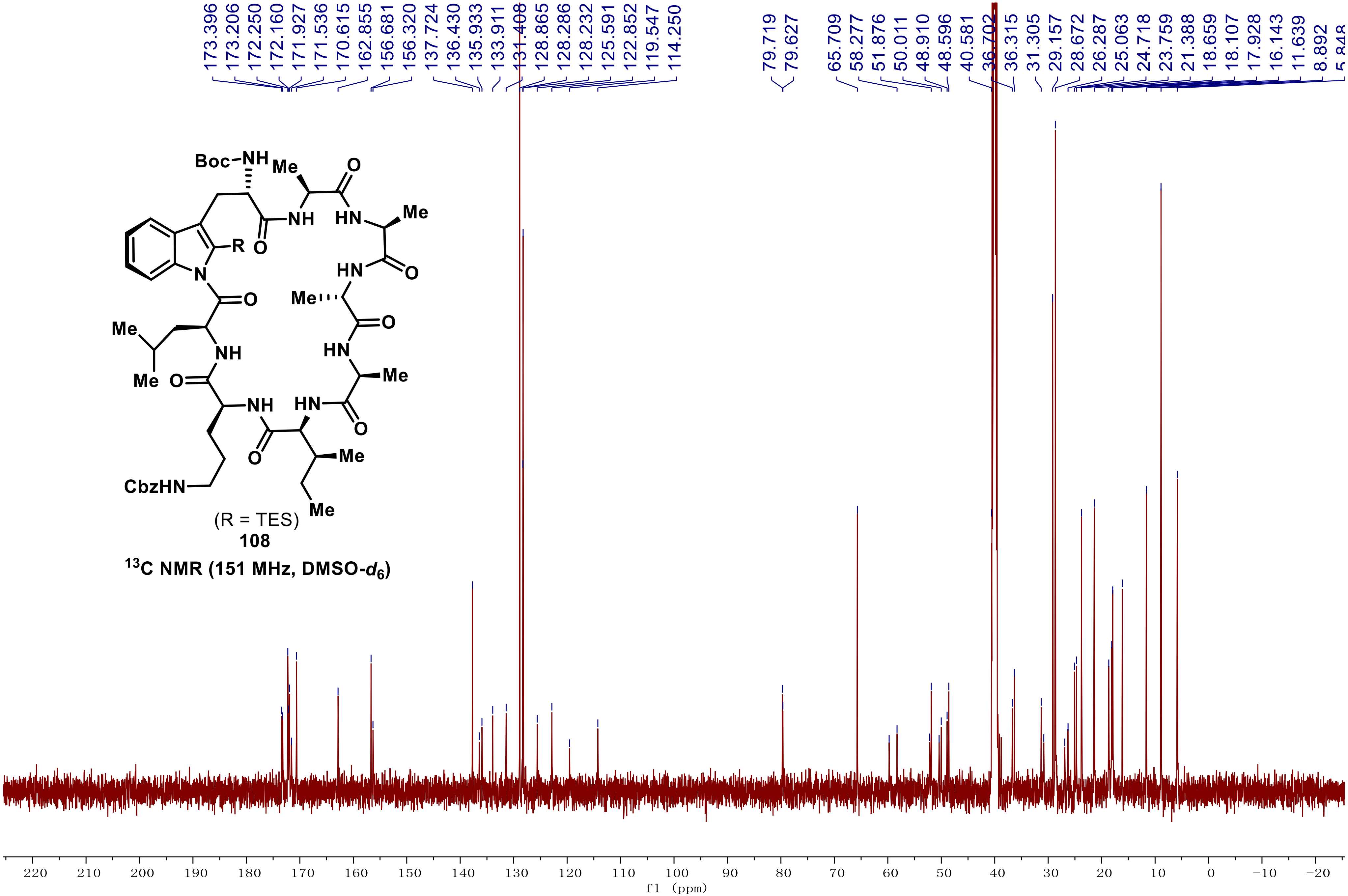
173.396
173.206
172.250
172.160
171.927
171.536
170.615
162.855
156.681
156.320
137.724
136.430
135.933
133.911
131.408
128.865
128.286
128.232
125.591
122.852
119.547
114.250
79.719
79.627
65.709
58.277
51.876
50.011
48.910
48.596
40.581
36.702
36.315
31.305
29.157
28.672
26.287
25.063
24.718
23.759
21.388
18.659
18.107
17.928
16.143
11.639
8.892
5.848
Boc–NH
CbzHN
(R = TES)
108
^{13}C NMR (151 MHz, DMSO-d_6)
220 210 200 190 180 170 160 150 140 130 120 110 100 90 80 70 60 50 40 30 20 10 0 −10 −20
f1 (ppm)

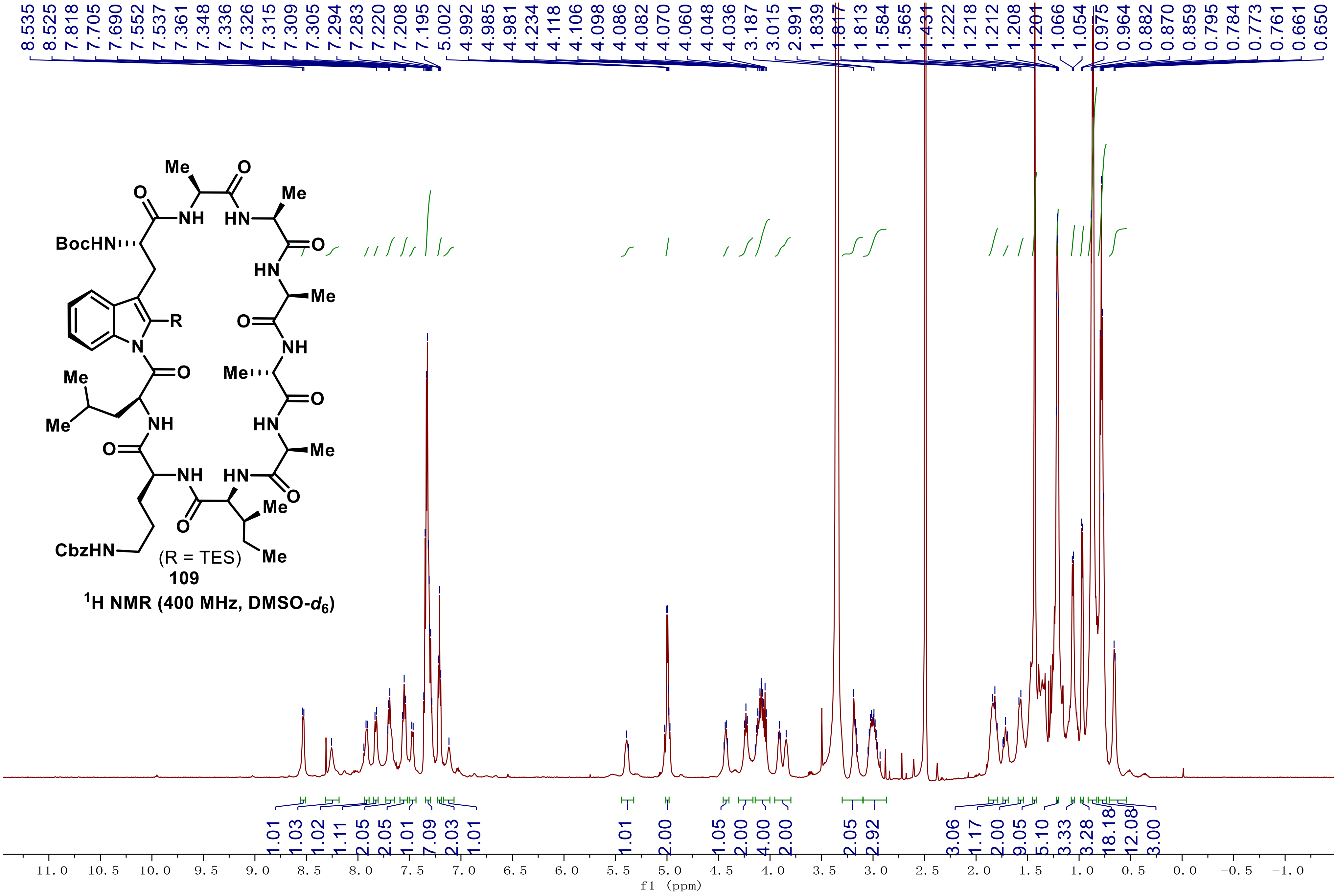

BocHN
CbzHN
(R = TES)
109
^{1}H NMR (400 MHz, DMSO-d_6)
f1 (ppm)

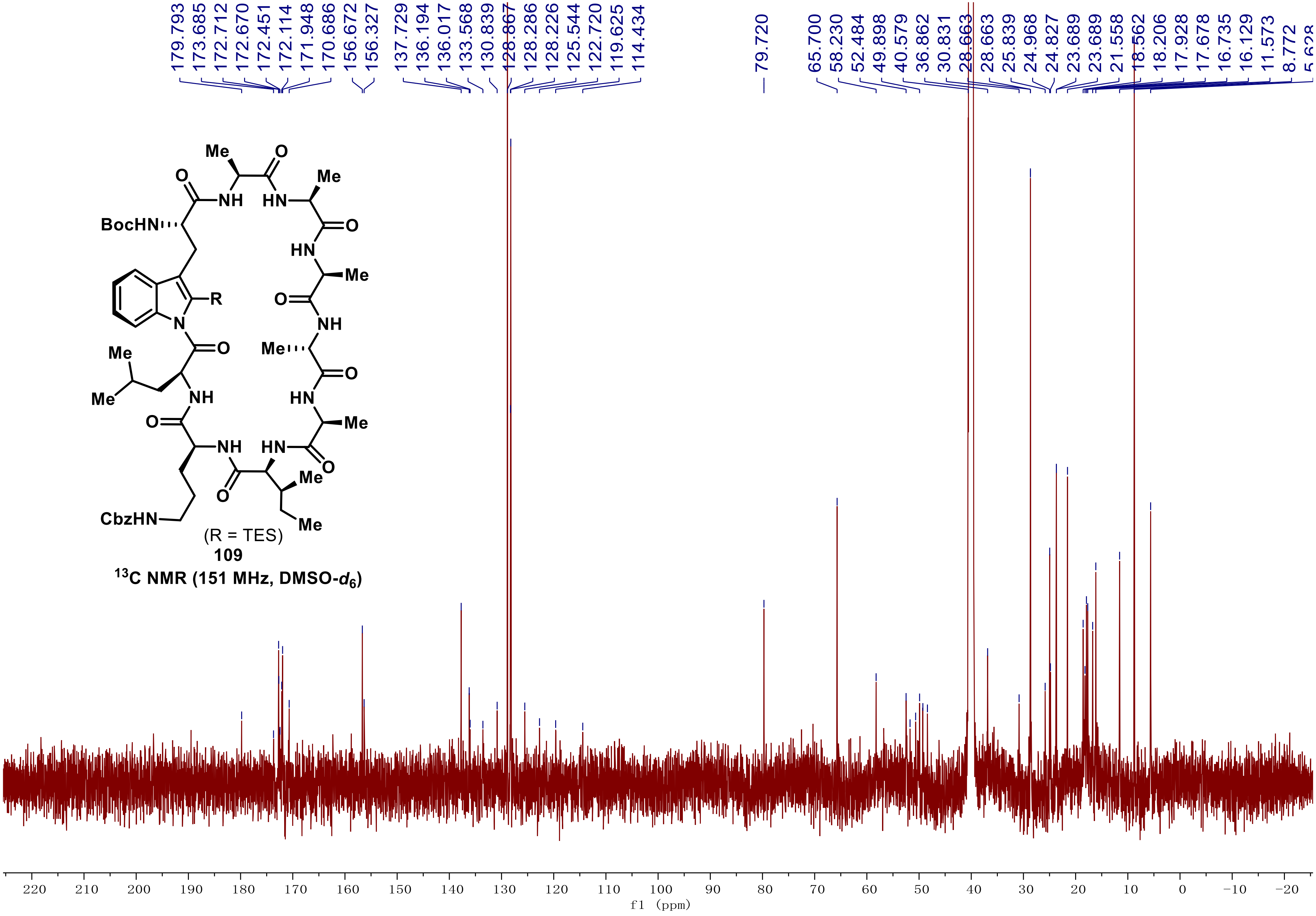

(R = TES)

109

^{13}C NMR (151 MHz, DMSO-d_6)

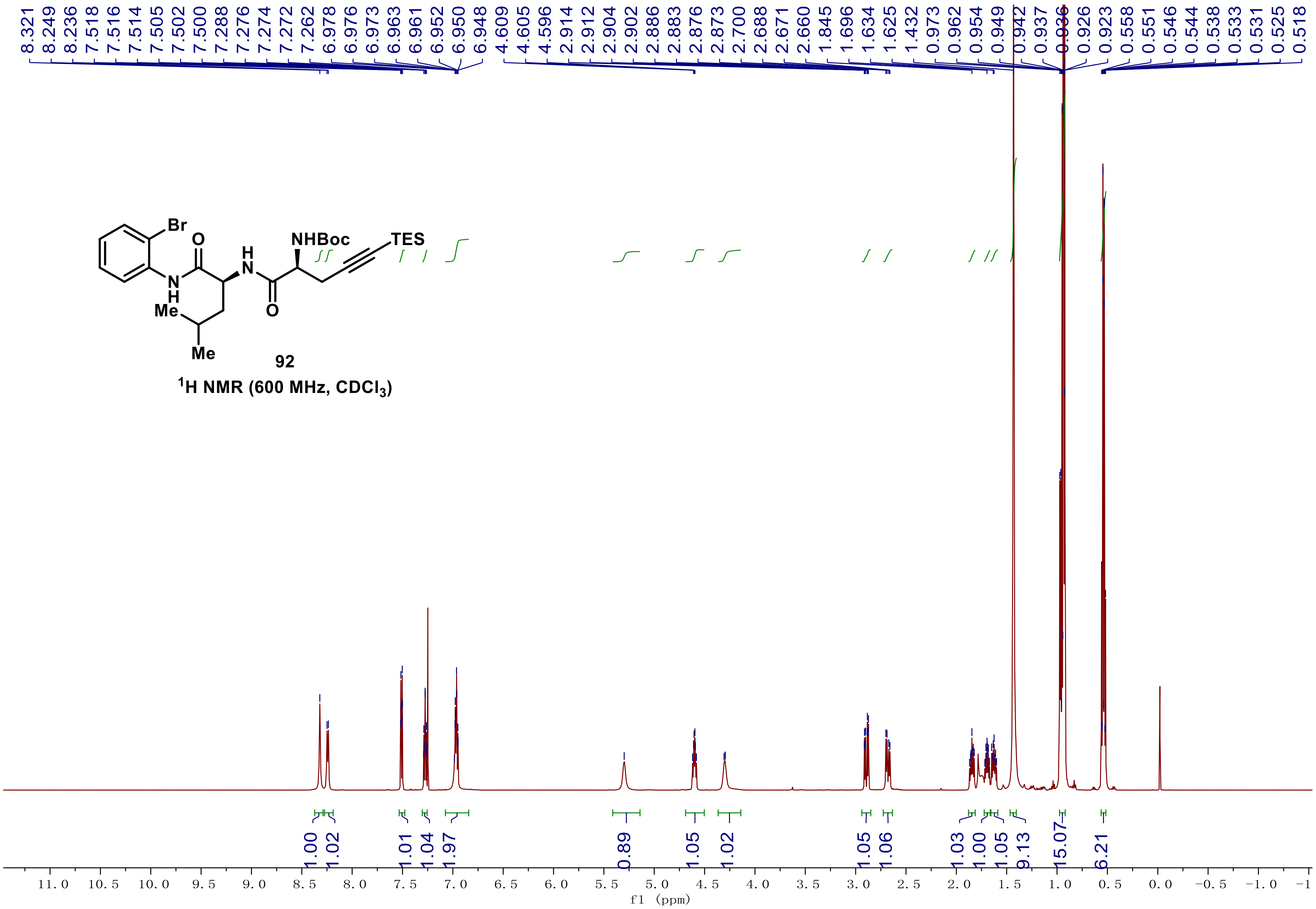

^{1}H NMR (600 MHz, $CDCl_3$)

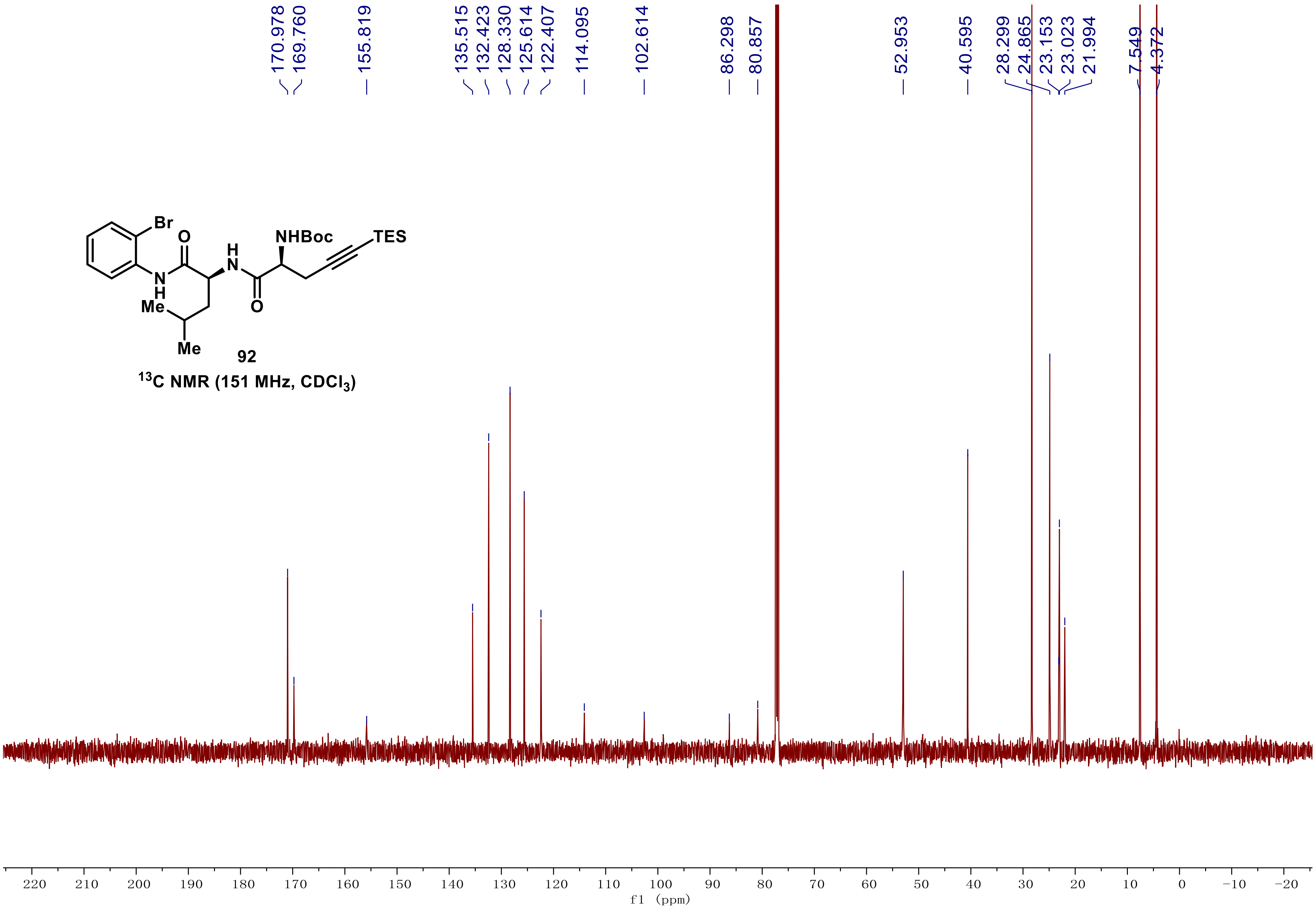

170.978
169.760
155.819
135.515
132.423
128.330
125.614
122.407
114.095
102.614
86.298
80.857
52.953
40.595
28.299
24.865
23.153
23.023
21.994
7.549
4.372
Br
O
H
N
NHBoc
TES
N
H
Me
O
Me
92
^{13}C NMR (151 MHz, $CDCl_3$)
220 210 200 190 180 170 160 150 140 130 120 110 100 90 80 70 60 50 40 30 20 10 0 −10 −20
f1 (ppm)

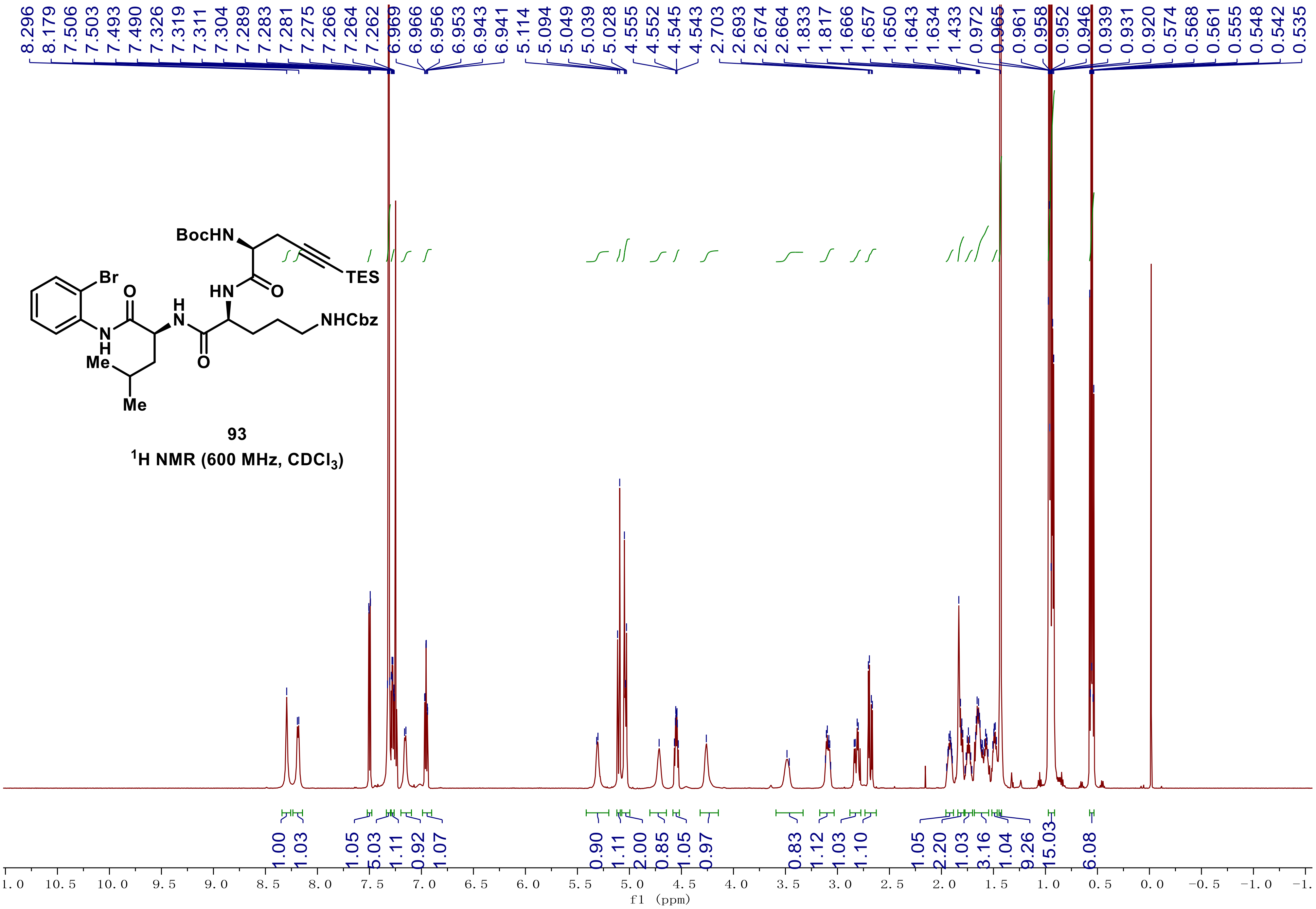

93

^{1}H NMR (600 MHz, $CDCl_3$)

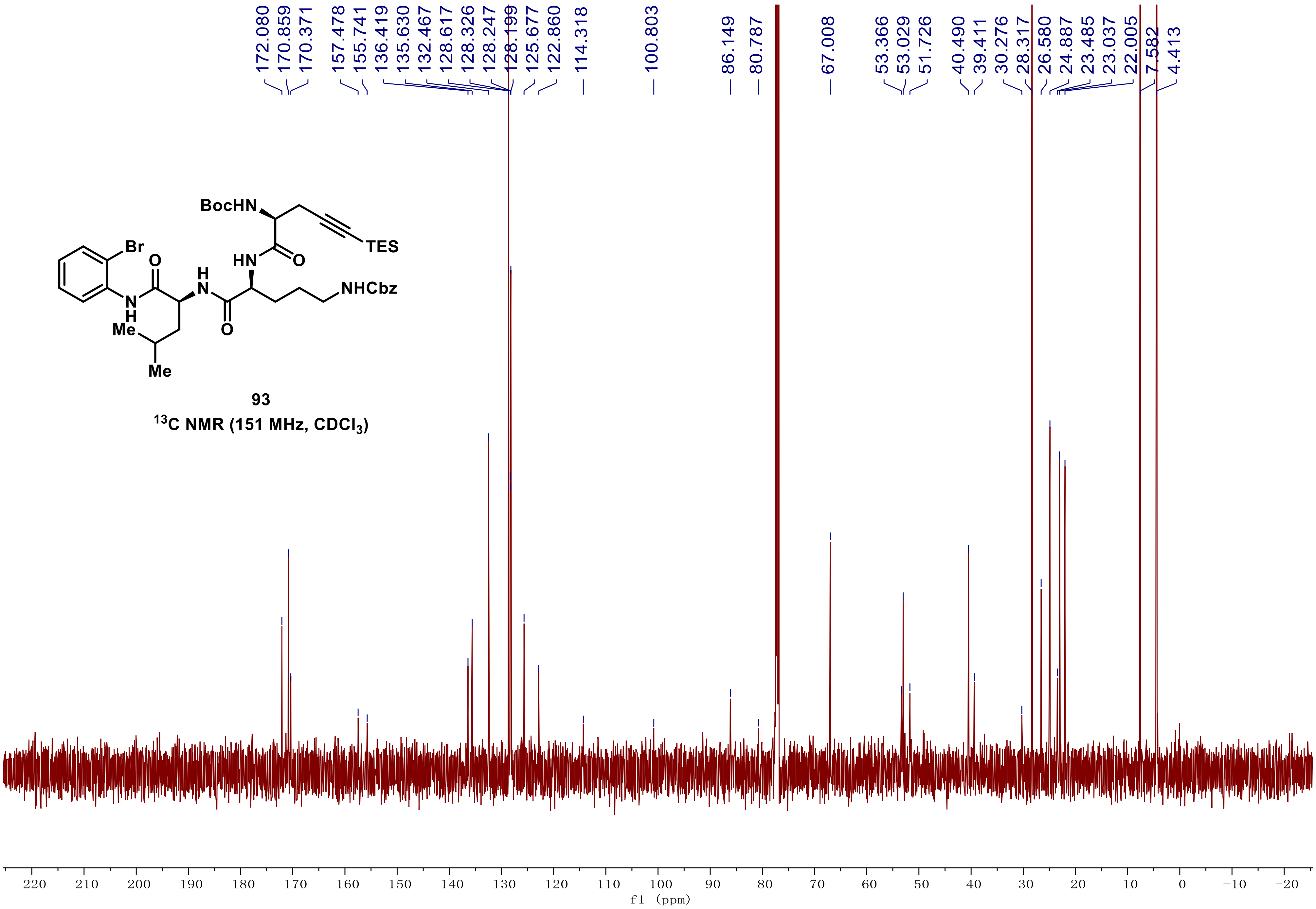
172.080
170.859
170.371
157.478
155.741
136.419
135.630
132.467
128.617
128.326
128.247
128.199
125.677
122.860
114.318
100.803
86.149
80.787
67.008
53.366
53.029
51.726
40.490
39.411
30.276
28.317
26.580
24.887
23.485
23.037
22.005
7.582
4.413
BocHN
TES
Br
O
HN
O
H
N
NHCbz
N
H
Me
O
Me
93
13C NMR (151 MHz, CDCl3)
220 210 200 190 180 170 160 150 140 130 120 110 100 90 80 70 60 50 40 30 20 10 0 −10 −20
f1 (ppm)

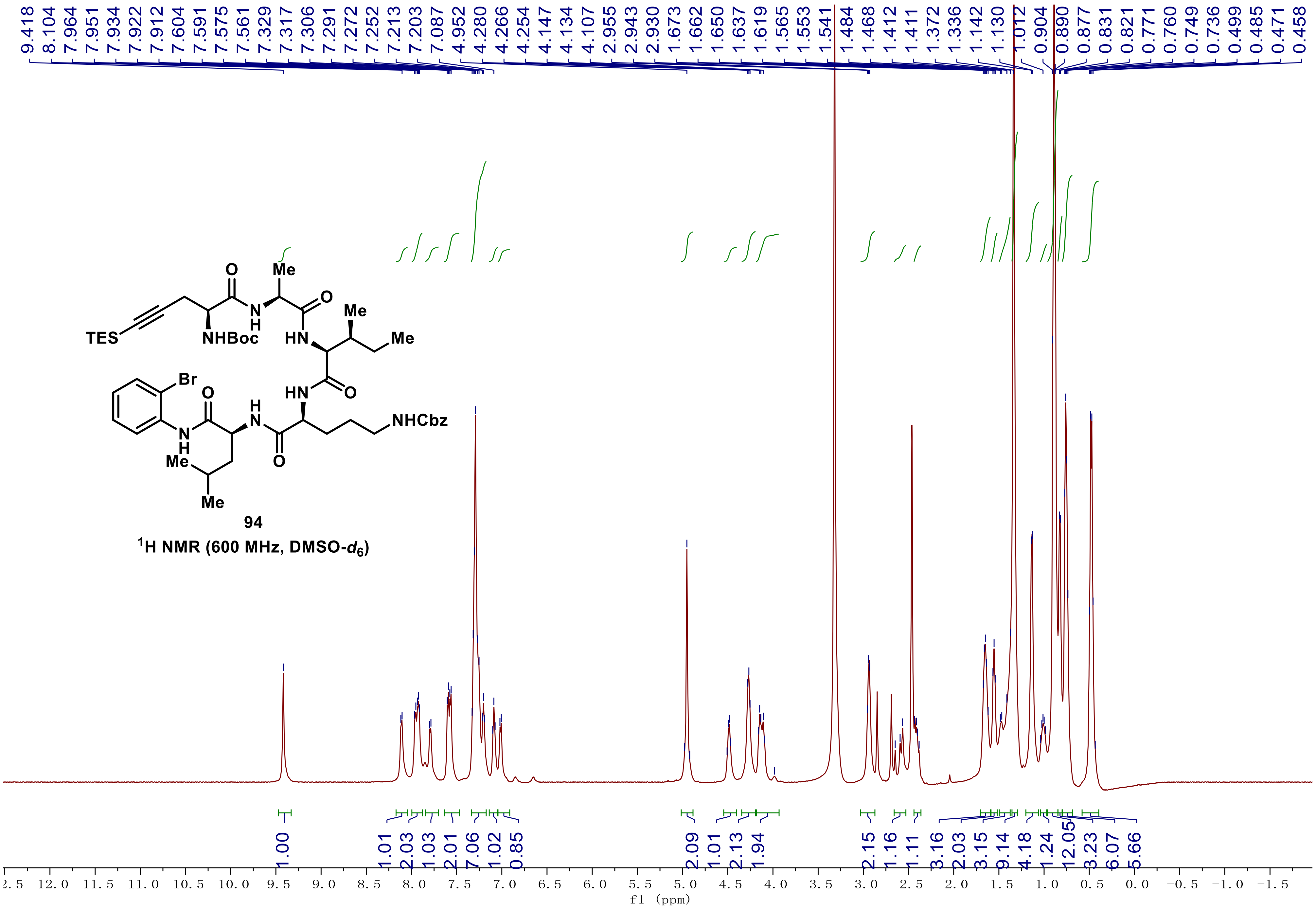

TES
NHBoc
Me
HN
Me
Me
Br
NHCbz
Me
Me
94
1H NMR (600 MHz, DMSO-d6)
f1 (ppm)

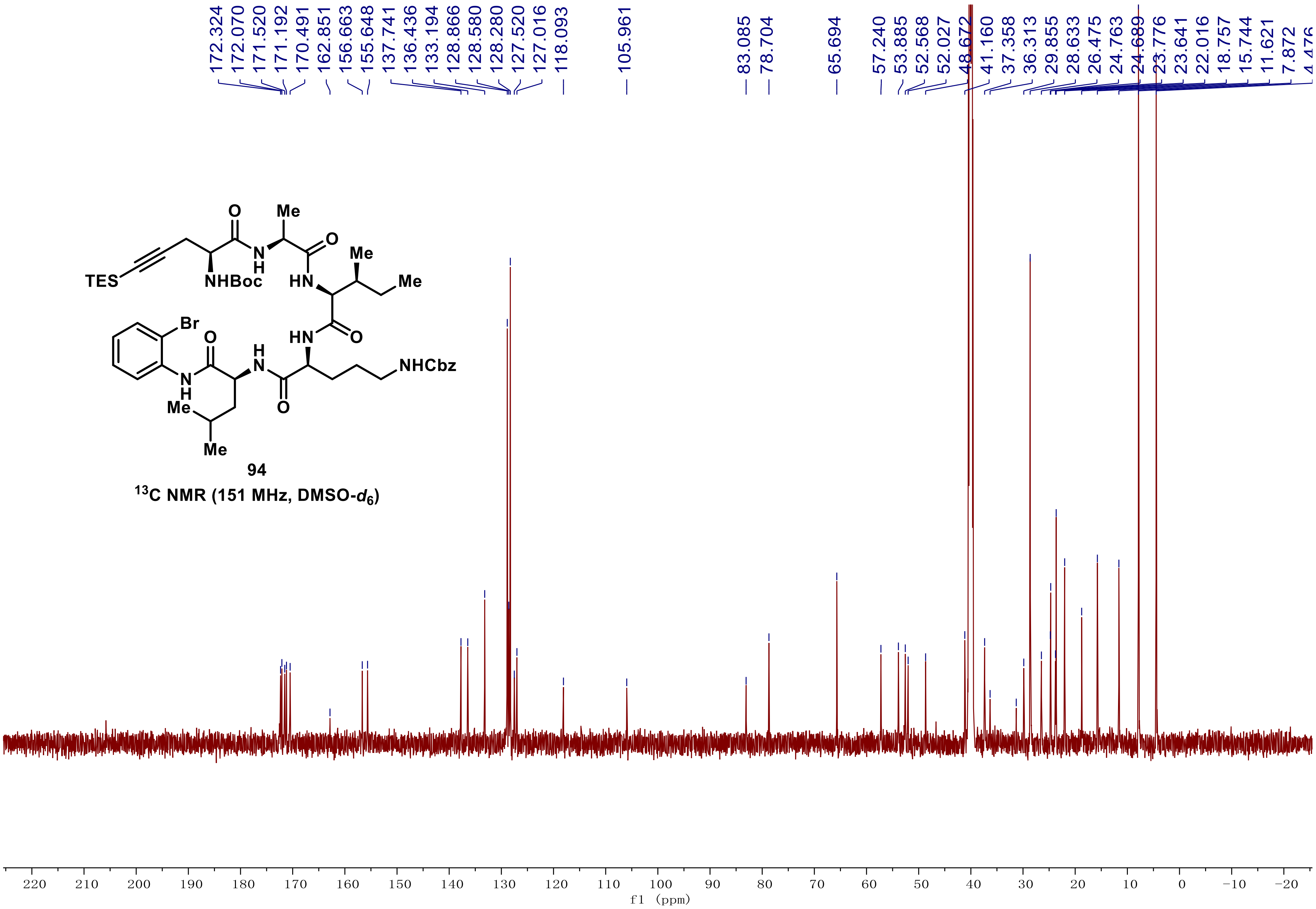

94

^{13}C NMR (151 MHz, DMSO-d_6)

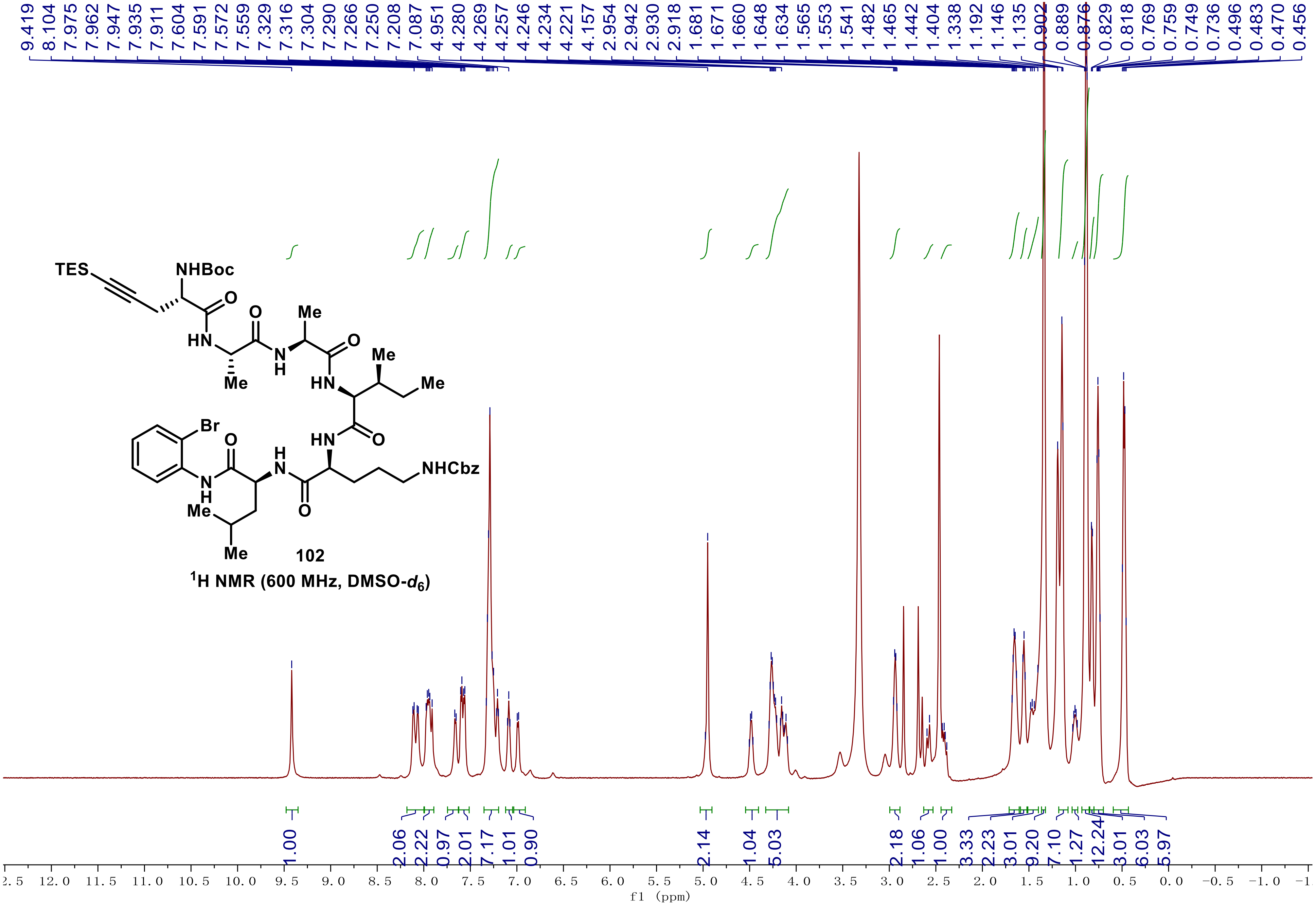
9.419 8.104 7.975 7.962 7.947 7.935 7.911 7.604 7.591 7.572 7.559 7.329 7.316 7.304 7.290 7.266 7.250 7.208 7.087 4.951 4.280 4.269 4.257 4.246 4.234 4.221 4.157 2.954 2.942 2.930 2.918 1.681 1.671 1.660 1.648 1.634 1.565 1.553 1.541 1.482 1.465 1.442 1.404 1.338 1.192 1.146 1.135 0.902 0.889 0.876 0.829 0.818 0.769 0.759 0.749 0.736 0.496 0.483 0.470 0.456
TES
NHBoc
O
HN
Me
N
H
HN
Br
NHCbz
Me
102
^{1}H NMR (600 MHz, DMSO-d_6)
1.00 2.06 2.22 0.97 2.01 7.17 1.01 0.90 2.14 1.04 5.03 2.18 1.06 1.00 3.33 2.23 3.01 9.20 7.10 1.27 12.24 3.01 6.03 5.97
f1 (ppm)

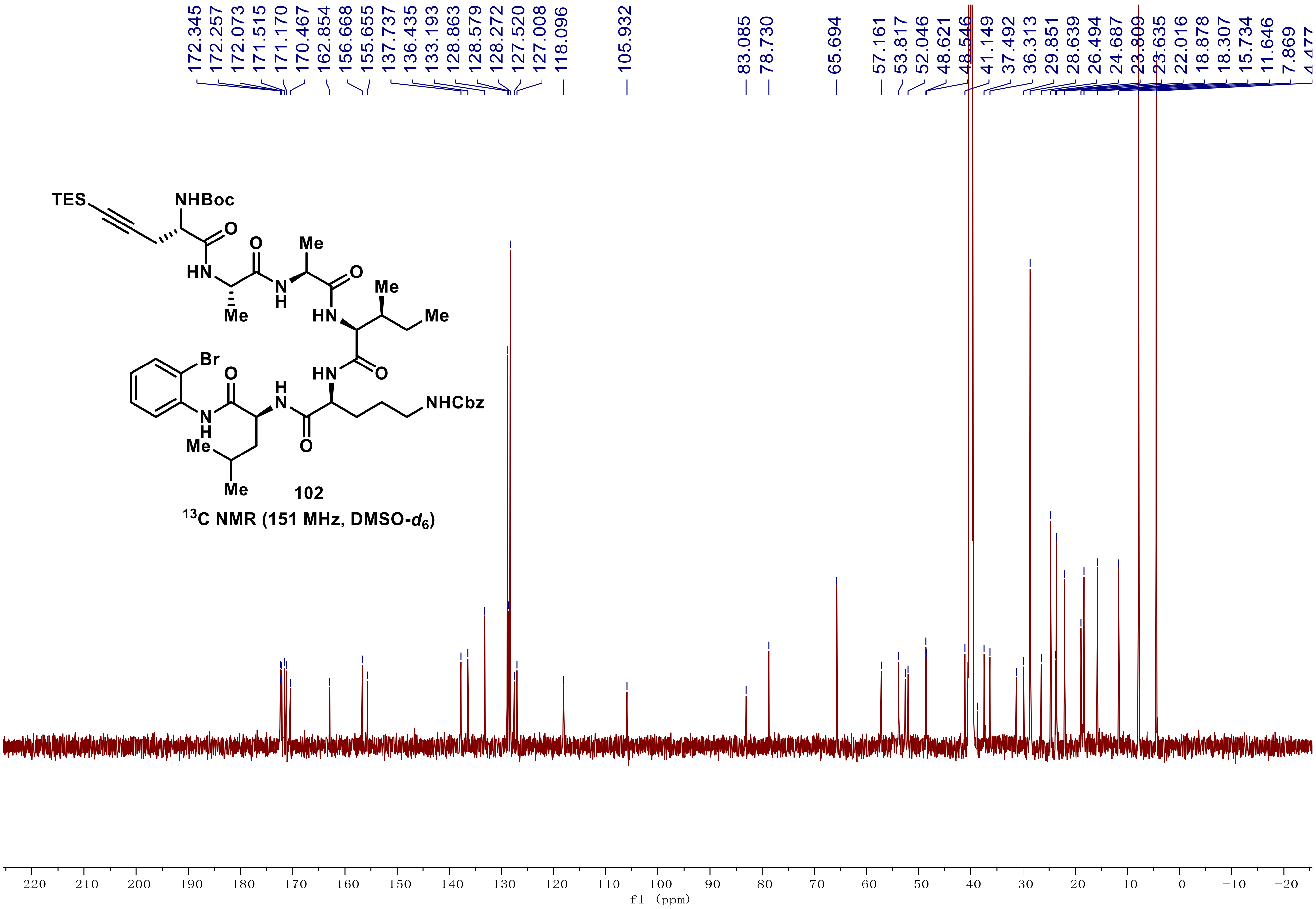
172.345
172.257
172.073
171.515
171.170
170.467
162.854
156.668
155.655
137.737
136.435
133.193
128.863
128.579
128.272
127.520
127.008
118.096
105.932
83.085
78.730
65.694
57.161
53.817
52.046
48.621
48.546
41.149
37.492
36.313
29.851
28.639
26.494
24.687
23.809
23.635
22.016
18.878
18.307
15.734
11.646
7.869
4.477
TES
NHBoc
O
HN
Me
N
H
HN
Br
NHCbz
102
^{13}C NMR (151 MHz, DMSO-d_6)
220 210 200 190 180 170 160 150 140 130 120 110 100 90 80 70 60 50 40 30 20 10 0 −10 −20
f1 (ppm)

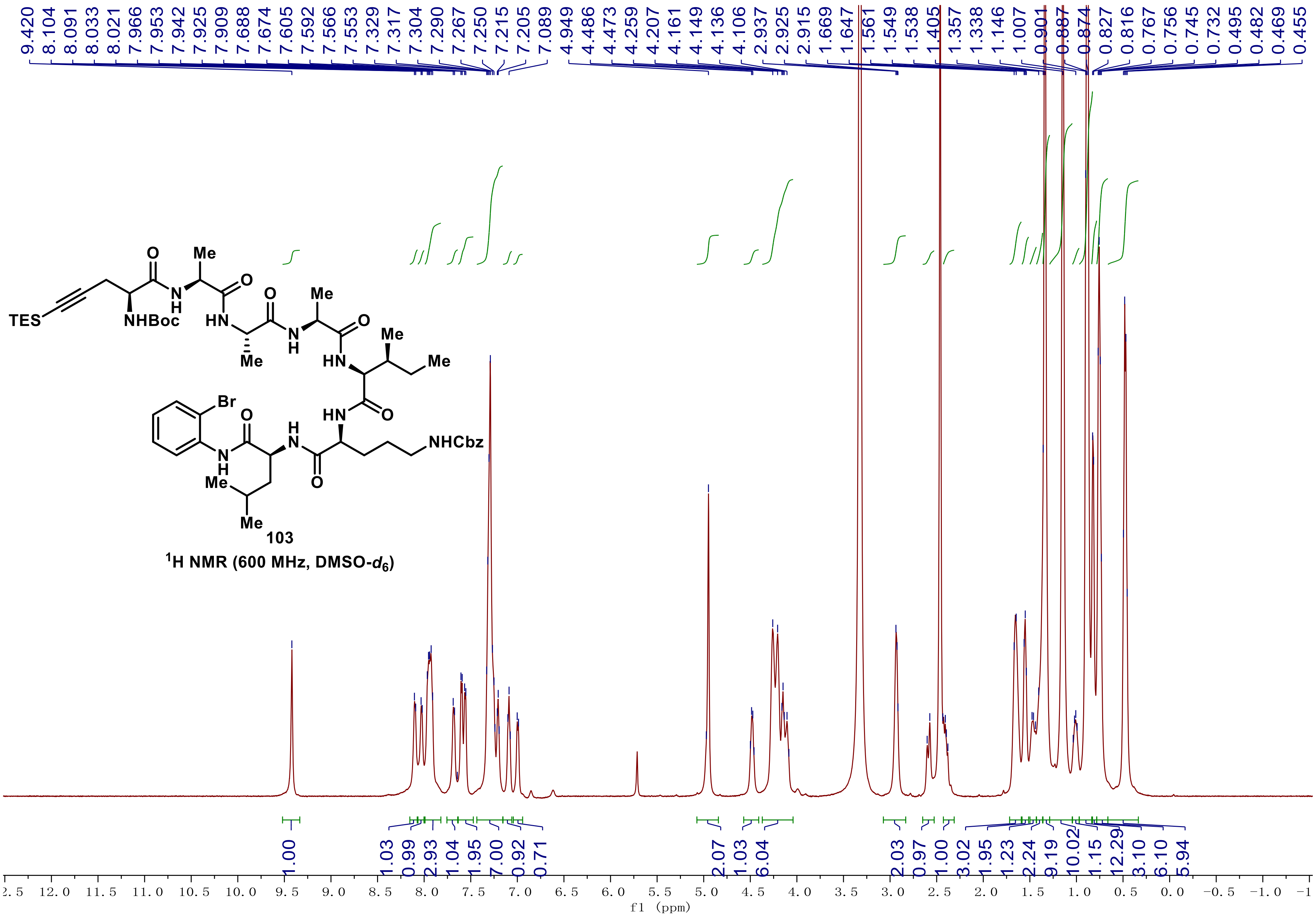

TES
NHBoc
NHCbz
Br
103
^{1}H NMR (600 MHz, DMSO-d_6)
f1 (ppm)

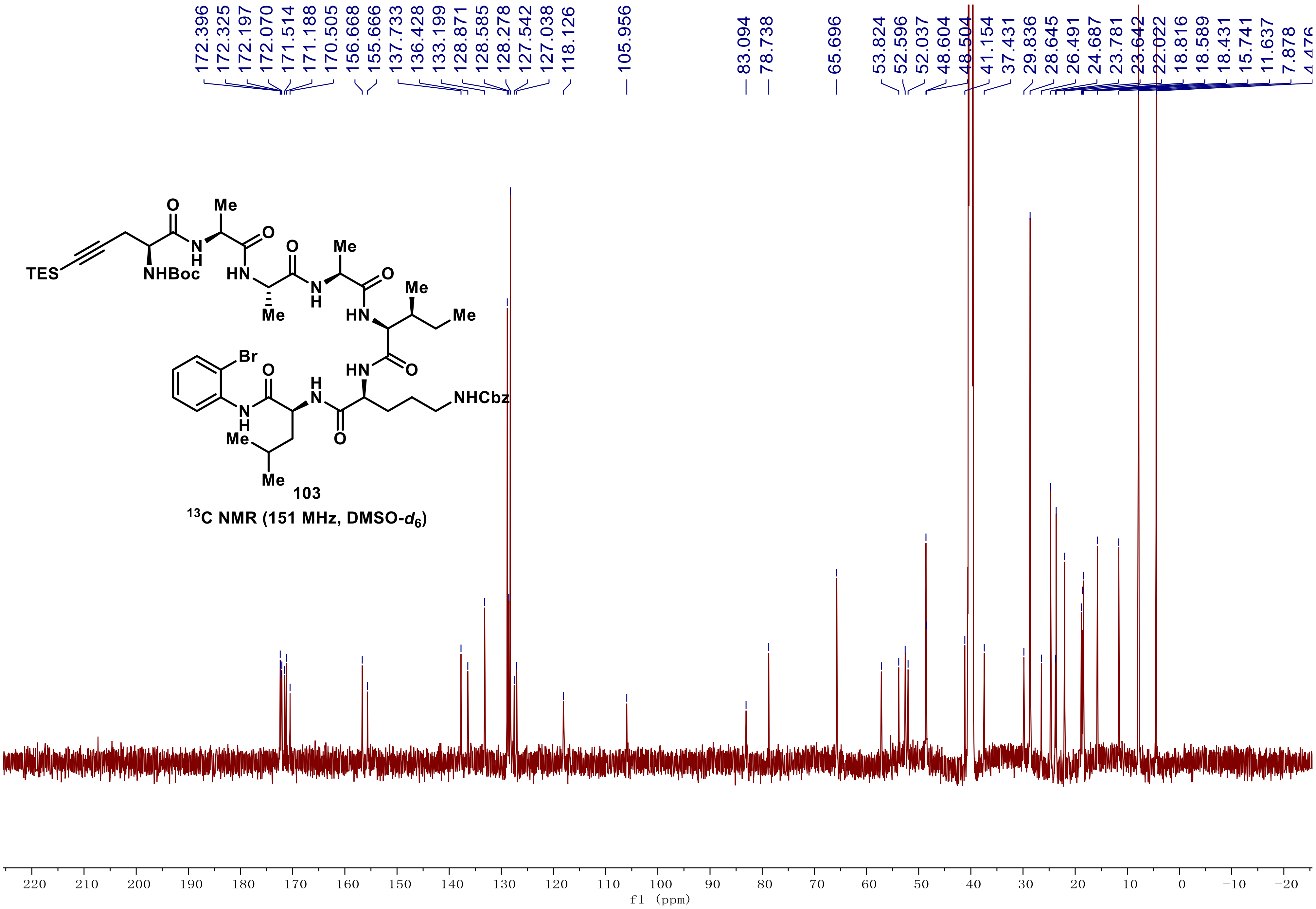

103

^{13}C NMR (151 MHz, DMSO-d_6)

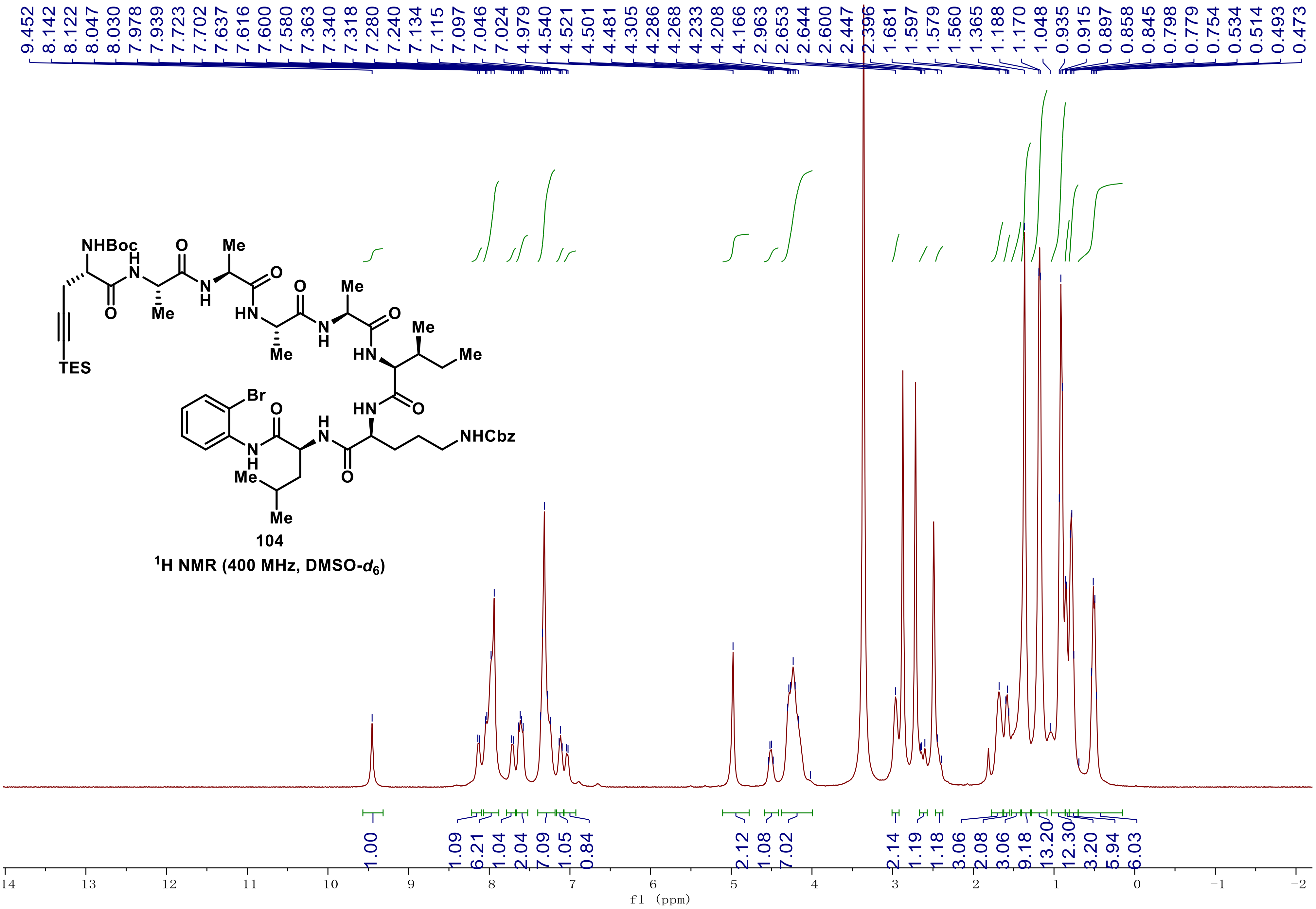
9.452 8.142 8.122 8.047 8.030 7.978 7.939 7.723 7.702 7.637 7.616 7.600 7.580 7.363 7.340 7.318 7.280 7.240 7.134 7.115 7.097 7.046 7.024 4.979 4.540 4.521 4.501 4.481 4.305 4.286 4.268 4.233 4.208 4.166 2.963 2.653 2.644 2.600 2.447 2.396 1.681 1.597 1.579 1.560 1.365 1.188 1.170 1.048 0.935 0.915 0.897 0.858 0.845 0.798 0.779 0.754 0.534 0.514 0.493 0.473
NHBoc
O
Me
H
N
O
O
Me
TES
Me
HN
N
H
Me
Me
Br
HN
Me
NHCbz
Me
Me
104
1H NMR (400 MHz, DMSO-d6)
1.00 1.09 6.21 1.04 2.04 7.09 1.05 0.84 2.12 1.08 7.02 2.14 1.19 1.18 3.06 2.08 3.06 9.18 13.20 12.30 3.20 5.94 6.03
14 13 12 11 10 9 8 7 6 5 4 3 2 1 0 −1 −2
f1 (ppm)

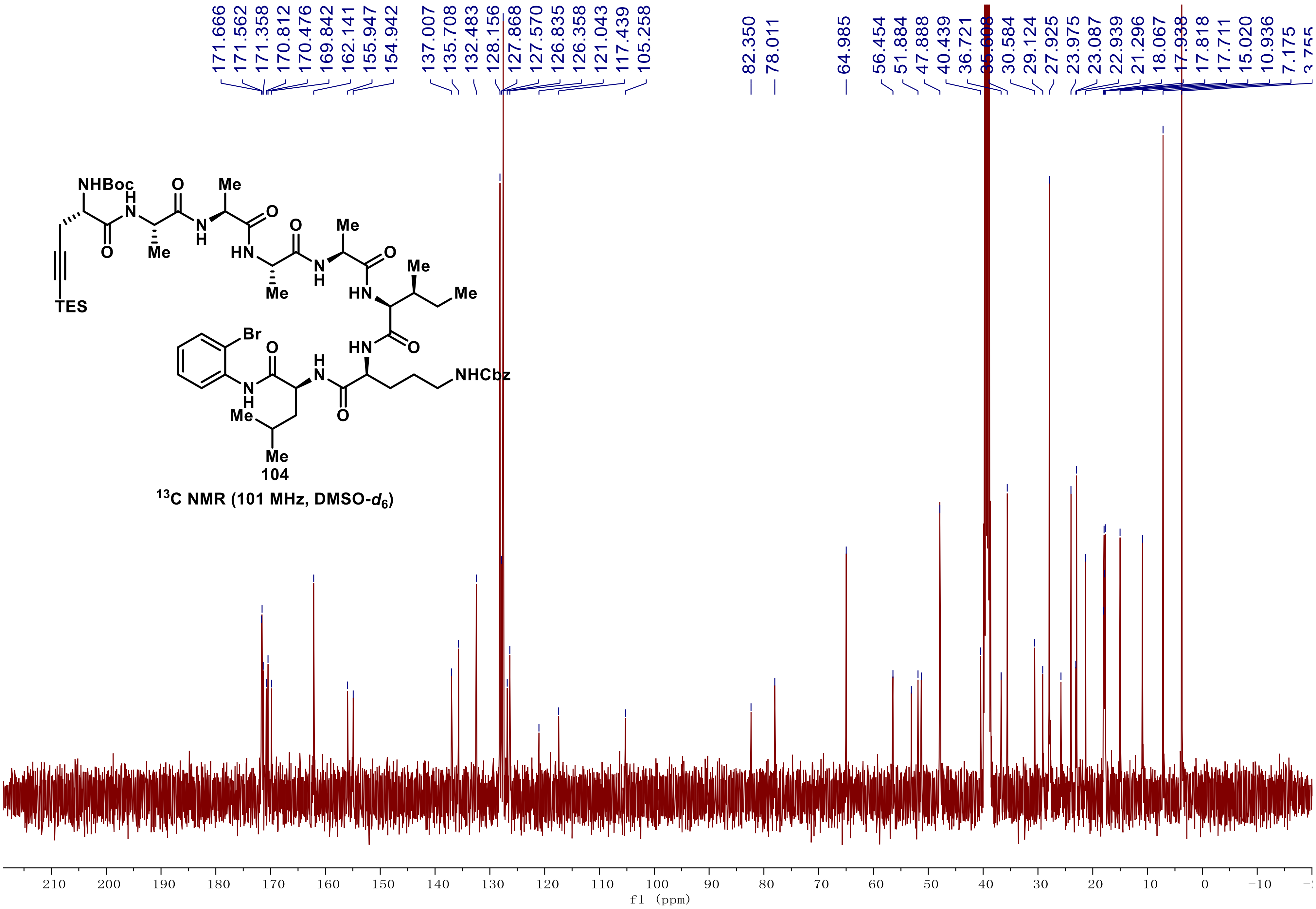

104

^{13}C NMR (101 MHz, DMSO-d_6)

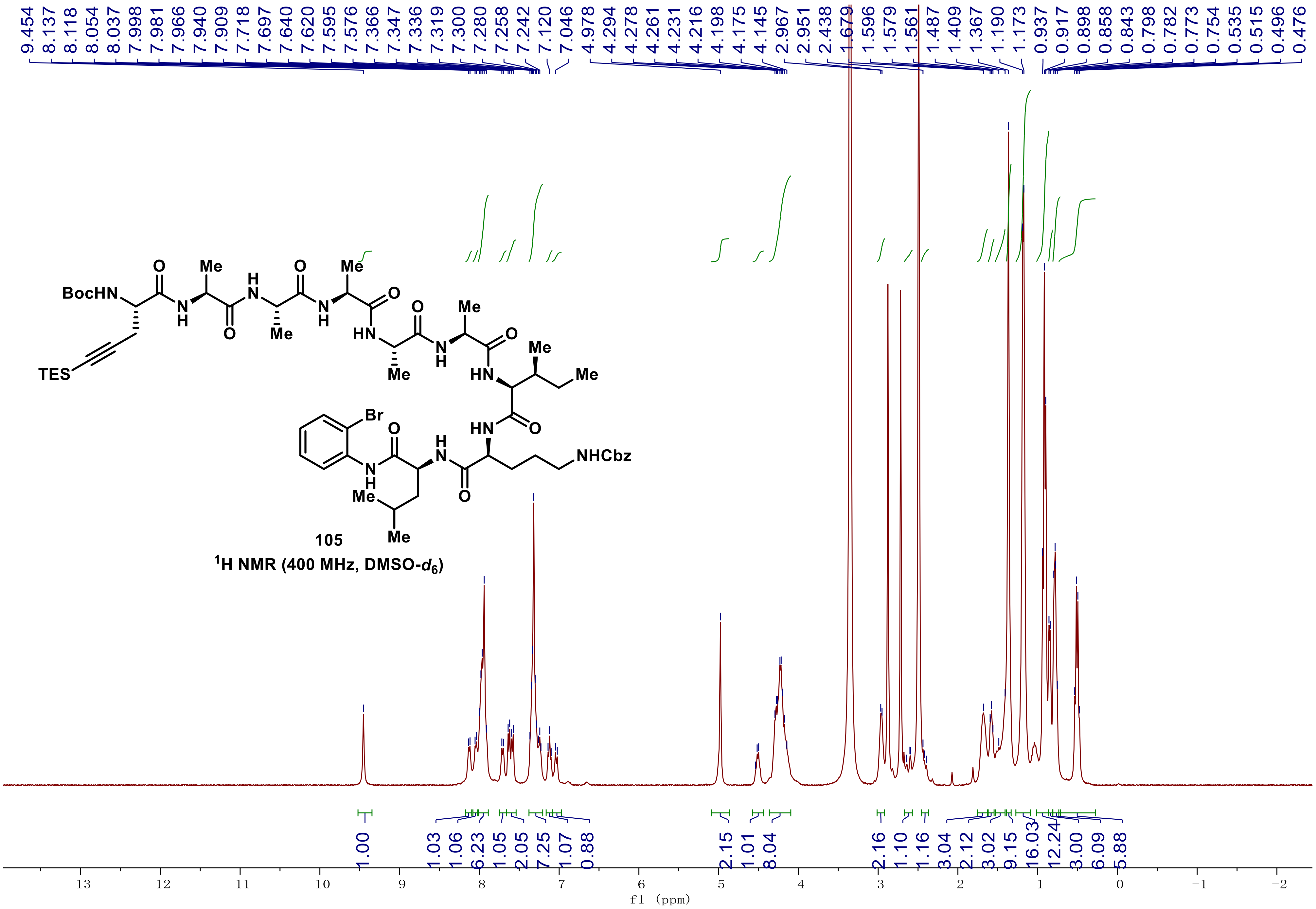

105

^{1}H NMR (400 MHz, DMSO-d_6)

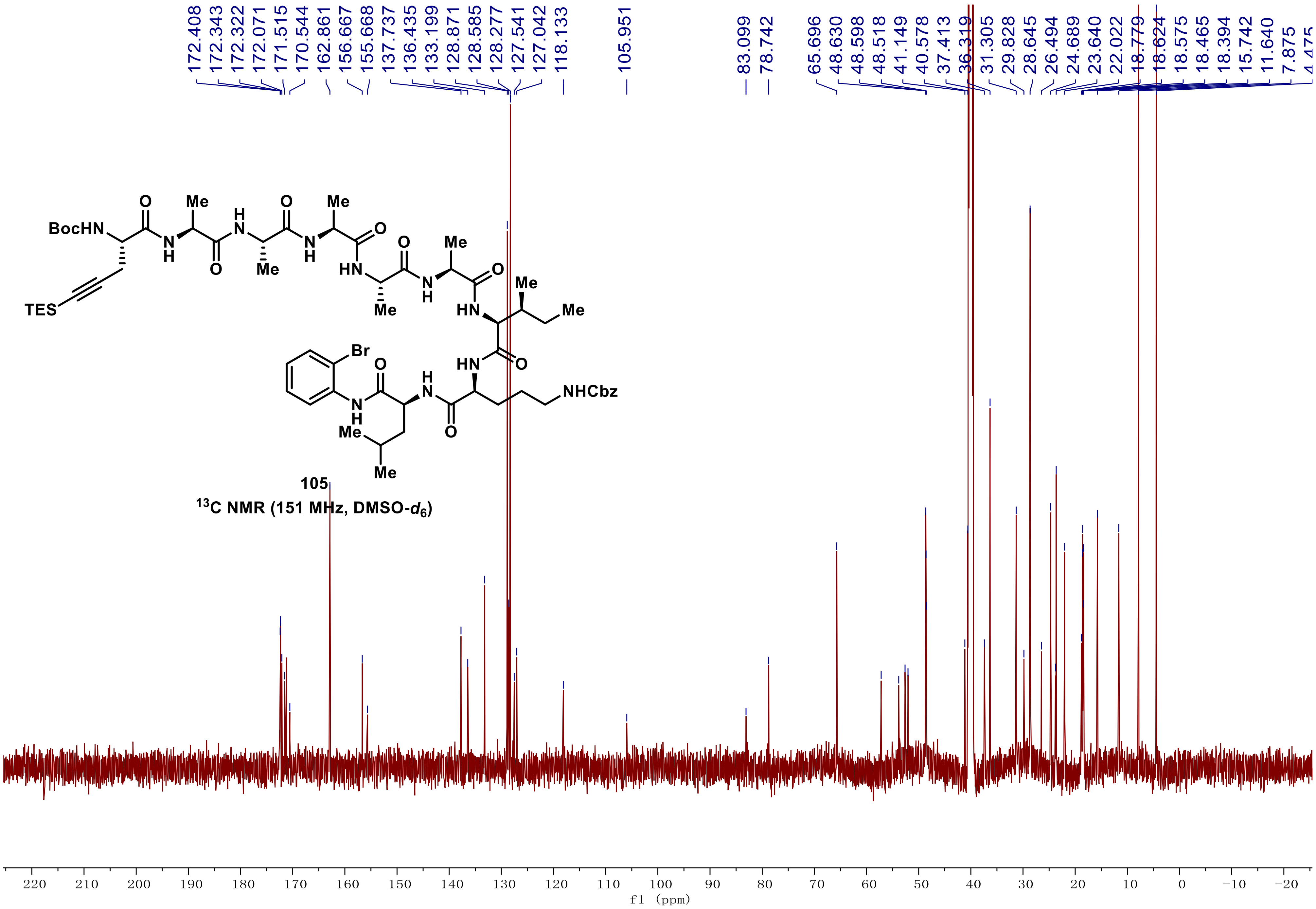

105

^{13}C NMR (151 MHz, DMSO-d_6)

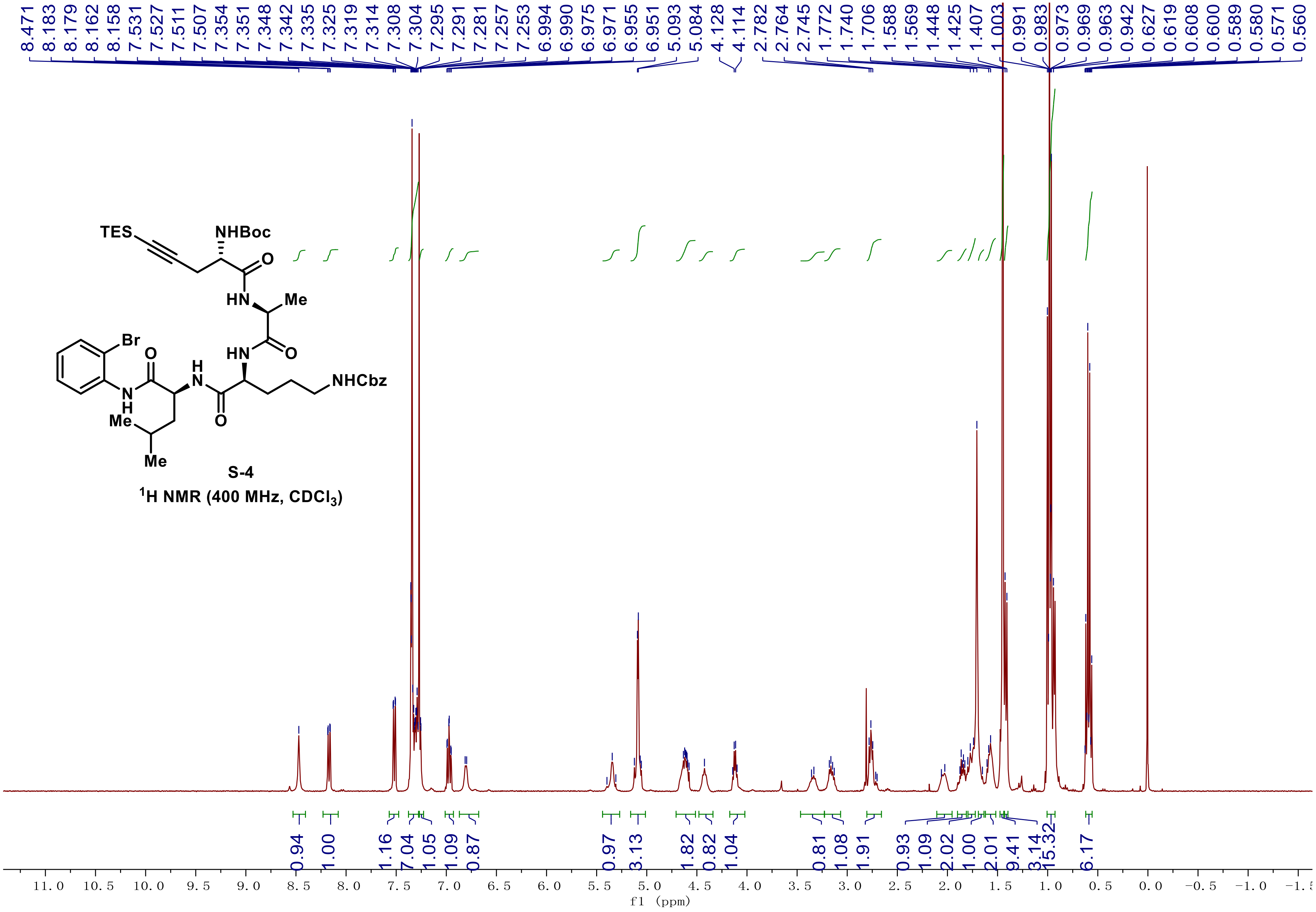
8.471 8.183 8.179 8.162 8.158 7.531 7.527 7.511 7.507 7.354 7.351 7.348 7.342 7.335 7.325 7.319 7.314 7.308 7.304 7.295 7.291 7.281 7.257 7.253 6.994 6.990 6.975 6.971 6.955 6.951 5.093 5.084 4.128 4.114 2.782 2.764 2.745 1.772 1.740 1.706 1.588 1.569 1.448 1.425 1.407 1.003 0.991 0.983 0.973 0.969 0.963 0.942 0.627 0.619 0.608 0.600 0.589 0.580 0.571 0.560
TES
NHBoc
O
HN
Me
Br
O
H
N
HN
O
NHCbz
N
H
Me
O
Me
S-4
1H NMR (400 MHz, CDCl3)
0.94 1.00 1.16 7.04 1.05 1.09 0.87 0.97 3.13 1.82 0.82 1.04 0.81 1.08 1.91 0.93 1.09 2.02 1.00 2.01 9.41 3.14 15.32 6.17
11.0 10.5 10.0 9.5 9.0 8.5 8.0 7.5 7.0 6.5 6.0 5.5 5.0 4.5 4.0 3.5 3.0 2.5 2.0 1.5 1.0 0.5 0.0 -0.5 -1.0
f1 (ppm)

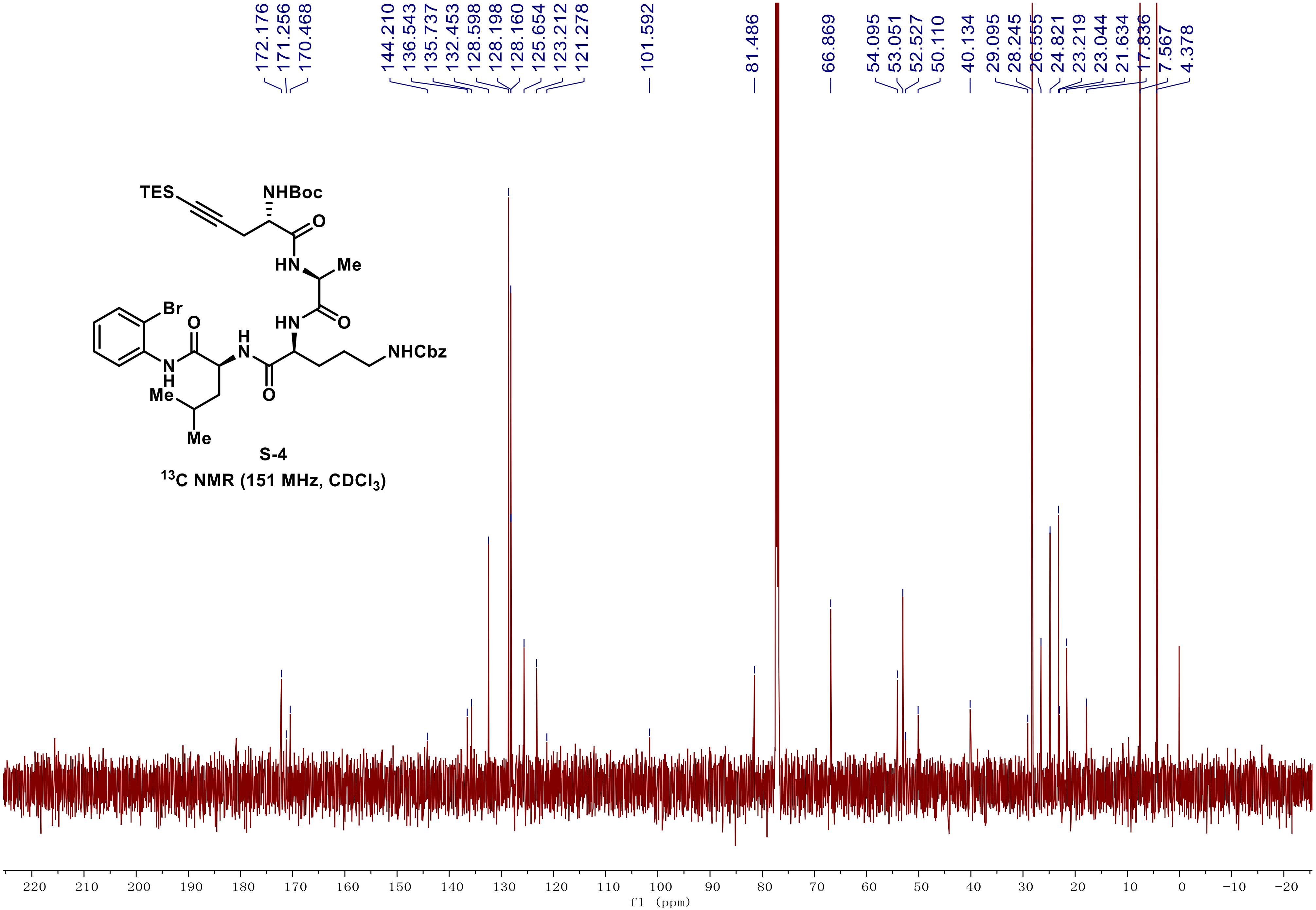

S-4

^{13}C NMR (151 MHz, $CDCl_3$)

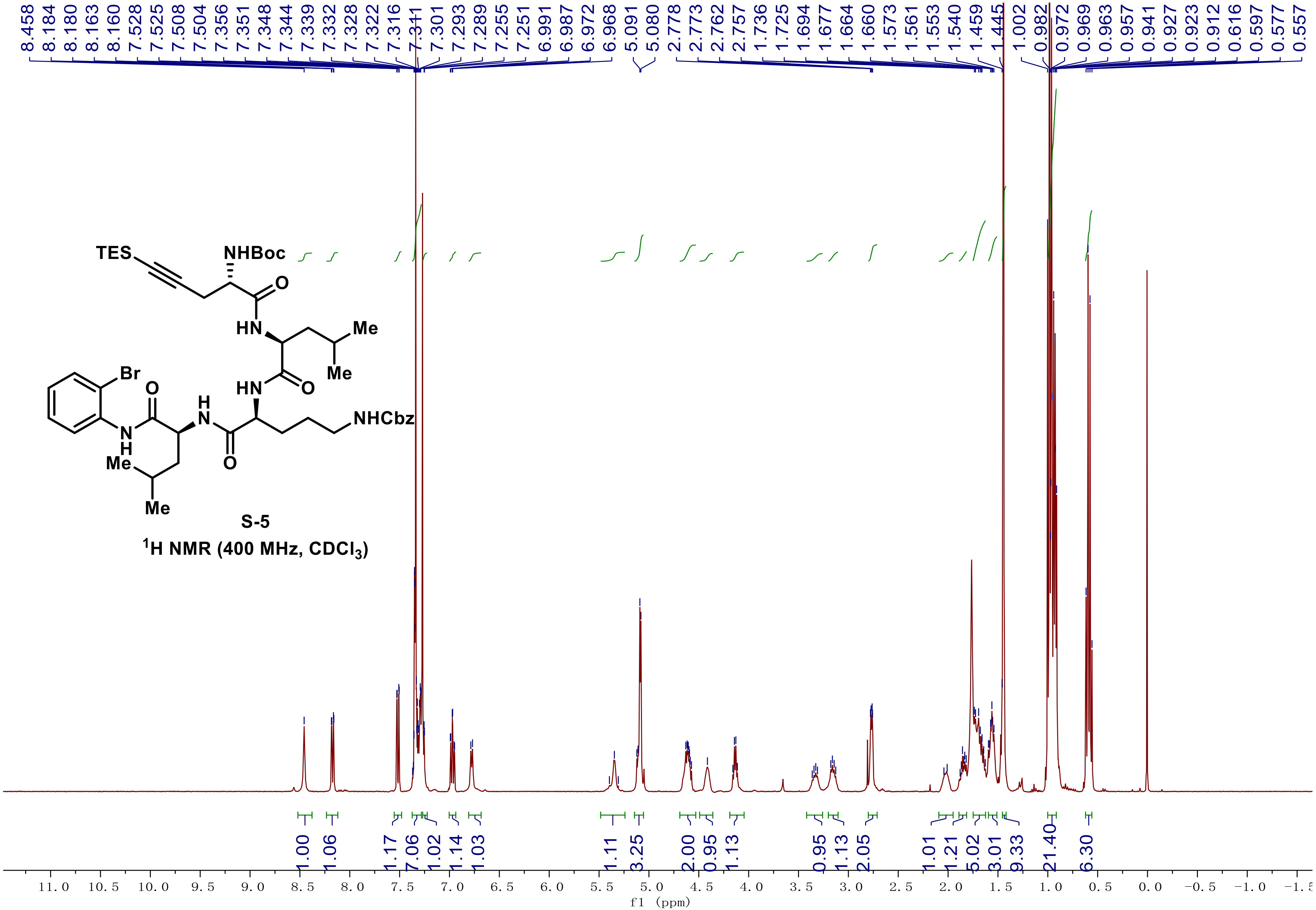

S-5
¹H NMR (400 MHz, CDCl₃)
f1 (ppm)

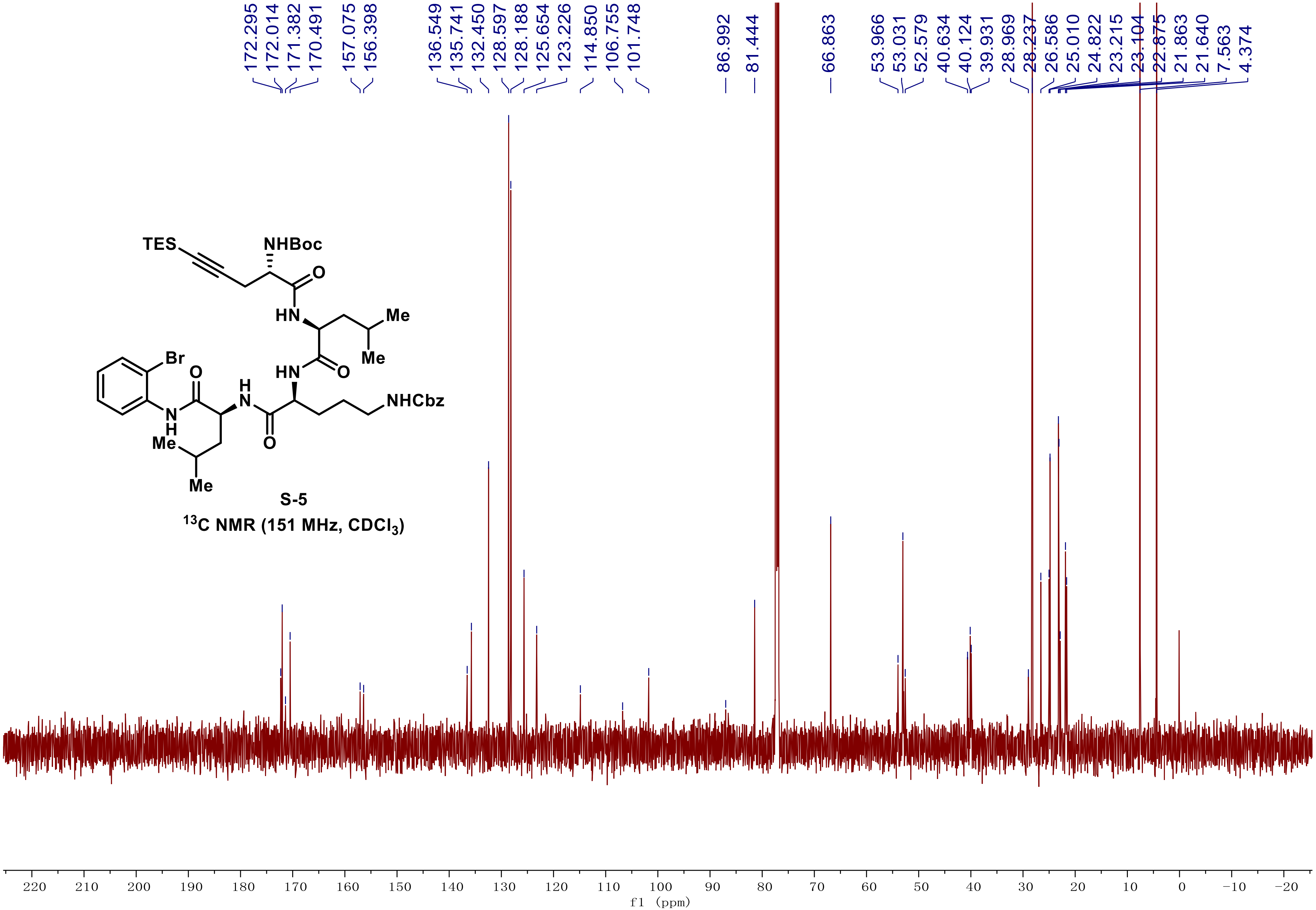
172.295
172.014
171.382
170.491
157.075
156.398
136.549
135.741
132.450
128.597
128.188
125.654
123.226
114.850
106.755
101.748
86.992
81.444
66.863
53.966
53.031
52.579
40.634
40.124
39.931
28.969
28.237
26.586
25.010
24.822
23.215
23.104
22.875
21.863
21.640
7.563
4.374
TES
NHBoc
O
HN
Me
Me
Br
O
H
N
HN
O
NHCbz
N
H
Me
O
Me
S-5
^{13}C NMR (151 MHz, $CDCl_3$)
f1 (ppm)

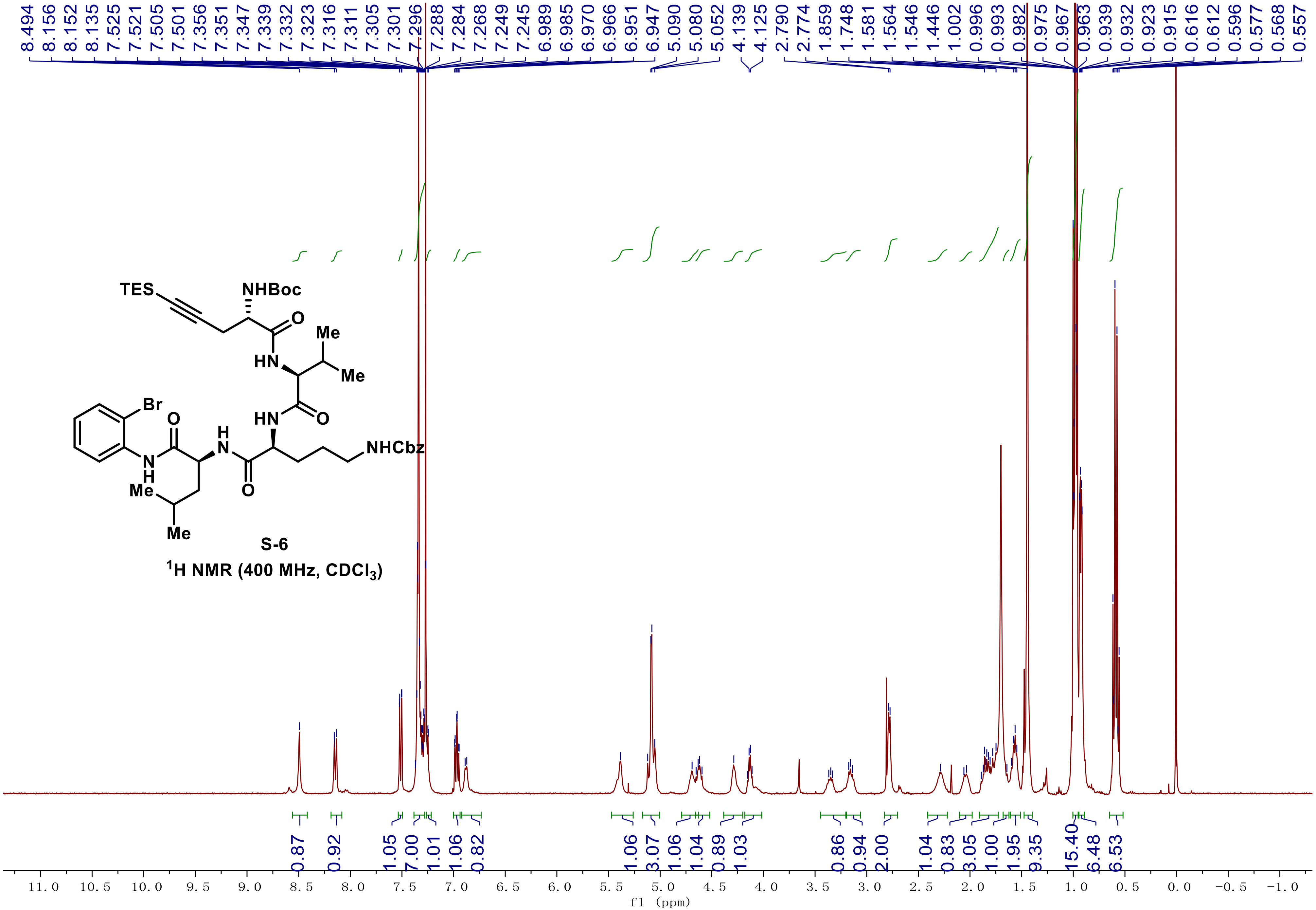

8.494 8.156 8.152 8.135 7.525 7.521 7.505 7.501 7.356 7.351 7.347 7.339 7.332 7.323 7.316 7.311 7.305 7.301 7.296 7.288 7.284 7.268 7.249 7.245 6.989 6.985 6.970 6.966 6.951 6.947 5.090 5.080 5.052 4.139 4.125 2.790 2.774 1.859 1.748 1.581 1.564 1.546 1.446 1.002 0.996 0.993 0.982 0.975 0.967 0.963 0.939 0.932 0.923 0.915 0.616 0.612 0.596 0.577 0.568 0.557
TES
NHBoc
O
Me
HN
Me
Br
O
H
N
HN
O
NHCbz
N
H
Me
O
Me
S-6
¹H NMR (400 MHz, CDCl₃)
0.87 0.92 1.05 7.00 1.01 1.06 0.82 1.06 3.07 1.06 1.04 0.89 1.03 0.86 0.94 2.00 1.04 0.83 3.05 1.00 1.95 9.35 15.40 6.48 6.53
11.0 10.5 10.0 9.5 9.0 8.5 8.0 7.5 7.0 6.5 6.0 5.5 5.0 4.5 4.0 3.5 3.0 2.5 2.0 1.5 1.0 0.5 0.0 −0.5 −1.0
f1 (ppm)

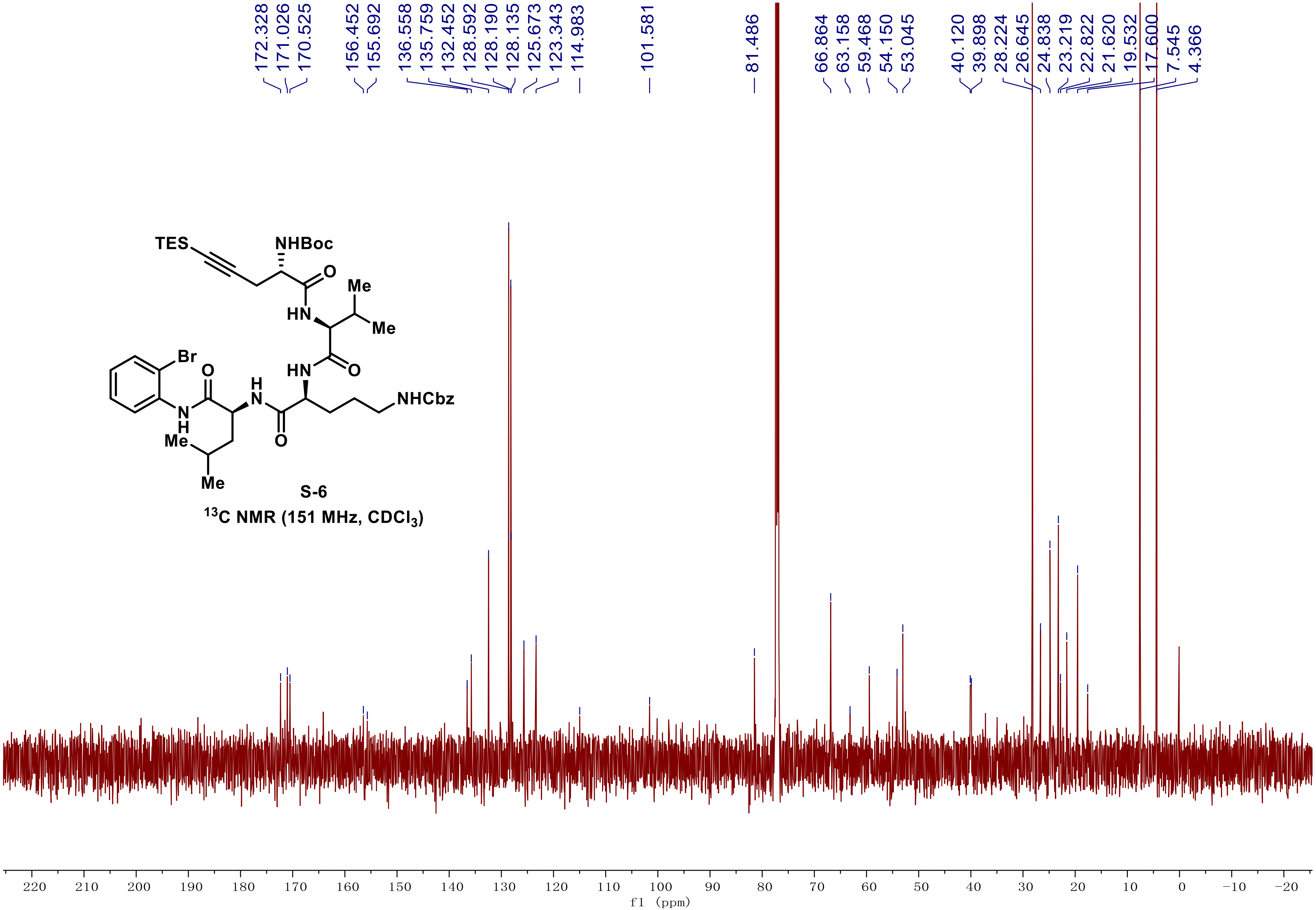
172.328
171.026
170.525
156.452
155.692
136.558
135.759
132.452
128.592
128.190
128.135
125.673
123.343
114.983
101.581
81.486
66.864
63.158
59.468
54.150
53.045
40.120
39.898
28.224
26.645
24.838
23.219
22.822
21.620
19.532
17.600
7.545
4.366
TES
NHBoc
O
Me
HN
Me
Br
O
HN
O
H
N
NHCbz
N
H
Me
O
Me
S-6
13C NMR (151 MHz, CDCl3)
f1 (ppm)

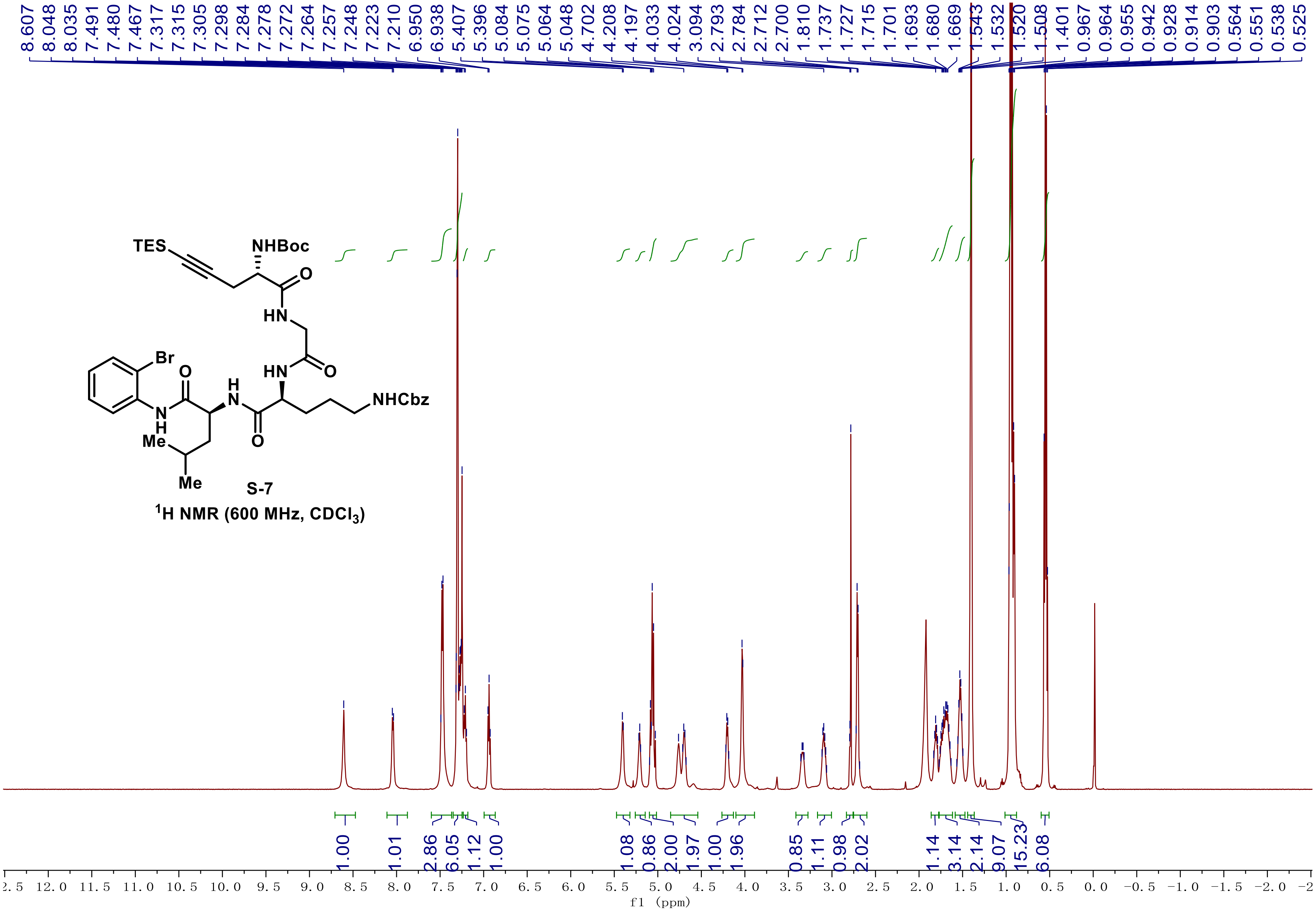

TES
NHBoc
O
HN
Br
O
H
N
HN
O
NHCbz
N
H
Me
O
Me
S-7
^{1}H NMR (600 MHz, CDCl$_3$)
f1 (ppm)

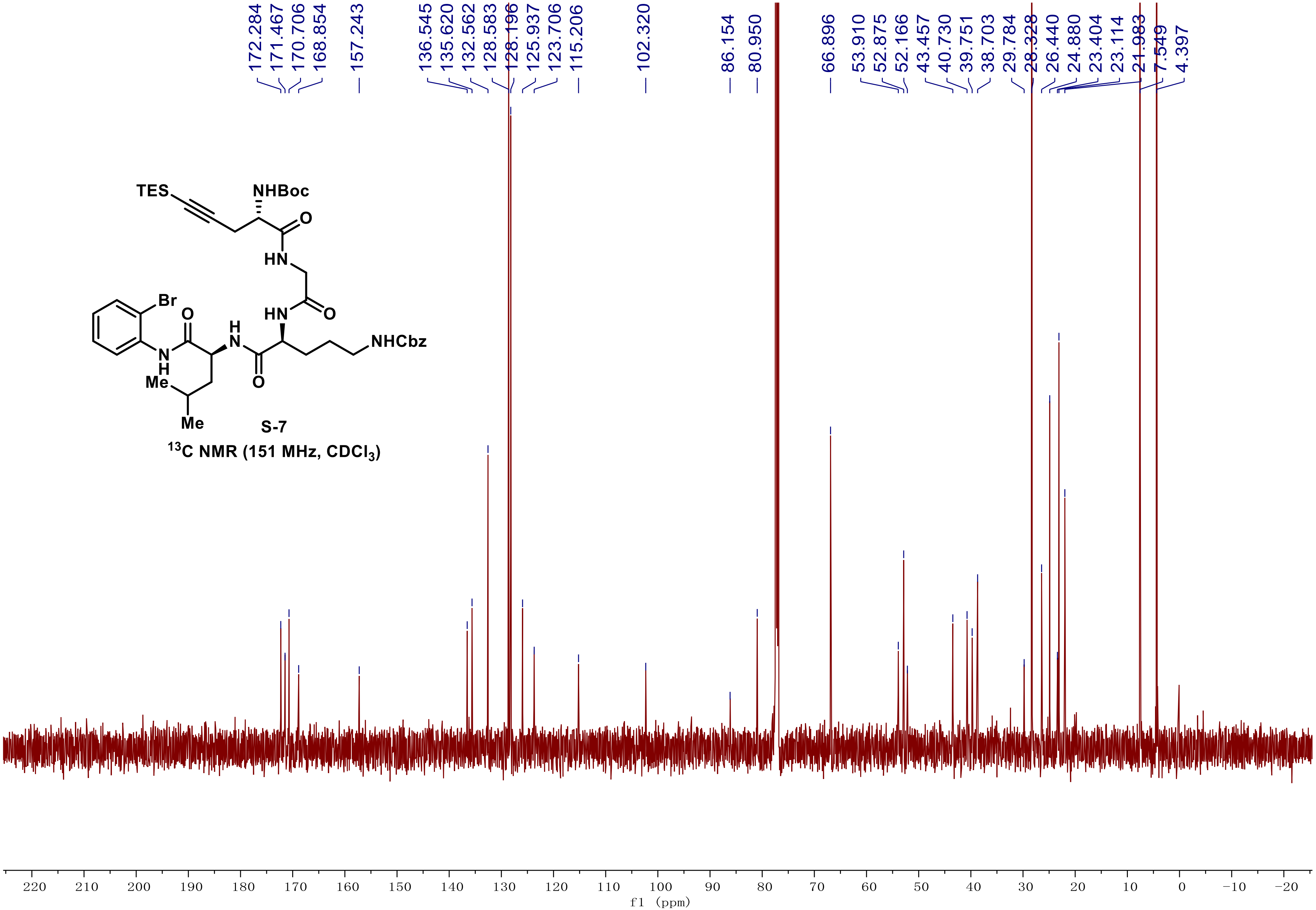
172.284
171.467
170.706
168.854
157.243
136.545
135.620
132.562
128.583
128.196
125.937
123.706
115.206
102.320
86.154
80.950
66.896
53.910
52.875
52.166
43.457
40.730
39.751
38.703
29.784
28.328
26.440
24.880
23.404
23.114
21.983
7.549
4.397
TES
NHBoc
O
HN
Br
O
HN
O
H
N
NHCbz
N
H
Me
O
Me
S-7
13C NMR (151 MHz, CDCl3)
f1 (ppm)

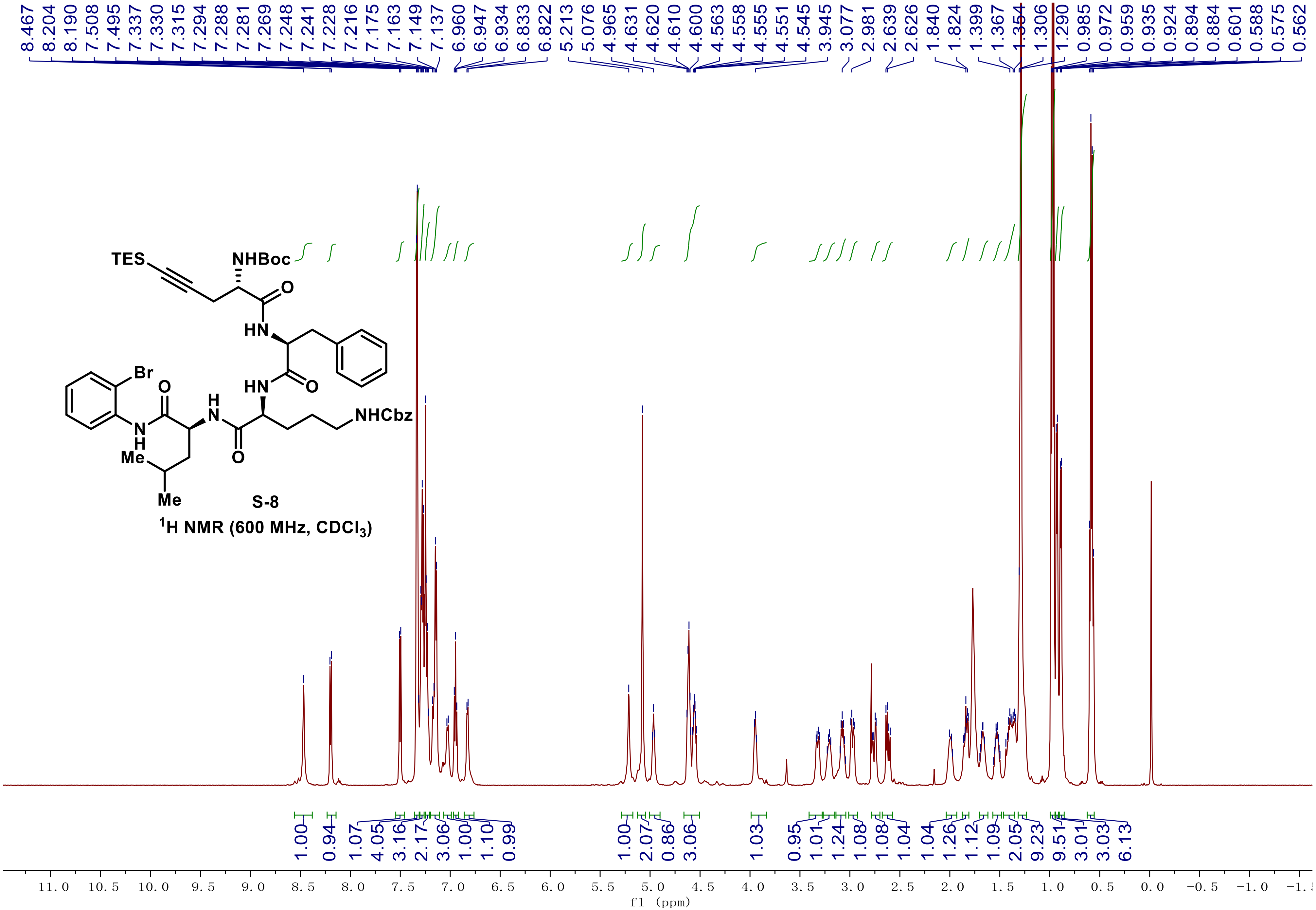

TES
NHBoc
HN
HN
NHCbz
Br
Me
Me
S-8
¹H NMR (600 MHz, CDCl₃)
f1 (ppm)

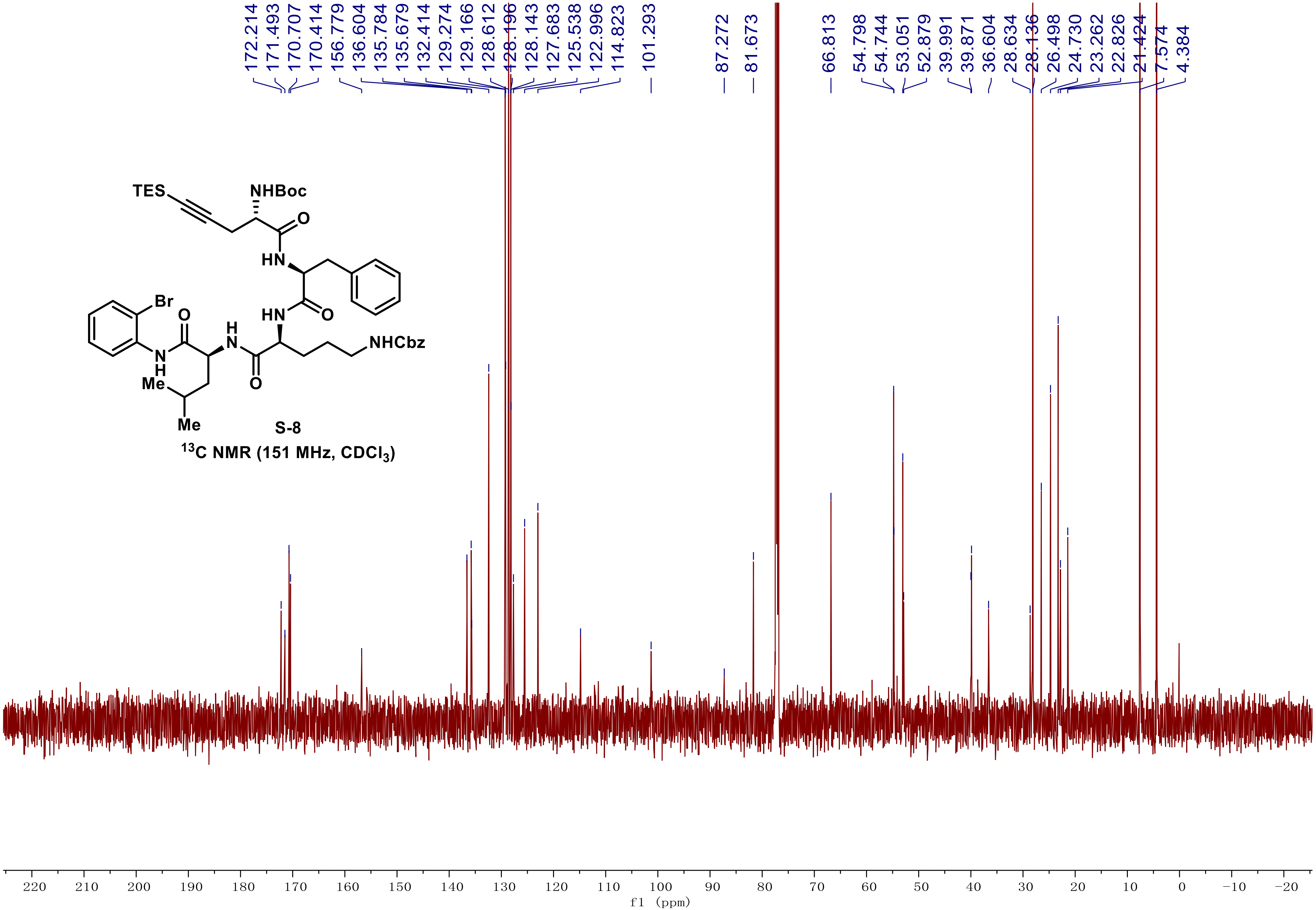
172.214
171.493
170.707
170.414
156.779
136.604
135.784
135.679
132.414
129.274
129.166
128.612
128.196
128.143
127.683
125.538
122.996
114.823
101.293
87.272
81.673
66.813
54.798
54.744
53.051
52.879
39.991
39.871
36.604
28.634
28.136
26.498
24.730
23.262
22.826
21.424
7.574
4.384
TES
NHBoc
O
HN
Br
O
H
N
HN
O
NHCbz
N
H
Me
O
Me
S-8
^{13}C NMR (151 MHz, $CDCl_3$)
220 210 200 190 180 170 160 150 140 130 120 110 100 90 80 70 60 50 40 30 20 10 0 −10 −20
f1 (ppm)

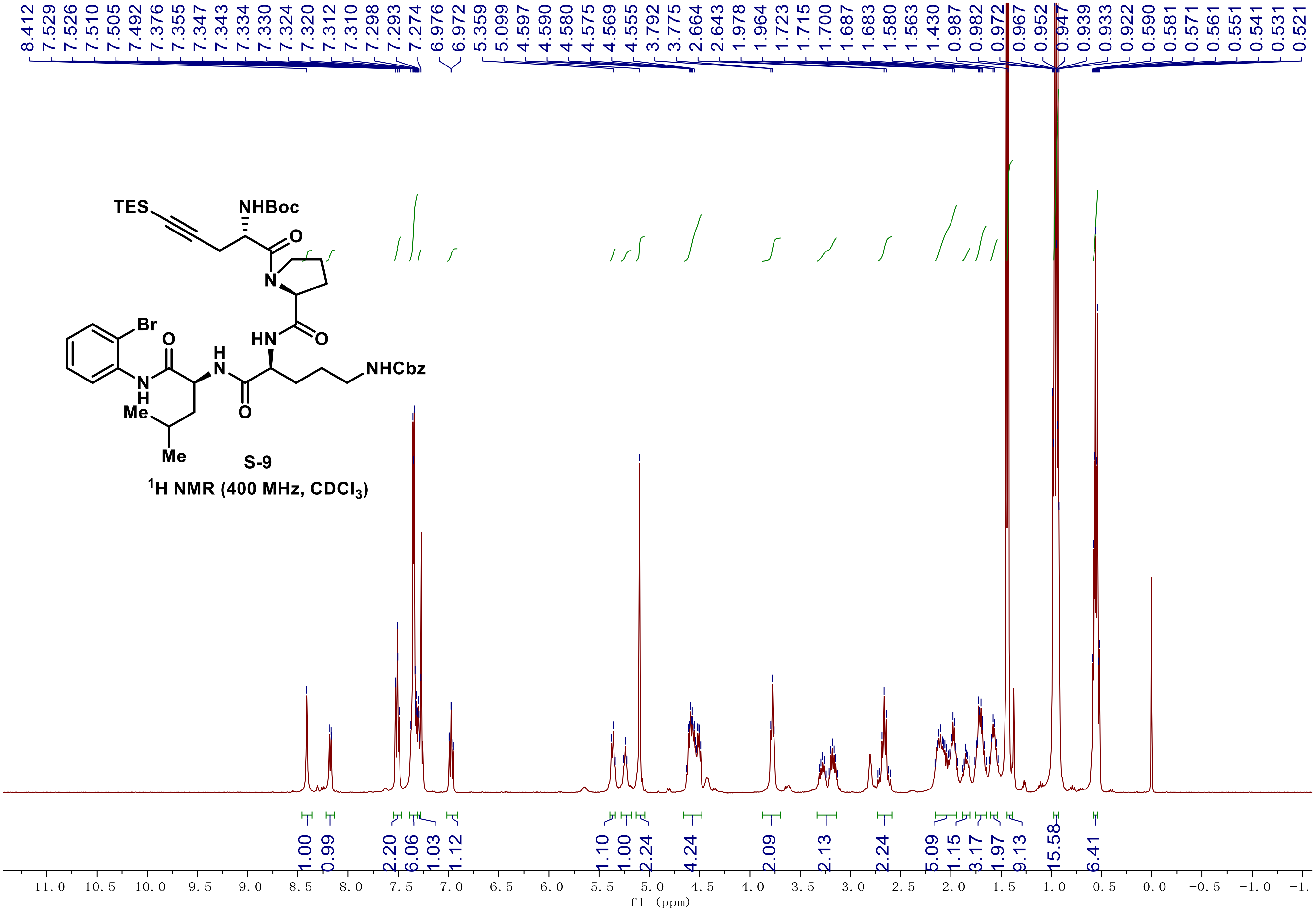
8.412 7.529 7.526 7.510 7.505 7.492 7.376 7.355 7.347 7.343 7.334 7.330 7.324 7.320 7.312 7.310 7.298 7.293 7.274 6.976 6.972 5.359 5.099 4.597 4.590 4.580 4.575 4.569 4.555 3.792 3.775 2.664 2.643 1.978 1.964 1.723 1.715 1.700 1.687 1.683 1.580 1.563 1.430 0.987 0.982 0.972 0.967 0.952 0.947 0.939 0.933 0.922 0.590 0.581 0.571 0.561 0.551 0.541 0.531 0.521
TES
NHBoc
Br
NHCbz
Me
Me
S-9
^{1}H NMR (400 MHz, CDCl$_3$)
1.00 0.99 2.20 6.06 1.03 1.12 1.10 1.00 2.24 4.24 2.09 2.13 2.24 5.09 1.15 3.17 1.97 9.13 15.58 6.41
11.0 10.5 10.0 9.5 9.0 8.5 8.0 7.5 7.0 6.5 6.0 5.5 5.0 4.5 4.0 3.5 3.0 2.5 2.0 1.5 1.0 0.5 0.0 −0.5 −1.0
f1 (ppm)

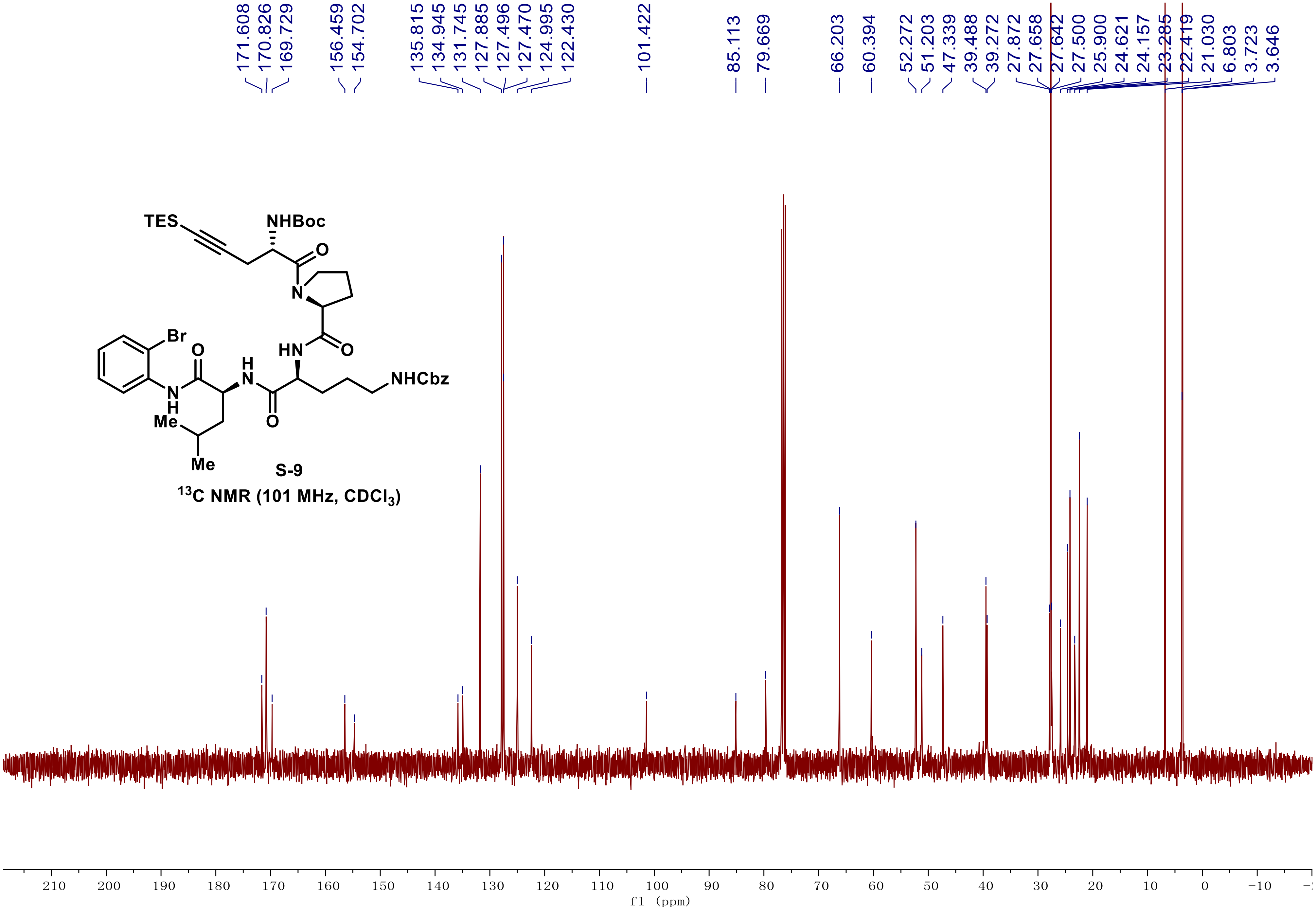
171.608
170.826
169.729
156.459
154.702
135.815
134.945
131.745
127.885
127.496
127.470
124.995
122.430
101.422
85.113
79.669
66.203
60.394
52.272
51.203
47.339
39.488
39.272
27.872
27.658
27.642
27.500
25.900
24.621
24.157
23.285
22.419
21.030
6.803
3.723
3.646
TES
NHBoc
O
N
Br
O
HN
O
H
N
NHCbz
N
H
Me
O
Me
S-9
13C NMR (101 MHz, CDCl3)
210
200
190
180
170
160
150
140
130
120
110
100
90
80
70
60
50
40
30
20
10
0
−10
f1 (ppm)

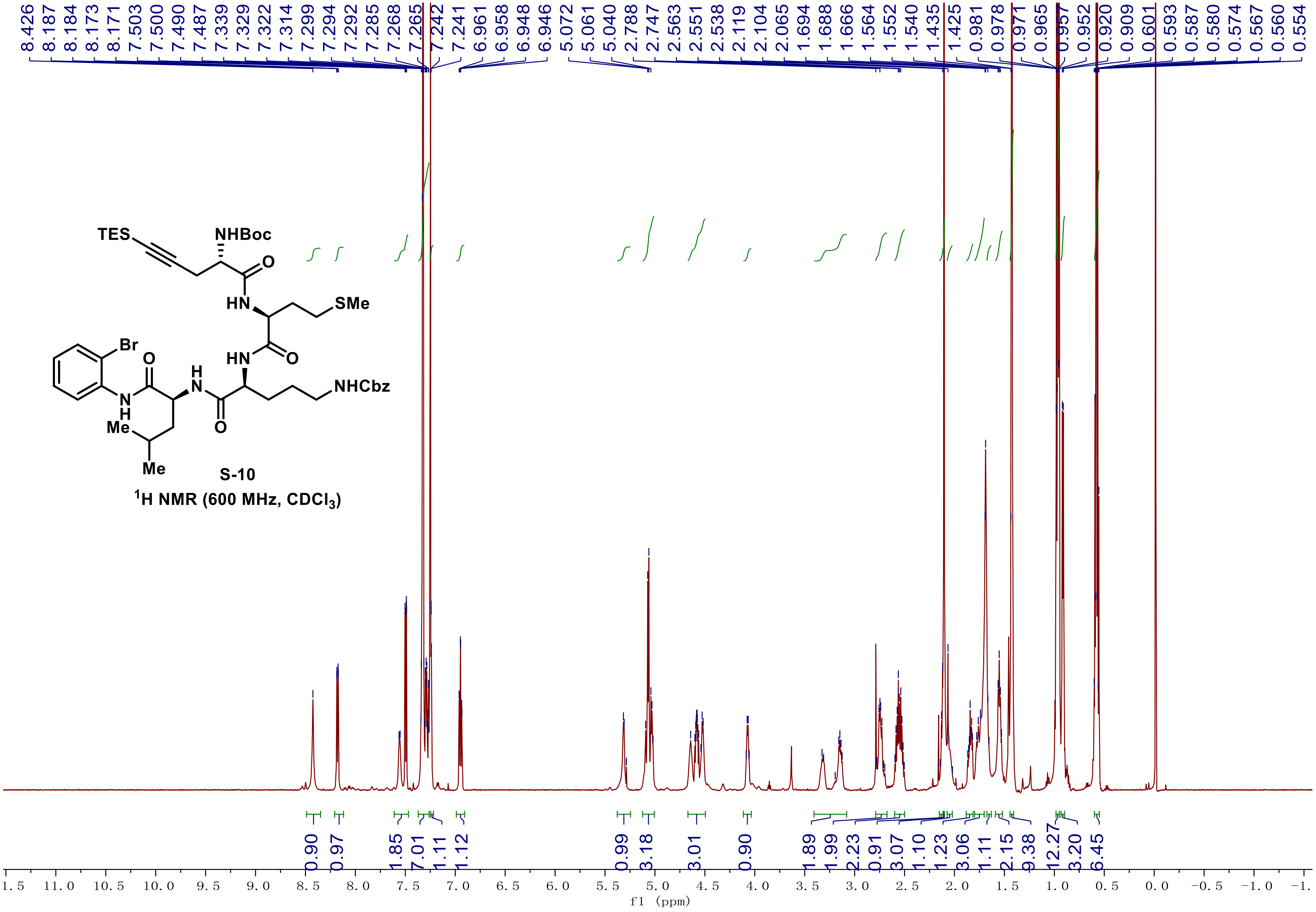

S-10

^{1}H NMR (600 MHz, $CDCl_3$)

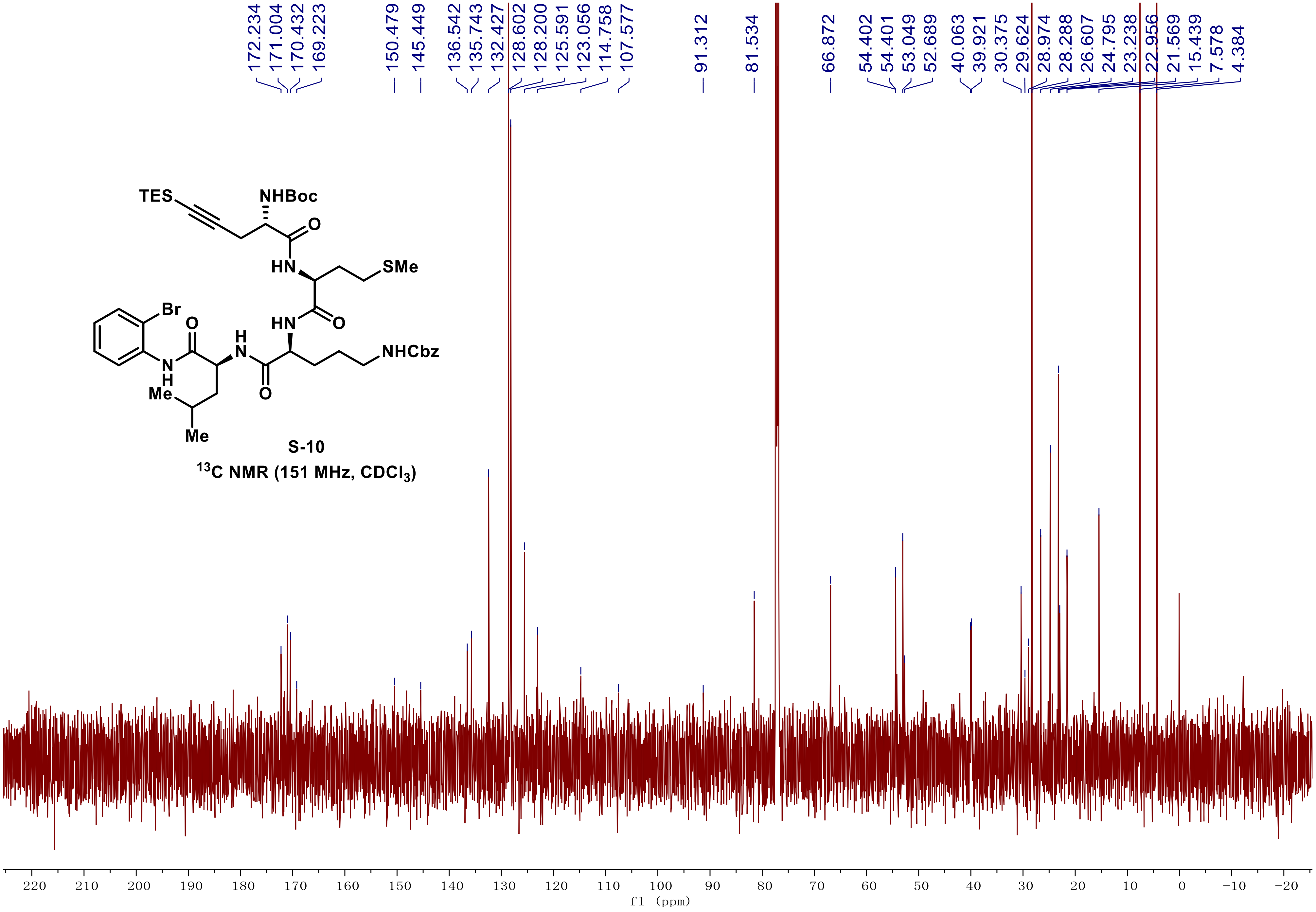

S-10

^{13}C NMR (151 MHz, CDCl$_3$)

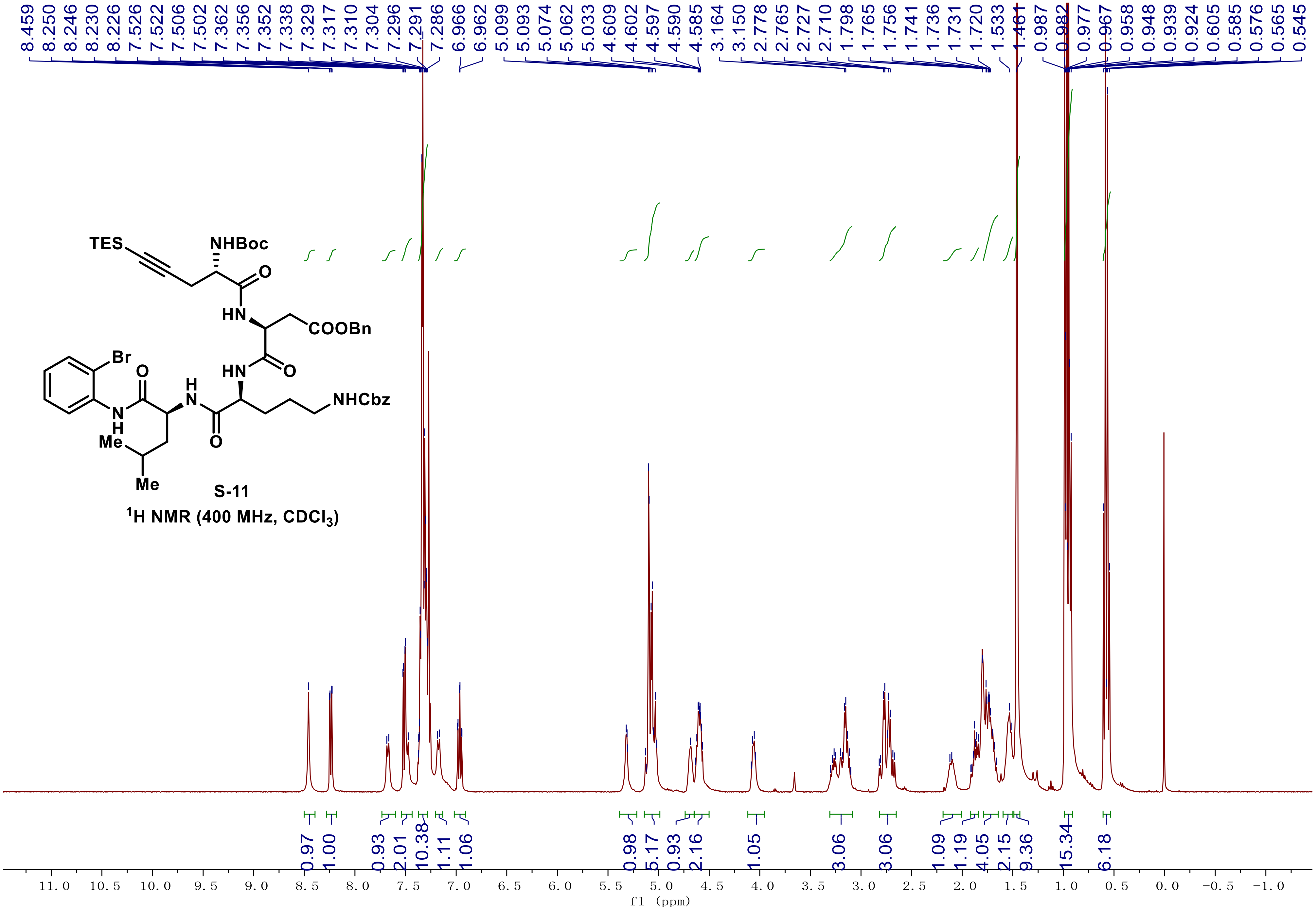
8.459 8.250 8.246 8.230 8.226 7.526 7.522 7.506 7.502 7.362 7.356 7.352 7.338 7.329 7.317 7.310 7.304 7.296 7.291 7.286 6.966 6.962 5.099 5.093 5.074 5.062 5.033 4.609 4.602 4.597 4.590 4.585 3.164 3.150 2.778 2.765 2.727 2.710 1.798 1.765 1.756 1.741 1.736 1.731 1.720 1.533 1.461 0.987 0.982 0.977 0.967 0.958 0.948 0.939 0.924 0.605 0.585 0.576 0.565 0.545
TES
NHBoc
HN
COOBn
Br
O
H
N
HN
NHCbz
H
Me
Me
S-11
^{1}H NMR (400 MHz, $CDCl_3$)
0.97 1.00 0.93 2.01 10.38 1.11 1.06 0.98 5.17 0.93 2.16 1.05 3.06 3.06 1.09 1.19 4.05 2.15 9.36 15.34 6.18
11.0 10.5 10.0 9.5 9.0 8.5 8.0 7.5 7.0 6.5 6.0 5.5 5.0 4.5 4.0 3.5 3.0 2.5 2.0 1.5 1.0 0.5 0.0 −0.5 −1.0
f1 (ppm)

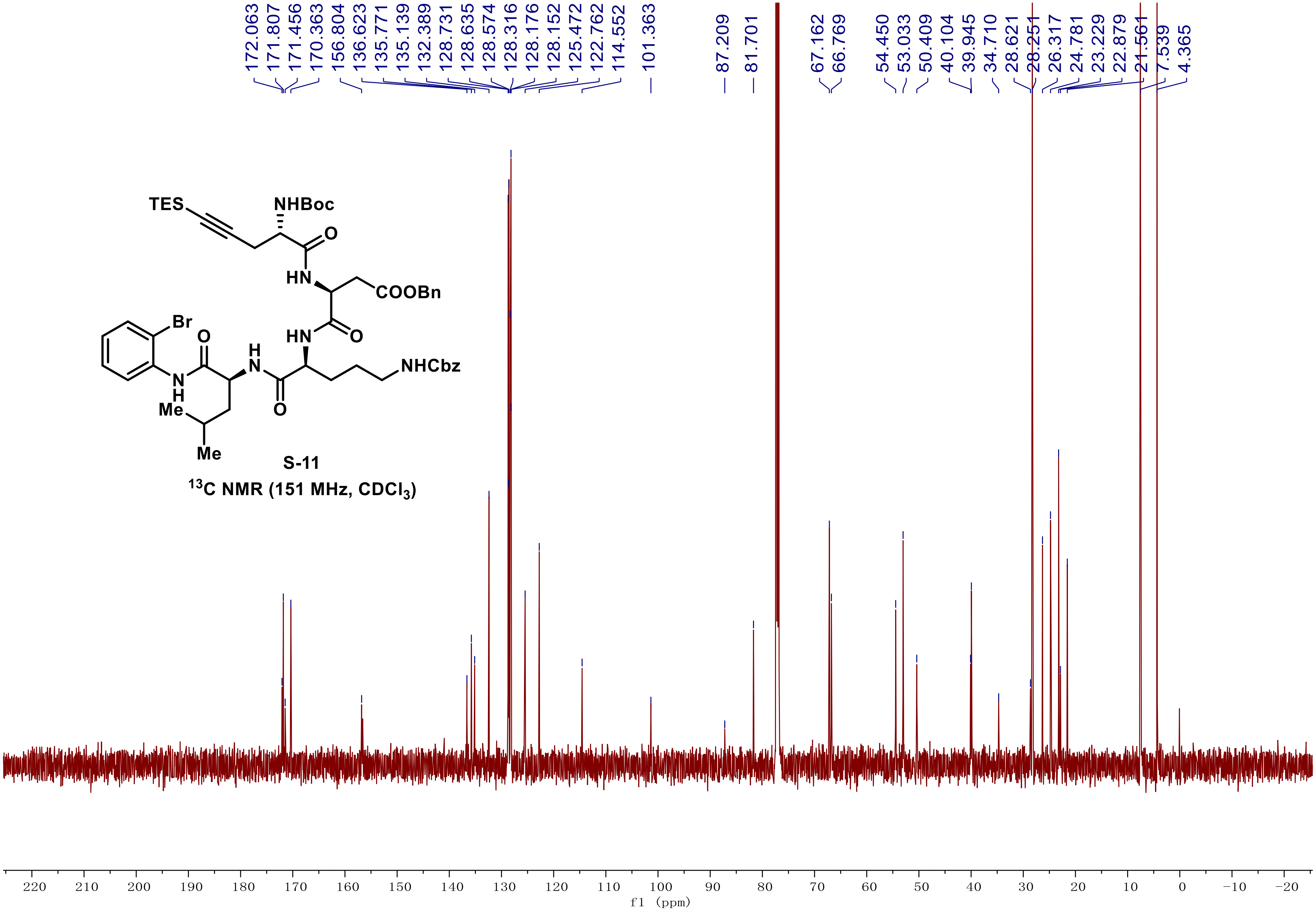
172.063
171.807
171.456
170.363
156.804
136.623
135.771
135.139
132.389
128.731
128.635
128.574
128.316
128.176
128.152
125.472
122.762
114.552
101.363
87.209
81.701
67.162
66.769
54.450
53.033
50.409
40.104
39.945
34.710
28.621
28.251
26.317
24.781
23.229
22.879
21.561
7.539
4.365
TES
NHBoc
O
HN
COOBn
Br
O
H
N
HN
O
NHCbz
N
H
Me
O
Me
S-11
13C NMR (151 MHz, CDCl3)
220 210 200 190 180 170 160 150 140 130 120 110 100 90 80 70 60 50 40 30 20 10 0 −10 −20
f1 (ppm)

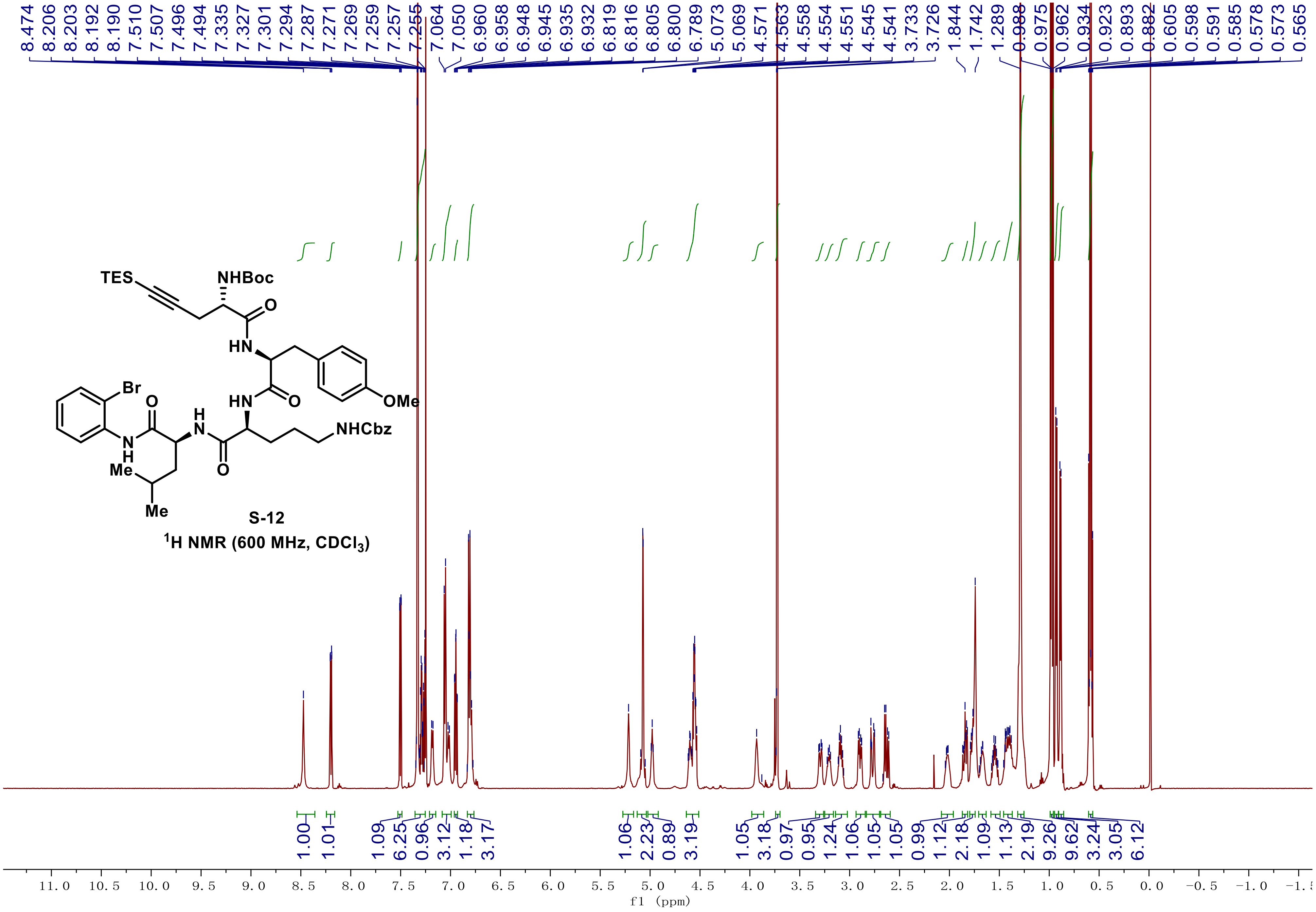

S-12

^{1}H NMR (600 MHz, CDCl$_3$)

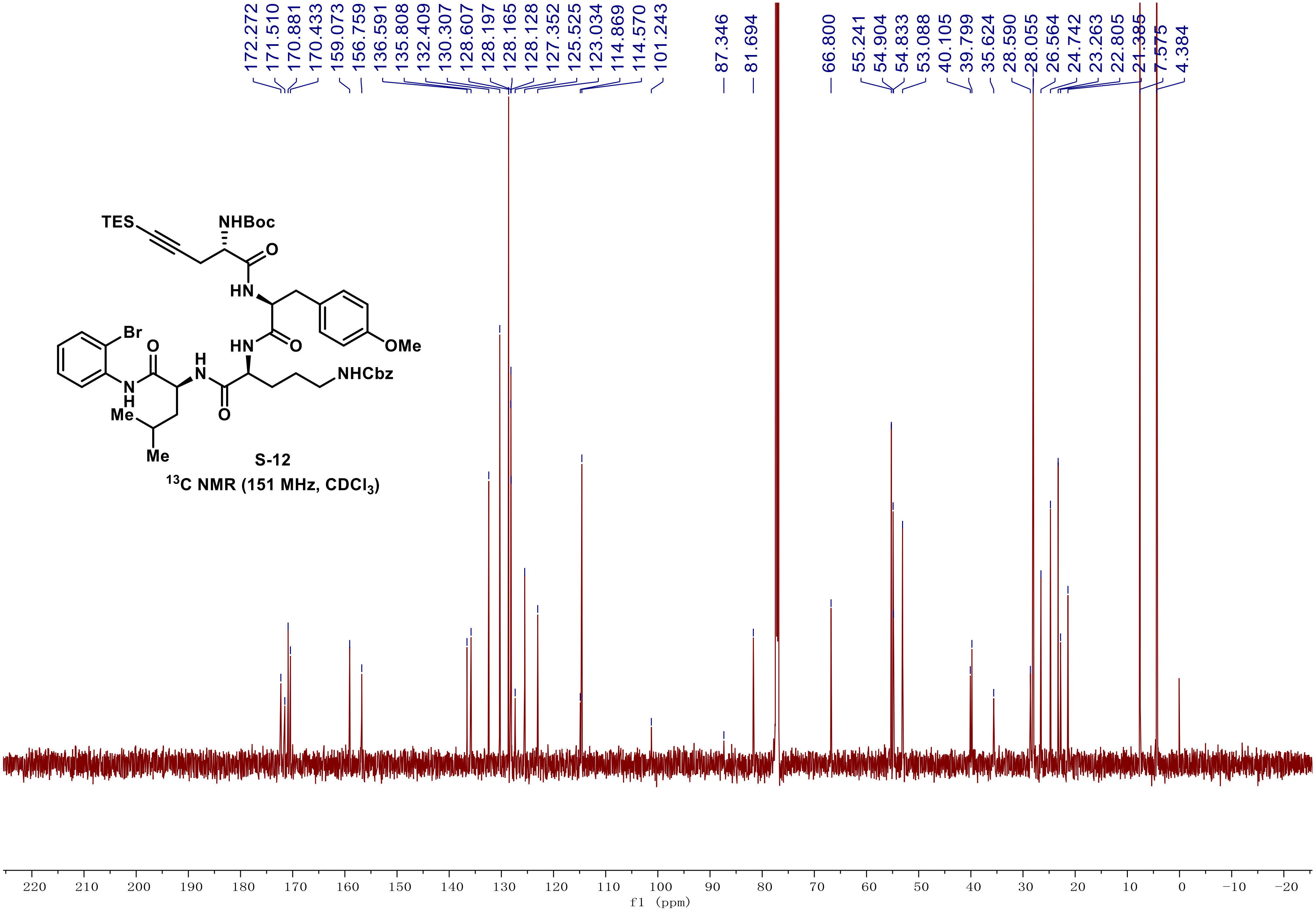
172.272
171.510
170.881
170.433
159.073
156.759
136.591
135.808
132.409
130.307
128.607
128.197
128.165
128.128
127.352
125.525
123.034
114.869
114.570
101.243
87.346
81.694
66.800
55.241
54.904
54.833
53.088
40.105
39.799
35.624
28.590
28.055
26.564
24.742
23.263
22.805
21.385
7.575
4.384
TES
NHBoc
O
HN
Br
O
HN
O
OMe
H
N
NHCbz
N
H
Me
O
Me
S-12
^{13}C NMR (151 MHz, CDCl$_3$)
f1 (ppm)

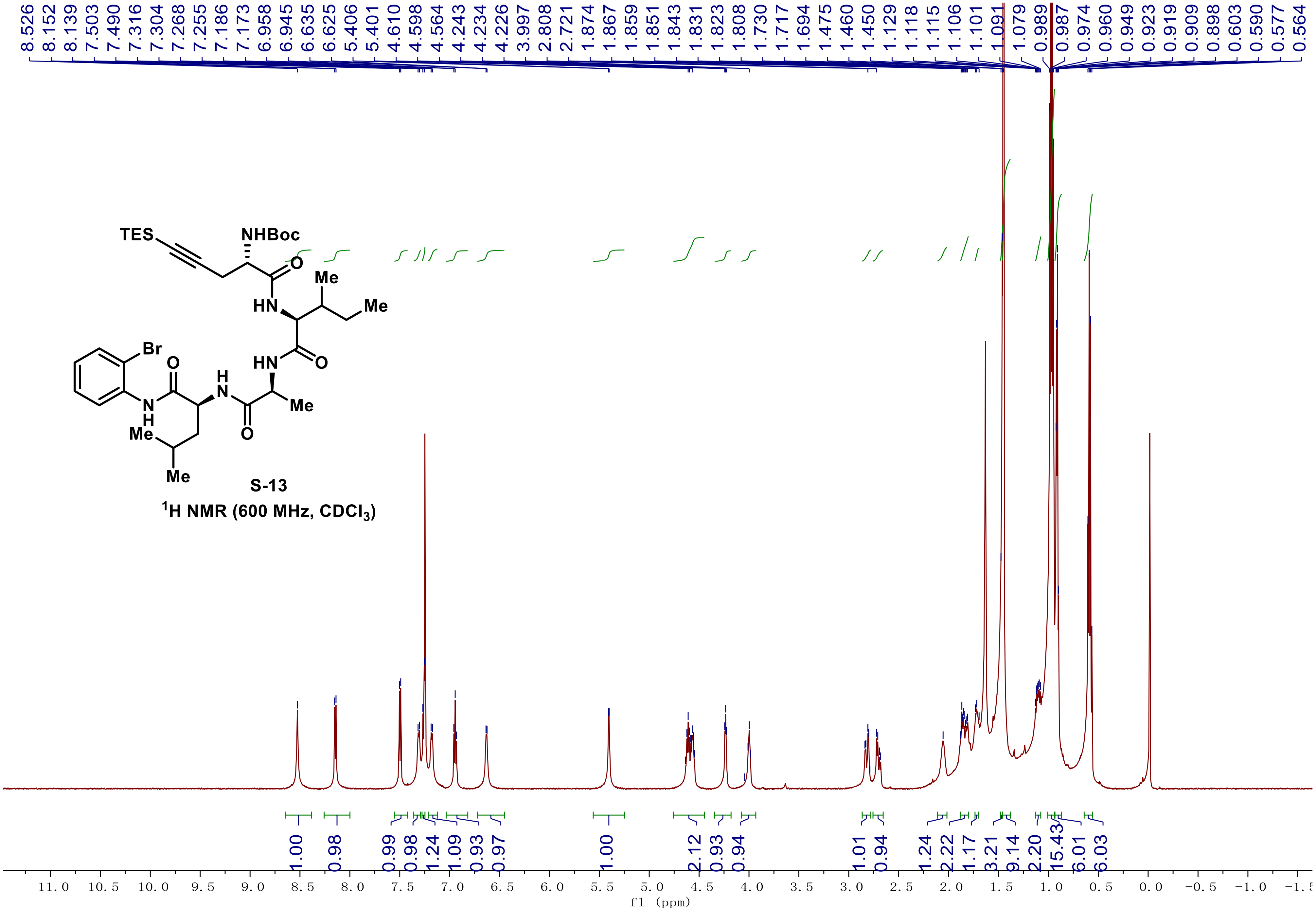
8.526 8.152 8.139 7.503 7.490 7.316 7.304 7.268 7.255 7.186 7.173 6.958 6.945 6.635 6.625 5.406 5.401 4.610 4.598 4.564 4.243 4.234 4.226 3.997 2.808 2.721 1.874 1.867 1.859 1.851 1.843 1.831 1.823 1.808 1.730 1.717 1.694 1.475 1.460 1.450 1.129 1.118 1.115 1.106 1.101 1.091 1.079 0.989 0.987 0.974 0.960 0.949 0.923 0.919 0.909 0.898 0.603 0.590 0.577 0.564
TES
NHBoc
O
Me
HN
Me
Br
O
HN
O
H
N
N
H
Me
O
Me
Me
S-13
1H NMR (600 MHz, CDCl3)
1.00 0.98 0.99 0.98 1.24 1.09 0.93 0.97 1.00 2.12 0.93 0.94 1.01 0.94 1.24 2.22 1.17 3.21 9.14 2.20 15.43 6.01 6.03
11.0 10.5 10.0 9.5 9.0 8.5 8.0 7.5 7.0 6.5 6.0 5.5 5.0 4.5 4.0 3.5 3.0 2.5 2.0 1.5 1.0 0.5 0.0 -0.5 -1.0
f1 (ppm)

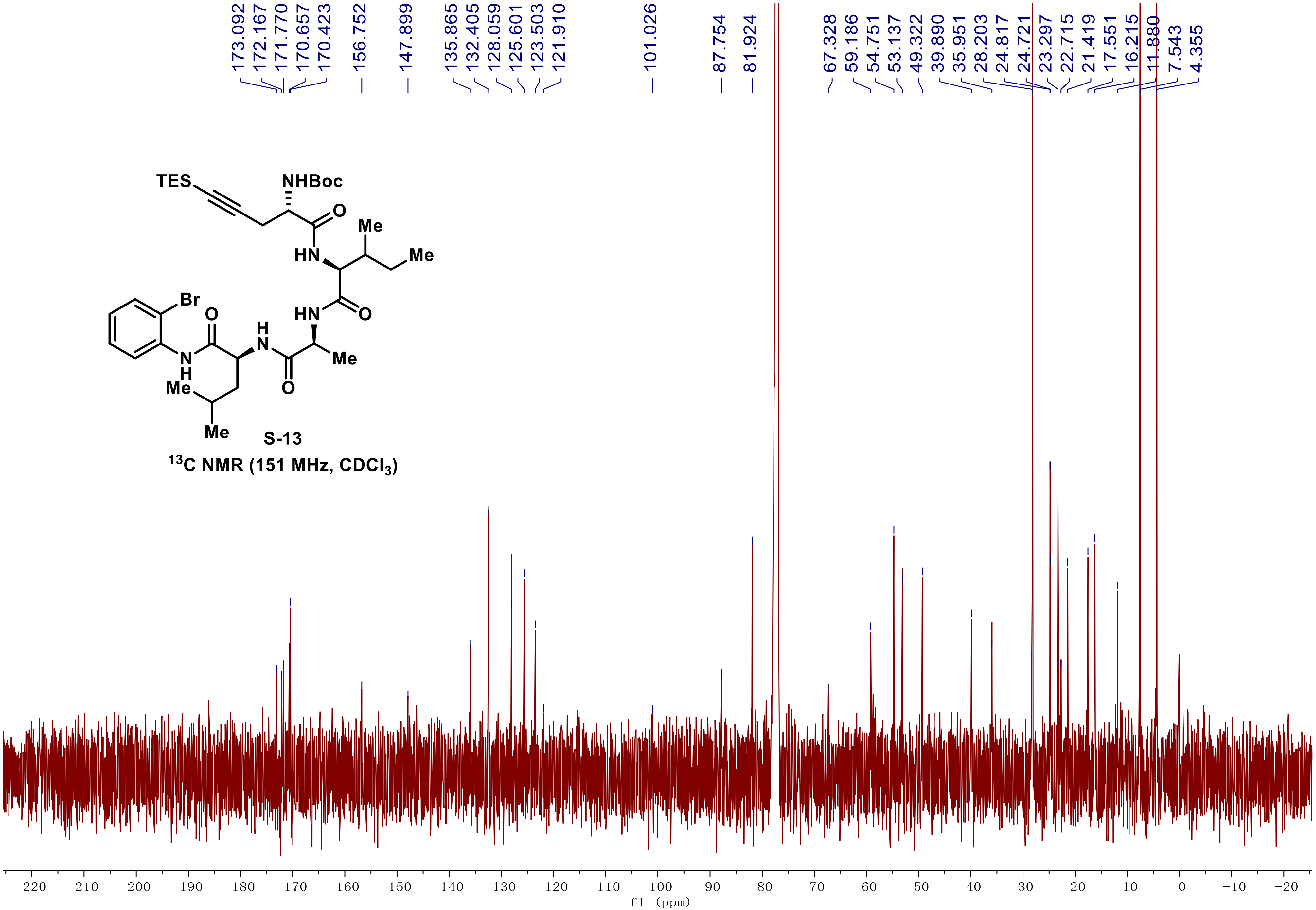
173.092
172.167
171.770
170.657
170.423
156.752
147.899
135.865
132.405
128.059
125.601
123.503
121.910
101.026
87.754
81.924
67.328
59.186
54.751
53.137
49.322
39.890
35.951
28.203
24.817
24.721
23.297
22.715
21.419
17.551
16.215
11.880
7.543
4.355
TES
NHBoc
O
Me
HN
Me
Br
O
HN
O
H
N
N
H
Me
Me
O
Me
S-13
^{13}C NMR (151 MHz, CDCl$_3$)
220 210 200 190 180 170 160 150 140 130 120 110 100 90 80 70 60 50 40 30 20 10 0 −10 −20
f1 (ppm)

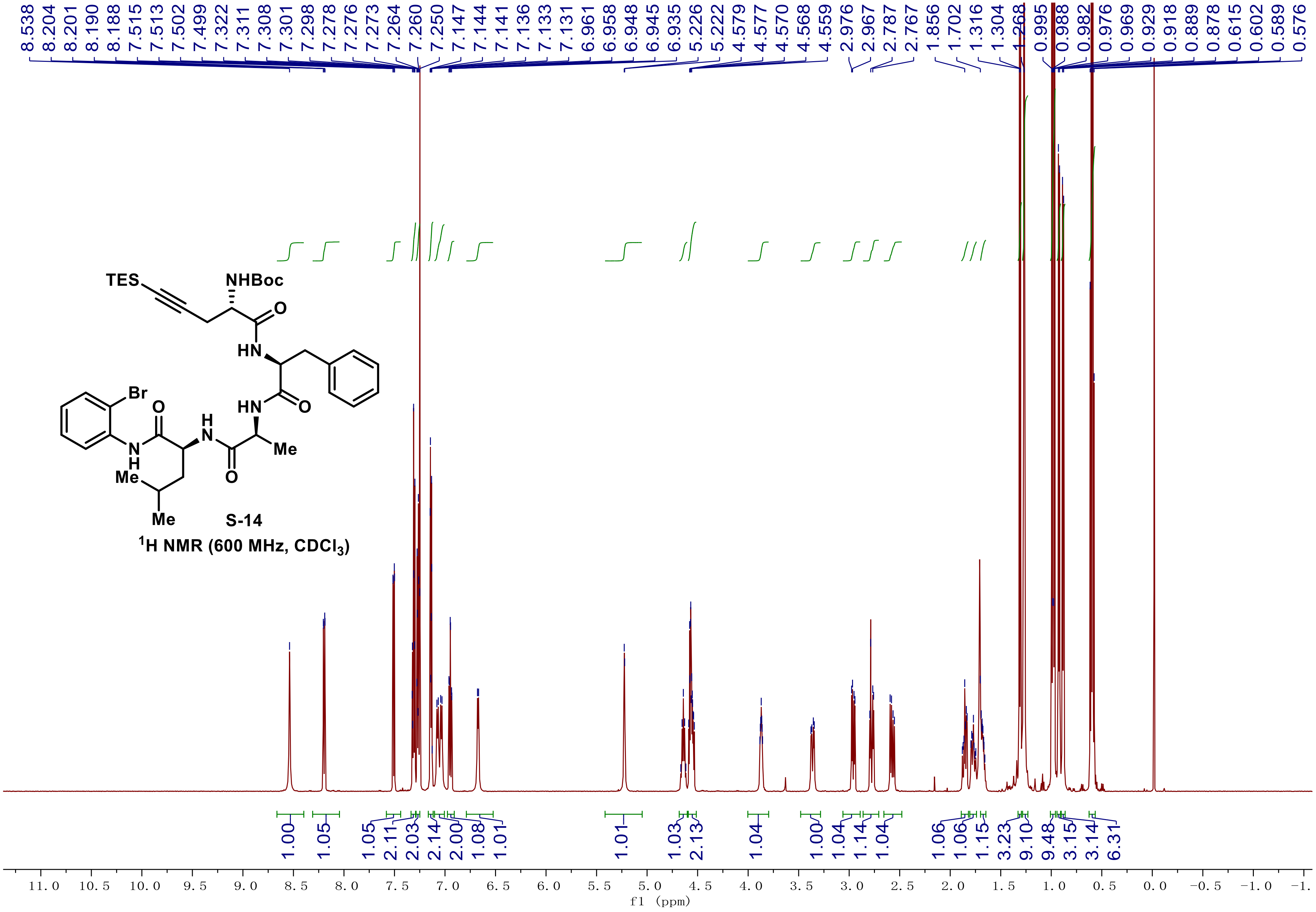
TES
NHBoc
O
HN
Br
O
H
N
HN
O
N
H
Me
Me
O
Me
S-14
1H NMR (600 MHz, CDCl3)
8.538 8.204 8.201 8.190 8.188 7.515 7.513 7.502 7.499 7.322 7.311 7.308 7.301 7.298 7.278 7.276 7.273 7.264 7.260 7.250 7.147 7.144 7.141 7.136 7.133 7.131 6.961 6.958 6.948 6.945 6.935 5.226 5.222 4.579 4.577 4.570 4.568 4.559 2.976 2.967 2.787 2.767 1.856 1.702 1.316 1.304 0.995 0.988 0.982 0.976 0.969 0.929 0.918 0.889 0.878 0.615 0.602 0.589 0.576
1.00 1.05 1.05 2.11 2.03 2.14 2.00 1.08 1.01 1.01 1.03 2.13 1.04 1.00 1.04 1.14 1.04 1.06 1.06 1.15 3.23 9.10 9.48 3.15 3.14 6.31
11.0 10.5 10.0 9.5 9.0 8.5 8.0 7.5 7.0 6.5 6.0 5.5 5.0 4.5 4.0 3.5 3.0 2.5 2.0 1.5 1.0 0.5 0.0 -0.5 -1.0
f1 (ppm)

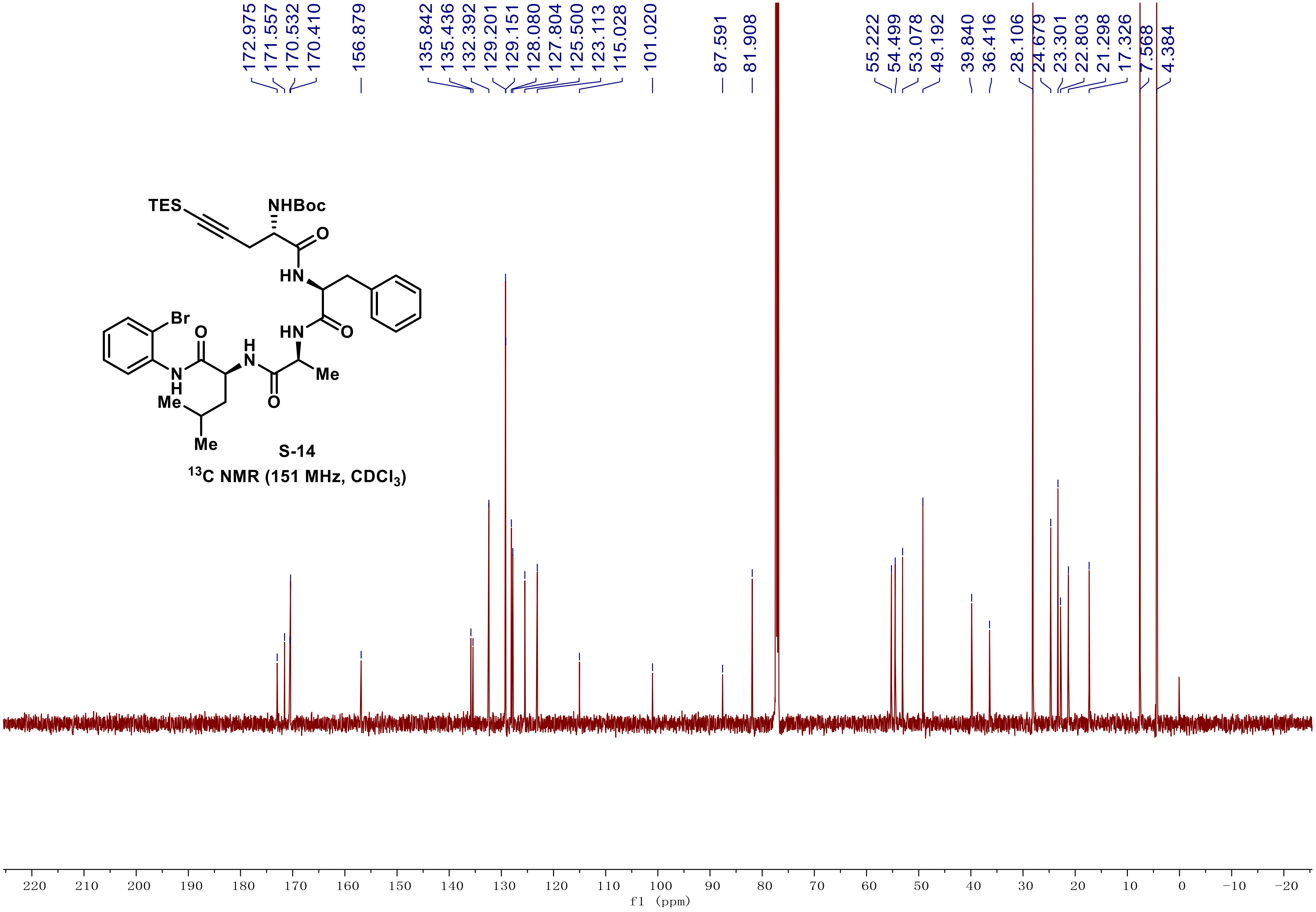

172.975
171.557
170.532
170.410
156.879
135.842
135.436
132.392
129.201
129.151
128.080
127.804
125.500
123.113
115.028
101.020
87.591
81.908
55.222
54.499
53.078
49.192
39.840
36.416
28.106
24.679
23.301
22.803
21.298
17.326
7.568
4.384
TES
NHBoc
O
HN
Br
O
HN
O
H
N
N
H
Me
Me
O
Me
S-14
13C NMR (151 MHz, CDCl3)
f1 (ppm)

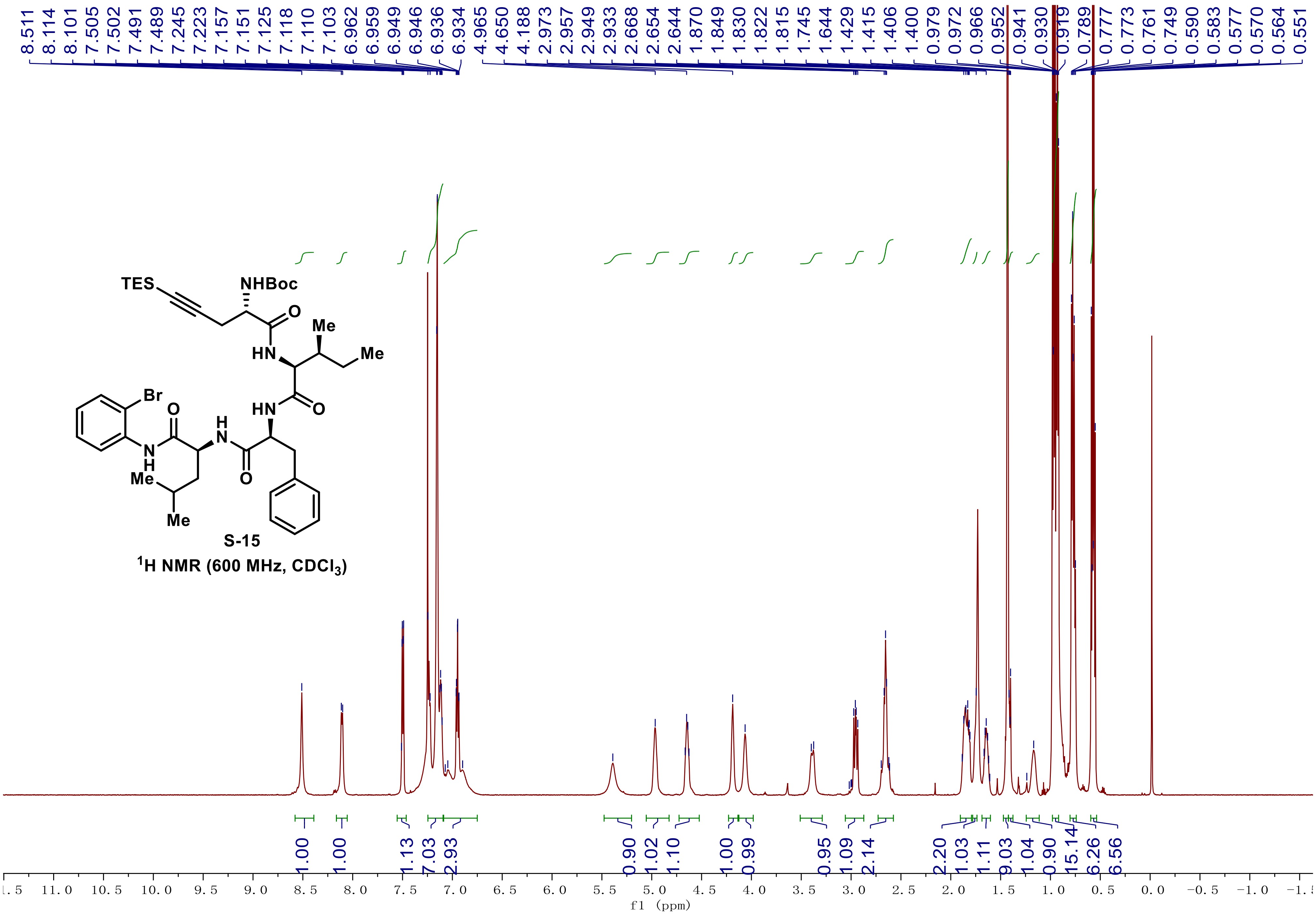

TES
NHBoc
O
Me
HN
Me
Br
O
HN
O
H
N
N
H
Me
O
Me
S-15
1H NMR (600 MHz, CDCl3)
f1 (ppm)

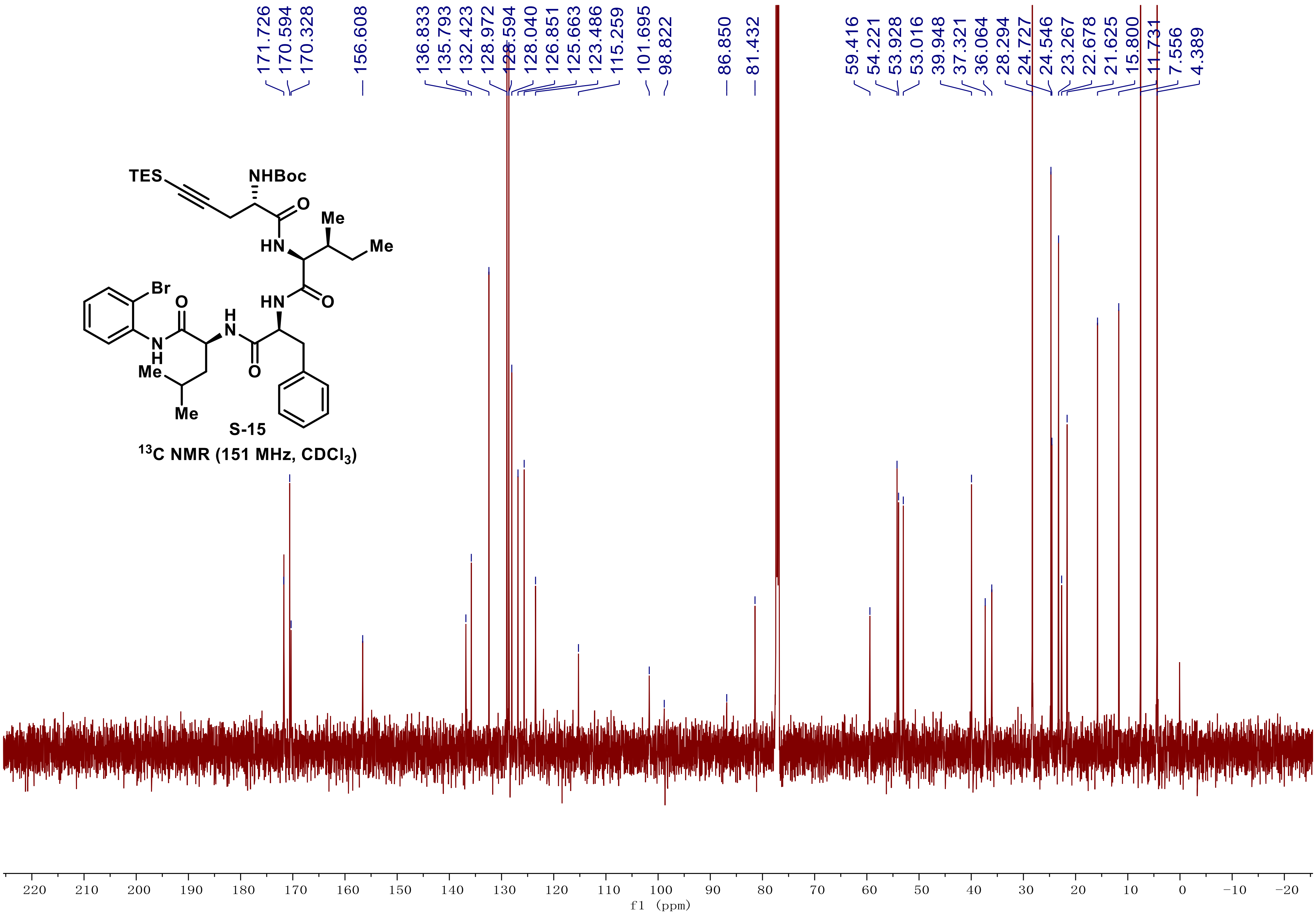
171.726
170.594
170.328
156.608
136.833
135.793
132.423
128.972
128.594
128.040
126.851
125.663
123.486
115.259
101.695
98.822
86.850
81.432
59.416
54.221
53.928
53.016
39.948
37.321
36.064
28.294
24.727
24.546
23.267
22.678
21.625
15.800
11.731
7.556
4.389
TES
NHBoc
O
Me
HN
Me
Br
O
HN
O
H
N
N
H
Me
O
Me
S-15
^{13}C NMR (151 MHz, CDCl$_3$)
220
210
200
190
180
170
160
150
140
130
120
110
100
90
80
70
60
50
40
30
20
10
0
−10
−20
f1 (ppm)

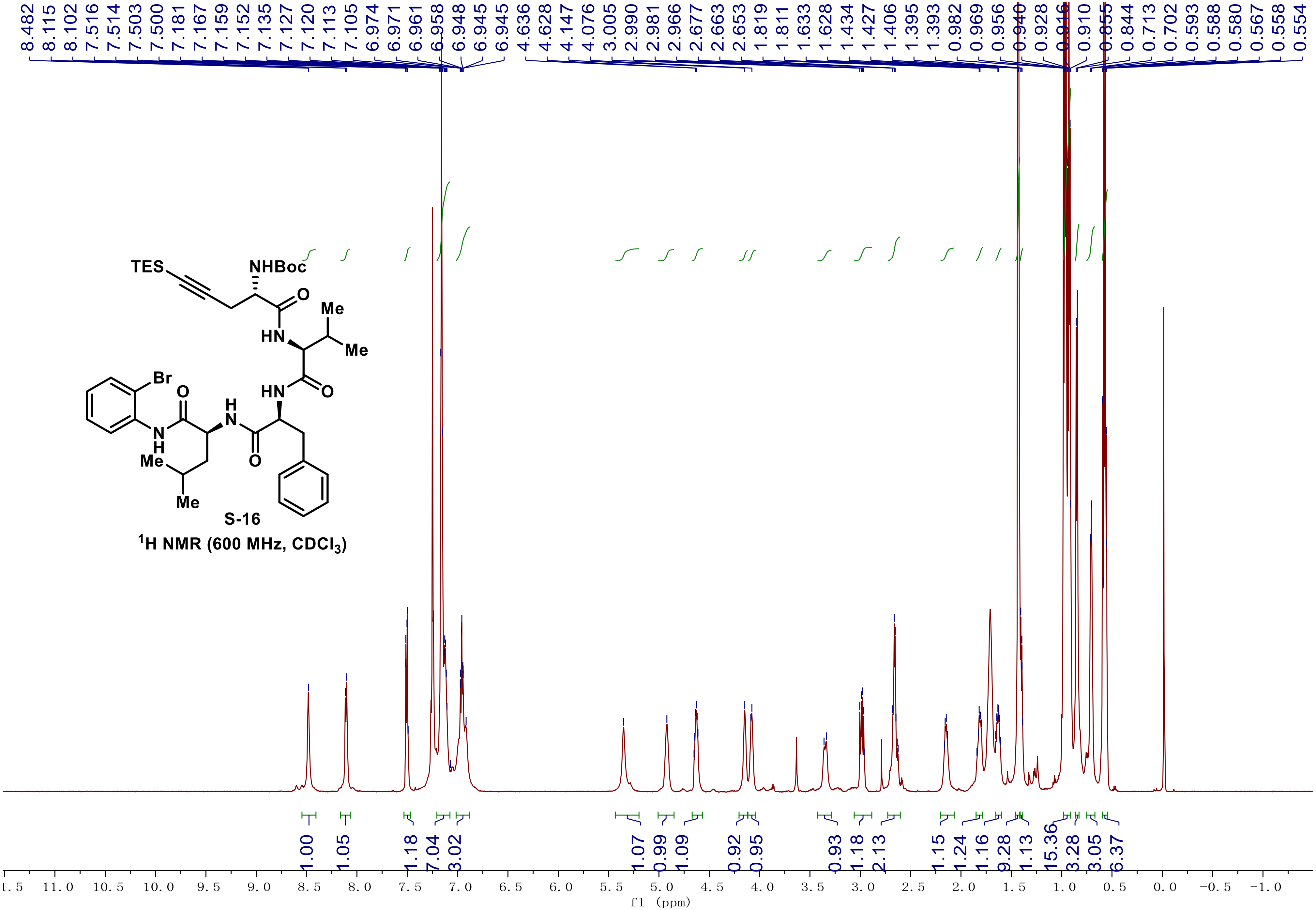
8.482 8.115 8.102 7.516 7.514 7.503 7.500 7.181 7.167 7.159 7.152 7.135 7.127 7.120 7.113 7.105 6.974 6.971 6.961 6.958 6.948 6.945 6.945 4.636 4.628 4.147 4.076 3.005 2.990 2.981 2.966 2.677 2.663 2.653 1.819 1.811 1.633 1.628 1.434 1.427 1.406 1.395 1.393 0.982 0.969 0.956 0.940 0.928 0.916 0.910 0.855 0.844 0.713 0.702 0.593 0.588 0.580 0.567 0.558 0.554
TES
NHBoc
Me
Me
HN
HN
Br
H
N
N
H
Me
Me
S-16
^{1}H NMR (600 MHz, CDCl$_3$)
1.00 1.05 1.18 7.04 3.02 1.07 0.99 1.09 0.92 0.95 0.93 1.18 2.13 1.15 1.24 1.16 9.28 1.13 15.36 3.28 3.05 6.37
f1 (ppm)

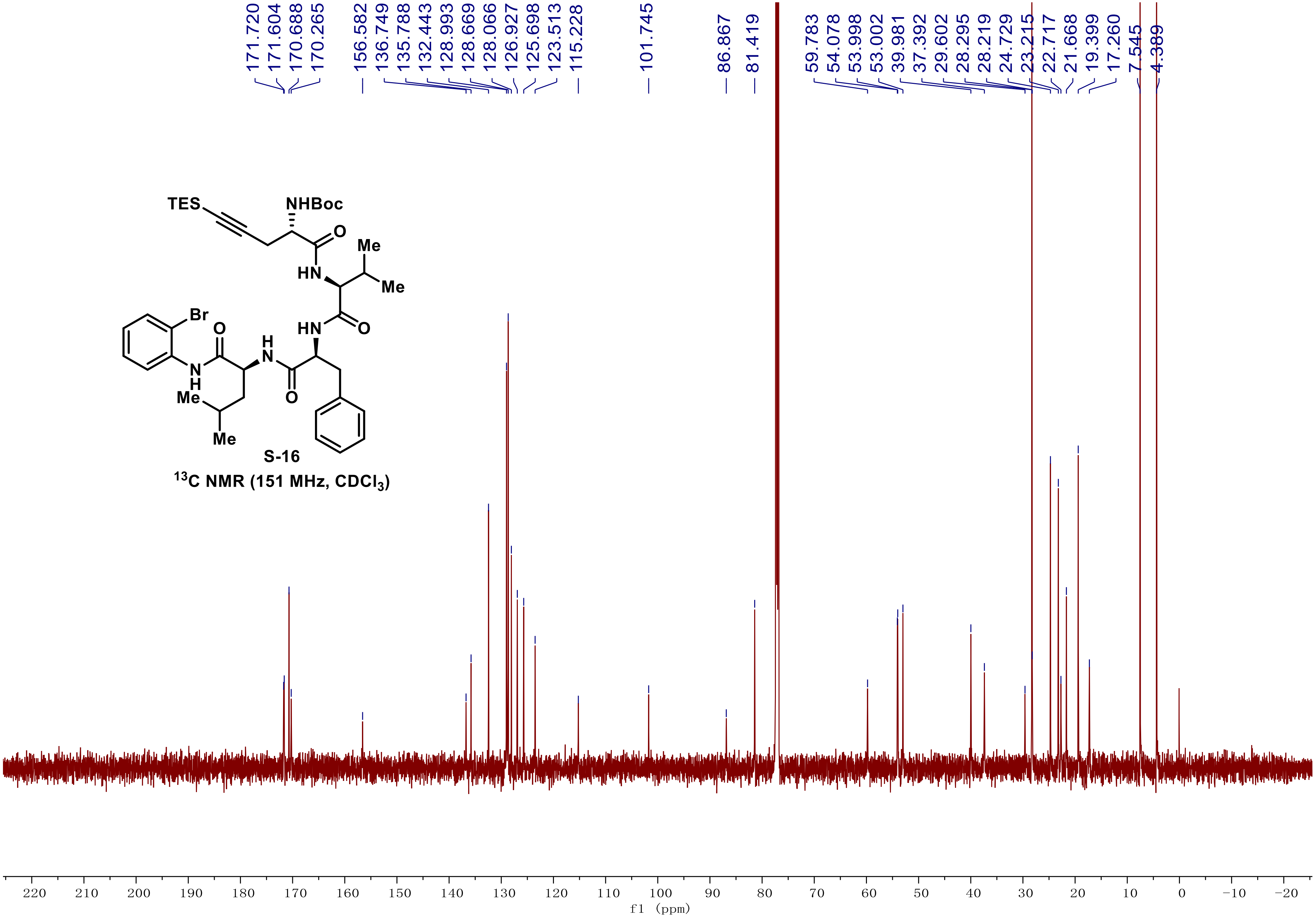
171.720
171.604
170.688
170.265
156.582
136.749
135.788
132.443
128.993
128.669
128.066
126.927
125.698
123.513
115.228
101.745
86.867
81.419
59.783
54.078
53.998
53.002
39.981
37.392
29.602
28.295
28.219
24.729
23.215
22.717
21.668
19.399
17.260
7.545
4.389
TES
NHBoc
O
Me
HN
Me
Br
O
H
N
HN
O
N
H
Me
O
Me
S-16
¹³C NMR (151 MHz, CDCl₃)
f1 (ppm)

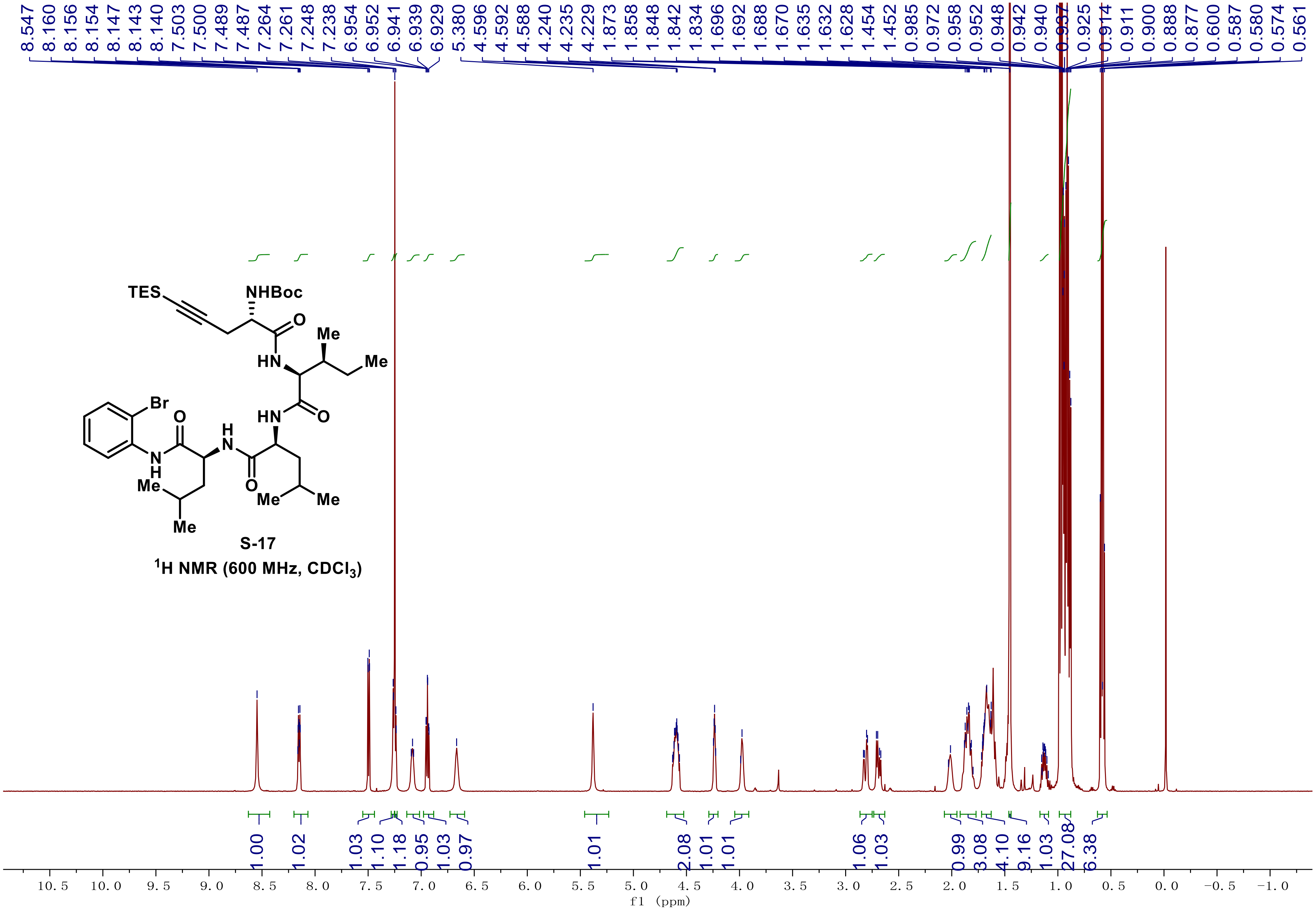

S-17

^{1}H NMR (600 MHz, $CDCl_3$)

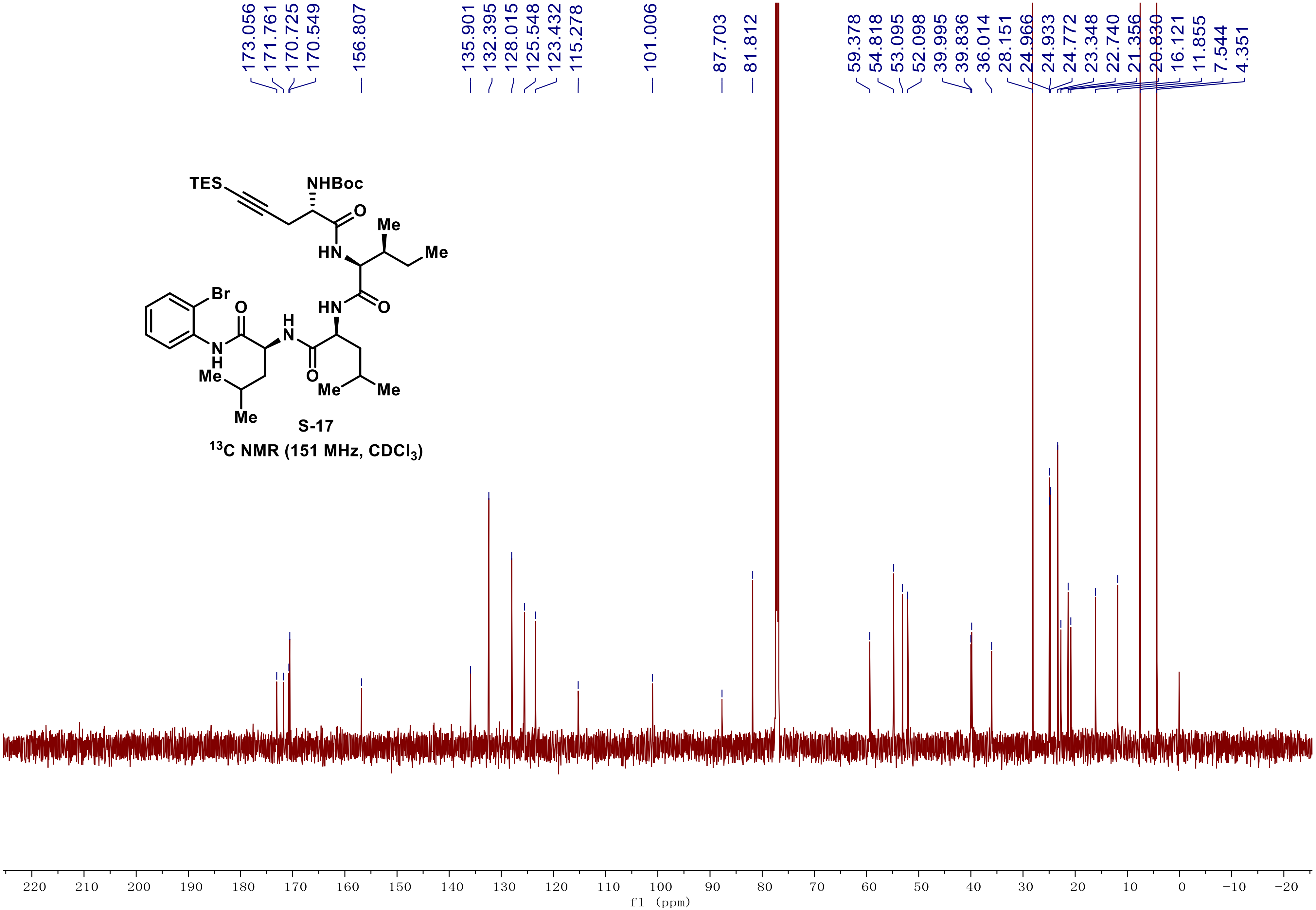
173.056
171.761
170.725
170.549
156.807
135.901
132.395
128.015
125.548
123.432
115.278
101.006
87.703
81.812
59.378
54.818
53.095
52.098
39.995
39.836
36.014
28.151
24.966
24.933
24.772
23.348
22.740
21.356
20.830
16.121
11.855
7.544
4.351
TES
NHBoc
O
Me
HN
Me
Br
O
H
N
HN
O
N
H
Me
O
Me
Me
Me
S-17
^{13}C NMR (151 MHz, CDCl$_3$)
f1 (ppm)

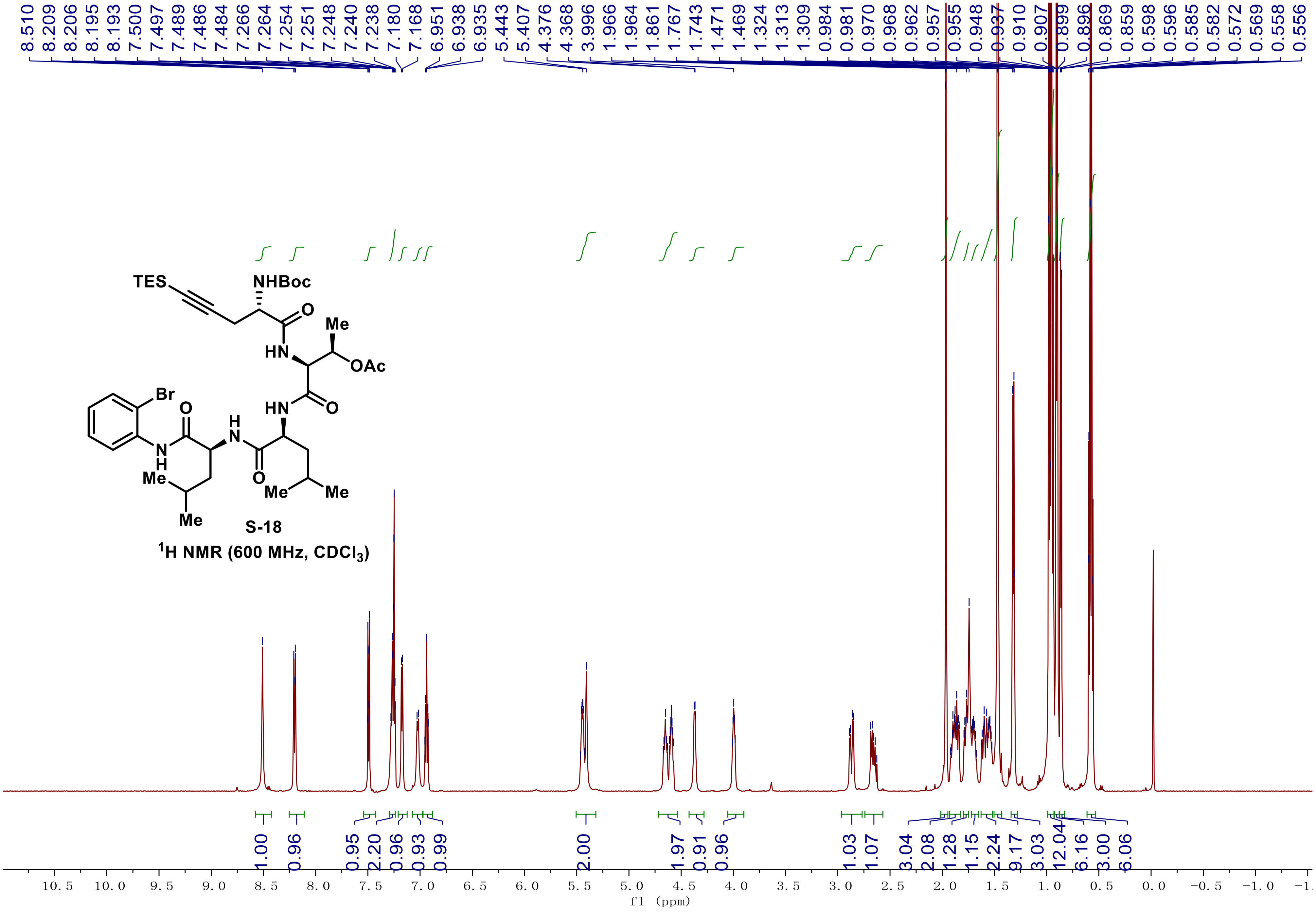

S-18

^{1}H NMR (600 MHz, $CDCl_3$)

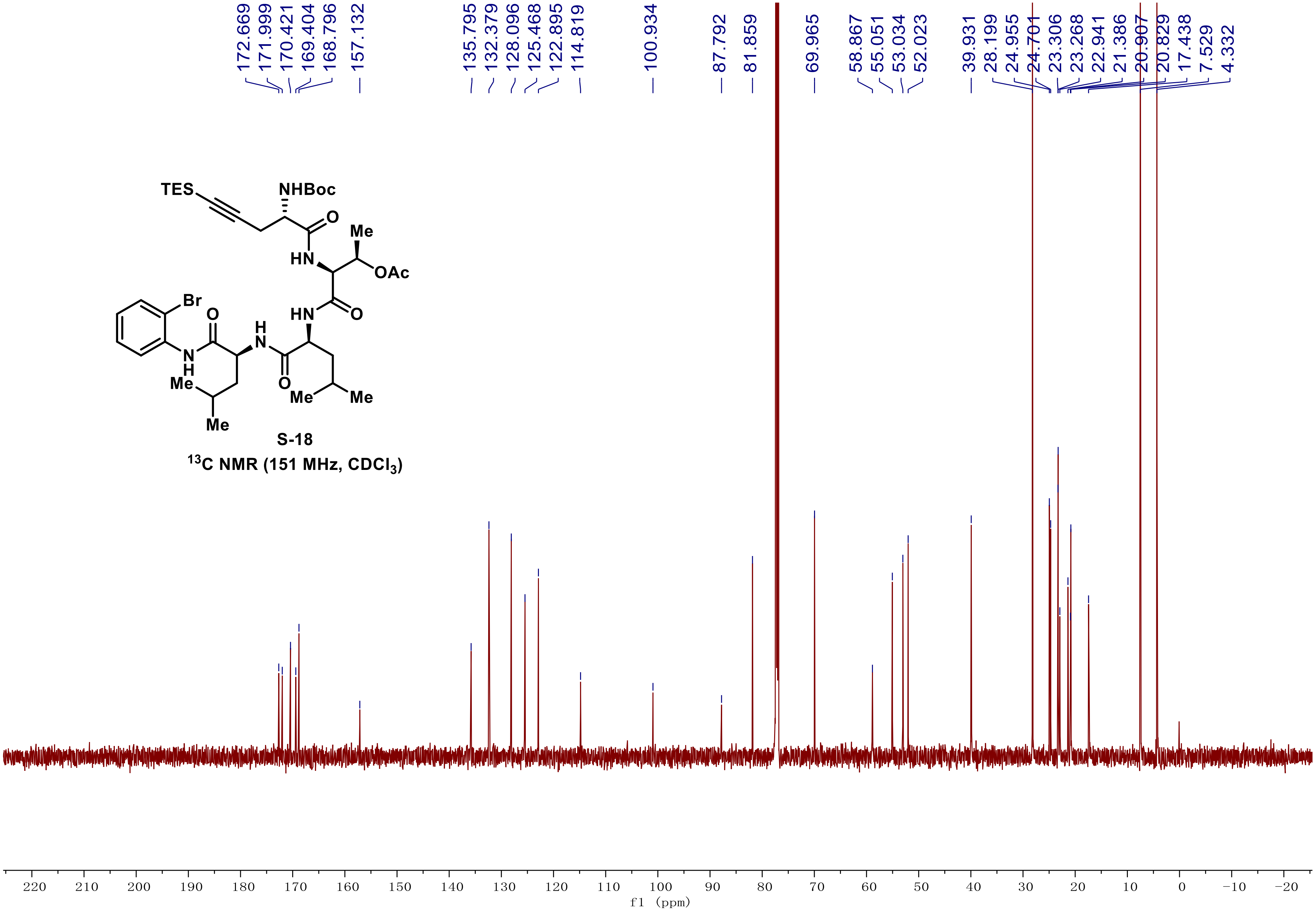
172.669
171.999
170.421
169.404
168.796
157.132
135.795
132.379
128.096
125.468
122.895
114.819
100.934
87.792
81.859
69.965
58.867
55.051
53.034
52.023
39.931
28.199
24.955
24.701
23.306
23.268
22.941
21.386
20.907
20.829
17.438
7.529
4.332
TES
NHBoc
O
Me
HN
OAc
Br
O
HN
O
H
N
N
H
O
Me
Me
Me
Me
S-18
13C NMR (151 MHz, CDCl3)
f1 (ppm)

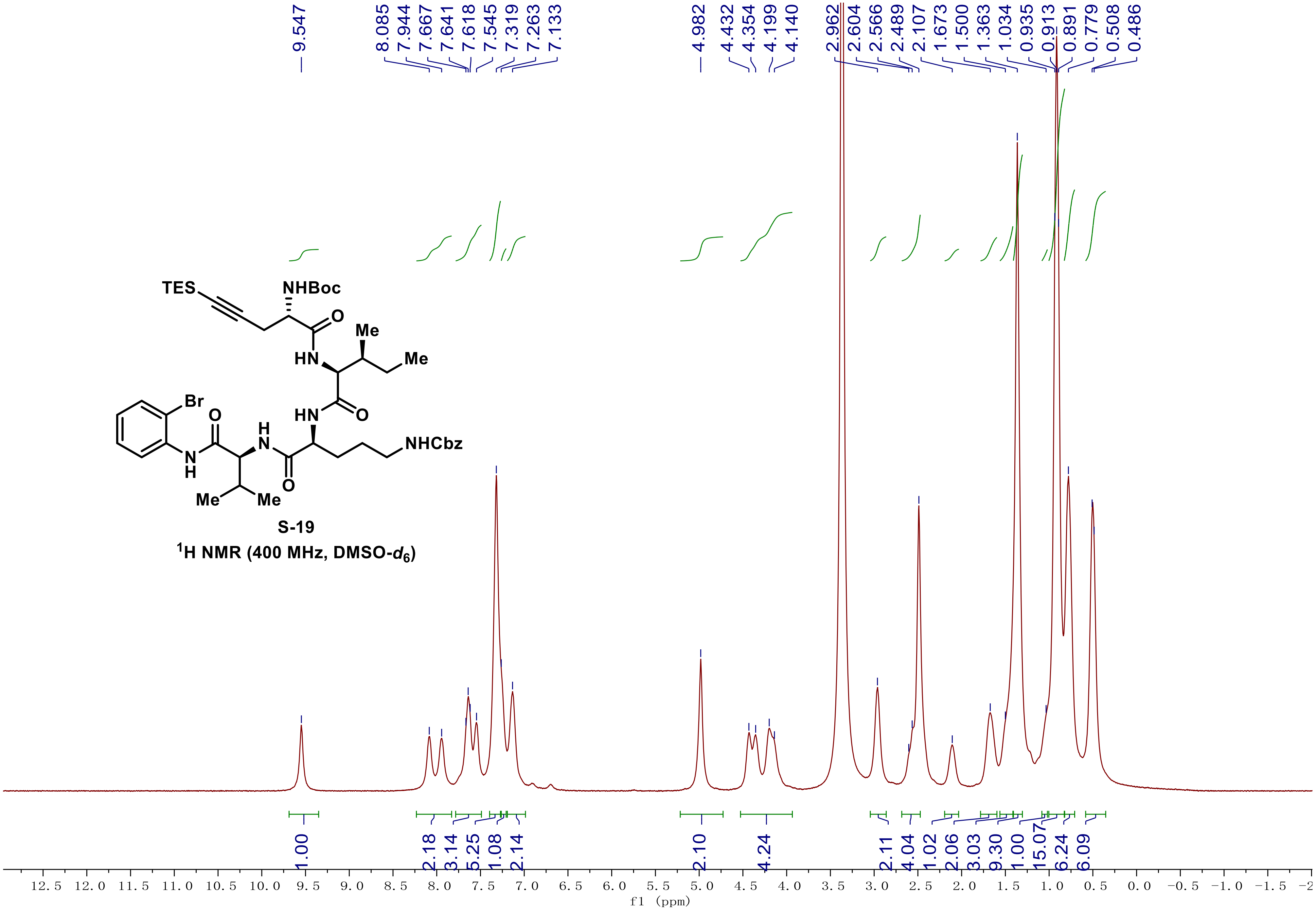
S-19
1H NMR (400 MHz, DMSO-d6)
f1 (ppm)

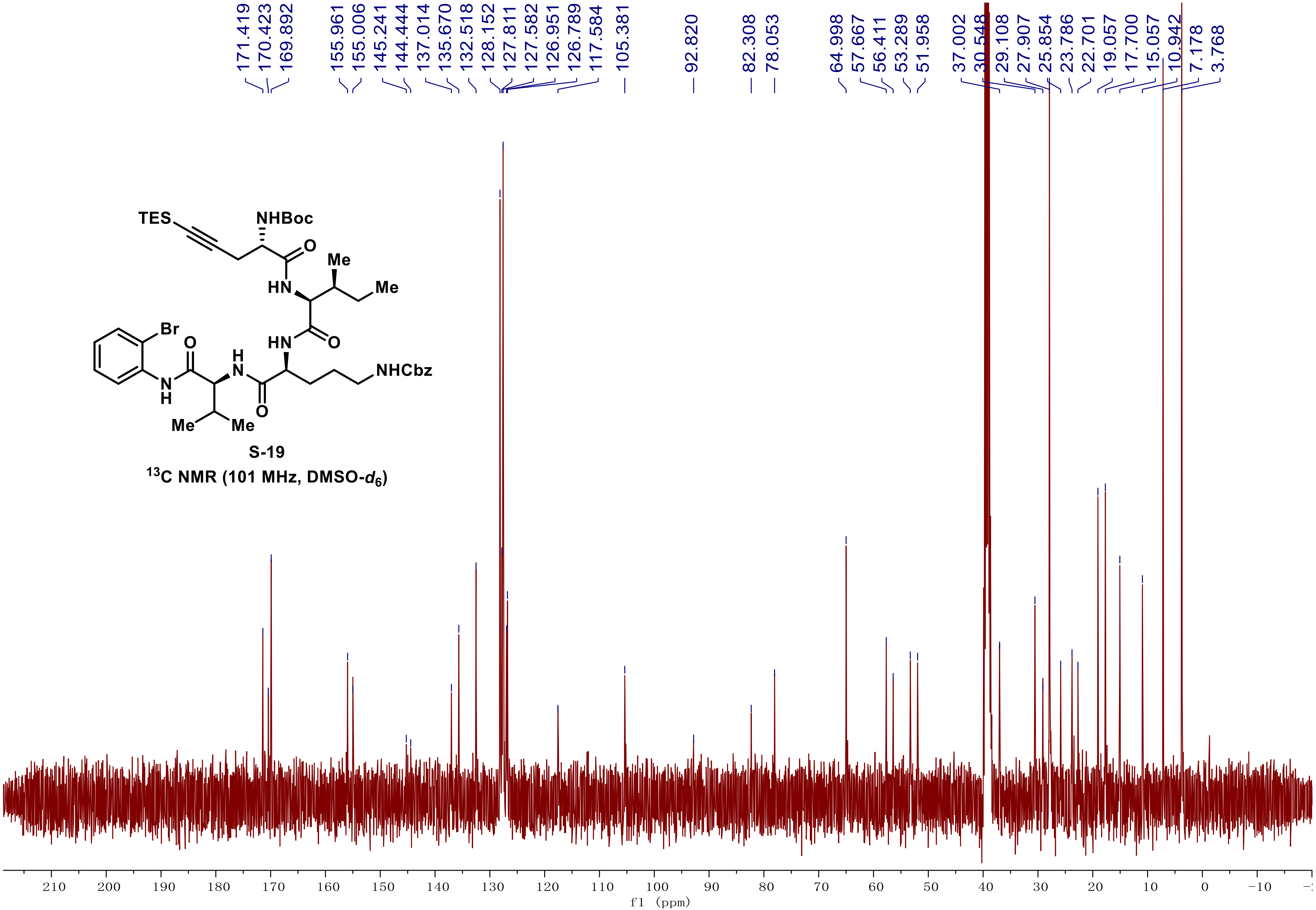

171.419
170.423
169.892
155.961
155.006
145.241
144.444
137.014
135.670
132.518
128.152
127.811
127.582
126.951
126.789
117.584
105.381
92.820
82.308
78.053
64.998
57.667
56.411
53.289
51.958
37.002
30.548
29.108
27.907
25.854
23.786
22.701
19.057
17.700
15.057
10.942
7.178
3.768
TES
NHBoc
O
Me
HN
Me
Br
O
H
N
HN
O
NHCbz
N
H
O
Me
Me
S-19
^{13}C NMR (101 MHz, DMSO-d_6)
f1 (ppm)

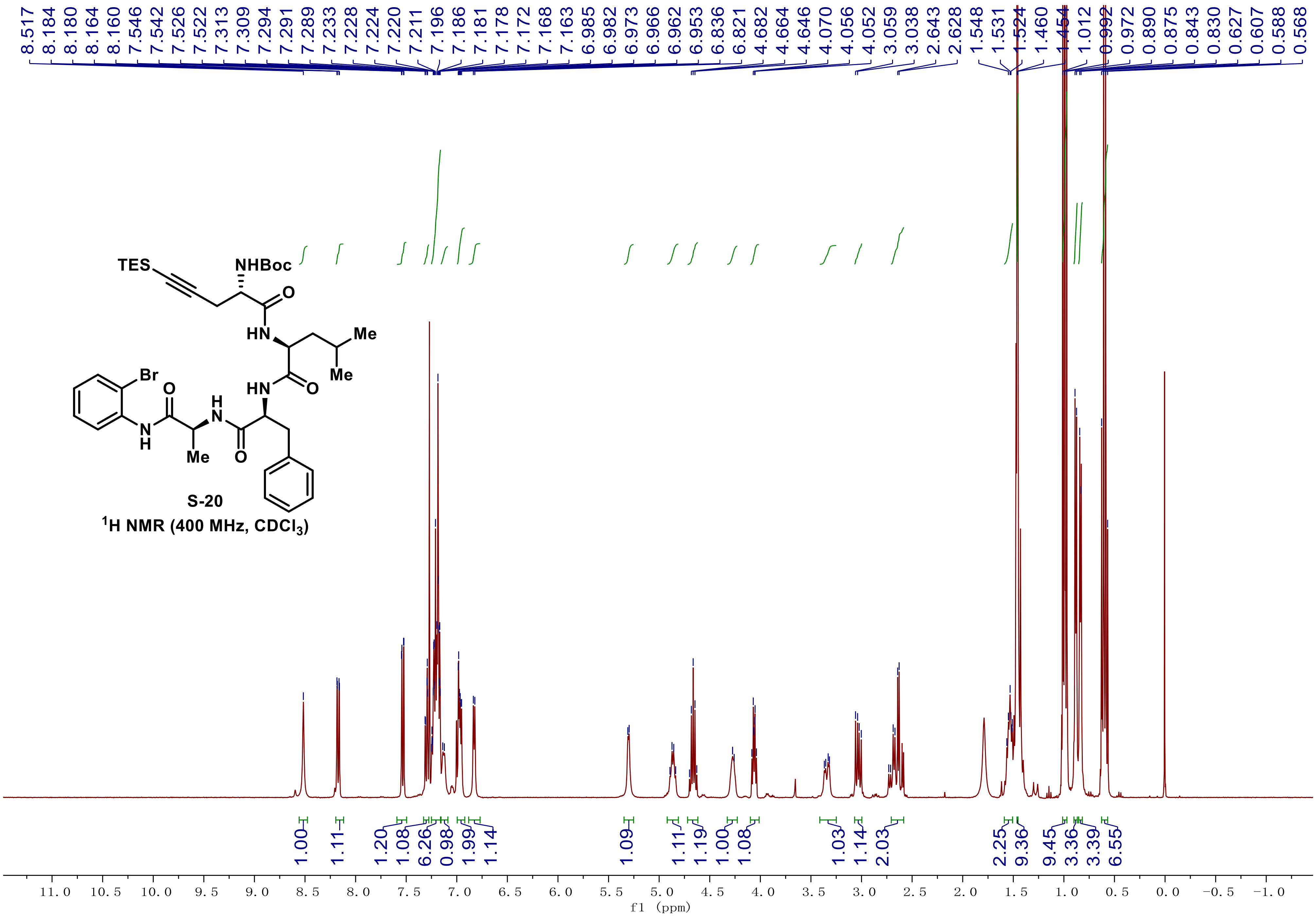
TES
NHBoc
O
HN
Me
Me
Br
O
H
N
HN
O
N
H
Me
O
S-20
1H NMR (400 MHz, CDCl3)
f1 (ppm)

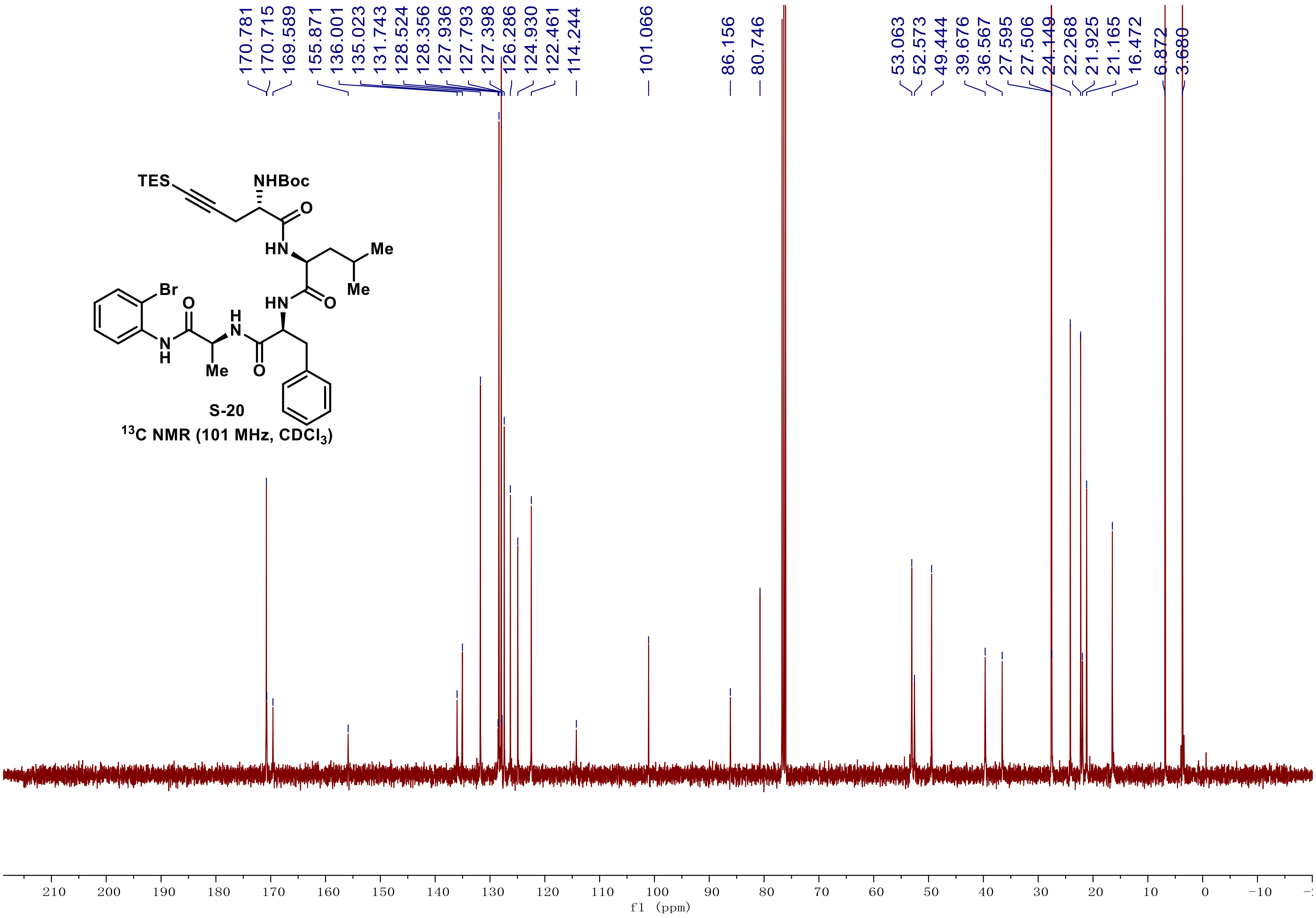
170.781
170.715
169.589
155.871
136.001
135.023
131.743
128.524
128.356
127.936
127.793
127.398
126.286
124.930
122.461
114.244
101.066
86.156
80.746
53.063
52.573
49.444
39.676
36.567
27.595
27.506
24.149
22.268
21.925
21.165
16.472
6.872
3.680
TES
NHBoc
O
HN
Me
Me
Br
O
H
N
HN
O
N
H
Me
O
S-20
13C NMR (101 MHz, CDCl3)
f1 (ppm)

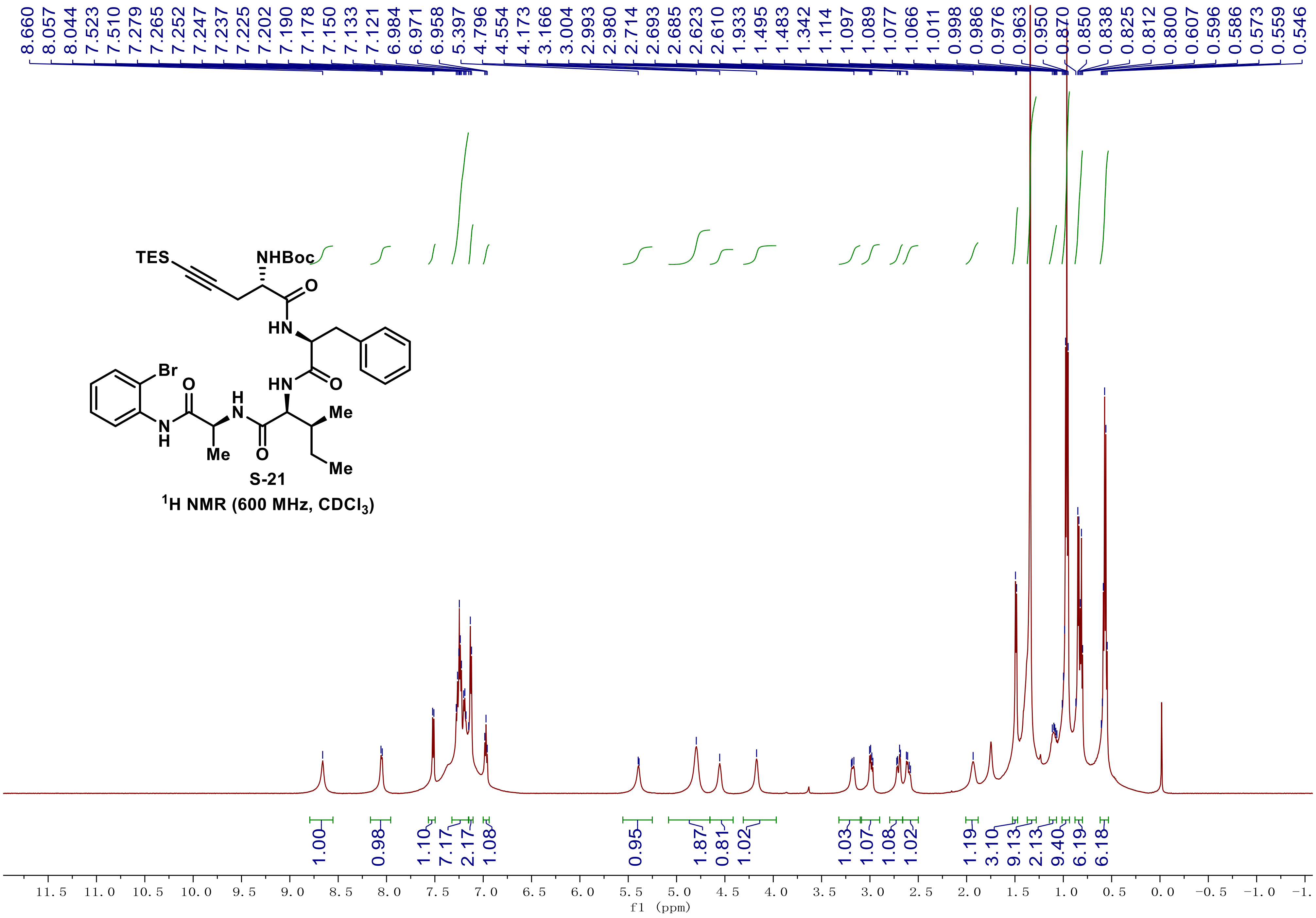
S-21
1H NMR (600 MHz, CDCl3)
TES
NHBoc
Br
f1 (ppm)

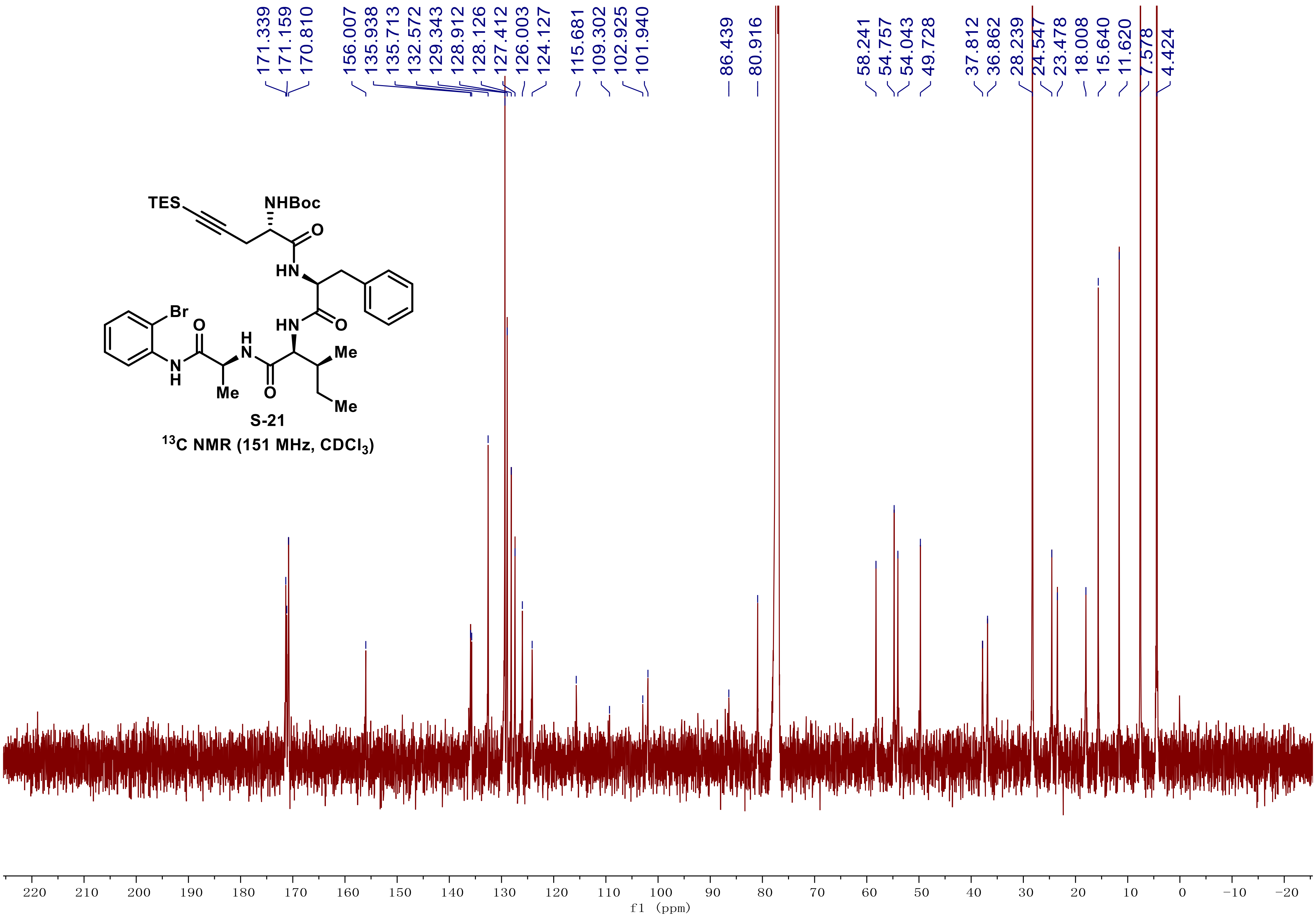

171.339
171.159
170.810
156.007
135.938
135.713
132.572
129.343
128.912
128.126
127.412
126.003
124.127
115.681
109.302
102.925
101.940
86.439
80.916
58.241
54.757
54.043
49.728
37.812
36.862
28.239
24.547
23.478
18.008
15.640
11.620
7.578
4.424
TES
NHBoc
O
HN
Br
O
HN
O
H
N
Me
N
H
Me
O
Me
S-21
13C NMR (151 MHz, CDCl3)
f1 (ppm)

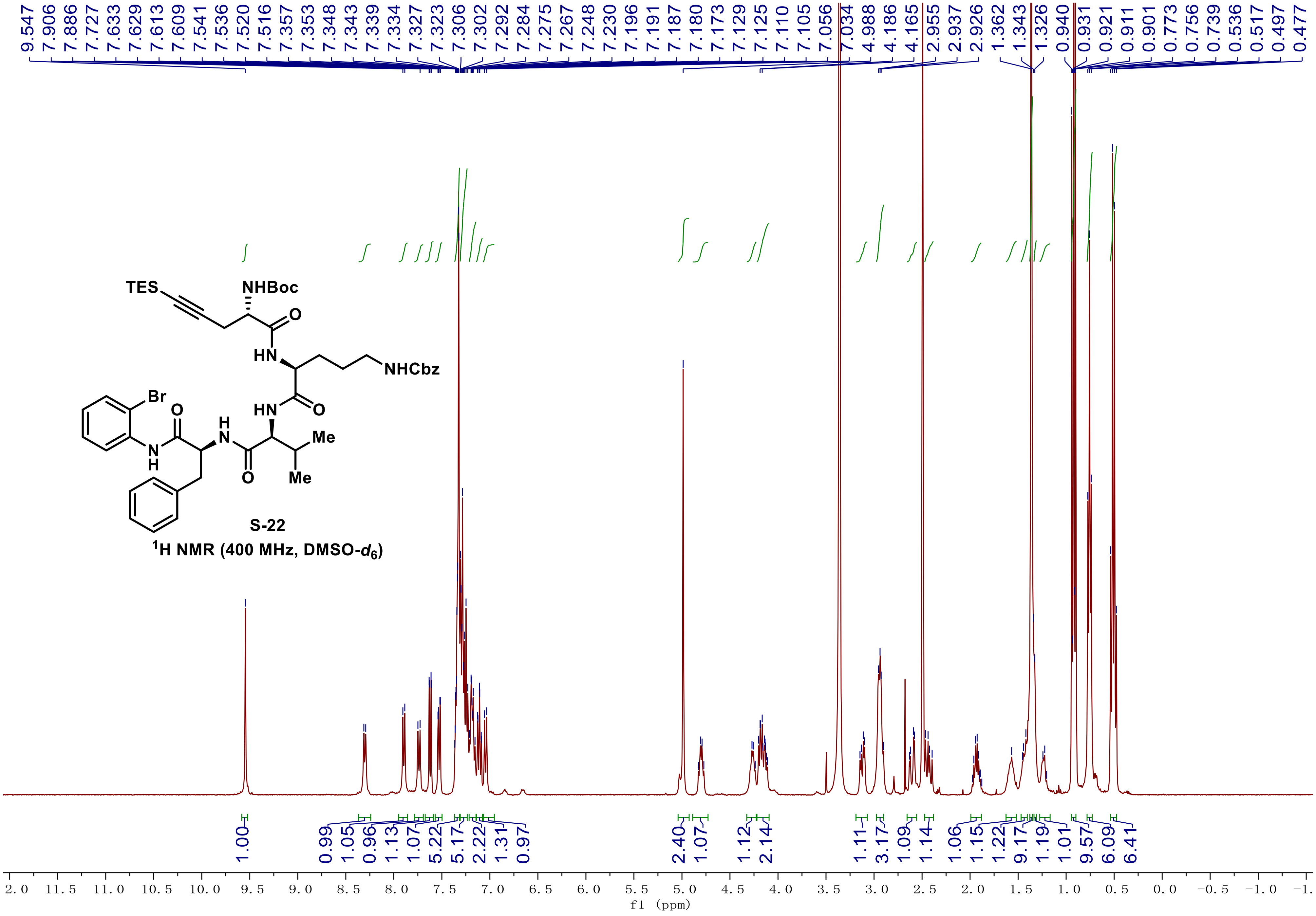
9.547 7.906 7.886 7.727 7.633 7.629 7.613 7.609 7.541 7.536 7.520 7.516 7.357 7.353 7.348 7.343 7.339 7.334 7.327 7.323 7.306 7.302 7.292 7.284 7.275 7.267 7.248 7.230 7.196 7.191 7.187 7.180 7.173 7.129 7.125 7.110 7.105 7.056 7.034 4.988 4.186 4.165 2.955 2.937 2.926 1.362 1.343 1.326 0.940 0.931 0.921 0.911 0.901 0.773 0.756 0.739 0.536 0.517 0.497 0.477
TES
NHBoc
O
HN
NHCbz
Br
O
H
N
HN
O
Me
N
H
O
Me
S-22
^{1}H NMR (400 MHz, DMSO-d_6)
1.00 0.99 1.05 0.96 1.13 1.07 5.22 5.17 2.22 1.31 0.97 2.40 1.07 1.12 2.14 1.11 3.17 1.09 1.14 1.06 1.15 1.22 9.17 1.19 1.01 9.57 6.09 6.41
f1 (ppm)

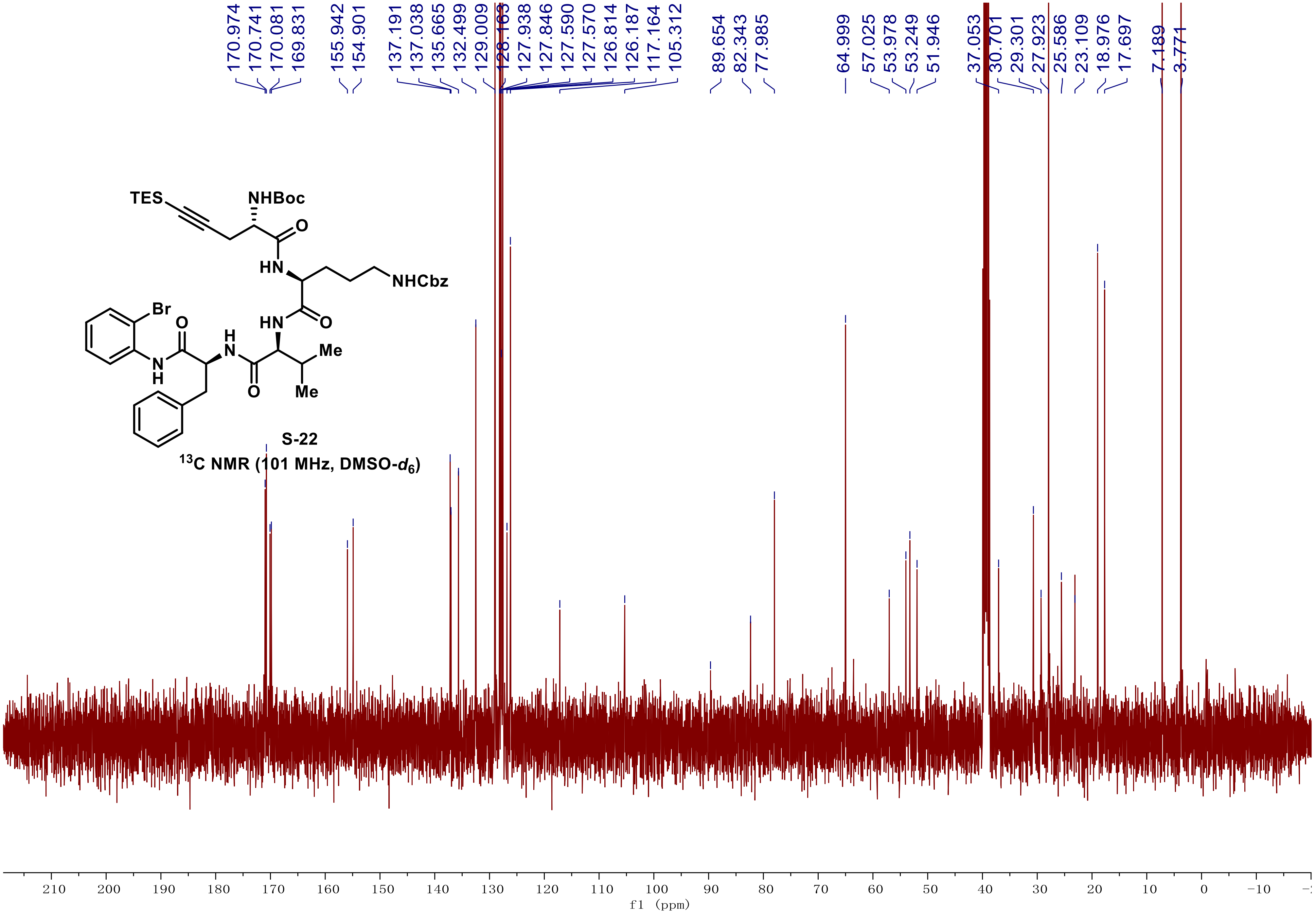

S-22

^{13}C NMR (101 MHz, DMSO-d_6)

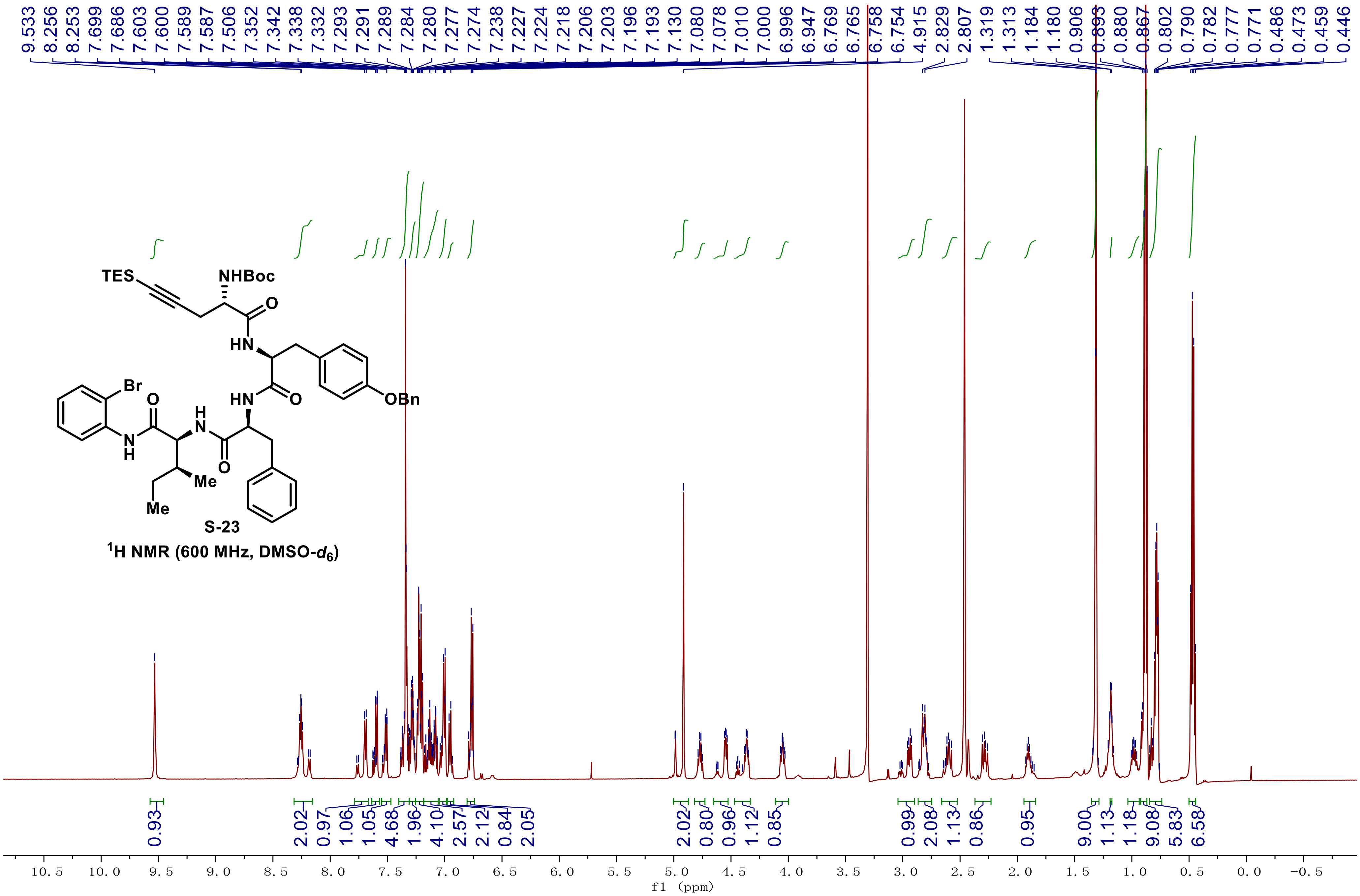
9.533 8.256 8.253 7.699 7.686 7.603 7.600 7.589 7.587 7.506 7.352 7.342 7.338 7.332 7.293 7.291 7.289 7.284 7.280 7.277 7.274 7.238 7.227 7.224 7.218 7.206 7.203 7.196 7.193 7.130 7.080 7.078 7.010 7.000 6.996 6.947 6.769 6.765 6.758 6.754 4.915 2.829 2.807 1.319 1.313 1.184 1.180 0.906 0.893 0.880 0.867 0.802 0.790 0.782 0.777 0.771 0.486 0.473 0.459 0.446
TES
NHBoc
O
HN
HN
O
OBn
Br
O
H
N
N
H
O
Me
Me
S-23
1H NMR (600 MHz, DMSO-d6)
0.93 2.02 0.97 1.06 1.05 4.68 1.96 4.10 2.57 2.12 0.84 2.05 2.02 0.80 0.96 1.12 0.85 0.99 2.08 1.13 0.86 0.95 9.00 1.13 1.18 9.08 5.83 6.58
10.5 10.0 9.5 9.0 8.5 8.0 7.5 7.0 6.5 6.0 5.5 5.0 4.5 4.0 3.5 3.0 2.5 2.0 1.5 1.0 0.5 0.0 -0.5
f1 (ppm)

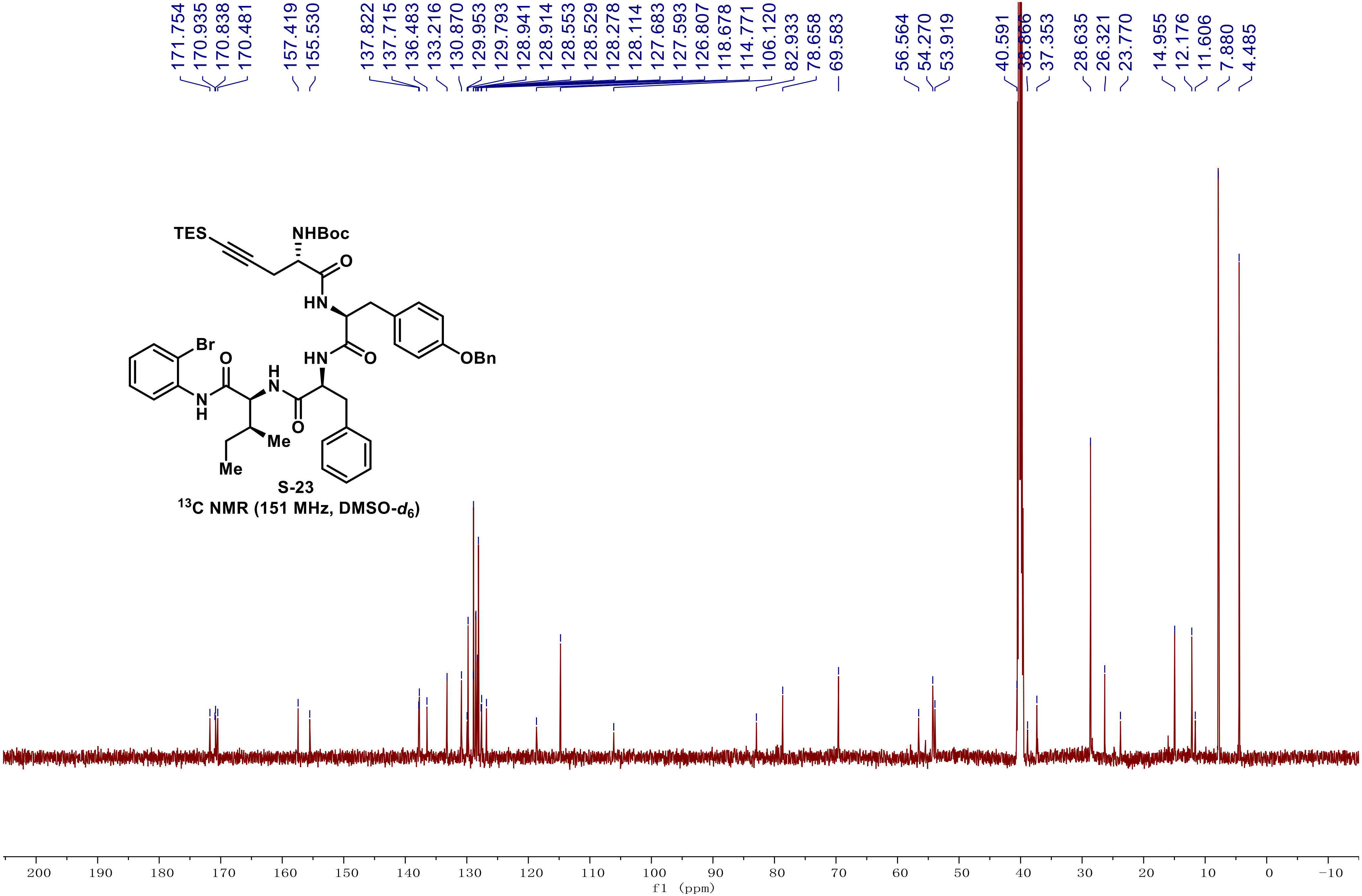

S-23

^{13}C NMR (151 MHz, DMSO-d_6)

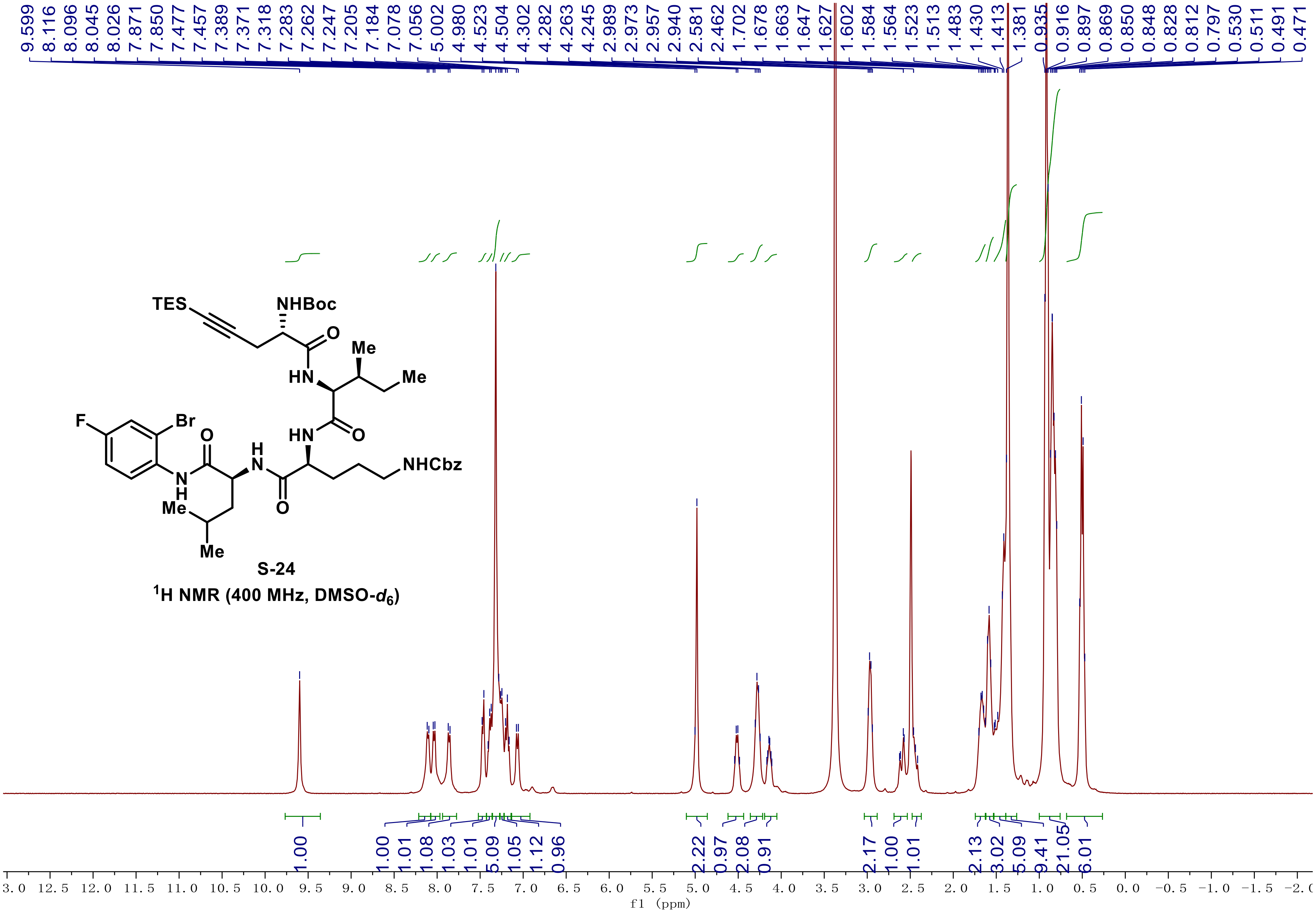

9.599 8.116 8.096 8.045 8.026 7.871 7.850 7.477 7.457 7.389 7.371 7.318 7.283 7.262 7.247 7.205 7.184 7.078 7.056 5.002 4.980 4.523 4.504 4.302 4.282 4.263 4.245 2.989 2.973 2.957 2.940 2.581 2.462 1.702 1.678 1.663 1.647 1.627 1.602 1.584 1.564 1.523 1.513 1.483 1.430 1.413 1.381 0.935 0.916 0.897 0.869 0.850 0.848 0.828 0.812 0.797 0.530 0.511 0.491 0.471
TES
NHBoc
O
Me
HN
Me
F
Br
HN
O
O
H
N
NHCbz
N
H
Me
O
Me
S-24
1H NMR (400 MHz, DMSO-d6)
1.00 1.00 1.01 1.08 1.03 1.01 5.09 1.05 1.12 0.96 2.22 0.97 2.08 0.91 2.17 1.00 1.01 2.13 3.02 5.09 9.41 21.05 6.01
f1 (ppm)

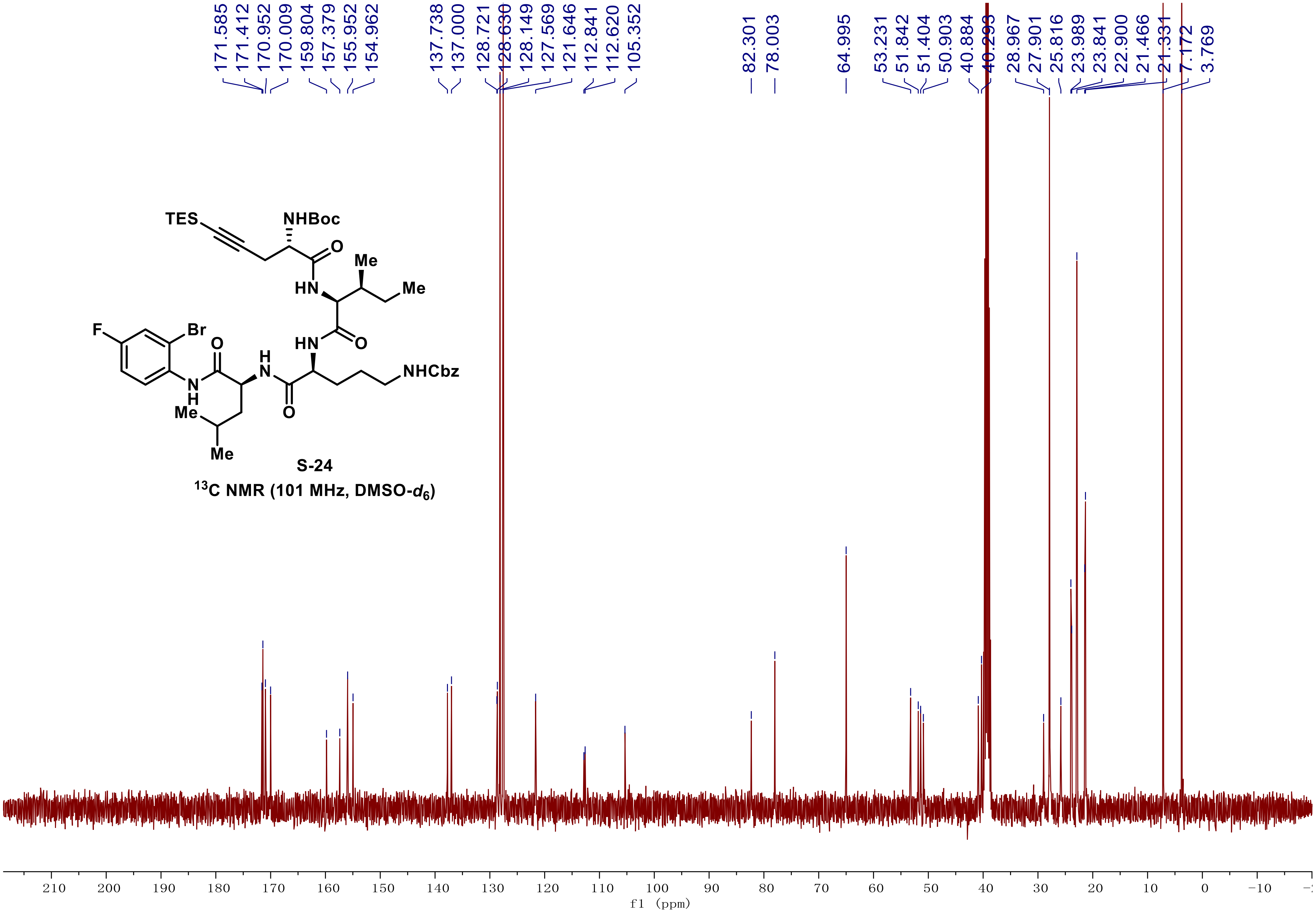

S-24

^{13}C NMR (101 MHz, DMSO-d_6)

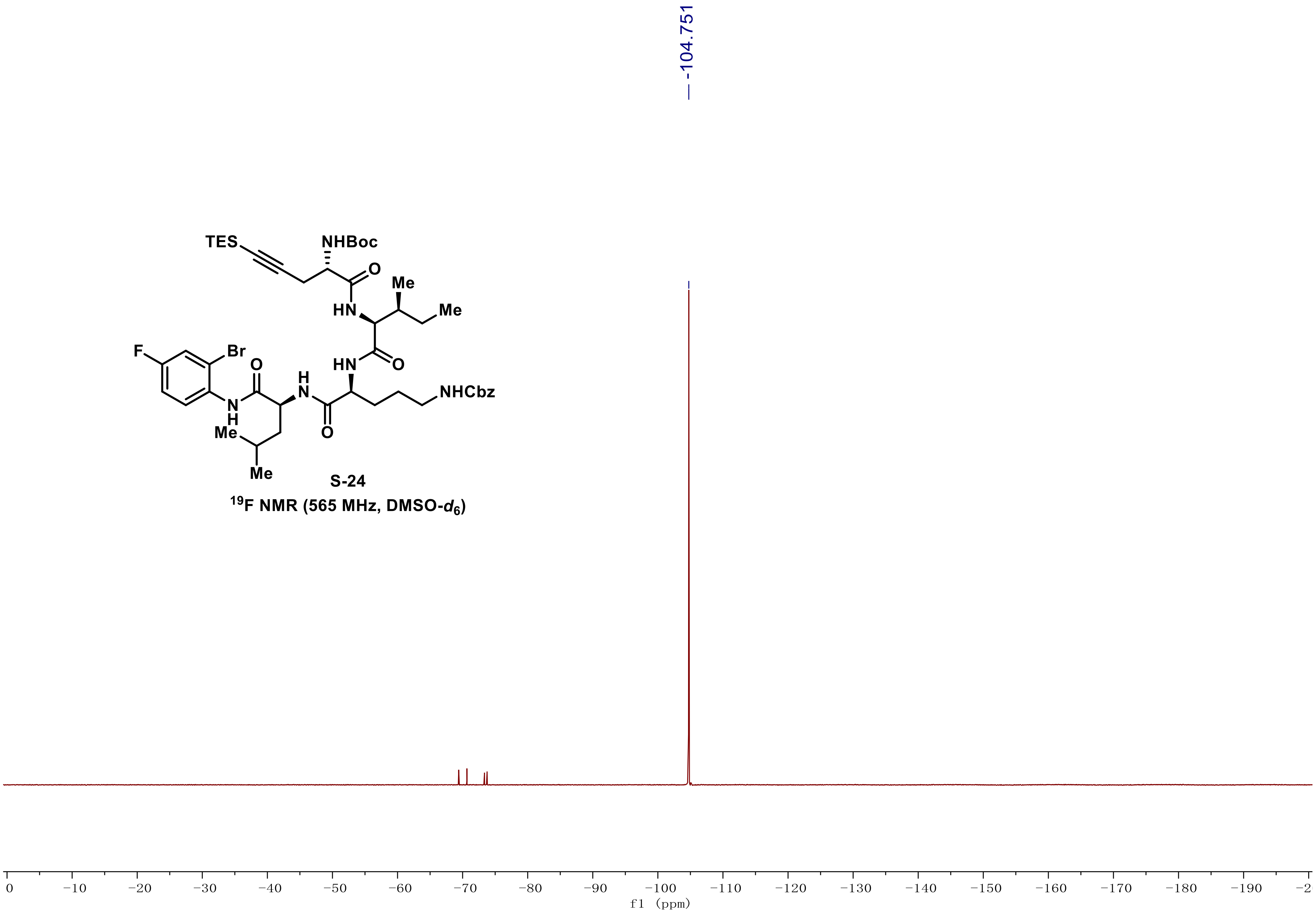

S-24

^{19}F NMR (565 MHz, DMSO-d_6)

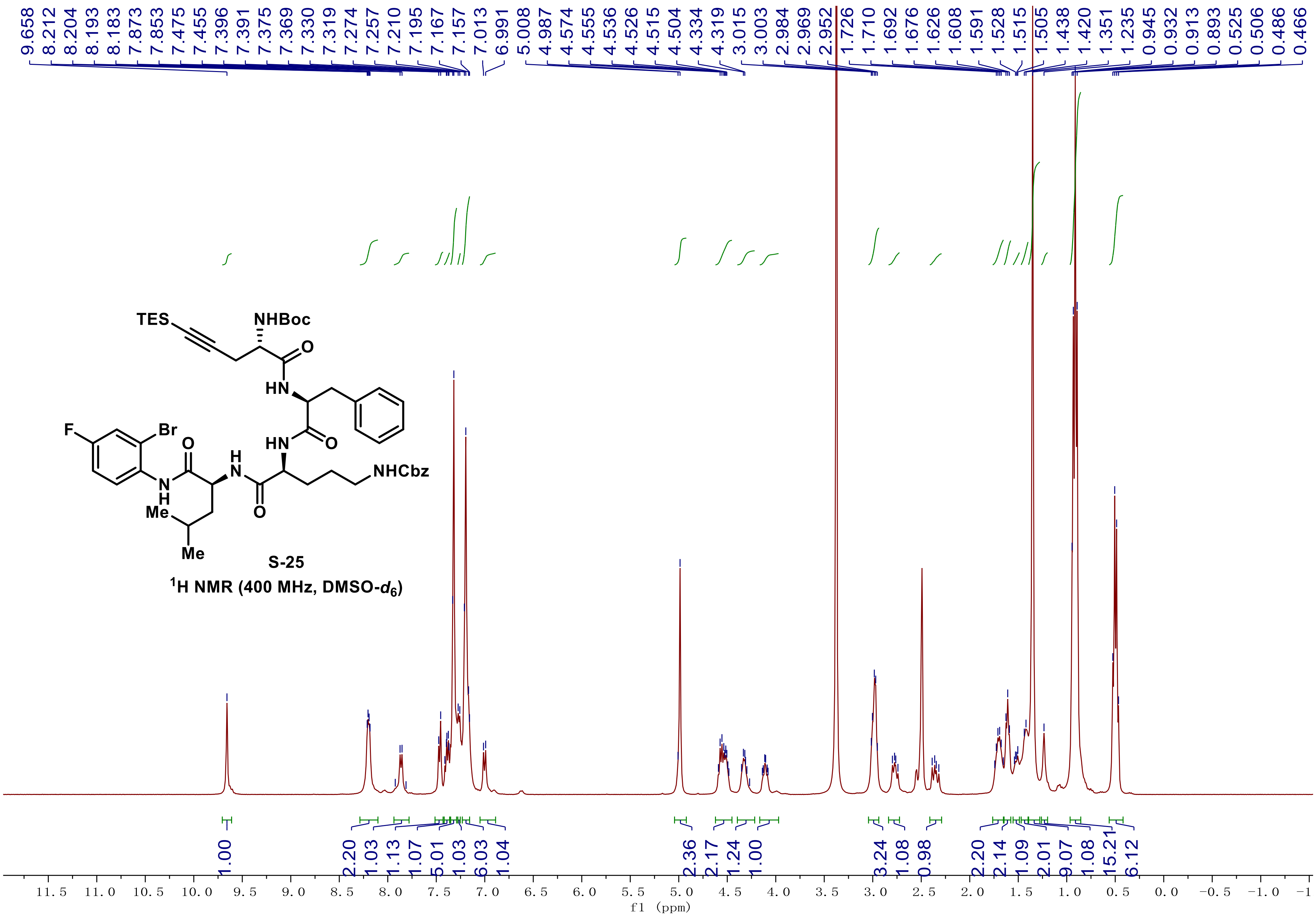

S-25

^{1}H NMR (400 MHz, DMSO-d_6)

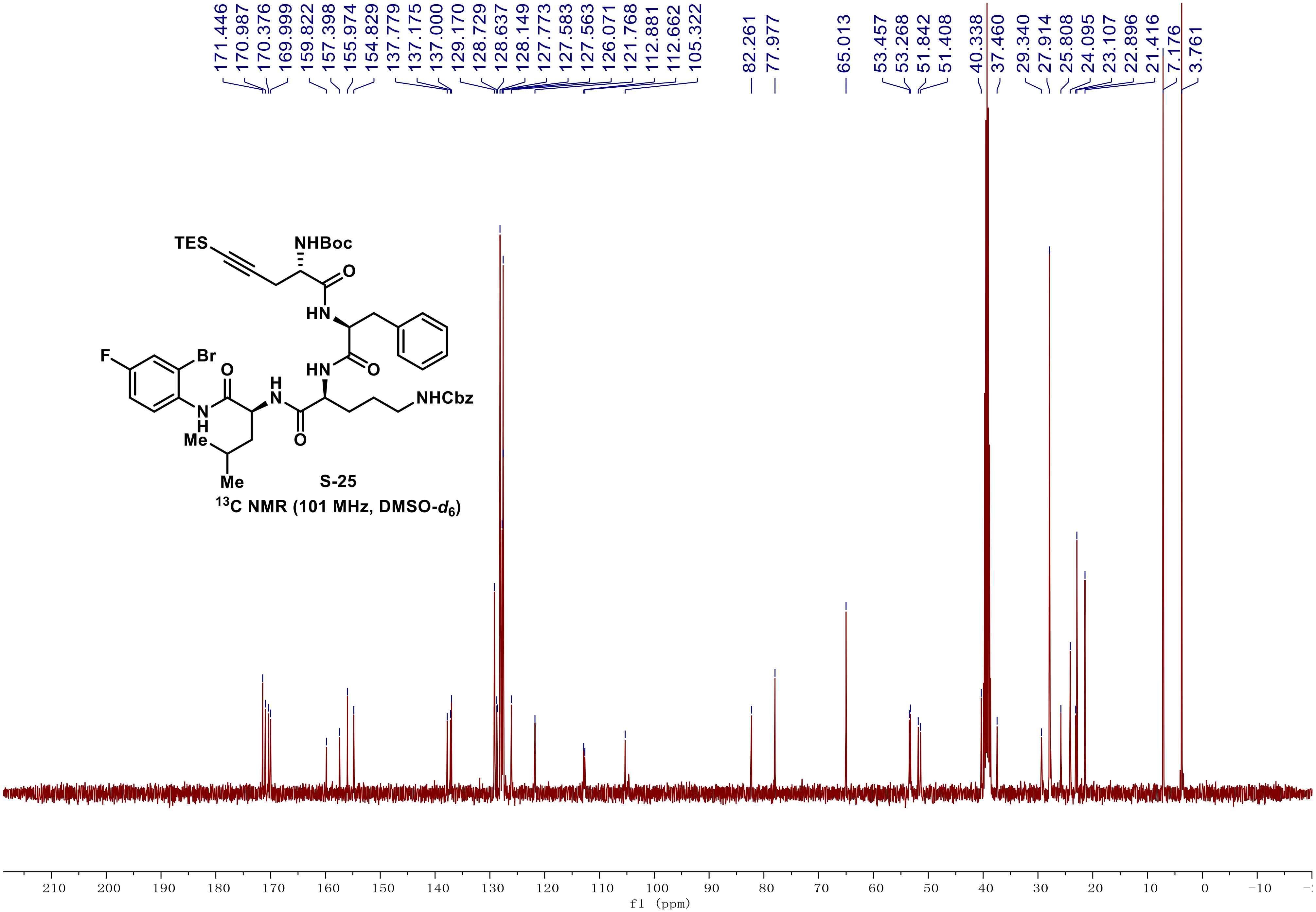
171.446
170.987
170.376
169.999
159.822
157.398
155.974
154.829
137.779
137.175
137.000
129.170
128.729
128.637
128.149
127.773
127.583
127.563
126.071
121.768
112.881
112.662
105.322
82.261
77.977
65.013
53.457
53.268
51.842
51.408
40.338
37.460
29.340
27.914
25.808
24.095
23.107
22.896
21.416
7.176
3.761
TES
NHBoc
O
HN
F
Br
O
H
N
HN
O
NHCbz
N
H
Me
O
Me
S-25
^{13}C NMR (101 MHz, DMSO-d_6)
210 200 190 180 170 160 150 140 130 120 110 100 90 80 70 60 50 40 30 20 10 0 −10
f1 (ppm)

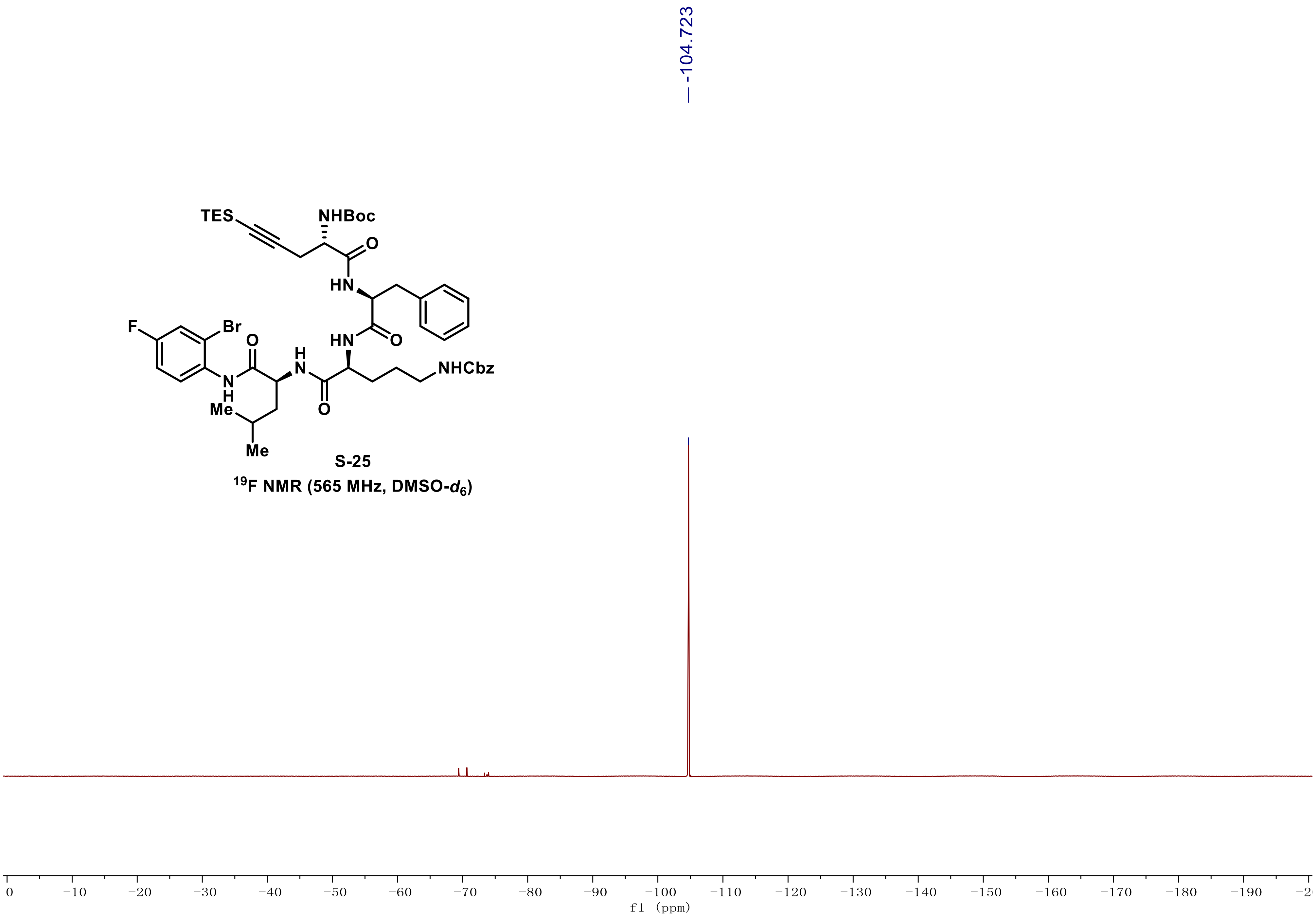

S-25

^{19}F NMR (565 MHz, DMSO-d_6)

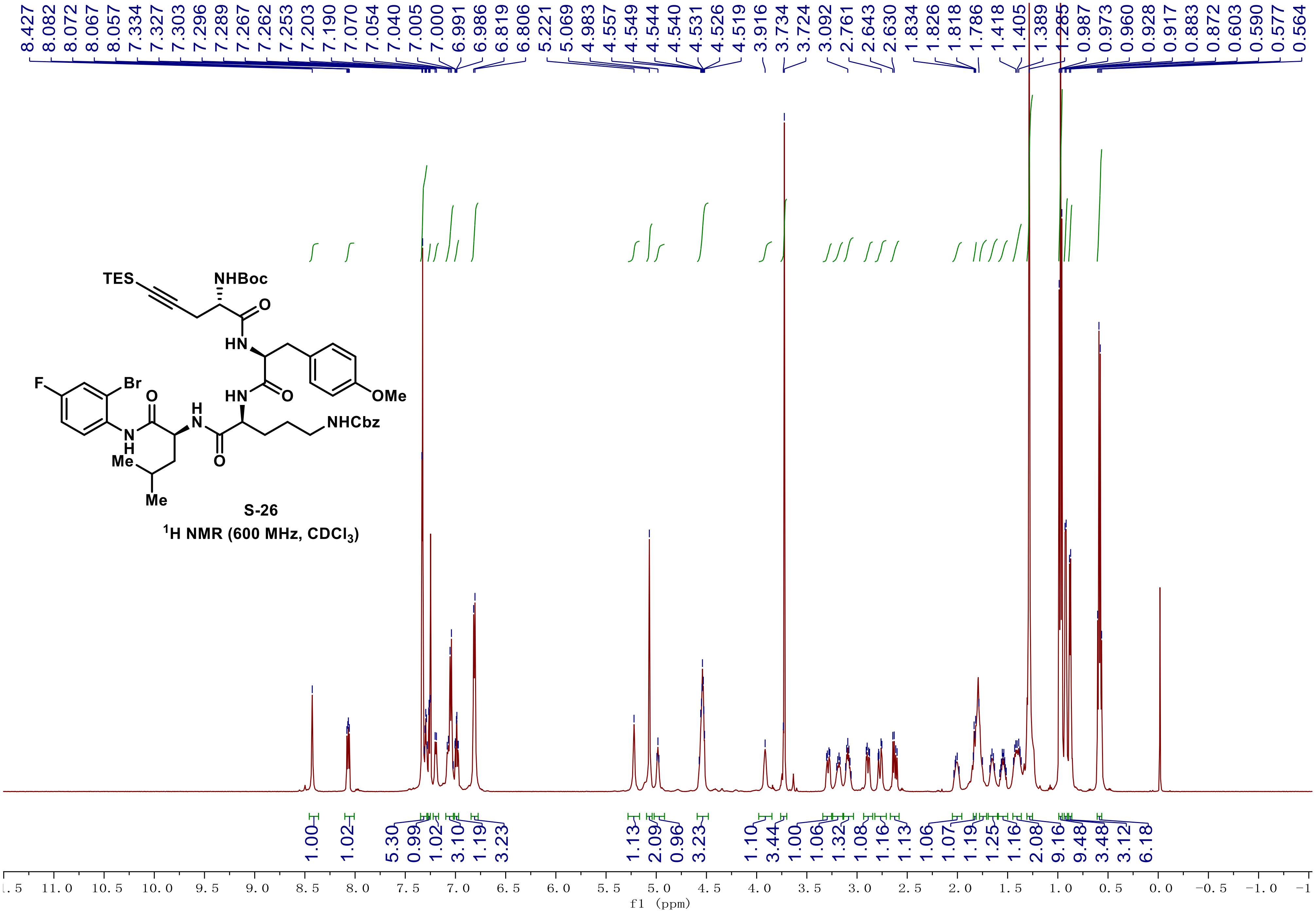

S-26

^{1}H NMR (600 MHz, $CDCl_3$)

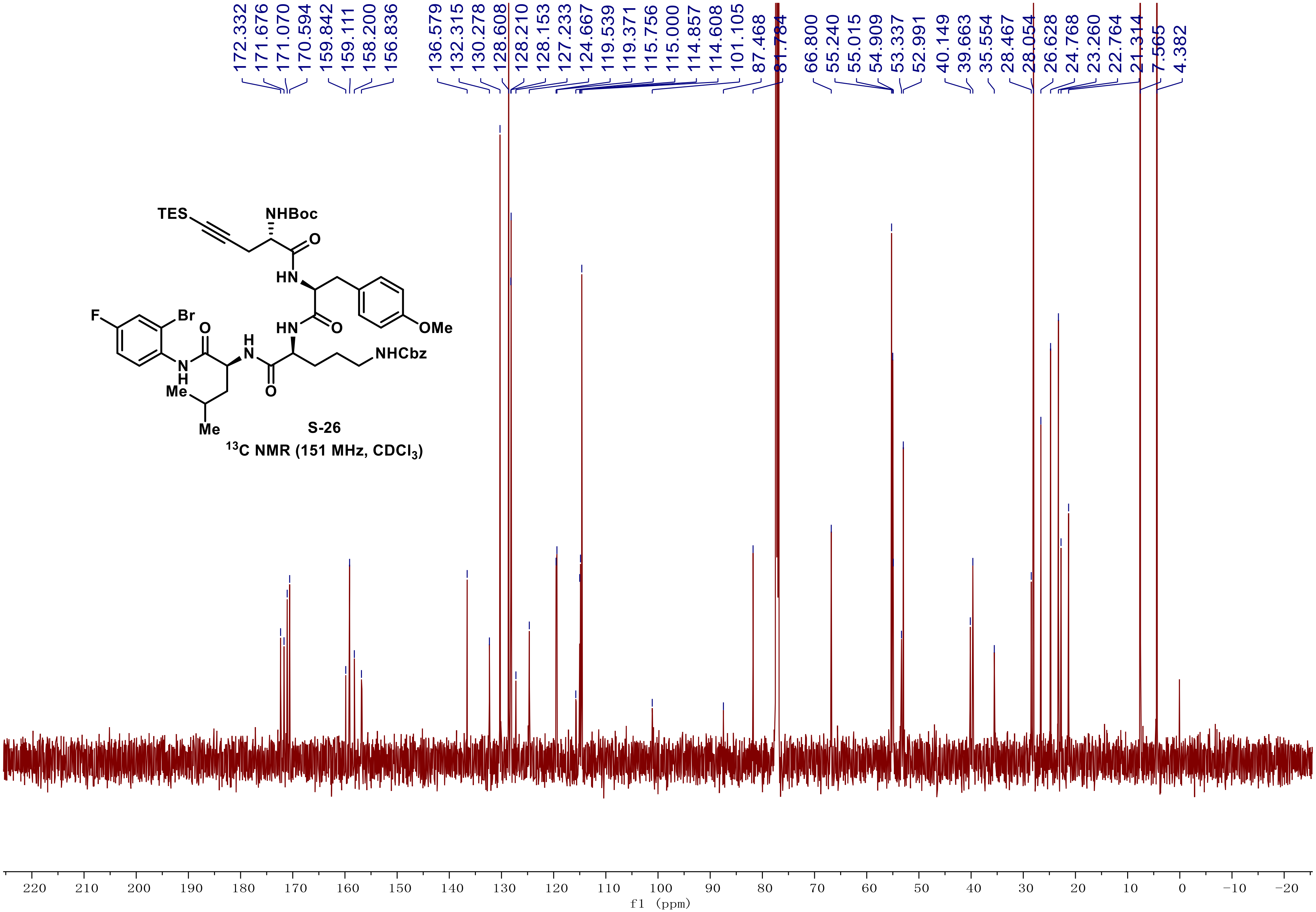
172.332
171.676
171.070
170.594
159.842
159.111
158.200
156.836
136.579
132.315
130.278
128.608
128.210
128.153
127.233
124.667
119.539
119.371
115.756
115.000
114.857
114.608
101.105
87.468
81.784
66.800
55.240
55.015
54.909
53.337
52.991
40.149
39.663
35.554
28.467
28.054
26.628
24.768
23.260
22.764
21.314
7.565
4.382
TES
NHBoc
HN
OMe
F
Br
NHCbz
Me
S-26
^{13}C NMR (151 MHz, $CDCl_3$)
f1 (ppm)

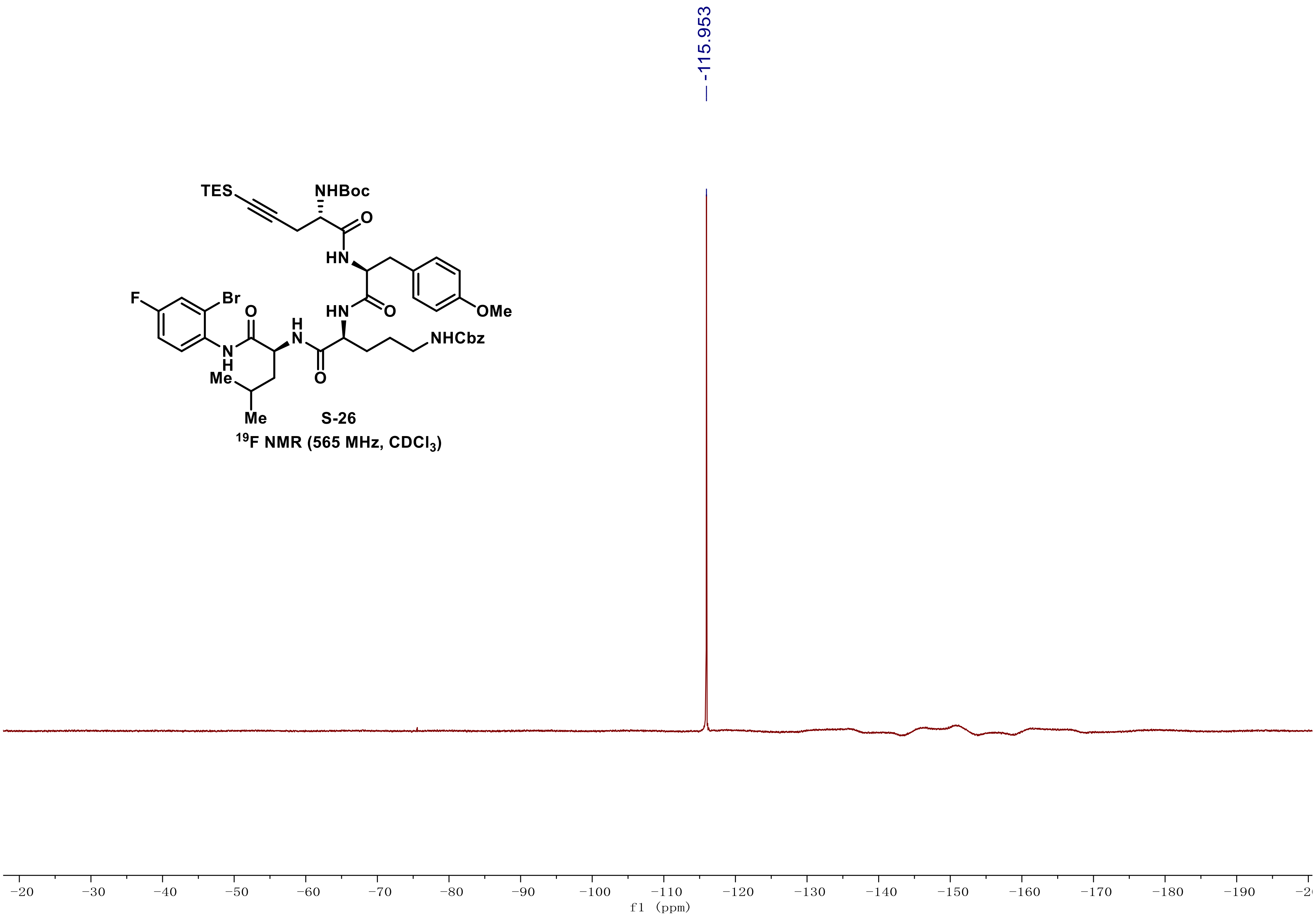
-115.953
TES
NHBoc
O
HN
F
Br
O
H
N
HN
O
OMe
N
H
NHCbz
Me
O
Me
S-26
^{19}F NMR (565 MHz, $CDCl_3$)
−20 −30 −40 −50 −60 −70 −80 −90 −100 −110 −120 −130 −140 −150 −160 −170 −180 −190
f1 (ppm)

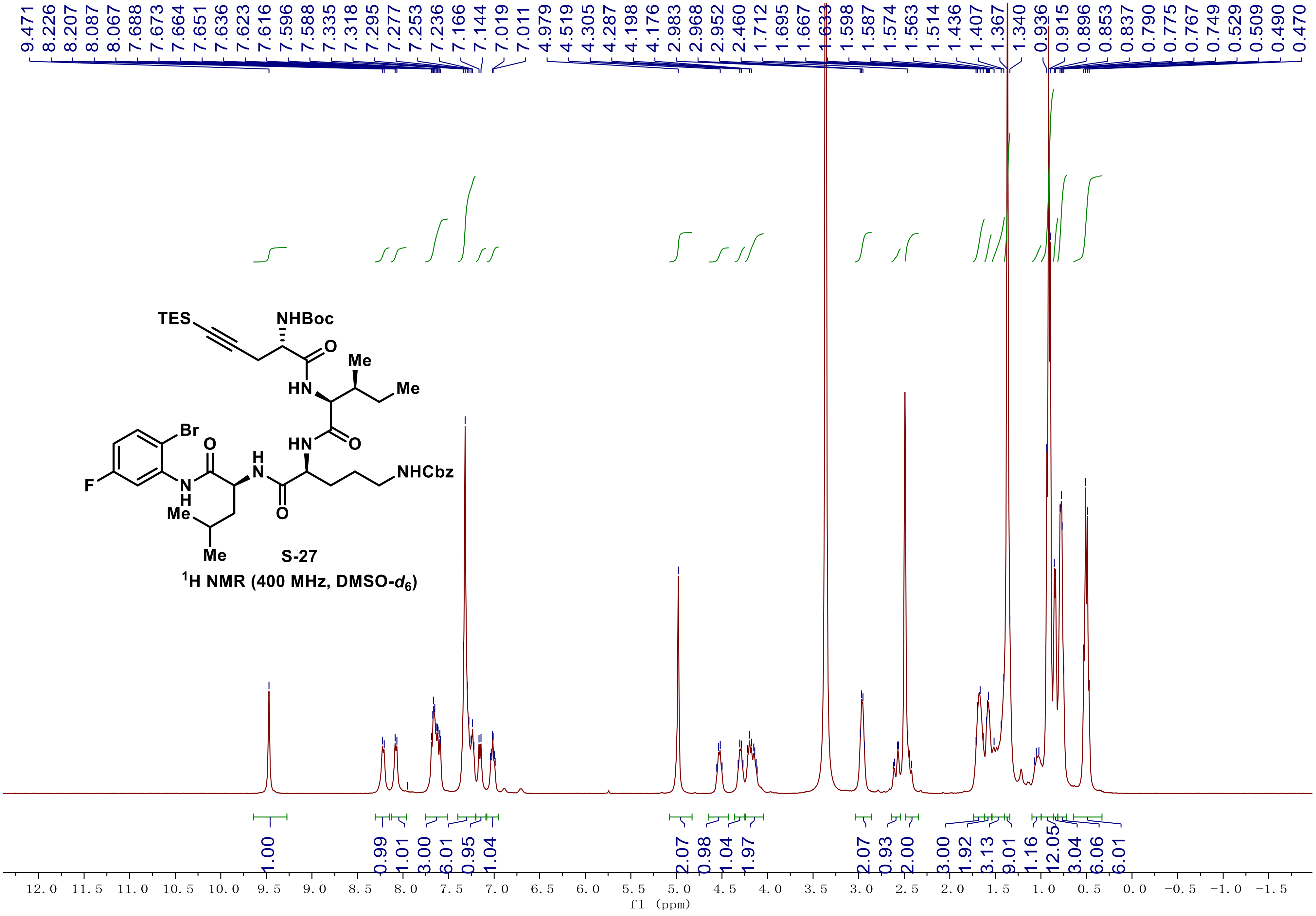
TES
NHBoc
O
Me
HN
Me
Br
O
HN
O
H
N
NHCbz
F
N
H
Me
O
Me
S-27
^{1}H NMR (400 MHz, DMSO-d_6)
f1 (ppm)

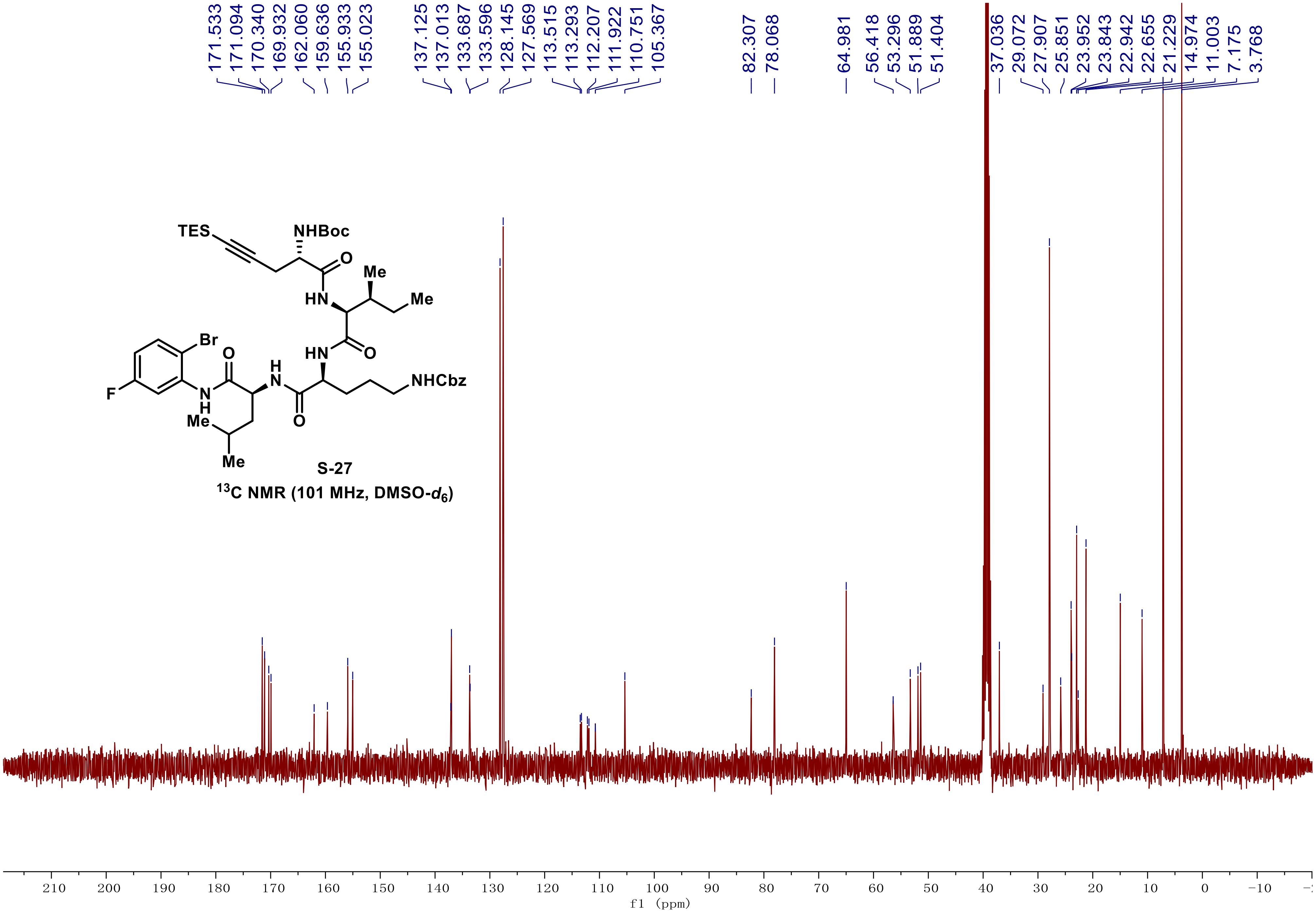

S-27

^{13}C NMR (101 MHz, DMSO-d_6)

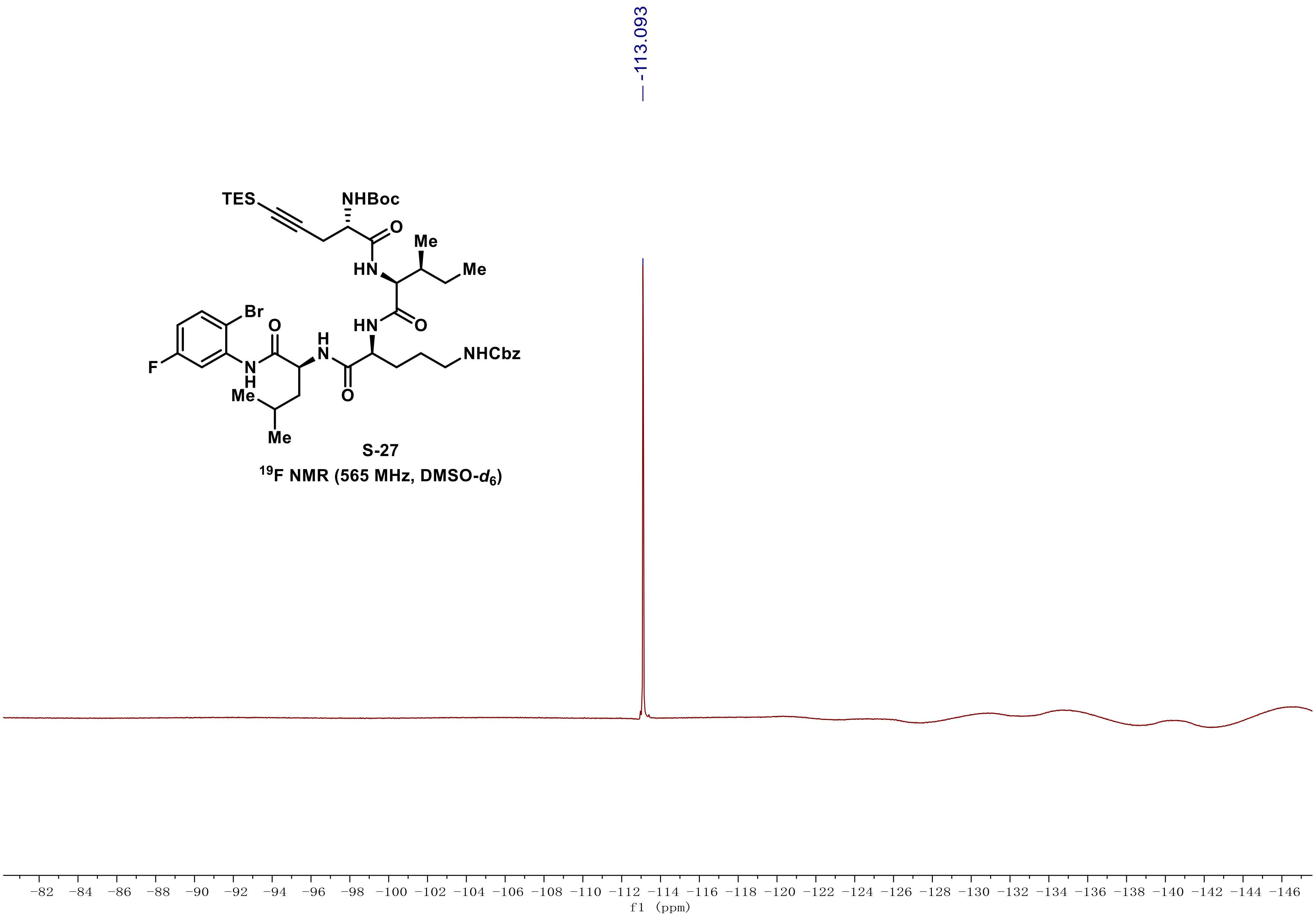

S-27

^{19}F NMR (565 MHz, DMSO-d_6)

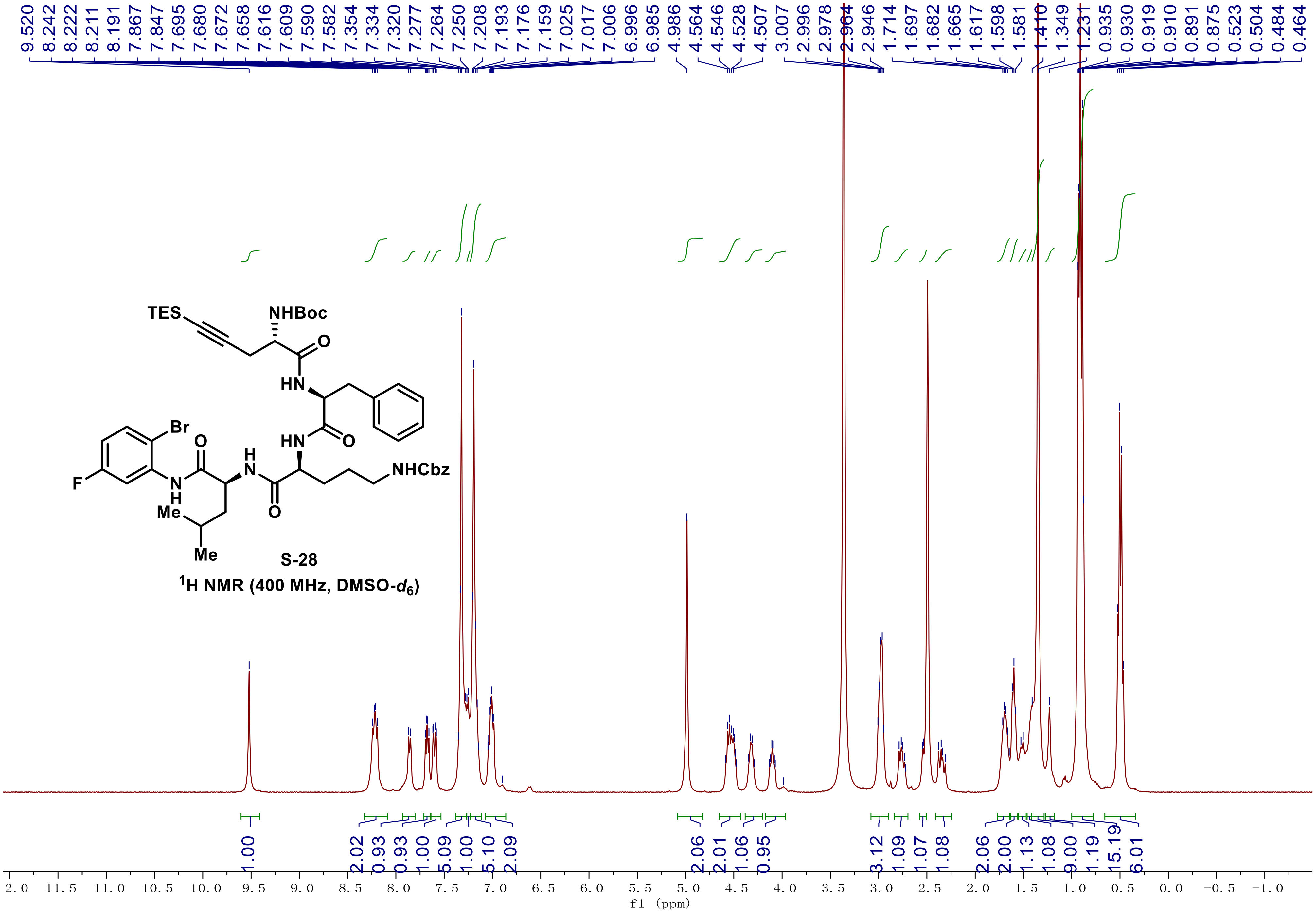
9.520 8.242 8.222 8.211 8.191 7.867 7.847 7.695 7.680 7.672 7.658 7.616 7.609 7.590 7.582 7.354 7.334 7.320 7.277 7.264 7.250 7.208 7.193 7.176 7.159 7.025 7.017 7.006 6.996 6.985 4.986 4.564 4.546 4.528 4.507 3.007 2.996 2.978 2.964 2.946 1.714 1.697 1.682 1.665 1.617 1.598 1.581 1.410 1.349 1.231 0.935 0.930 0.919 0.910 0.891 0.875 0.523 0.504 0.484 0.464
TES
NHBoc
O
HN
HN
O
Br
O
H
N
NHCbz
F
N
H
Me
O
Me
S-28
1H NMR (400 MHz, DMSO-d6)
1.00 2.02 0.93 0.93 1.00 5.09 1.00 5.10 2.09 2.06 2.01 1.06 0.95 3.12 1.09 1.07 1.08 2.06 2.00 1.13 1.08 9.00 1.19 15.19 6.01
2.0 11.5 11.0 10.5 10.0 9.5 9.0 8.5 8.0 7.5 7.0 6.5 6.0 5.5 5.0 4.5 4.0 3.5 3.0 2.5 2.0 1.5 1.0 0.5 0.0 -0.5 -1.0
f1 (ppm)

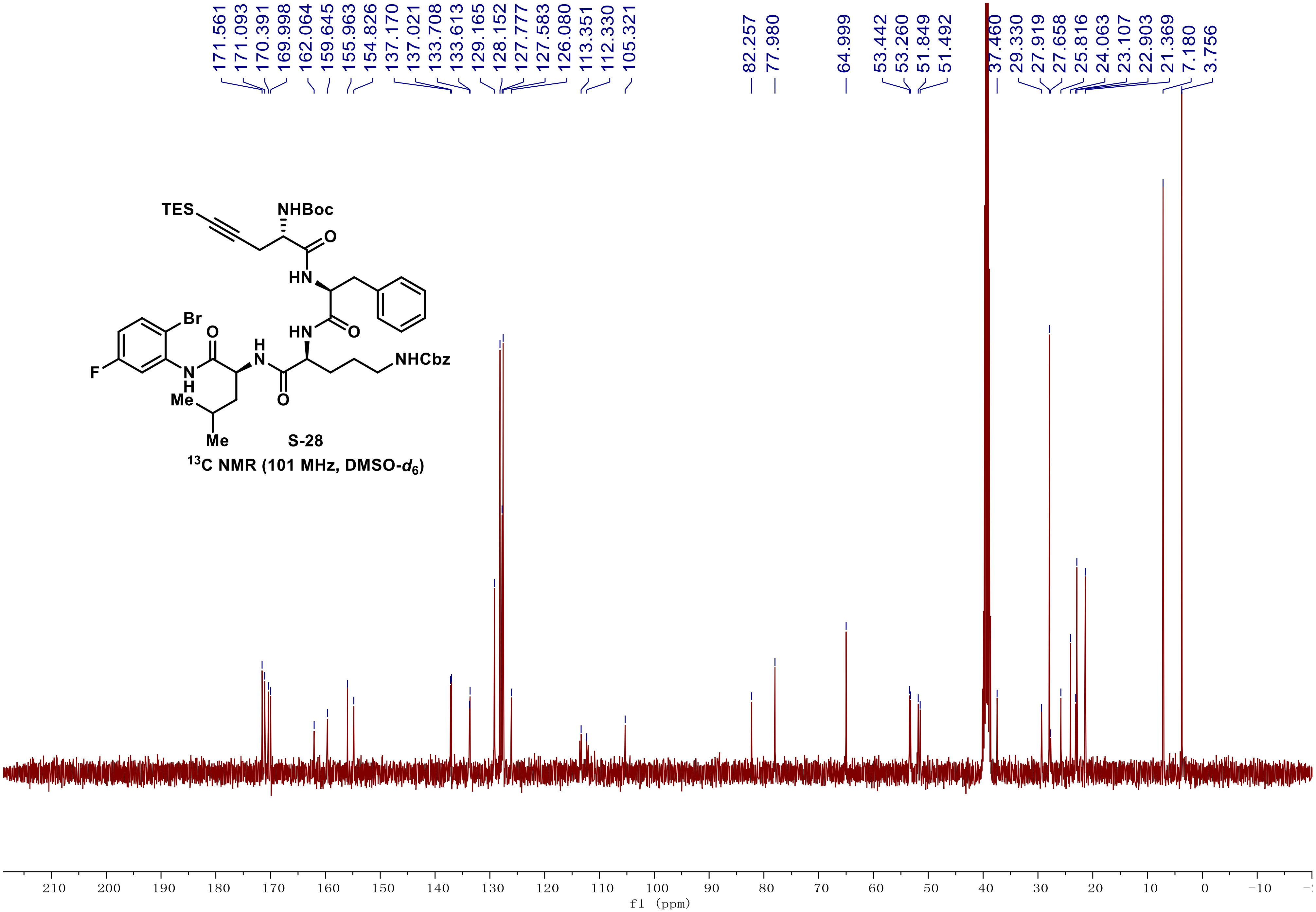

171.561
171.093
170.391
169.998
162.064
159.645
155.963
154.826
137.170
137.021
133.708
133.613
129.165
128.152
127.777
127.583
126.080
113.351
112.330
105.321
82.257
77.980
64.999
53.442
53.260
51.849
51.492
37.460
29.330
27.919
27.658
25.816
24.063
23.107
22.903
21.369
7.180
3.756
TES
NHBoc
O
HN
HN
O
Br
O
H
N
NHCbz
F
N
H
O
Me
Me
S-28
13C NMR (101 MHz, DMSO-d6)
210
200
190
180
170
160
150
140
130
120
110
100
90
80
70
60
50
40
30
20
10
0
-10
f1 (ppm)

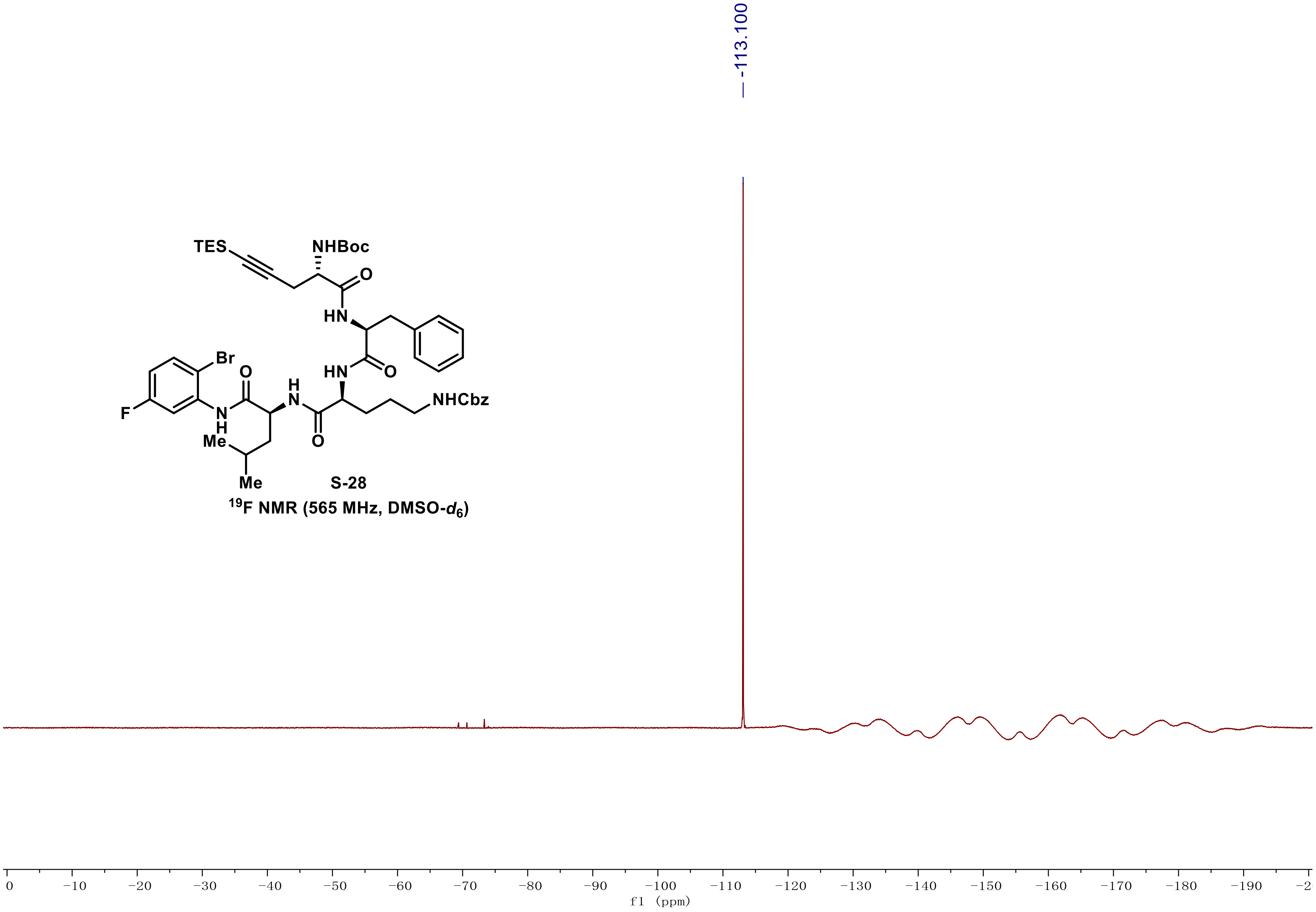
-113.100
TES
NHBoc
O
HN
Br
O
H
N
HN
O
NHCbz
F
N
H
Me
O
Me
S-28
19F NMR (565 MHz, DMSO-d6)
0
−10
−20
−30
−40
−50
−60
−70
−80
−90
−100
−110
−120
−130
−140
−150
−160
−170
−180
−190
f1 (ppm)

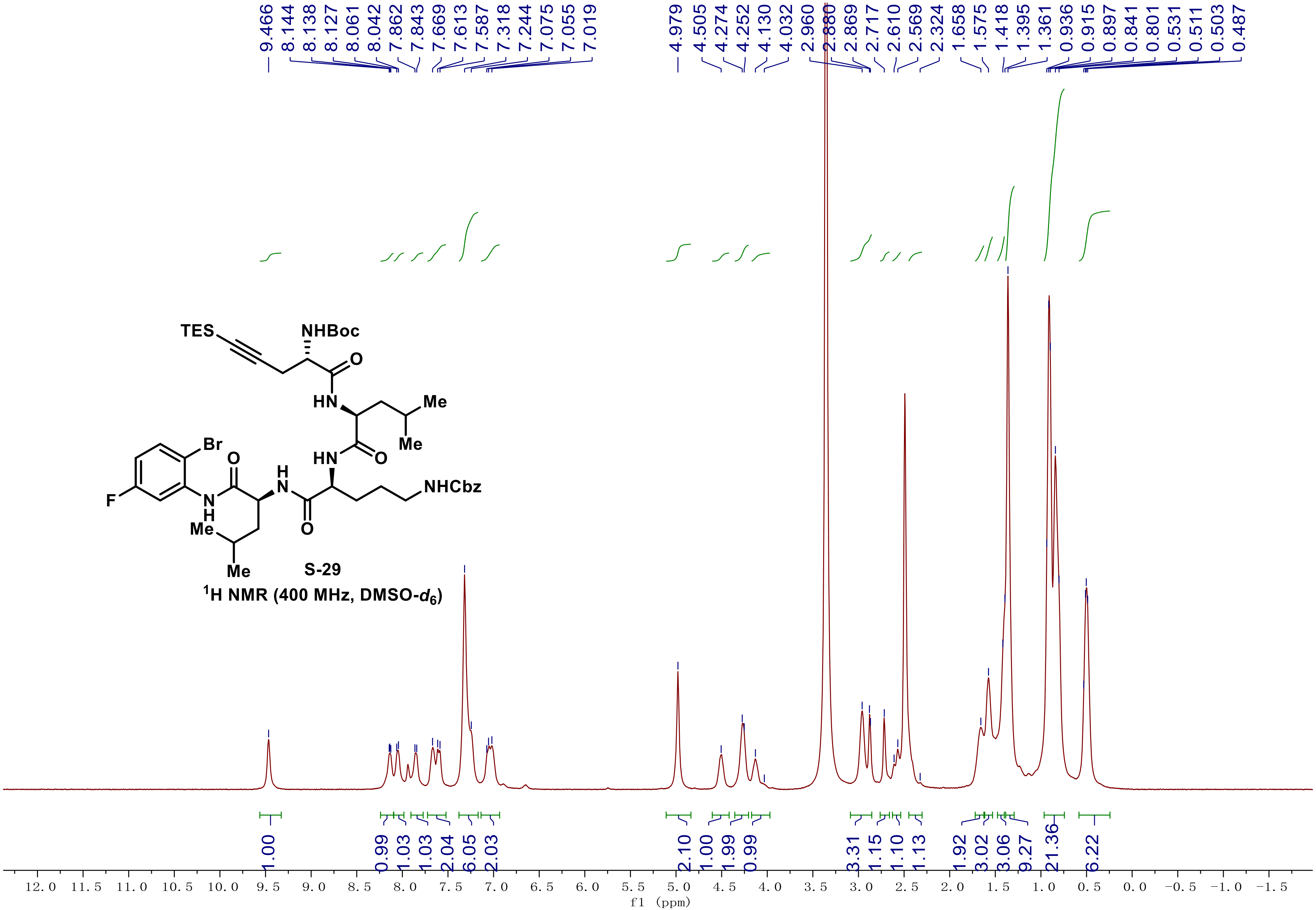

S-29

^{1}H NMR (400 MHz, DMSO-d_6)

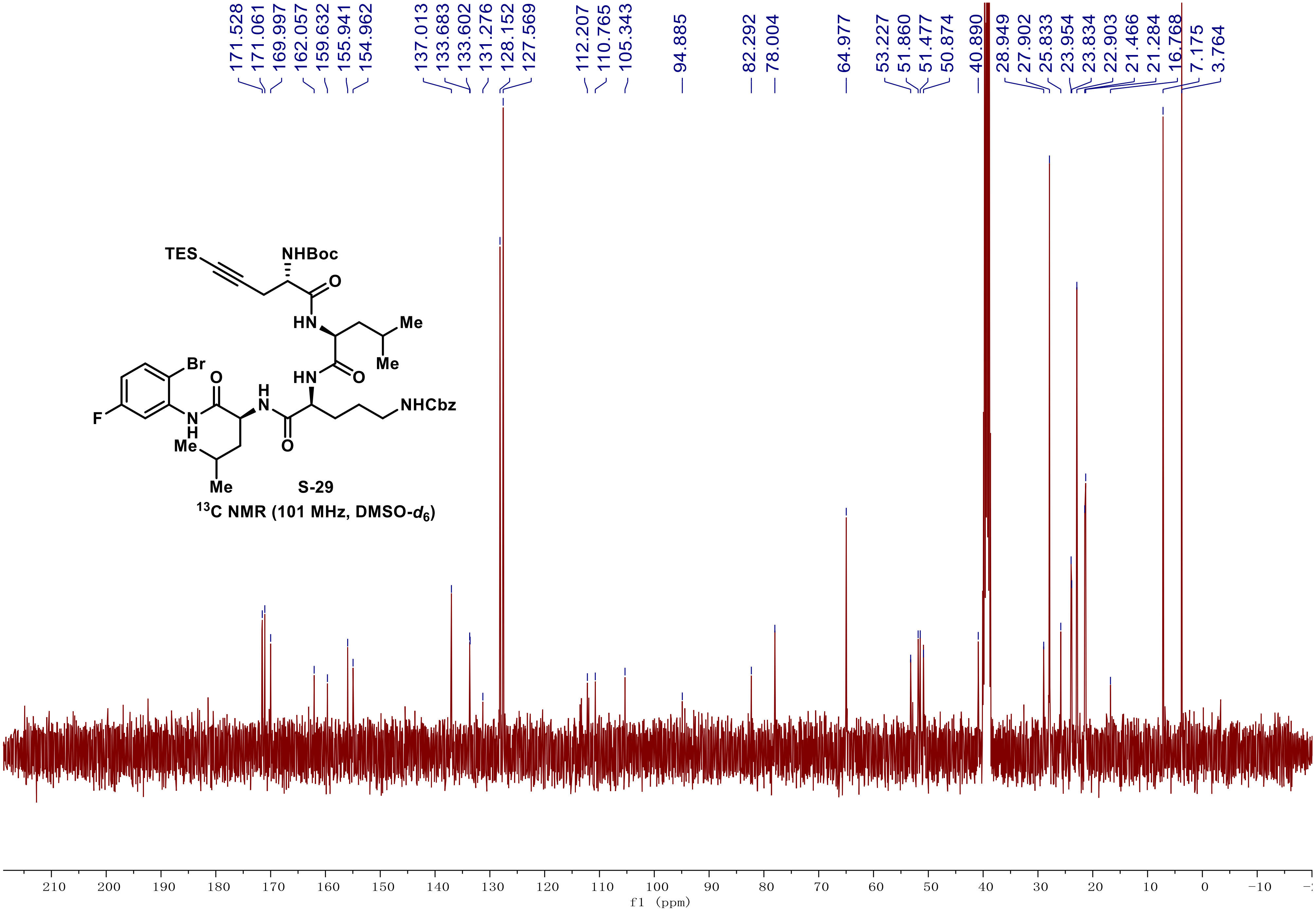
171.528
171.061
169.997
162.057
159.632
155.941
154.962
137.013
133.683
133.602
131.276
128.152
127.569
112.207
110.765
105.343
94.885
82.292
78.004
64.977
53.227
51.860
51.477
50.874
40.890
28.949
27.902
25.833
23.954
23.834
22.903
21.466
21.284
16.768
7.175
3.764
TES
NHBoc
O
HN
Me
Me
Br
O
H
N
HN
O
NHCbz
F
N
H
Me
O
Me
S-29
^{13}C NMR (101 MHz, DMSO-d_6)
f1 (ppm)

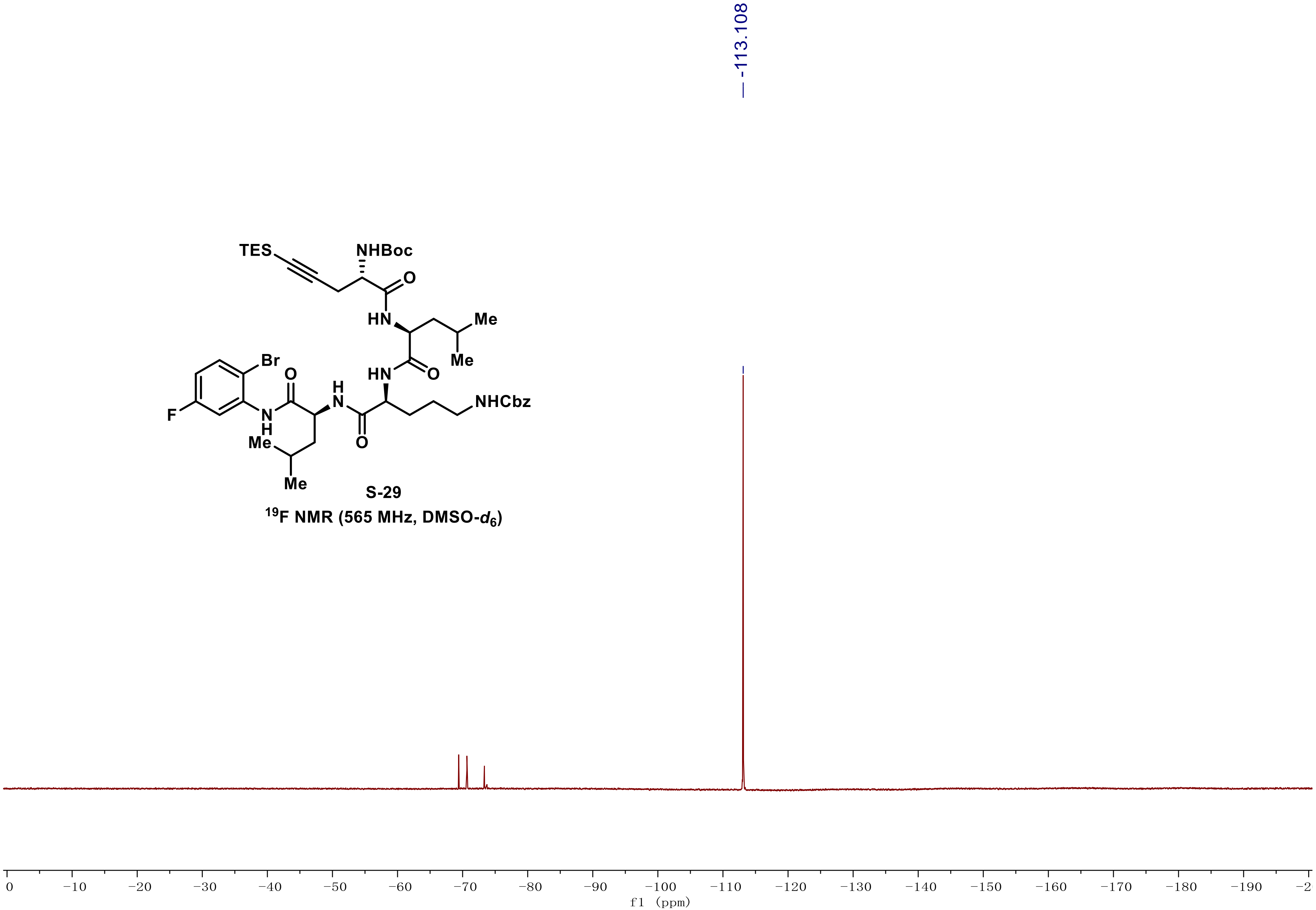
-113.108
TES
NHBoc
O
HN
Me
Me
Br
O
H
N
HN
O
NHCbz
F
N
H
Me
O
Me
S-29
19F NMR (565 MHz, DMSO-d6)
0
-10
-20
-30
-40
-50
-60
-70
-80
-90
-100
-110
-120
-130
-140
-150
-160
-170
-180
-190
f1 (ppm)

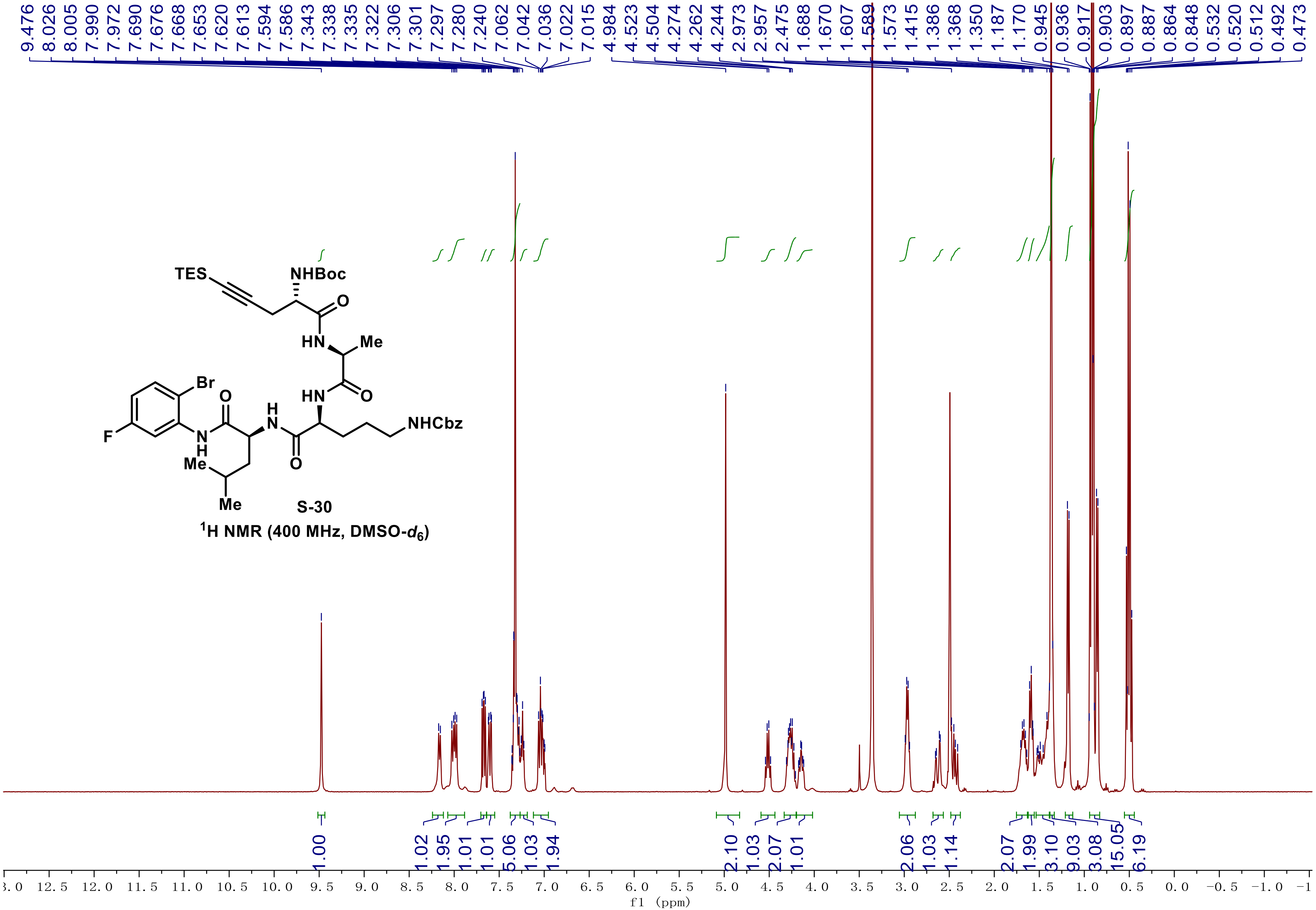

9.476 8.026 8.005 7.990 7.972 7.690 7.676 7.668 7.653 7.620 7.613 7.594 7.586 7.343 7.338 7.335 7.322 7.306 7.301 7.297 7.280 7.240 7.062 7.042 7.036 7.022 7.015 4.984 4.523 4.504 4.274 4.262 4.244 2.973 2.957 2.475 1.688 1.670 1.607 1.589 1.573 1.415 1.386 1.368 1.350 1.187 1.170 0.945 0.936 0.917 0.903 0.897 0.887 0.864 0.848 0.532 0.520 0.512 0.492 0.473
TES
NHBoc
O
HN
Me
Br
HN
O
H
N
O
F
N
H
NHCbz
Me
O
Me
S-30
1H NMR (400 MHz, DMSO-d6)
1.00 1.02 1.95 1.01 1.01 5.06 1.03 1.94 2.10 1.03 2.07 1.01 2.06 1.03 1.14 2.07 1.99 3.10 9.03 3.08 15.05 6.19
f1 (ppm)

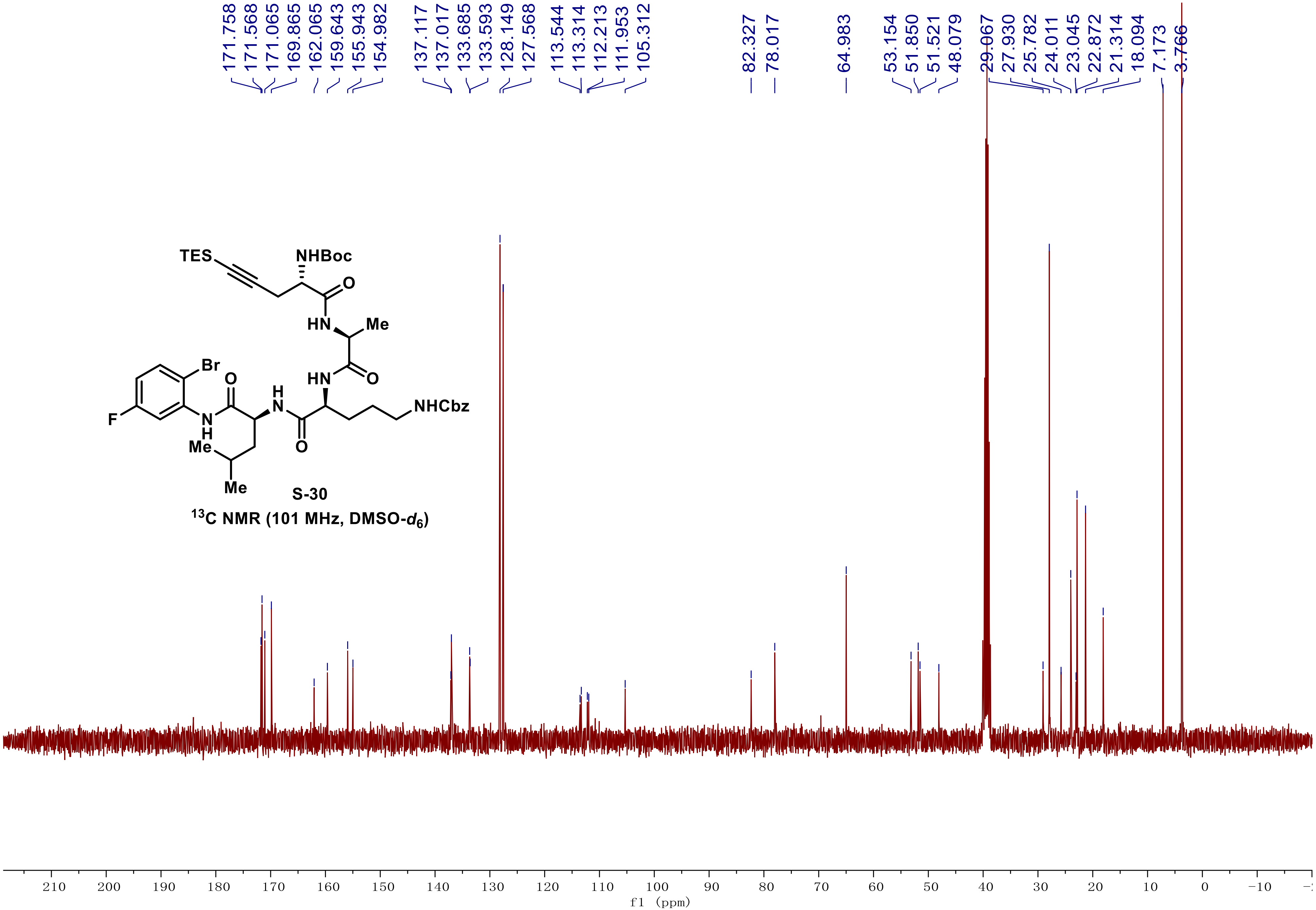
171.758
171.568
171.065
169.865
162.065
159.643
155.943
154.982
137.117
137.017
133.685
133.593
128.149
127.568
113.544
113.314
112.213
111.953
105.312
82.327
78.017
64.983
53.154
51.850
51.521
48.079
29.067
27.930
25.782
24.011
23.045
22.872
21.314
18.094
7.173
3.766
TES
NHBoc
O
HN
Me
Br
O
HN
O
H
N
NHCbz
F
N
H
Me
O
Me
S-30
13C NMR (101 MHz, DMSO-d6)
f1 (ppm)

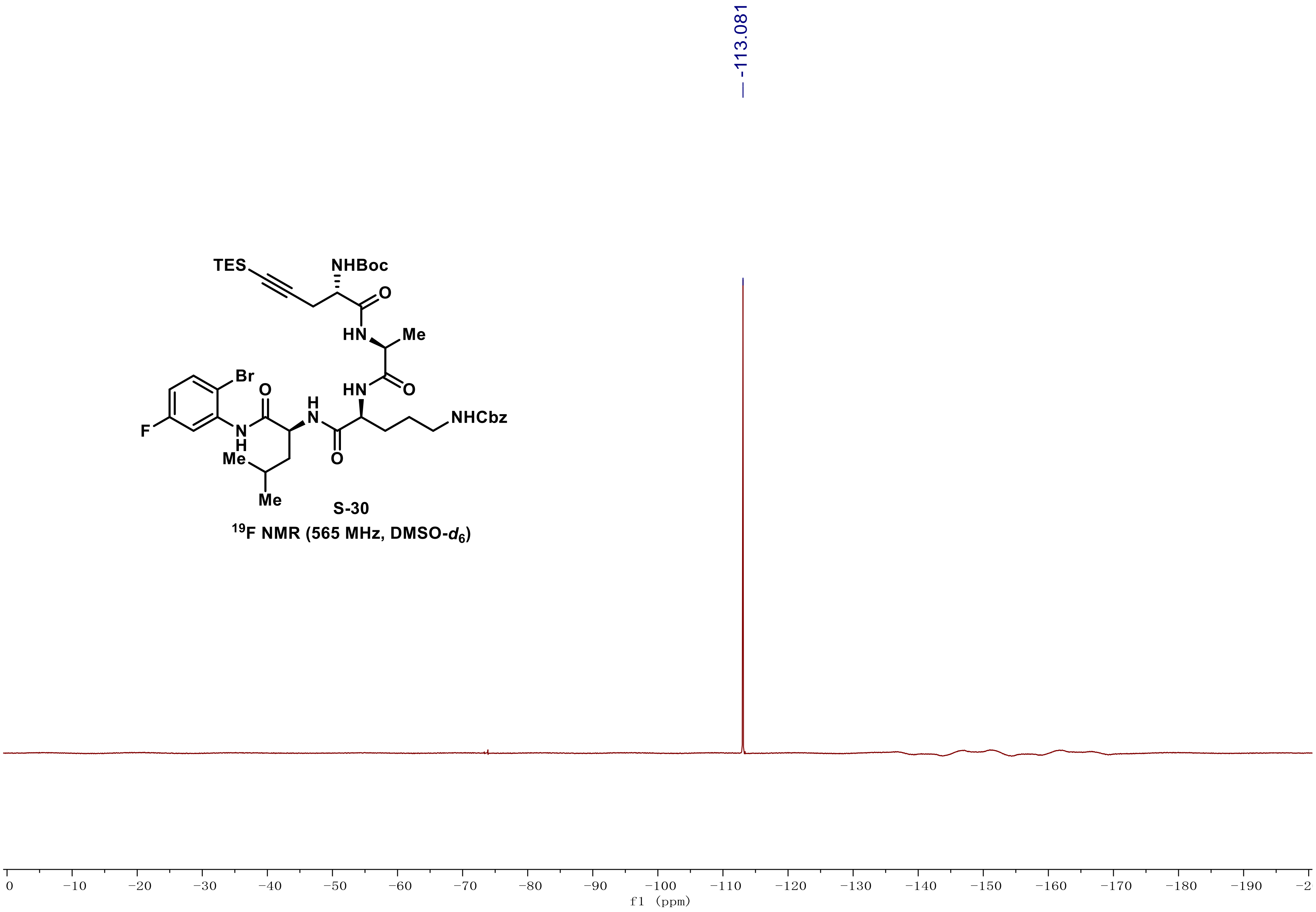

-113.081
TES
NHBoc
O
HN
Me
Br
O
H
N
HN
O
NHCbz
F
N
H
Me
O
Me
S-30
19F NMR (565 MHz, DMSO-d6)
0
−10
−20
−30
−40
−50
−60
−70
−80
−90
−100
−110
−120
−130
−140
−150
−160
−170
−180
−190
f1 (ppm)

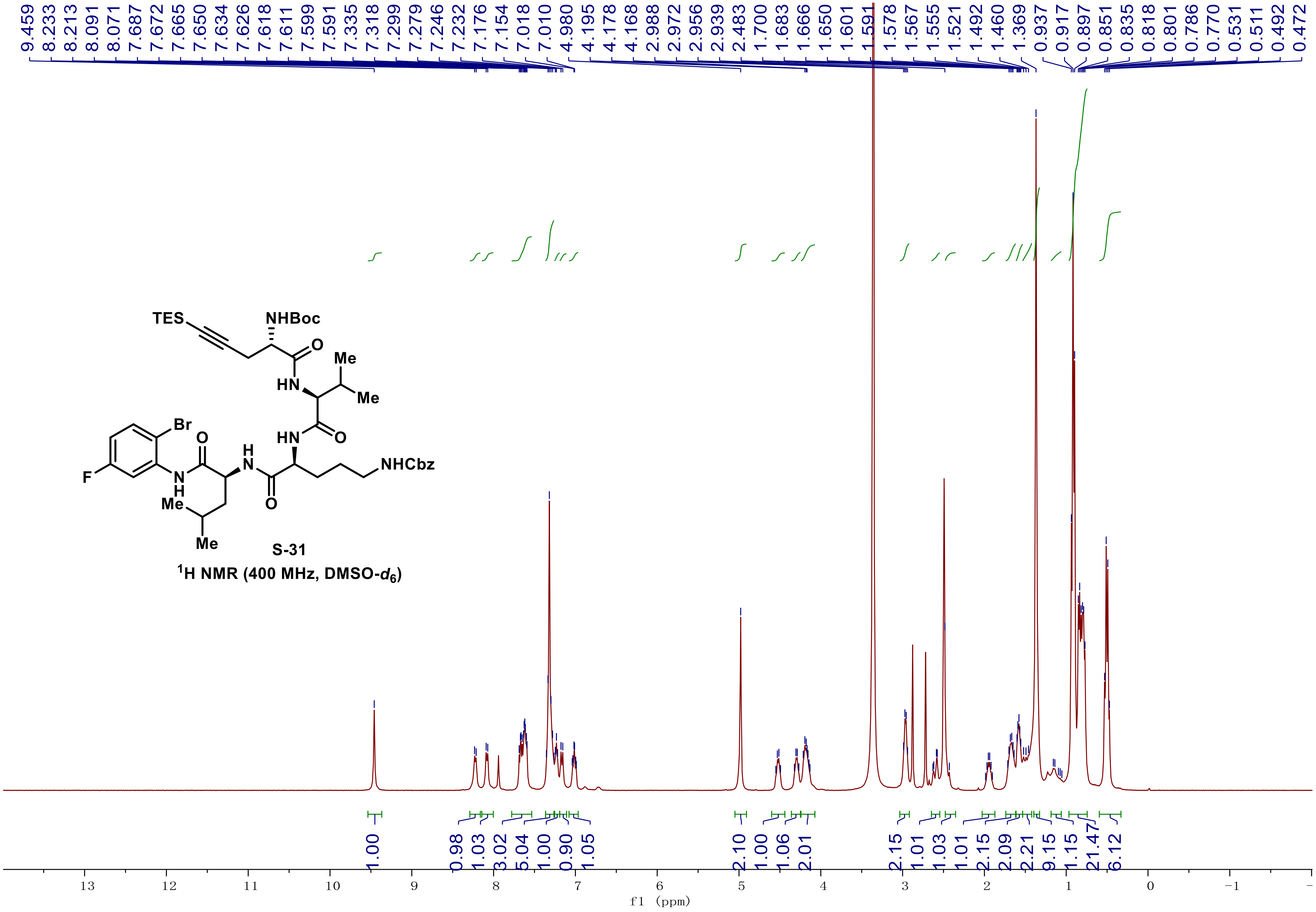

9.459 8.233 8.213 8.091 8.071 7.687 7.672 7.665 7.650 7.634 7.626 7.618 7.611 7.599 7.591 7.335 7.318 7.299 7.279 7.246 7.232 7.176 7.154 7.018 7.010 4.980 4.195 4.178 4.168 2.988 2.972 2.956 2.939 2.483 1.700 1.683 1.666 1.650 1.601 1.591 1.578 1.567 1.555 1.521 1.492 1.460 1.369 0.937 0.917 0.897 0.851 0.835 0.818 0.801 0.786 0.770 0.531 0.511 0.492 0.472
TES
NHBoc
O
Me
HN
Me
Br
O
HN
O
H
N
NHCbz
F
N
H
Me
O
Me
S-31
^{1}H NMR (400 MHz, DMSO-d_6)
1.00 0.98 1.03 3.02 5.04 1.00 0.90 1.05 2.10 1.00 1.06 2.01 2.15 1.01 1.03 1.01 2.15 2.09 2.21 9.15 1.15 21.47 6.12
13 12 11 10 9 8 7 6 5 4 3 2 1 0 −1
f1 (ppm)

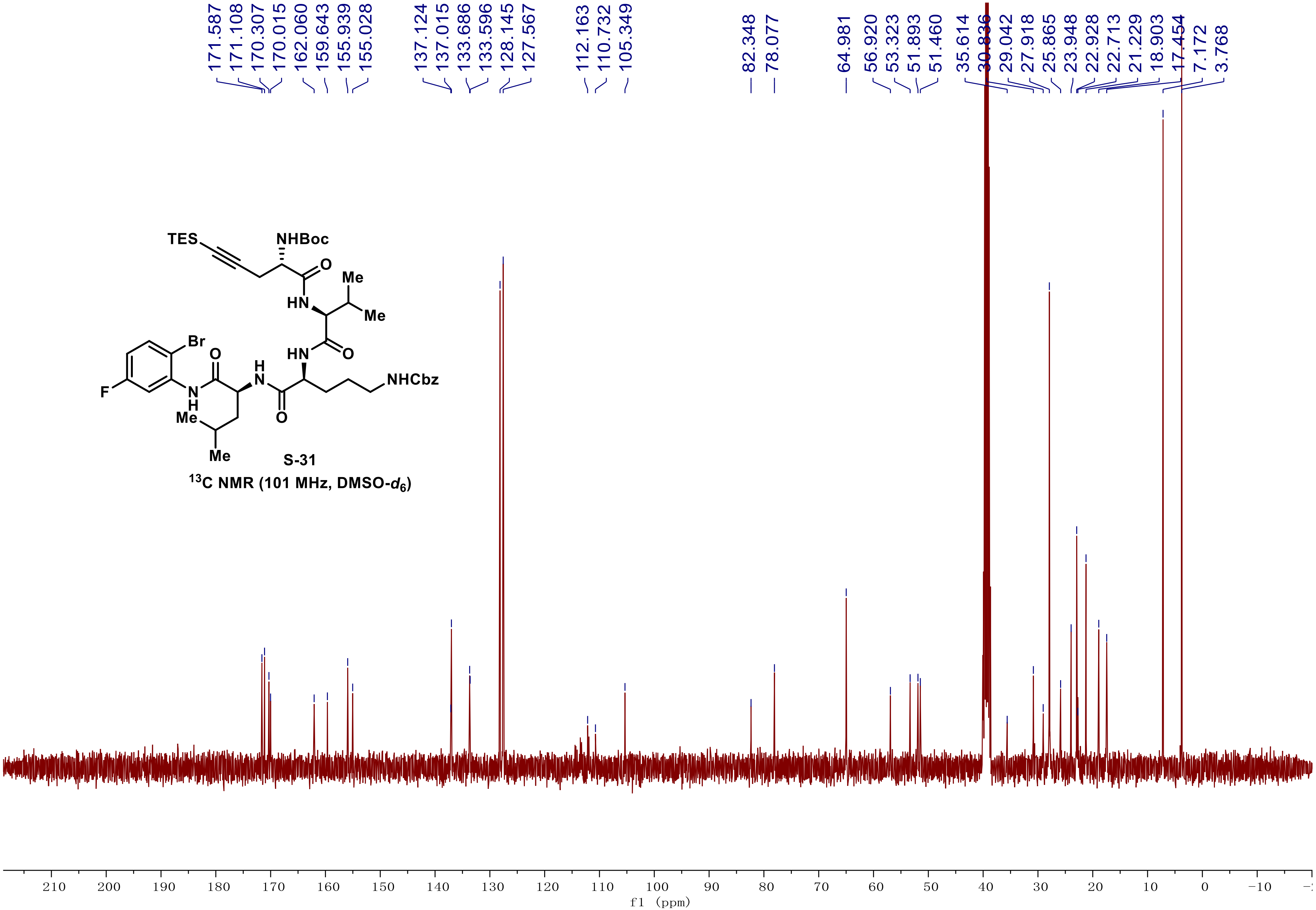

S-31

^{13}C NMR (101 MHz, DMSO-d_6)

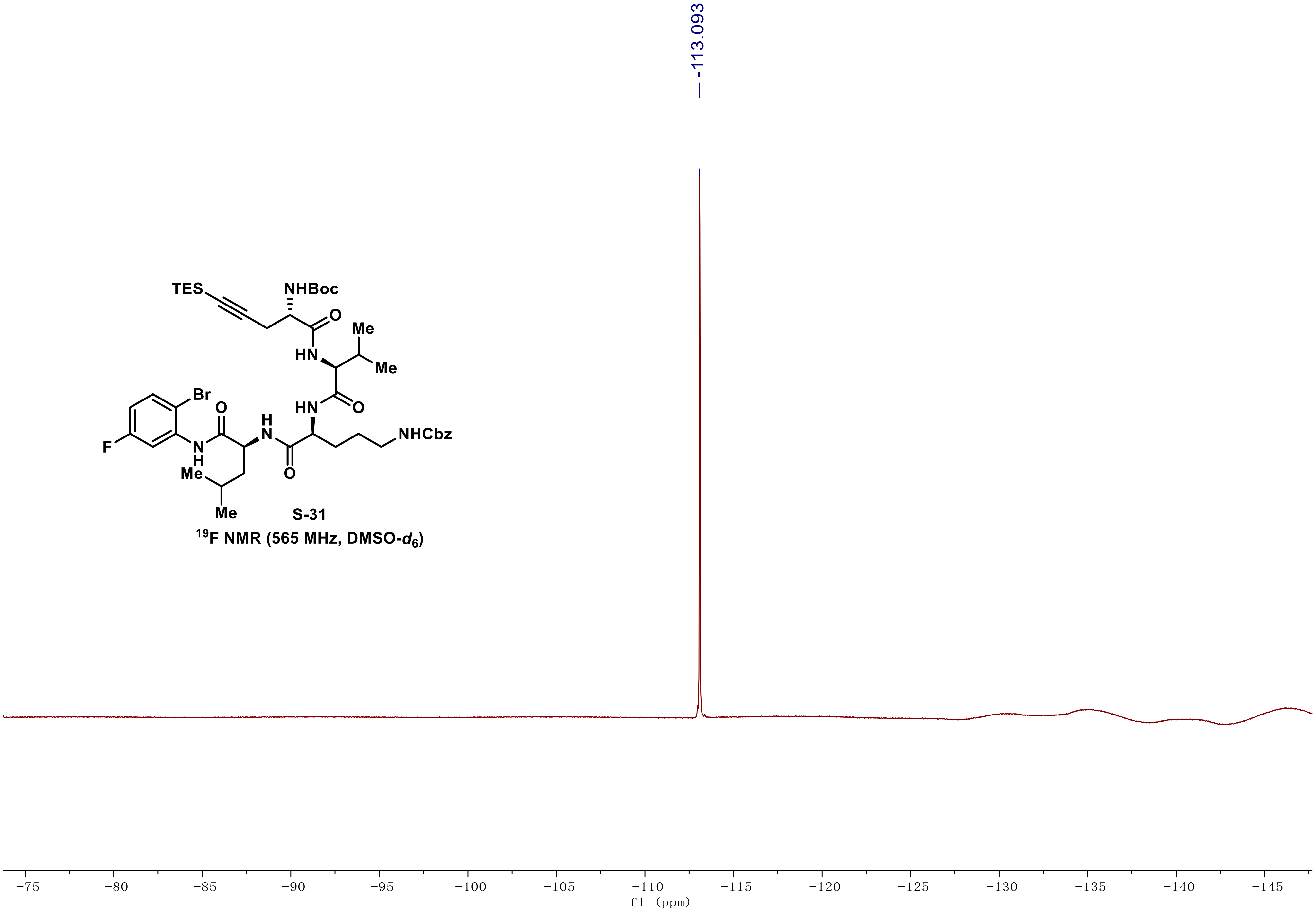
-113.093
TES
NHBoc
O
Me
HN
Me
Br
O
HN
O
H
N
NHCbz
F
N
H
Me
O
Me
S-31
19F NMR (565 MHz, DMSO-d6)
−75
−80
−85
−90
−95
−100
−105
−110
−115
−120
−125
−130
−135
−140
−145
f1 (ppm)

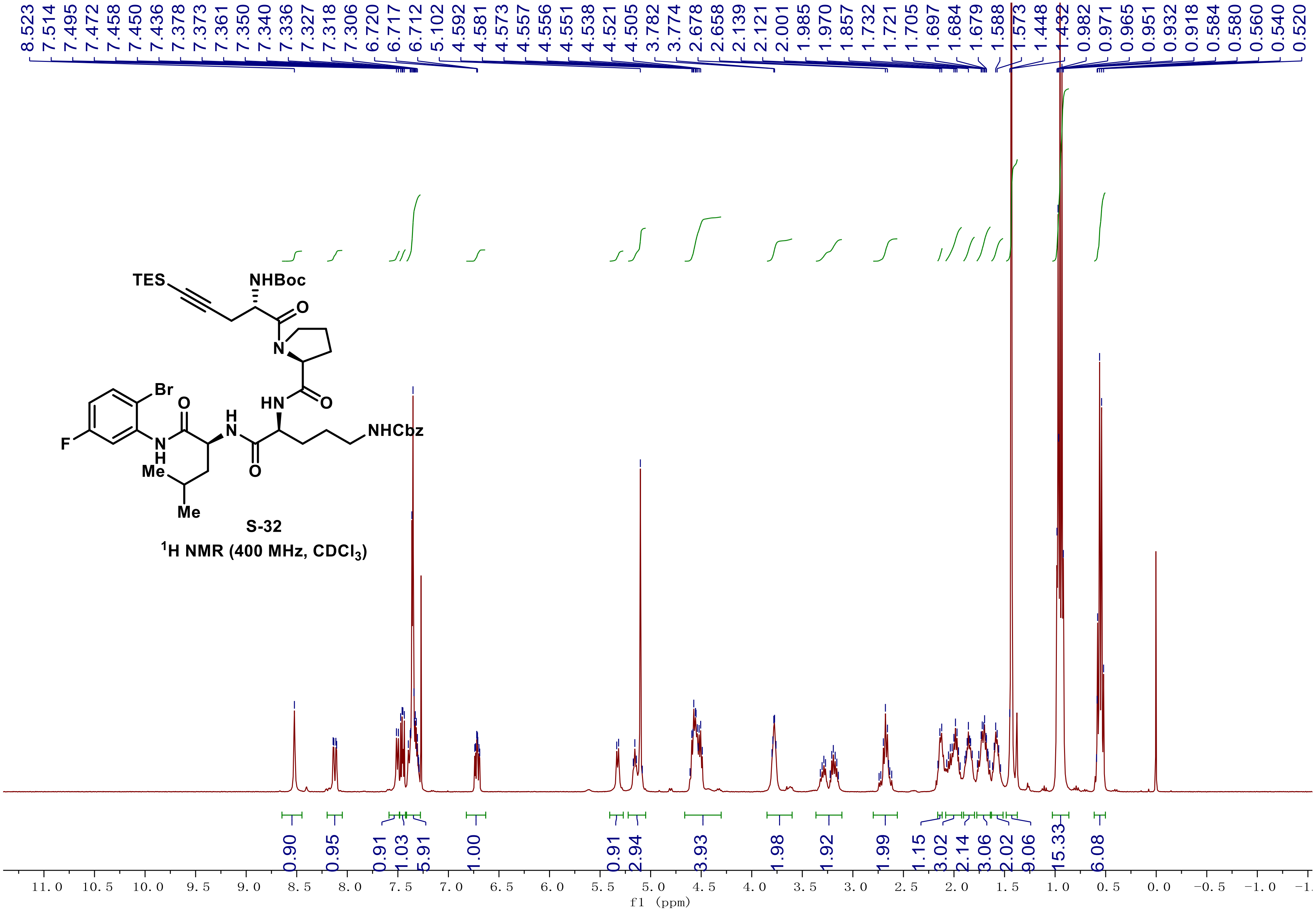
TES
NHBoc
Br
F
NHCbz
Me
Me
S-32
^{1}H NMR (400 MHz, CDCl$_3$)
8.523 7.514 7.495 7.472 7.458 7.450 7.436 7.378 7.373 7.361 7.350 7.340 7.336 7.327 7.318 7.306 6.720 6.717 6.712 5.102 4.592 4.581 4.573 4.557 4.556 4.551 4.538 4.521 4.505 3.782 3.774 2.678 2.658 2.139 2.121 2.001 1.985 1.970 1.857 1.732 1.721 1.705 1.697 1.684 1.679 1.588 1.573 1.448 1.432 0.982 0.971 0.965 0.951 0.932 0.918 0.584 0.580 0.560 0.540 0.520
0.90 0.95 0.91 1.03 5.91 1.00 0.91 2.94 3.93 1.98 1.92 1.99 1.15 3.02 2.14 3.06 2.02 9.06 15.33 6.08
f1 (ppm)

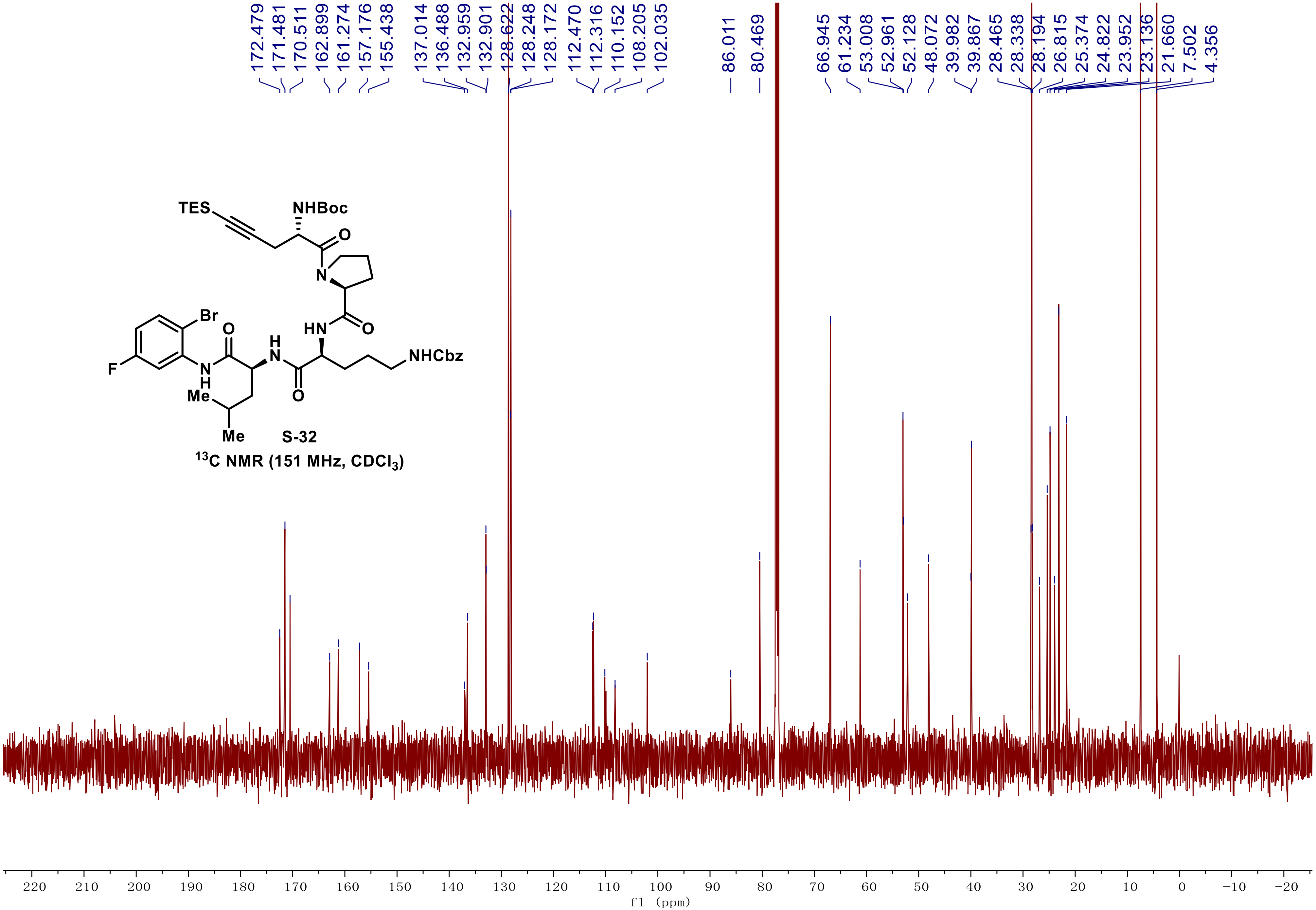
172.479
171.481
170.511
162.899
161.274
157.176
155.438
137.014
136.488
132.959
132.901
128.622
128.248
128.172
112.470
112.316
110.152
108.205
102.035
86.011
80.469
66.945
61.234
53.008
52.961
52.128
48.072
39.982
39.867
28.465
28.338
28.194
26.815
25.374
24.822
23.952
23.136
21.660
7.502
4.356
TES
NHBoc
O
N
Br
O
HN
O
F
N
H
H
N
NHCbz
Me
O
Me
S-32
^{13}C NMR (151 MHz, CDCl$_3$)
f1 (ppm)

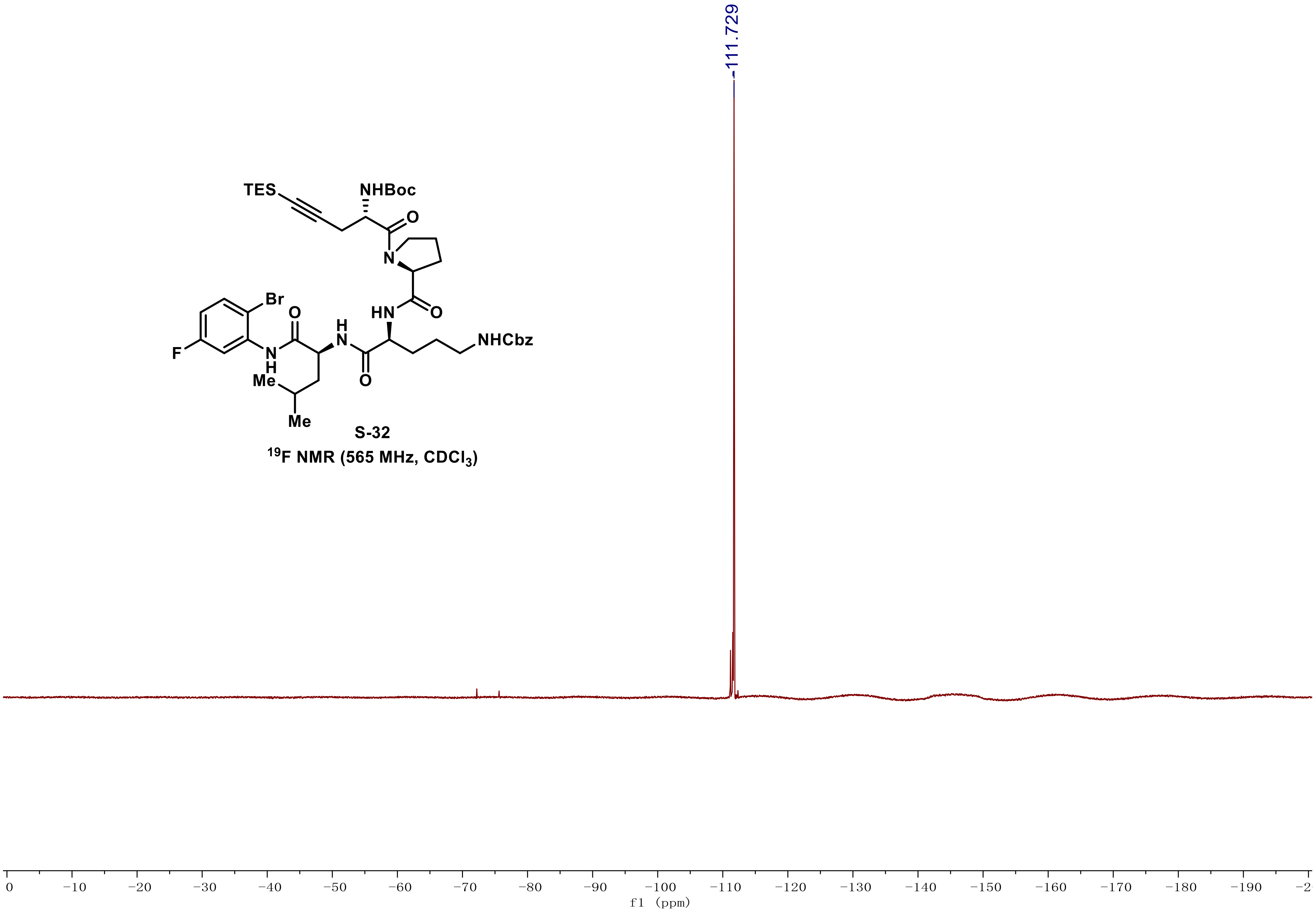

-111.729
TES
NHBoc
O
N
HN
O
Br
O
H
N
F
N
H
NHCbz
Me
Me
O
S-32
19F NMR (565 MHz, CDCl3)
0 −10 −20 −30 −40 −50 −60 −70 −80 −90 −100 −110 −120 −130 −140 −150 −160 −170 −180 −190
f1 (ppm)

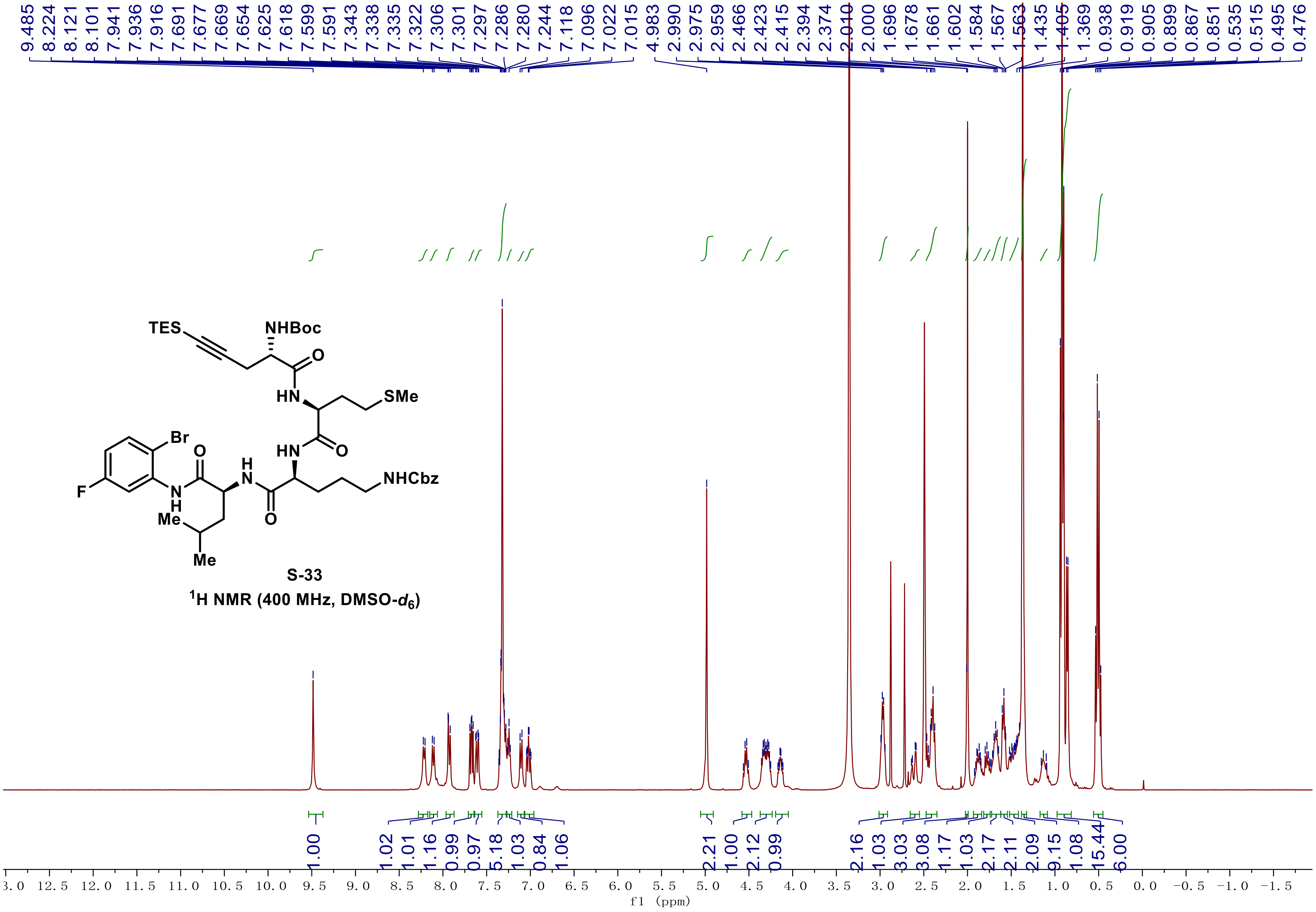
9.485
8.224
8.121
8.101
7.941
7.936
7.916
7.691
7.677
7.669
7.654
7.625
7.618
7.599
7.591
7.343
7.338
7.335
7.322
7.306
7.301
7.297
7.286
7.280
7.244
7.118
7.096
7.022
7.015
4.983
2.990
2.975
2.959
2.466
2.423
2.415
2.394
2.374
2.010
2.000
1.696
1.678
1.661
1.602
1.584
1.567
1.563
1.435
1.405
1.369
0.938
0.919
0.905
0.899
0.867
0.851
0.535
0.515
0.495
0.476
TES
NHBoc
O
HN
SMe
Br
O
H
N
HN
O
F
N
H
NHCbz
Me
O
Me
S-33
¹H NMR (400 MHz, DMSO-d6)
1.00
1.02
1.01
1.16
0.99
0.97
5.18
1.03
0.84
1.06
2.21
1.00
2.12
0.99
2.16
1.03
3.03
3.08
1.17
1.03
2.17
2.11
2.09
9.15
1.08
15.44
6.00
f1 (ppm)

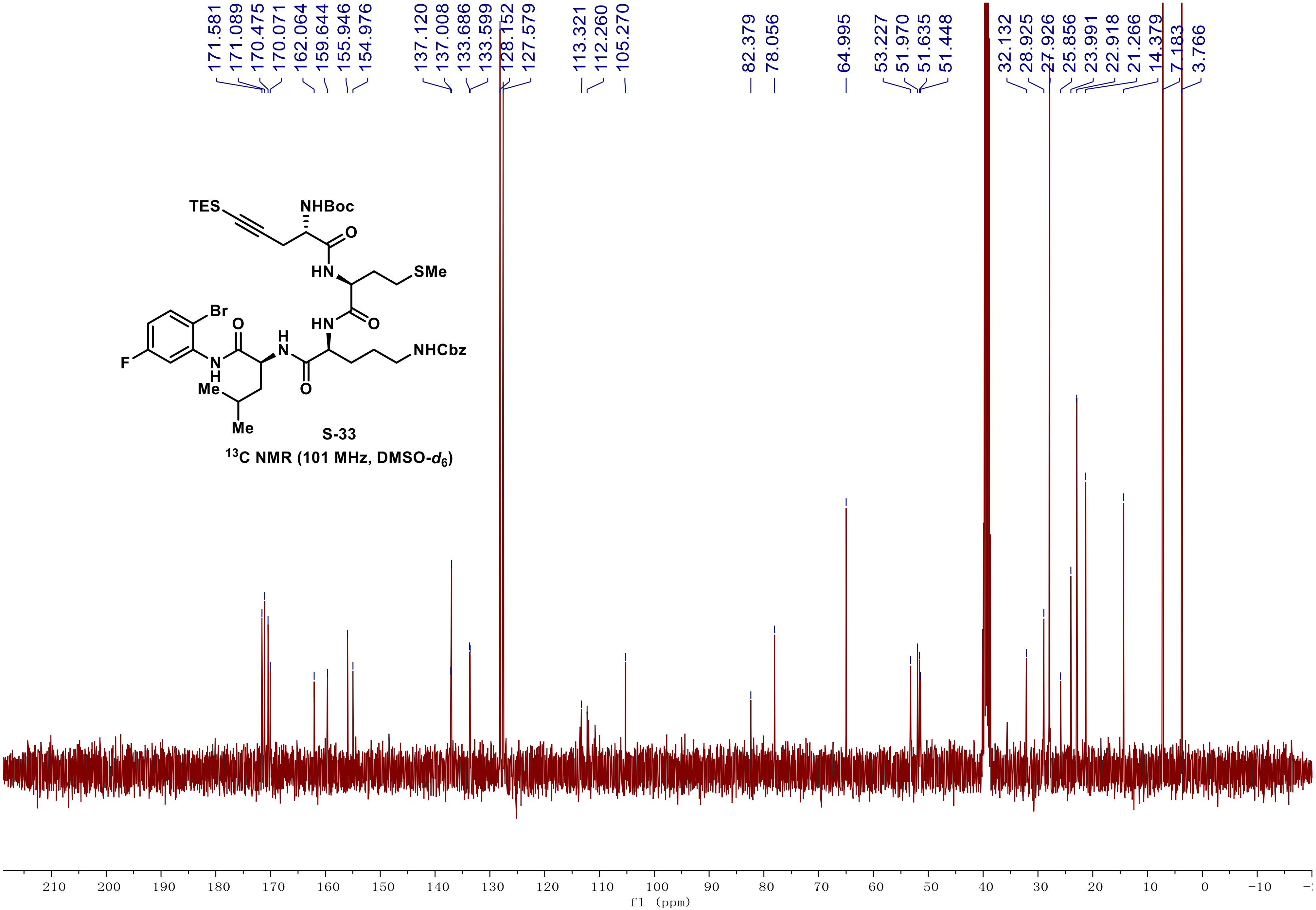

171.581
171.089
170.475
170.071
162.064
159.644
155.946
154.976
137.120
137.008
133.686
133.599
128.152
127.579
113.321
112.260
105.270
82.379
78.056
64.995
53.227
51.970
51.635
51.448
32.132
28.925
27.926
25.856
23.991
22.918
21.266
14.379
7.183
3.766
TES
NHBoc
O
HN
SMe
Br
O
H
N
HN
O
NHCbz
F
N
H
Me
O
Me
S-33
^{13}C NMR (101 MHz, DMSO-d_6)
210
200
190
180
170
160
150
140
130
120
110
100
90
80
70
60
50
40
30
20
10
0
-10
f1 (ppm)

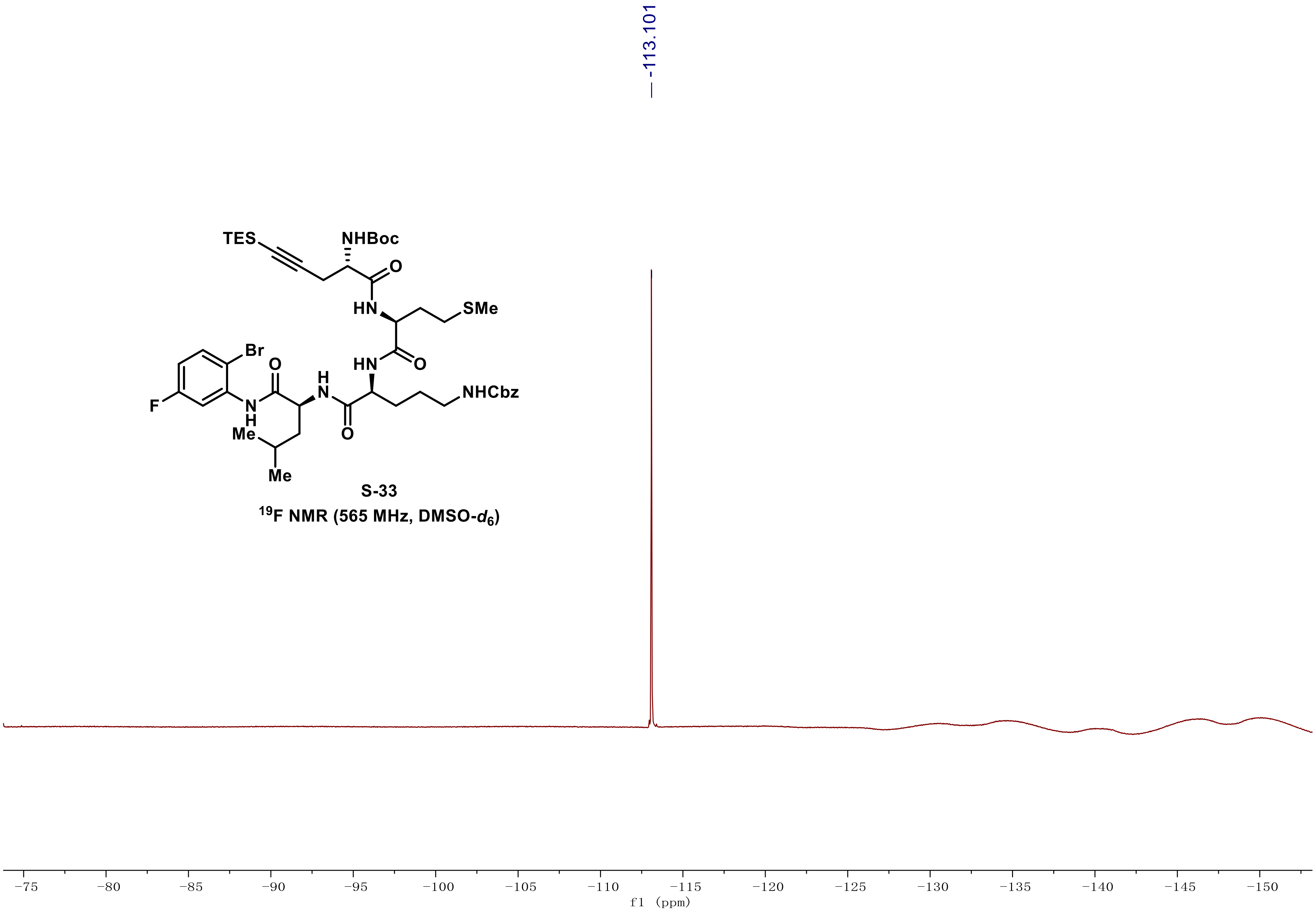
-113.101
TES
NHBoc
O
HN
SMe
Br
O
H
N
HN
O
NHCbz
F
N
H
Me
O
Me
S-33
^{19}F NMR (565 MHz, DMSO-d_6)
−75
−80
−85
−90
−95
−100
−105
−110
−115
−120
−125
−130
−135
−140
−145
−150
f1 (ppm)

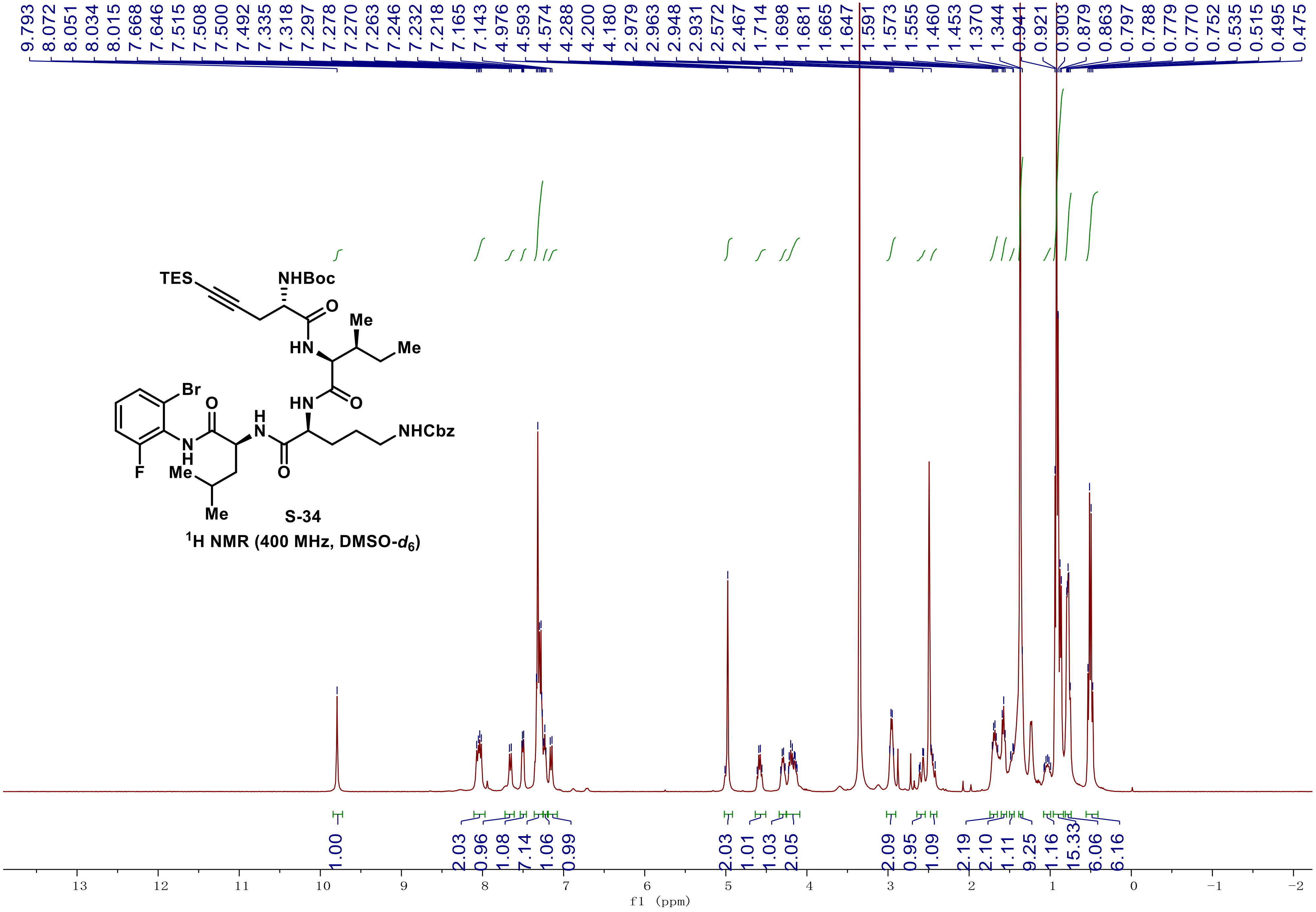

9.793 8.072 8.051 8.034 8.015 7.668 7.646 7.515 7.508 7.500 7.492 7.335 7.318 7.297 7.278 7.270 7.263 7.246 7.232 7.218 7.165 7.143 4.976 4.593 4.574 4.288 4.200 4.180 2.979 2.963 2.948 2.931 2.572 2.467 1.714 1.698 1.681 1.665 1.647 1.591 1.573 1.555 1.460 1.453 1.370 1.344 0.941 0.921 0.903 0.879 0.863 0.797 0.788 0.779 0.770 0.752 0.535 0.515 0.495 0.475
TES
NHBoc
O
Me
HN
Me
Br
O
HN
O
H
N
NHCbz
N
H
F
Me
O
Me
S-34
1H NMR (400 MHz, DMSO-d6)
1.00 2.03 0.96 1.08 7.14 1.06 0.99 2.03 1.01 1.03 2.05 2.09 0.95 1.09 2.19 2.10 1.11 9.25 1.16 15.33 6.06 6.16
13 12 11 10 9 8 7 6 5 4 3 2 1 0 −1 −2
f1 (ppm)

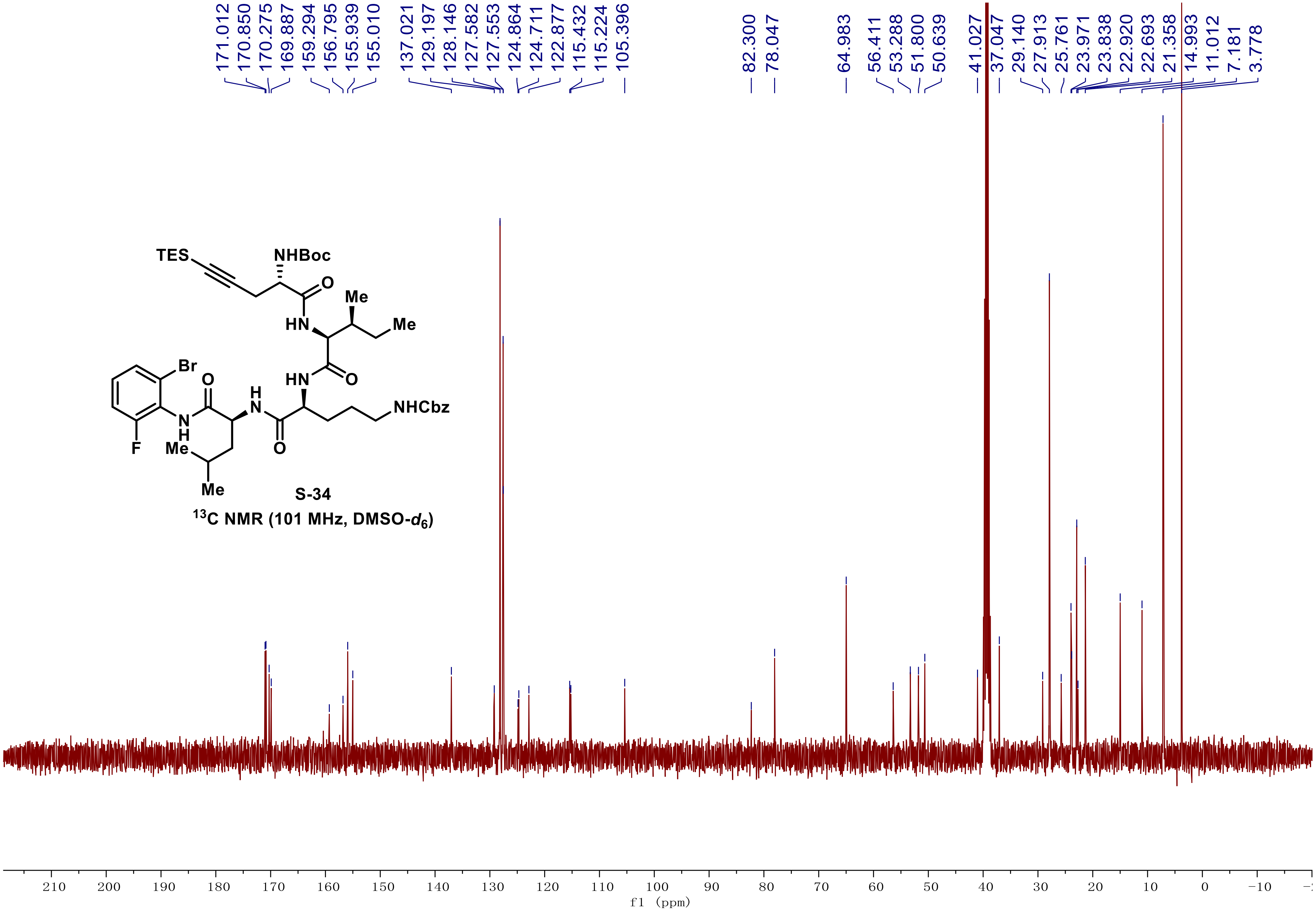
171.012
170.850
170.275
169.887
159.294
156.795
155.939
155.010
137.021
129.197
128.146
127.582
127.553
124.864
124.711
122.877
115.432
115.224
105.396
82.300
78.047
64.983
56.411
53.288
51.800
50.639
41.027
37.047
29.140
27.913
25.761
23.971
23.838
22.920
22.693
21.358
14.993
11.012
7.181
3.778
TES
NHBoc
O
Me
HN
Me
Br
O
H
N
HN
O
NHCbz
N
H
F
Me
O
Me
S-34
^{13}C NMR (101 MHz, DMSO-d_6)
210 200 190 180 170 160 150 140 130 120 110 100 90 80 70 60 50 40 30 20 10 0 −10
f1 (ppm)

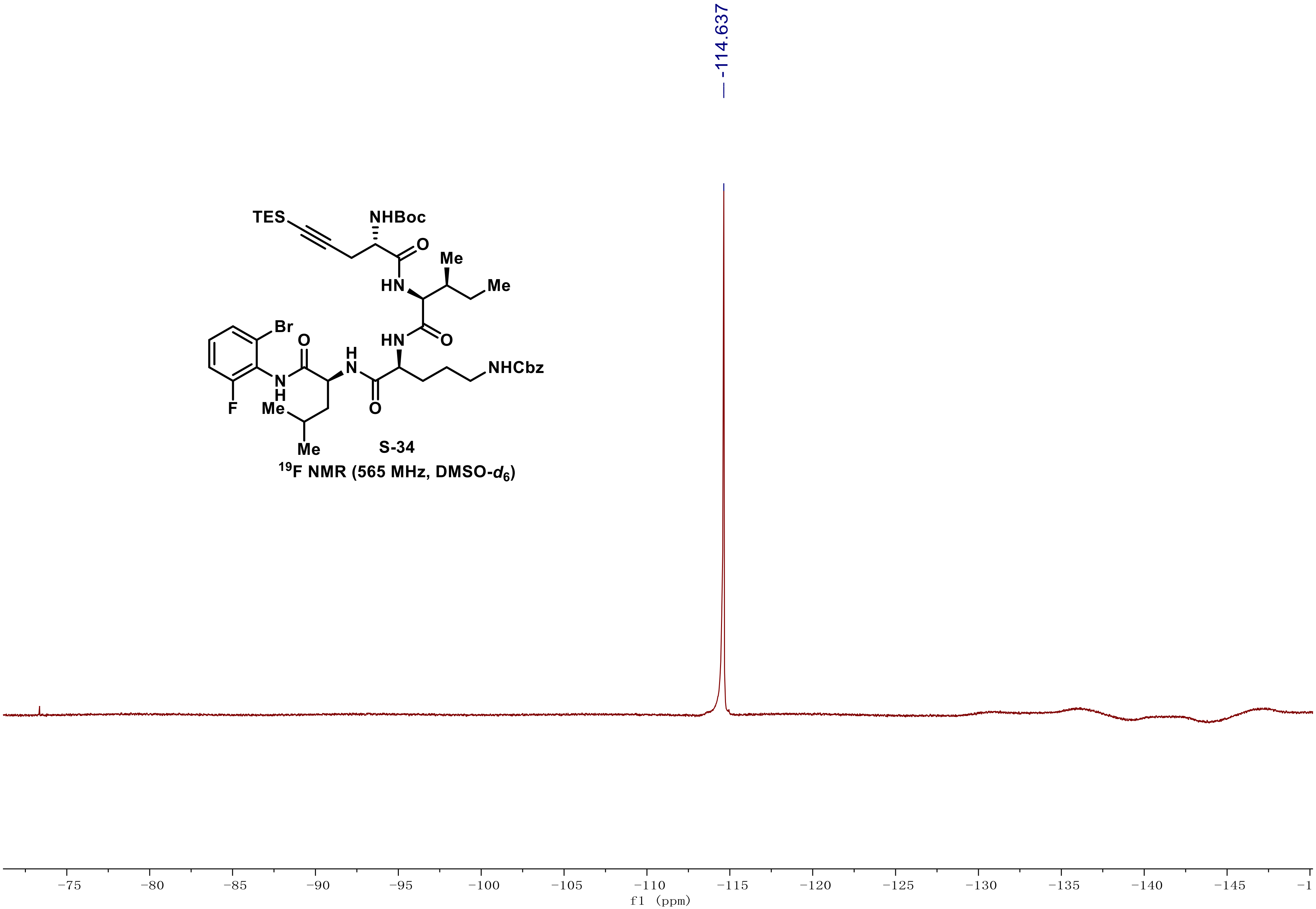

-114.637
TES
NHBoc
O
Me
HN
Me
Br
O
HN
O
H
N
NHCbz
N
H
F
Me
O
Me
S-34
19F NMR (565 MHz, DMSO-d6)
−75
−80
−85
−90
−95
−100
−105
−110
−115
−120
−125
−130
−135
−140
−145
f1 (ppm)

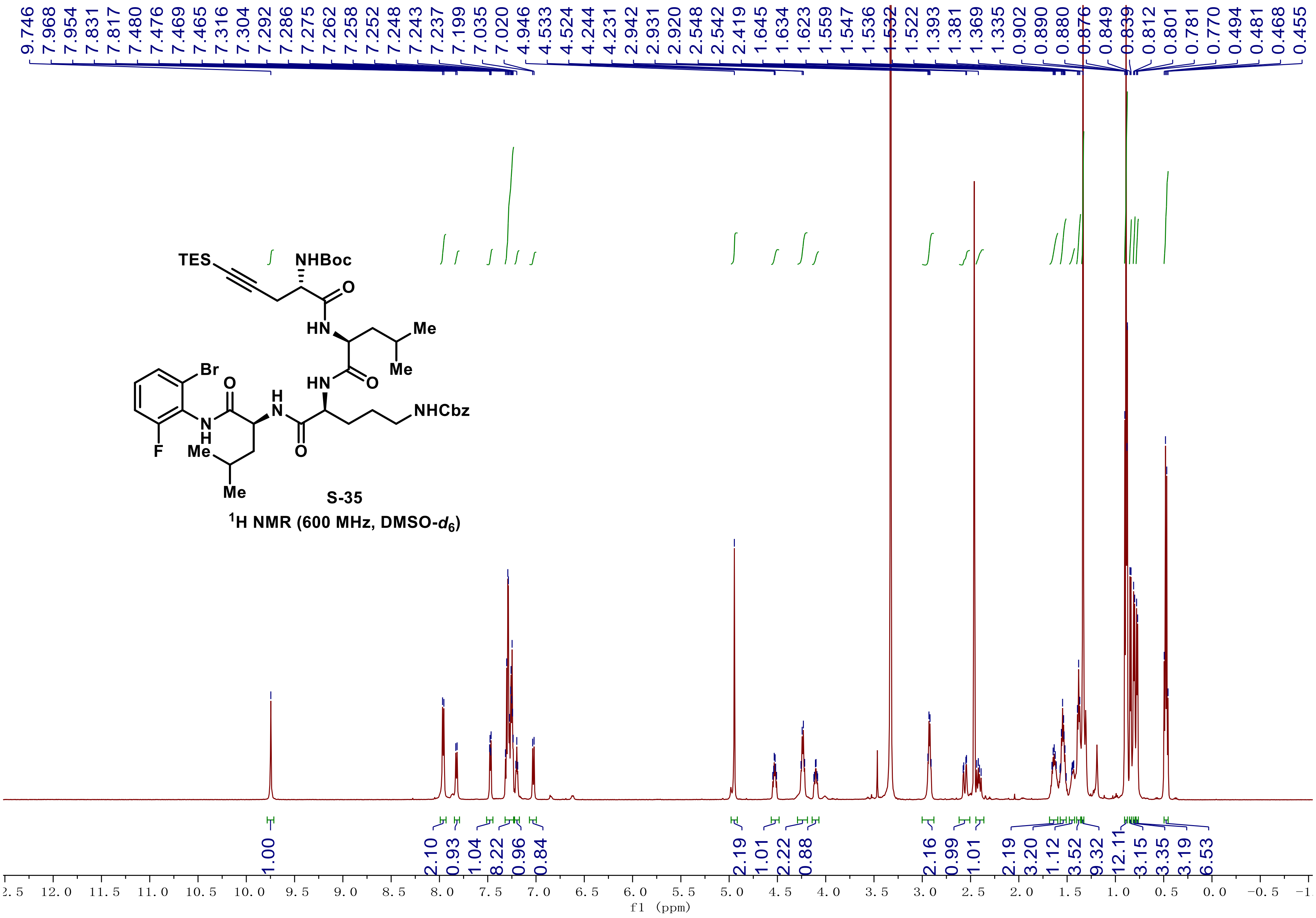
TES
NHBoc
O
HN
Me
Me
HN
O
Br
O
H
N
NHCbz
N
H
F
Me
O
Me
S-35
1H NMR (600 MHz, DMSO-d6)
f1 (ppm)

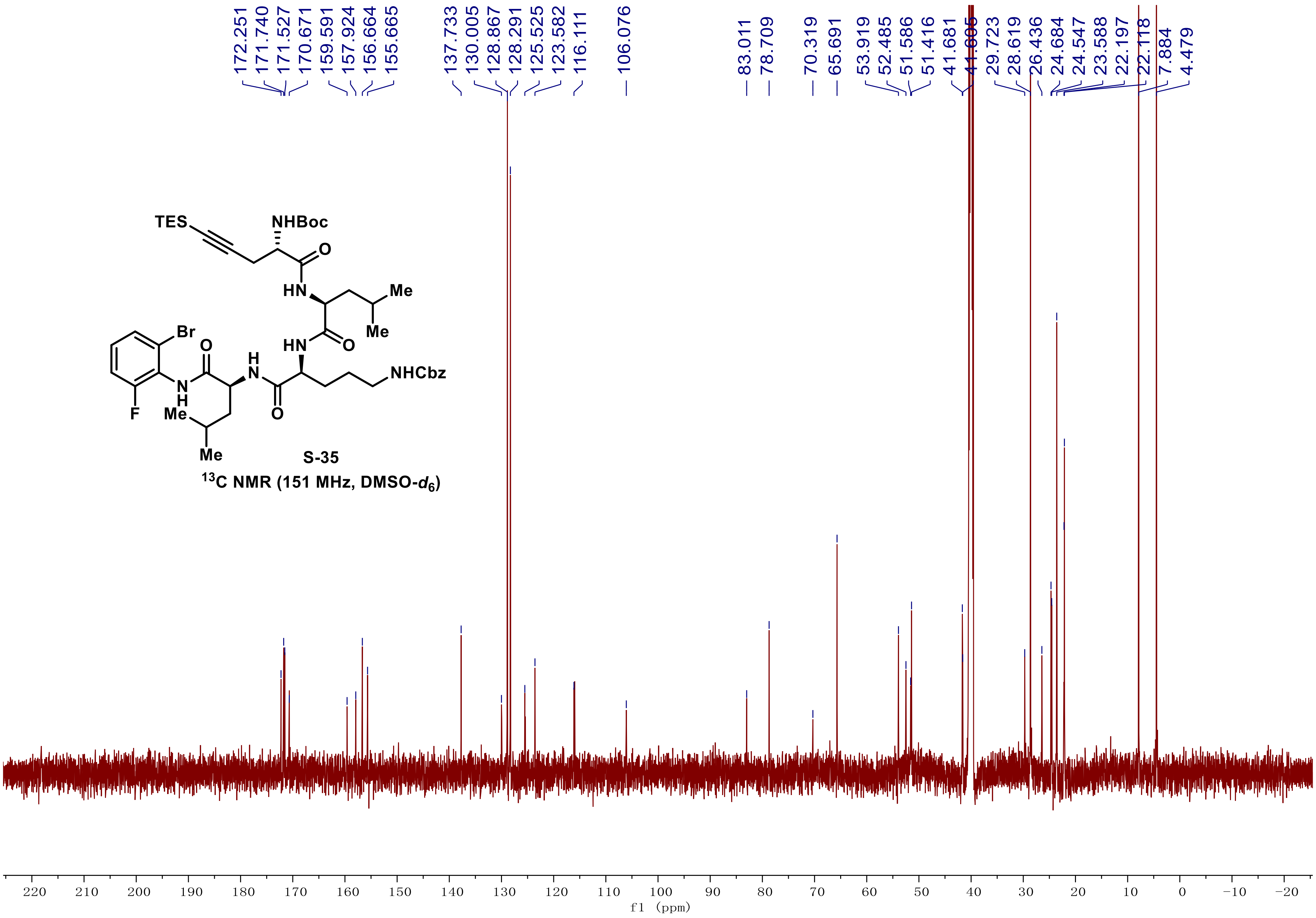

^{13}C NMR (151 MHz, DMSO-d_6)

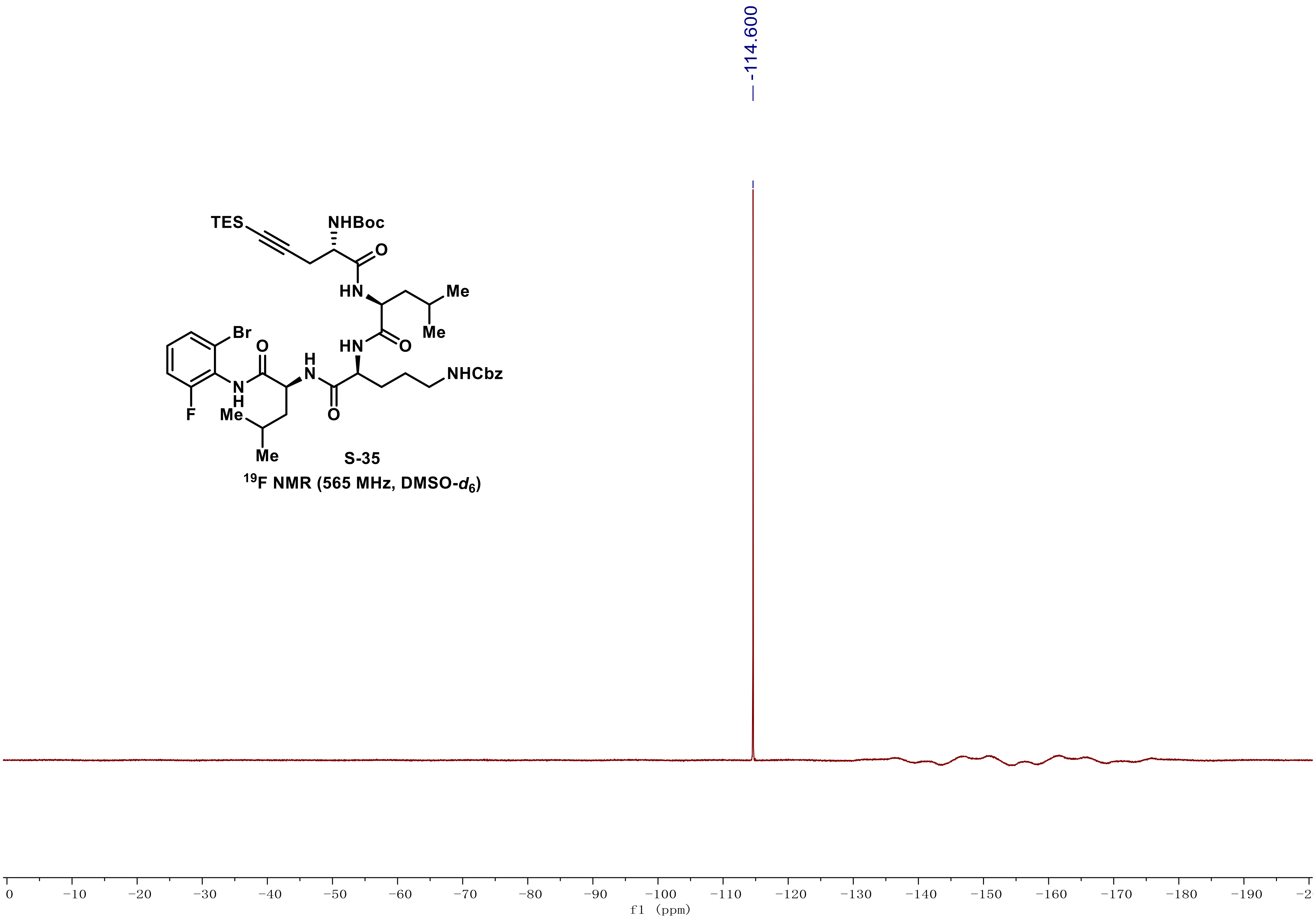
-114.600
TES
NHBoc
O
HN
Me
Me
Br
O
H
N
HN
O
NHCbz
N
H
F
Me
O
Me
S-35
19F NMR (565 MHz, DMSO-d6)
0 −10 −20 −30 −40 −50 −60 −70 −80 −90 −100 −110 −120 −130 −140 −150 −160 −170 −180 −190 −2
f1 (ppm)

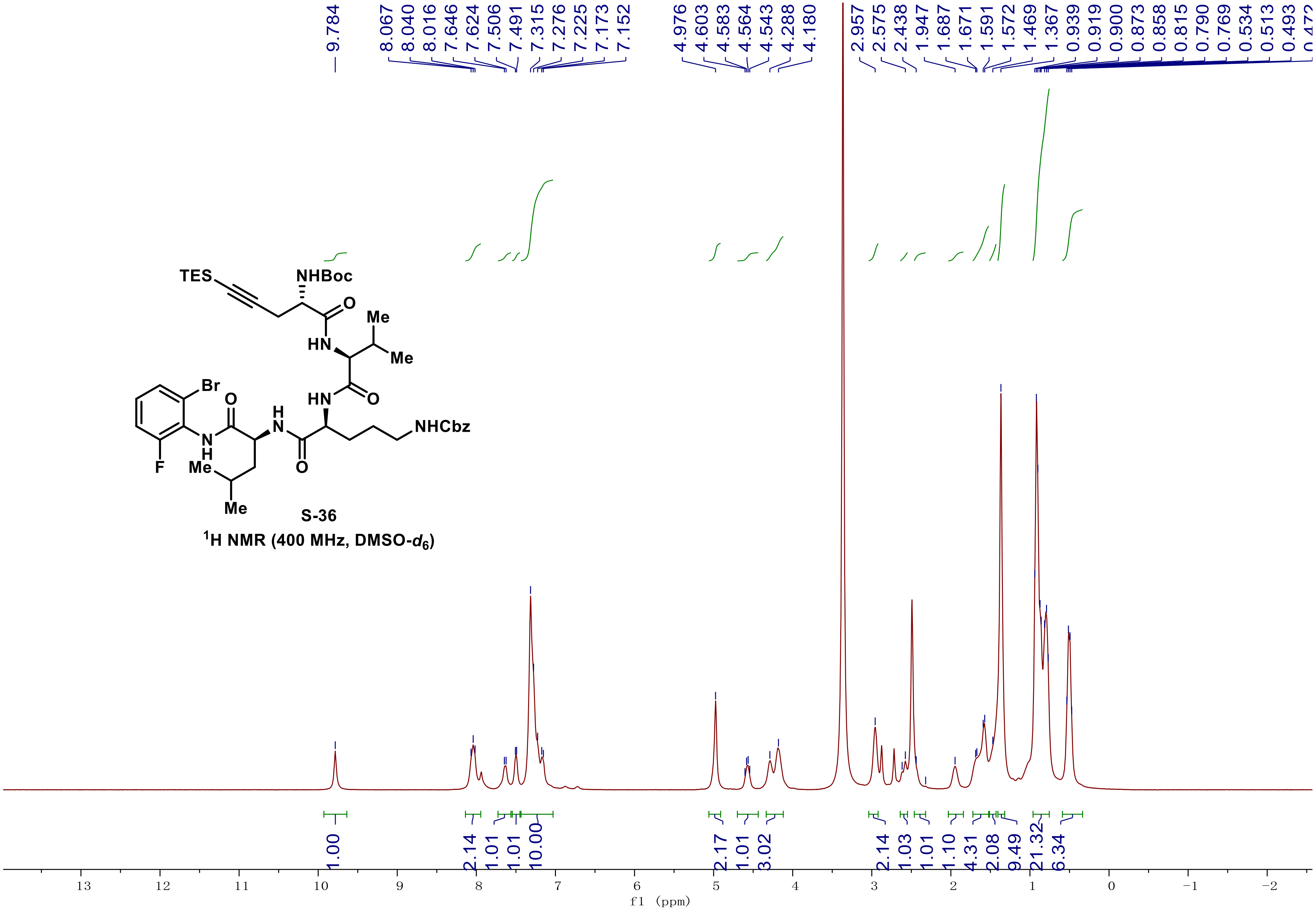

S-36
1H NMR (400 MHz, DMSO-d6)
f1 (ppm)

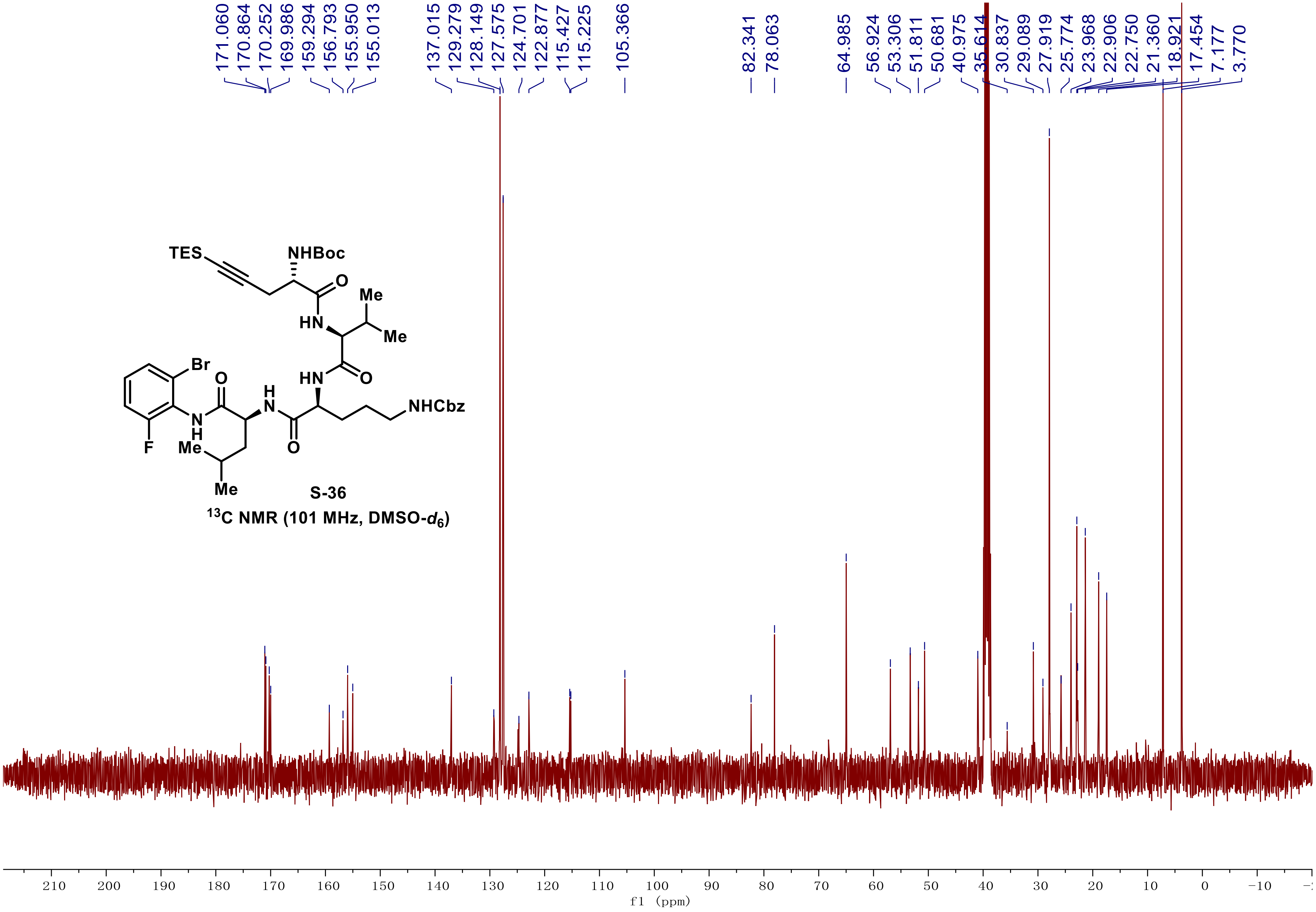

S-36

^{13}C NMR (101 MHz, DMSO-d_6)

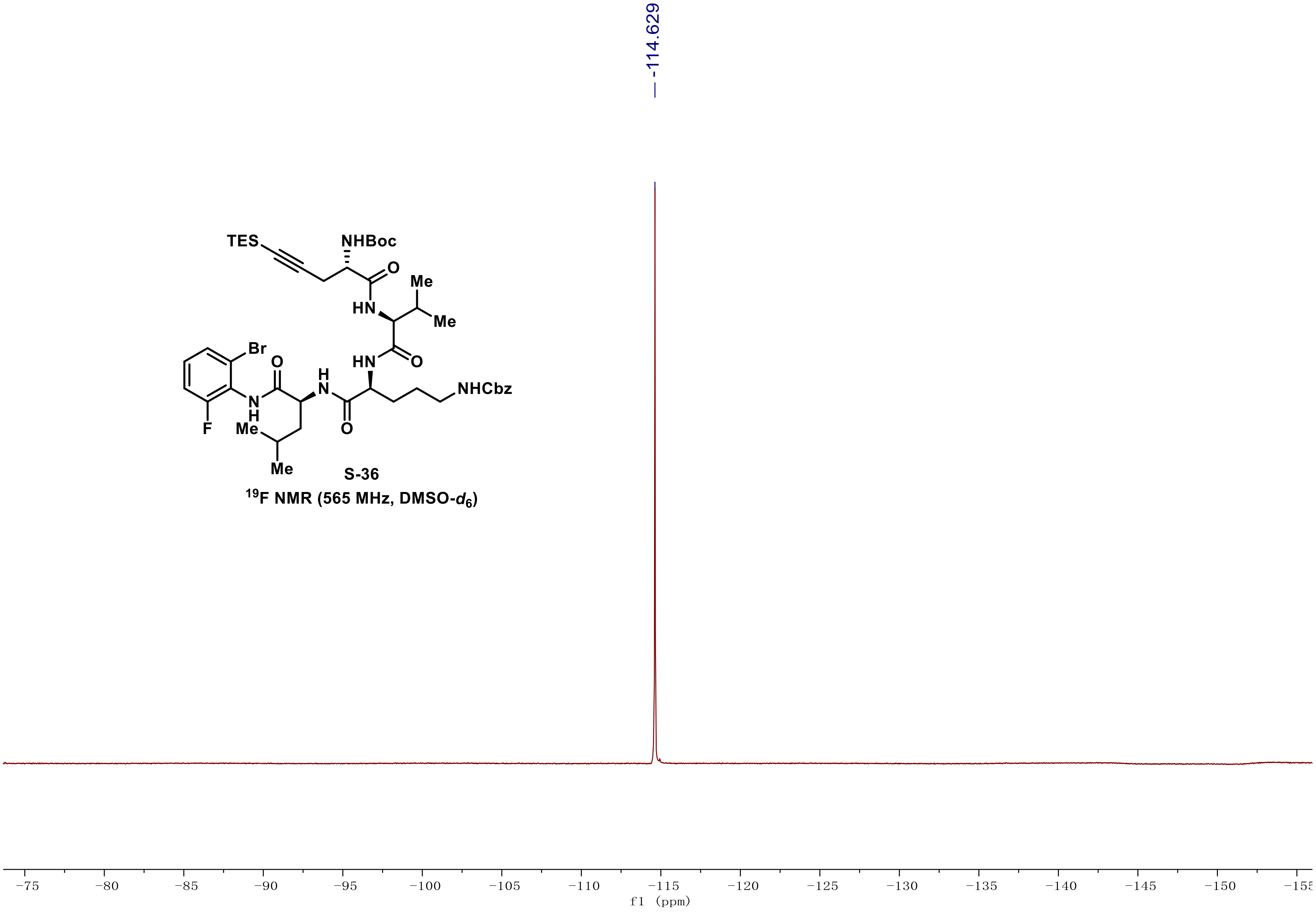

-114.629
TES
NHBoc
O
Me
HN
Me
Br
O
H
N
HN
O
NHCbz
N
H
F
Me
O
Me
S-36
^{19}F NMR (565 MHz, DMSO-d_6)
−75
−80
−85
−90
−95
−100
−105
−110
−115
−120
−125
−130
−135
−140
−145
−150
f1 (ppm)

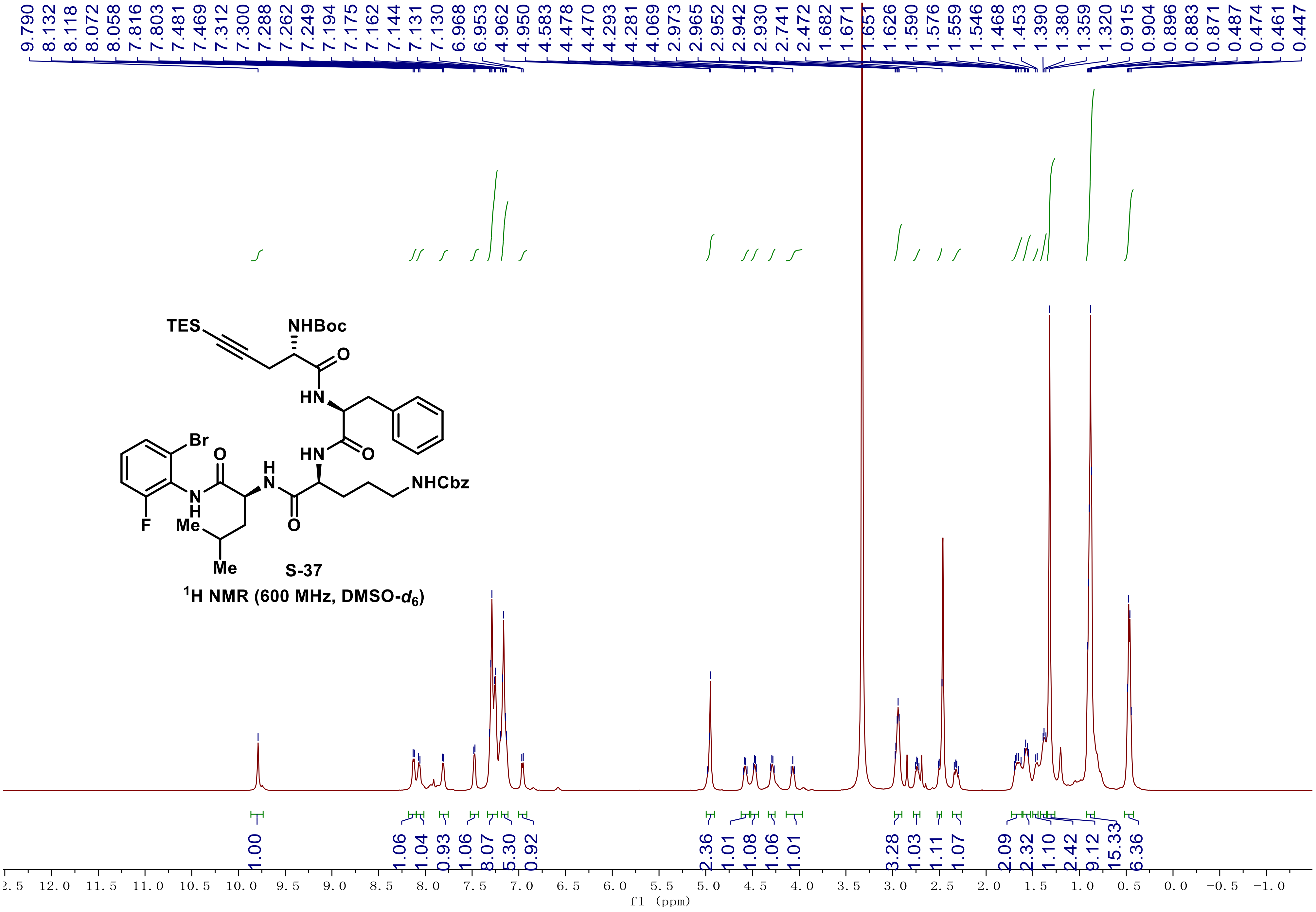
9.790
8.132
8.118
8.072
8.058
7.816
7.803
7.481
7.469
7.312
7.300
7.288
7.262
7.249
7.194
7.175
7.162
7.144
7.131
7.130
6.968
6.953
4.962
4.950
4.583
4.478
4.470
4.293
4.281
4.069
2.973
2.965
2.952
2.942
2.930
2.741
2.472
1.682
1.671
1.651
1.626
1.590
1.576
1.559
1.546
1.468
1.453
1.390
1.380
1.359
1.320
0.915
0.904
0.896
0.883
0.871
0.487
0.474
0.461
0.447
TES
NHBoc
O
HN
Br
O
HN
O
H
N
NHCbz
N
H
F
Me
O
Me
S-37
¹H NMR (600 MHz, DMSO-d6)
1.00
1.06
1.04
0.93
1.06
8.07
5.30
0.92
2.36
1.01
1.08
1.06
1.01
3.28
1.03
1.11
1.07
2.09
2.32
1.10
2.42
9.12
15.33
6.36
f1 (ppm)

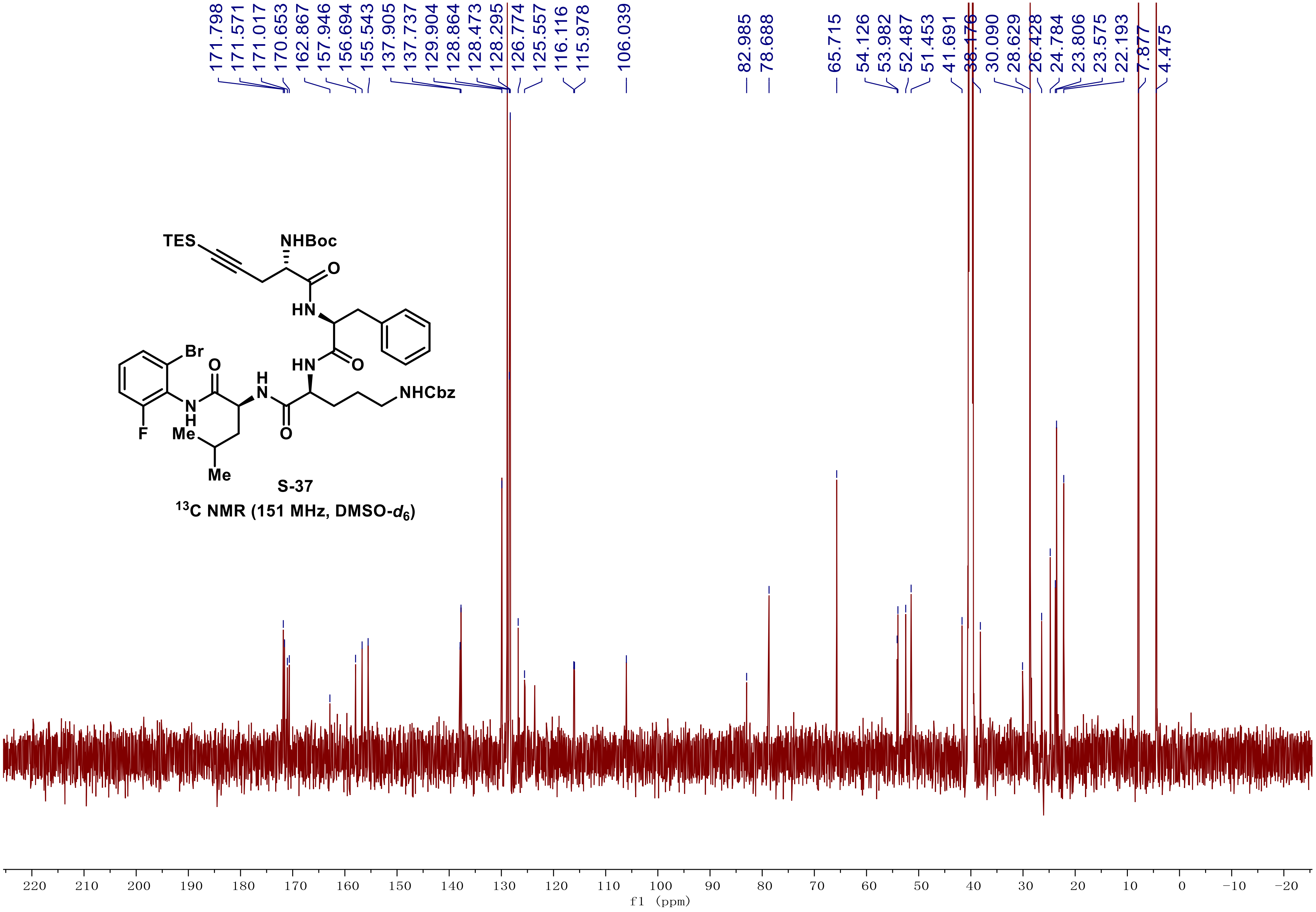
171.798
171.571
171.017
170.653
162.867
157.946
156.694
155.543
137.905
137.737
129.904
128.864
128.473
128.295
126.774
125.557
116.116
115.978
106.039
82.985
78.688
65.715
54.126
53.982
52.487
51.453
41.691
38.176
30.090
28.629
26.428
24.784
23.806
23.575
22.193
7.877
4.475
TES
NHBoc
O
HN
HN
O
Br
O
H
N
NHCbz
N
H
F
Me
O
Me
S-37
^{13}C NMR (151 MHz, DMSO-d_6)
220 210 200 190 180 170 160 150 140 130 120 110 100 90 80 70 60 50 40 30 20 10 0 −10 −20
f1 (ppm)

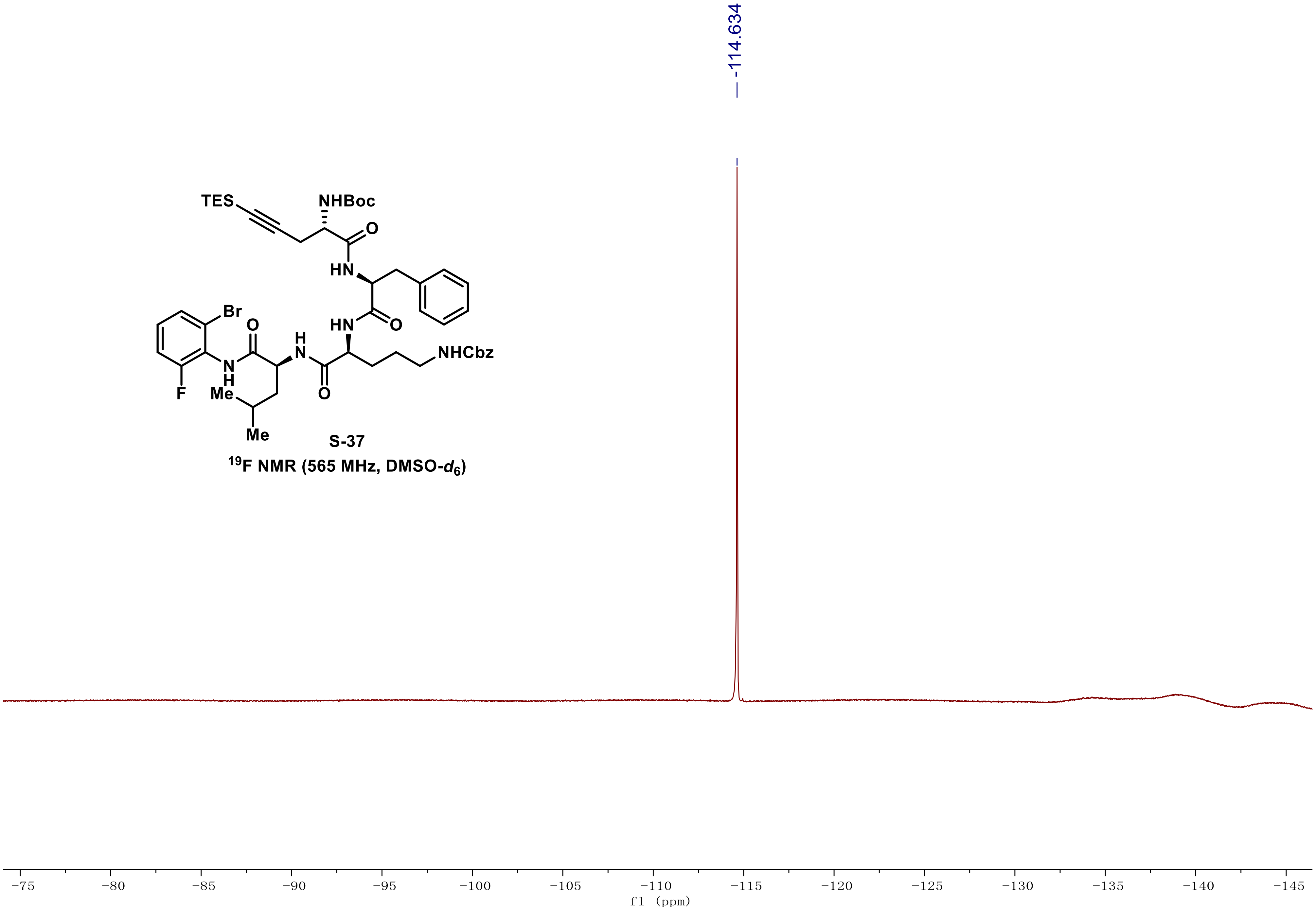

S-37

^{19}F NMR (565 MHz, DMSO-d_6)

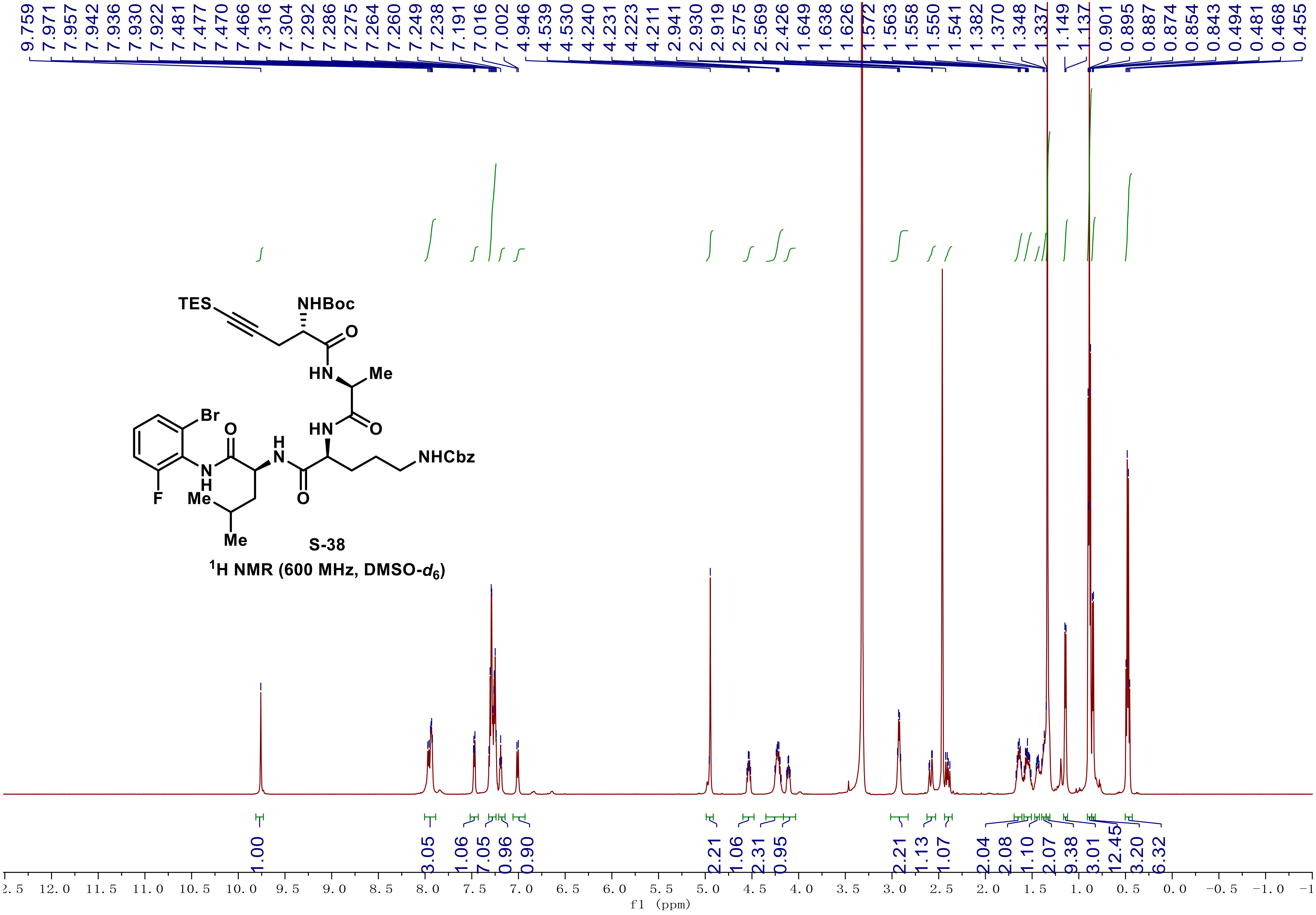
9.759 7.971 7.957 7.942 7.936 7.930 7.922 7.481 7.477 7.470 7.466 7.316 7.304 7.292 7.286 7.275 7.264 7.260 7.249 7.238 7.191 7.016 7.002 4.946 4.539 4.530 4.240 4.231 4.223 4.211 2.941 2.930 2.919 2.575 2.569 2.426 1.649 1.638 1.626 1.572 1.563 1.558 1.550 1.541 1.382 1.370 1.348 1.337 1.149 1.137 0.901 0.895 0.887 0.874 0.854 0.843 0.494 0.481 0.468 0.455
TES
NHBoc
O
HN
Me
Br
O
H
N
HN
O
NHCbz
N
H
F
Me
O
Me
S-38
1H NMR (600 MHz, DMSO-d6)
1.00 3.05 1.06 7.05 0.96 0.90 2.21 1.06 2.31 0.95 2.21 1.13 1.07 2.04 2.08 1.10 2.07 9.38 3.01 12.45 3.20 6.32
12.5 12.0 11.5 11.0 10.5 10.0 9.5 9.0 8.5 8.0 7.5 7.0 6.5 6.0 5.5 5.0 4.5 4.0 3.5 3.0 2.5 2.0 1.5 1.0 0.5 0.0 −0.5 −1.0 −1
f1 (ppm)

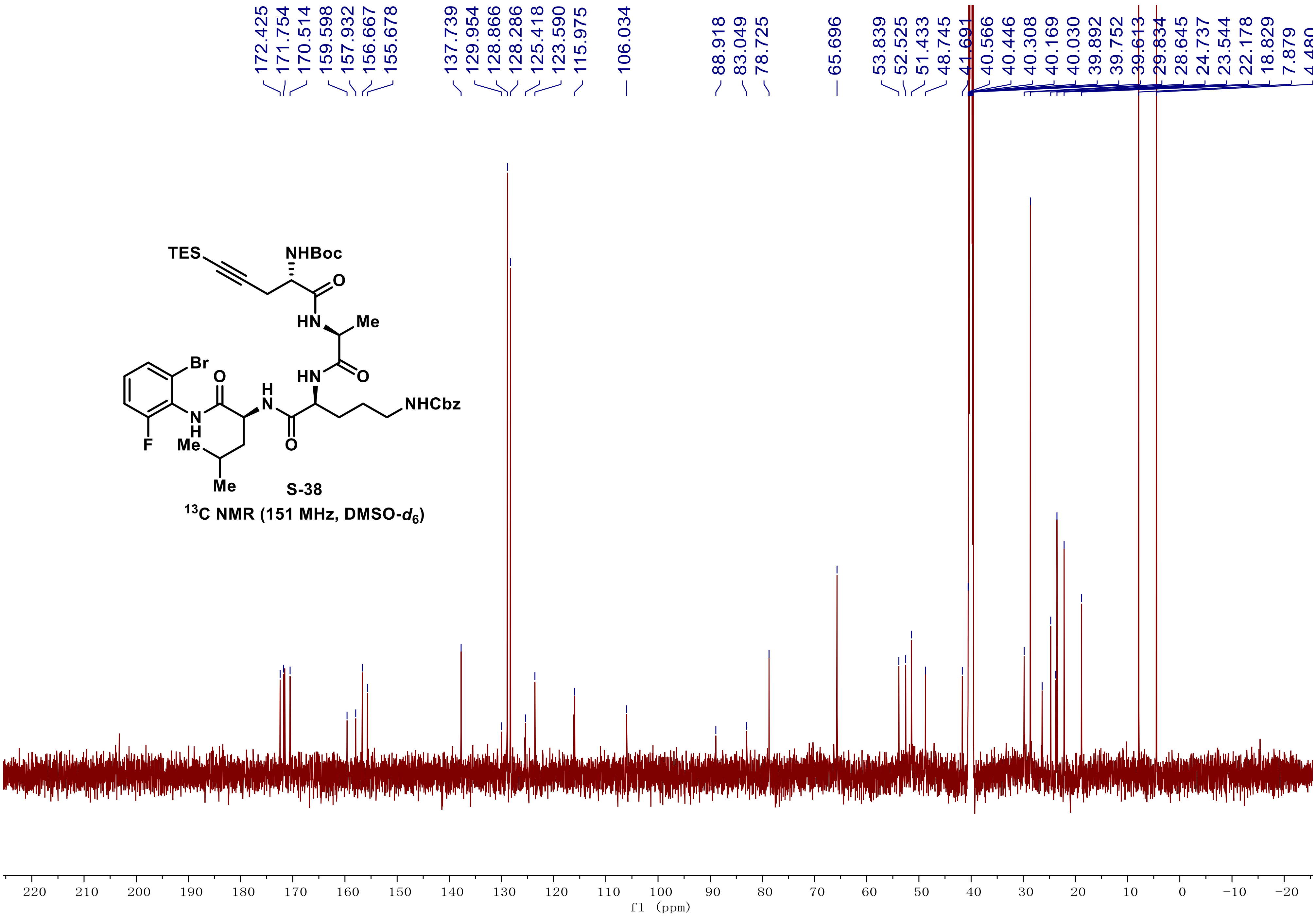

S-38

^{13}C NMR (151 MHz, DMSO-d_6)

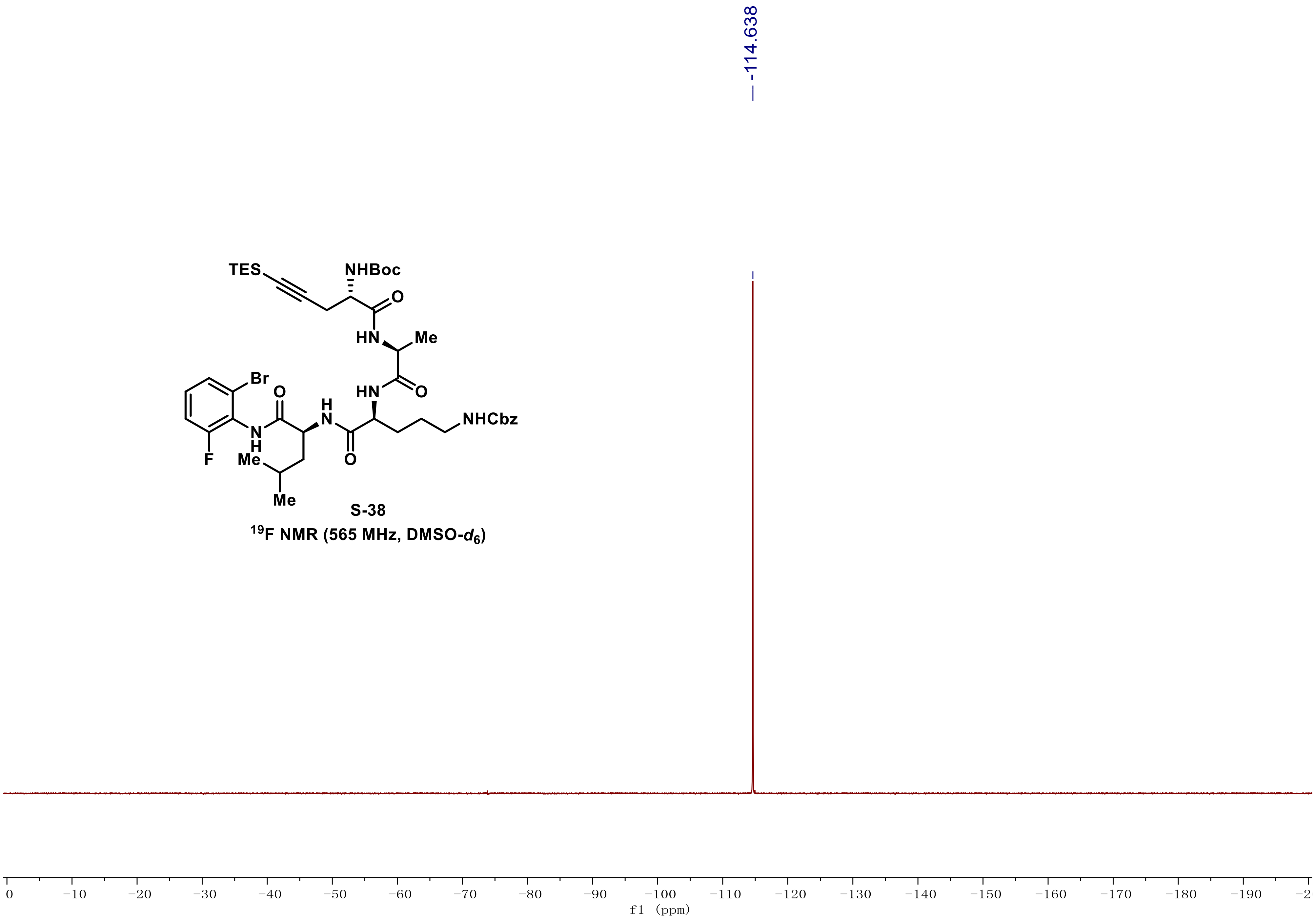

S-38

^{19}F NMR (565 MHz, DMSO-d_6)

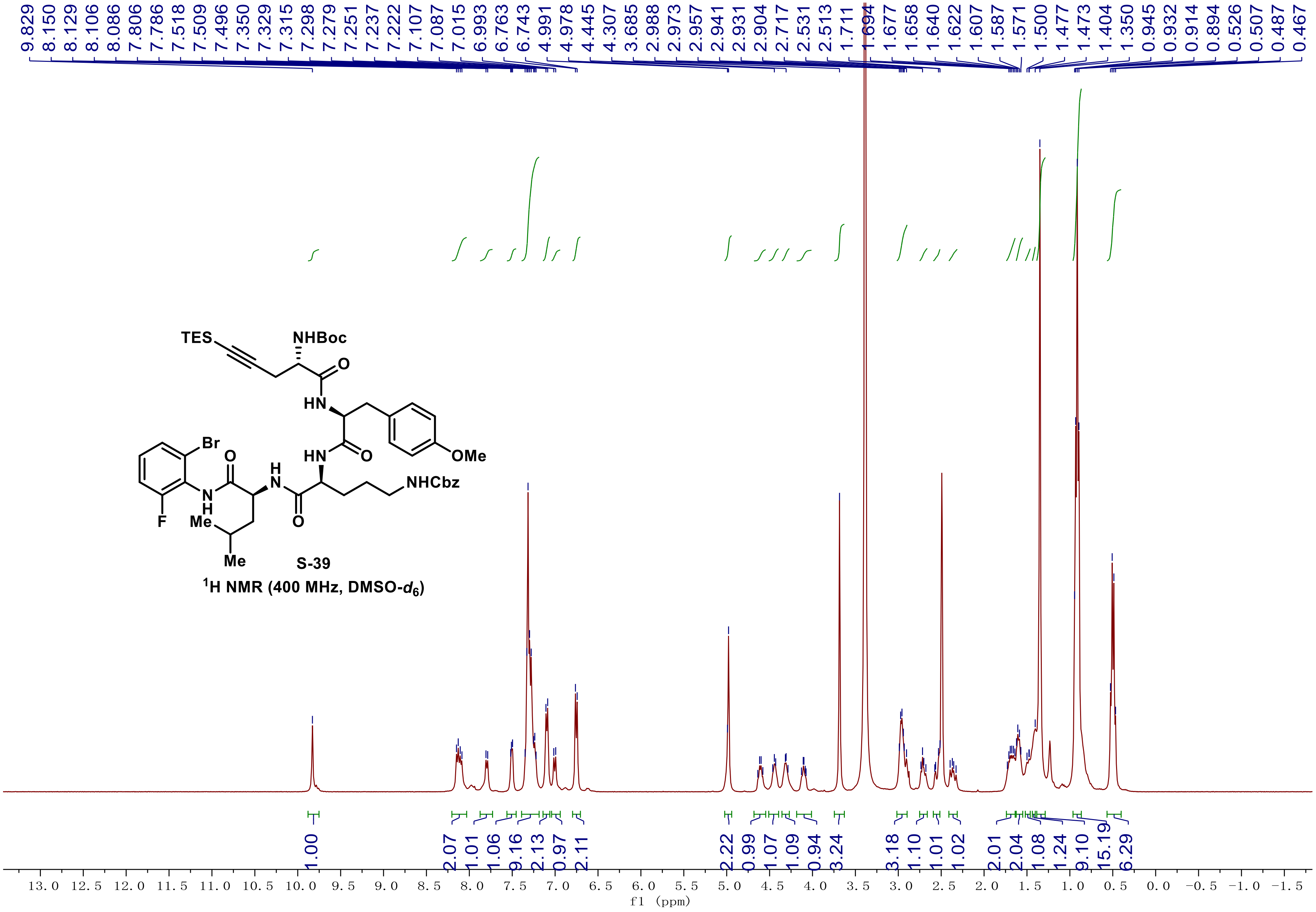

S-39

^{1}H NMR (400 MHz, DMSO-d_6)

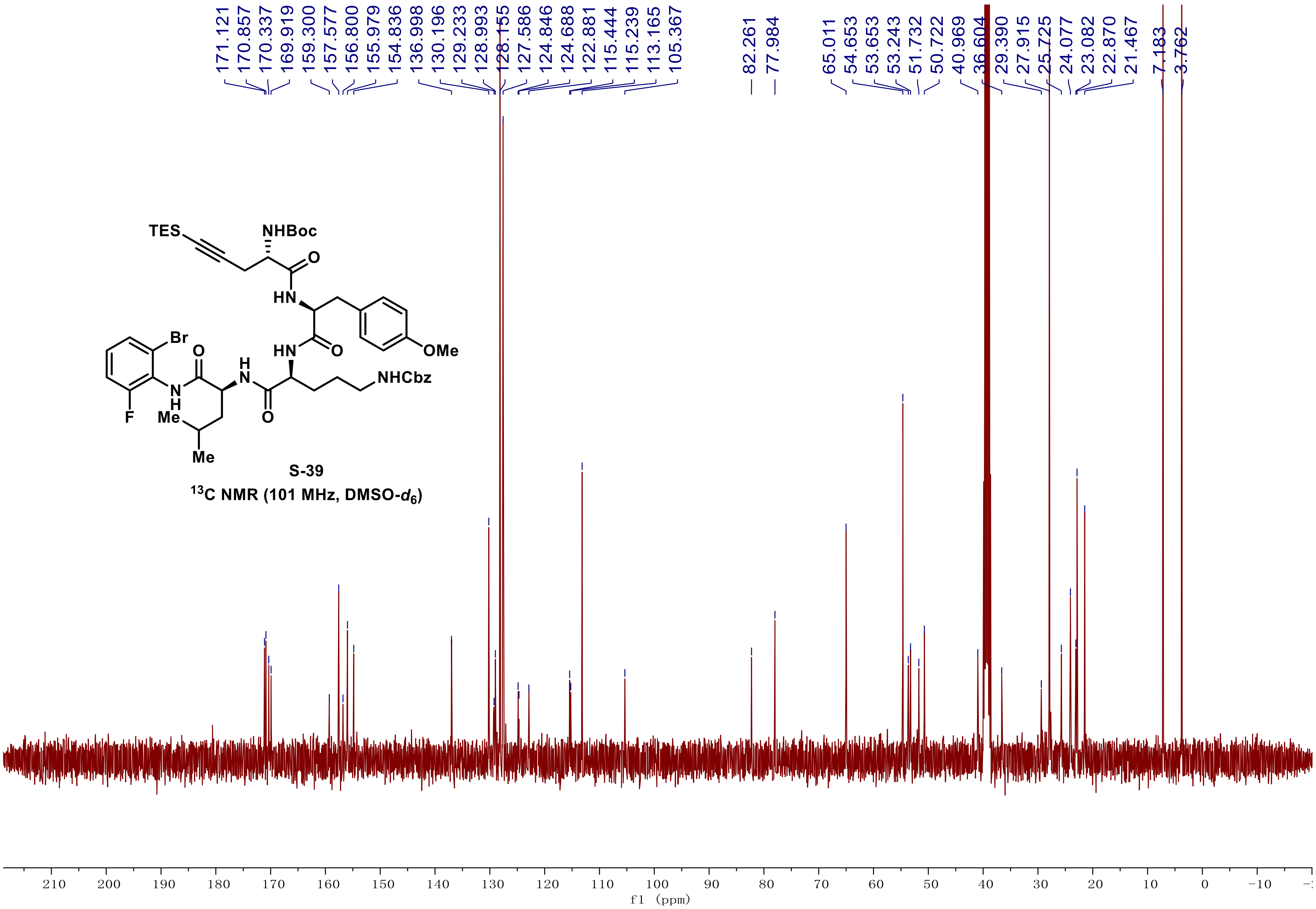

S-39

^{13}C NMR (101 MHz, DMSO-d_6)

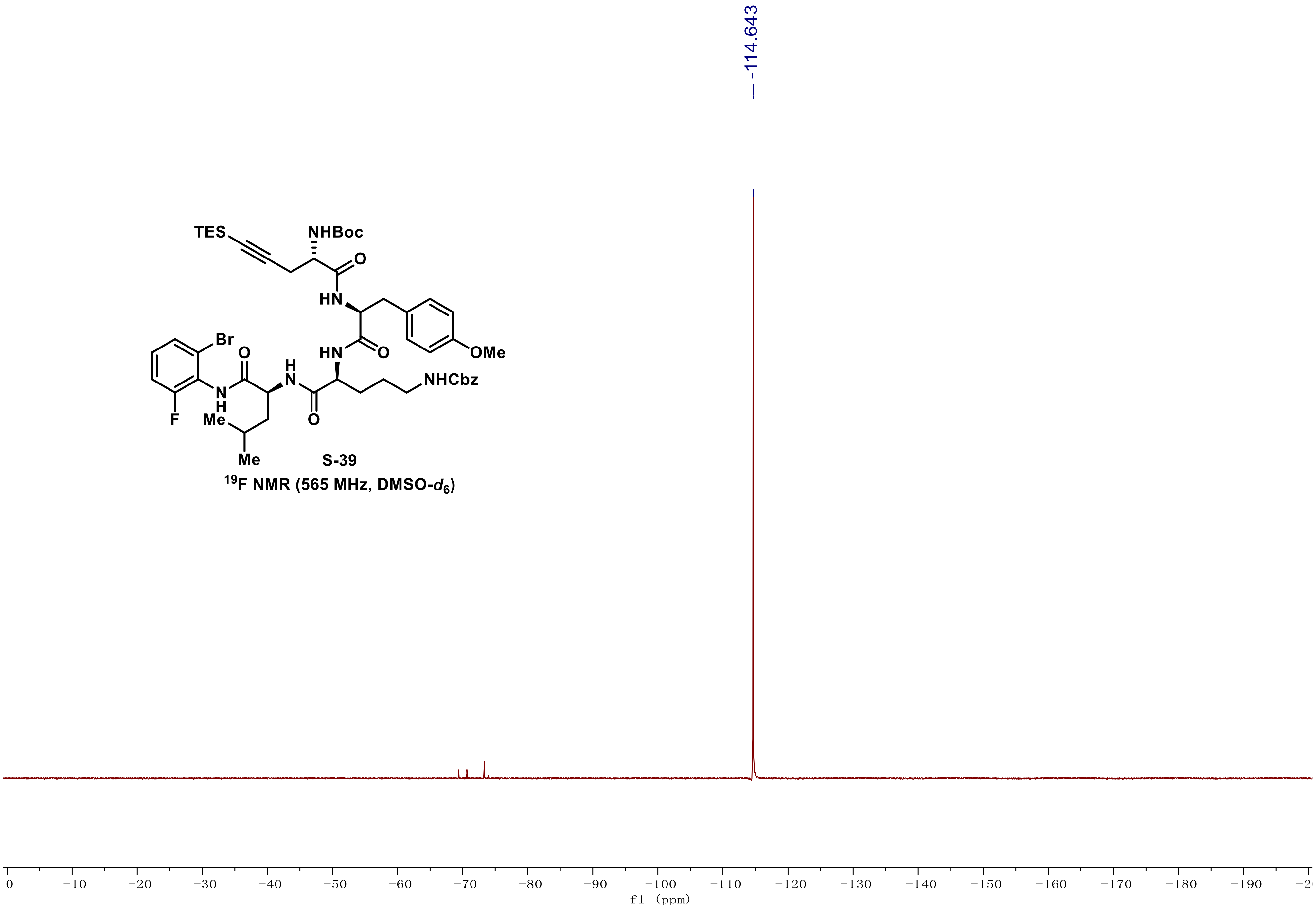
-114.643
TES
NHBoc
O
HN
OMe
Br
O
H
N
HN
O
NHCbz
N
H
F
Me
O
Me
S-39
^{19}F NMR (565 MHz, DMSO-d_6)
0 −10 −20 −30 −40 −50 −60 −70 −80 −90 −100 −110 −120 −130 −140 −150 −160 −170 −180 −190
f1 (ppm)

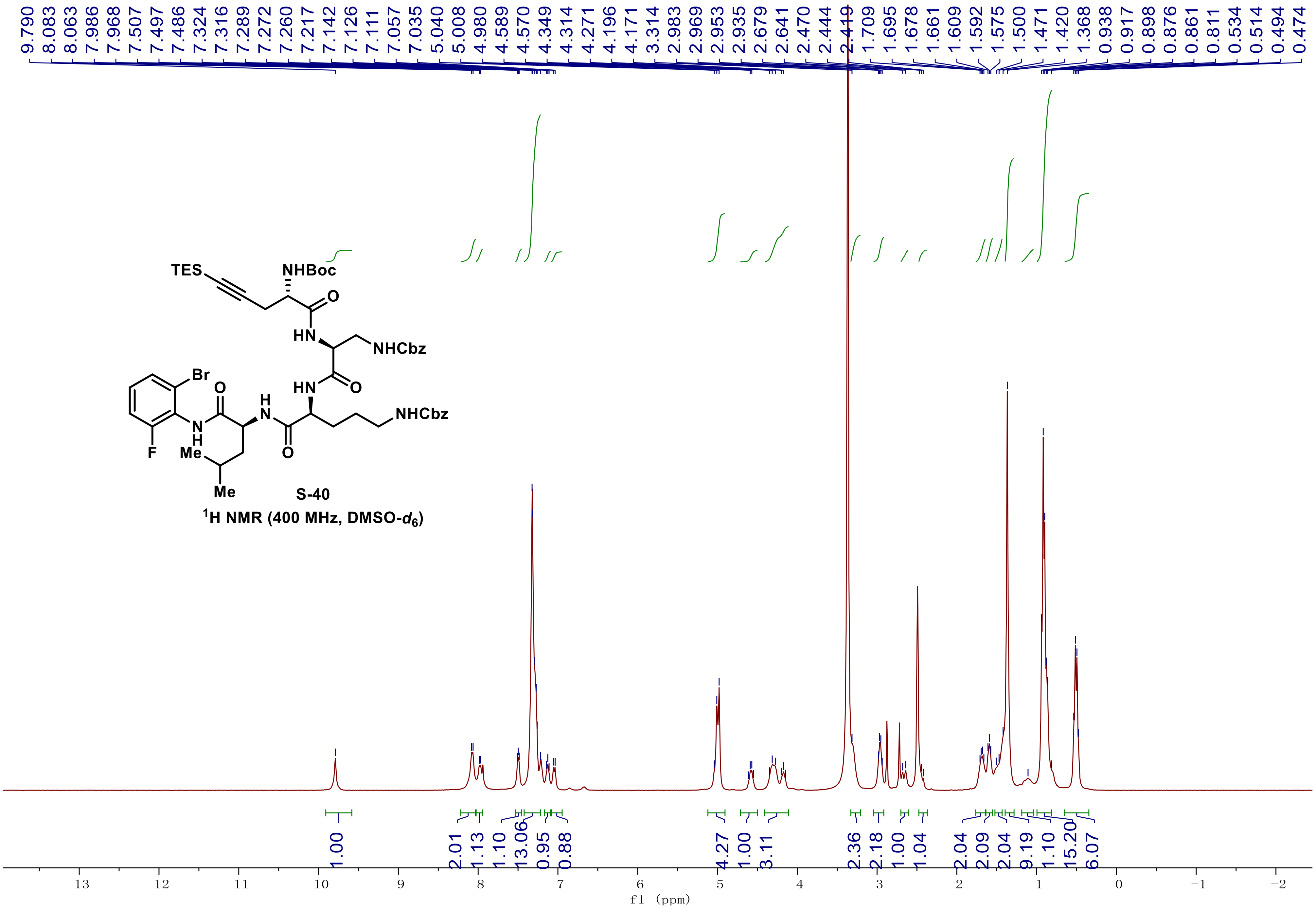
9.790 8.083 8.063 7.986 7.968 7.507 7.497 7.486 7.324 7.316 7.289 7.272 7.260 7.217 7.142 7.126 7.111 7.057 7.035 5.040 5.008 4.980 4.589 4.570 4.349 4.314 4.271 4.196 4.171 3.314 2.983 2.969 2.953 2.935 2.679 2.641 2.470 2.444 2.419 1.709 1.695 1.678 1.661 1.609 1.592 1.575 1.500 1.471 1.420 1.368 0.938 0.917 0.898 0.876 0.861 0.811 0.534 0.514 0.494 0.474
TES
NHBoc
O
HN
NHCbz
Br
O
H
N
HN
O
N
H
NHCbz
F
Me
O
Me
S-40
^{1}H NMR (400 MHz, DMSO-d_6)
1.00 2.01 1.13 1.10 13.06 0.95 0.88 4.27 1.00 3.11 2.36 2.18 1.00 1.04 2.04 2.09 2.04 9.19 1.10 15.20 6.07
13 12 11 10 9 8 7 6 5 4 3 2 1 0 −1 −2
f1 (ppm)

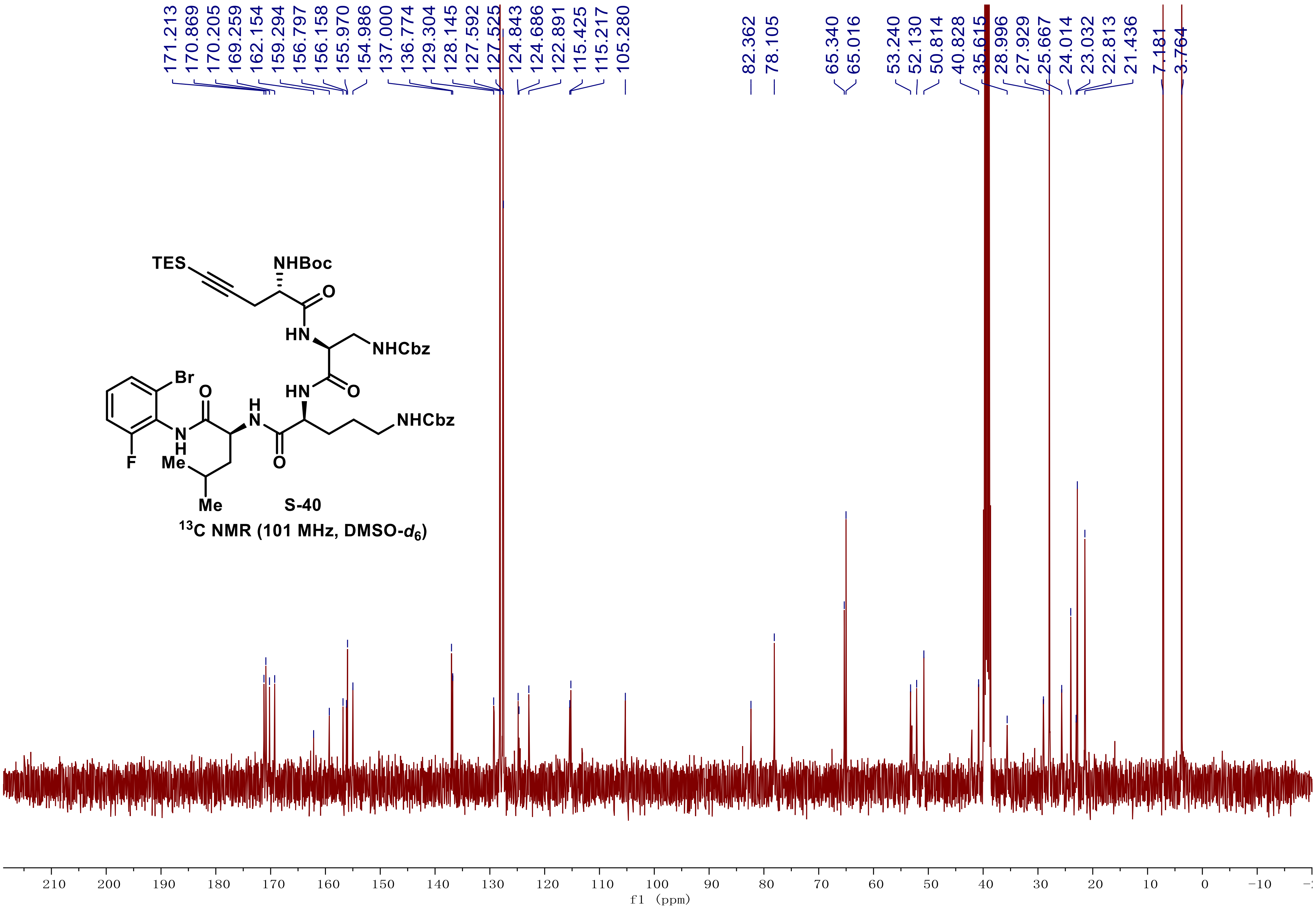
171.213
170.869
170.205
169.259
162.154
159.294
156.797
156.158
155.970
154.986
137.000
136.774
129.304
128.145
127.592
127.525
124.843
124.686
122.891
115.425
115.217
105.280
82.362
78.105
65.340
65.016
53.240
52.130
50.814
40.828
35.615
28.996
27.929
25.667
24.014
23.032
22.813
21.436
7.181
3.764
TES
NHBoc
NHCbz
Br
F
Me
S-40
13C NMR (101 MHz, DMSO-d6)
f1 (ppm)

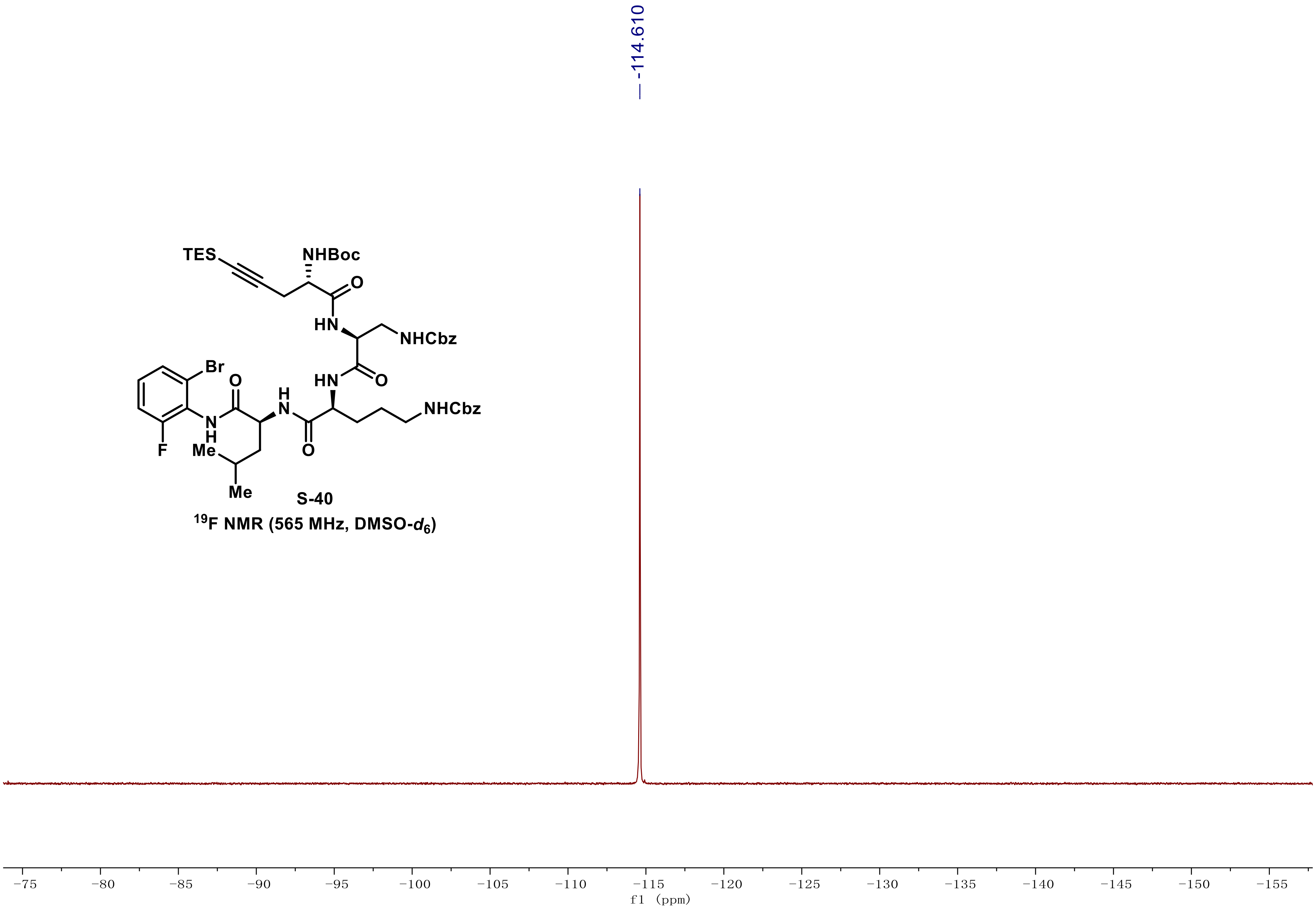
-114.610
TES
NHBoc
O
HN
NHCbz
Br
O
H
N
HN
O
NHCbz
N
H
F
Me
O
Me
S-40
^{19}F NMR (565 MHz, DMSO-d_6)
−75 −80 −85 −90 −95 −100 −105 −110 −115 −120 −125 −130 −135 −140 −145 −150 −155
f1 (ppm)

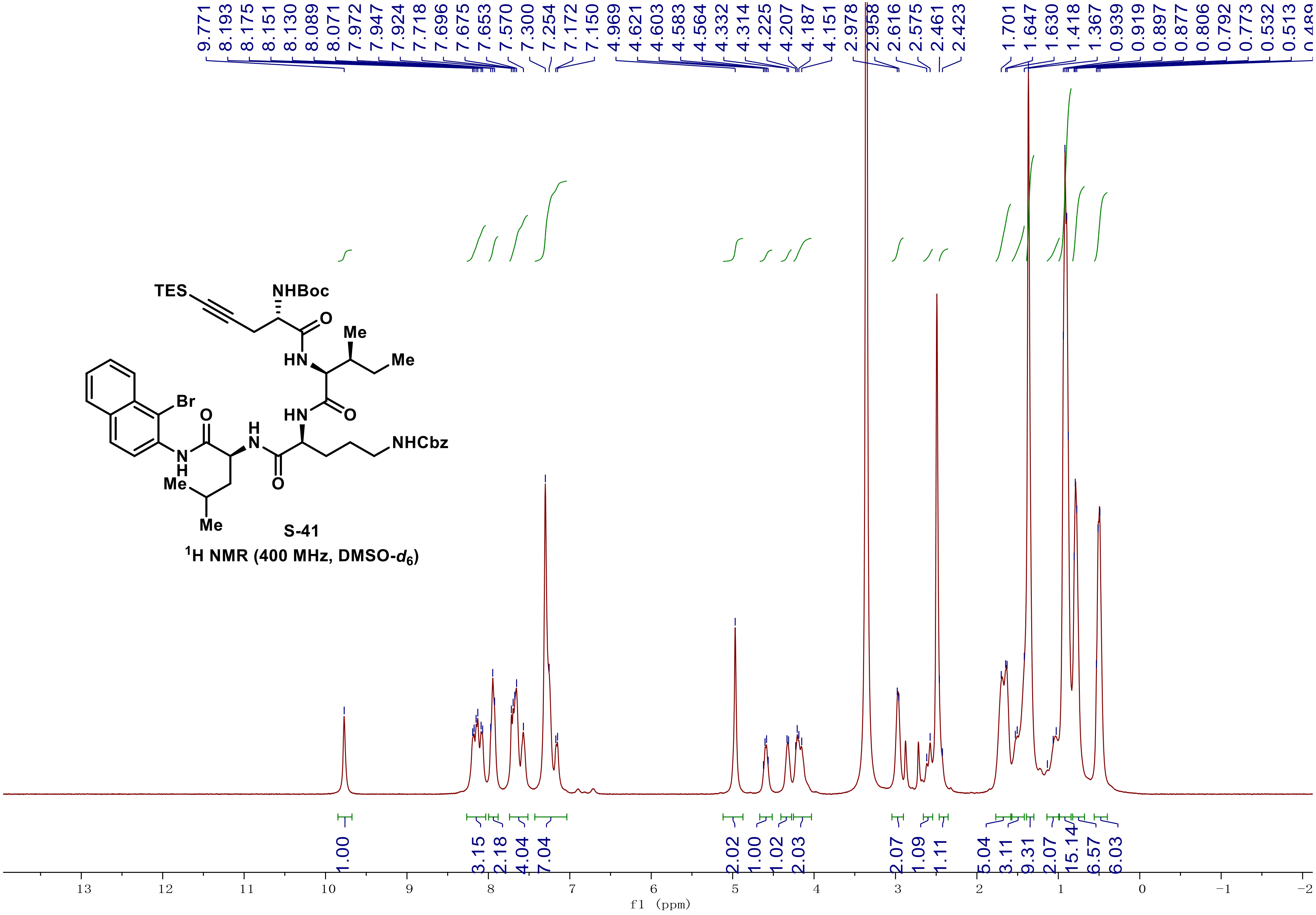

9.771
8.193
8.175
8.151
8.130
8.089
8.071
7.972
7.947
7.924
7.718
7.696
7.675
7.653
7.570
7.300
7.254
7.172
7.150
4.969
4.621
4.603
4.583
4.564
4.332
4.314
4.225
4.207
4.187
4.151
2.978
2.958
2.616
2.575
2.461
2.423
1.701
1.647
1.630
1.418
1.367
0.939
0.919
0.897
0.877
0.806
0.792
0.773
0.532
0.513
TES
NHBoc
O
Me
HN
Me
Br
O
HN
O
H
N
NHCbz
N
H
Me
O
Me
S-41
1H NMR (400 MHz, DMSO-d6)
1.00
3.15
2.18
4.04
7.04
2.02
1.00
1.02
2.03
2.07
1.09
1.11
5.04
3.11
9.31
2.07
15.14
6.57
6.03
13
12
11
10
9
8
7
6
5
4
3
2
1
0
-1
-2
f1 (ppm)

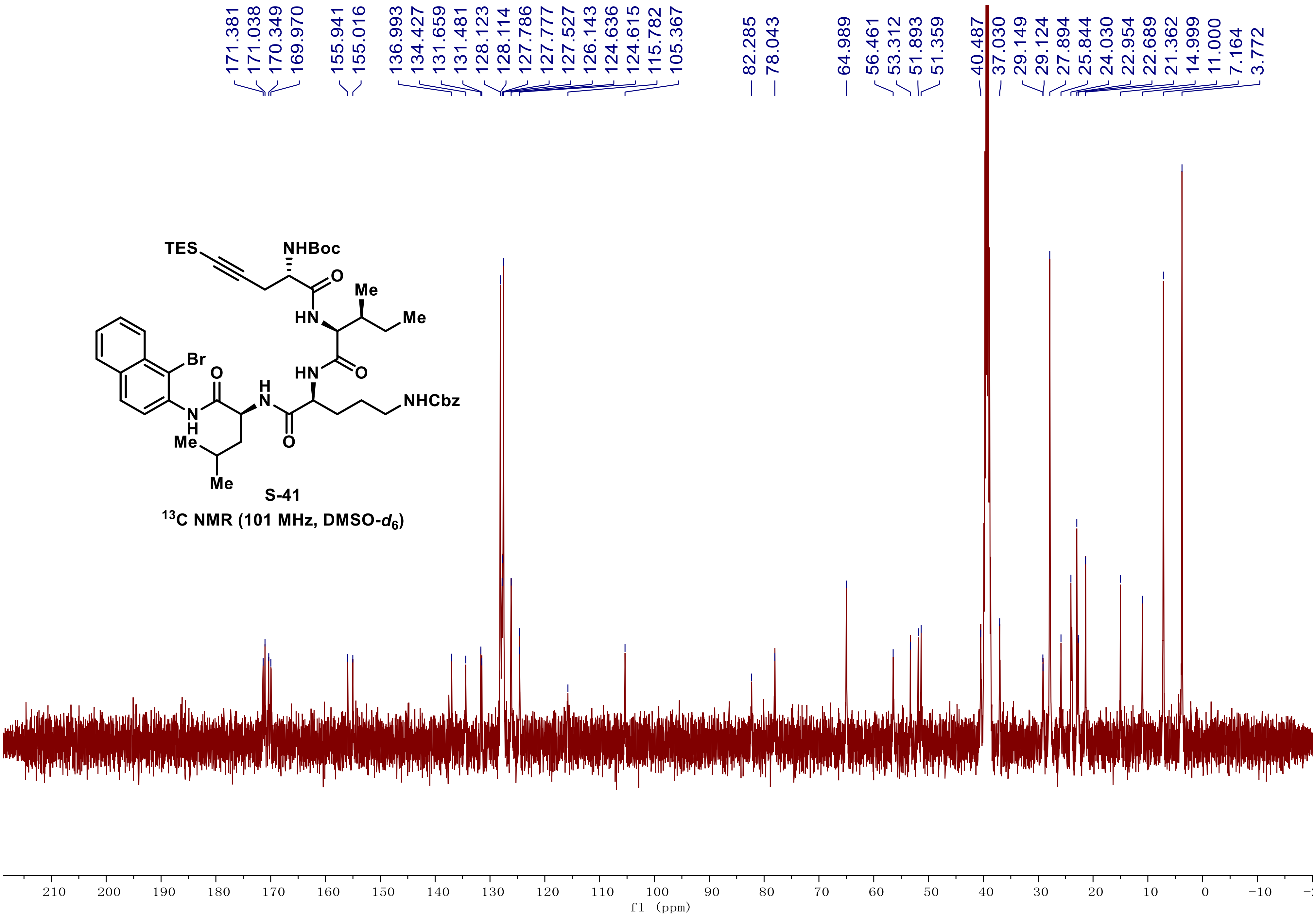

S-41

^{13}C NMR (101 MHz, DMSO-d_6)

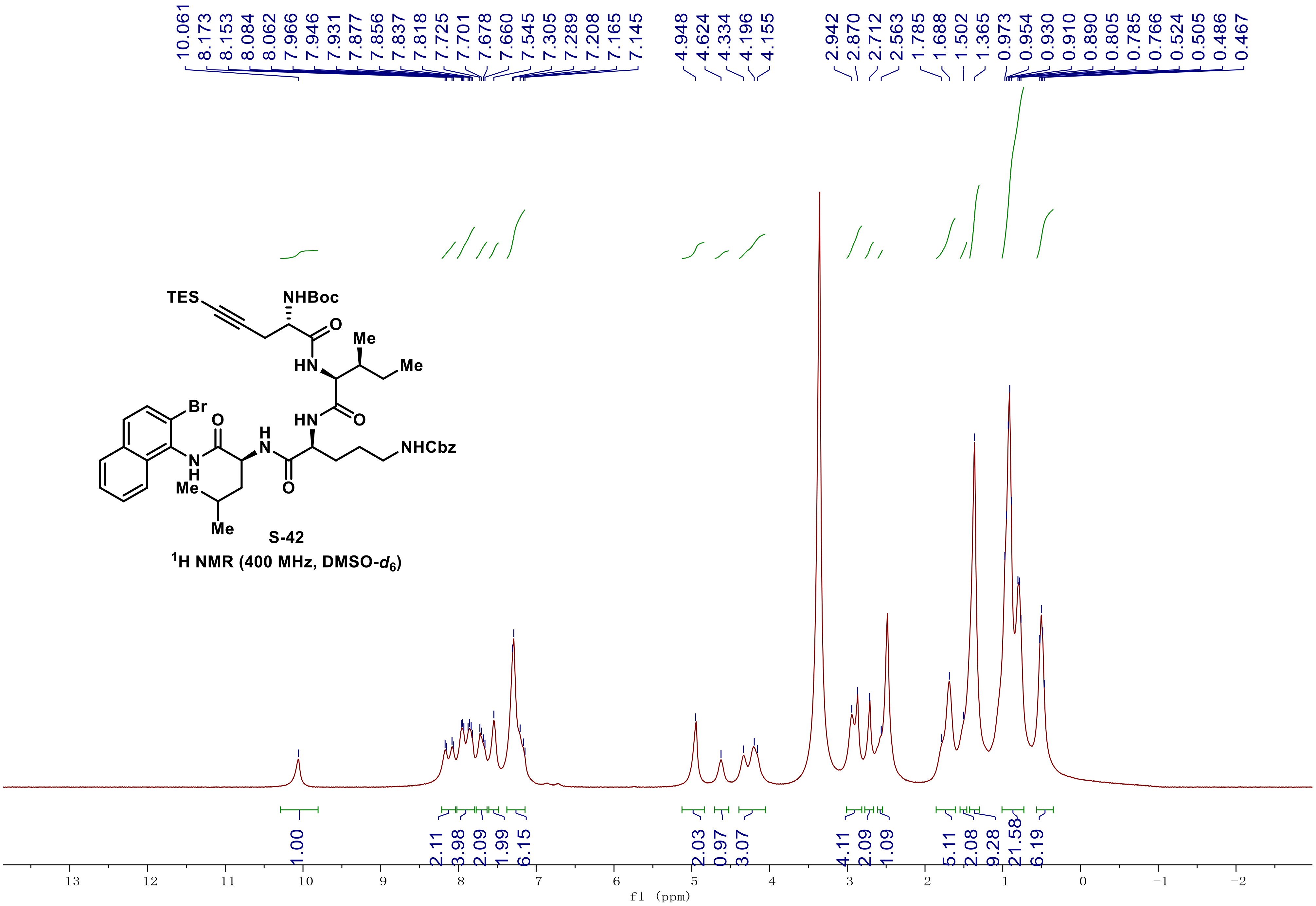

S-42

^{1}H NMR (400 MHz, DMSO-d_6)

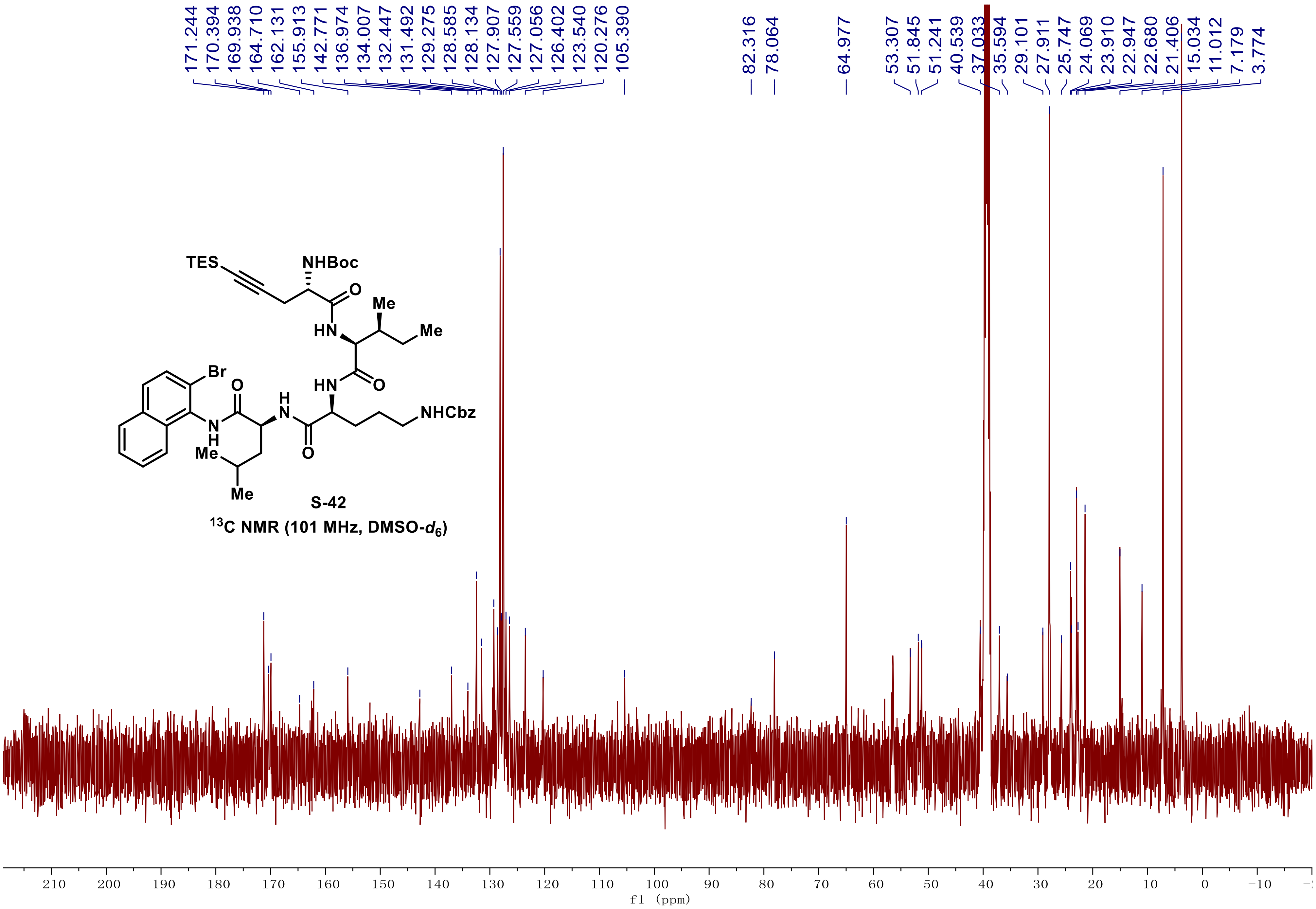
171.244
170.394
169.938
164.710
162.131
155.913
142.771
136.974
134.007
132.447
131.492
129.275
128.585
128.134
127.907
127.559
127.056
126.402
123.540
120.276
105.390
82.316
78.064
64.977
53.307
51.845
51.241
40.539
37.033
35.594
29.101
27.911
25.747
24.069
23.910
22.947
22.680
21.406
15.034
11.012
7.179
3.774
TES
NHBoc
O
Me
HN
Me
Br
O
HN
O
H
N
NHCbz
N
H
O
Me
Me
S-42
^{13}C NMR (101 MHz, DMSO-d_6)
f1 (ppm)

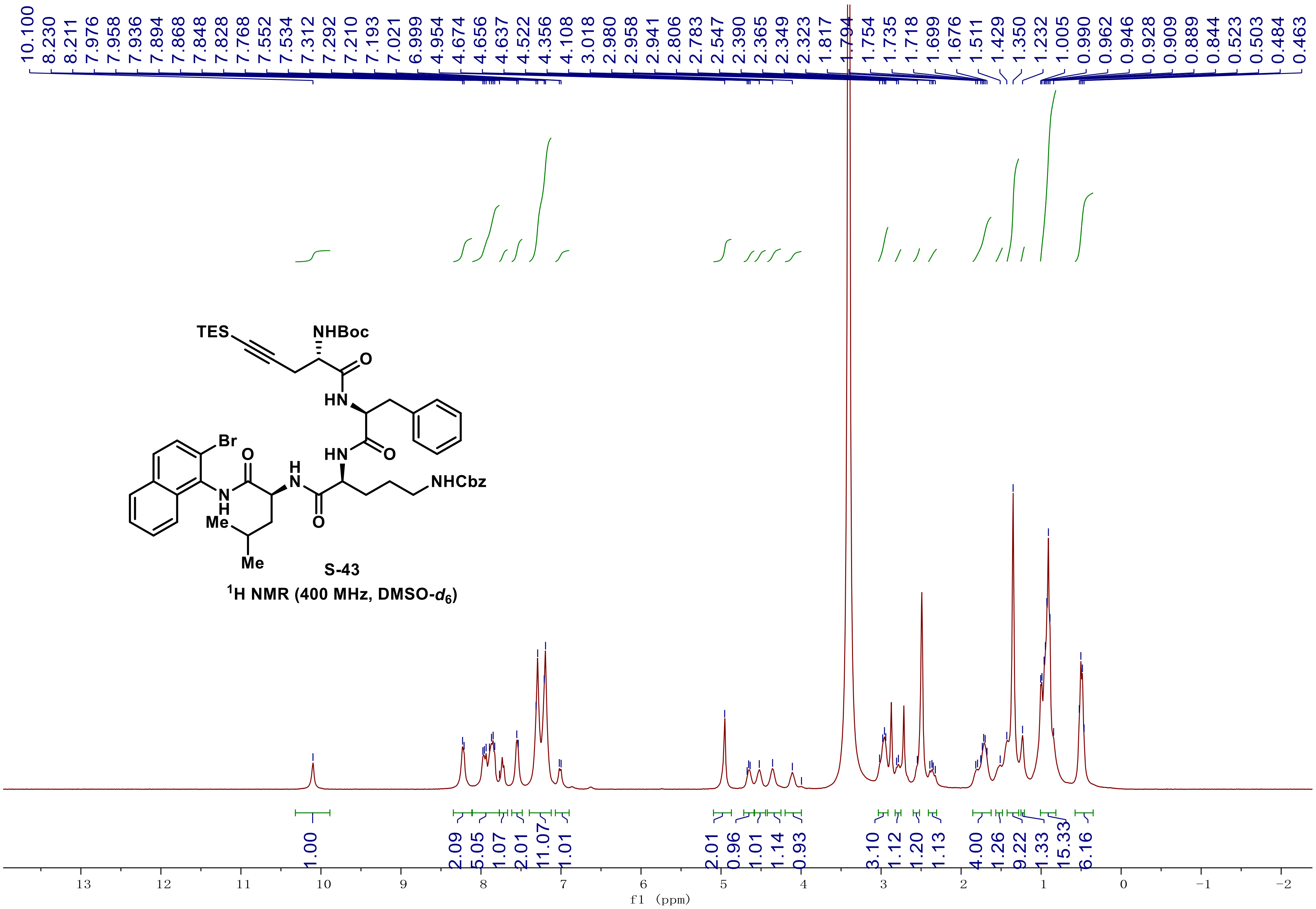
S-43
^{1}H NMR (400 MHz, DMSO-d_6)
f1 (ppm)

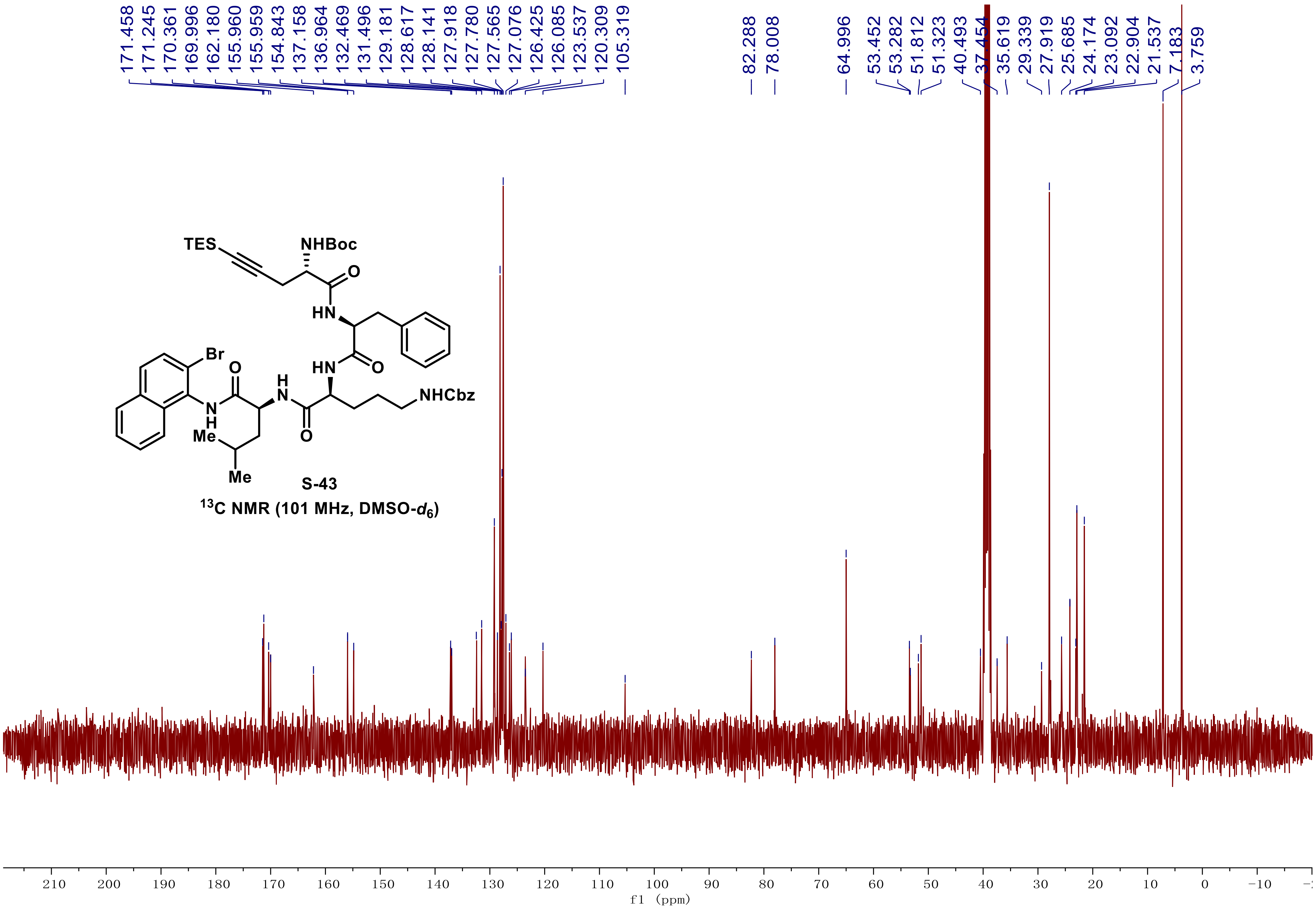
171.458
171.245
170.361
169.996
162.180
155.960
155.959
154.843
137.158
136.964
132.469
131.496
129.181
128.617
128.141
127.918
127.780
127.565
127.076
126.425
126.085
123.537
120.309
105.319
82.288
78.008
64.996
53.452
53.282
51.812
51.323
40.493
37.454
35.619
29.339
27.919
25.685
24.174
23.092
22.904
21.537
7.183
3.759
TES
NHBoc
O
HN
HN
O
Br
O
H
N
NHCbz
N
H
Me
O
Me
S-43
13C NMR (101 MHz, DMSO-d6)
f1 (ppm)

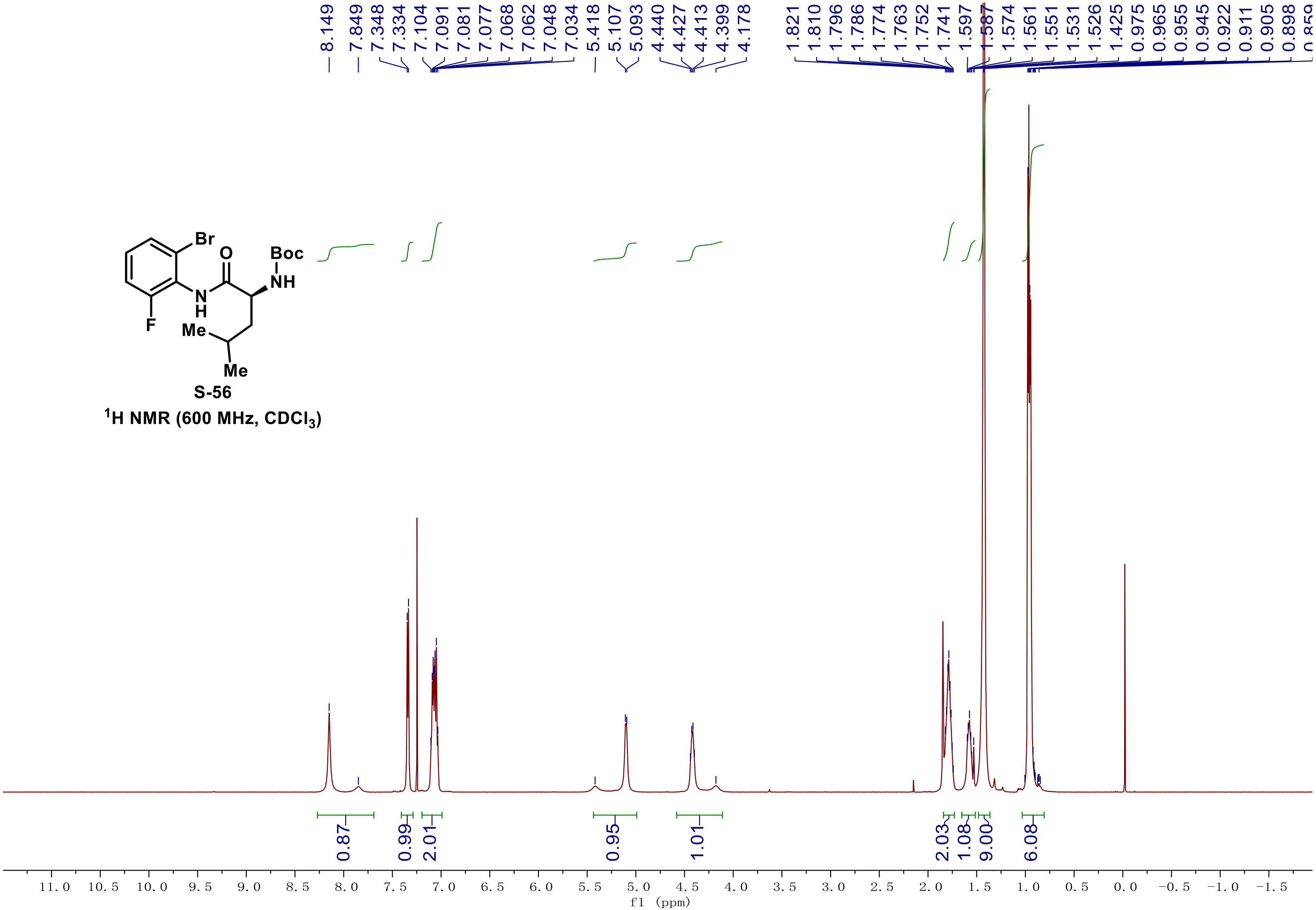

S-56

^{1}H NMR (600 MHz, $CDCl_3$)

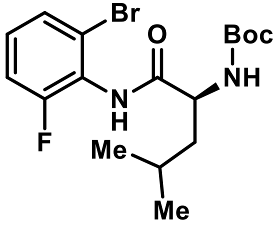

S-56

^{13}C NMR (151 MHz, $CDCl_3$)

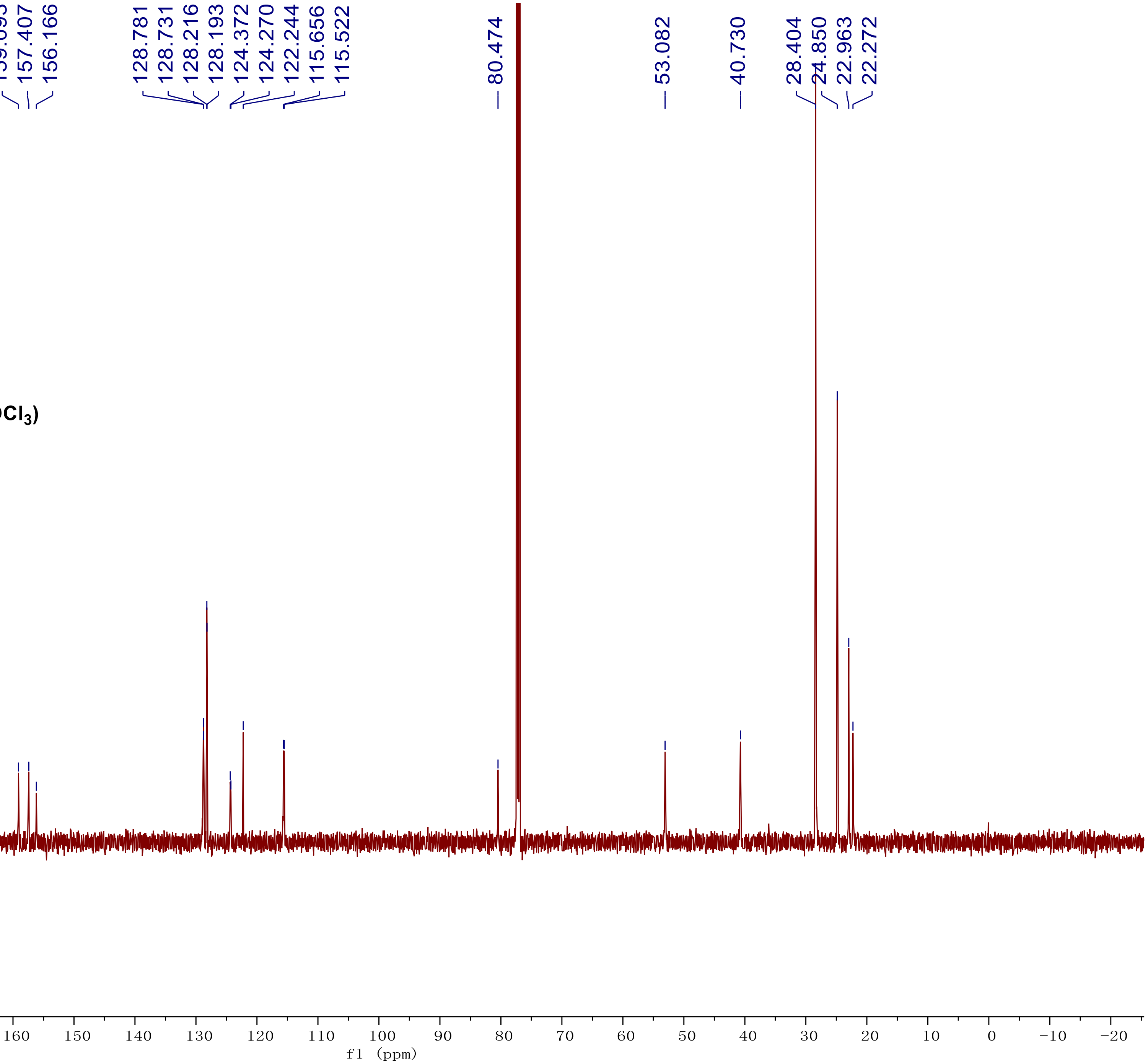

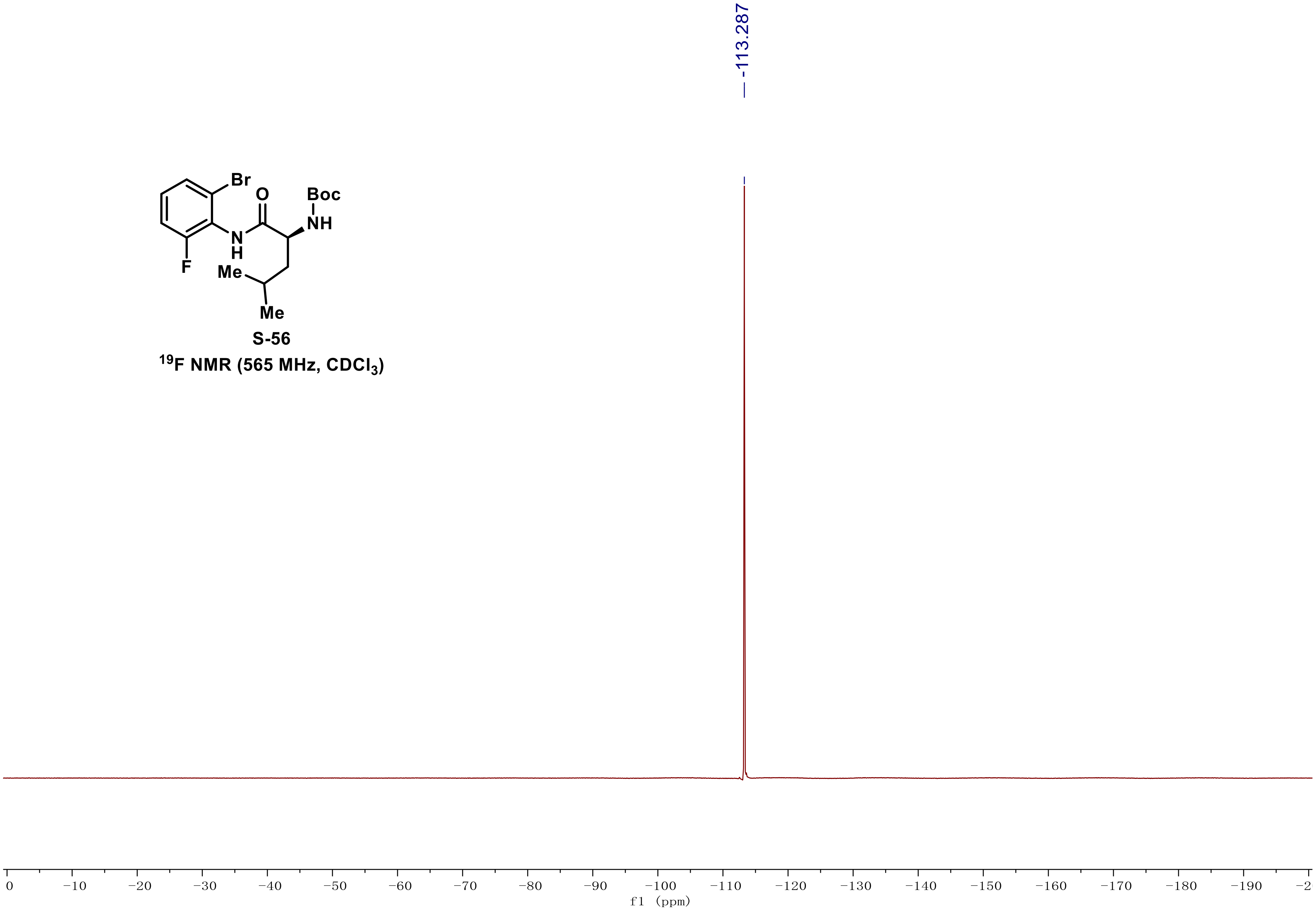

S-56
^{19}F NMR (565 MHz, $CDCl_3$)
-113.287
0
−10
−20
−30
−40
−50
−60
−70
−80
−90
−100
−110
−120
−130
−140
−150
−160
−170
−180
−190
f1 (ppm)

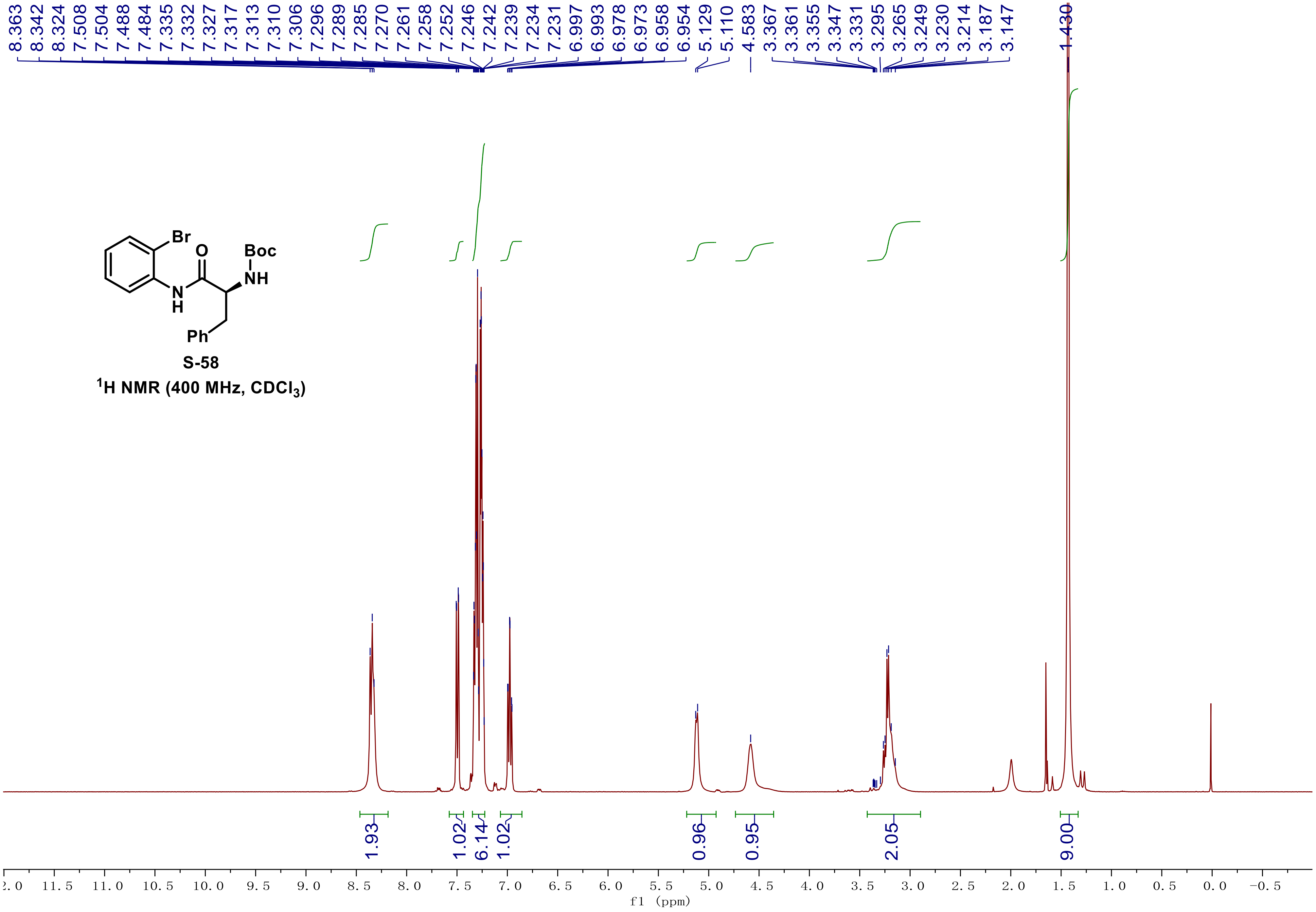

S-58

^{1}H NMR (400 MHz, CDCl$_3$)

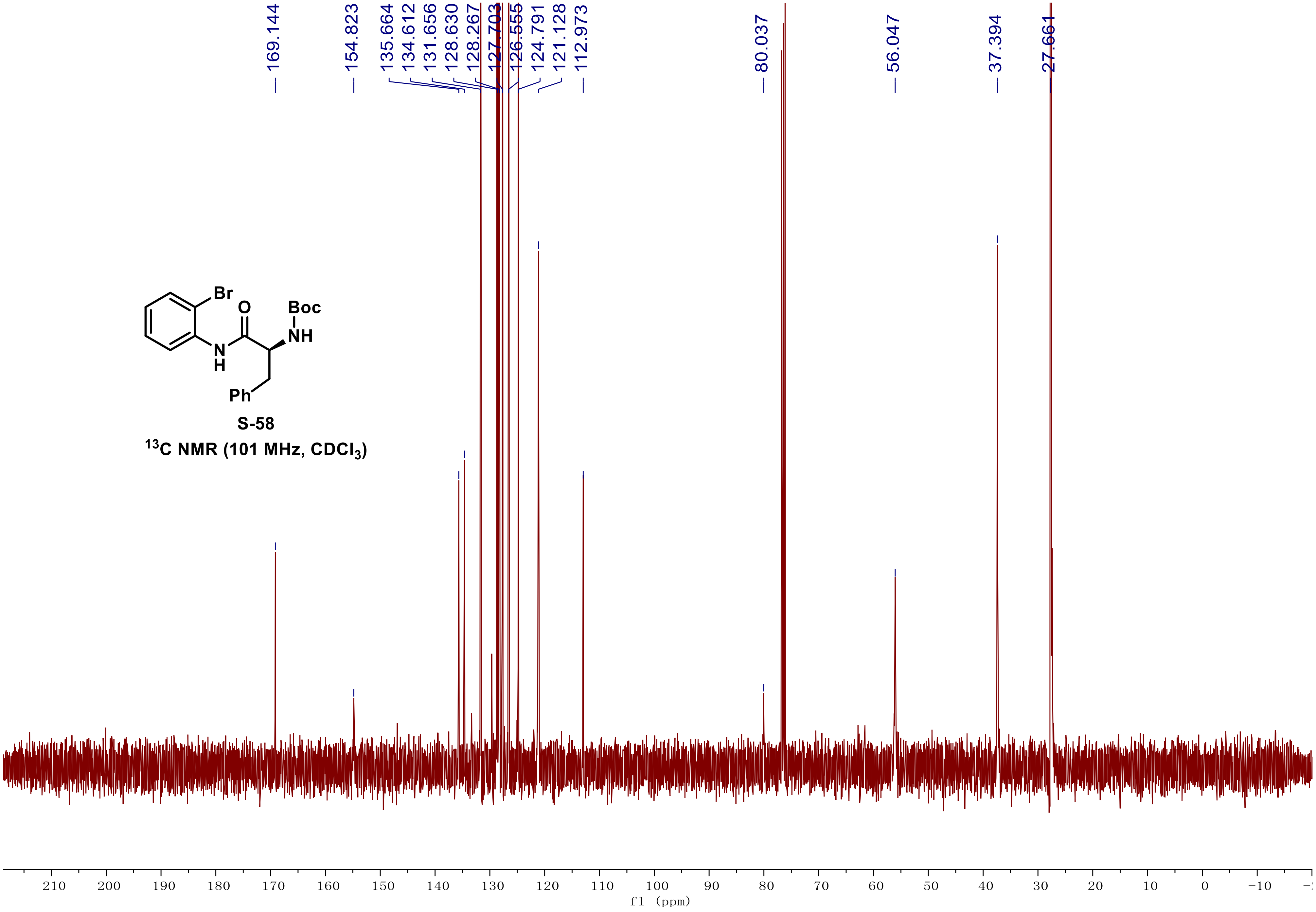
169.144
154.823
135.664
134.612
131.656
128.630
128.267
127.703
126.555
124.791
121.128
112.973
80.037
56.047
37.394
27.661
Br
O
Boc
NH
N
H
Ph
S-58
^{13}C NMR (101 MHz, $CDCl_3$)
210 200 190 180 170 160 150 140 130 120 110 100 90 80 70 60 50 40 30 20 10 0 −10
f1 (ppm)

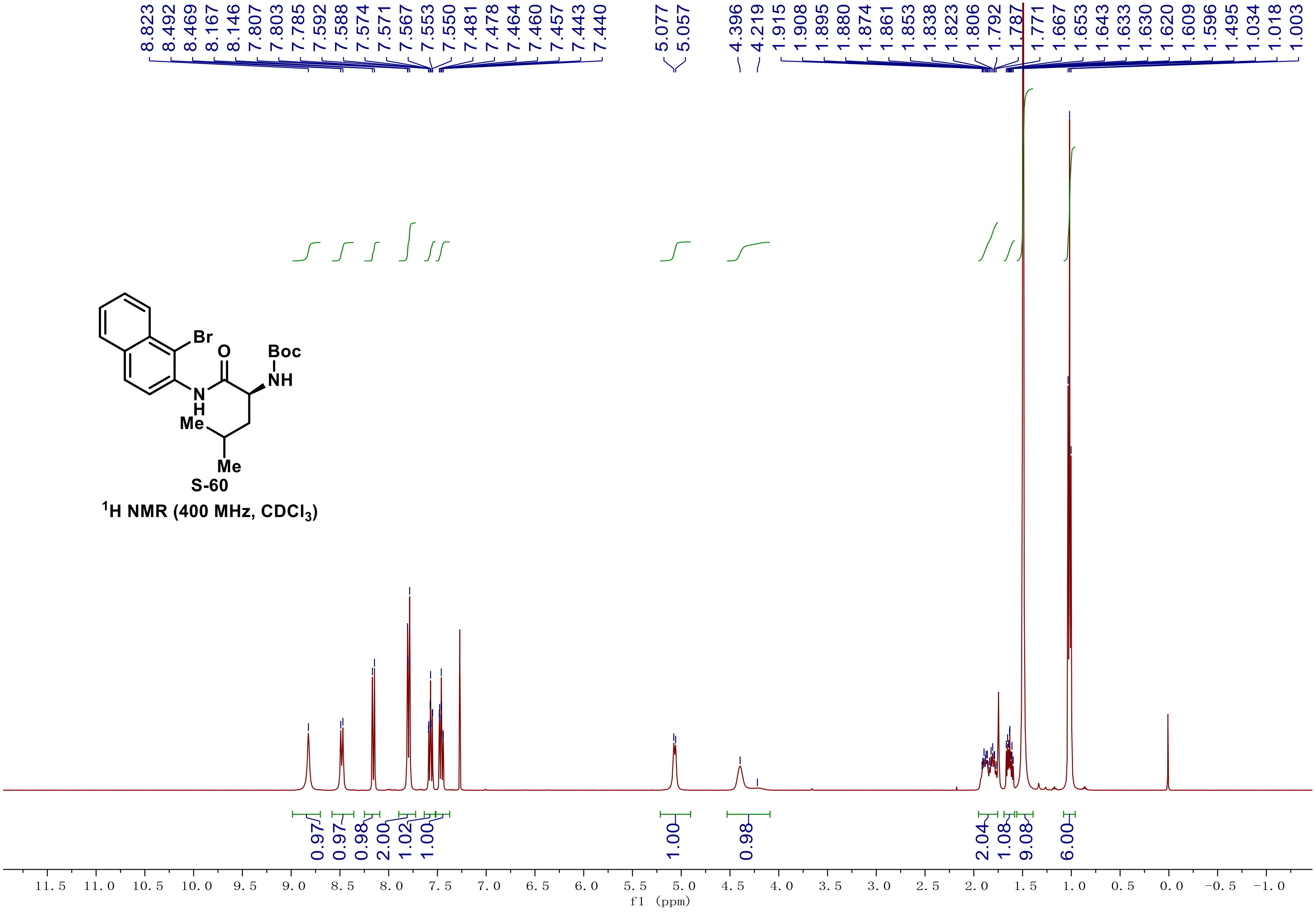
Br
O
Boc
NH
N
H
Me
Me
S-60
^{1}H NMR (400 MHz, CDCl$_3$)
8.823
8.492
8.469
8.167
8.146
7.807
7.803
7.785
7.592
7.588
7.574
7.571
7.567
7.553
7.550
7.481
7.478
7.464
7.460
7.457
7.443
7.440
5.077
5.057
4.396
4.219
1.915
1.908
1.895
1.880
1.874
1.861
1.853
1.838
1.823
1.806
1.792
1.787
1.771
1.667
1.653
1.643
1.633
1.630
1.620
1.609
1.596
1.495
1.034
1.018
1.003
0.97
0.97
0.98
2.00
1.02
1.00
1.00
0.98
2.04
1.08
9.08
6.00
f1 (ppm)

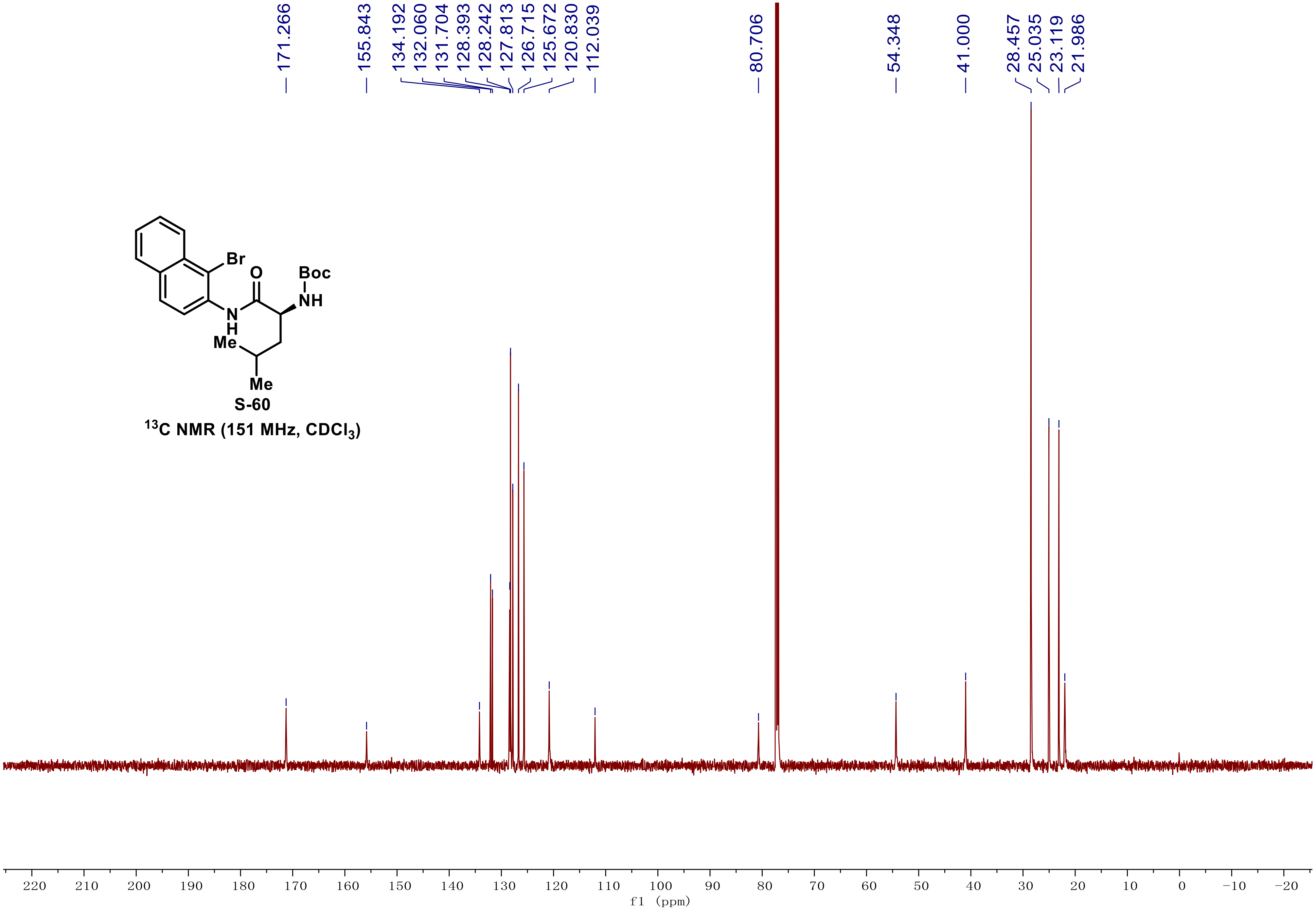

S-60

^{13}C NMR (151 MHz, $CDCl_3$)

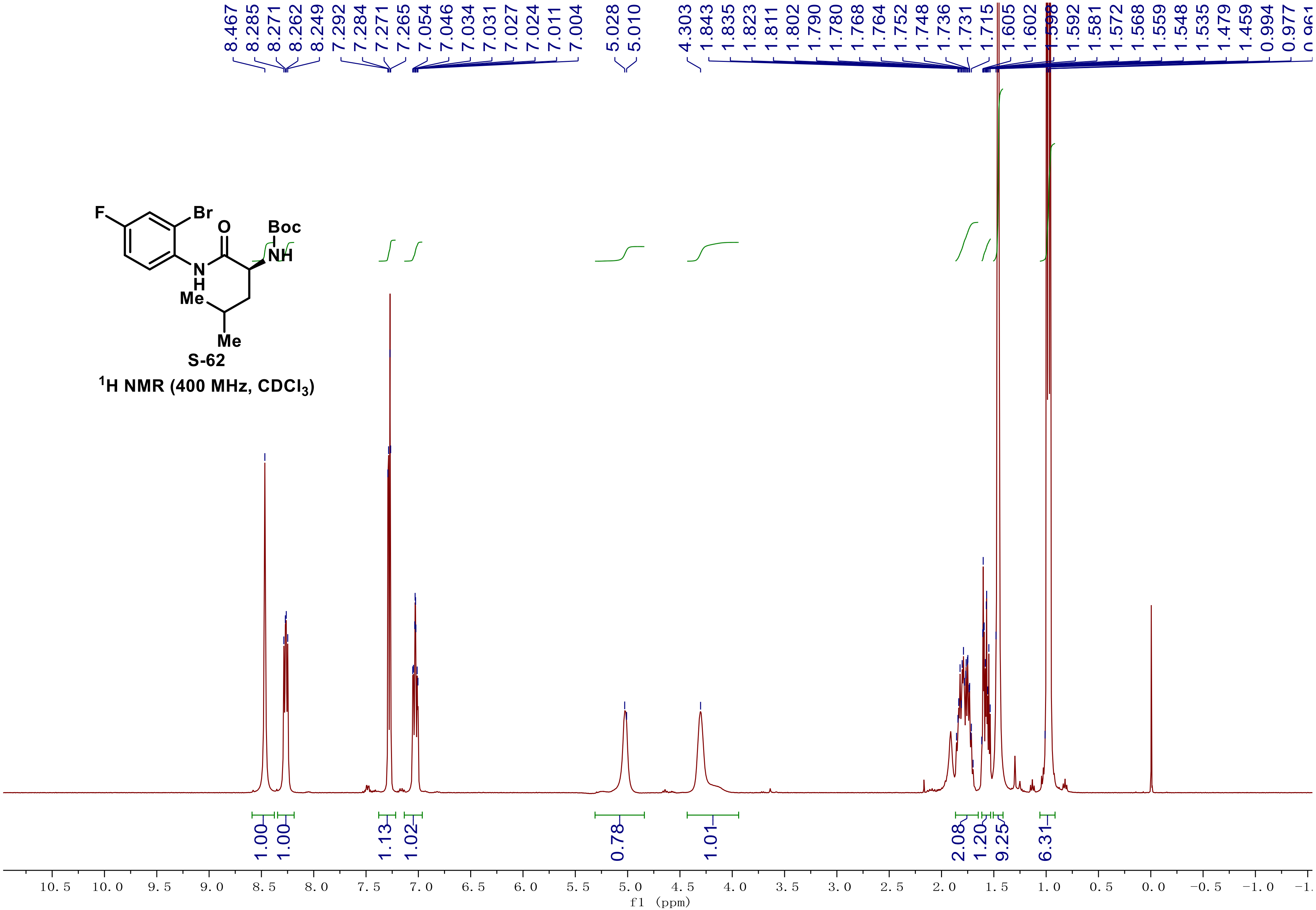
8.467 8.285 8.271 8.262 8.249 7.292 7.284 7.271 7.265 7.054 7.046 7.034 7.031 7.027 7.024 7.011 7.004 5.028 5.010 4.303 1.843 1.835 1.823 1.811 1.802 1.790 1.780 1.768 1.764 1.752 1.748 1.736 1.731 1.715 1.605 1.602 1.592 1.581 1.572 1.568 1.559 1.548 1.535 1.479 1.459 0.994 0.977
F
Br
O
Boc
NH
N
H
Me
Me
S-62
1H NMR (400 MHz, CDCl3)
1.00 1.00 1.13 1.02 0.78 1.01 2.08 1.20 9.25 6.31
f1 (ppm)

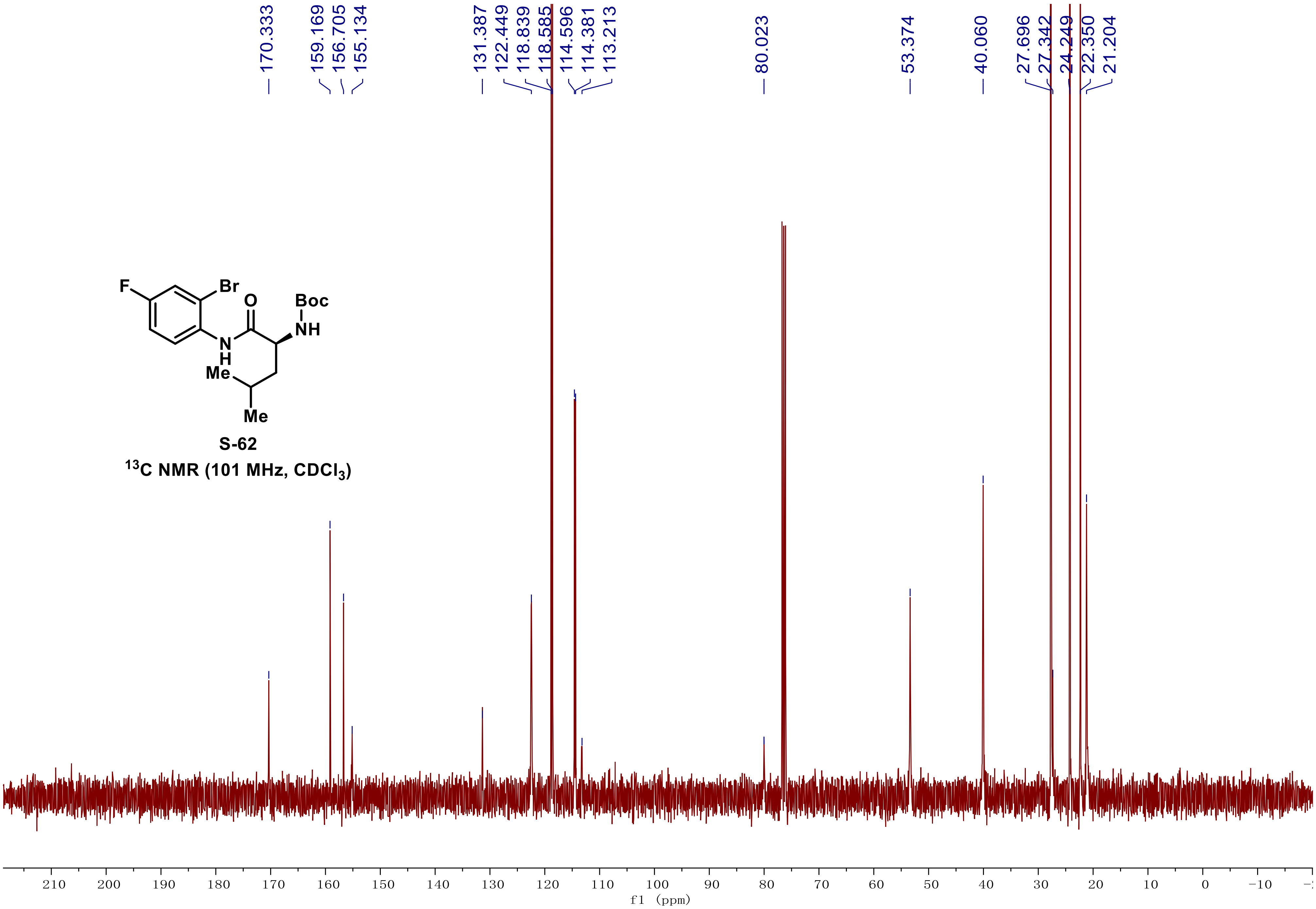

S-62

^{13}C NMR (101 MHz, $CDCl_3$)

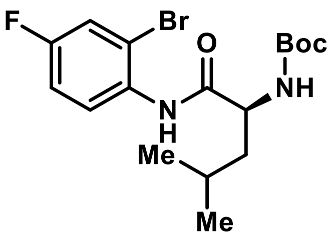

S-62

^{19}F NMR (565 MHz, $CDCl_3$)

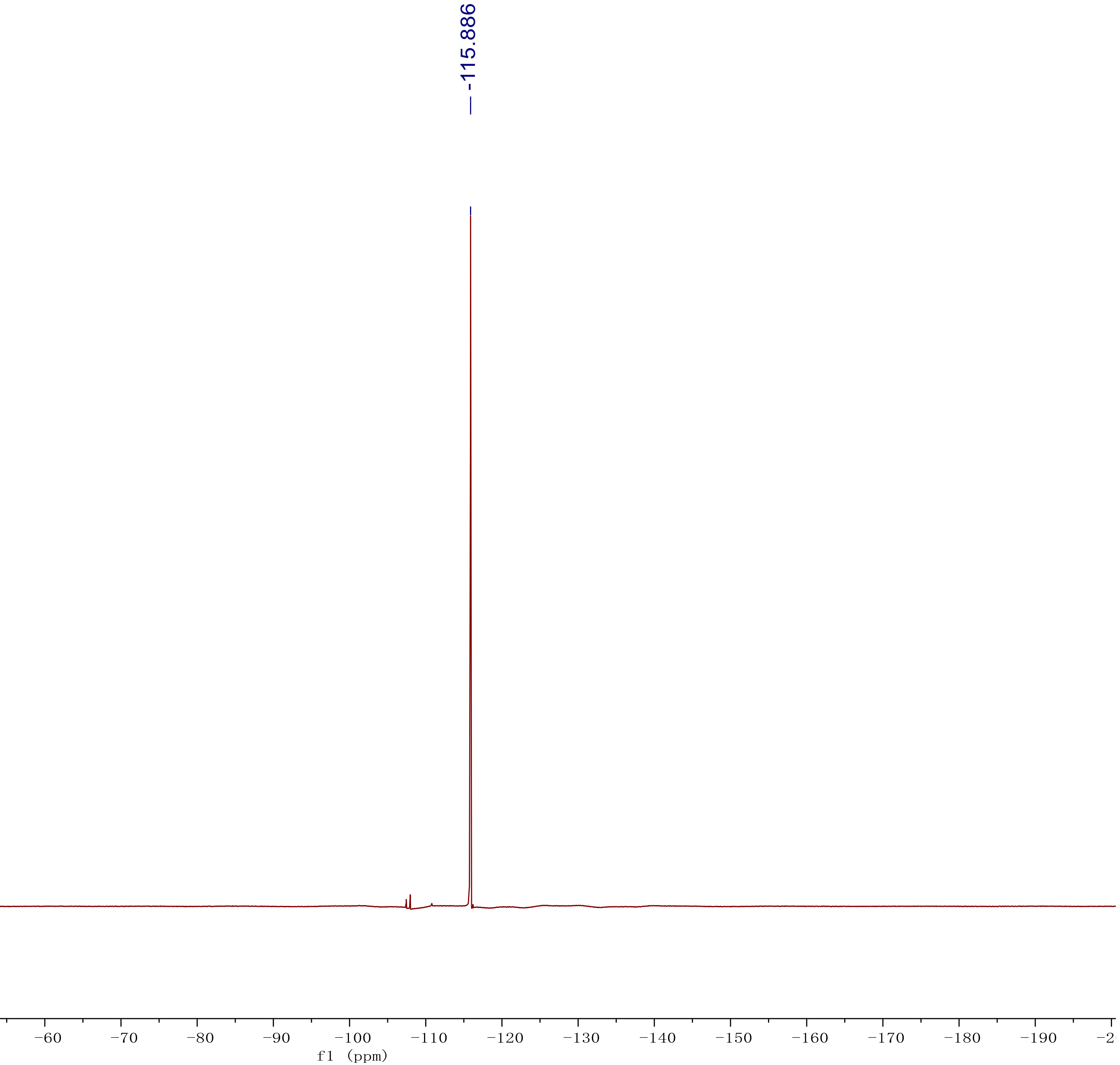

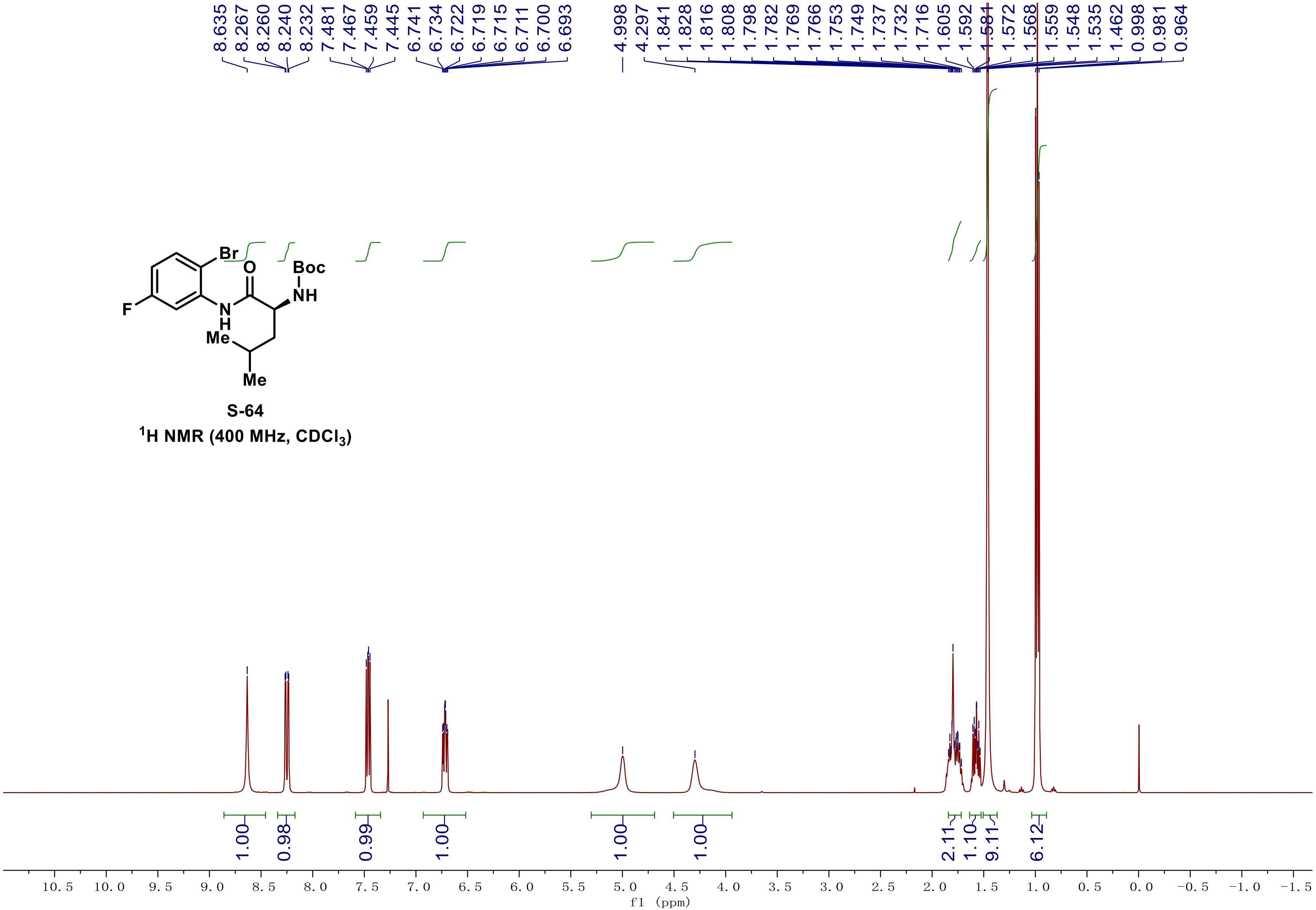
8.635
8.267
8.260
8.240
8.232
7.481
7.467
7.459
7.445
6.741
6.734
6.722
6.719
6.715
6.711
6.700
6.693
4.998
4.297
1.841
1.828
1.816
1.808
1.798
1.782
1.769
1.766
1.753
1.749
1.737
1.732
1.716
1.605
1.592
1.581
1.572
1.568
1.559
1.548
1.535
1.462
0.998
0.981
0.964
Br
O
Boc
NH
F
N
H
Me
Me
S-64
1H NMR (400 MHz, CDCl3)
1.00
0.98
0.99
1.00
1.00
1.00
2.11
1.10
9.11
6.12
f1 (ppm)

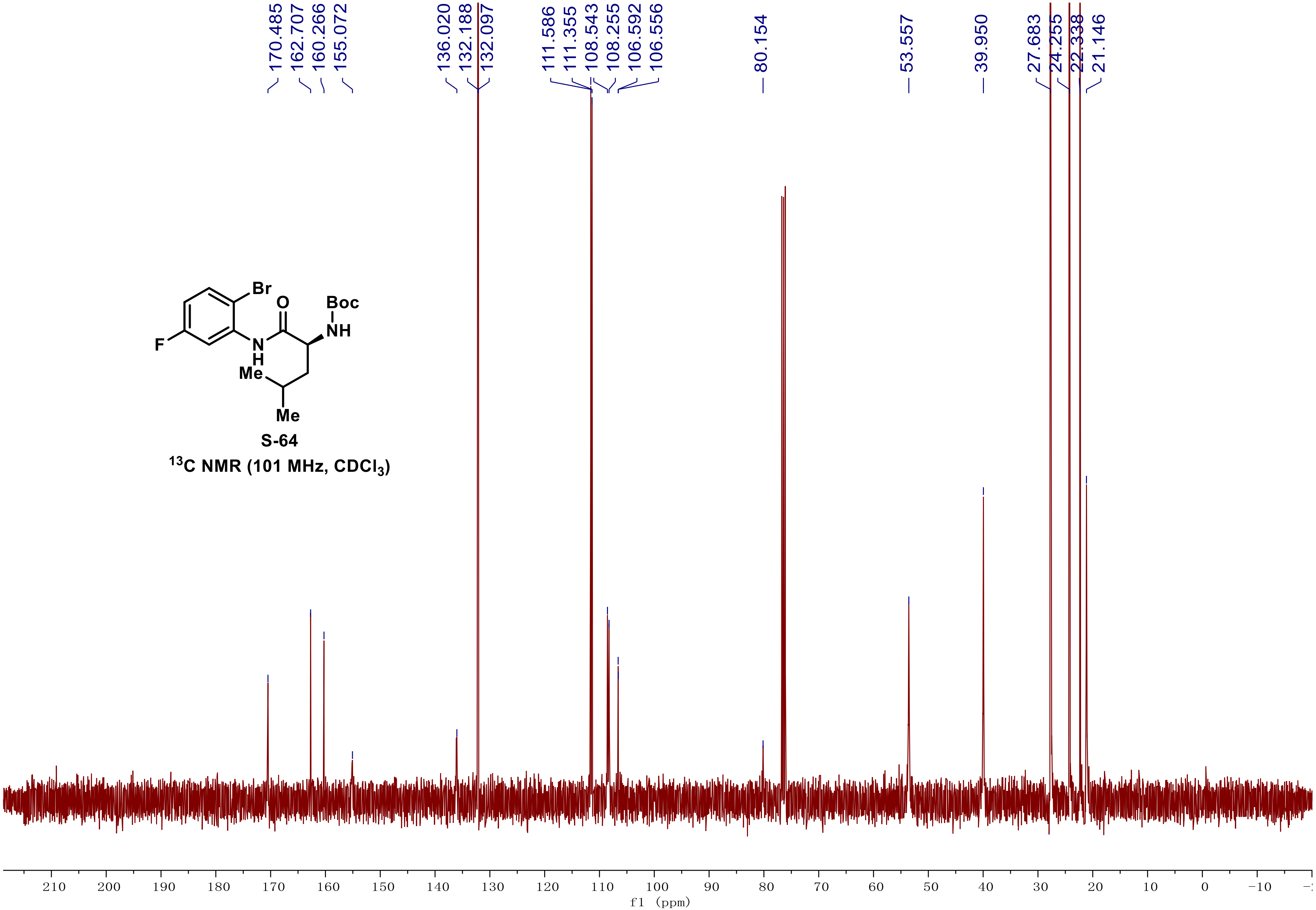
170.485
162.707
160.266
155.072
136.020
132.188
132.097
111.586
111.355
108.543
108.255
106.592
106.556
80.154
53.557
39.950
27.683
24.255
22.338
21.146
Br
O
Boc
NH
F
N
H
Me
Me
S-64
^{13}C NMR (101 MHz, $CDCl_3$)
f1 (ppm)

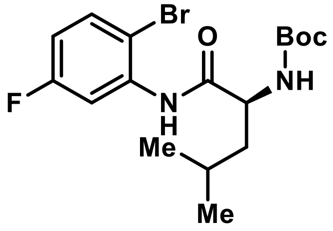

S-64

^{19}F NMR (565 MHz, $CDCl_3$)

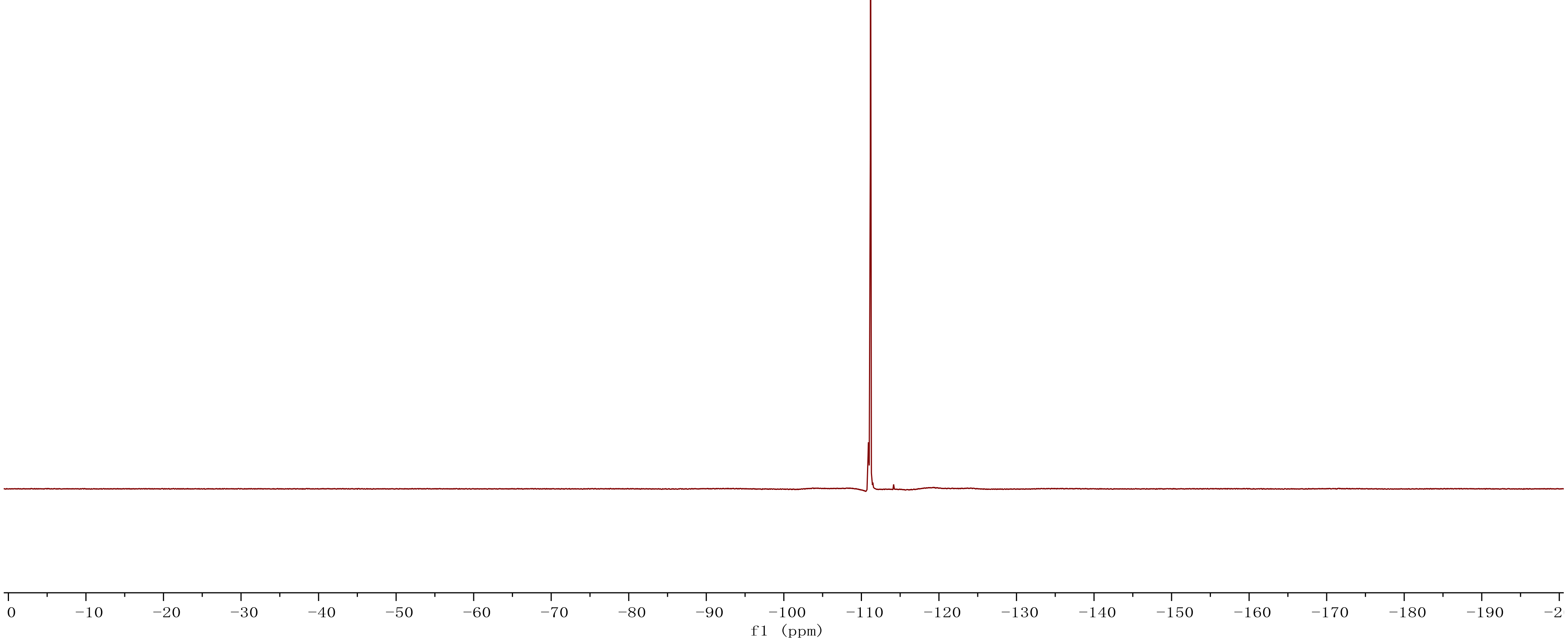

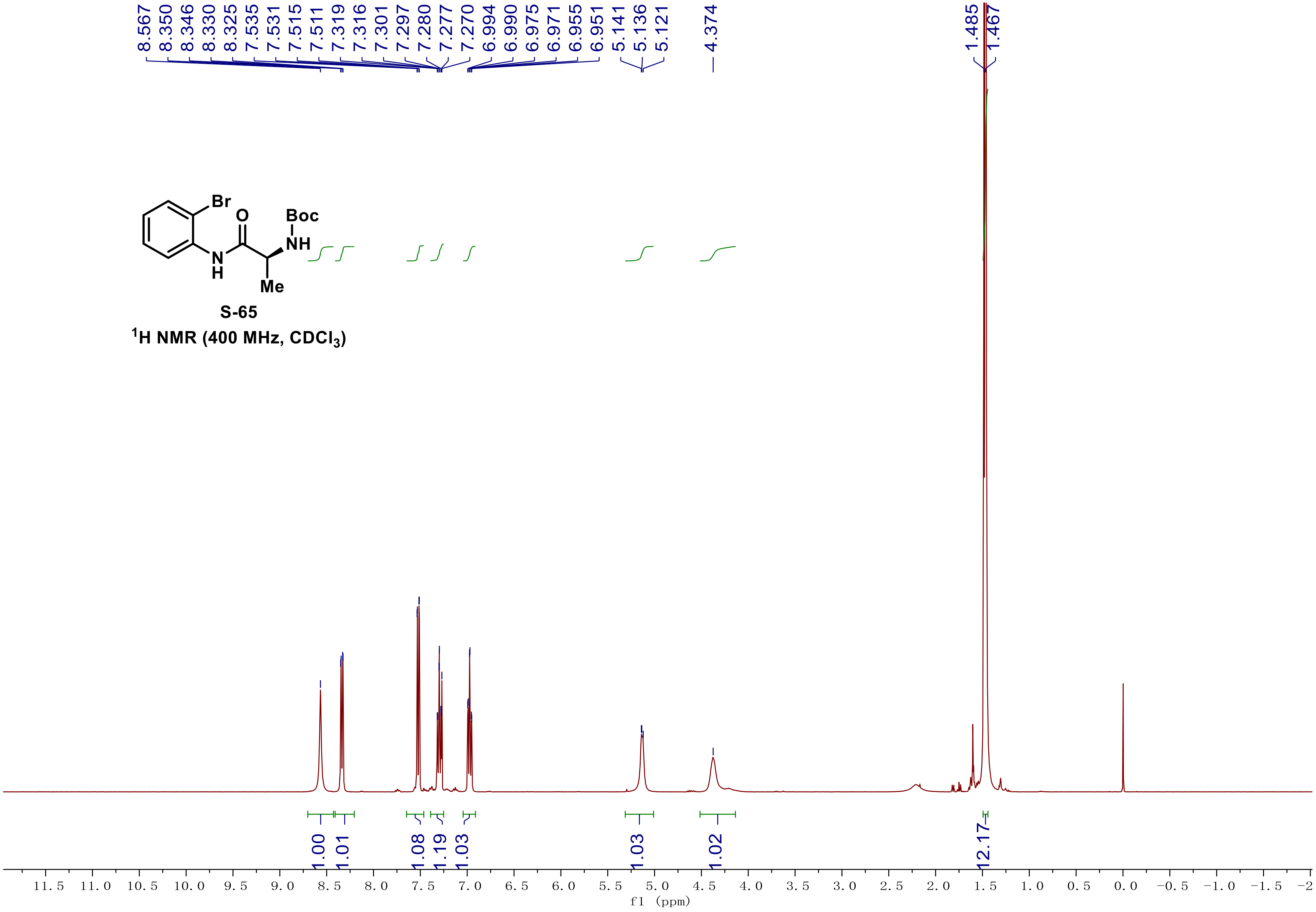
8.567
8.350
8.346
8.330
8.325
7.535
7.531
7.515
7.511
7.319
7.316
7.301
7.297
7.280
7.277
7.270
6.994
6.990
6.975
6.971
6.955
6.951
5.141
5.136
5.121
4.374
1.485
1.467
Br
O
Boc
NH
N
H
Me
S-65
1H NMR (400 MHz, CDCl3)
1.00
1.01
1.08
1.19
1.03
1.03
1.02
12.17
f1 (ppm)

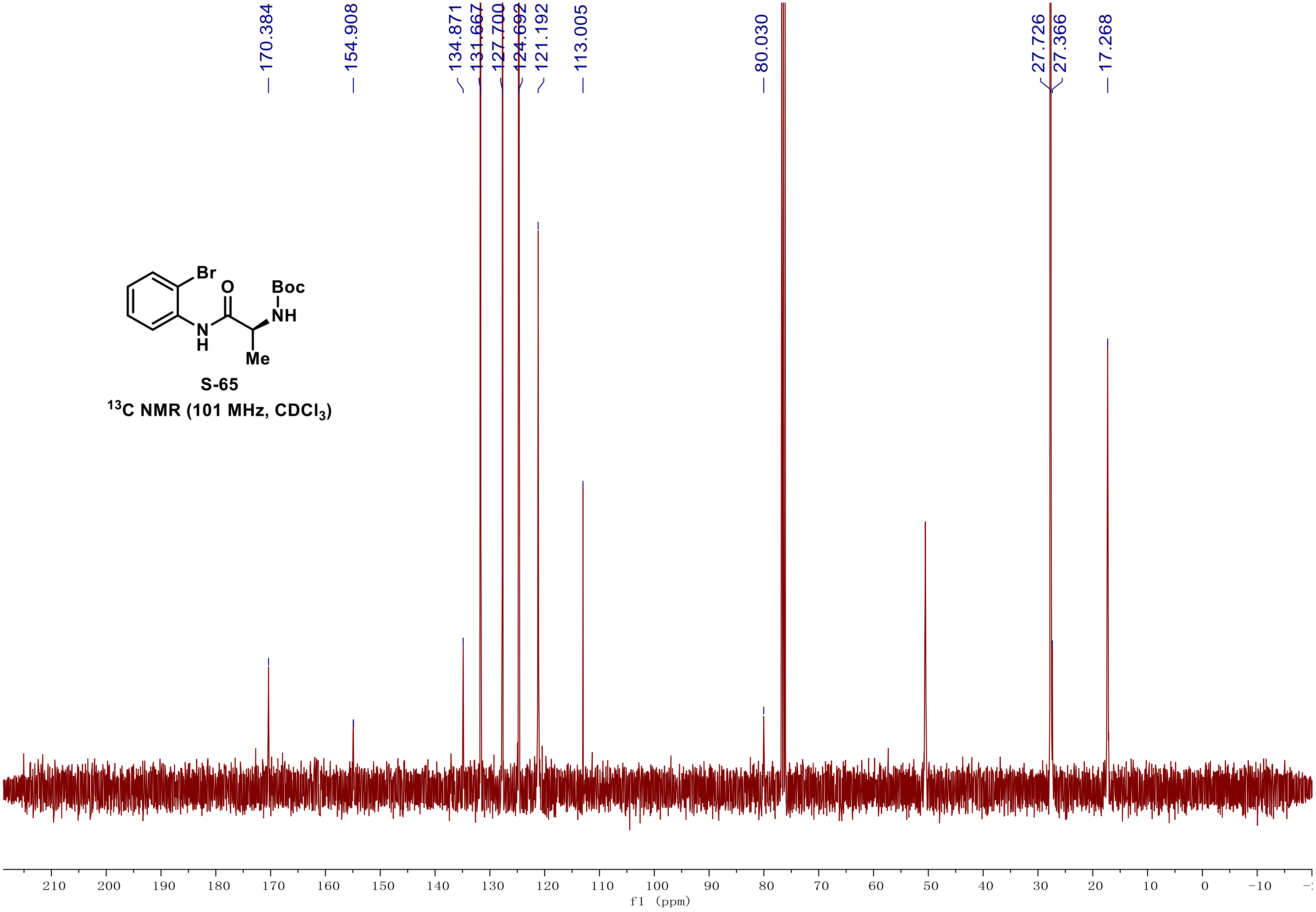

S-65

^{13}C NMR (101 MHz, CDCl$_3$)

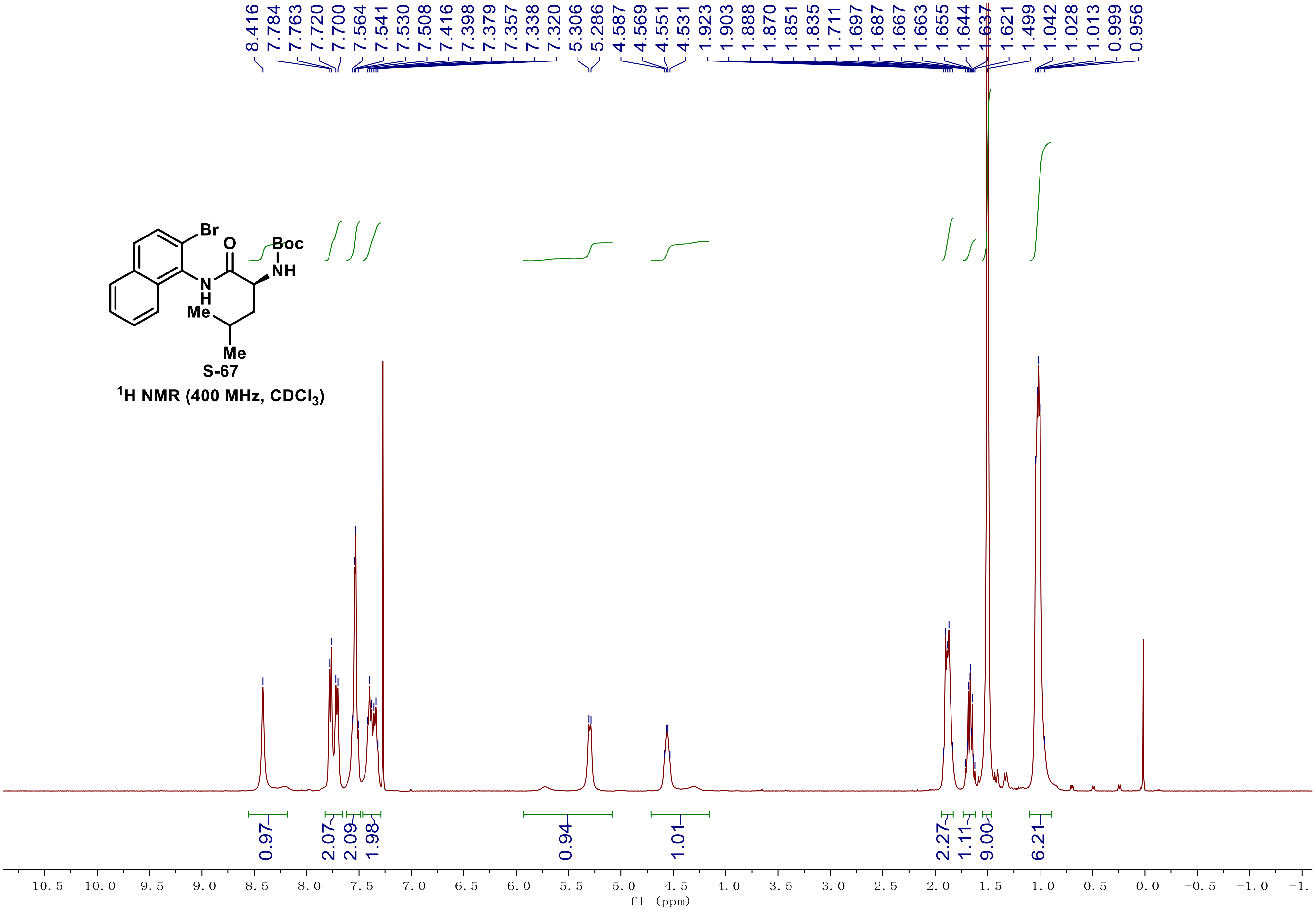
8.416
7.784
7.763
7.720
7.700
7.564
7.541
7.530
7.508
7.416
7.398
7.379
7.357
7.338
7.320
5.306
5.286
4.587
4.569
4.551
4.531
1.923
1.903
1.888
1.870
1.851
1.835
1.711
1.697
1.687
1.667
1.663
1.655
1.644
1.637
1.621
1.499
1.042
1.028
1.013
0.999
0.956
Br
O
Boc
NH
N
H
Me
Me
S-67
^{1}H NMR (400 MHz, CDCl$_3$)
0.97
2.07
2.09
1.98
0.94
1.01
2.27
1.11
9.00
6.21
10.5
10.0
9.5
9.0
8.5
8.0
7.5
7.0
6.5
6.0
5.5
5.0
4.5
4.0
3.5
3.0
2.5
2.0
1.5
1.0
0.5
0.0
−0.5
−1.0
f1 (ppm)

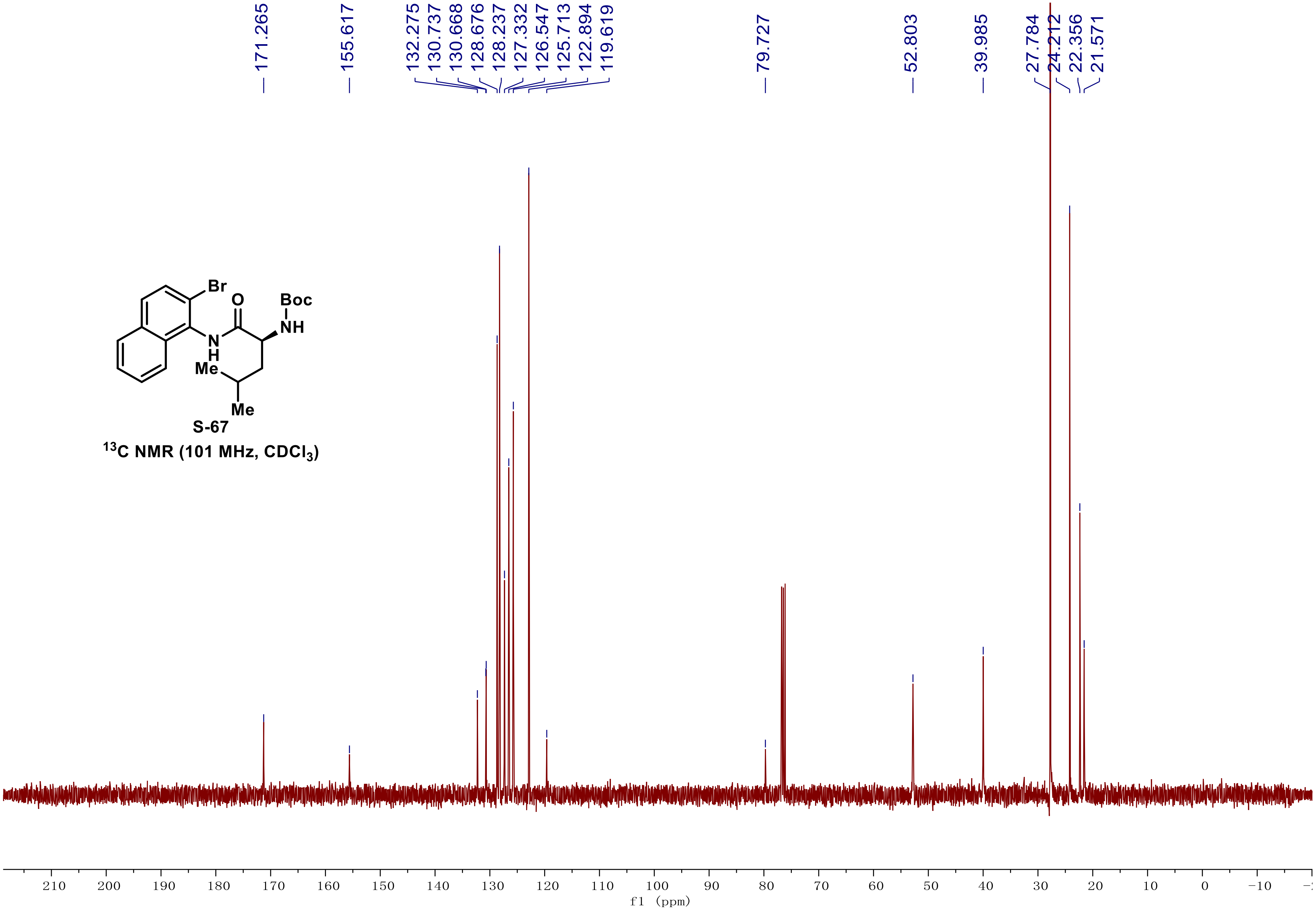

171.265
155.617
132.275
130.737
130.668
128.676
128.237
127.332
126.547
125.713
122.894
119.619
79.727
52.803
39.985
27.784
24.212
22.356
21.571
Br
O
Boc
NH
N
H
Me
Me
S-67
^{13}C NMR (101 MHz, $CDCl_3$)
210
200
190
180
170
160
150
140
130
120
110
100
90
80
70
60
50
40
30
20
10
0
−10
f1 (ppm)

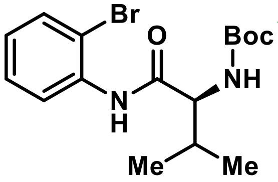

S-69

^{1}H NMR (400 MHz, $CDCl_3$)

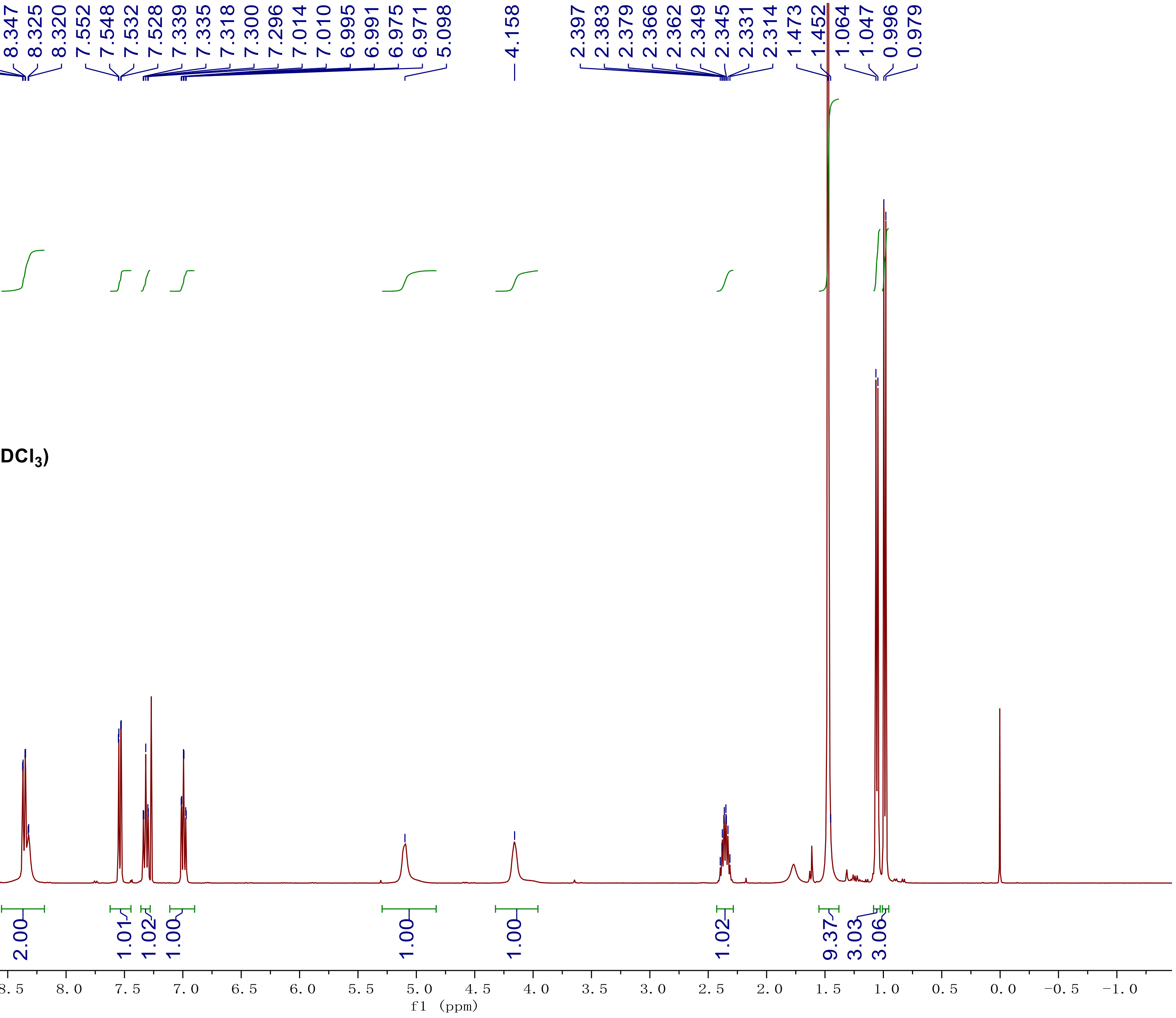

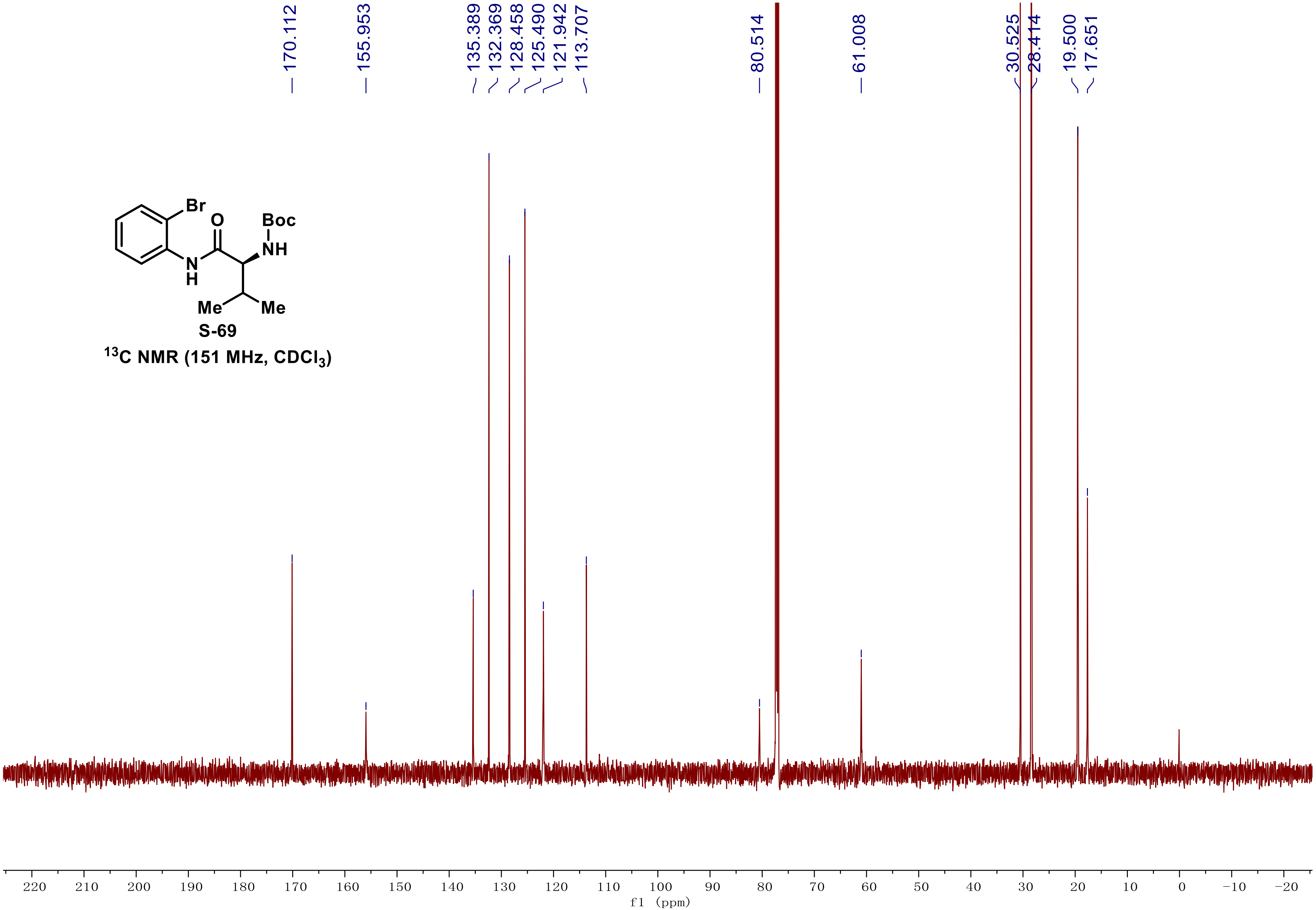
170.112
155.953
135.389
132.369
128.458
125.490
121.942
113.707
80.514
61.008
30.525
28.414
19.500
17.651
Br
O
Boc
NH
N
H
Me
Me
S-69
^{13}C NMR (151 MHz, $CDCl_3$)
220
210
200
190
180
170
160
150
140
130
120
110
100
90
80
70
60
50
40
30
20
10
0
−10
−20
f1 (ppm)

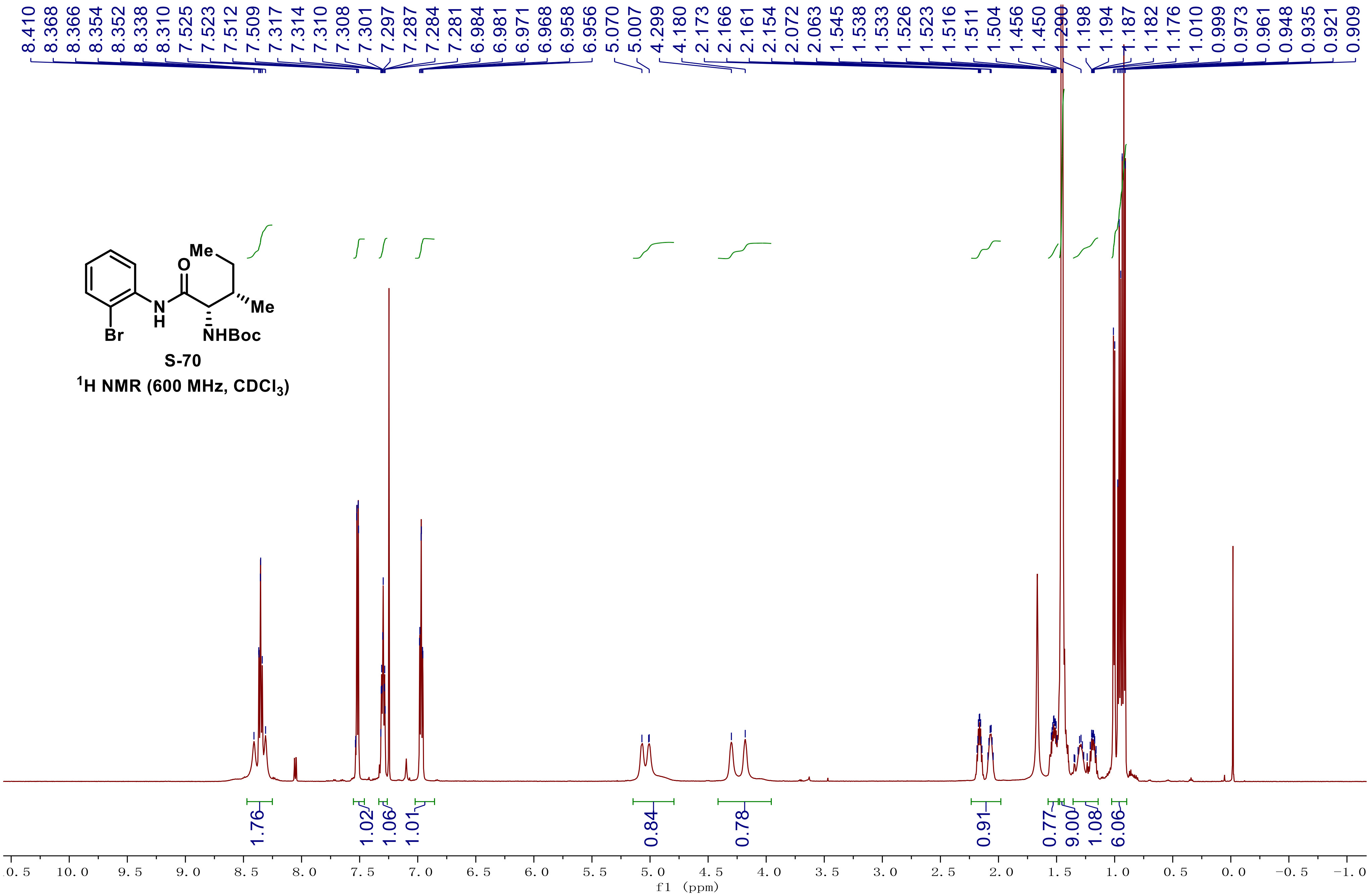

S-70

^{1}H NMR (600 MHz, $CDCl_3$)

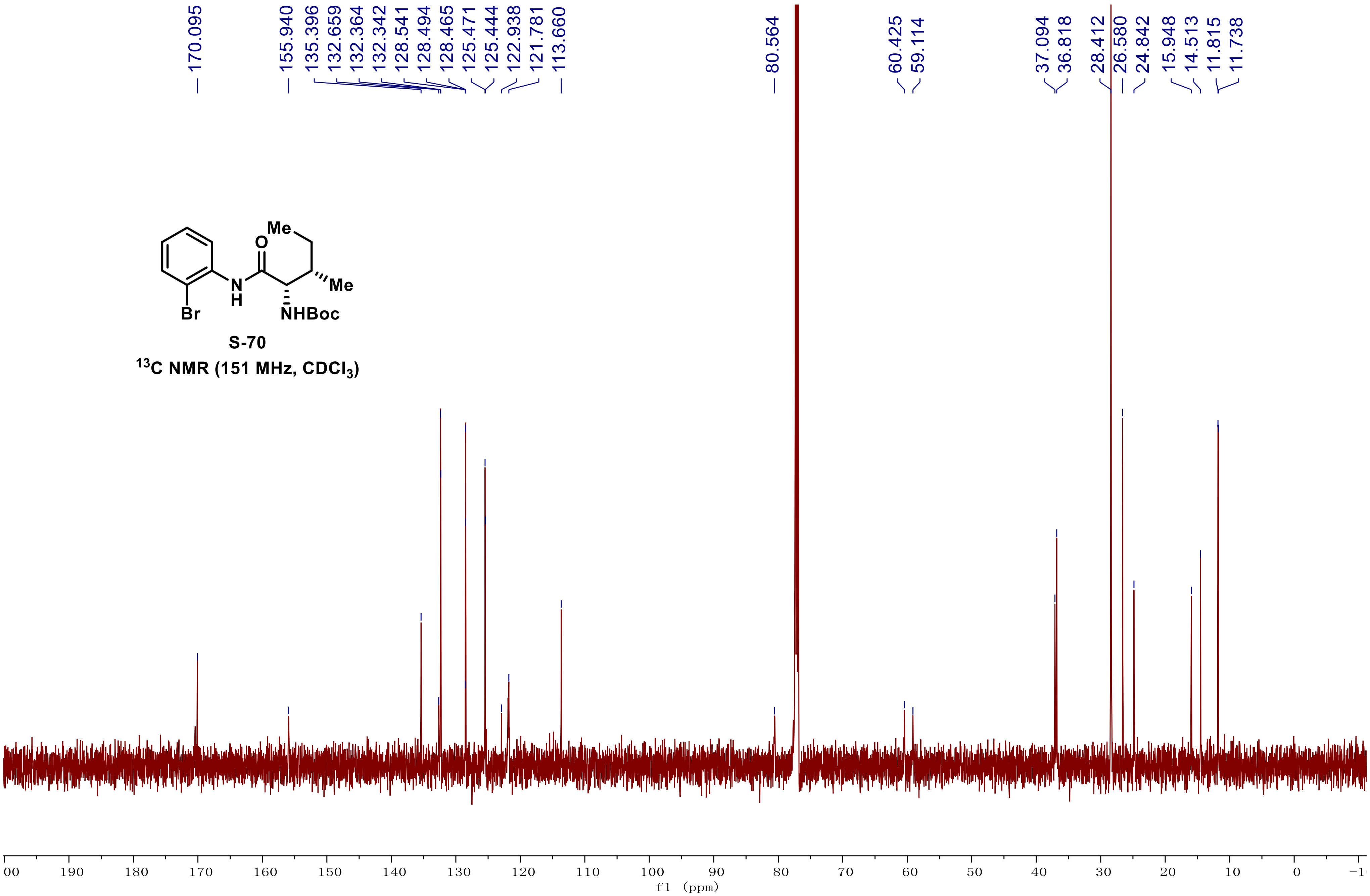

170.095
155.940
135.396
132.659
132.364
132.342
128.541
128.494
128.465
125.471
125.444
122.938
121.781
113.660
80.564
60.425
59.114
37.094
36.818
28.412
26.580
24.842
15.948
14.513
11.815
11.738
Me
O
N
H
Br
Me
NHBoc
S-70
^{13}C NMR (151 MHz, $CDCl_3$)
f1 (ppm)
